# Implementation of resource-efficient fetal echocardiography detection algorithms in edge computing

**DOI:** 10.1371/journal.pone.0305250

**Published:** 2024-09-23

**Authors:** Yuchen Zhu, Yi Gao, Meng Wang, Mei Li, Kun Wang

**Affiliations:** 1 School of Information Engineering, China University of Geosciences, Beijing, China; 2 Shijiazhuang Obstetrics and Gynecology Hospital, Shijiazhuang, China; 3 Hebei Maternity Hospital, Hebei, China; Shanghai Maritime University, CHINA

## Abstract

Recent breakthroughs in medical AI have proven the effectiveness of deep learning in fetal echocardiography. However, the limited processing power of edge devices hinders real-time clinical application. We aim to pioneer the future of intelligent echocardiography equipment by enabling real-time recognition and tracking in fetal echocardiography, ultimately assisting medical professionals in their practice. Our study presents the YOLOv5s_emn (Extremely Mini Network) Series, a collection of resource-efficient algorithms for fetal echocardiography detection. Built on the YOLOv5s architecture, these models, through backbone substitution, pruning, and inference optimization, while maintaining high accuracy, the models achieve a significant reduction in size and number of parameters, amounting to only 5%-19% of YOLOv5s. Tested on the NVIDIA Jetson Nano, the YOLOv5s_emn Series demonstrated superior inference speed, being 52.8–125.0 milliseconds per frame(ms/f) faster than YOLOv5s, showcasing their potential for efficient real-time detection in embedded systems.

## Introduction

Echocardiography has significant advantages such as portability, the absence of ionizing radiation, high temporal resolution, and low costs. Accurate and reliable echocardiographic assessment is the premise of high-quality clinical decision-making [1]. Fetal echocardiography, for example, has been widely used. It can accurately diagnose value of echocardiography for fetal congenitally unguarded tricuspid valve orifice [2] and has positive effects on reducing maternal anxiety [3]. Over the past decade, there has been an increasing application of artificial intelligence technology in cardiovascular imaging, including echocardiography, as it may reduce medical costs and avoid unnecessary tests [4]. It may be increasingly advantageous to introduce artificial intelligence technology for the identification of echocardiography images [5, 6]. However, there is a relative lack of research on its application in fetal echocardiographic image recognition [7, 8]. Fetal Echocardiography images have lower resolution and signal-to-noise ratio compared to ordinary echocardiography images, resulting in poor image quality and blurred object edges. Only internal echo signals of objects can be obtained, making it difficult to directly recognize objects. In addition, the fetus can freely rotate and roll over in the uterus, making its posture and position difficult to predict, which increases the difficulty of recognition. Accuracy is highly dependent on physician experience, making it difficult to adopt in primary care hospitals.

Traditional machine learning, an early branch of artificial intelligence, saw rapid advancement through algorithms like decision trees, which seek to minimize information entropy based on information theory; support vector machines, rooted in statistical learning; Bayesian classifiers, derived from Bayesian decision theory; and ensemble learning, which merges multiple learners to tackle tasks. Despite achieving notable results in specific applications, these traditional algorithms face challenges in universality and intelligence. In recent years, neural networks have emerged as a groundbreaking development in the field of machine learning. The first artificial neuron model, the MCP neuron, was conceptualized in 1943 by Warren McCulloch and Walter Pitts. They examined the connectivity of human brain neurons to develop this model. Connectionism, underpinned by neural networks, became a dominant technique until 1986. In that year, D.E. Rumelhart and others [9], invented the backpropagation algorithm, enabling neural networks to address a multitude of practical issues effectively. In 2006, Geoffrey Hinton unveiled deep belief networks in his paper titled "A fast learning algorithm for deep belief net" [10]. This development addressed the training challenges of deep neural networks and spurred swift progress in neural network algorithms. In 2009, Professor Li Fei-Fei of Stanford University initiated the ImageNet dataset [11], facilitating the development of seminal convolutional neural networks such as AlexNet [12], GoogLeNet [13], VGGNet [14], ResNet [15], and SENet [16] over the subsequent eight years, thus significantly propelling the advancement of deep learning technology. Deep learning models have demonstrated remarkably diverse and impressive performance in various subfields of artificial intelligence, such as sustainable urban development, the evolution of broadcasting, and the advancement of radio technology [17–19]. The integration of these AI technologies with real-life applications has achieved state-of-the-art results.

In recent years, computer vision technology has been developing at a rapid pace, with object detection algorithms becoming a research hotspot. Object detection algorithms can be roughly divided into two categories: two-stage object detection algorithms and single-stage object detection algorithms. Two-stage object detection algorithms are renowned for their high accuracy. Representative algorithms include R-CNN [20], Fast R-CNN [21], and Mask R-CNN [22]. However, the detection speed of these algorithms is relatively slow, making them unsuitable for deployment on embedded terminals to perform real-time object detection tasks. In contrast, single-stage object detection algorithms have garnered attention for their fast detection speed. These algorithms use regression-based methods to directly predict object categories and locations in a single step. Well-known single-stage object detection algorithms include SSD (Single Shot MultiBox Detector) [23] algorithm, RetinaNet [24], and YOLO (You Only Look Once) [25–27] algorithm. Among them, the YOLO algorithm has evolved over time, and multiple versions have been released.

Multiple convolutional neural network (CNN) architectures have recently been put forward to handle numerous challenges, and they boast improvements in model lightness and inferencing speed on embedded devices. These architectures include efficient convolutional neural networks that implement deep convolution structures, such as SqueezeNet [28], EffcientNet [29], MobileNets [30, 31], and GhostNet [32]. MobileNetV3, a new iteration introduced by Howard,A and his team at Google Brain, is the follow-up to MNASNet [33].

To enable real-time edge computing for fetal echocardiography, current detection models usually require video transmission to GPUs or TPUs with sufficient computational power, leading to high hardware costs. Moreover, severe network latency during data transmission can cause significant frame loss. This can result in the omission of crucial information. Even though integrating embedded devices with adequate computational capabilities into ultrasound equipment can reduce network impact, the cost remains substantial. Therefore, it is crucial to research how to deploy efficient fetal heart detection algorithm models on embedded terminals for edge computing. By integrating embedded devices equipped with efficient algorithms into ultrasound instruments, a direct connection between the ultrasound machine and the embedded device is established. This eliminates network and external influences and ensures smooth operation. As these algorithms are efficient, they significantly lower the hardware requirements for embedded devices, thereby reducing costs.

The main contributions of this research are:

1. We have provided a dataset named FE-Section Detection (FE-SD). The dataset was collected from case data at the Hebei Maternity Hospital, ensuring that it originates from real clinical settings. All images in the dataset were preprocessed to a consistent size and annotated for training the experimental models.
2. The backbone of the YOLOv5s model was optimized using MobilenetV3 methods. Model pruning and inference optimization were also performed. Five lightweight models were obtained according to model size. We collectively refer to these five lightweight models as the yolov5s_emn (Extremely Mini Network) series. Training results show that these methods greatly reduced training parameters and model complexity.
3. The YOLOv5s_emn Series models have undergone inference and recognition accuracy test on the NVIDIA Jetson Nano embedded platform. These tests indicate that the series consistently delivers inference speeds of between 52.8 and 125.0 milliseconds per frame, and it exhibits commendable recognition accuracy.

## Materials and methods

### Ethics statement

This study was approved by the Ethics Committee of Shijiazhuang fourth medical Hospital, written informed consents were signed by all patients. Shijiazhuang fourth Hospital medical Ethics Committee. Approval number: No.20220070.

### Dataset

The dataset utilized for fetal echocardiography image recognition in this research was sourced from the medical records of Hebei Maternity Hospital from January 1, 2017 to June 15, 2022.The dataset is available as S1 and S2 Datasets. This study was approved by the Ethics Committee of Shijiazhuang Fourth Medical Hospital, and written informed consent was obtained from all patients. We started accessing the dataset for research purposes in August 2022.A total of 2,798 fetal echocardiography images were collected. For deep learning training, we divided the ultrasound images into four categories: outflow tract section, abdominal transverse section, four-chamber heart section, and three-vessel section. We used the labeling tool LabelMe [34] to label all the images. Next, we randomly divided the image data of each type into training, validation, and testing subsets following a 7:2:1 distribution (for the ’Three vessels’ category, which had fewer data, we ensured the quantity of the test set by dividing approximately in a 6:2:2 ratio). The distribution of the dataset samples is shown in Table 1. The images in the dataset were preprocessed before training by resizing them to 640x640 pixels.

**Table 1:** Dataset sample distribution.

Class	Train	Validation	Test
Abdomen	772	219	110
Four-chamber	923	262	131
Outflow Tract	192	50	25
Three vessels	73	20	20
Total	1960	551	286

### Algorithm-related YOLOv5s

The findings from the paper "YOLO9000: Better, Faster, Stronger" [35] have shown that One-Stage Detection achieve faster detection speeds compared to Two-Stage Detection, allowing for real-time or near-real-time object detection. Consequently, our research direction is centered around One-Stage Detection models. To validate this, we conducted a series of experiments by training and inferencing several representative models from the YOLO family on our fetal cardiac echocardiography dataset in local machine. The experimental results, depicted in Table 2, YOLOv5s and YOLOv5n stand out from the other models, boasting substantially fewer parameters and swifter inference speeds. Between these two models, YOLOv5s showcases a superior Average Precision (mAP) compared to YOLOv5n. Taking into account the importance of selecting a model that demonstrates well-rounded and balanced attributes, we ultimately opted for YOLOv5s as the cornerstone architecture for our research endeavors.

**Table 2:** Comparison of YOLO family models.

Model	Parameters (million)	mAP@0.5	mAP@.5:.95	Processing speed (ms/f)
YOLOv3	61.54	0.961	0.62	38.4
YOLOv4	58.33	0.972	0.634	34.7
YOLOv5n	1.76	0.968	0.611	3.6
YOLOv5s	7.03	0.971	0.636	7.0
YOLOv5m	20.87	0.966	0.637	16.9
YOLOv5l	46.12	0.971	0.632	29.1
YOLOv5x	86.24	0.965	0.627	49.4

**Fig 1:**
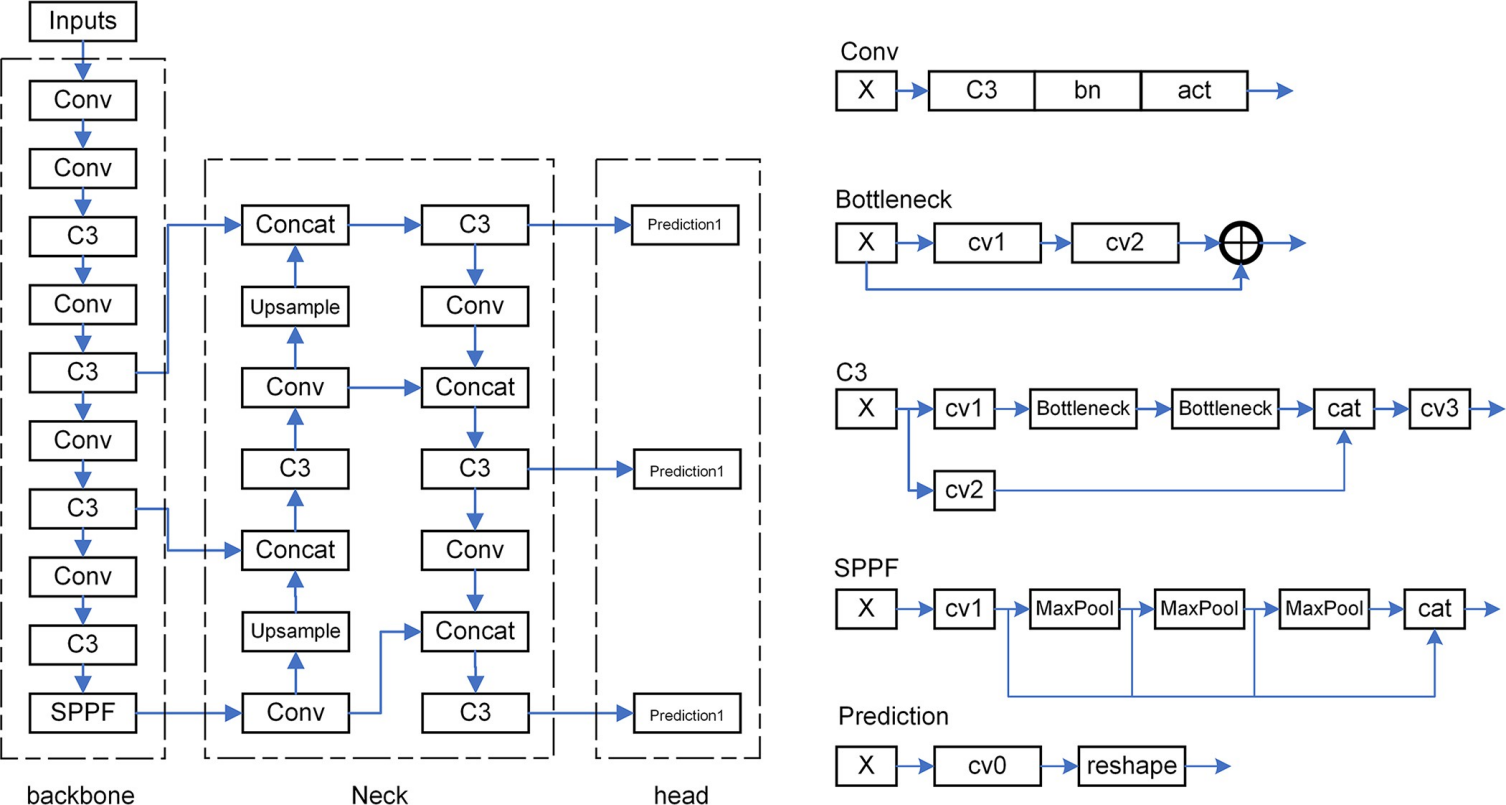
The overall structure of the YOLOv5s model. The YOLOv5s framework is structured around the input module, the backbone, the neck, and the head. Within its architecture, it incorporates key elements like the C3 module, conventional convolutions (Conv), bottlenecks, SPPF (Spatial Pyramid Pooling—Fast), and prediction layers.

The overall structure of the YOLOv5s model is shown in Fig 1. This algorithm increases Mosaic data enhancement, adaptive anchor frame calculation, and adaptive image scaling at the input end. The first convolutional layer of backbone network uses a convolution kernel size of 6, the first two convolutional layers of neck network use a kernel size of 1, while the rest of the convolutional layers employ a kernel size of 3. In C3 structure Bottleneck Blocks are employed to enhance the fusion of information from different network levels. It integrates Conv structure and C3 structure detection algorithms at the backbone network end. At the neck network, a Concat layer is often inserted between the Backbone and the final Head output layer, which greatly improves both speed and precision. Therefore, it is suitable for the detection task of fetal echocardiography.

### Algorithm-related MobileNetV3

MobileNetV3 introduces a new non-linear feature, named h-swish, to replace the ReLU6 activation function used in MobileNetV2. The swish function has been instrumental in enhancing model accuracy; however, it comes with a significant computational overhead, as shown in Eq (1). On the other hand, the h-swish function reduces computational requirements, as defined in Eq (2). Moreover, h-swish offers higher accuracy and, unlike sigmoid and other activation functions, doesn’t suffer from saturation problems. Importantly, the computation speed of h-swish is noticeably faster than that of the swish and sigmoid functions.


swish(x)=x*σ(x) (1)

$$hswish(x)=\frac{ReLU6(x+3)}{6}$$ (2)

**Fig 2:**
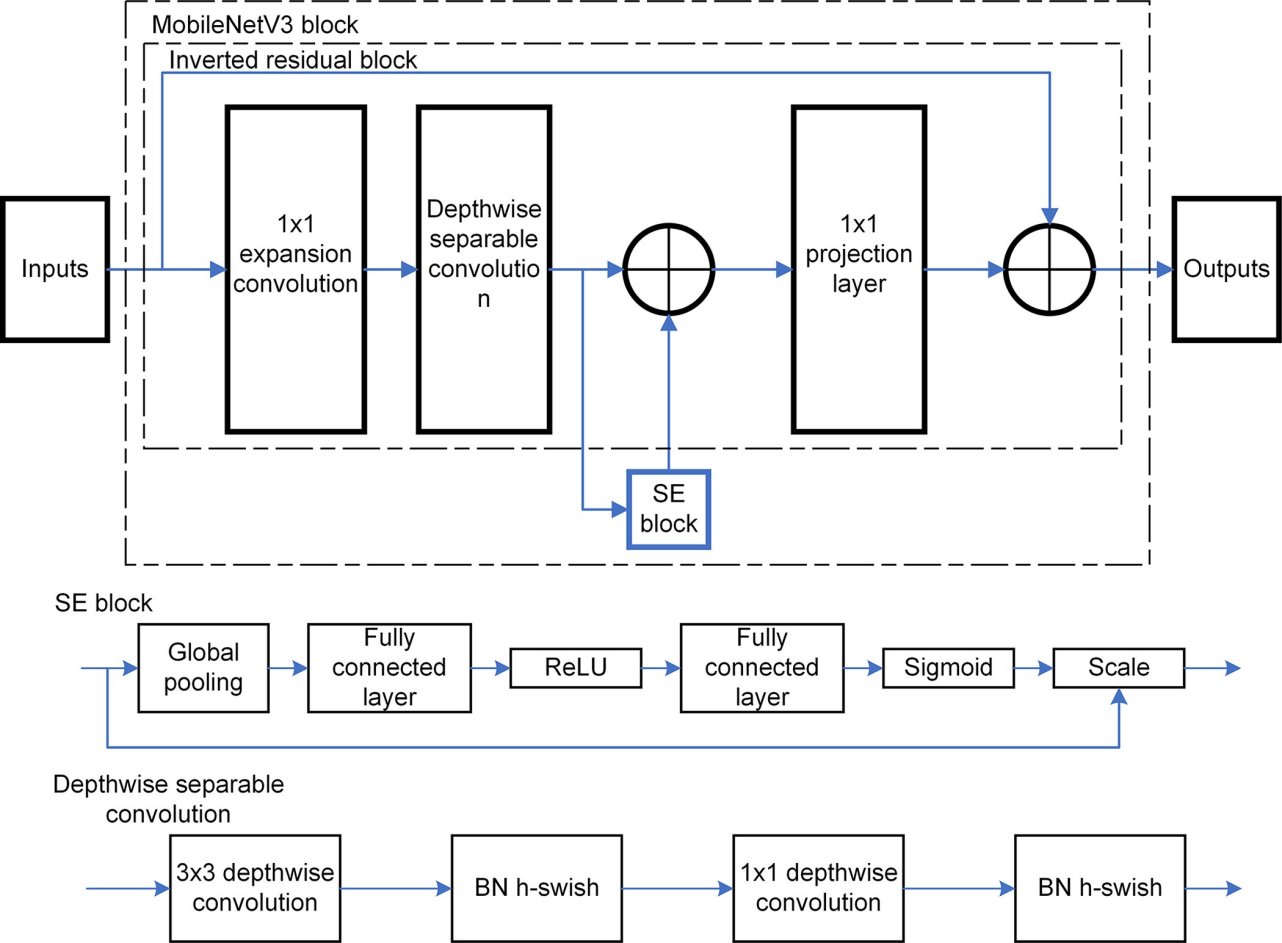
The overall structure of the MobileNetV3 block. The MobileNetV3 Block comprises an input layer, an expansion convolution, a Depthwise separable convolution, an SE (Squeeze-and-Excitation) block, a projection layer, and an output layer.

MobileNetV3 utilizes the Platform-Aware NAS method. Initially, it establishes the overall network architecture via a block-level search. Subsequently, it fine-tunes the network layers layer-by-layer using Netadapt [36]. These two processes play complementary roles in global and local search scopes respectively. Furthermore, MobileNetV3 has optimized the structures of the input and output stages, reducing the computational burden without compromising accuracy. As depicted in Fig 2, The MobileNetV3 block incorporates the idea of integrating the Squeeze-and-Excite (SE) module and the inverted residual block in parallel, a concept inspired by SENet [37]. The SE module is added on top of the inverted residual block. The SE module compresses the entire channel of input feature maps. The output scale can enhance important features and weaken nonessential ones, making the extracted features more directionally focused.

Given the strengths of MobileNetV3 mentioned above, we decided to employ this model to refine the YOLOv5s architecture. This enhancement is designed to maintain high accuracy while significantly reducing parameter count, aligning with our objectives.

### YOLOv5s_emn series

**Fig 3:**
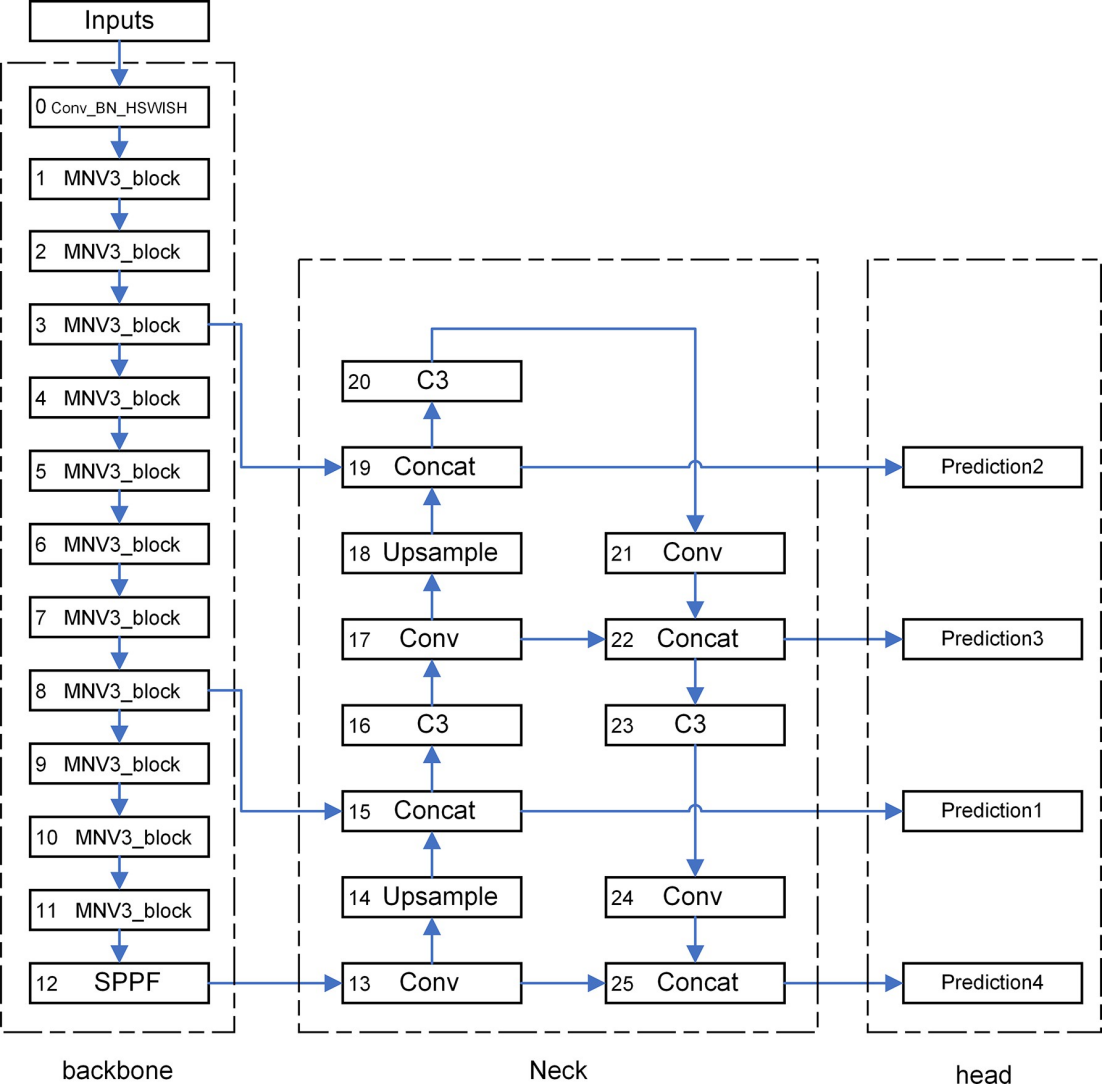
The overall structure of YOLOv5s_emn series model. The YOLOv5s_emn Series model’s framework consists of four components: the input module, the backbone module, the neck module, and the prediction module. The input module’s duty is to feed the fetal echocardiography image data into the model. The backbone module is designed to extract features from the fetal echocardiography image. The neck module facilitates the integration of the extracted features. Meanwhile, the prediction module is responsible for predicting the four types of fetal echocardiography images.

This section introduces the overall framework of the YOLOv5s_emn Series model and the classification of models proposed in this study. The architecture of the YOLOv5s_emn Series model is illustrated in Fig 3. In the Backbone section, we replace the YOLOv5s Backbone with a combination of MobileNetV3 blocks and SPPF layers. First, the image is processed by the MobileNetV3 algorithm, and then a fixed-dimensional adaptive output is produced through the SPPF. SPPF is an optimized version of the SPP [38] method, as developed by the author of YOLOV5. While its purpose and calculation formula are the same, but the structure is slightly modified. Compared to SPP, SPPF reduces the computational load and enhances the model’s speed, the Spatial Pyramid Pooling method connects convolutional layers with outputs of undefined sizes to a fixed-size fully connected layer. On one hand, it can generate a fixed-length output that adapts to different image input sizes, eliminating the need for preprocessing to unify image sizes. On the other hand, it can extract spatial feature data at various scales, thereby enhancing the model’s robustness to different spatial arrangements and object distortions. Originally proposed under specific conditions, the method initially lacked universality. Eq (4) is a modified version that takes into account the input size (w), output size (n), pooling window size (k), stride (s), and padding (p).


$$k=s=ceil(\left[\frac{w}{n}\right])$$ (3)

$$p=floor(\left[\frac{k*n-w+1}{2}\right])$$ (4)

In the inference optimization section, we refined the hierarchical structure by replacing the original C3 module in the prediction stage with a Concat module. This change reduces the need to calculate one C3 module for each prediction, which in turn decreases the number of training parameters and computational layers, allowing for a smaller model size. Additionally, we increased the number of predictions bounding boxes from the original three in YOLOV5 to four, which correspond to the Concat layers at model layers 15, 19, 22, and 25. This modification enables the model to predict bounding boxes of four different sizes, enhancing flexibility and detection accuracy, and allowing for the selection of bounding boxes based on the specific scenario.

In the model pruning section, we can apply pruning techniques to both the backbone and neck sections of the model to decrease the number of training parameters further. Specifically. In the backbone section, we can eliminate some of the MobileNetV3 blocks. By selecting only 2–3 out of the four prediction boxes for inference, we can then prune the unnecessary layers in the neck section that are not required for computation. These two strategies contribute to a significant reduction in the number of training parameters, resulting in a more lightweight model.

This study aims to adapt the model for real-world production use, considering the varying computing capabilities of different embedded devices. We have categorized YOLOv5s_emn into five distinct versions—YOLOv5s_emns, YOLOv5s_emnm, YOLOv5s_emnl, YOLOv5s_emnx, and YOLOv5s_emnxx—based on model size, computational resources, and accuracy requirements. The specific layers and prediction boxes for each model version are detailed in Table 3.

**Table 3:** YOLOv5s_emn series model parameter.

Model	Parameters(million)	Model Size (MB)	layers	Prediction boxes
YOLOv5s_emnxx	1.28	2.86	0–25	Prediction2、3、4
YOLOv5s_emnx	0.92	2.11	0–22	Prediction2、3
YOLOv5s_emnl	0.81	1.89	0–19	Prediction1、2
YOLOv5s_emnm	0.44	1.12	0–9, 12–22	Prediction2、3
YOLOv5s_emns	0.33	0.93	0–9, 12–19	Prediction1、2

### Implemented on edge computing platforms

The study utilizes a deep learning project developed with the PyTorch toolkit. to train the YOLOv5, YOLOv5s_ghost, YOLOv5s_Efficient_B0 and five models of YOLOv5s_emn Series. Subsequently, the models are deployed on the Jetson Nano embedded terminal and performs inference testing.

Initially, the models are trained using deep learning techniques. Once training is completed, the optimal weights for each model are chosen based on the results, resulting in the pt-format weights and the performance metrics from the training process. These pt-format weights are then transferred and deployed on the Jetson Nano development board. resulting in the pt-format weights and the performance metrics from the training process running on the embedded device.

### Model evaluation metrics

In this research, the model performance assessment metrics include precision, recall rate, mean Average Precision (mAP) with an IoU threshold of 0.5, single frame image inference time, the number of parameters, and model size. Precision is utilized to gauge the accuracy of model detection, as shown in Eq (5). Recall rate is utilized to assess the comprehensiveness of model detection, calculated as in Eq (6), where TP represents true positives, FN false negatives, and FP false positives. mAP signifies the overall performance across different confidence threshold levels, as defined in Eq (7).


$$Precision=\frac{TP}{TP+FP}$$ (5)

$$Recall=\frac{TP}{TP+FN}$$ (6)

mAP=AP(Precision,Recall) (7)

## Results and discussion

### Training and embedded platform

The model training employed 2 Tesla P4 GPUs with 8GB memory, Windows 10 64-bit OS, Python, and the PyTorch DL framework.

Model inference was carried out on the NVIDIA Jetson Nano development board. The board contains an NVIDIA Maxwell GPU with 128 CUDA cores and a quad-core ARM Cortex-A57 CPU with 4GB LPDDR4 memory. The Jetson Nano runs Ubuntu 18.04 OS and Python 3.6.

### Training parameters setting

The model training adheres to consistent parameter settings. It undergoes a training duration of 300 epochs with a batch size of 32. Pre-trained parameters are not utilized in any of the models. At the end of each epoch, the model is preserved and the top-performing model at the conclusion of the training session is chosen as the ultimate model.

### Training model results evaluation

**Fig 4:**
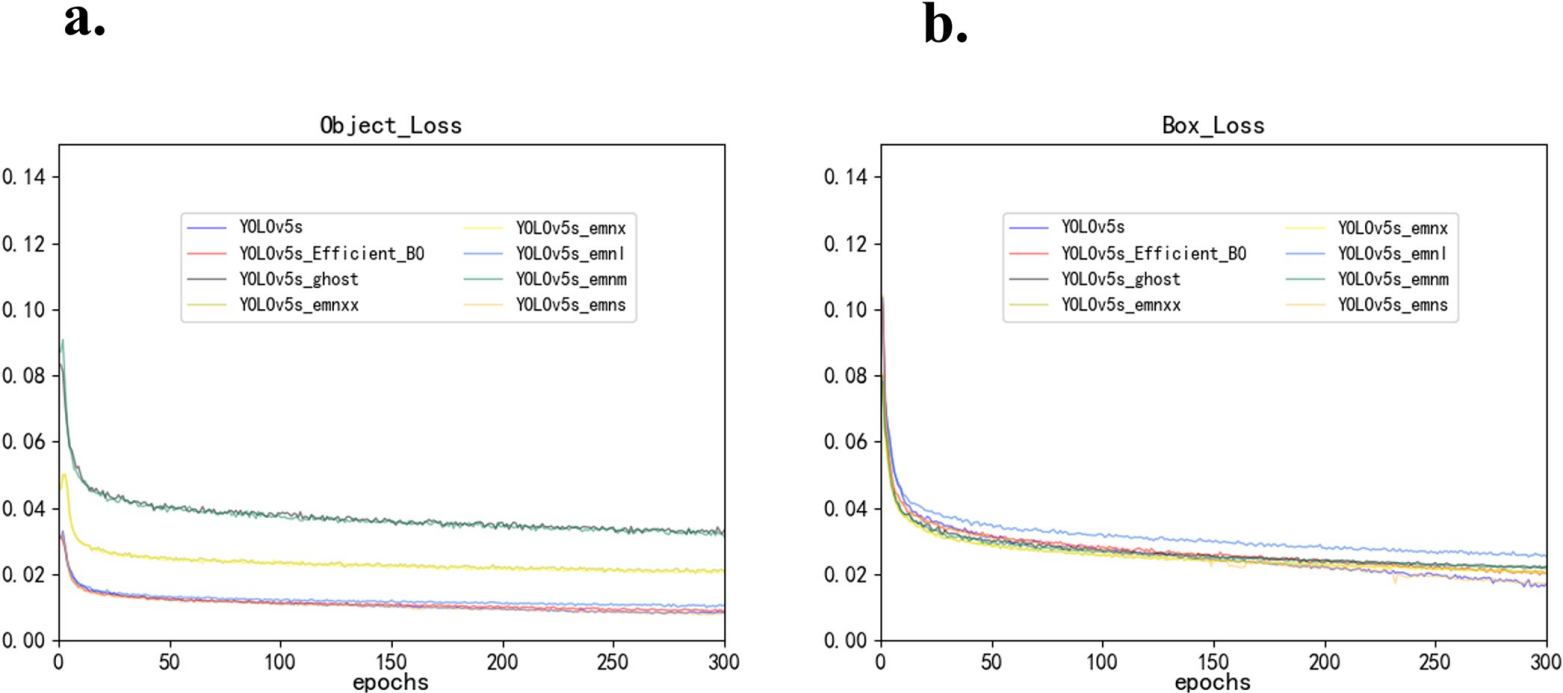
The tends of the bounding box loss and object loss. (a) bounding object loss, (b)bounding box loss.

In the experiment, as the training epochs progress, the decline in bounding box and the loss values from object detection or instance segmentation clearly signals a trend towards complete model convergence, as illustrated in Fig 4. This typically implies that the model’s performance on the target classification task is quite impressive, or at the very least, it performs well on the training data. These training results will be employed for further performance assessment.

**Fig 5:**
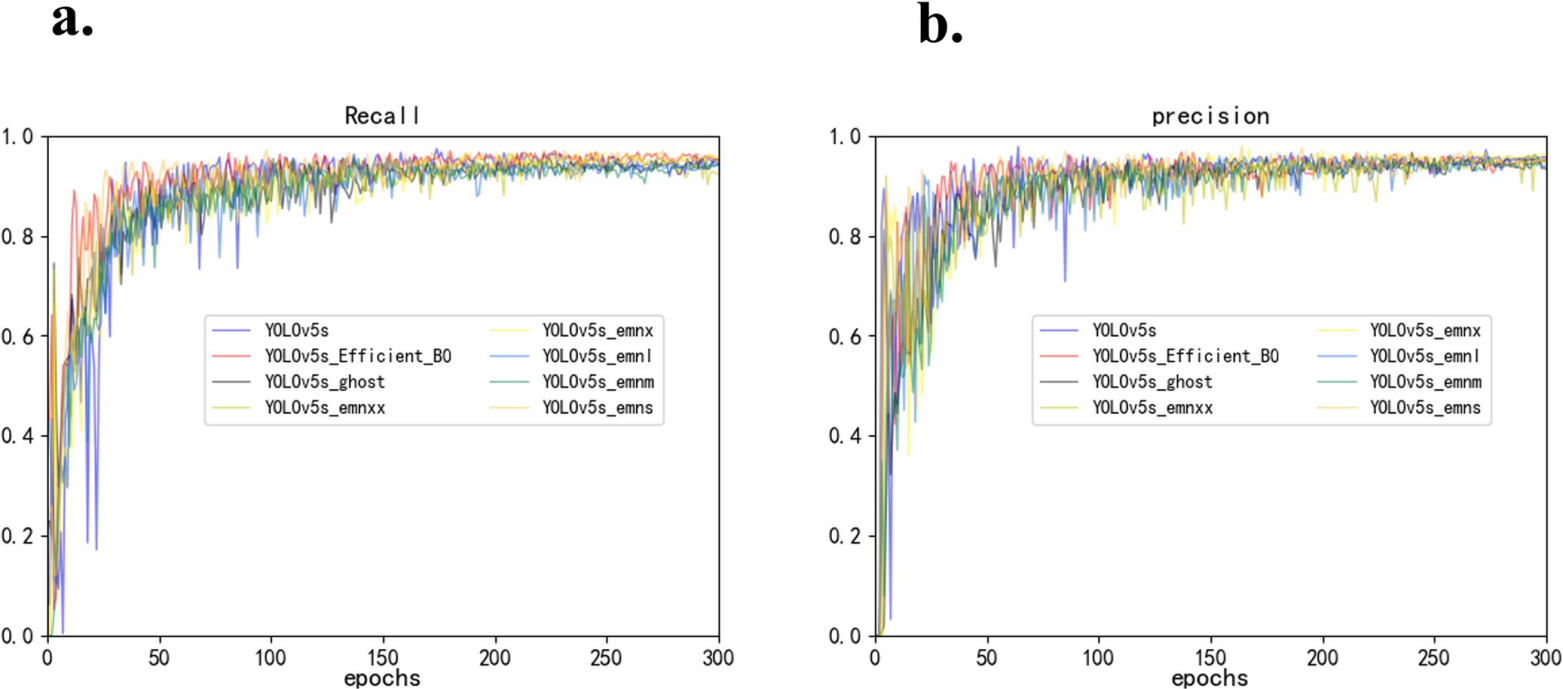
The tends of the he precision and recall rate. (a) Precision, (b)Recall rate.

Throughout the experiment, as the training epochs advance, the decreasing rate of the bounding box and the loss values from object detection or instance segmentation clearly demonstrates the model’s convergence, as specifically shown in Fig 5. This generally suggests that the performance of the model on the target classification task is quite remarkable, or at the very least, it performs well on the training data. Furthermore, the model’s precision and recall rates gradually improve with each training iteration. Generally, as the number of epochs increases, the precision of the model improves. After training for about 130 epochs, the values of precision and recall rates gradually stabilize, reaching a basic convergence, and the training progress hits a plateau. These training results will be employed for further performance assessment.

The training results are shown in Table 4. Compared with other models, the five models of YOLOv5_emn series exhibit superior performance in terms of parameter count and model size with no significant difference in precision and recall rate. It’s important to point out that the term ‘number of parameters’ refers to the total number of parameters in the model, specifically in the context of convolutional neural networks. The number of parameters is primarily by the weights in each convolutional layer. The number of parameters impacts memory usage, the rate of model initialization, and the model’s size, all of which can then subsequently influence the model’s performance in making inferences. The reduction of parameter numbers leads to the reduction of network complexity and convolutional network weight numbers, thereby reducing the computational complexity and making it easier to deploy on platforms with lower computing power, which meets our requirements.

**Table 4:** Model training performance result.

Model	layers	Parameters (million)	Model size (MB)
YOLOv5s	270	7.03	14.08
YOLOv5s_ghost	453	7.03	7.64
YOLOv5s_Efficient_B0	258	3.69	7.25
YOLOv5s_emnxx	287	1.28	2.86
YOLOv5s_emnx	258	0.92	2.11
YOLOv5s_emnl	230	0.81	1.89
YOLOv5s_emnm	224	0.43	1.12
YOLOv5s_emns	196	0.33	0.93

### Evaluation results and interpretation following model deployment

All models utilized in training were implemented on Jetson Nano for performance evaluation. The results of these tests are displayed in Table 5. The precision, recall rate, and mAp@0.5 of the YOLOv5s-emn Series model show no significant difference compared to other models. However, the inference speed is 96.7–168.9 milliseconds per frame(ms/f), which is significantly improved compared to other models. The YOLOv5s-emnx model exhibits exceptional performance metrics on the Jetson Nano. Based on the experimental results, the deployment of the YOLOv5s-emn series models promises to overcome the challenge of deploying deep learning algorithms in practical scenarios, which has been hindered by the relatively weaker image processing capabilities of edge devices.

**Table 5:** Deployment model performance result.

Model	Precision	Recall Rate	mAP@0.5	Processing speed (ms/f)
YOLOv5s	0.887	0.887	0.942	221.7
YOLOv5s_ghost	0.894	0.849	0.929	209.8
YOLOv5s_Efficient_B0	0.884	0.849	0.938	268.1
YOLOv5s_emnxx	0.893	0.829	0.922	168.9
YOLOv5s_emnx	0.922	0.877	0.946	127.4
YOLOv5s_emnl	0.884	0.884	0.952	118
YOLOv5s_emnm	0.901	0.836	0.939	107.5
YOLOv5s_emns	0.875	0.858	0.947	96.7

### Visualization of test results

**Fig 6:**
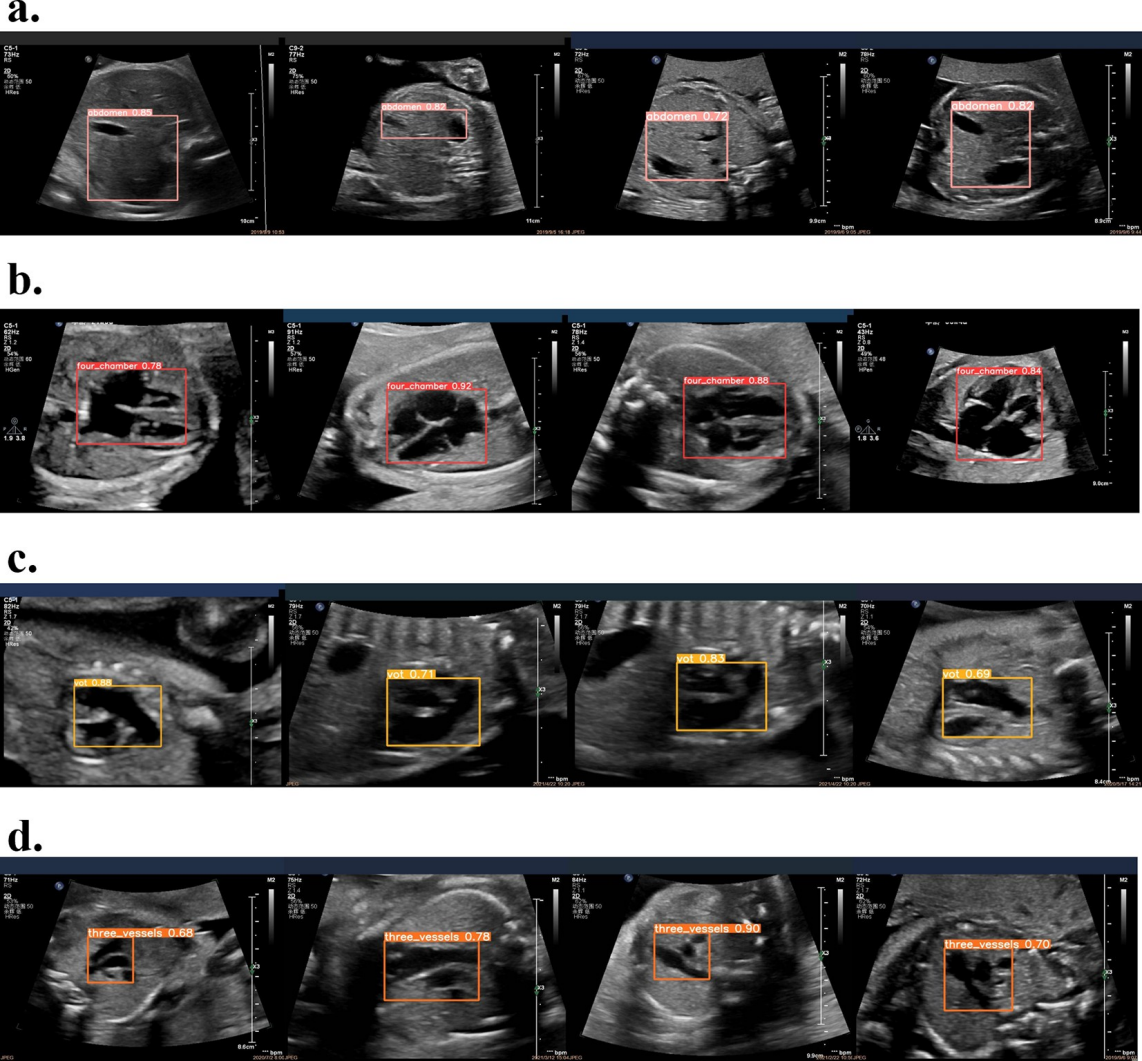
The result of four categories images. (a) Abdominal transverse section, (b)Four-chamber view of the heart, (c)Outflow tract section, (d) Three-vessel section. The label "abdomen" indicates the abdominal transverse section, the label "four_chamber" indicates the four-chamber view of the heart, the label "vot" indicates the outflow tract section, and the label "three_vessels" indicates the three-vessel view. The end of each classification result is attached with the classification confidence score.

The test results on Jetson Nano can be visualized. We choose to show the visualization prediction results of YOLOv5s_emns. The prediction results are shown in Fig 6, it clearly illustrates that the detection system is capable of precisely identifying the fetal echocardiography image within the video frame. The YOLOv5s-emn Series model has achieved good detection results under conditions such as speckle noise and artifacts in the image.

## Conclusion

This study aims to solve the problem that the image processing capability of edge devices is relatively low, and it is still difficult to deploy deep learning algorithms in actual application scenarios. We propose a lightweight fetal echocardiography detection series algorithm model called YOLOv5_emn Series. Replace the backbone, model pruning, optimizing inference and minimizing the number of parameters to decrease the overall size of the model. These results show that after optimizing three methods, The enhanced model implemented on the Jetson Nano terminal can significantly speed up the detection of fetal echocardiography fetal echocardiography images without significantly reducing the detection accuracy. This model contains five versions to choose from for deployment on embedded devices, which can meet the balance requirements between model size, computing resources, and accuracy in different scenarios. By implementing the target recognition model on the embedded device, this study achieves real-time detection of fetal echocardiography images and contributes to improving the level of medical intelligence. At present, our algorithm lacks the capability to discern the orientation of the fetal heart. Our subsequent research will prioritize addressing this limitation by integrating traditional visual processing techniques.

## Supporting information

S1 Dataset FE-section detection (FE-SD-1). (ZIP)

S2 Dataset FE-section detection (FE-SD-2). (ZIP)
