# Supplementary figures and images for: Implementation of resource-efficient fetal echocardiography detection algorithms in edge computing (part 2 of 4)
Source: PLoS One. 2024 Sep 23;19(9):e0305250. doi: 10.1371/journal.pone.0305250 (PMC11419364; doi:10.1371/journal.pone.0305250)

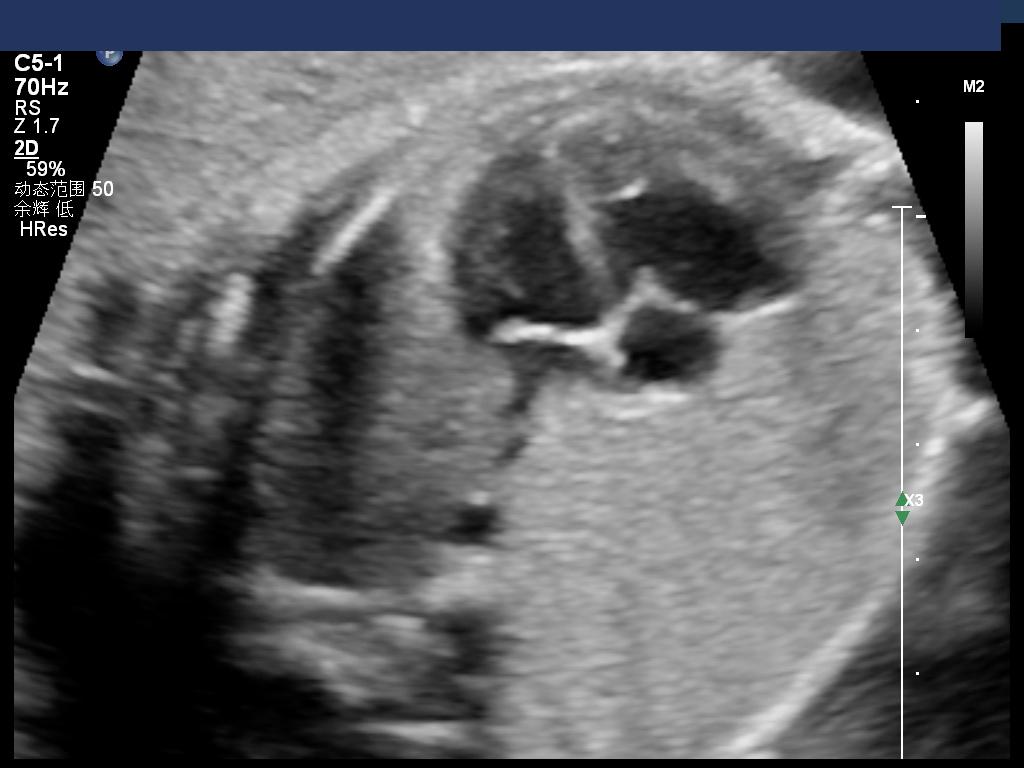

Supplement: S1 Dataset — (ZIP) [file pone.0305250.s001.zip › FE-SD-1/images/train_res/1076_fc.jpg]

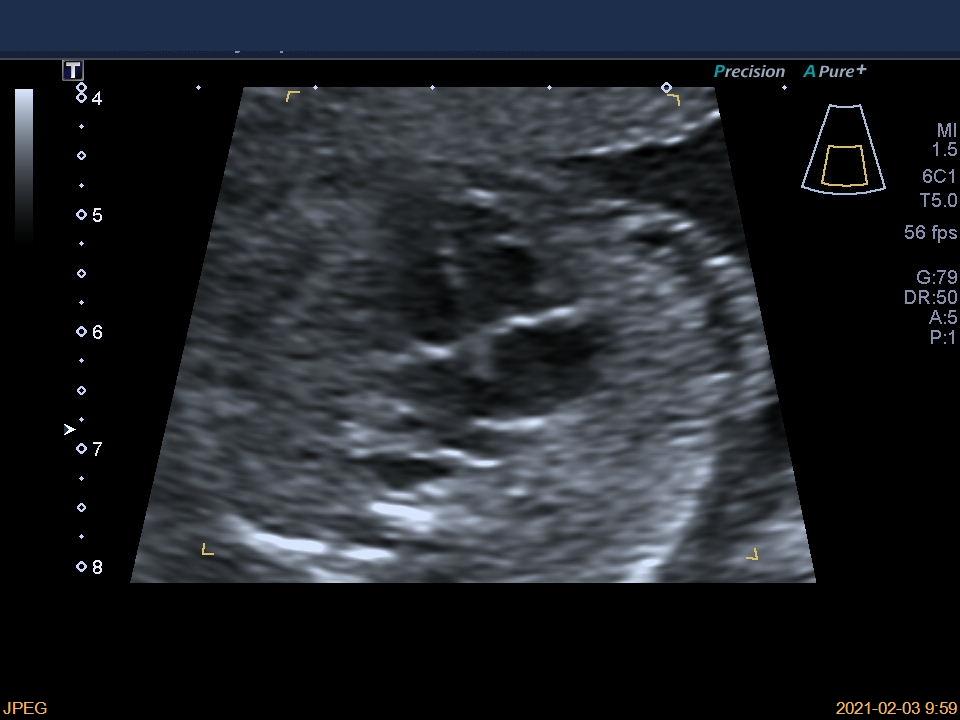

Supplement: S1 Dataset — (ZIP) [file pone.0305250.s001.zip › FE-SD-1/images/train_res/1078_fc.jpg]

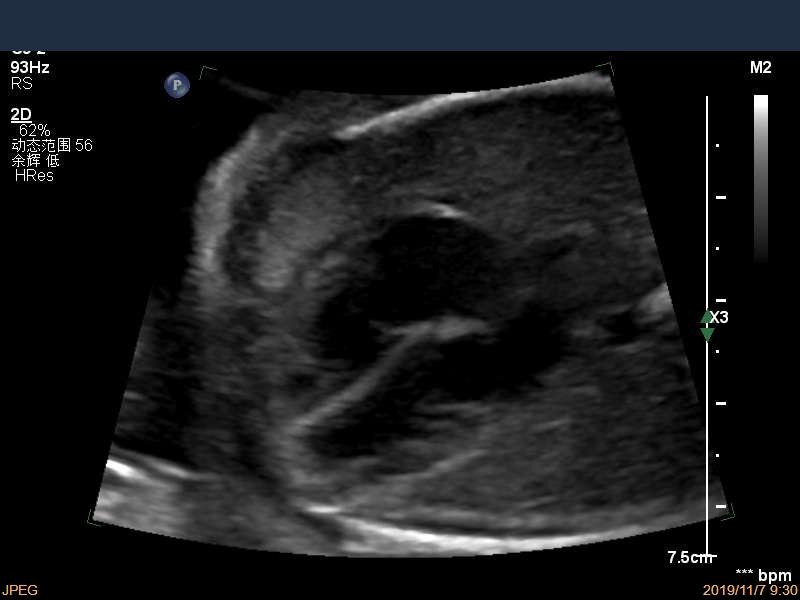

Supplement: S1 Dataset — (ZIP) [file pone.0305250.s001.zip › FE-SD-1/images/train_res/1079_fc.jpg]

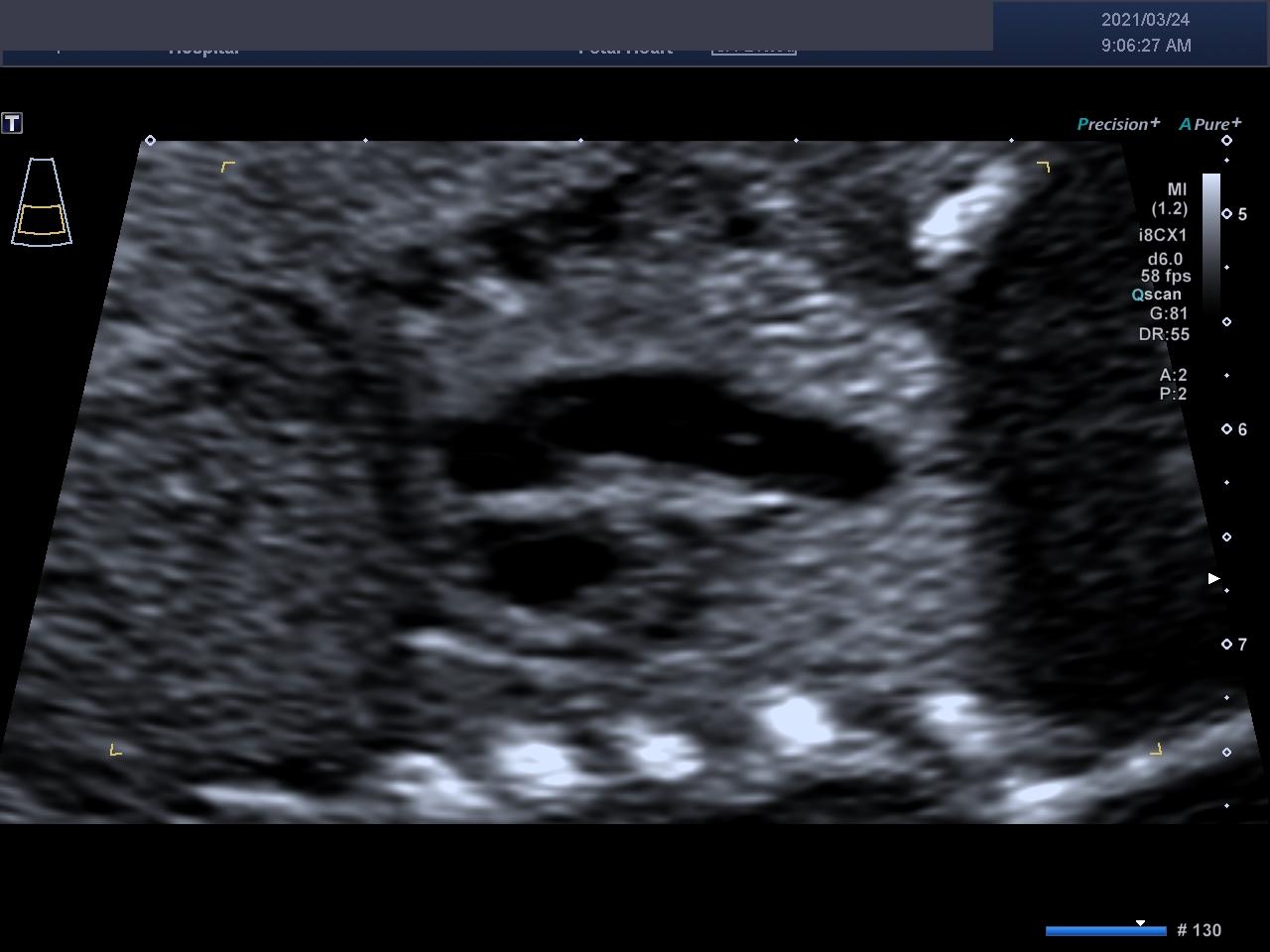

Supplement: S1 Dataset — (ZIP) [file pone.0305250.s001.zip › FE-SD-1/images/train_res/107_ro.jpg]

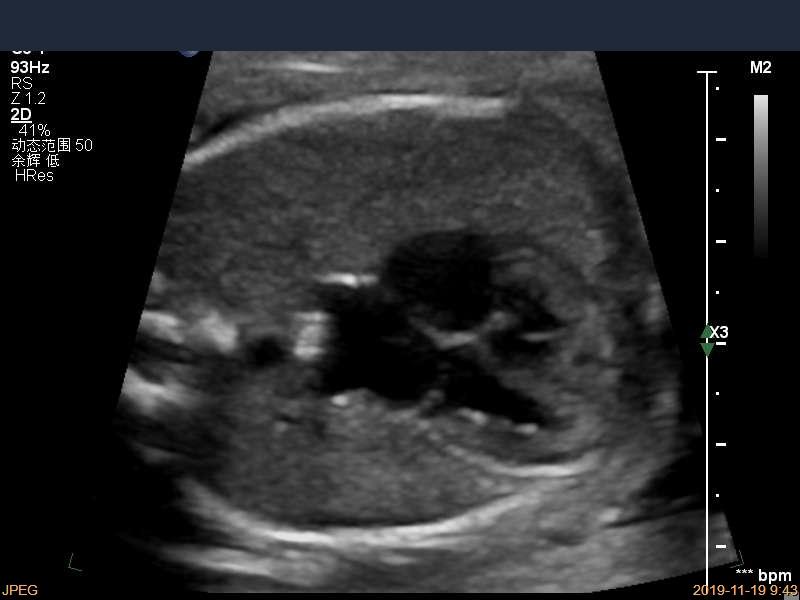

Supplement: S1 Dataset — (ZIP) [file pone.0305250.s001.zip › FE-SD-1/images/train_res/1080_fc.jpg]

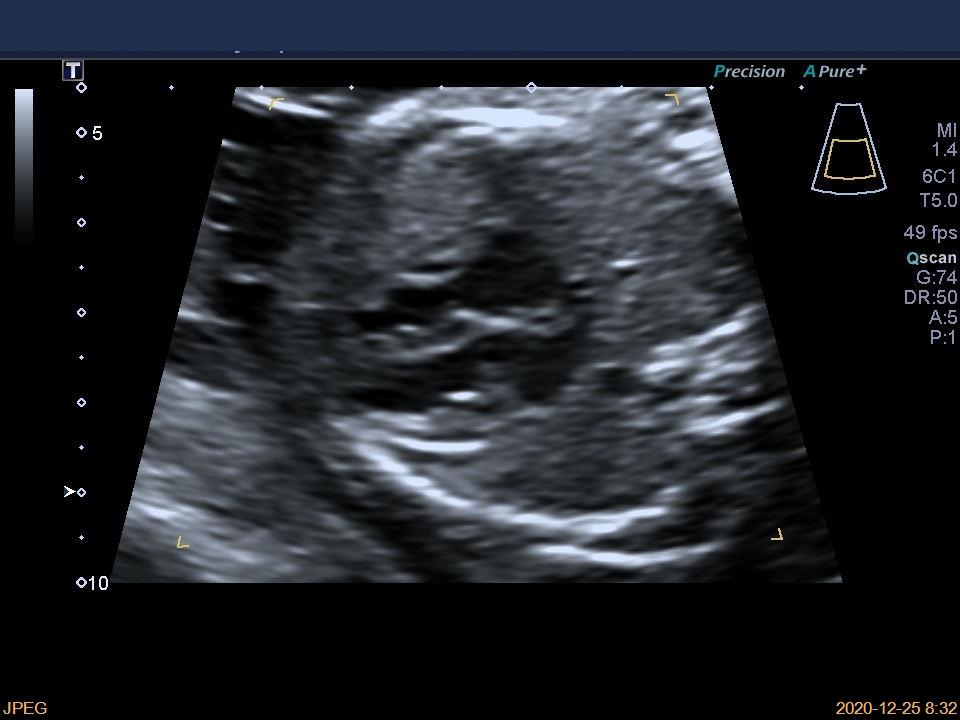

Supplement: S1 Dataset — (ZIP) [file pone.0305250.s001.zip › FE-SD-1/images/train_res/1081_fc.jpg]

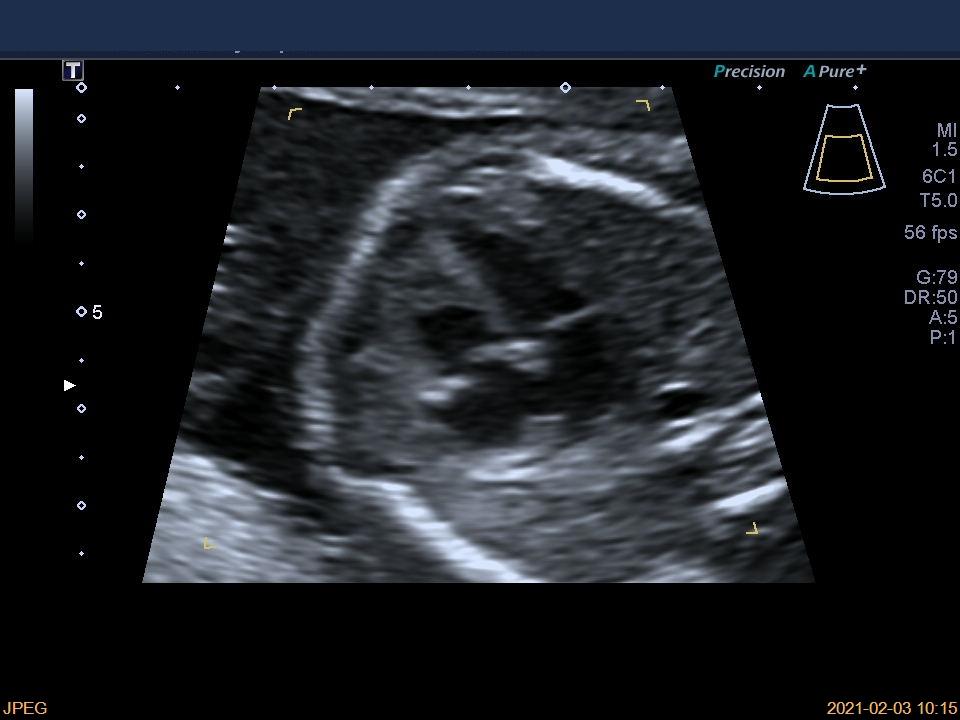

Supplement: S1 Dataset — (ZIP) [file pone.0305250.s001.zip › FE-SD-1/images/train_res/1082_fc.jpg]

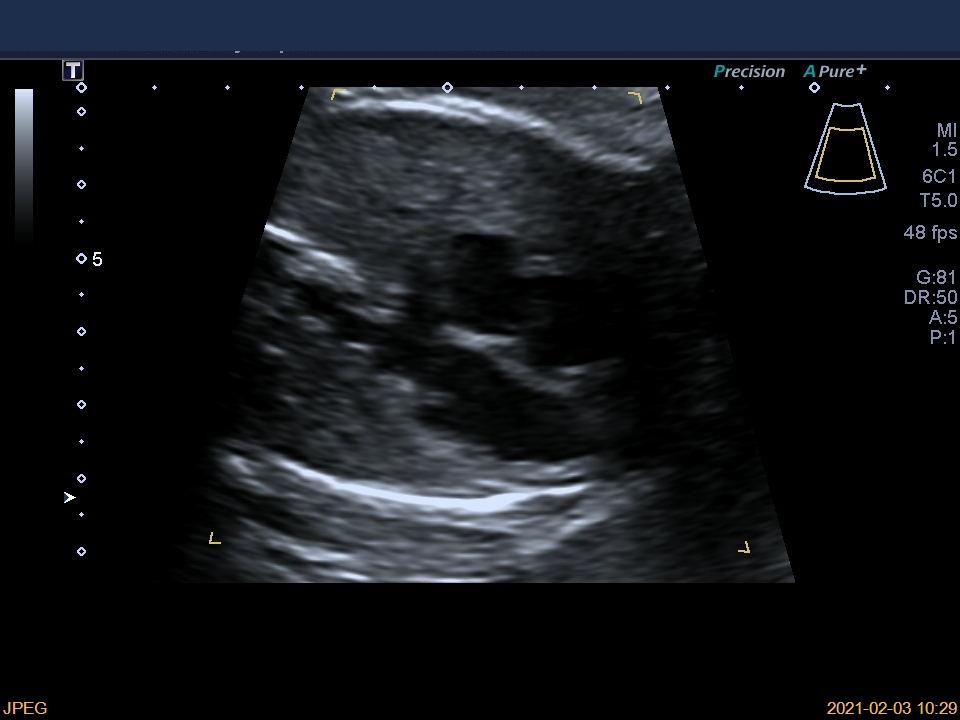

Supplement: S1 Dataset — (ZIP) [file pone.0305250.s001.zip › FE-SD-1/images/train_res/1084_fc.jpg]

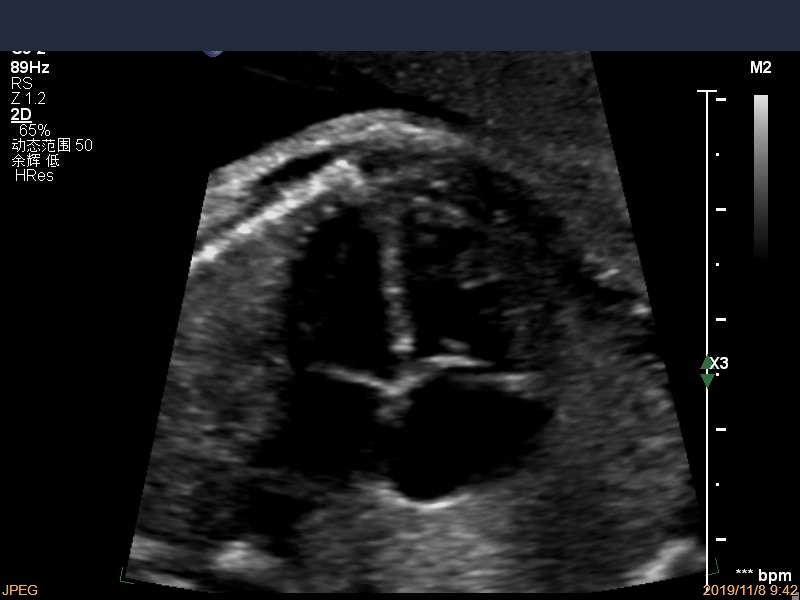

Supplement: S1 Dataset — (ZIP) [file pone.0305250.s001.zip › FE-SD-1/images/train_res/1085_fc.jpg]

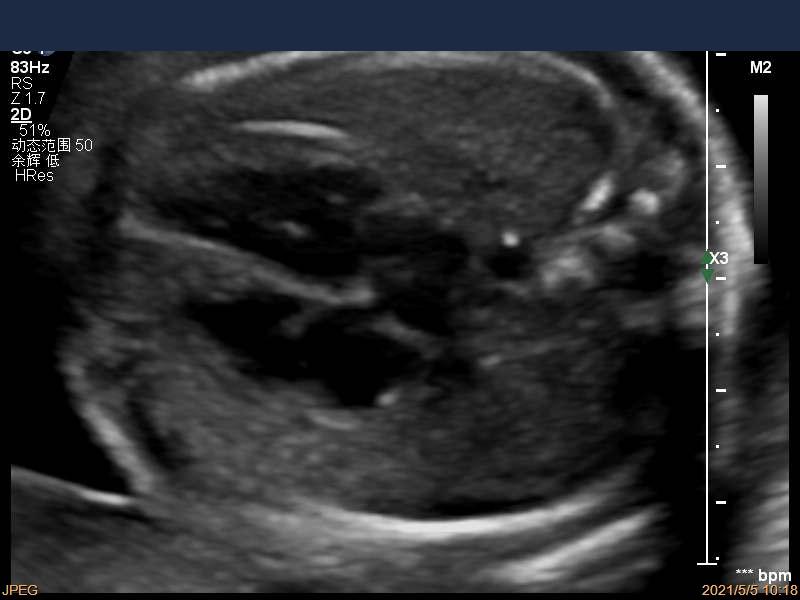

Supplement: S1 Dataset — (ZIP) [file pone.0305250.s001.zip › FE-SD-1/images/train_res/1086_fc.jpg]

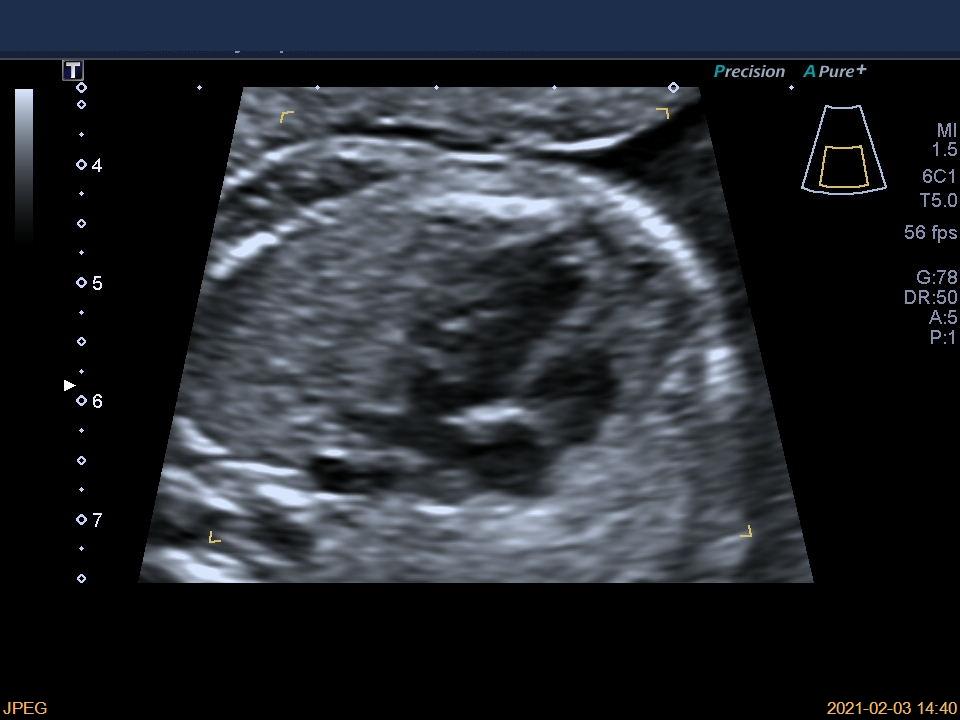

Supplement: S1 Dataset — (ZIP) [file pone.0305250.s001.zip › FE-SD-1/images/train_res/1089_fc.jpg]

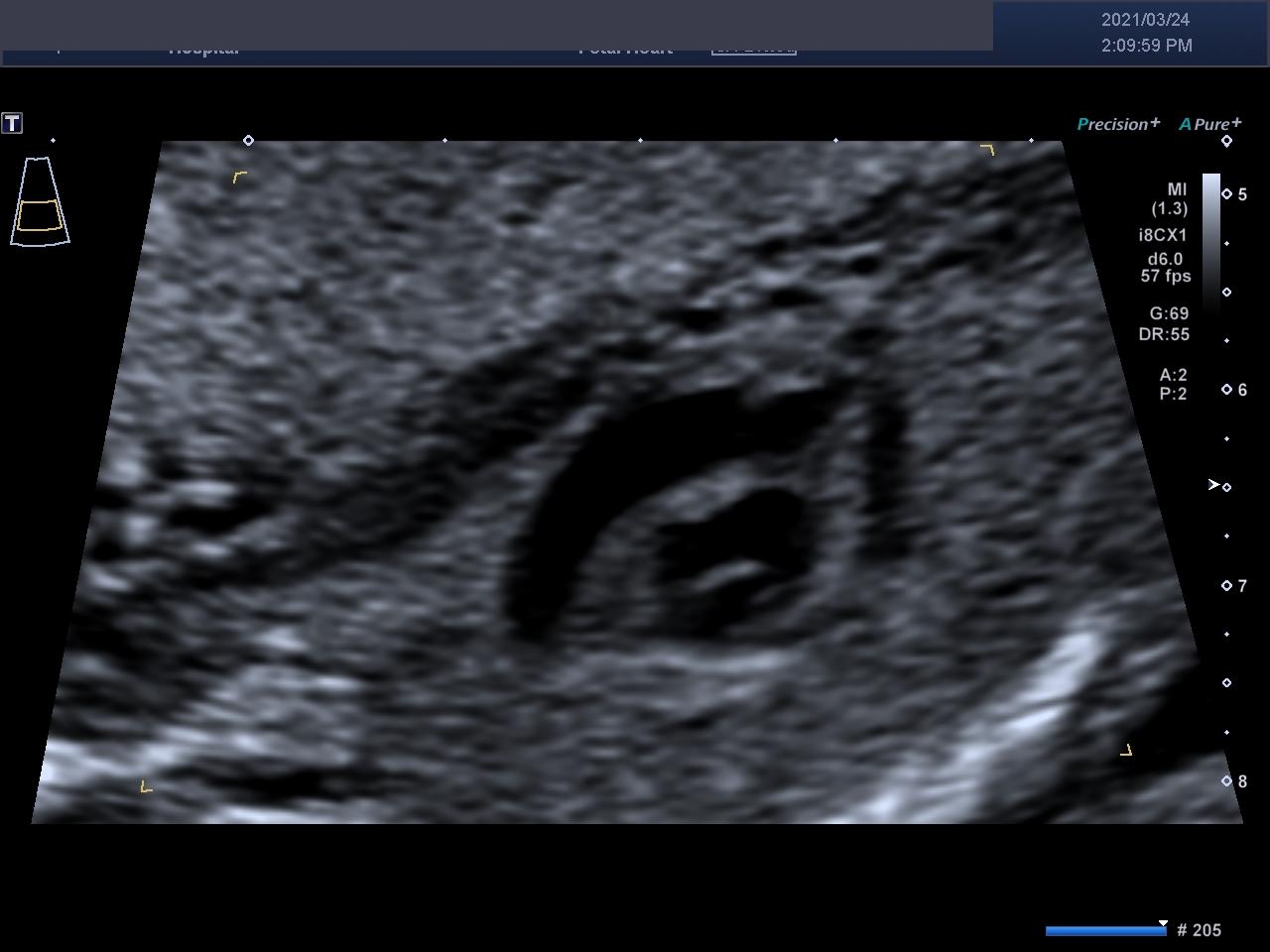

Supplement: S1 Dataset — (ZIP) [file pone.0305250.s001.zip › FE-SD-1/images/train_res/108_ro.jpg]

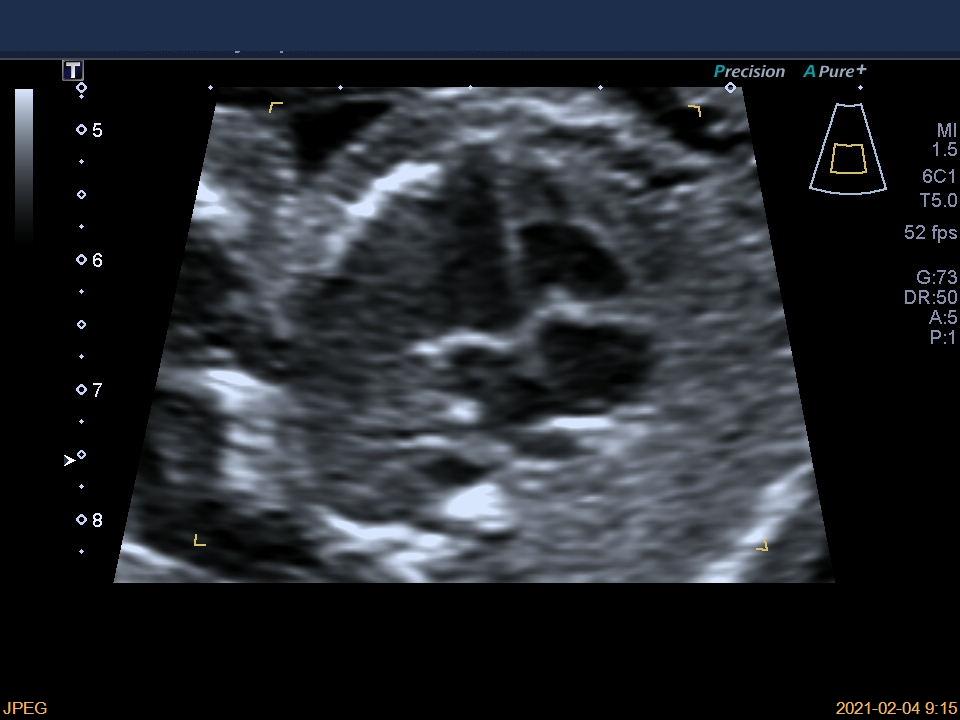

Supplement: S1 Dataset — (ZIP) [file pone.0305250.s001.zip › FE-SD-1/images/train_res/1090_fc.jpg]

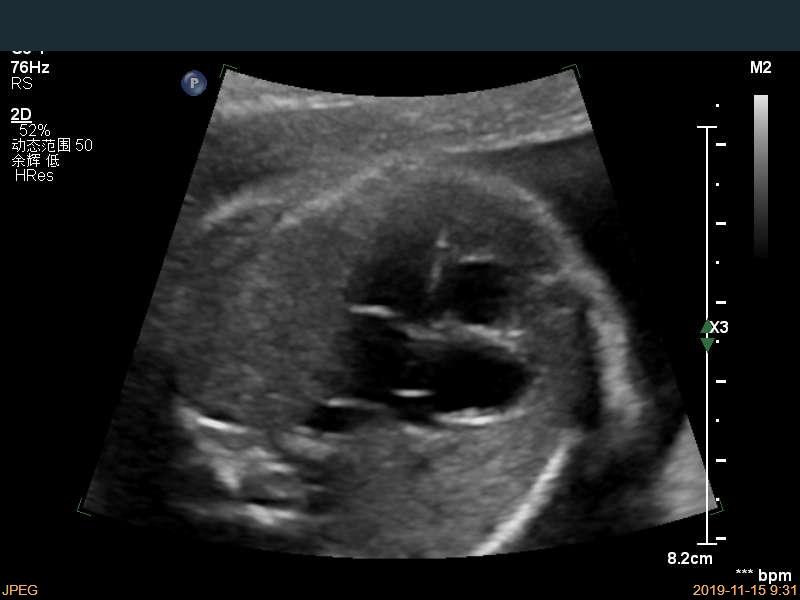

Supplement: S1 Dataset — (ZIP) [file pone.0305250.s001.zip › FE-SD-1/images/train_res/1094_fc.jpg]

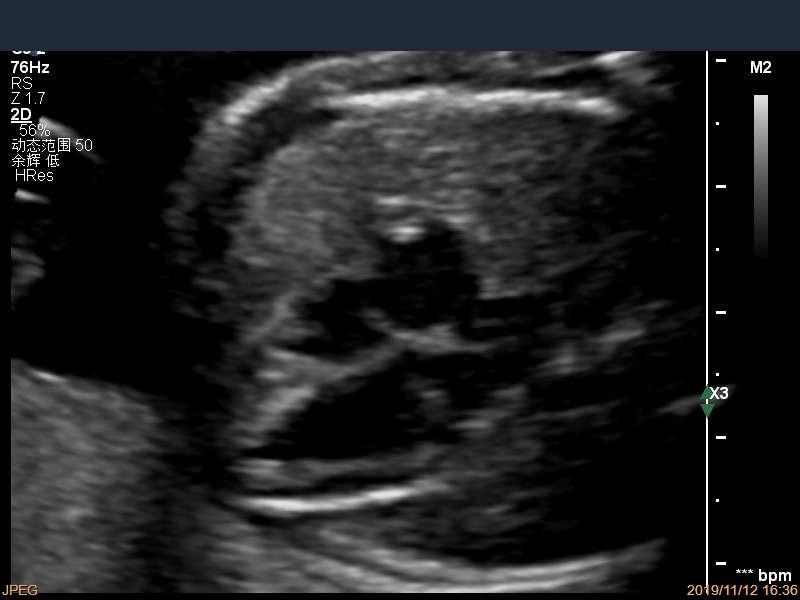

Supplement: S1 Dataset — (ZIP) [file pone.0305250.s001.zip › FE-SD-1/images/train_res/1095_fc.jpg]

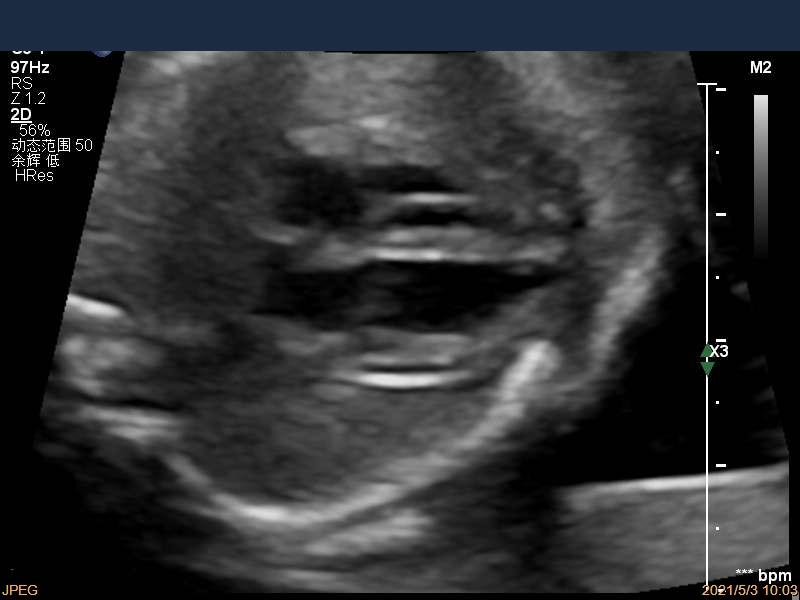

Supplement: S1 Dataset — (ZIP) [file pone.0305250.s001.zip › FE-SD-1/images/train_res/1097_fc.jpg]

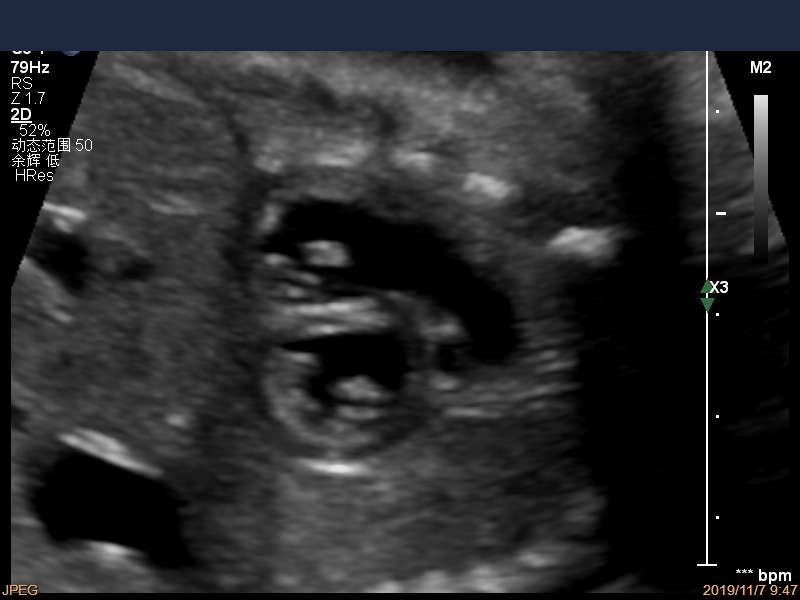

Supplement: S1 Dataset — (ZIP) [file pone.0305250.s001.zip › FE-SD-1/images/train_res/109_ro.jpg]

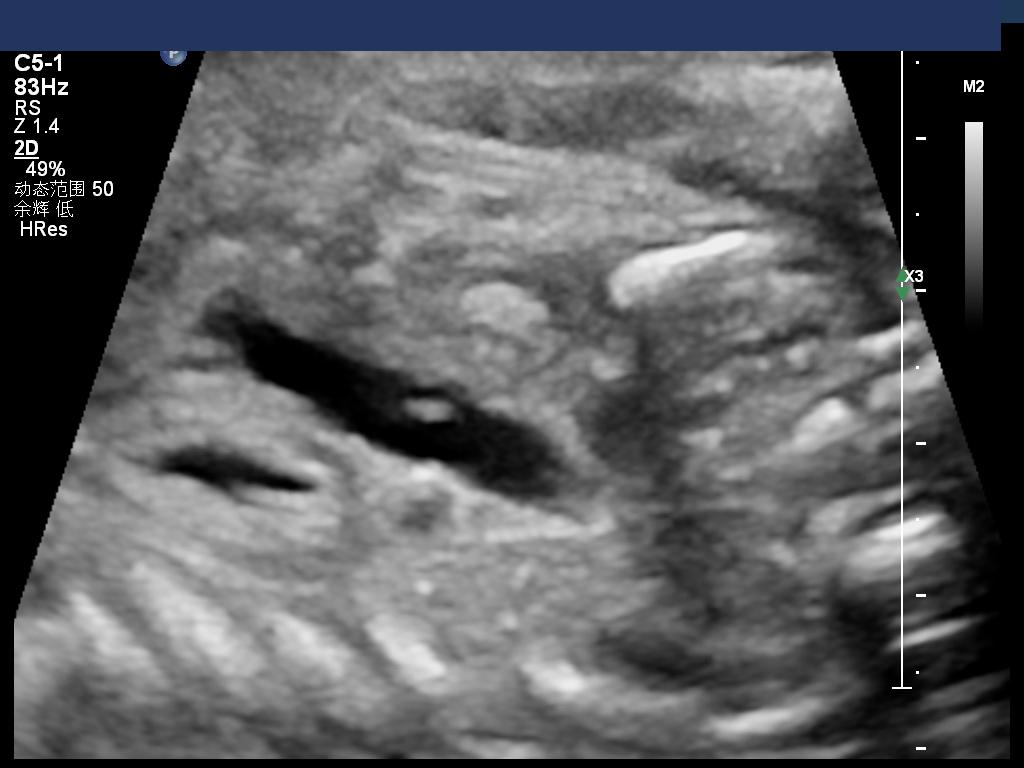

Supplement: S1 Dataset — (ZIP) [file pone.0305250.s001.zip › FE-SD-1/images/train_res/10_ro.jpg]

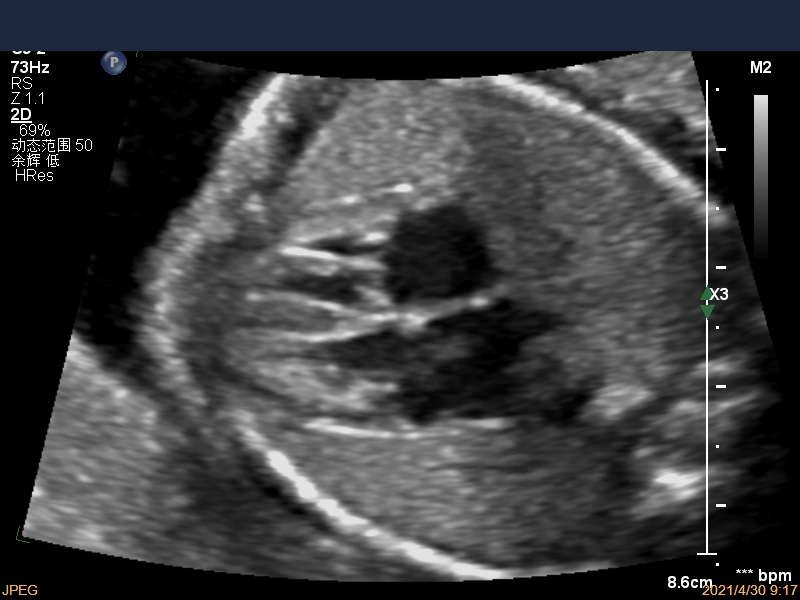

Supplement: S1 Dataset — (ZIP) [file pone.0305250.s001.zip › FE-SD-1/images/train_res/1100_fc.jpg]

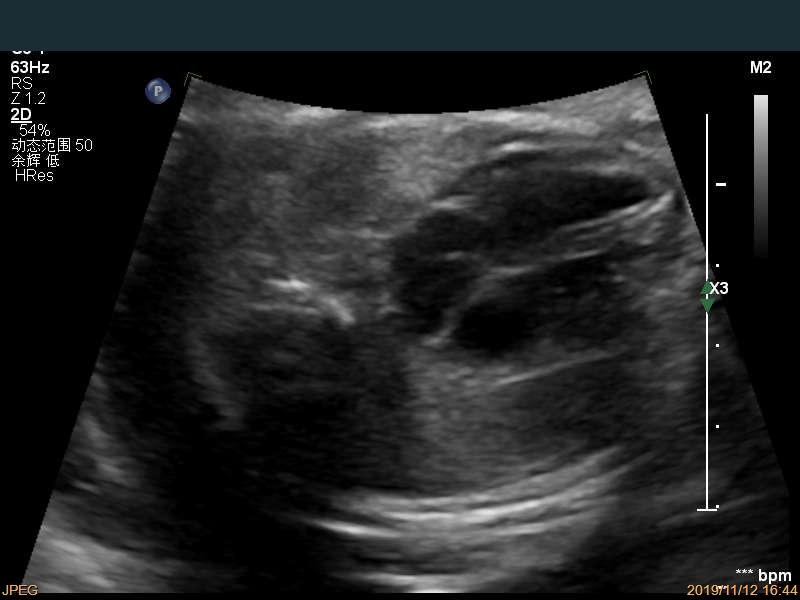

Supplement: S1 Dataset — (ZIP) [file pone.0305250.s001.zip › FE-SD-1/images/train_res/1102_fc.jpg]

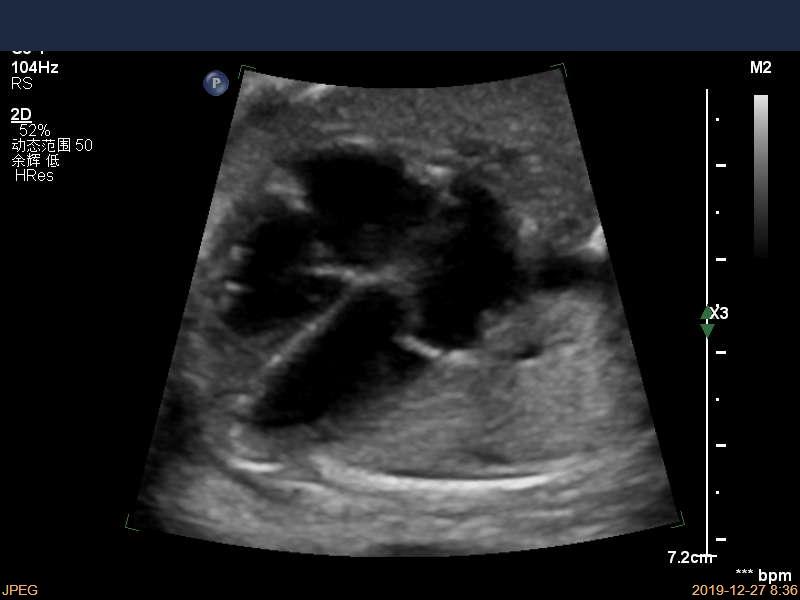

Supplement: S1 Dataset — (ZIP) [file pone.0305250.s001.zip › FE-SD-1/images/train_res/1107_fc.jpg]

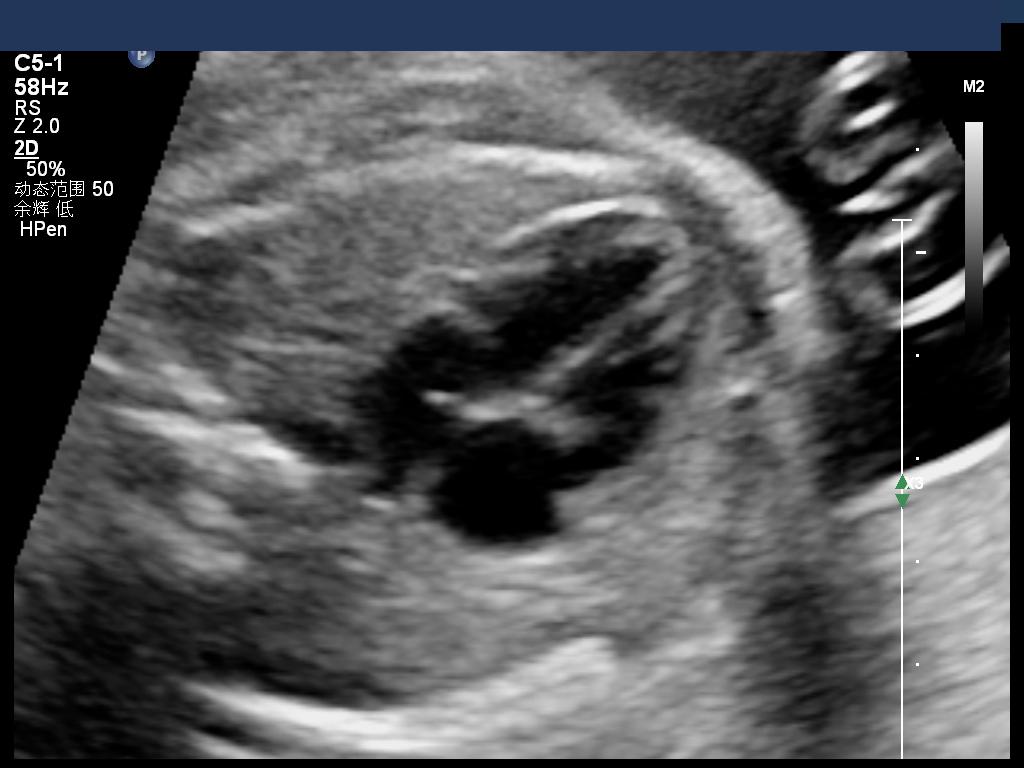

Supplement: S1 Dataset — (ZIP) [file pone.0305250.s001.zip › FE-SD-1/images/train_res/1108_fc.jpg]

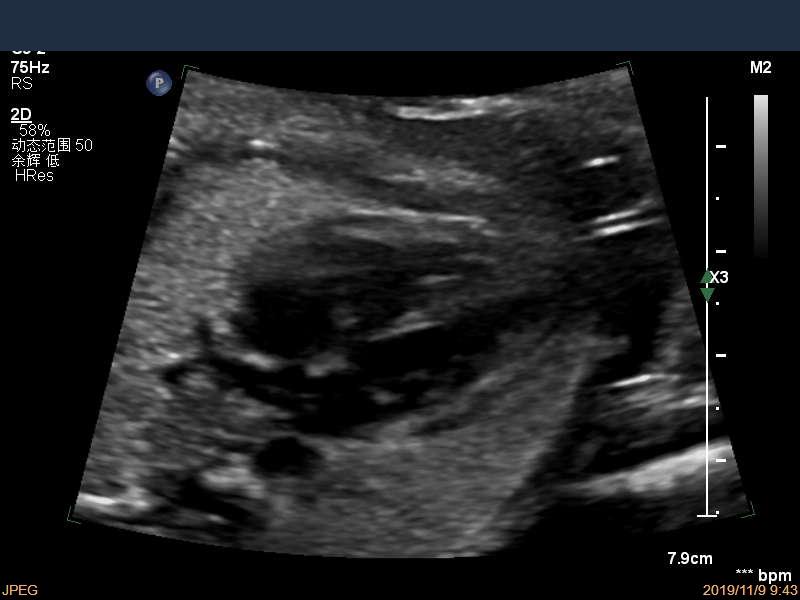

Supplement: S1 Dataset — (ZIP) [file pone.0305250.s001.zip › FE-SD-1/images/train_res/1110_fc.jpg]

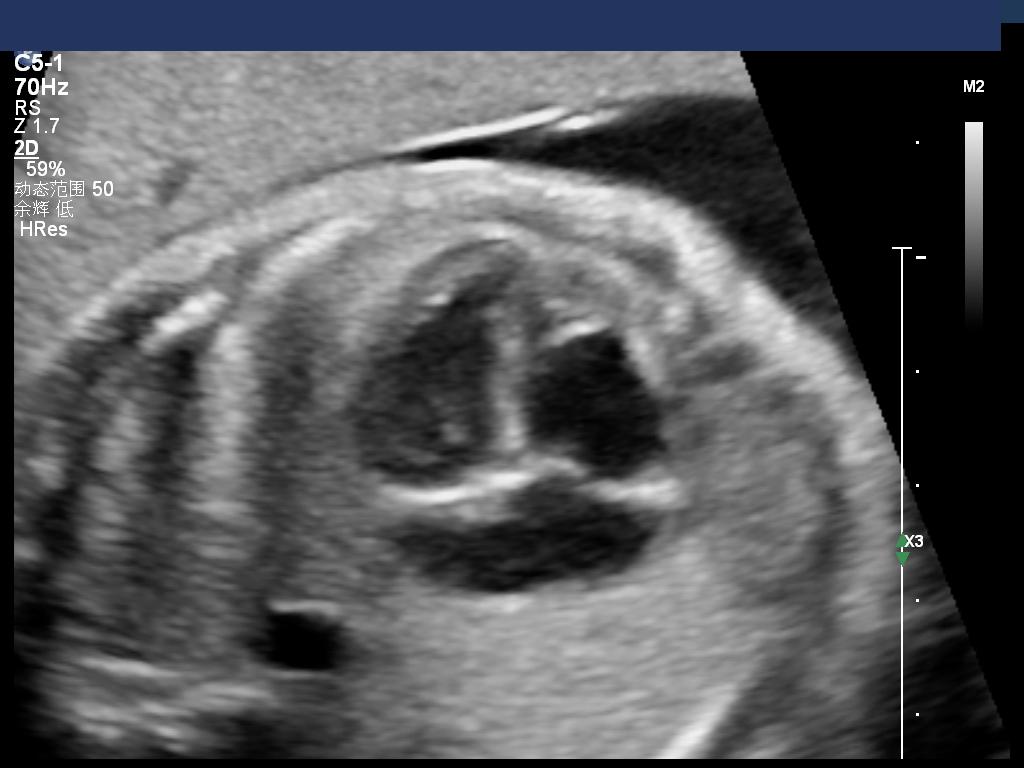

Supplement: S1 Dataset — (ZIP) [file pone.0305250.s001.zip › FE-SD-1/images/train_res/1112_fc.jpg]

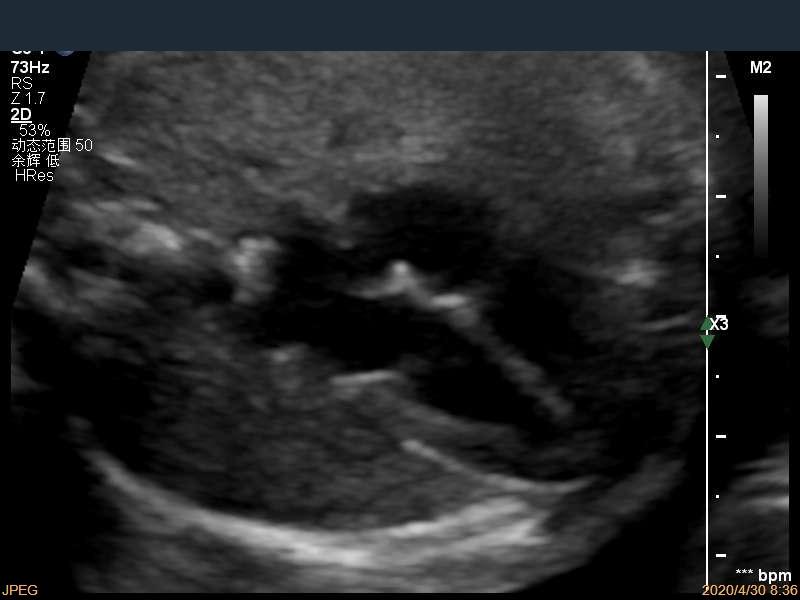

Supplement: S1 Dataset — (ZIP) [file pone.0305250.s001.zip › FE-SD-1/images/train_res/1115_fc.jpg]

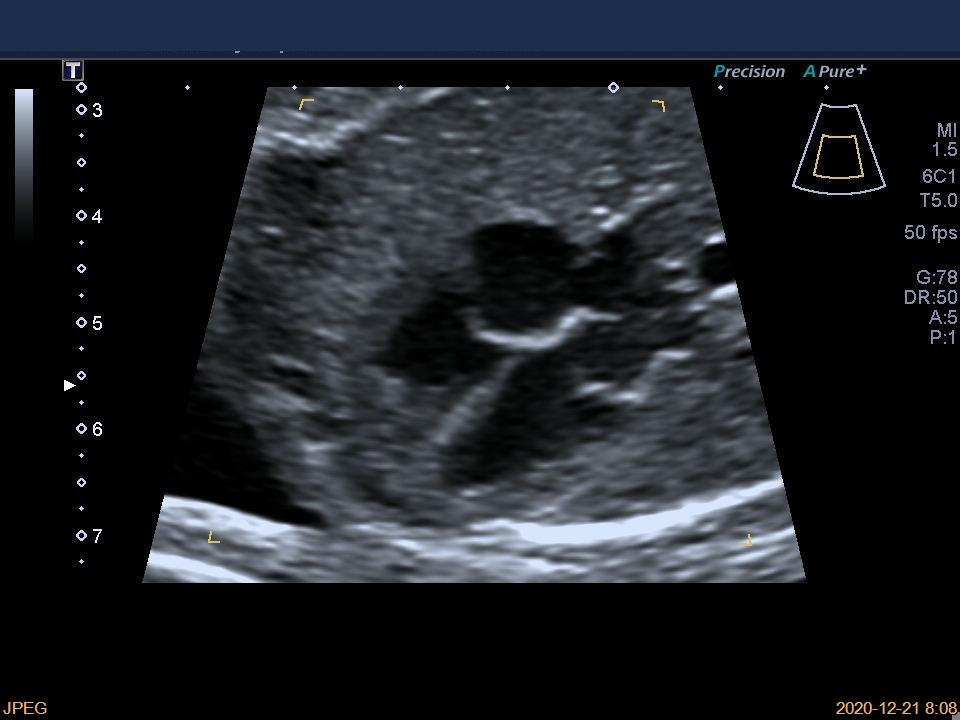

Supplement: S1 Dataset — (ZIP) [file pone.0305250.s001.zip › FE-SD-1/images/train_res/1117_fc.jpg]

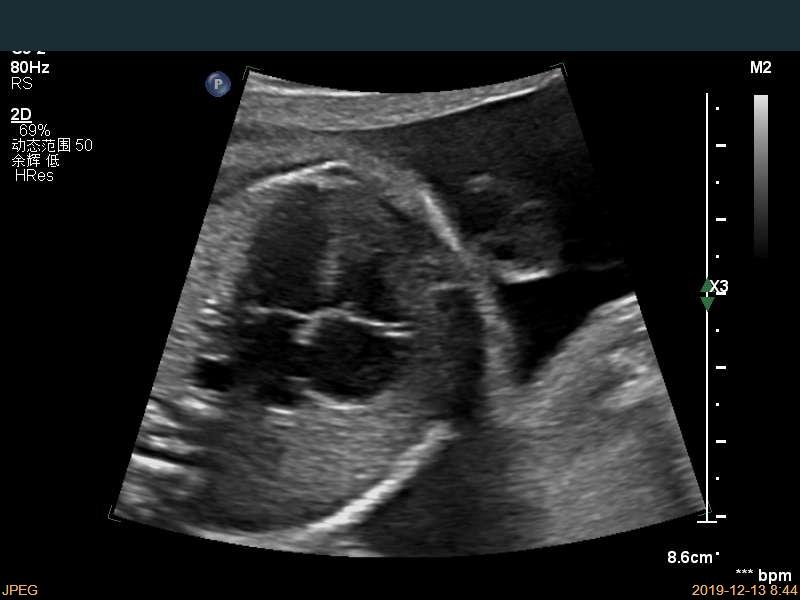

Supplement: S1 Dataset — (ZIP) [file pone.0305250.s001.zip › FE-SD-1/images/train_res/1118_fc.jpg]

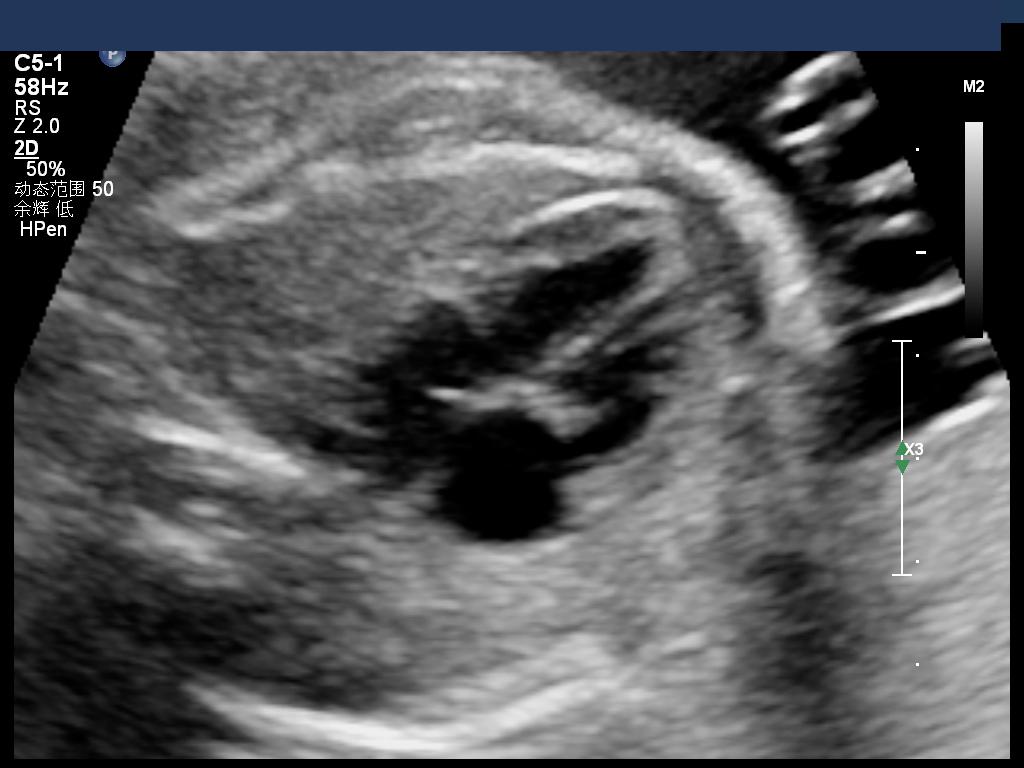

Supplement: S1 Dataset — (ZIP) [file pone.0305250.s001.zip › FE-SD-1/images/train_res/1119_fc.jpg]

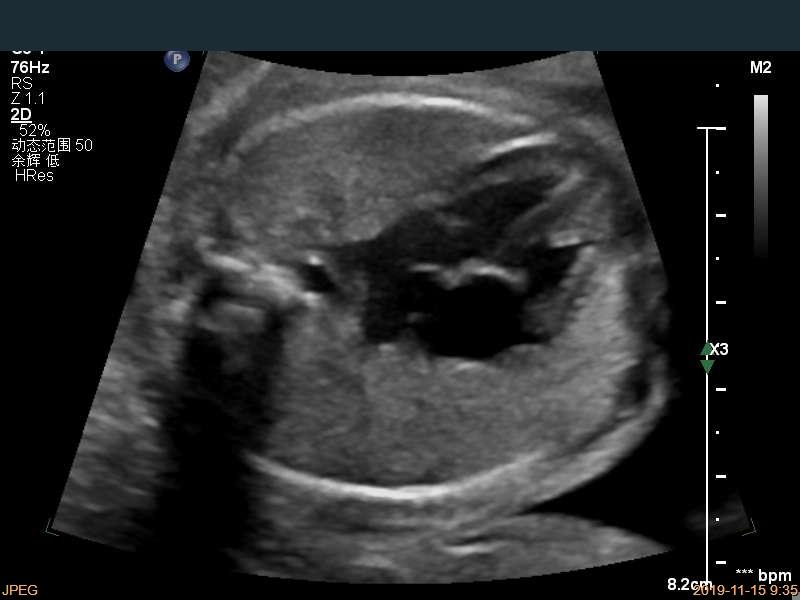

Supplement: S1 Dataset — (ZIP) [file pone.0305250.s001.zip › FE-SD-1/images/train_res/1121_fc.jpg]

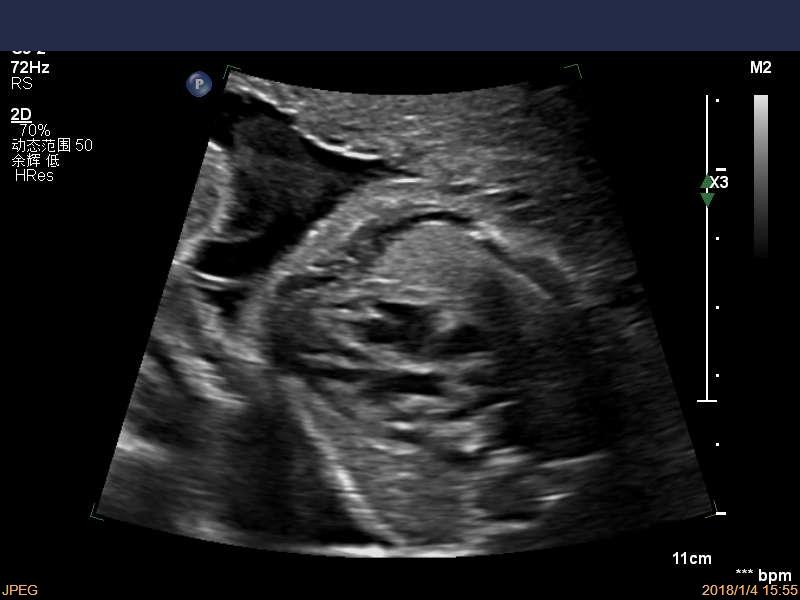

Supplement: S1 Dataset — (ZIP) [file pone.0305250.s001.zip › FE-SD-1/images/train_res/1122_fc.jpg]

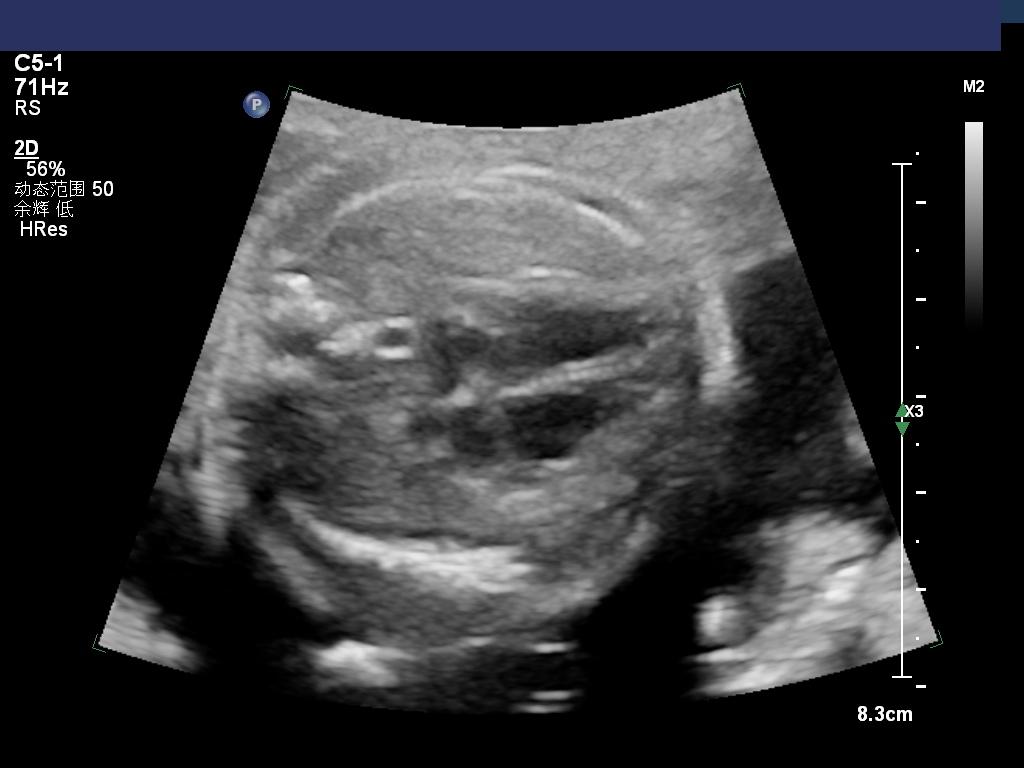

Supplement: S1 Dataset — (ZIP) [file pone.0305250.s001.zip › FE-SD-1/images/train_res/1124_fc.jpg]

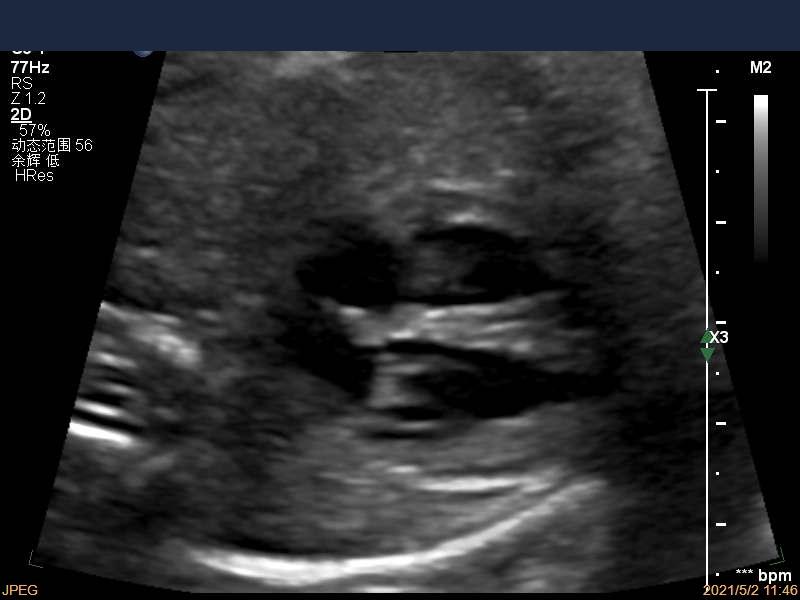

Supplement: S1 Dataset — (ZIP) [file pone.0305250.s001.zip › FE-SD-1/images/train_res/1125_fc.jpg]

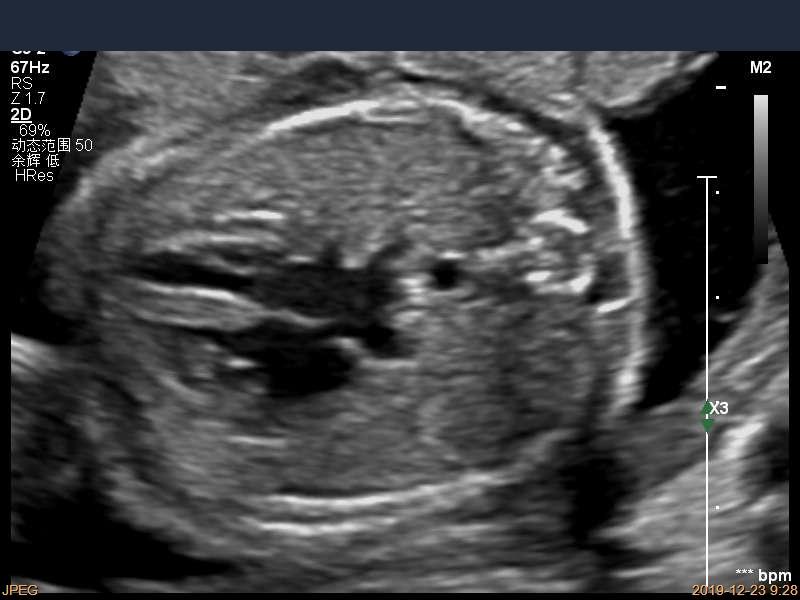

Supplement: S1 Dataset — (ZIP) [file pone.0305250.s001.zip › FE-SD-1/images/train_res/1126_fc.jpg]

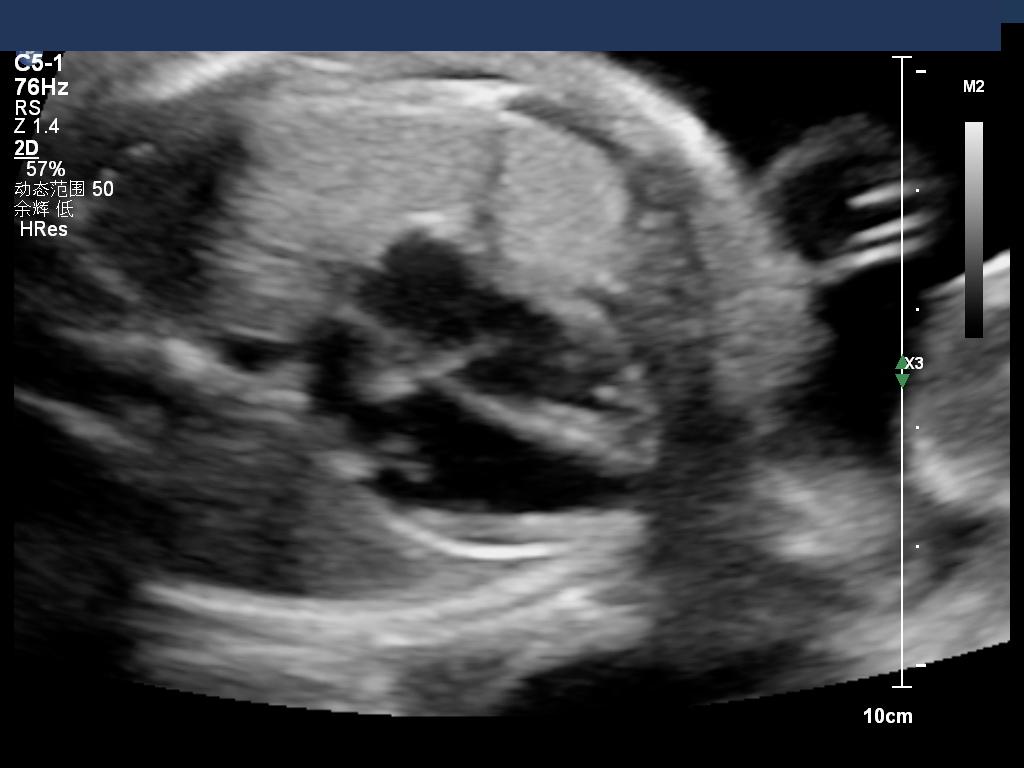

Supplement: S1 Dataset — (ZIP) [file pone.0305250.s001.zip › FE-SD-1/images/train_res/1130_fc.jpg]

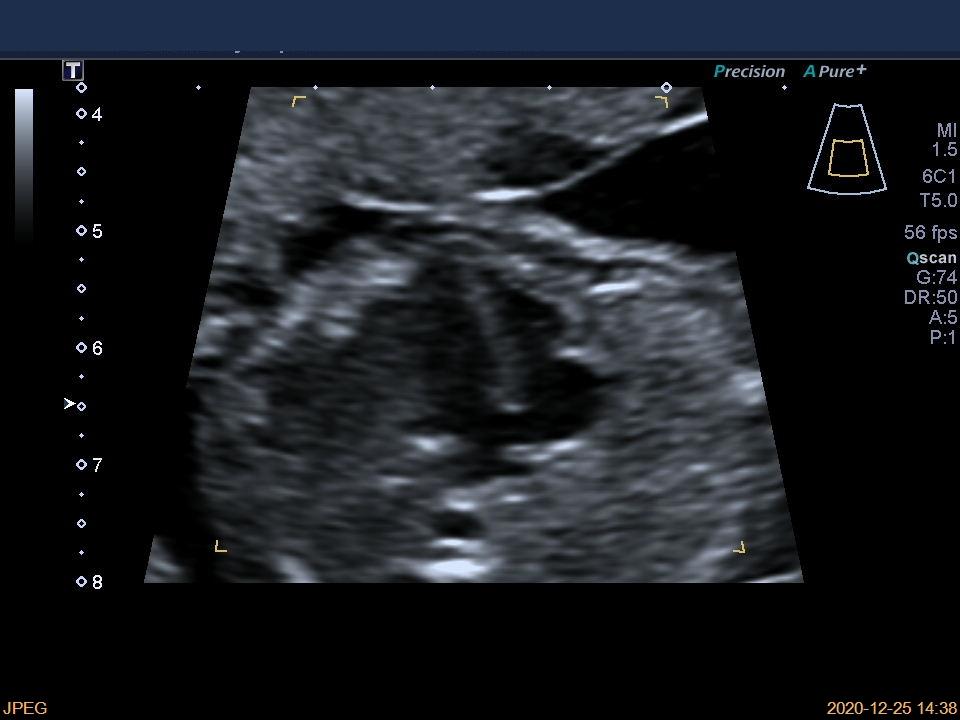

Supplement: S1 Dataset — (ZIP) [file pone.0305250.s001.zip › FE-SD-1/images/train_res/1135_fc.jpg]

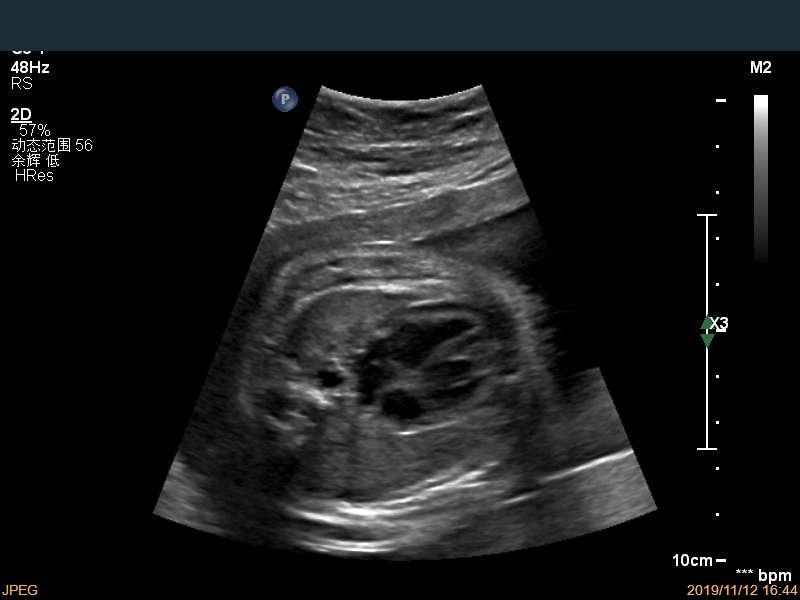

Supplement: S1 Dataset — (ZIP) [file pone.0305250.s001.zip › FE-SD-1/images/train_res/1136_fc.jpg]

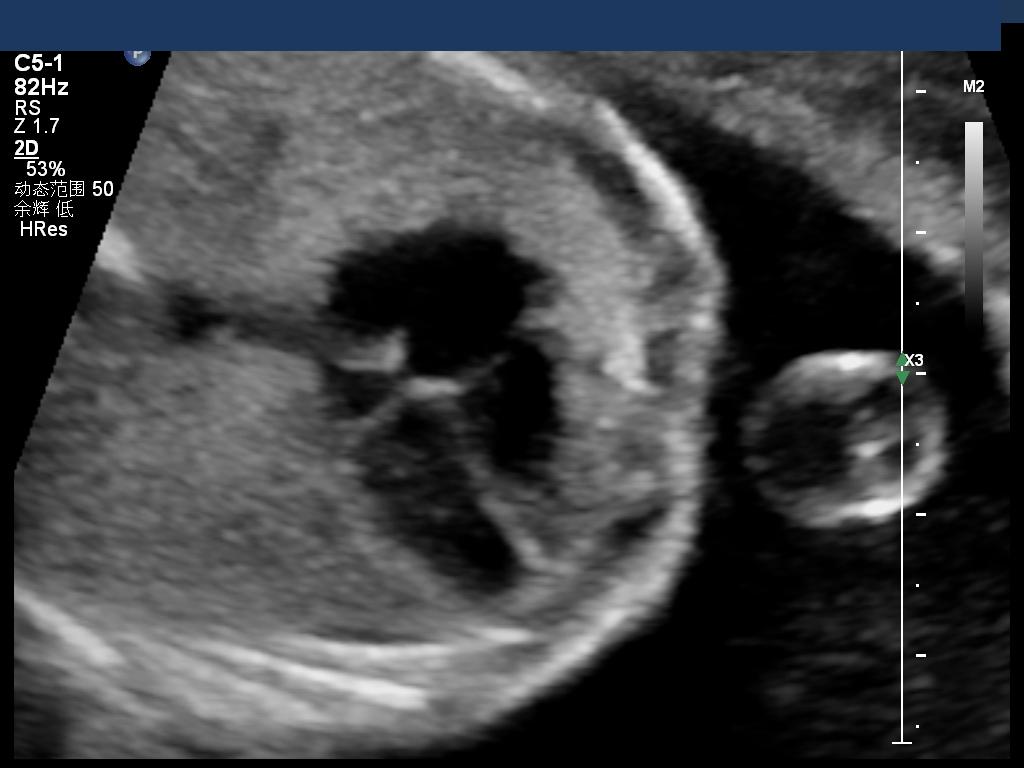

Supplement: S1 Dataset — (ZIP) [file pone.0305250.s001.zip › FE-SD-1/images/train_res/1137_fc.jpg]

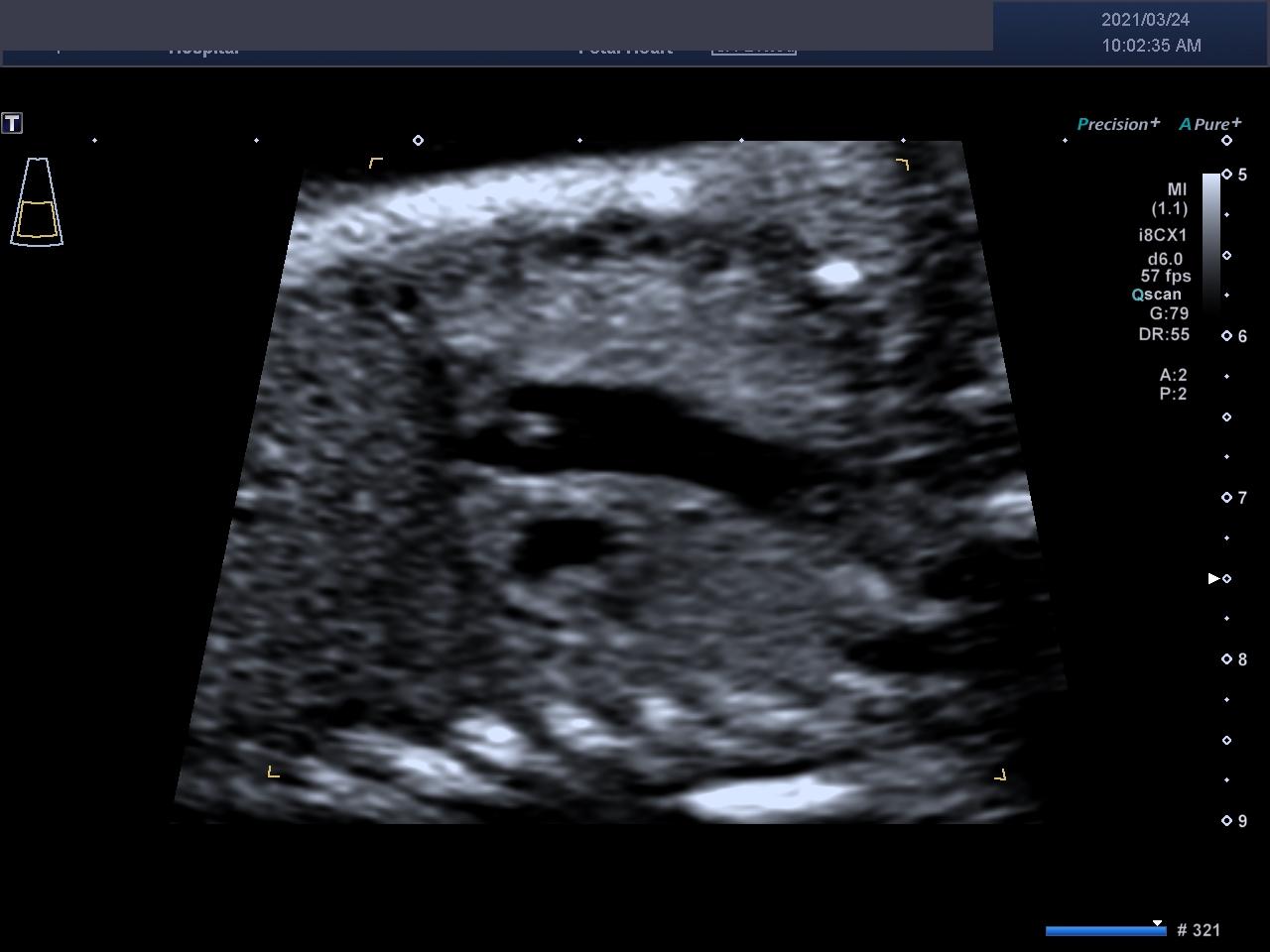

Supplement: S1 Dataset — (ZIP) [file pone.0305250.s001.zip › FE-SD-1/images/train_res/113_ro.jpg]

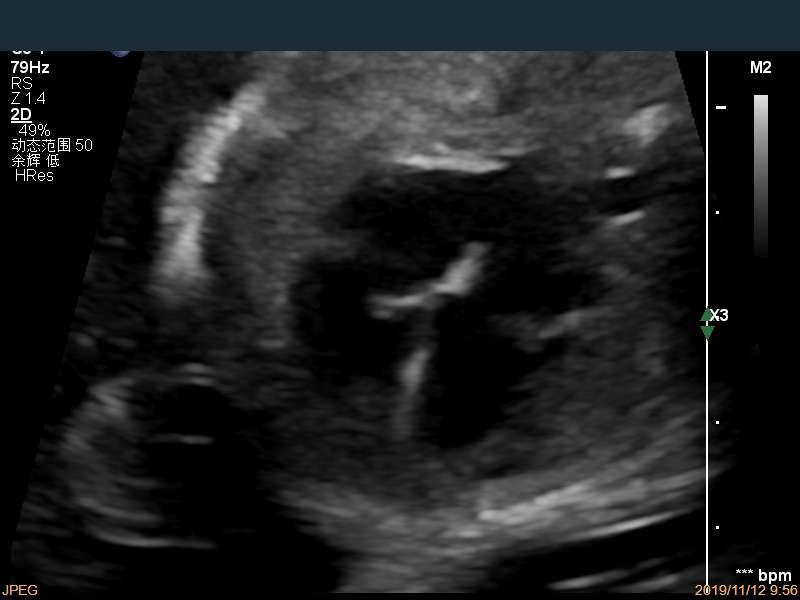

Supplement: S1 Dataset — (ZIP) [file pone.0305250.s001.zip › FE-SD-1/images/train_res/1140_fc.jpg]

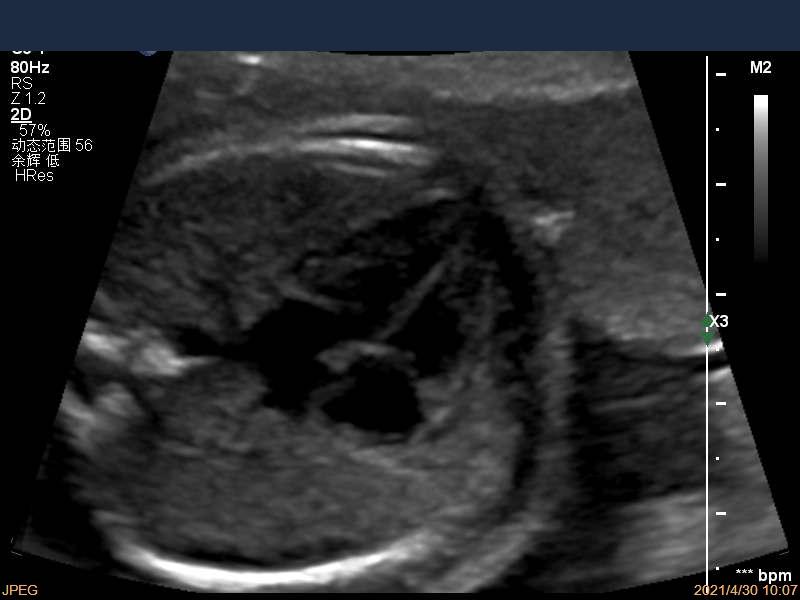

Supplement: S1 Dataset — (ZIP) [file pone.0305250.s001.zip › FE-SD-1/images/train_res/1141_fc.jpg]

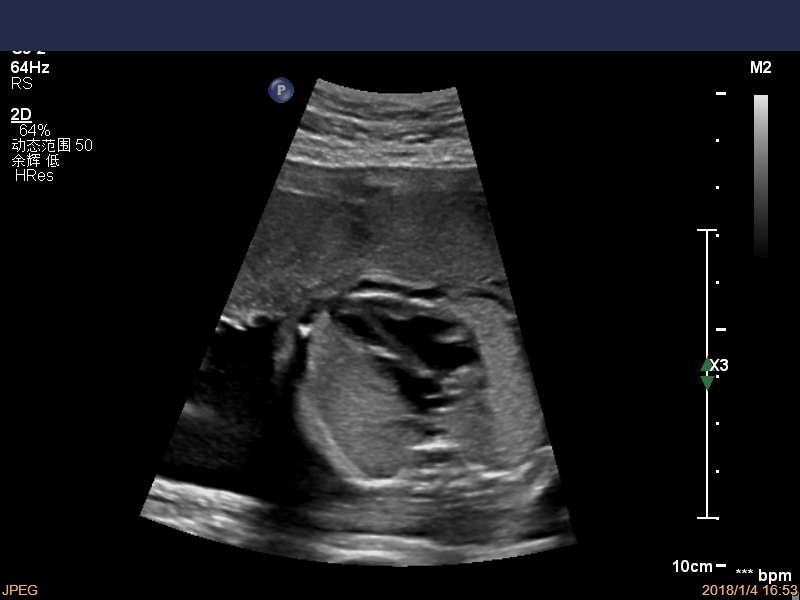

Supplement: S1 Dataset — (ZIP) [file pone.0305250.s001.zip › FE-SD-1/images/train_res/1142_fc.jpg]

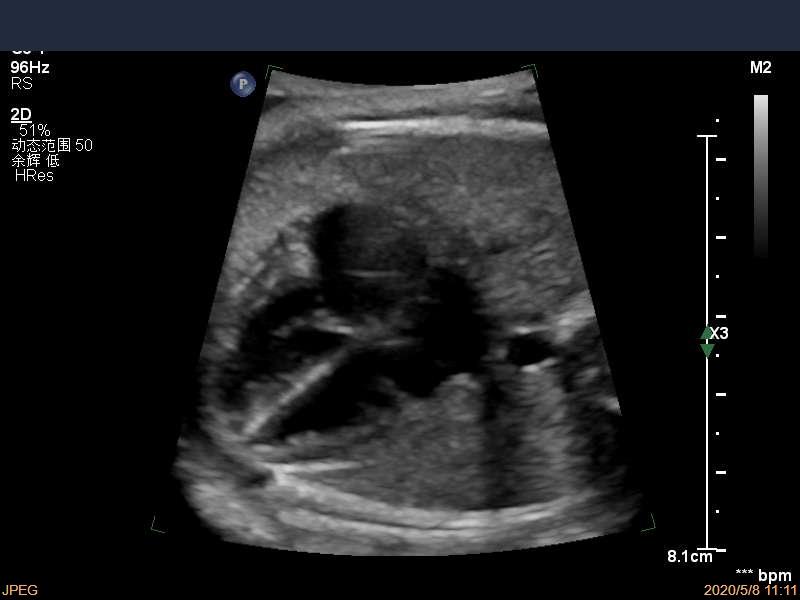

Supplement: S1 Dataset — (ZIP) [file pone.0305250.s001.zip › FE-SD-1/images/train_res/1147_fc.jpg]

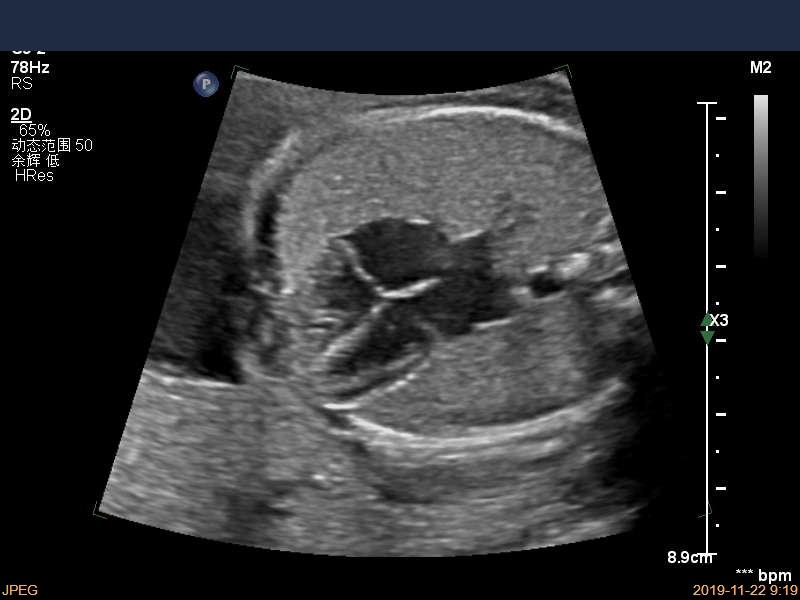

Supplement: S1 Dataset — (ZIP) [file pone.0305250.s001.zip › FE-SD-1/images/train_res/1148_fc.jpg]

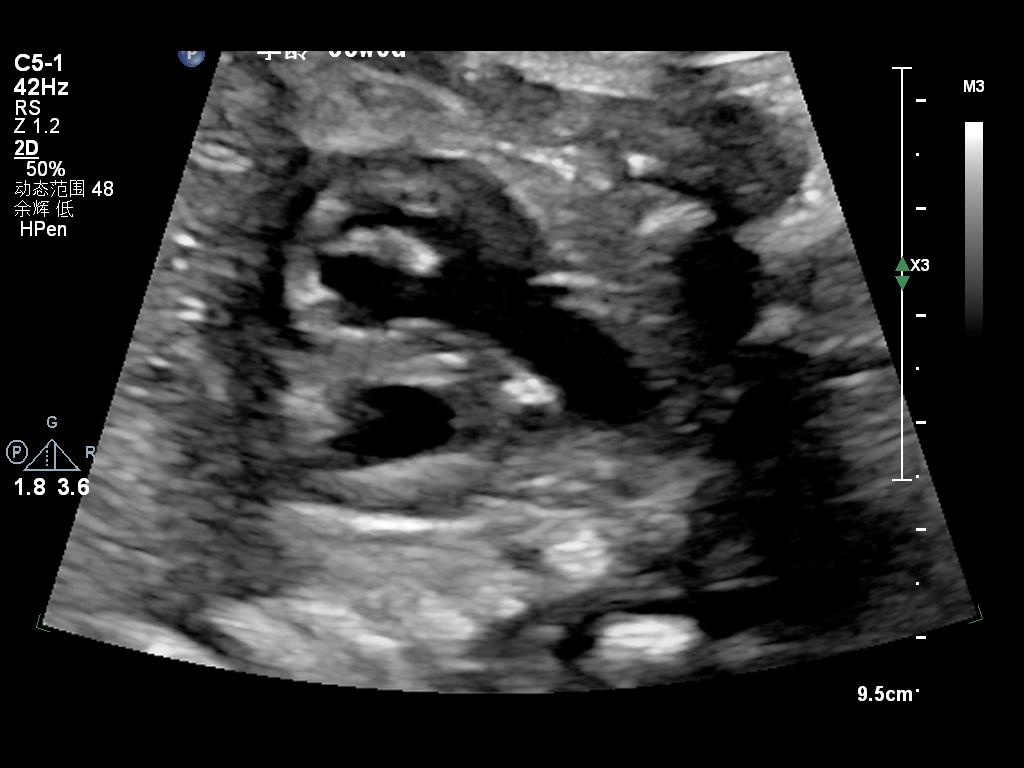

Supplement: S1 Dataset — (ZIP) [file pone.0305250.s001.zip › FE-SD-1/images/train_res/114_ro.jpg]

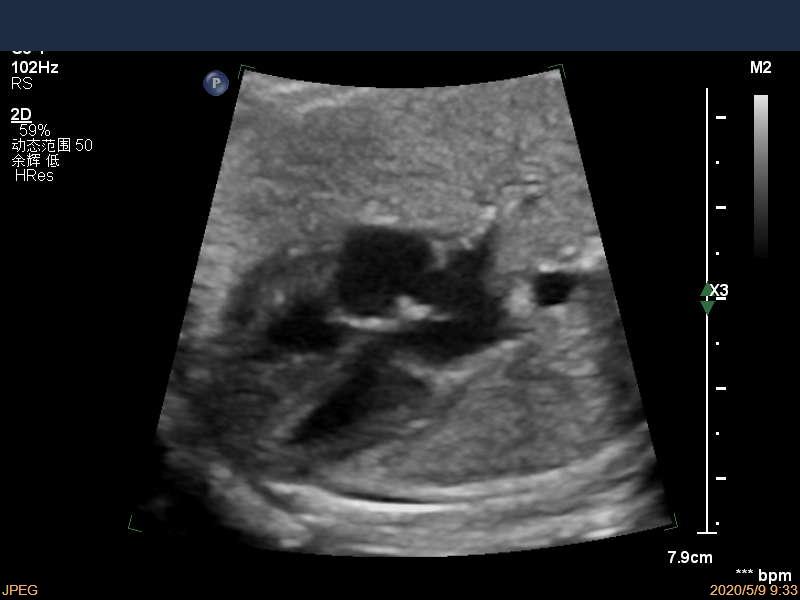

Supplement: S1 Dataset — (ZIP) [file pone.0305250.s001.zip › FE-SD-1/images/train_res/1150_fc.jpg]

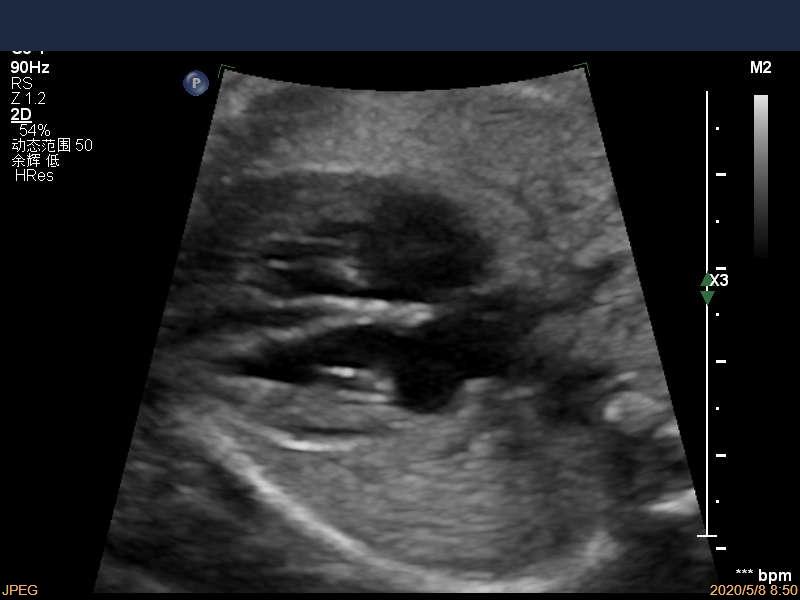

Supplement: S1 Dataset — (ZIP) [file pone.0305250.s001.zip › FE-SD-1/images/train_res/1151_fc.jpg]

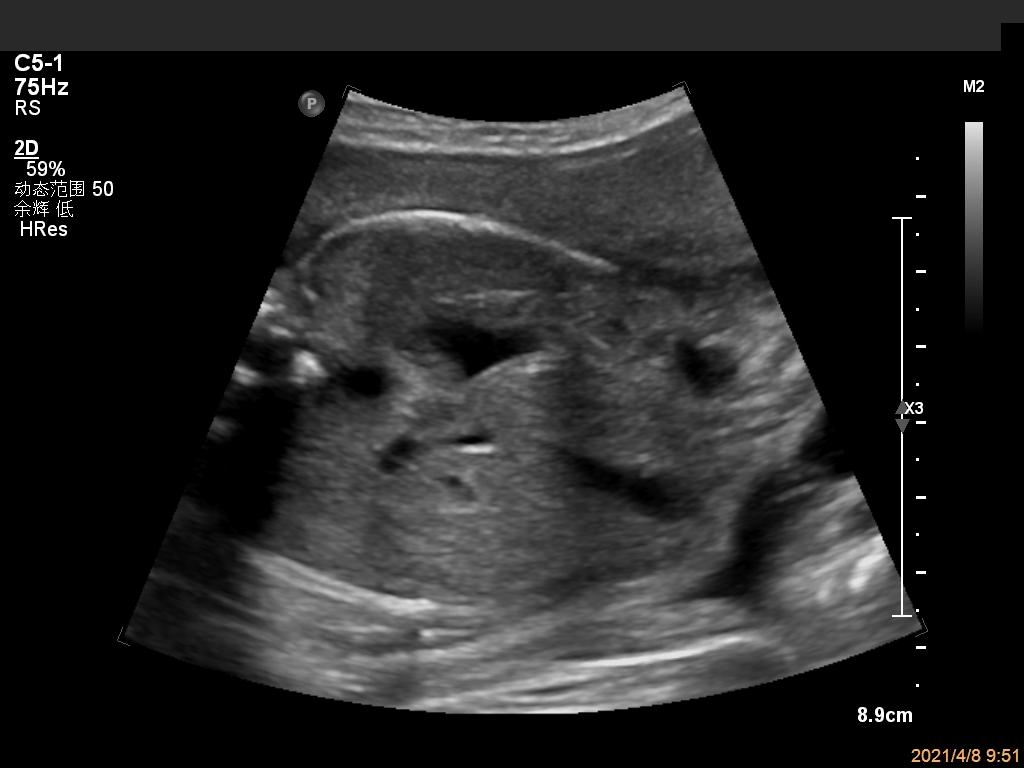

Supplement: S1 Dataset — (ZIP) [file pone.0305250.s001.zip › FE-SD-1/images/train_res/1152_ab.jpg]

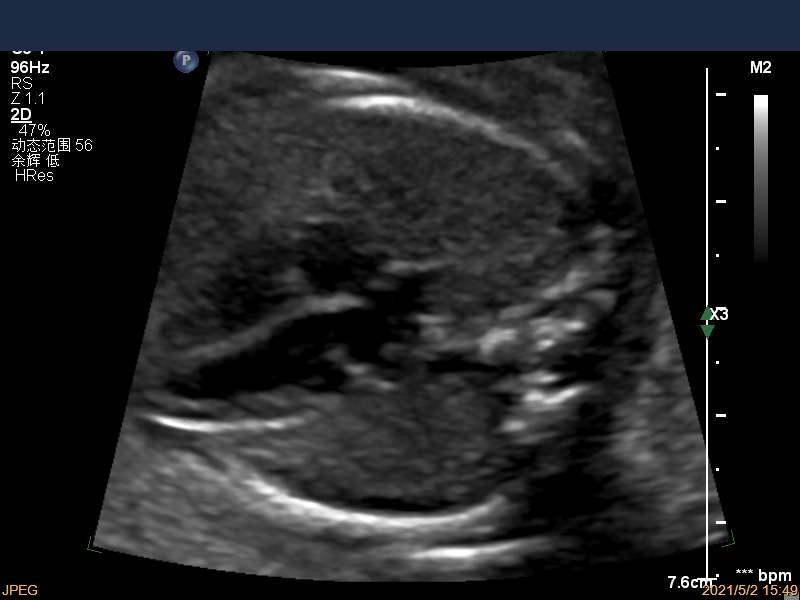

Supplement: S1 Dataset — (ZIP) [file pone.0305250.s001.zip › FE-SD-1/images/train_res/1152_fc.jpg]

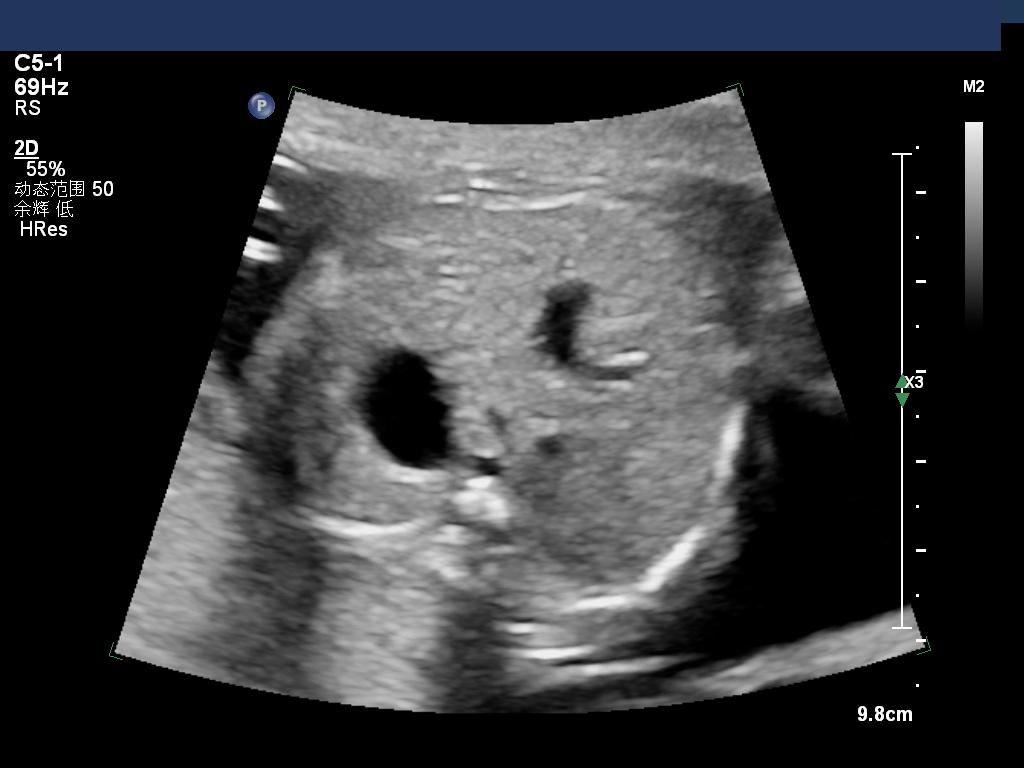

Supplement: S1 Dataset — (ZIP) [file pone.0305250.s001.zip › FE-SD-1/images/train_res/1153_ab.jpg]

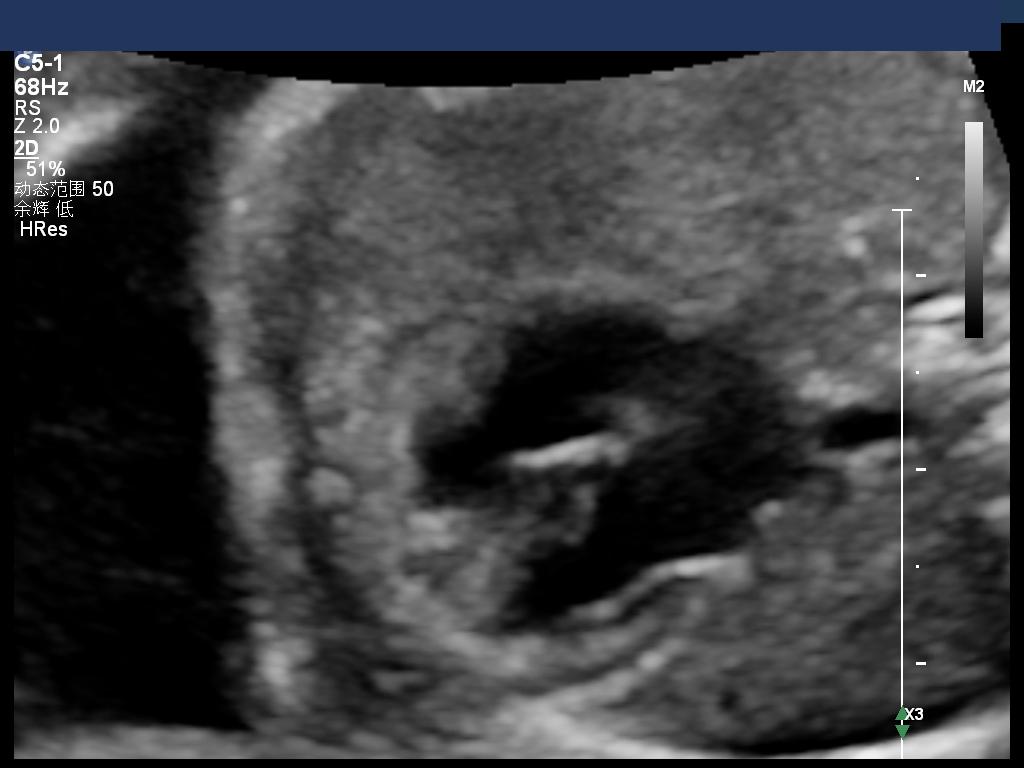

Supplement: S1 Dataset — (ZIP) [file pone.0305250.s001.zip › FE-SD-1/images/train_res/1153_fc.jpg]

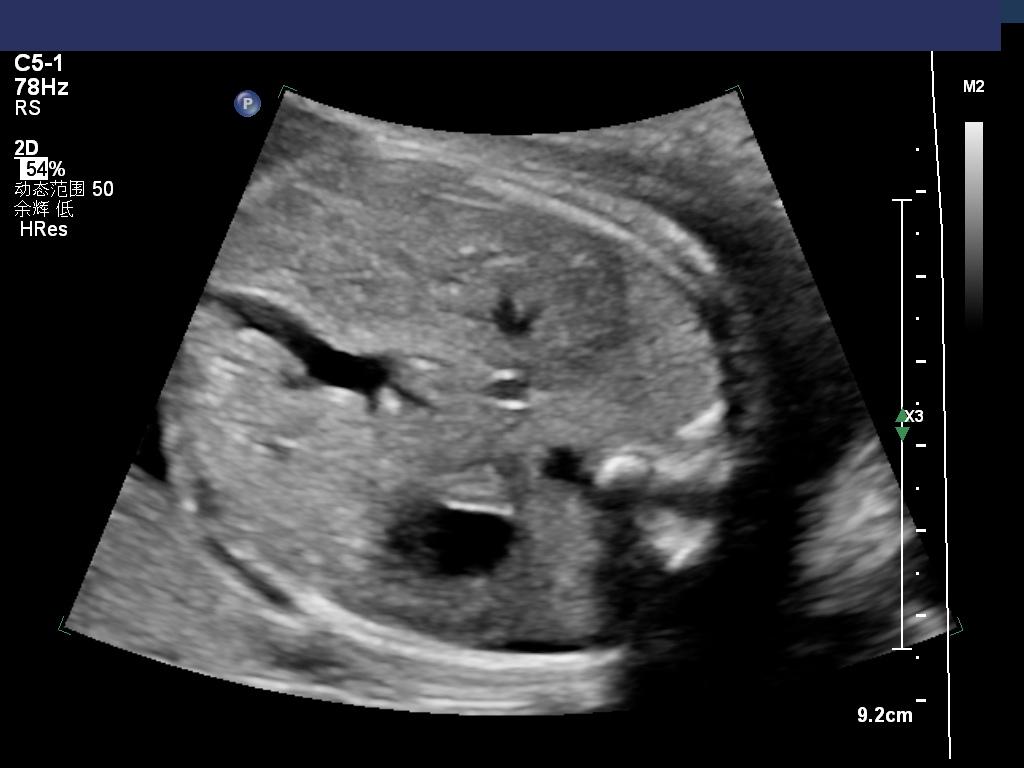

Supplement: S1 Dataset — (ZIP) [file pone.0305250.s001.zip › FE-SD-1/images/train_res/1154_ab.jpg]

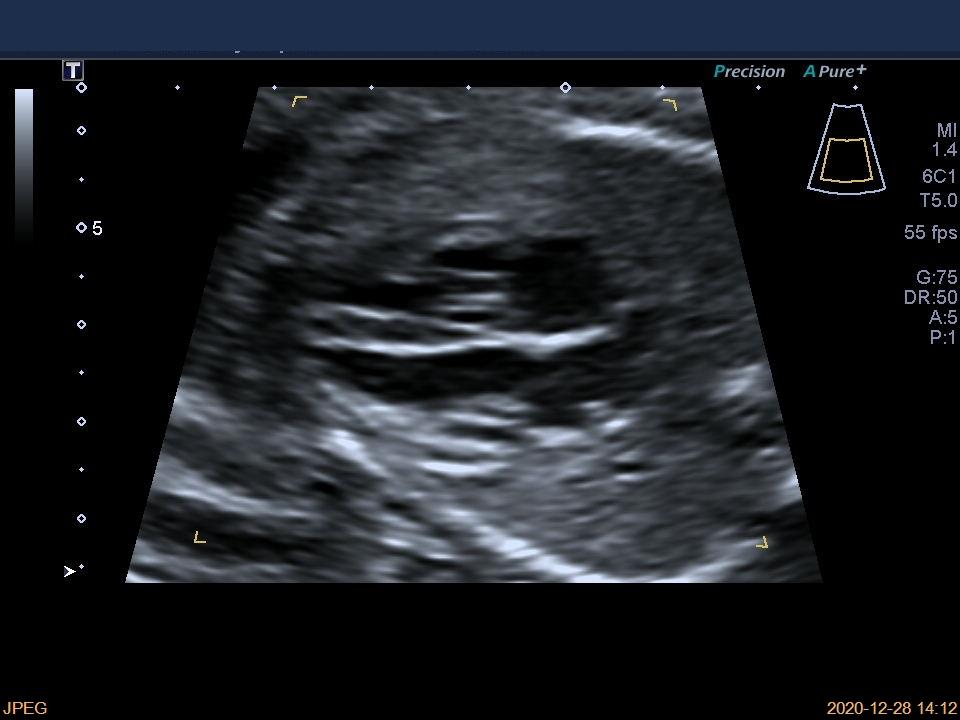

Supplement: S1 Dataset — (ZIP) [file pone.0305250.s001.zip › FE-SD-1/images/train_res/1154_fc.jpg]

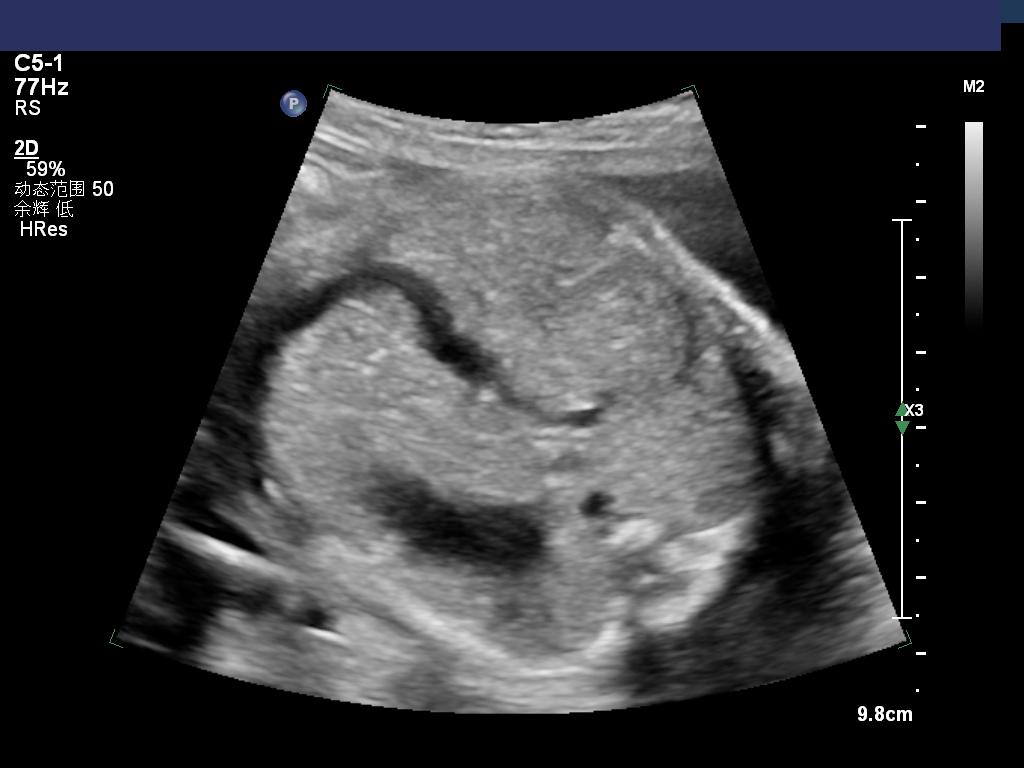

Supplement: S1 Dataset — (ZIP) [file pone.0305250.s001.zip › FE-SD-1/images/train_res/1155_ab.jpg]

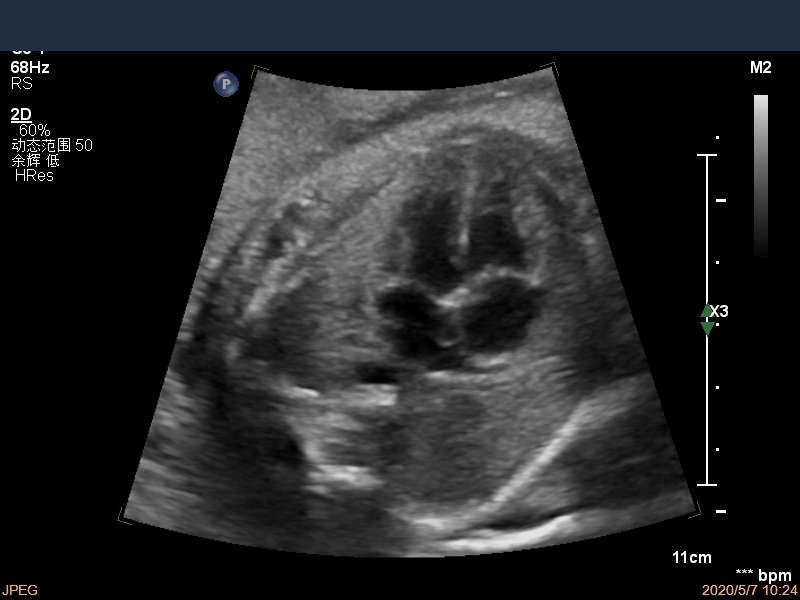

Supplement: S1 Dataset — (ZIP) [file pone.0305250.s001.zip › FE-SD-1/images/train_res/1155_fc.jpg]

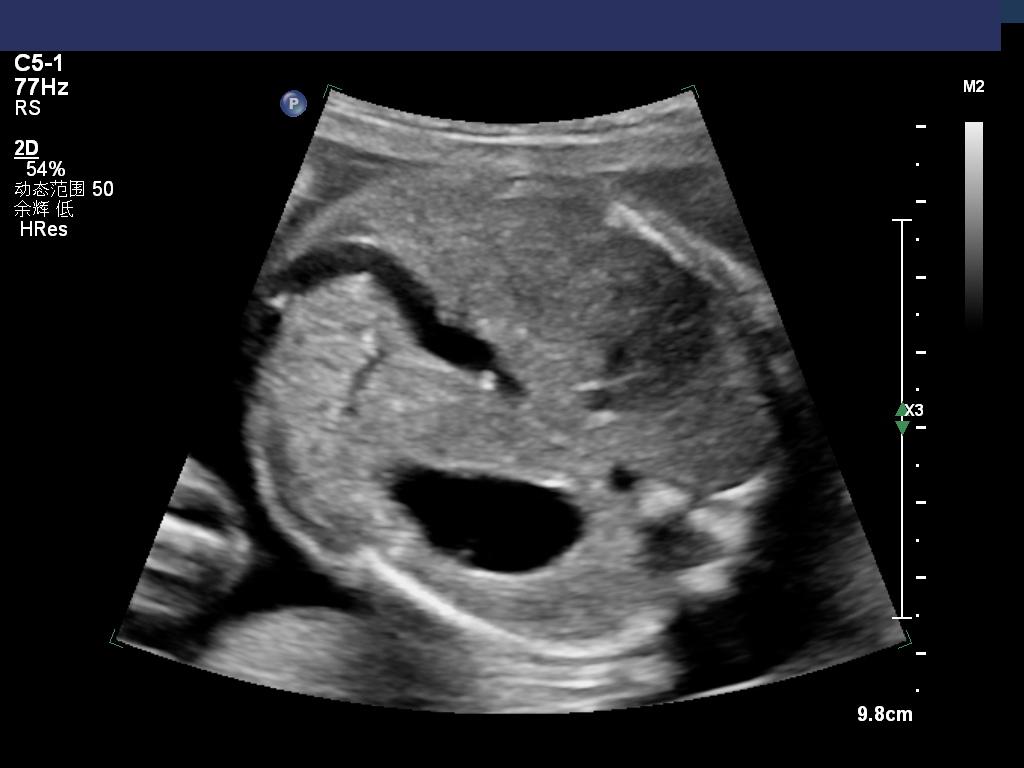

Supplement: S1 Dataset — (ZIP) [file pone.0305250.s001.zip › FE-SD-1/images/train_res/1156_ab.jpg]

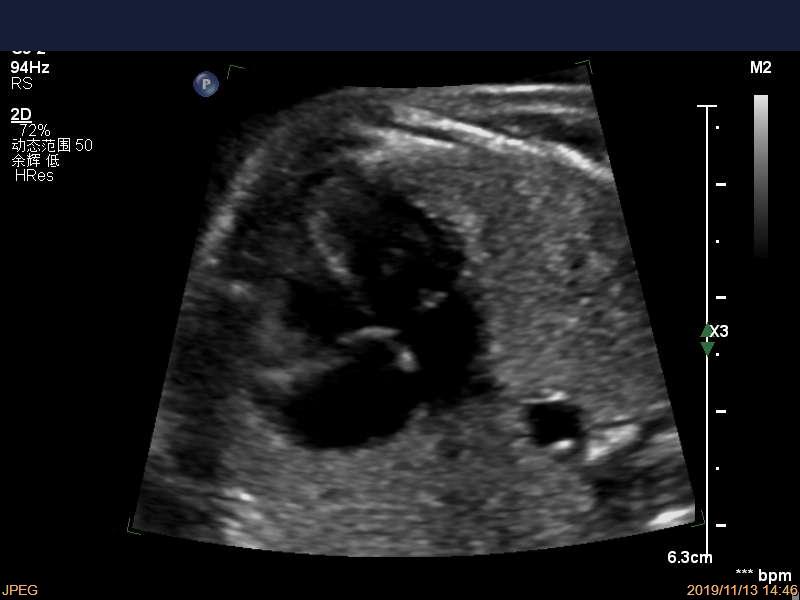

Supplement: S1 Dataset — (ZIP) [file pone.0305250.s001.zip › FE-SD-1/images/train_res/1156_fc.jpg]

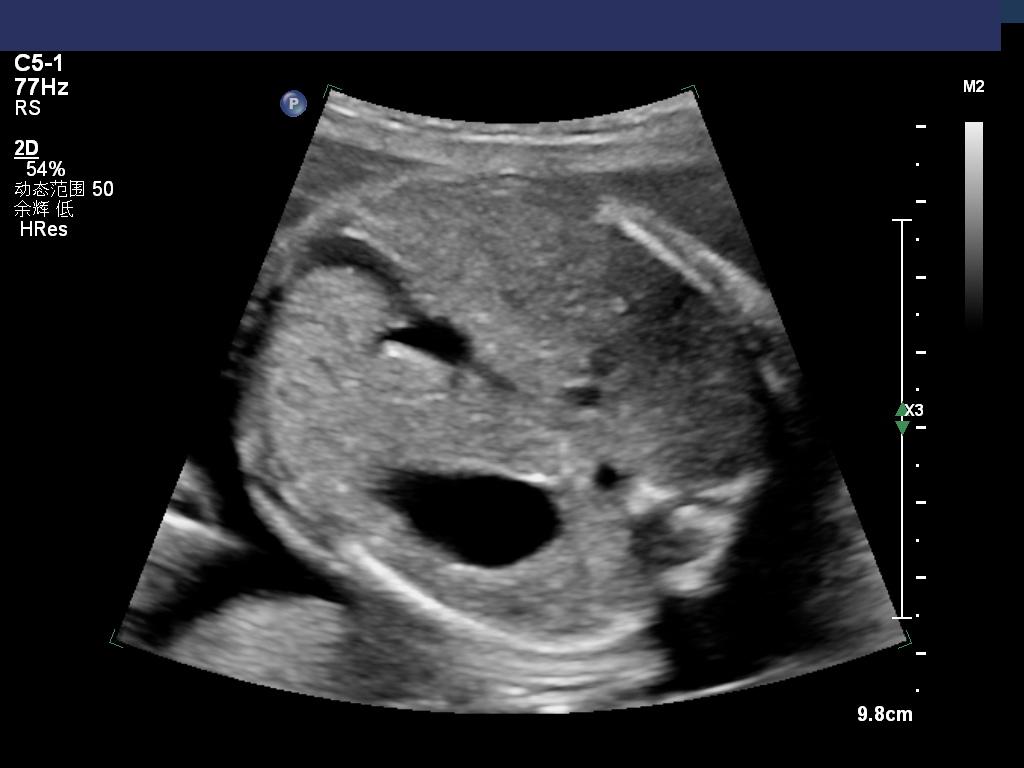

Supplement: S1 Dataset — (ZIP) [file pone.0305250.s001.zip › FE-SD-1/images/train_res/1157_ab.jpg]

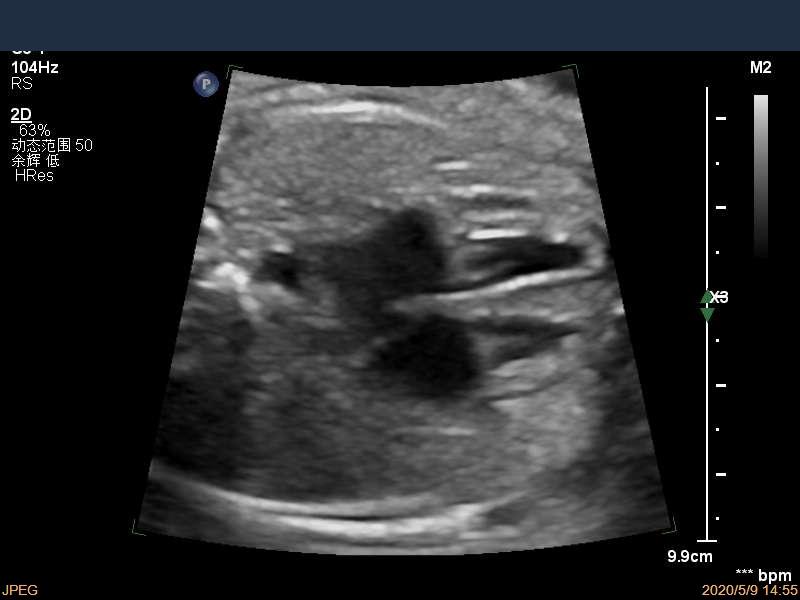

Supplement: S1 Dataset — (ZIP) [file pone.0305250.s001.zip › FE-SD-1/images/train_res/1157_fc.jpg]

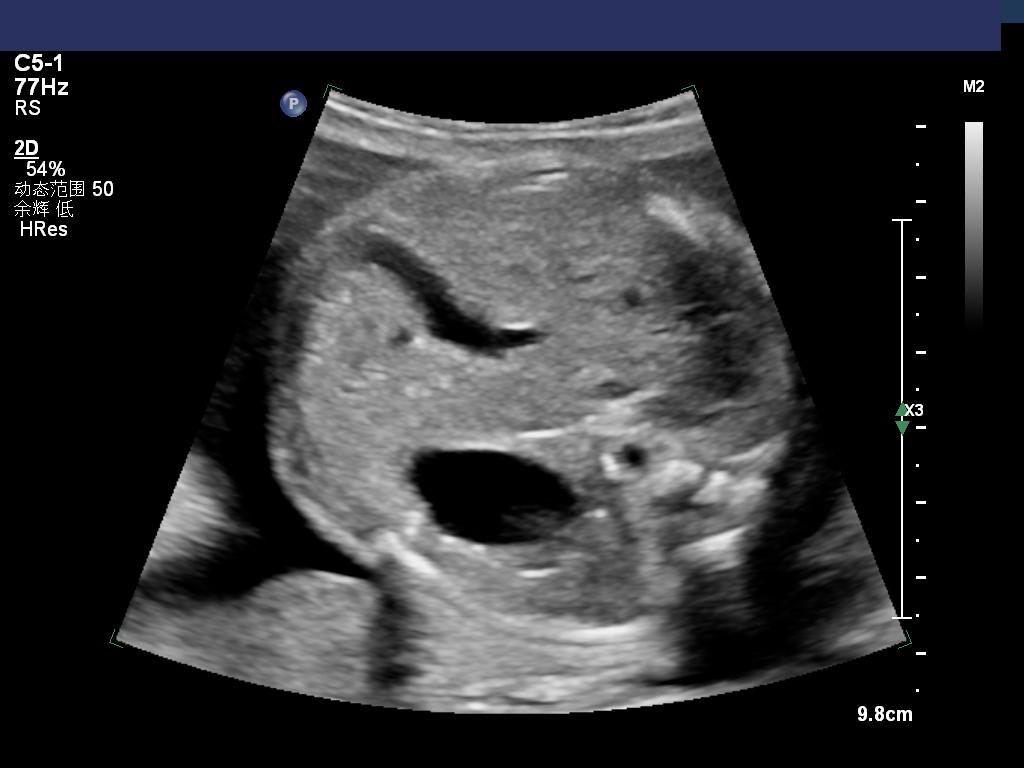

Supplement: S1 Dataset — (ZIP) [file pone.0305250.s001.zip › FE-SD-1/images/train_res/1158_ab.jpg]

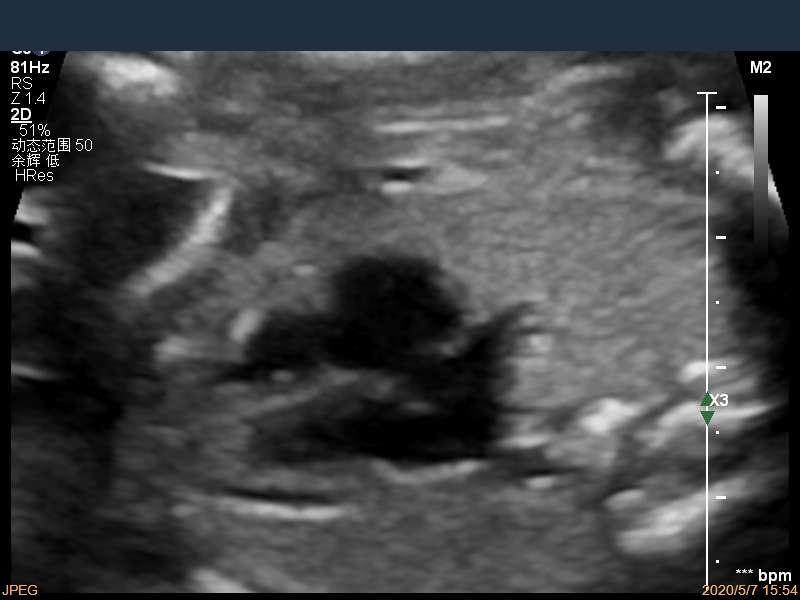

Supplement: S1 Dataset — (ZIP) [file pone.0305250.s001.zip › FE-SD-1/images/train_res/1158_fc.jpg]

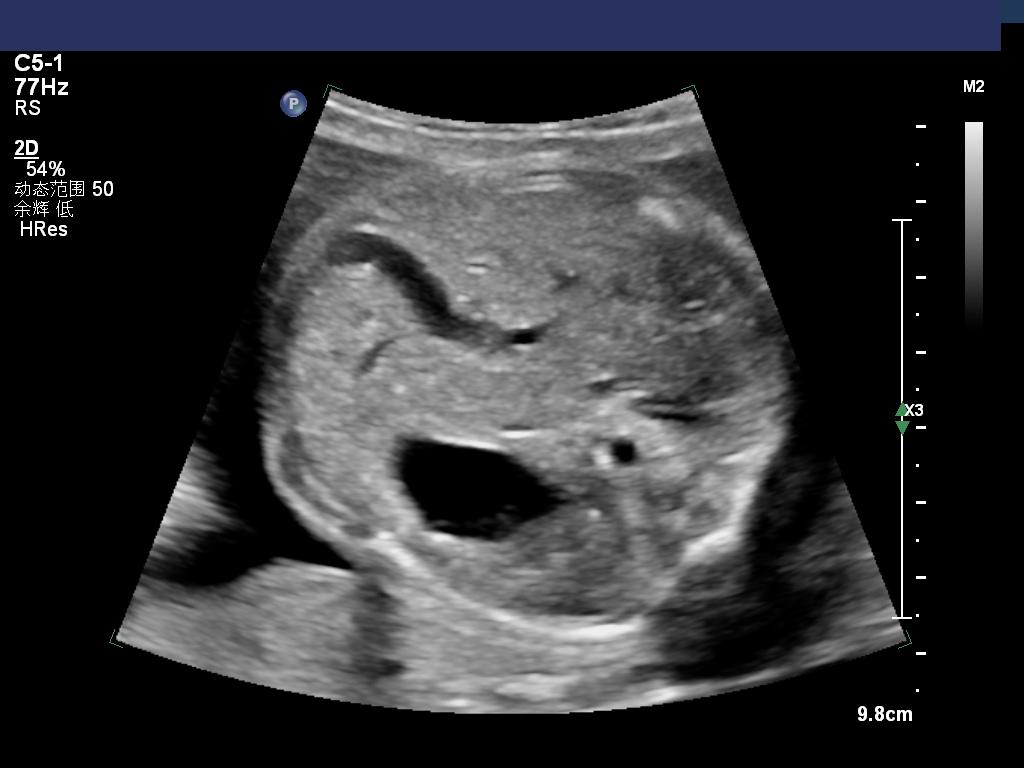

Supplement: S1 Dataset — (ZIP) [file pone.0305250.s001.zip › FE-SD-1/images/train_res/1159_ab.jpg]

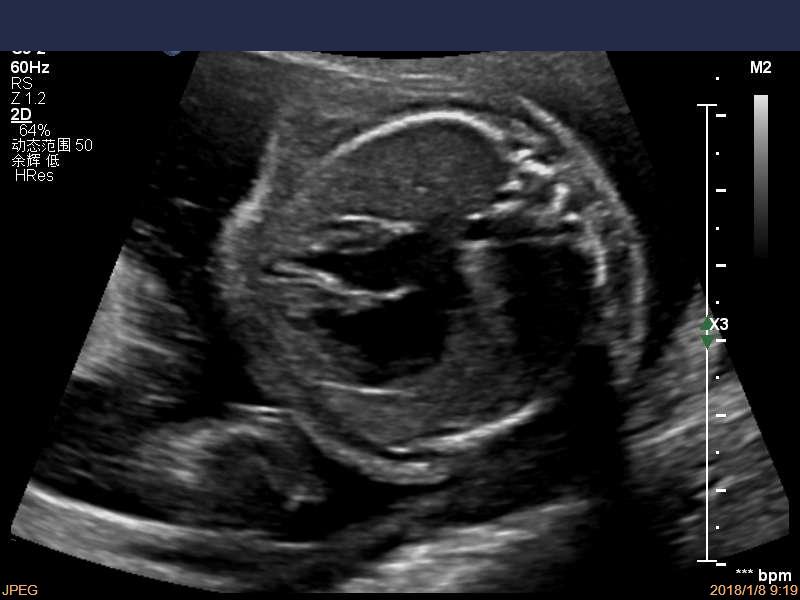

Supplement: S1 Dataset — (ZIP) [file pone.0305250.s001.zip › FE-SD-1/images/train_res/1159_fc.jpg]

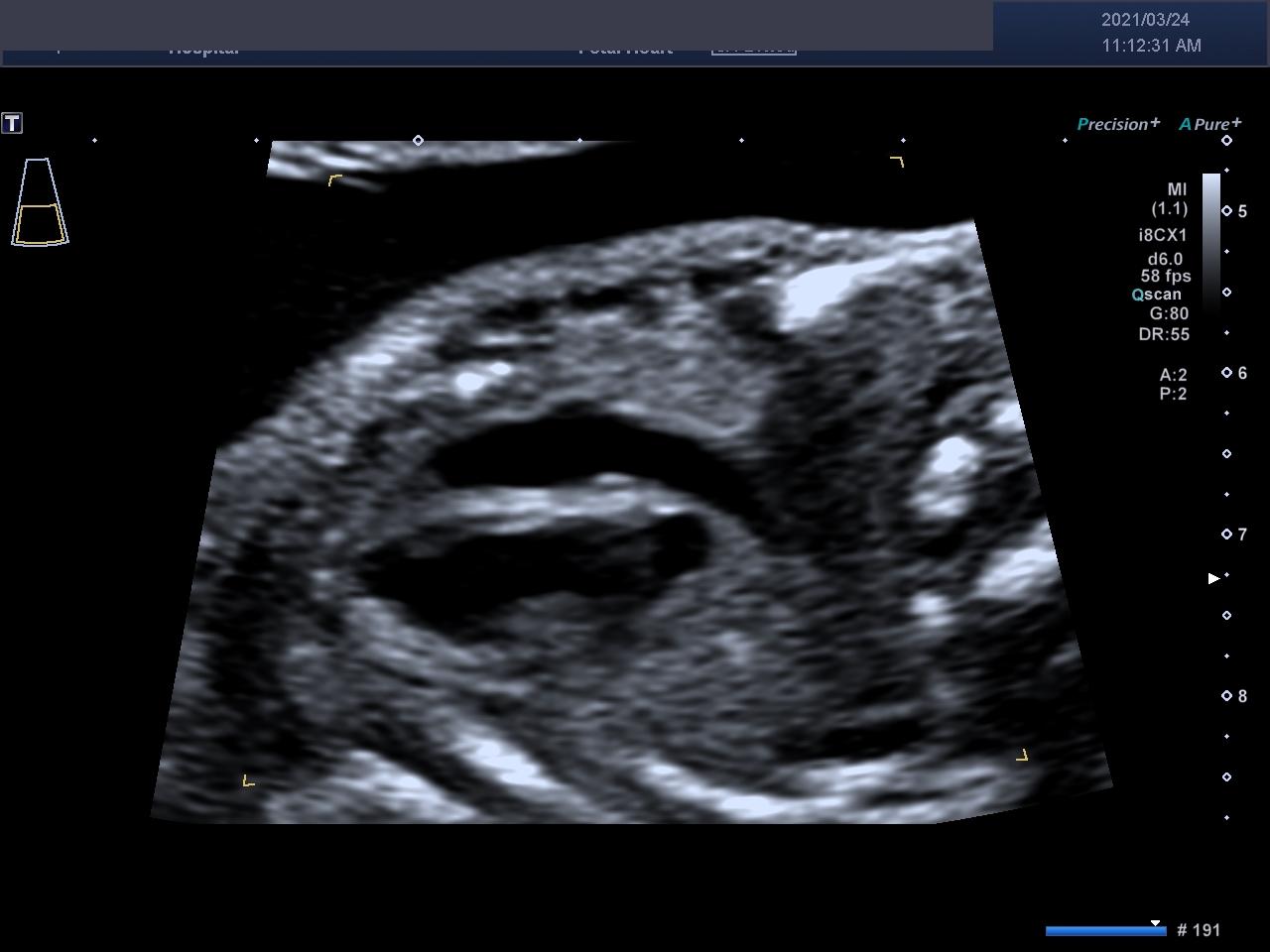

Supplement: S1 Dataset — (ZIP) [file pone.0305250.s001.zip › FE-SD-1/images/train_res/115_ro.jpg]

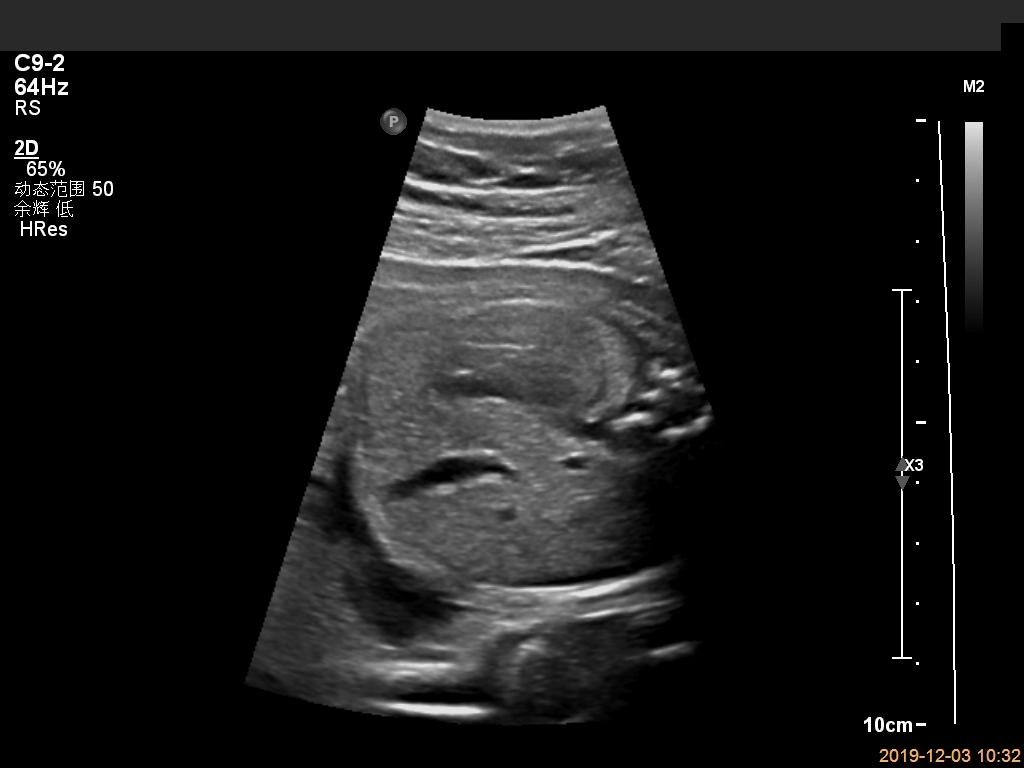

Supplement: S1 Dataset — (ZIP) [file pone.0305250.s001.zip › FE-SD-1/images/train_res/1160_ab.jpg]

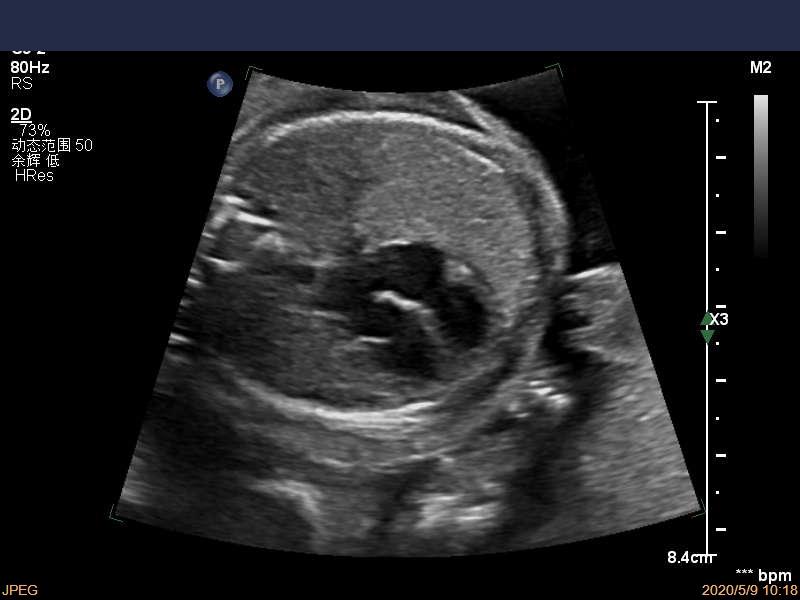

Supplement: S1 Dataset — (ZIP) [file pone.0305250.s001.zip › FE-SD-1/images/train_res/1160_fc.jpg]

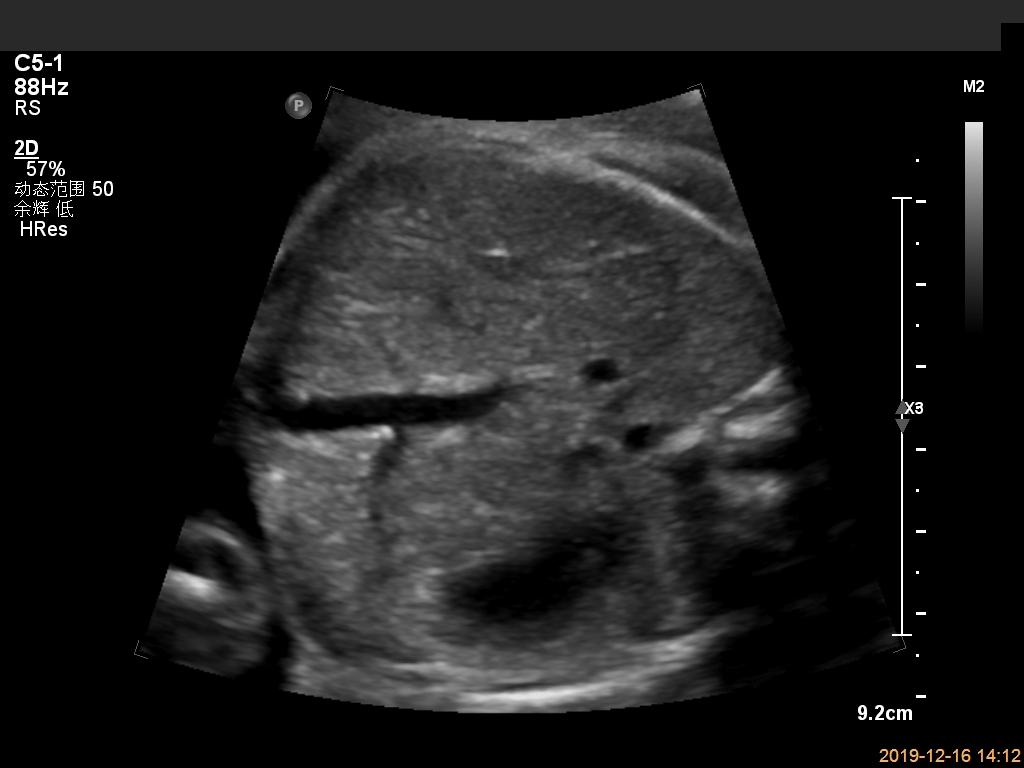

Supplement: S1 Dataset — (ZIP) [file pone.0305250.s001.zip › FE-SD-1/images/train_res/1161_ab.jpg]

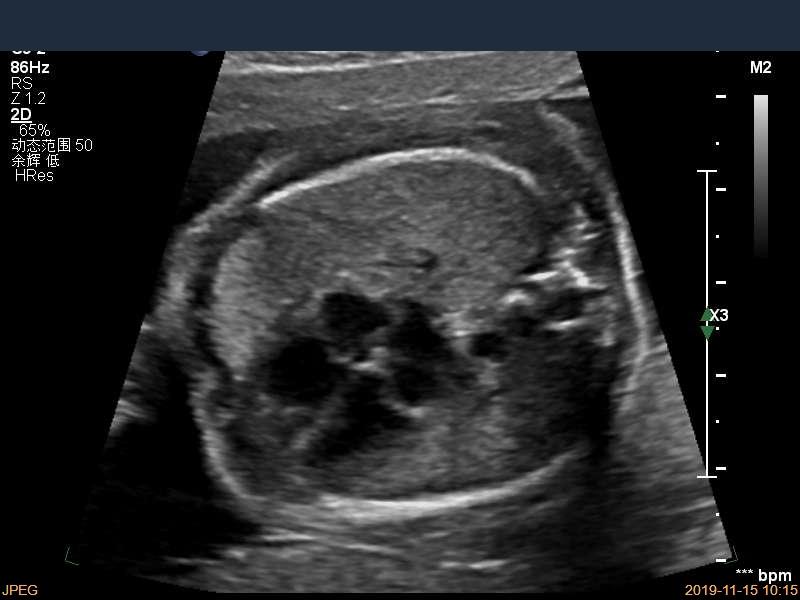

Supplement: S1 Dataset — (ZIP) [file pone.0305250.s001.zip › FE-SD-1/images/train_res/1163_fc.jpg]

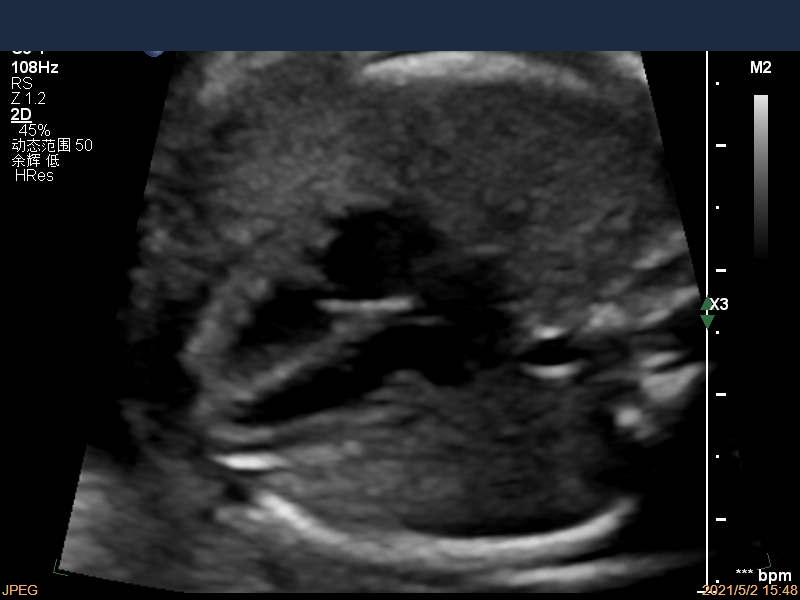

Supplement: S1 Dataset — (ZIP) [file pone.0305250.s001.zip › FE-SD-1/images/train_res/1164_fc.jpg]

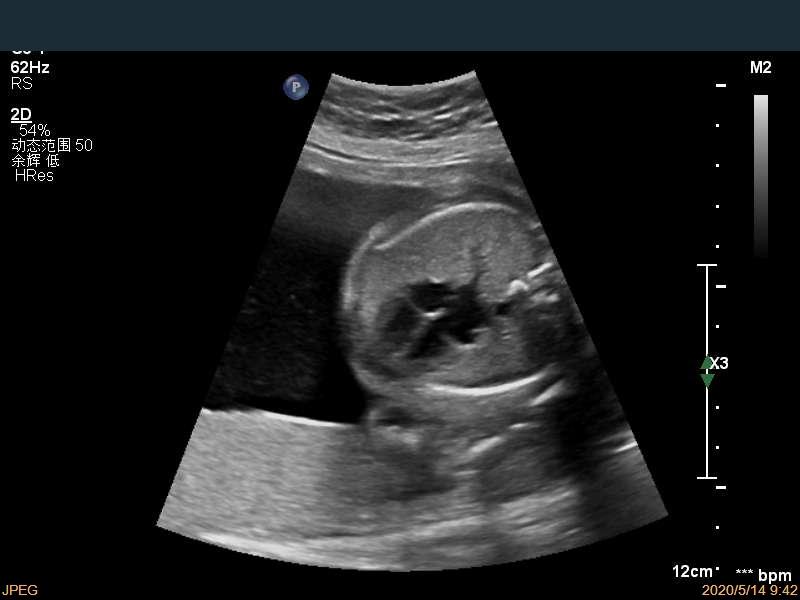

Supplement: S1 Dataset — (ZIP) [file pone.0305250.s001.zip › FE-SD-1/images/train_res/1165_fc.jpg]

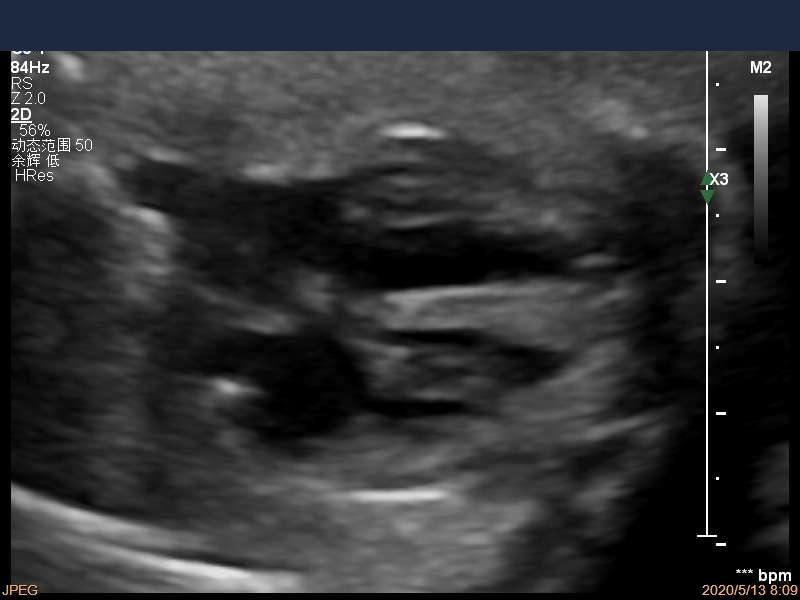

Supplement: S1 Dataset — (ZIP) [file pone.0305250.s001.zip › FE-SD-1/images/train_res/1166_fc.jpg]

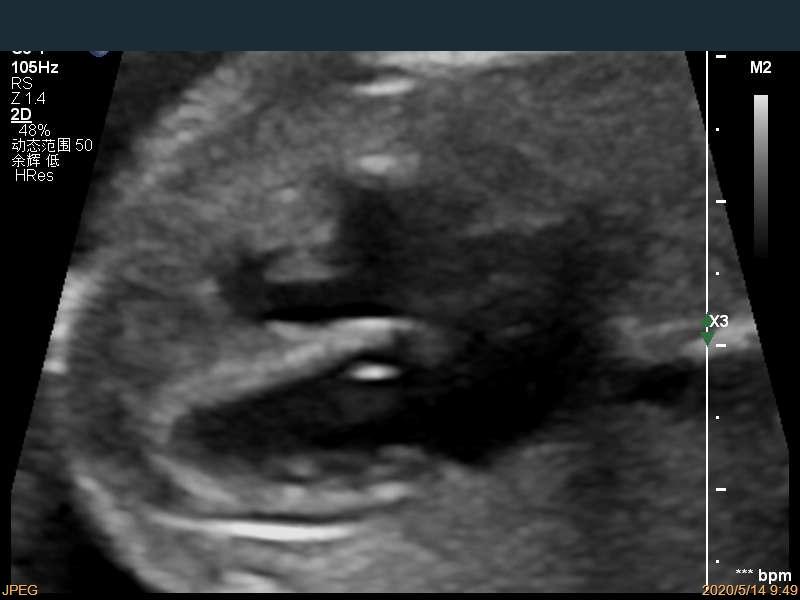

Supplement: S1 Dataset — (ZIP) [file pone.0305250.s001.zip › FE-SD-1/images/train_res/1167_fc.jpg]

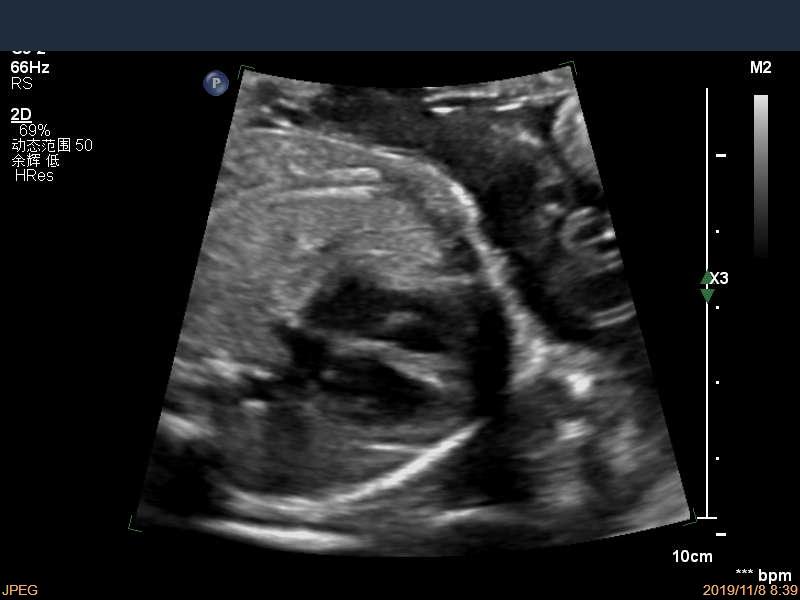

Supplement: S1 Dataset — (ZIP) [file pone.0305250.s001.zip › FE-SD-1/images/train_res/1169_fc.jpg]

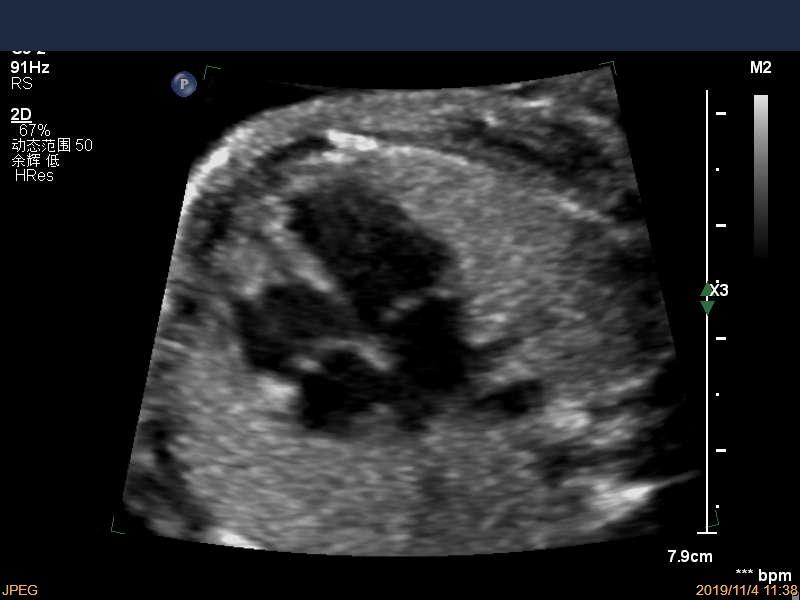

Supplement: S1 Dataset — (ZIP) [file pone.0305250.s001.zip › FE-SD-1/images/train_res/1170_fc.jpg]

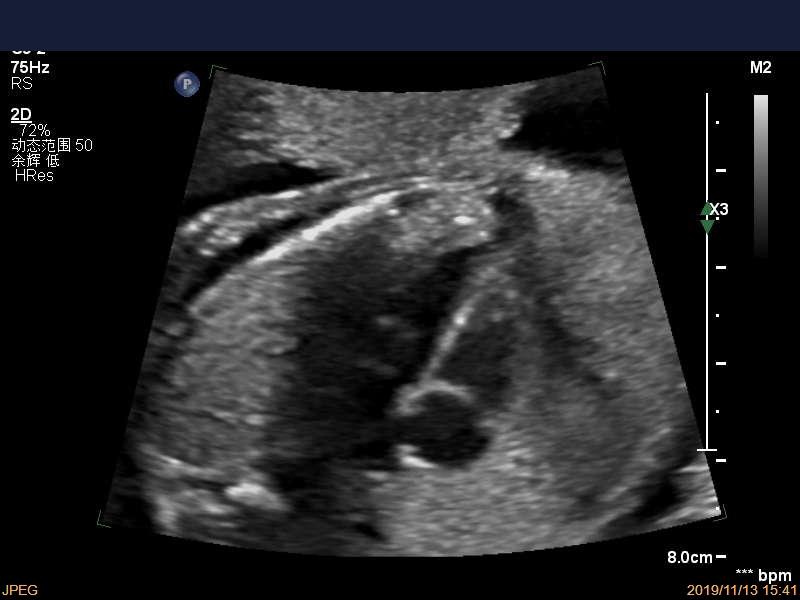

Supplement: S1 Dataset — (ZIP) [file pone.0305250.s001.zip › FE-SD-1/images/train_res/1171_fc.jpg]

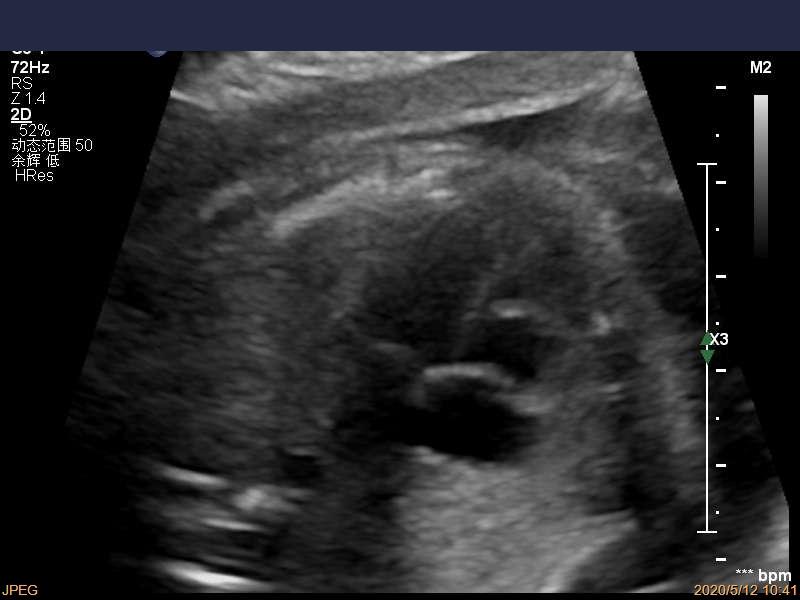

Supplement: S1 Dataset — (ZIP) [file pone.0305250.s001.zip › FE-SD-1/images/train_res/1172_fc.jpg]

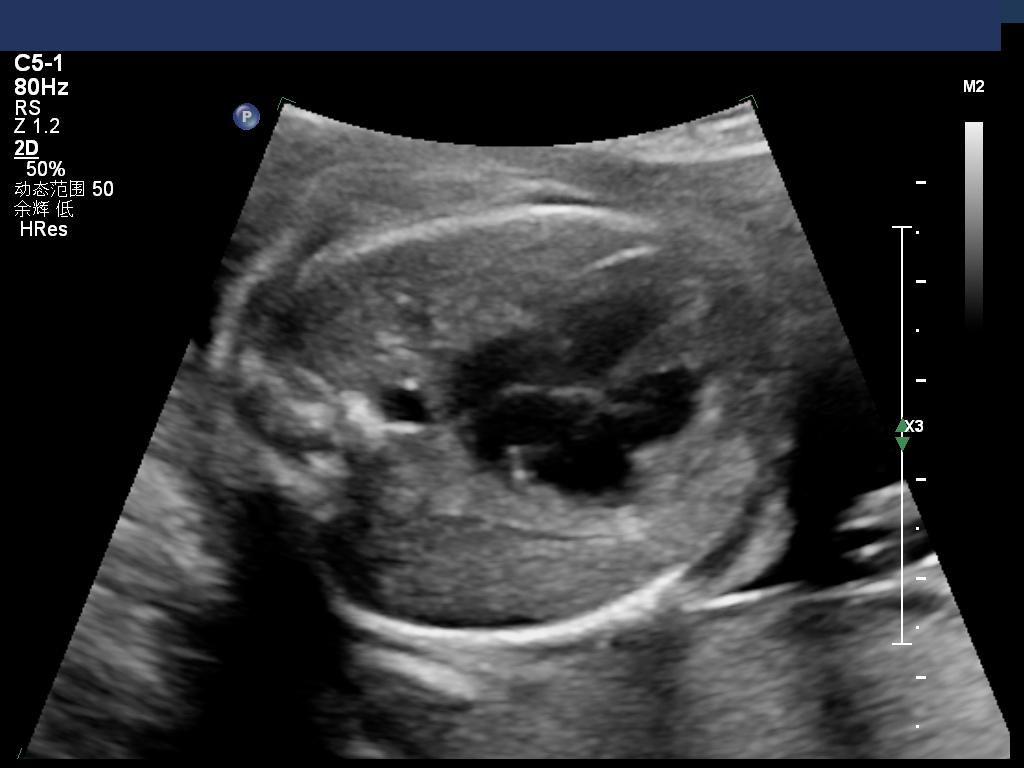

Supplement: S1 Dataset — (ZIP) [file pone.0305250.s001.zip › FE-SD-1/images/train_res/1175_fc.jpg]

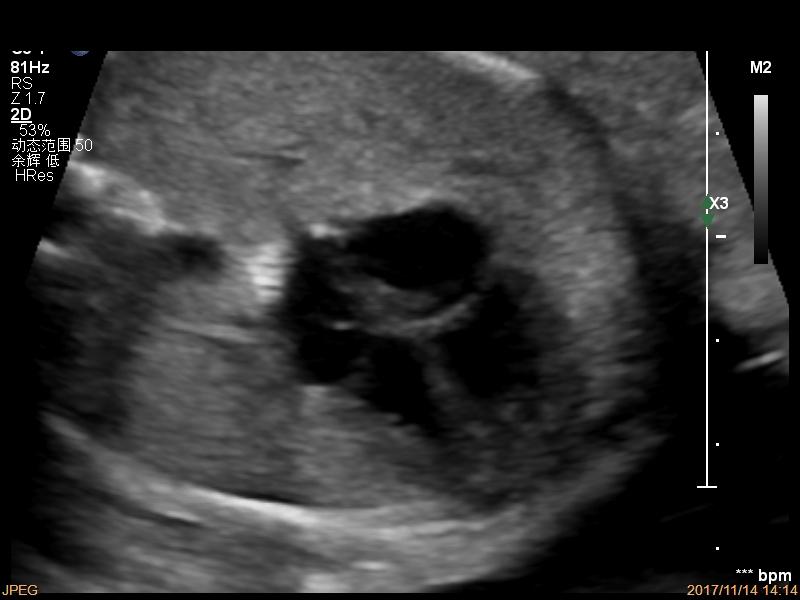

Supplement: S1 Dataset — (ZIP) [file pone.0305250.s001.zip › FE-SD-1/images/train_res/1177_fc.jpg]

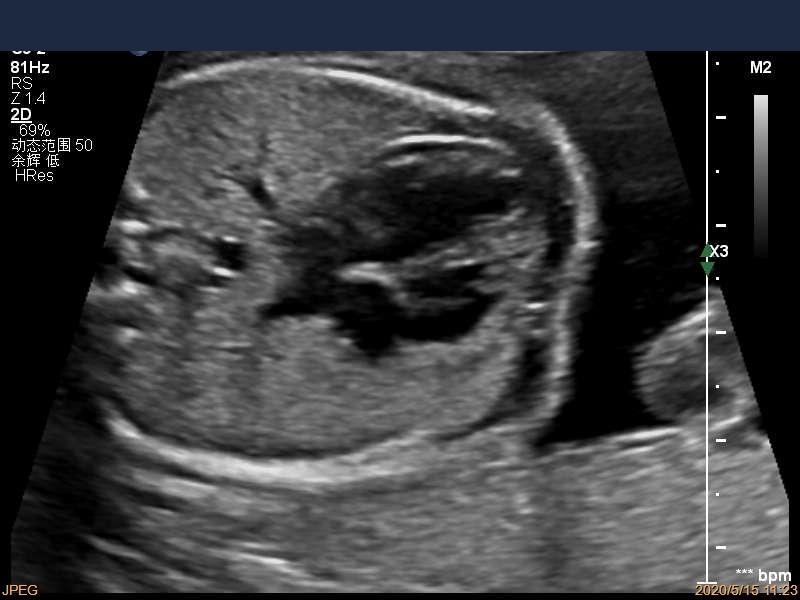

Supplement: S1 Dataset — (ZIP) [file pone.0305250.s001.zip › FE-SD-1/images/train_res/1178_fc.jpg]

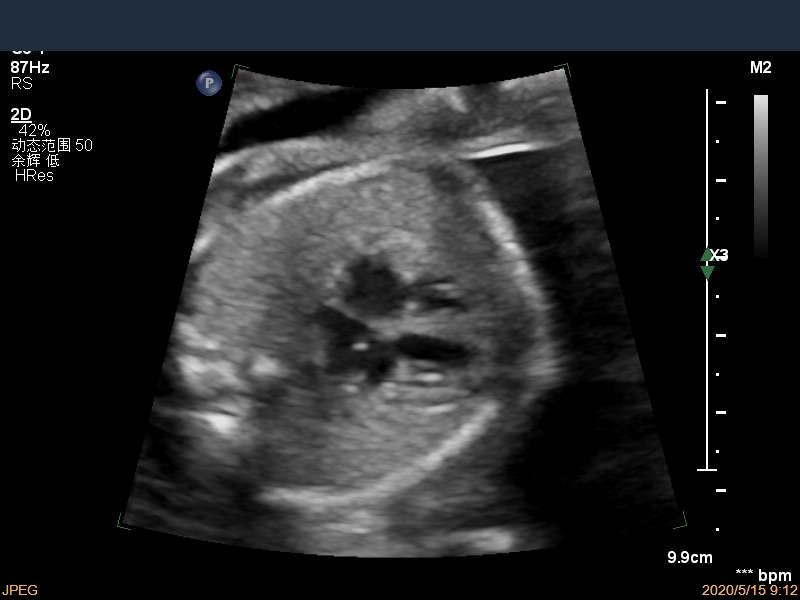

Supplement: S1 Dataset — (ZIP) [file pone.0305250.s001.zip › FE-SD-1/images/train_res/1180_fc.jpg]

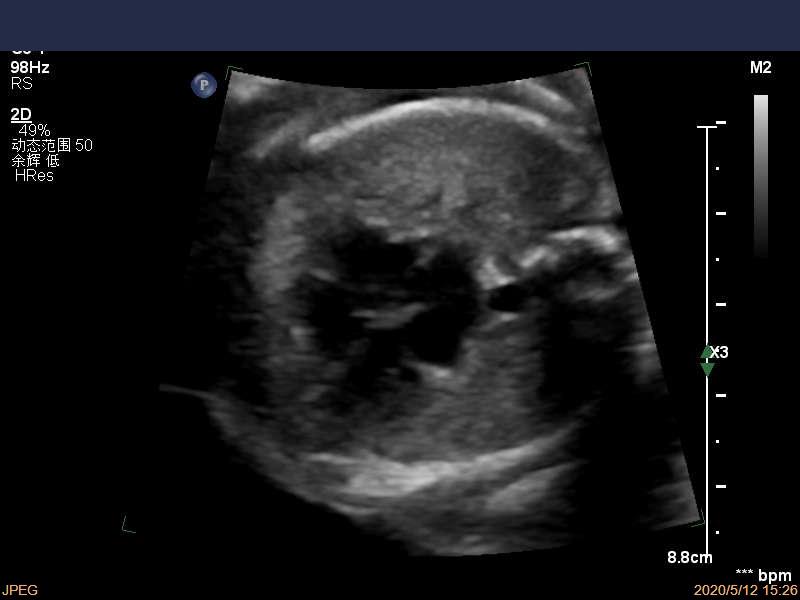

Supplement: S1 Dataset — (ZIP) [file pone.0305250.s001.zip › FE-SD-1/images/train_res/1181_fc.jpg]

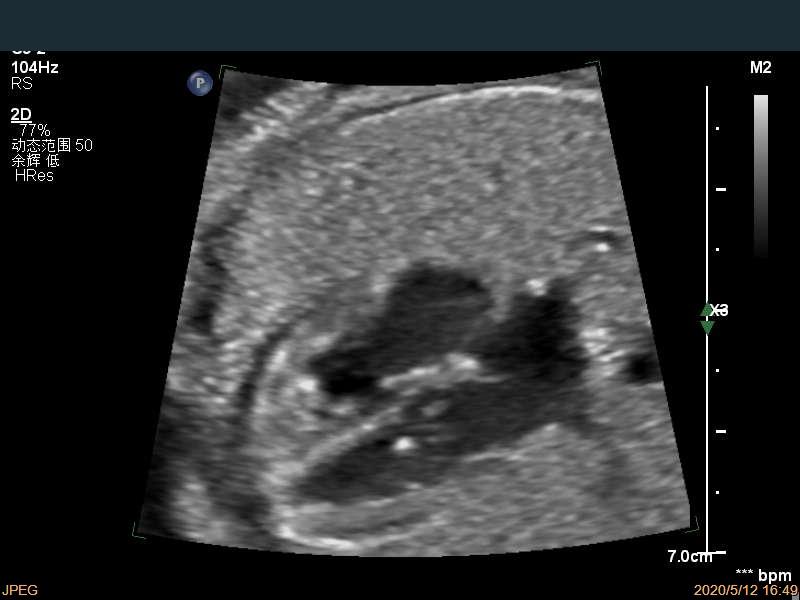

Supplement: S1 Dataset — (ZIP) [file pone.0305250.s001.zip › FE-SD-1/images/train_res/1183_fc.jpg]

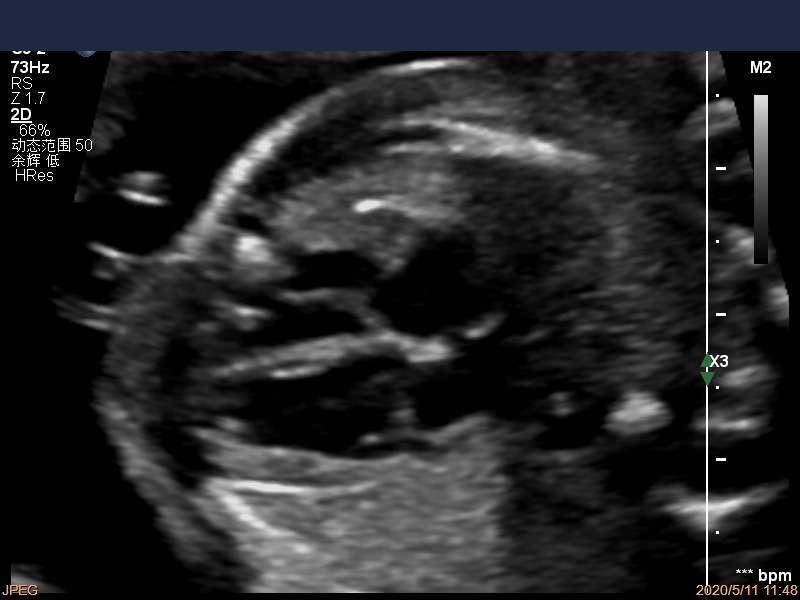

Supplement: S1 Dataset — (ZIP) [file pone.0305250.s001.zip › FE-SD-1/images/train_res/1184_fc.jpg]

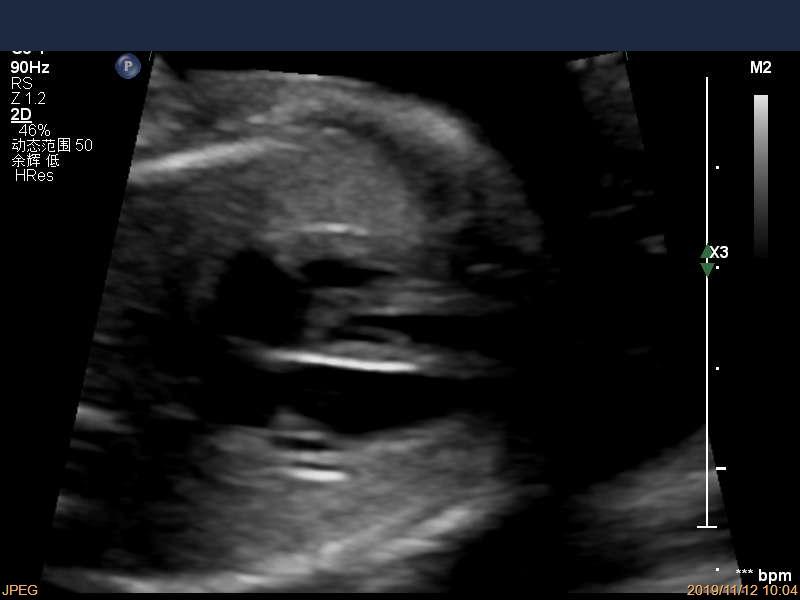

Supplement: S1 Dataset — (ZIP) [file pone.0305250.s001.zip › FE-SD-1/images/train_res/1185_fc.jpg]

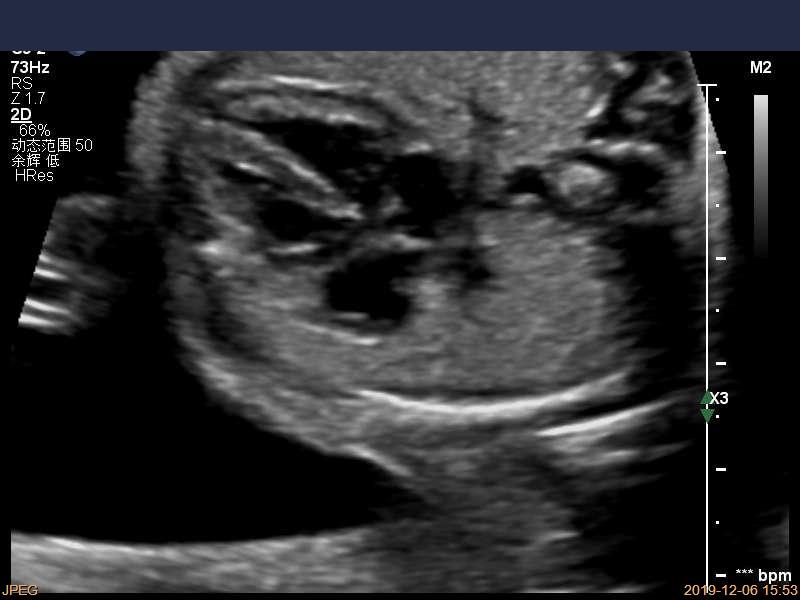

Supplement: S1 Dataset — (ZIP) [file pone.0305250.s001.zip › FE-SD-1/images/train_res/1186_fc.jpg]

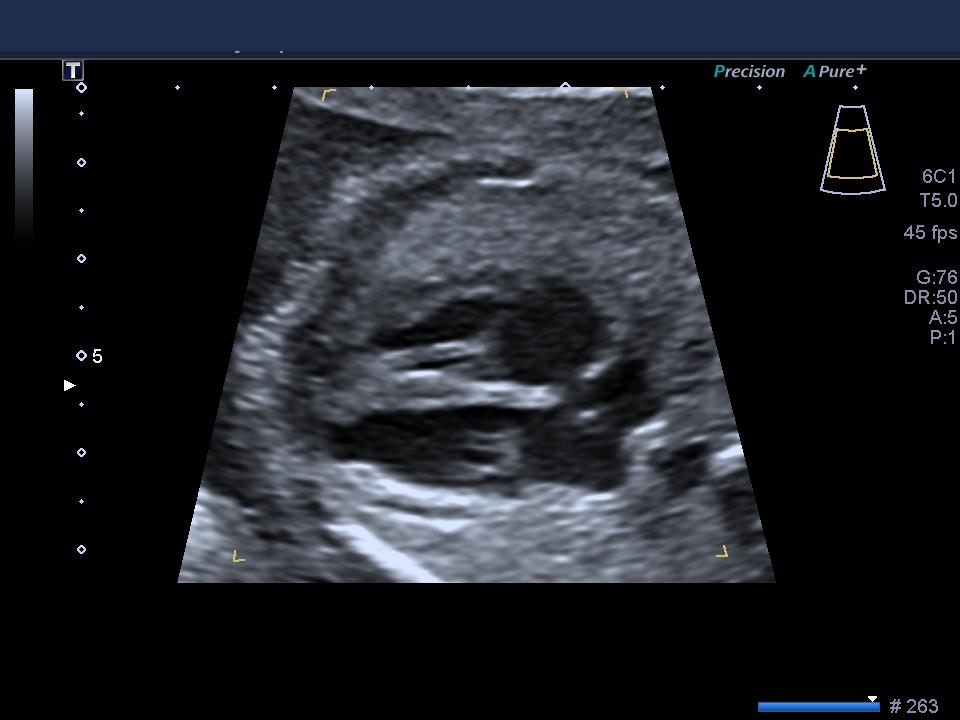

Supplement: S1 Dataset — (ZIP) [file pone.0305250.s001.zip › FE-SD-1/images/train_res/1188_fc.jpg]

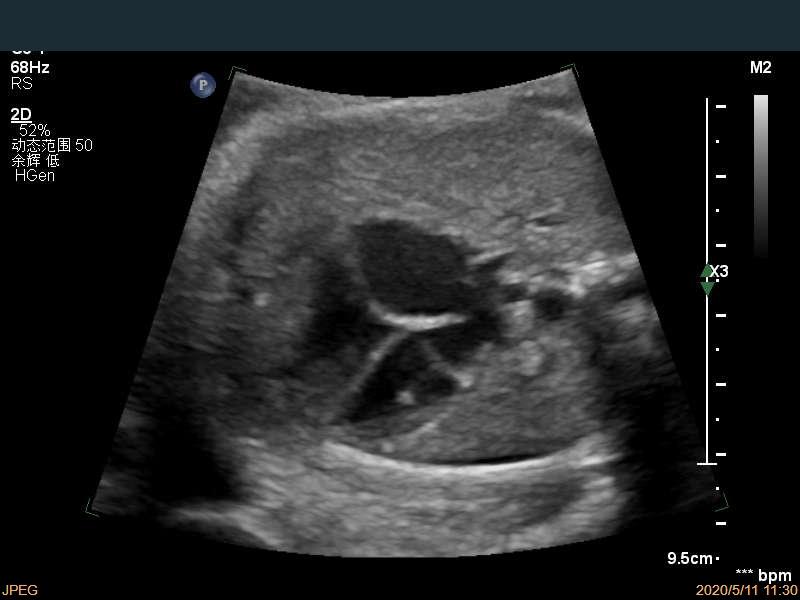

Supplement: S1 Dataset — (ZIP) [file pone.0305250.s001.zip › FE-SD-1/images/train_res/1189_fc.jpg]

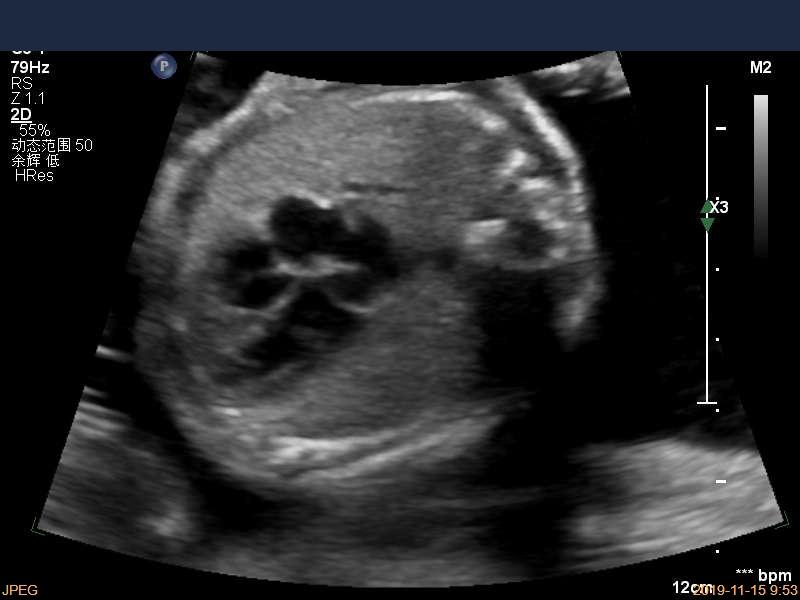

Supplement: S1 Dataset — (ZIP) [file pone.0305250.s001.zip › FE-SD-1/images/train_res/1190_fc.jpg]

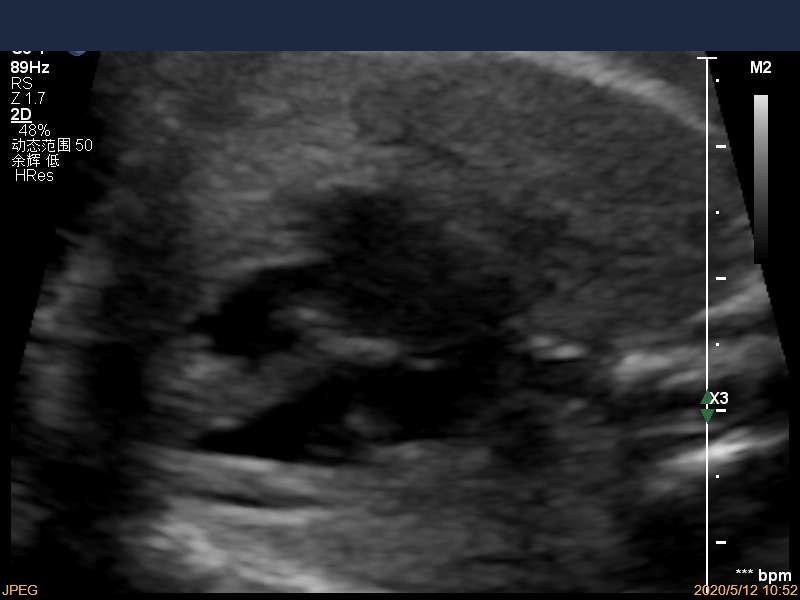

Supplement: S1 Dataset — (ZIP) [file pone.0305250.s001.zip › FE-SD-1/images/train_res/1191_fc.jpg]

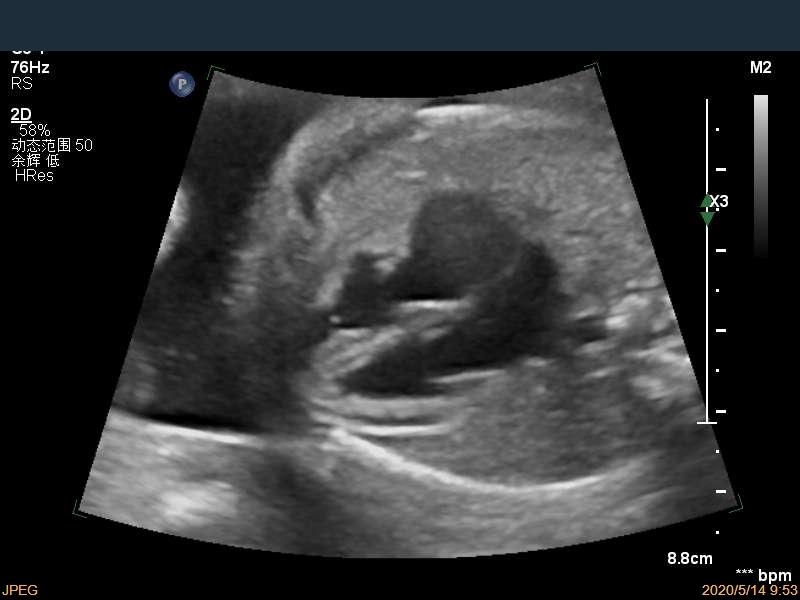

Supplement: S1 Dataset — (ZIP) [file pone.0305250.s001.zip › FE-SD-1/images/train_res/1192_fc.jpg]

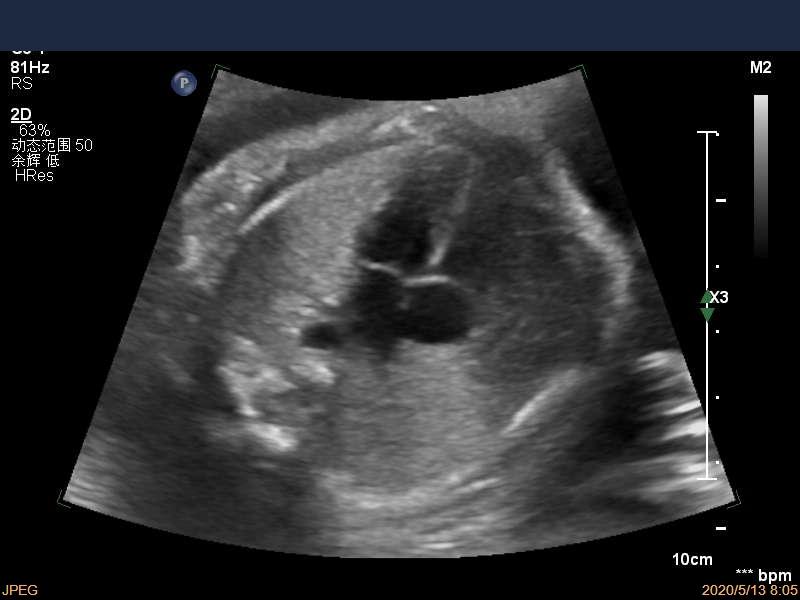

Supplement: S1 Dataset — (ZIP) [file pone.0305250.s001.zip › FE-SD-1/images/train_res/1193_fc.jpg]

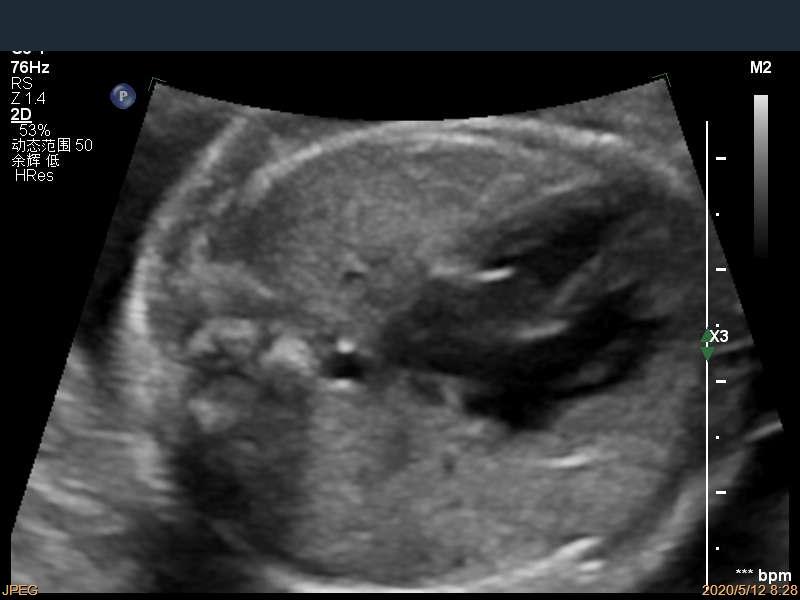

Supplement: S1 Dataset — (ZIP) [file pone.0305250.s001.zip › FE-SD-1/images/train_res/1194_fc.jpg]

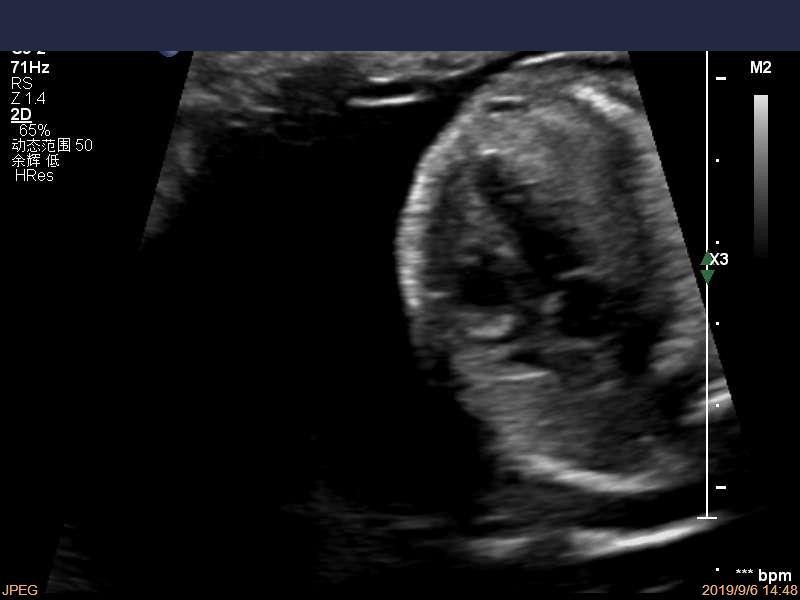

Supplement: S1 Dataset — (ZIP) [file pone.0305250.s001.zip › FE-SD-1/images/train_res/1195_fc.jpg]

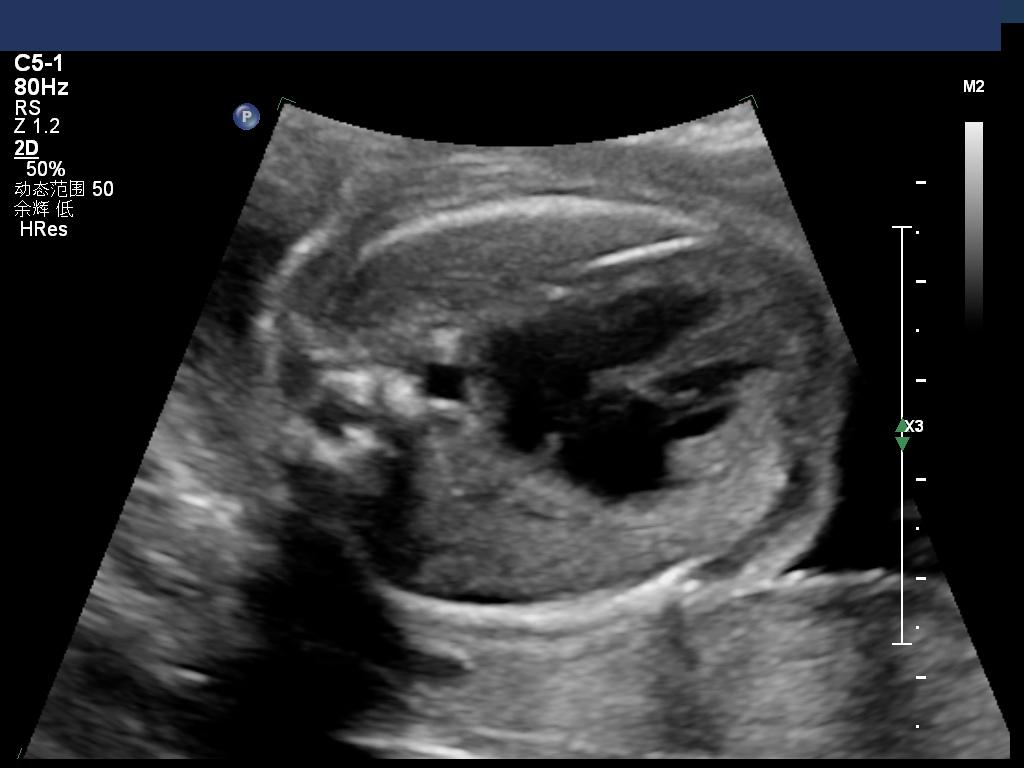

Supplement: S1 Dataset — (ZIP) [file pone.0305250.s001.zip › FE-SD-1/images/train_res/1197_fc.jpg]

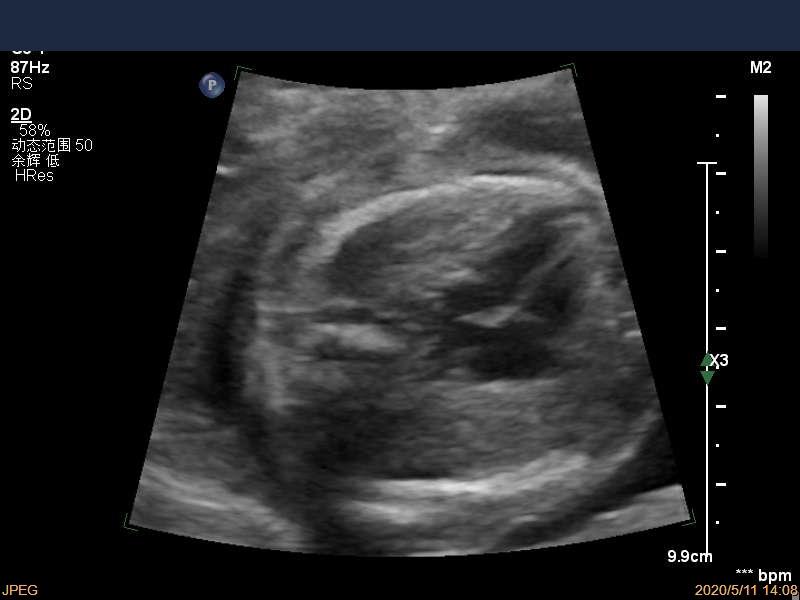

Supplement: S1 Dataset — (ZIP) [file pone.0305250.s001.zip › FE-SD-1/images/train_res/1198_fc.jpg]

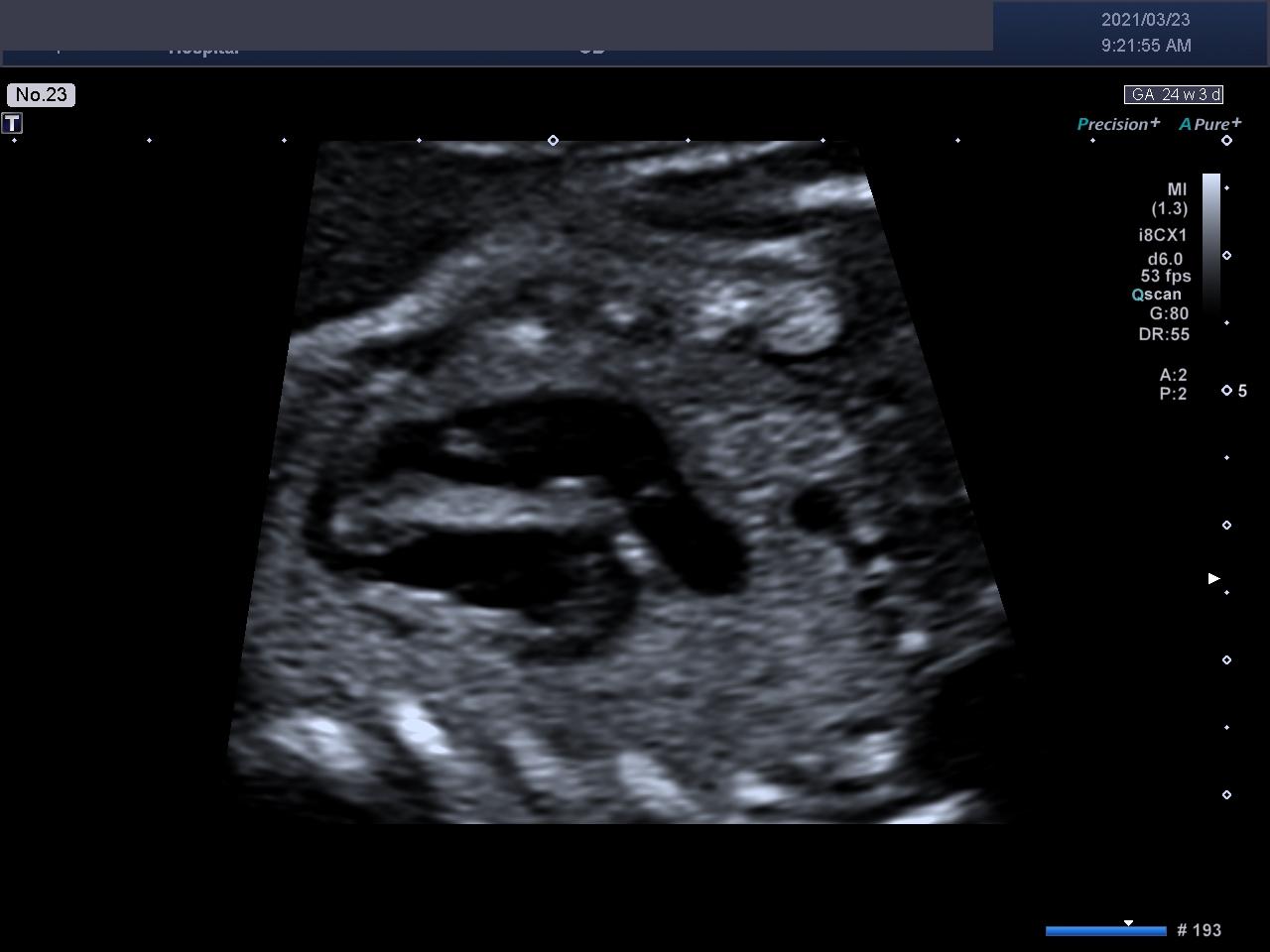

Supplement: S1 Dataset — (ZIP) [file pone.0305250.s001.zip › FE-SD-1/images/train_res/119_ro.jpg]

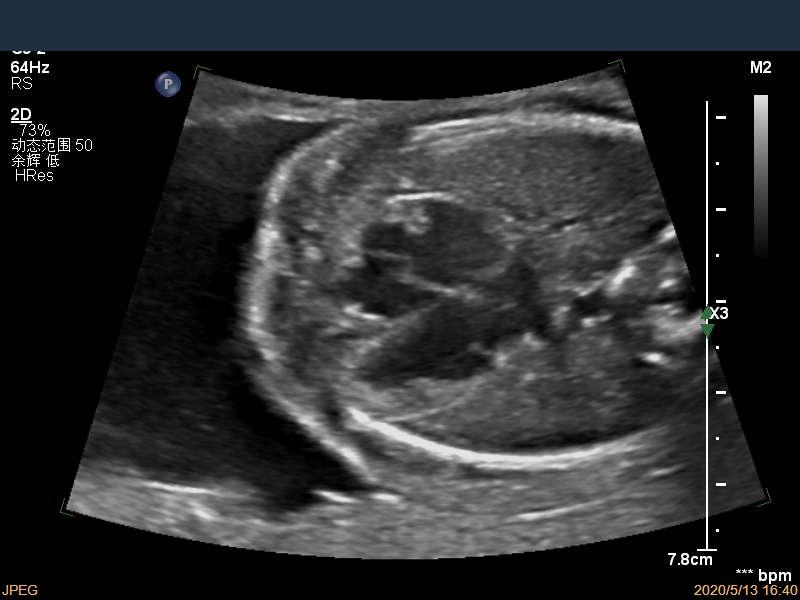

Supplement: S1 Dataset — (ZIP) [file pone.0305250.s001.zip › FE-SD-1/images/train_res/1200_fc.jpg]

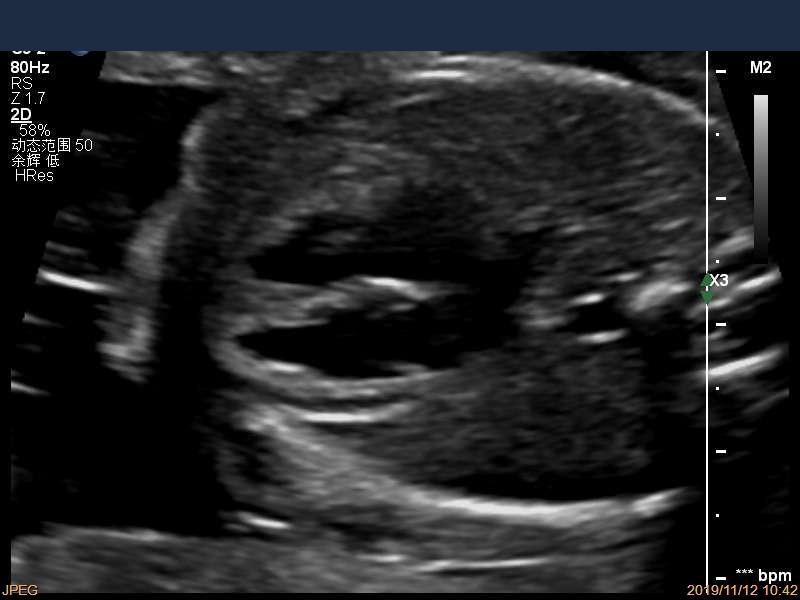

Supplement: S1 Dataset — (ZIP) [file pone.0305250.s001.zip › FE-SD-1/images/train_res/1203_fc.jpg]

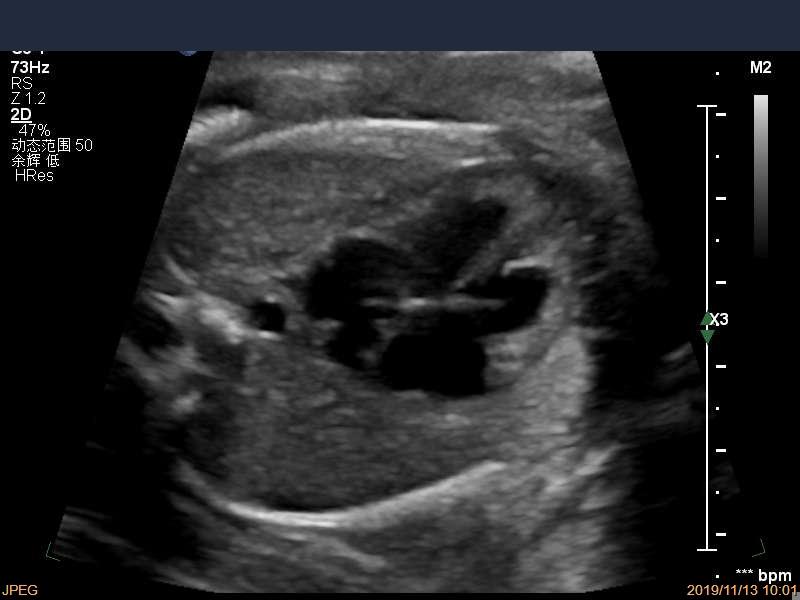

Supplement: S1 Dataset — (ZIP) [file pone.0305250.s001.zip › FE-SD-1/images/train_res/1204_fc.jpg]

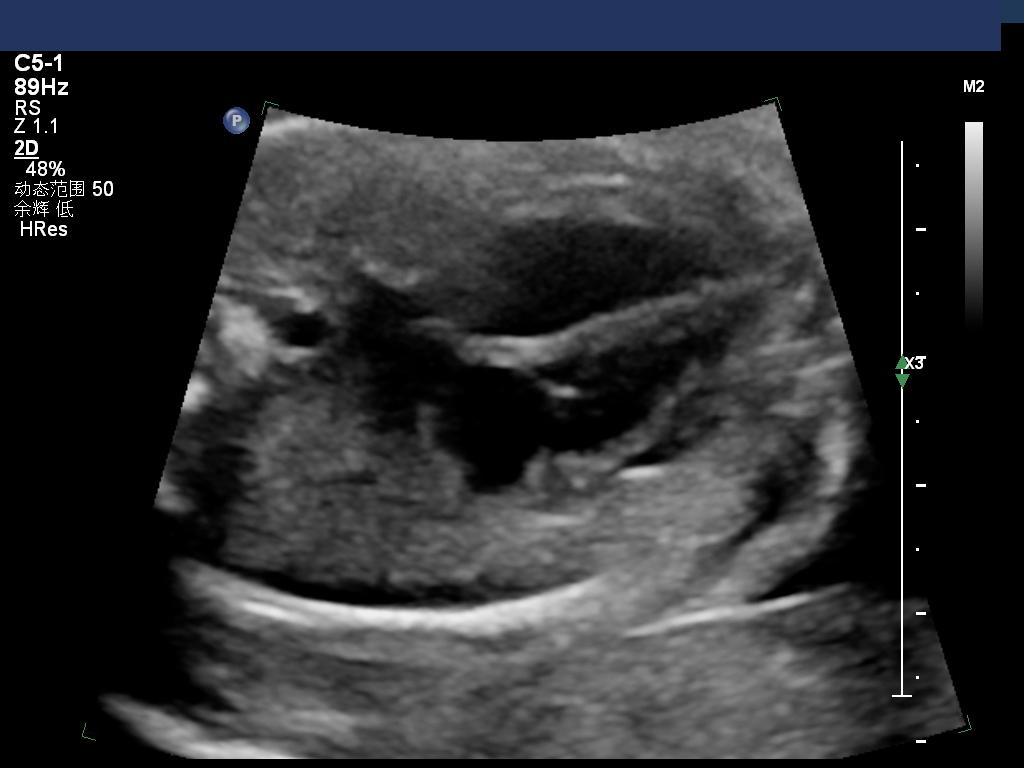

Supplement: S1 Dataset — (ZIP) [file pone.0305250.s001.zip › FE-SD-1/images/train_res/1208_fc.jpg]

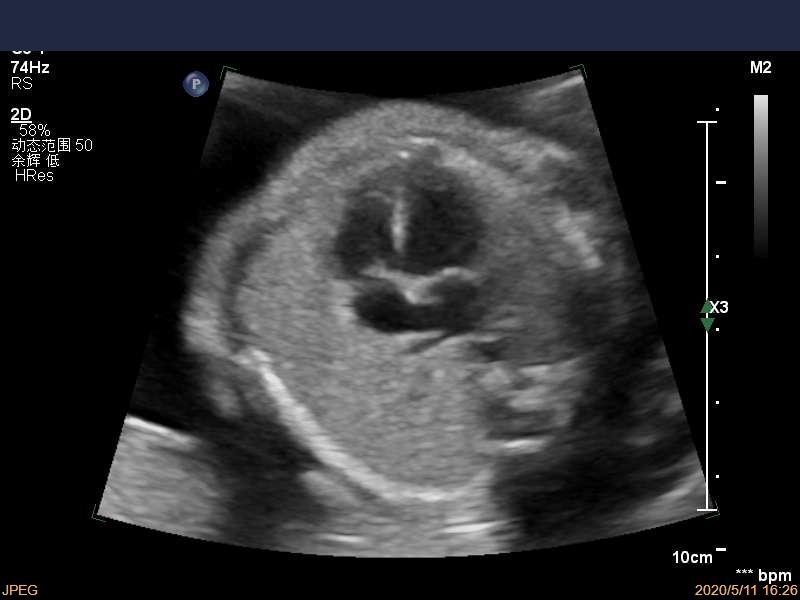

Supplement: S1 Dataset — (ZIP) [file pone.0305250.s001.zip › FE-SD-1/images/train_res/1209_fc.jpg]
