# Supplementary figures and images for: Implementation of resource-efficient fetal echocardiography detection algorithms in edge computing (part 1 of 4)
Source: PLoS One. 2024 Sep 23;19(9):e0305250. doi: 10.1371/journal.pone.0305250 (PMC11419364; doi:10.1371/journal.pone.0305250)

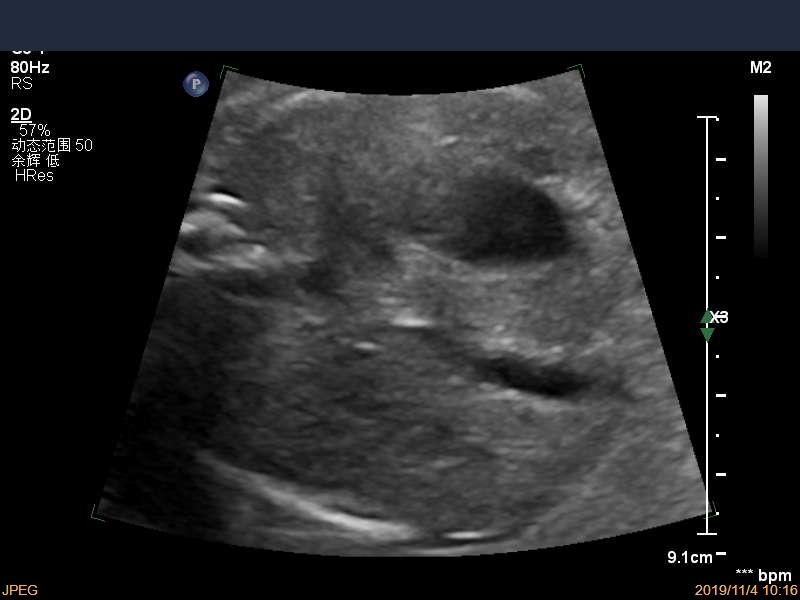

Supplement: S1 Dataset — (ZIP) [file pone.0305250.s001.zip › FE-SD-1/images/train_res/1000_ab.jpg]

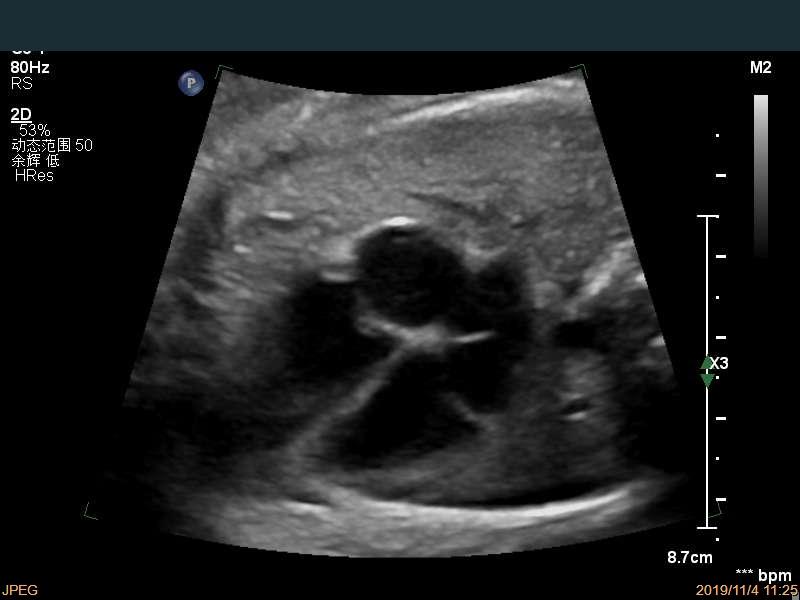

Supplement: S1 Dataset — (ZIP) [file pone.0305250.s001.zip › FE-SD-1/images/train_res/1000_fc.jpg]

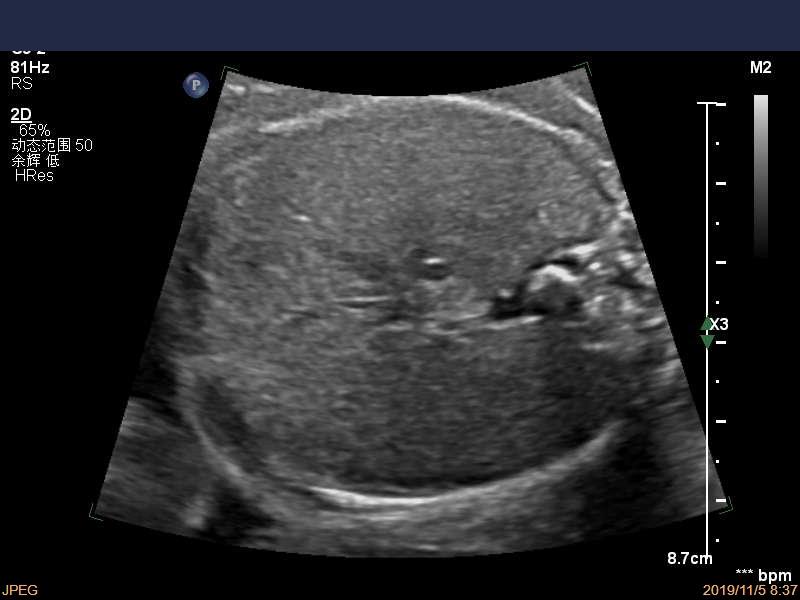

Supplement: S1 Dataset — (ZIP) [file pone.0305250.s001.zip › FE-SD-1/images/train_res/1001_ab.jpg]

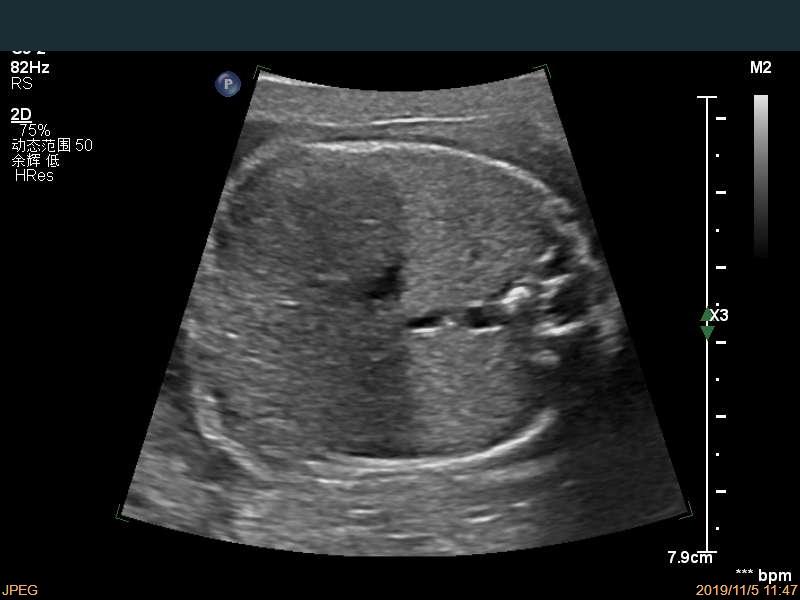

Supplement: S1 Dataset — (ZIP) [file pone.0305250.s001.zip › FE-SD-1/images/train_res/1002_ab.jpg]

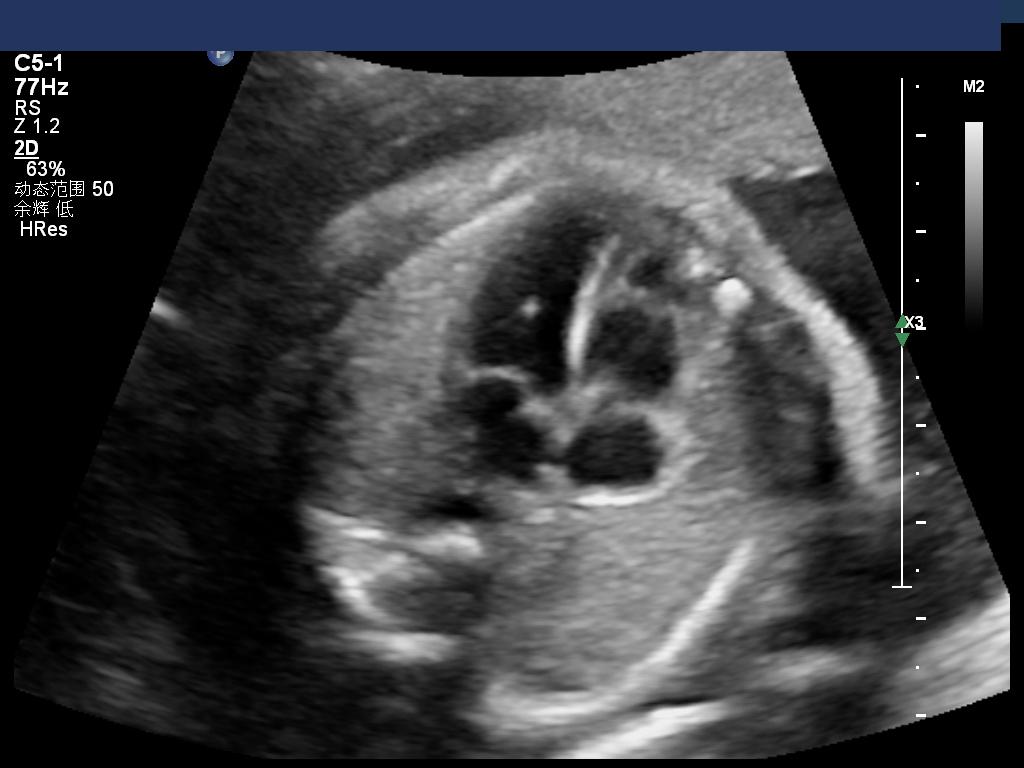

Supplement: S1 Dataset — (ZIP) [file pone.0305250.s001.zip › FE-SD-1/images/train_res/1002_fc.jpg]

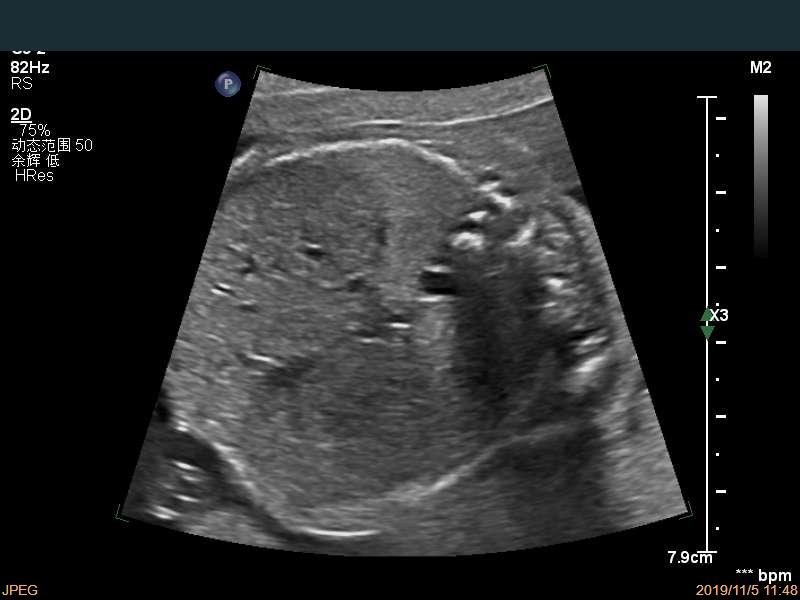

Supplement: S1 Dataset — (ZIP) [file pone.0305250.s001.zip › FE-SD-1/images/train_res/1003_ab.jpg]

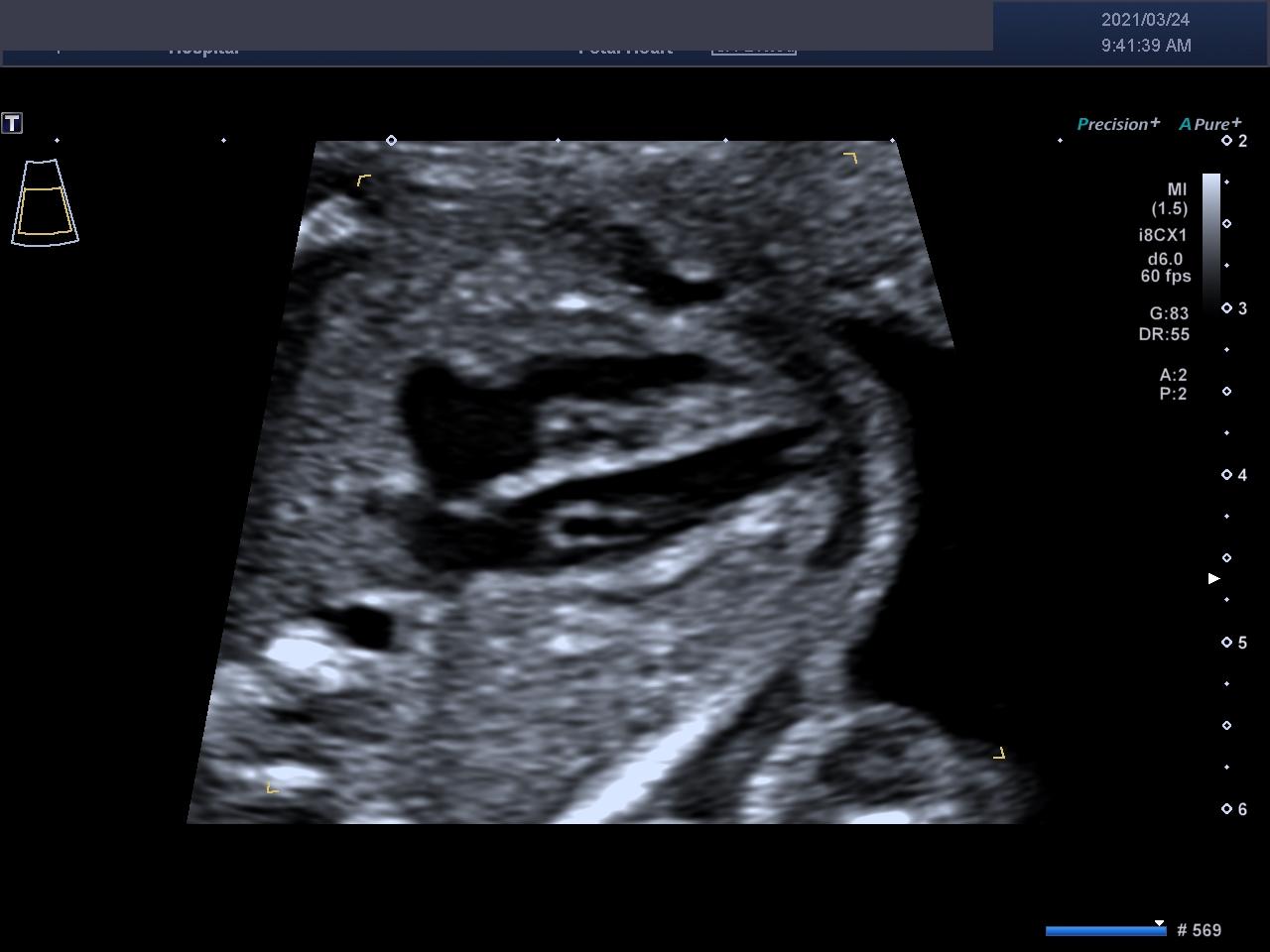

Supplement: S1 Dataset — (ZIP) [file pone.0305250.s001.zip › FE-SD-1/images/train_res/1003_fc.jpg]

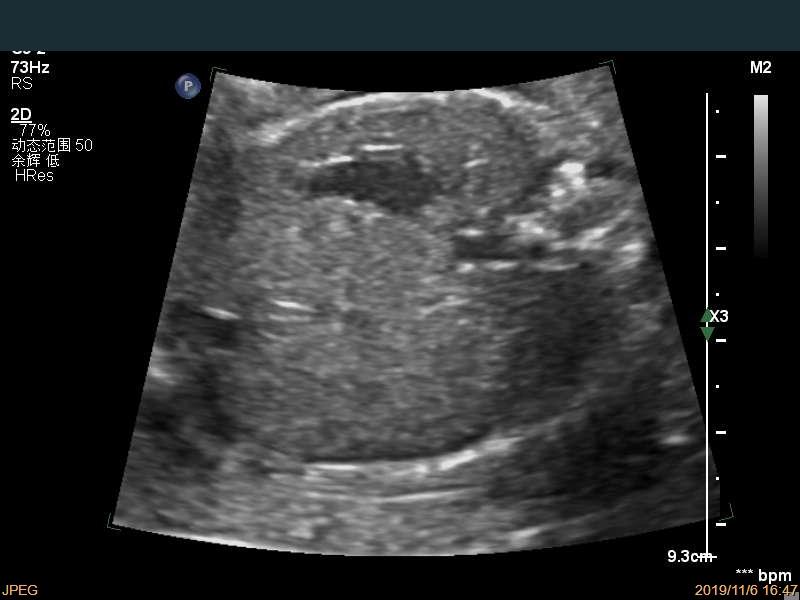

Supplement: S1 Dataset — (ZIP) [file pone.0305250.s001.zip › FE-SD-1/images/train_res/1004_ab.jpg]

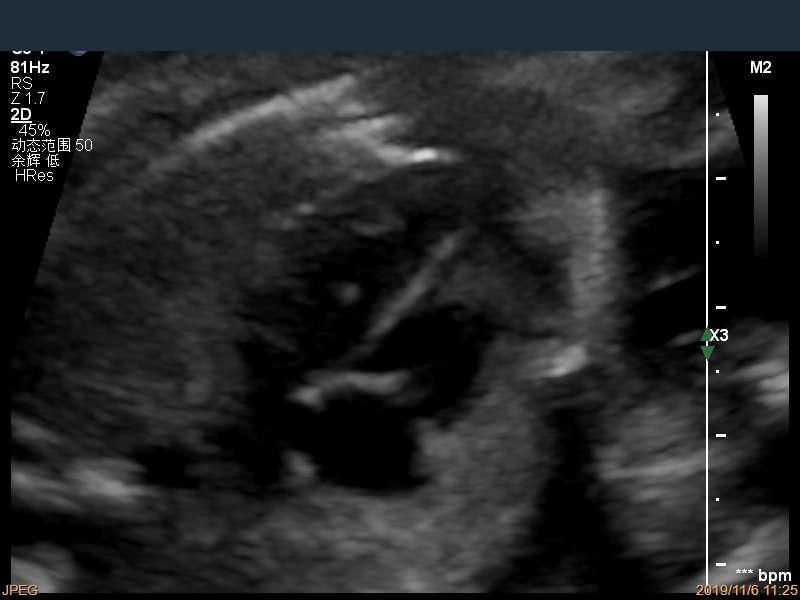

Supplement: S1 Dataset — (ZIP) [file pone.0305250.s001.zip › FE-SD-1/images/train_res/1004_fc.jpg]

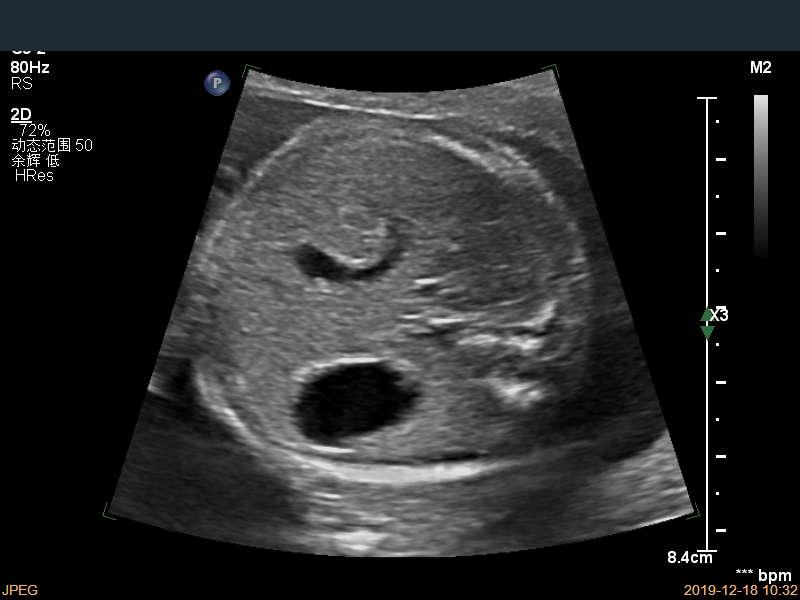

Supplement: S1 Dataset — (ZIP) [file pone.0305250.s001.zip › FE-SD-1/images/train_res/1005_ab.jpg]

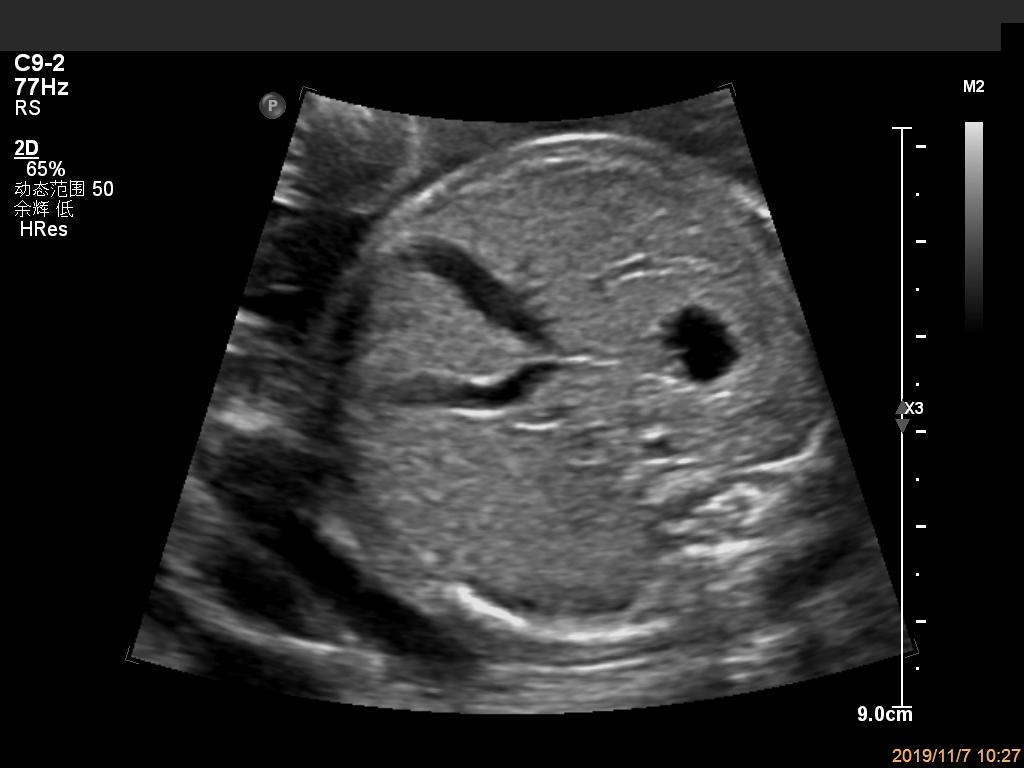

Supplement: S1 Dataset — (ZIP) [file pone.0305250.s001.zip › FE-SD-1/images/train_res/1007_ab.jpg]

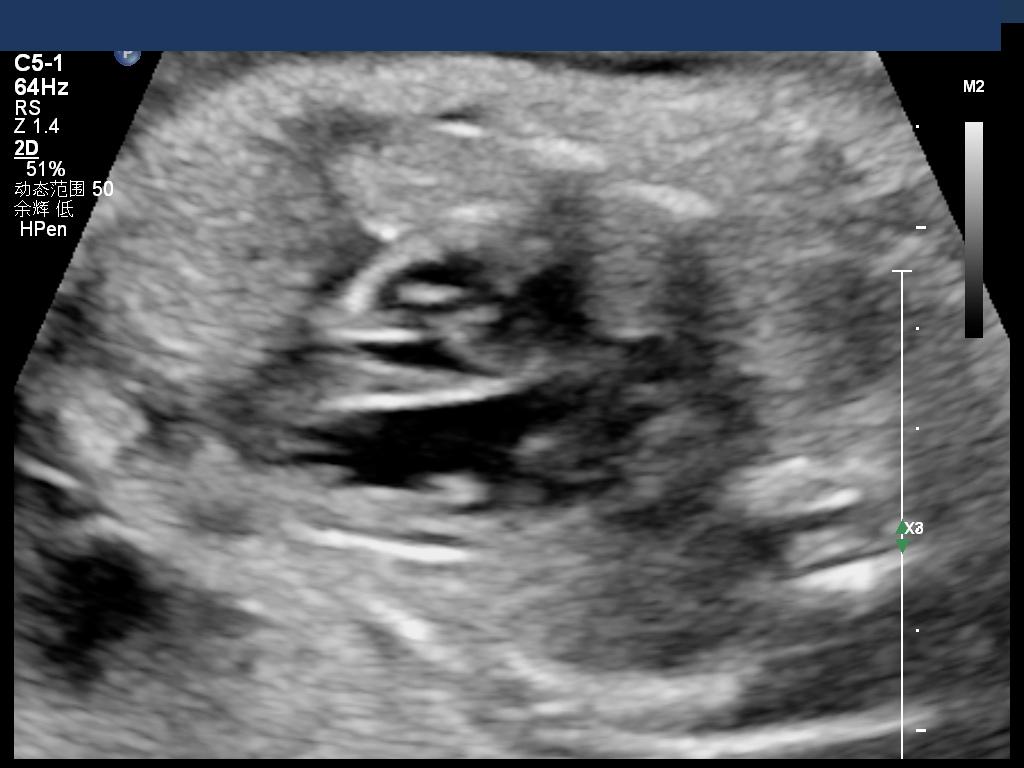

Supplement: S1 Dataset — (ZIP) [file pone.0305250.s001.zip › FE-SD-1/images/train_res/1008_fc.jpg]

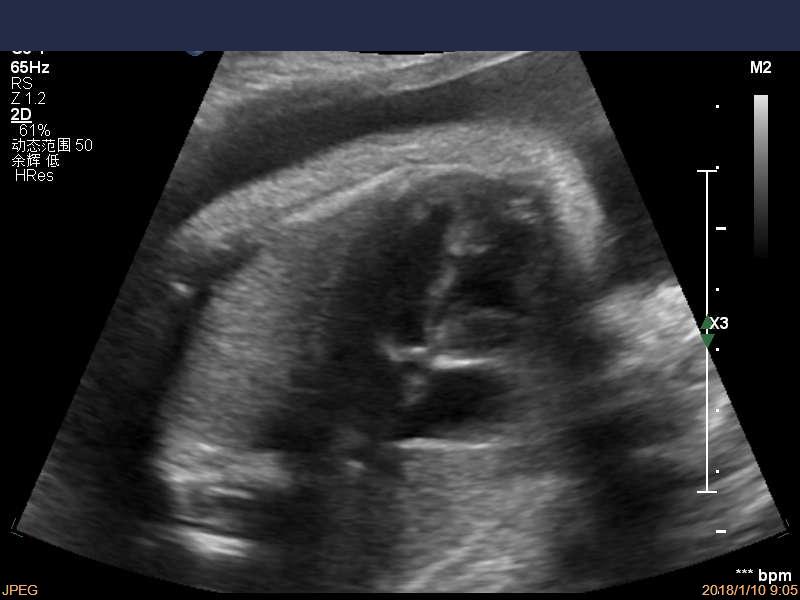

Supplement: S1 Dataset — (ZIP) [file pone.0305250.s001.zip › FE-SD-1/images/train_res/1009_fc.jpg]

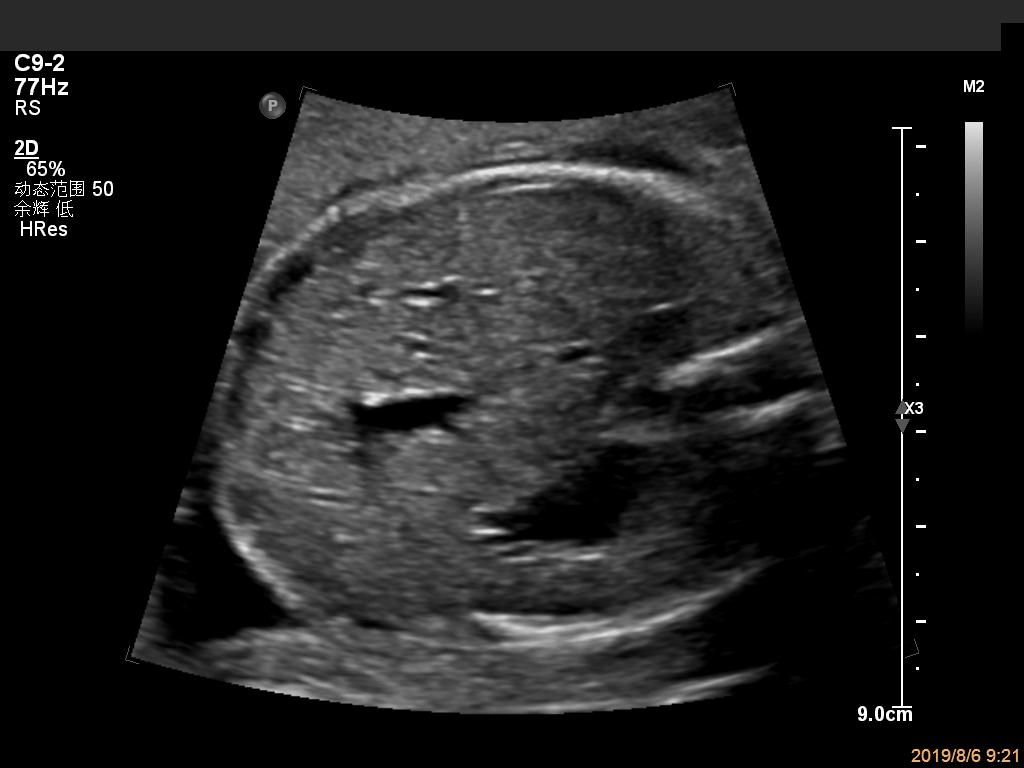

Supplement: S1 Dataset — (ZIP) [file pone.0305250.s001.zip › FE-SD-1/images/train_res/1010_ab.jpg]

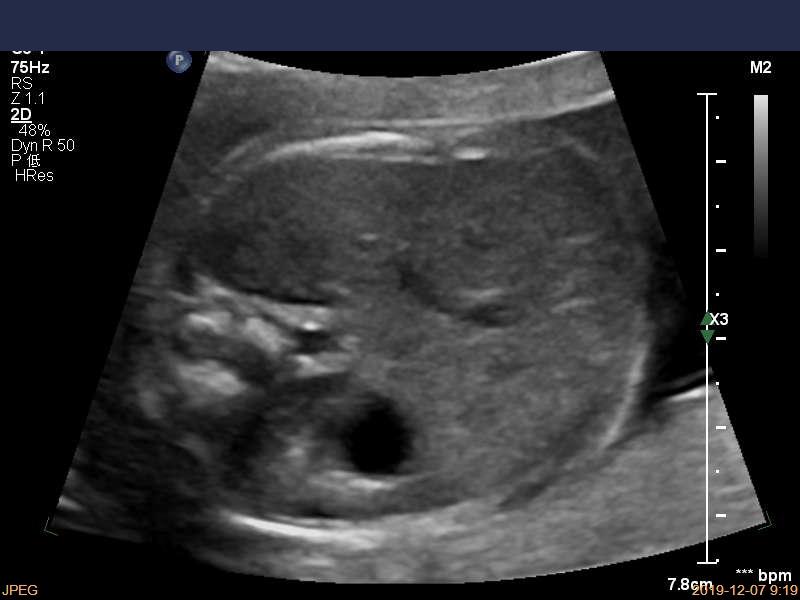

Supplement: S1 Dataset — (ZIP) [file pone.0305250.s001.zip › FE-SD-1/images/train_res/1011_ab.jpg]

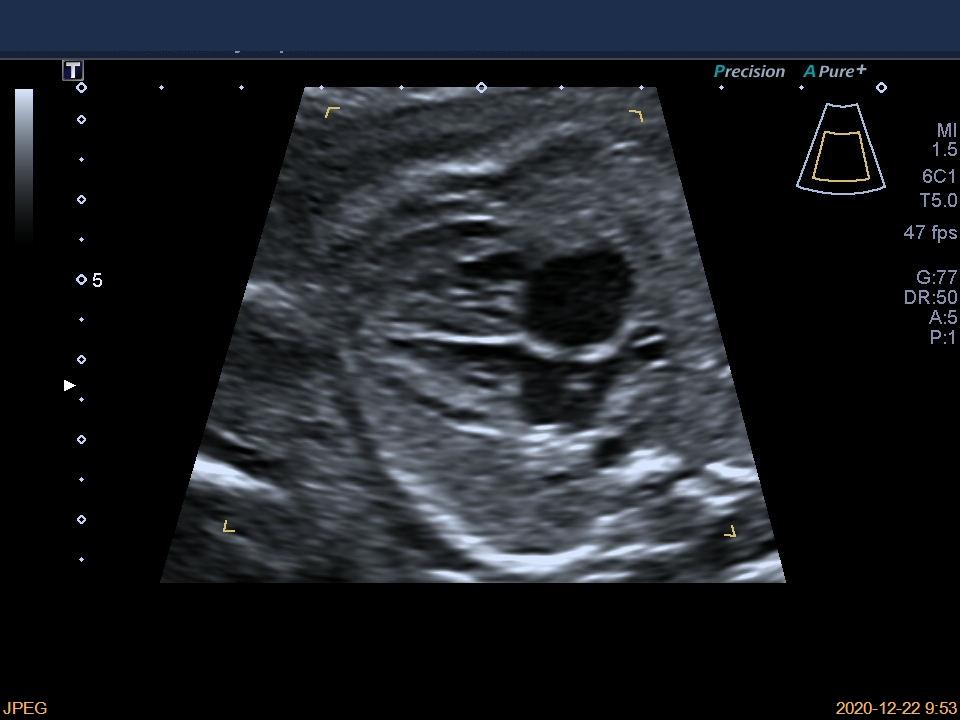

Supplement: S1 Dataset — (ZIP) [file pone.0305250.s001.zip › FE-SD-1/images/train_res/1012_fc.jpg]

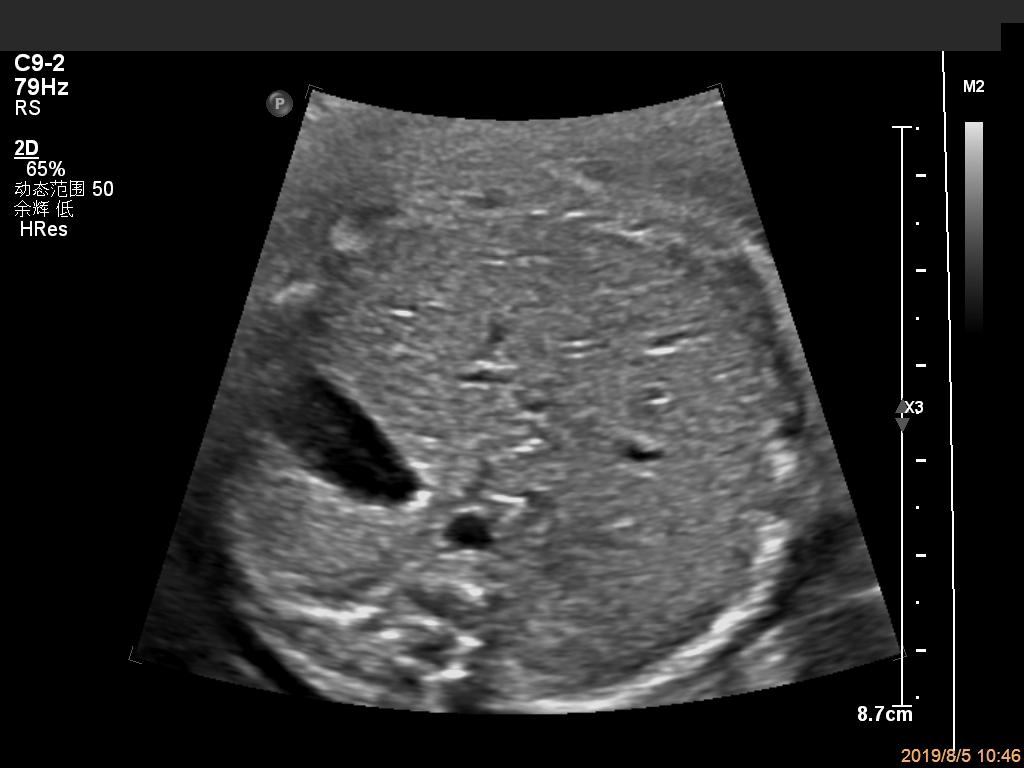

Supplement: S1 Dataset — (ZIP) [file pone.0305250.s001.zip › FE-SD-1/images/train_res/1013_ab.jpg]

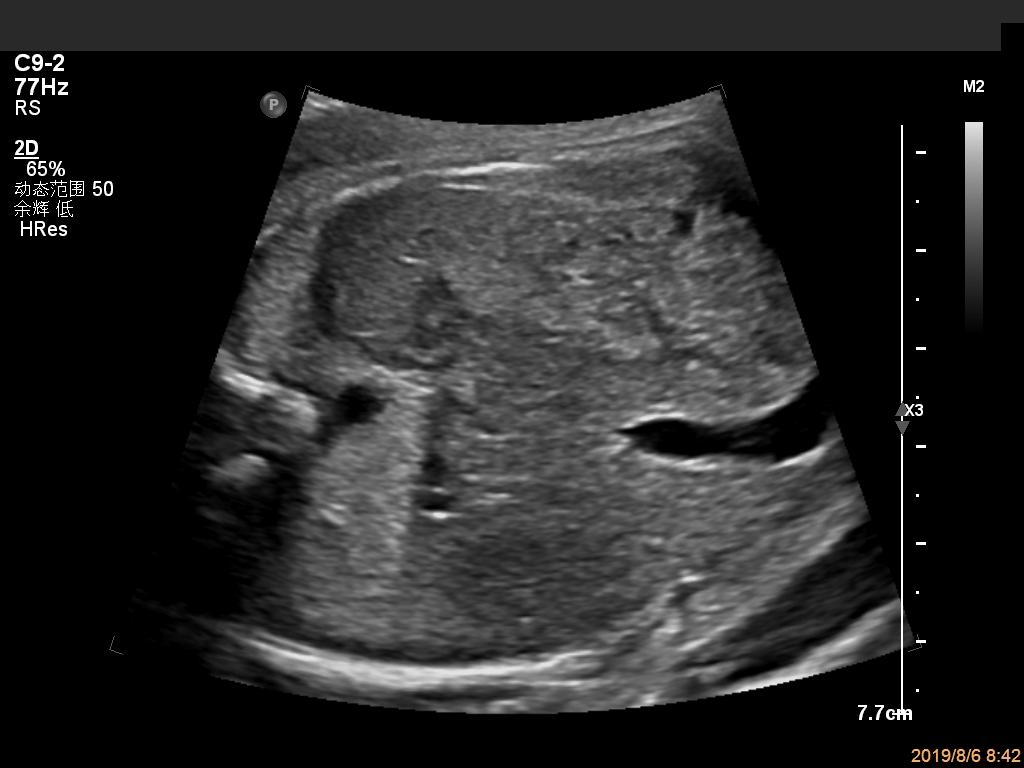

Supplement: S1 Dataset — (ZIP) [file pone.0305250.s001.zip › FE-SD-1/images/train_res/1014_ab.jpg]

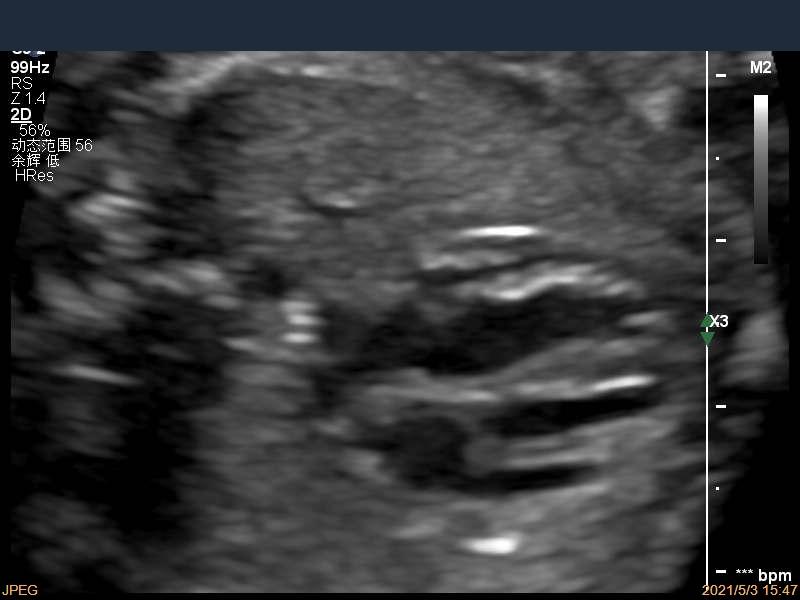

Supplement: S1 Dataset — (ZIP) [file pone.0305250.s001.zip › FE-SD-1/images/train_res/1014_fc.jpg]

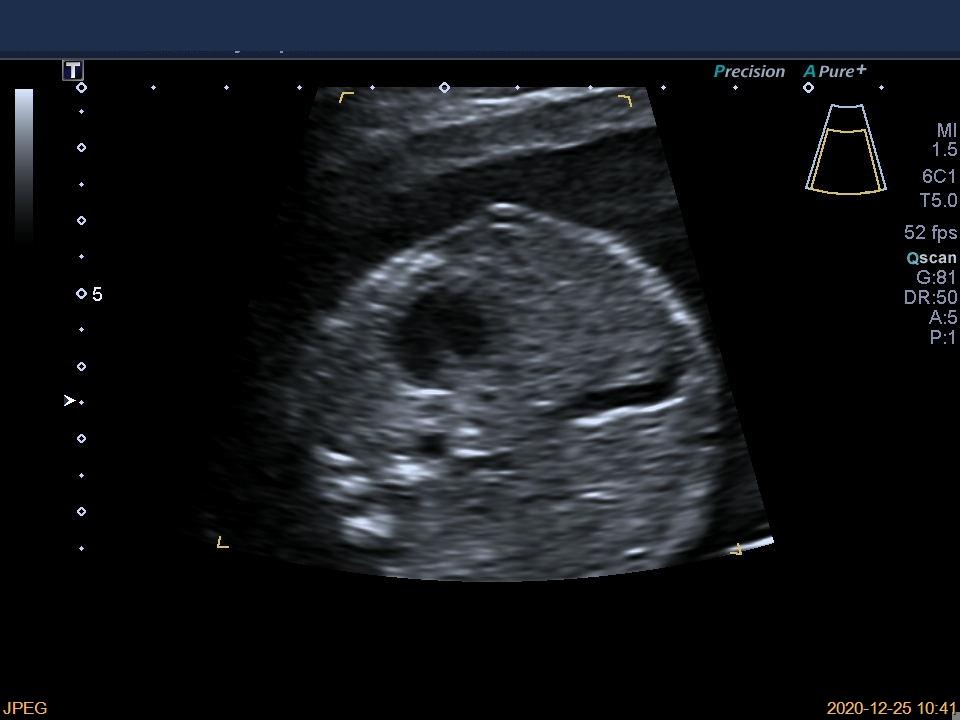

Supplement: S1 Dataset — (ZIP) [file pone.0305250.s001.zip › FE-SD-1/images/train_res/1016_ab.jpg]

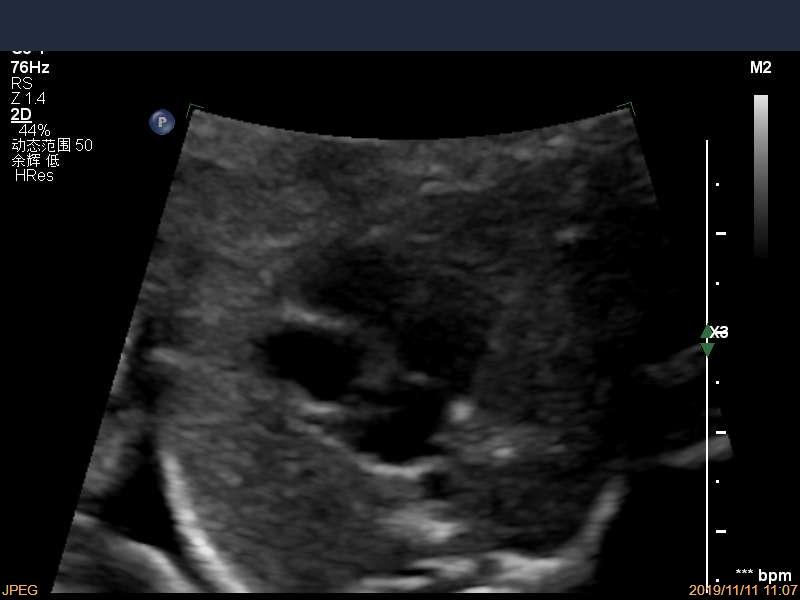

Supplement: S1 Dataset — (ZIP) [file pone.0305250.s001.zip › FE-SD-1/images/train_res/1017_fc.jpg]

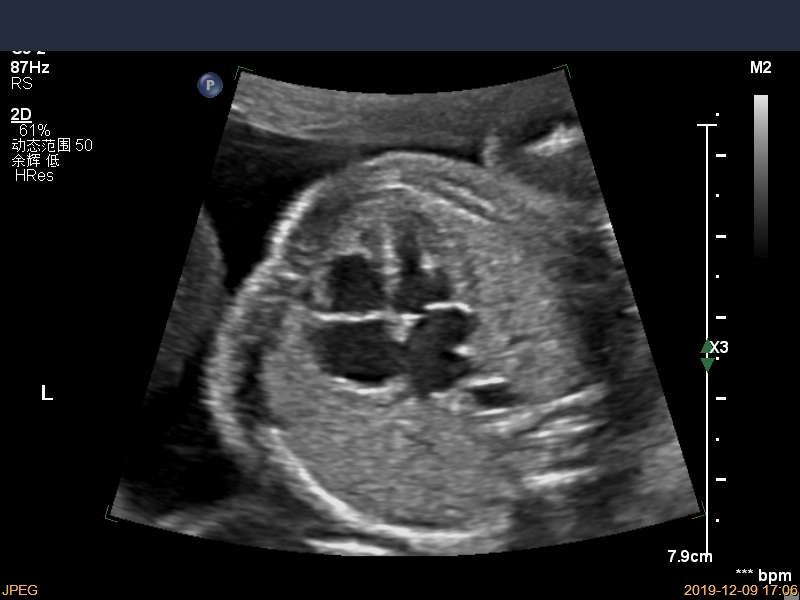

Supplement: S1 Dataset — (ZIP) [file pone.0305250.s001.zip › FE-SD-1/images/train_res/1018_fc.jpg]

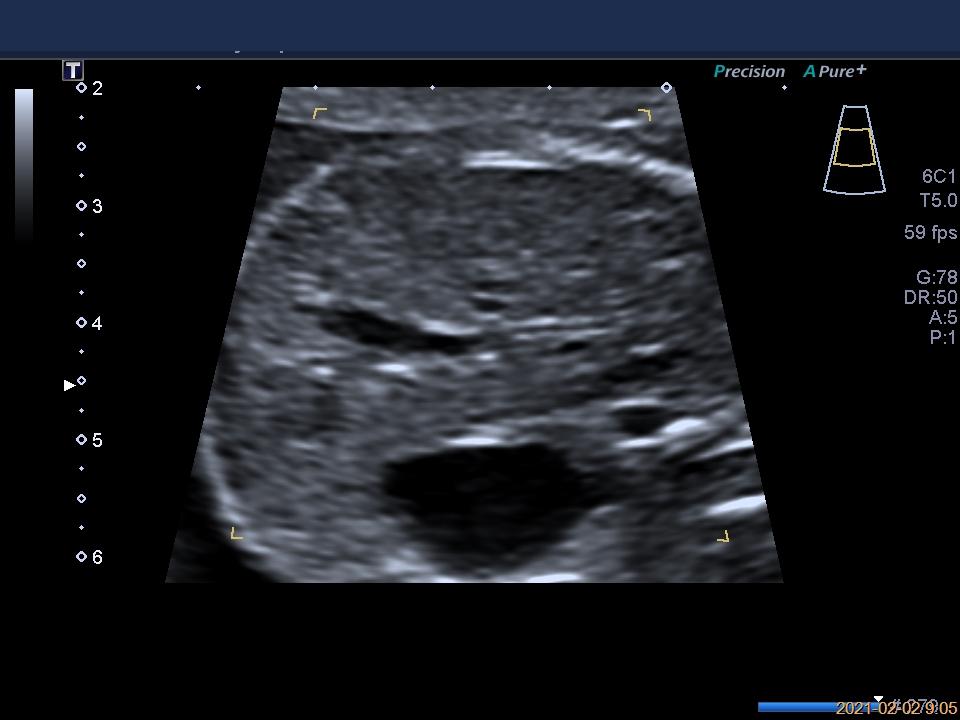

Supplement: S1 Dataset — (ZIP) [file pone.0305250.s001.zip › FE-SD-1/images/train_res/1019_ab.jpg]

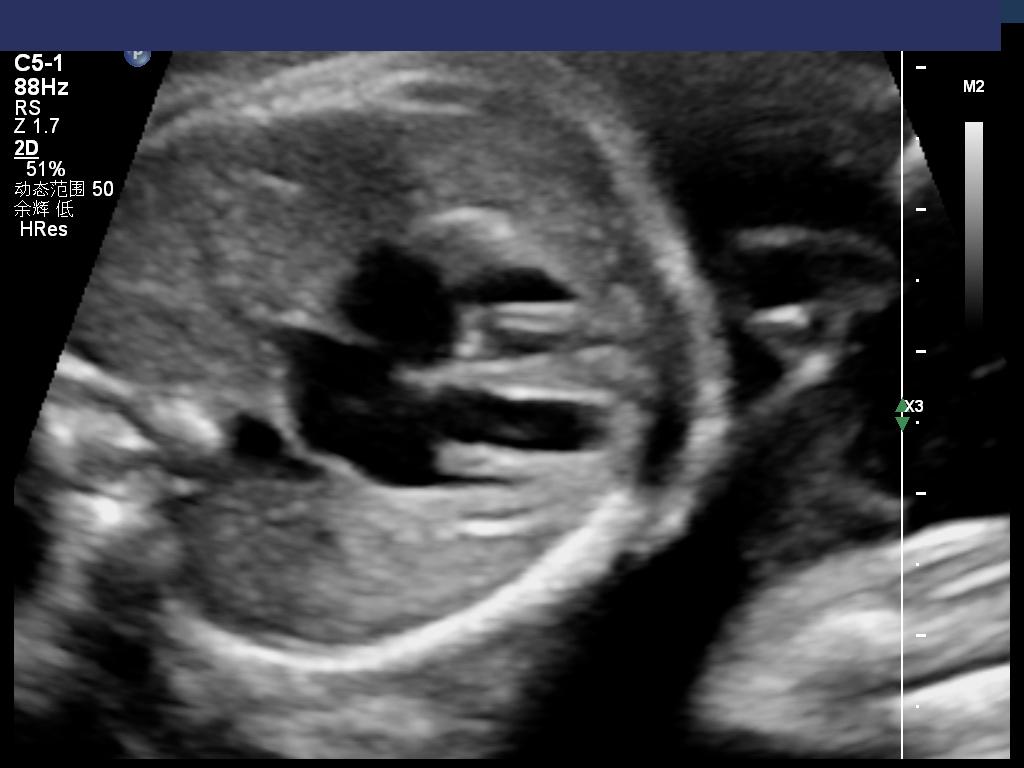

Supplement: S1 Dataset — (ZIP) [file pone.0305250.s001.zip › FE-SD-1/images/train_res/1019_fc.jpg]

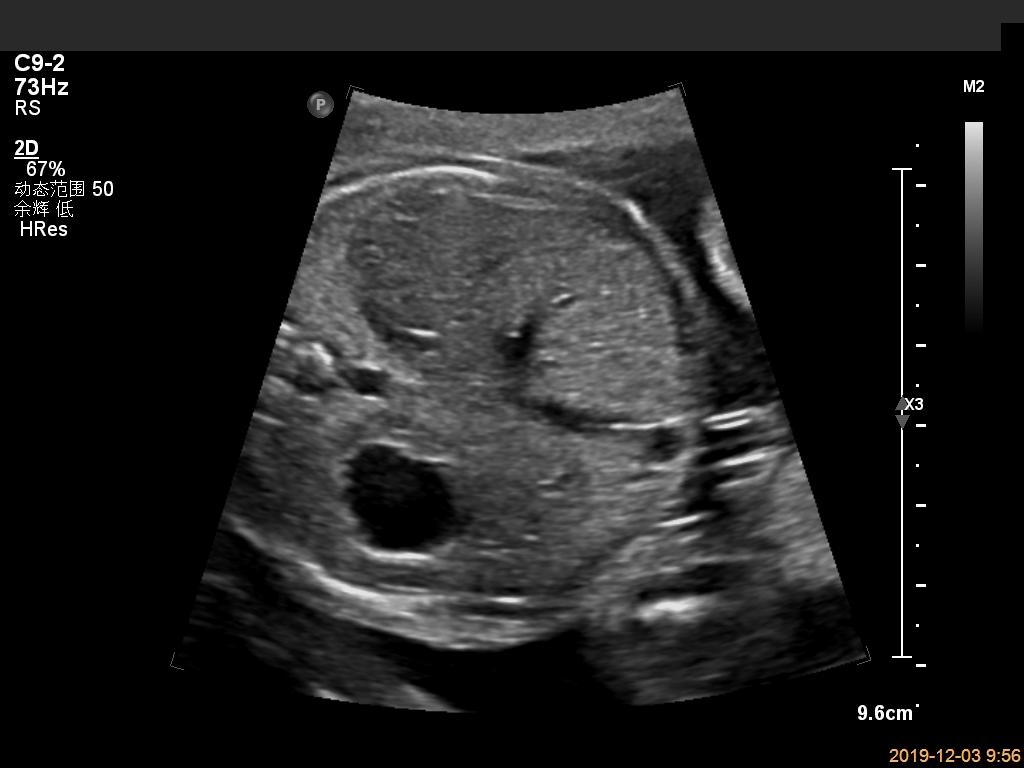

Supplement: S1 Dataset — (ZIP) [file pone.0305250.s001.zip › FE-SD-1/images/train_res/1020_ab.jpg]

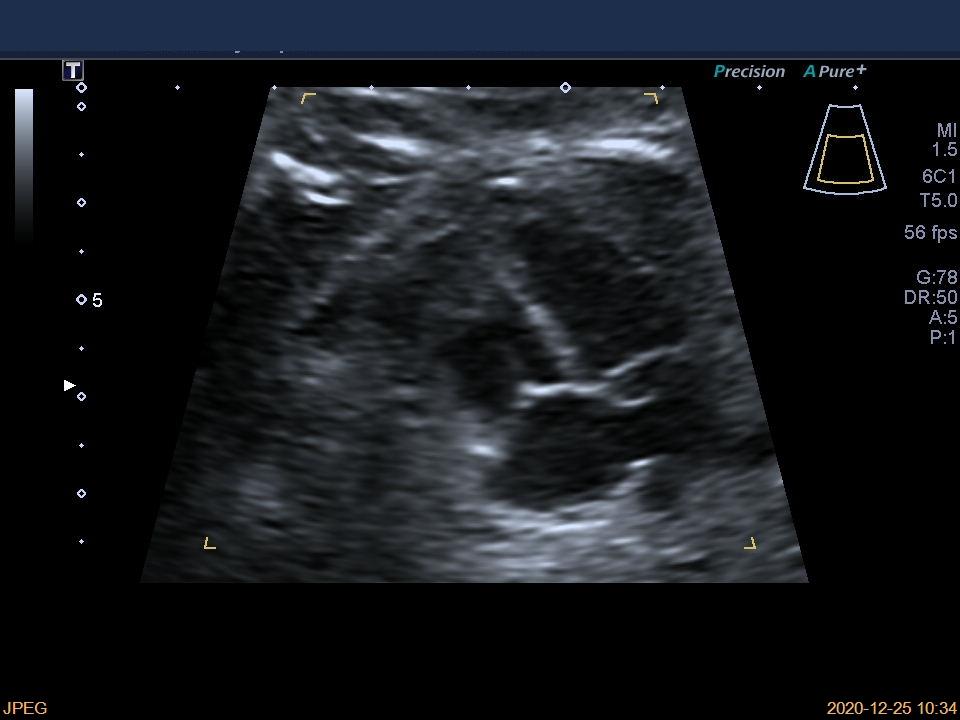

Supplement: S1 Dataset — (ZIP) [file pone.0305250.s001.zip › FE-SD-1/images/train_res/1020_fc.jpg]

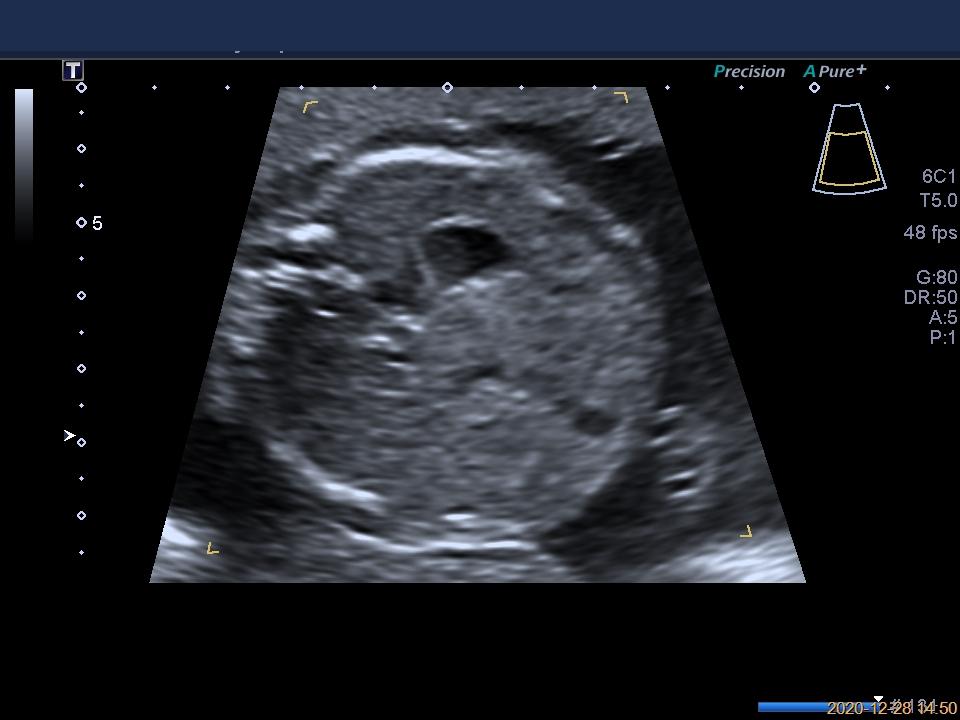

Supplement: S1 Dataset — (ZIP) [file pone.0305250.s001.zip › FE-SD-1/images/train_res/1021_ab.jpg]

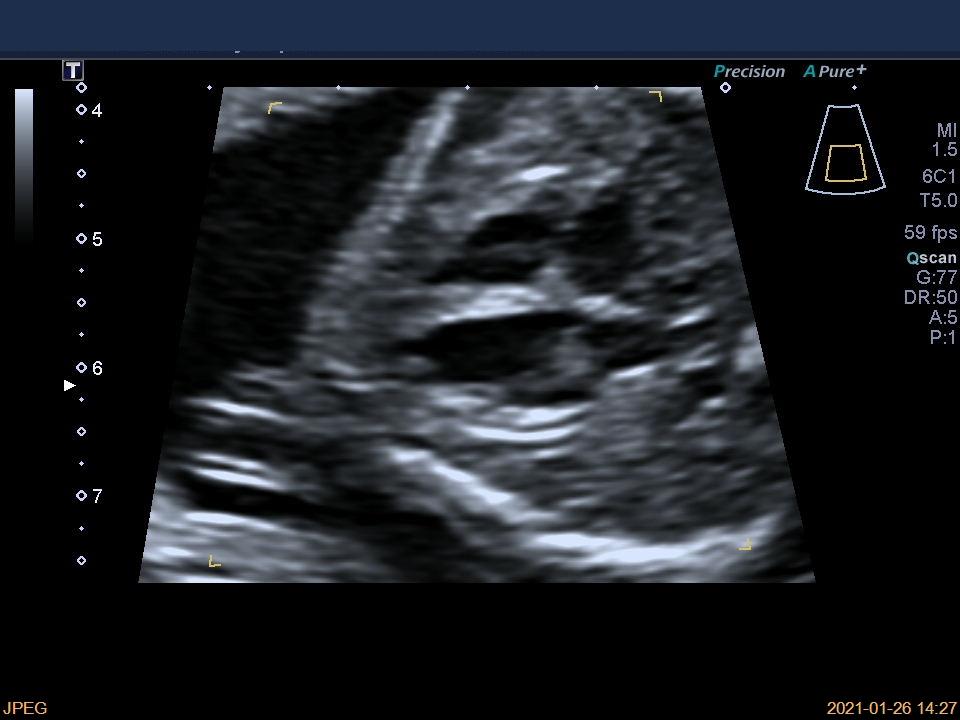

Supplement: S1 Dataset — (ZIP) [file pone.0305250.s001.zip › FE-SD-1/images/train_res/1021_fc.jpg]

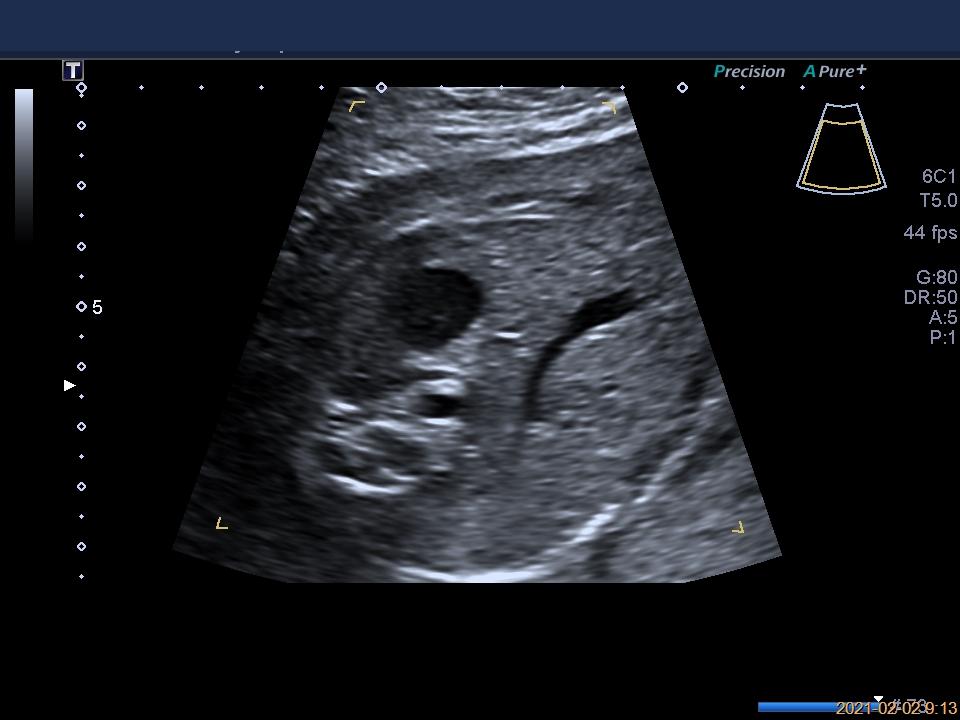

Supplement: S1 Dataset — (ZIP) [file pone.0305250.s001.zip › FE-SD-1/images/train_res/1022_ab.jpg]

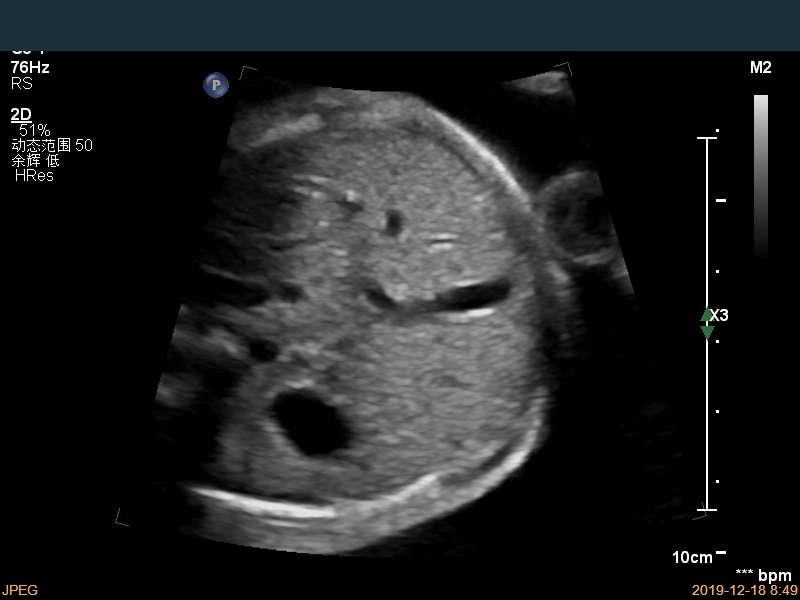

Supplement: S1 Dataset — (ZIP) [file pone.0305250.s001.zip › FE-SD-1/images/train_res/1023_ab.jpg]

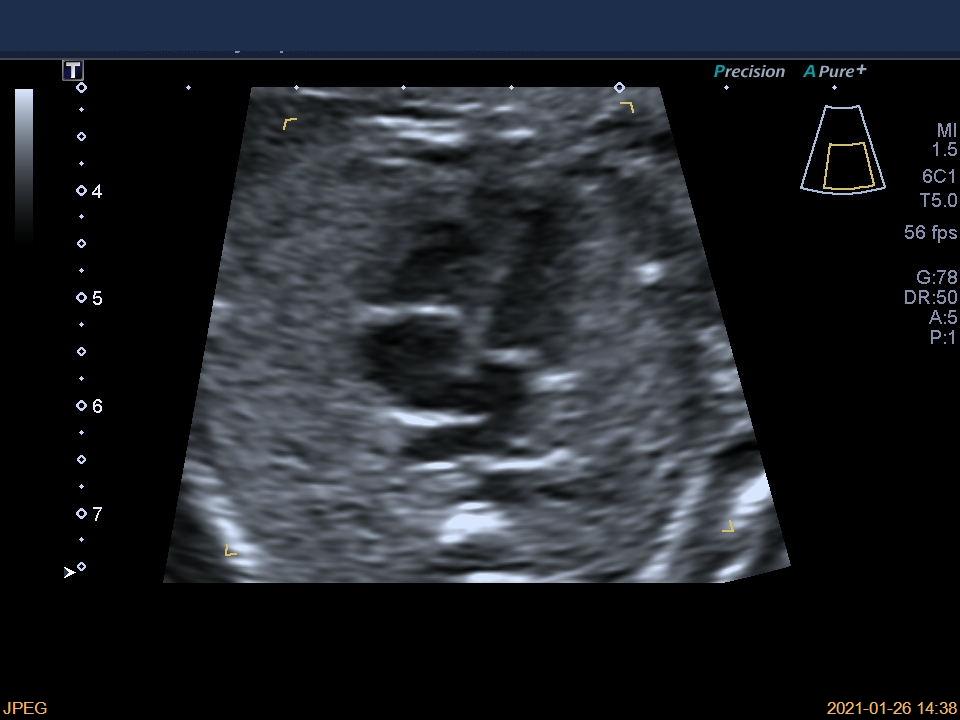

Supplement: S1 Dataset — (ZIP) [file pone.0305250.s001.zip › FE-SD-1/images/train_res/1023_fc.jpg]

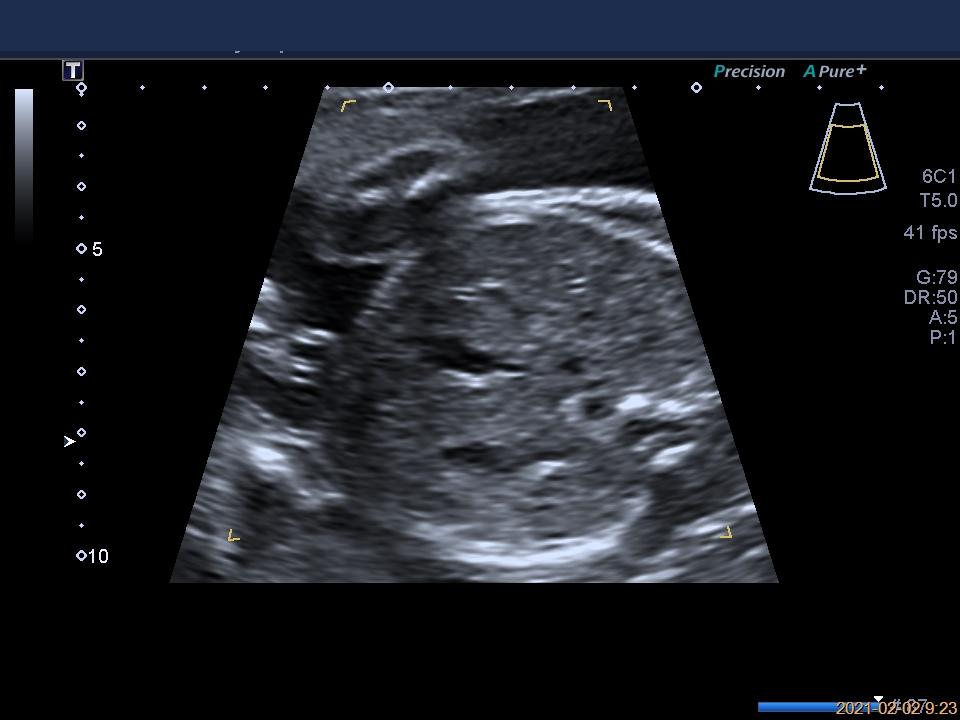

Supplement: S1 Dataset — (ZIP) [file pone.0305250.s001.zip › FE-SD-1/images/train_res/1024_ab.jpg]

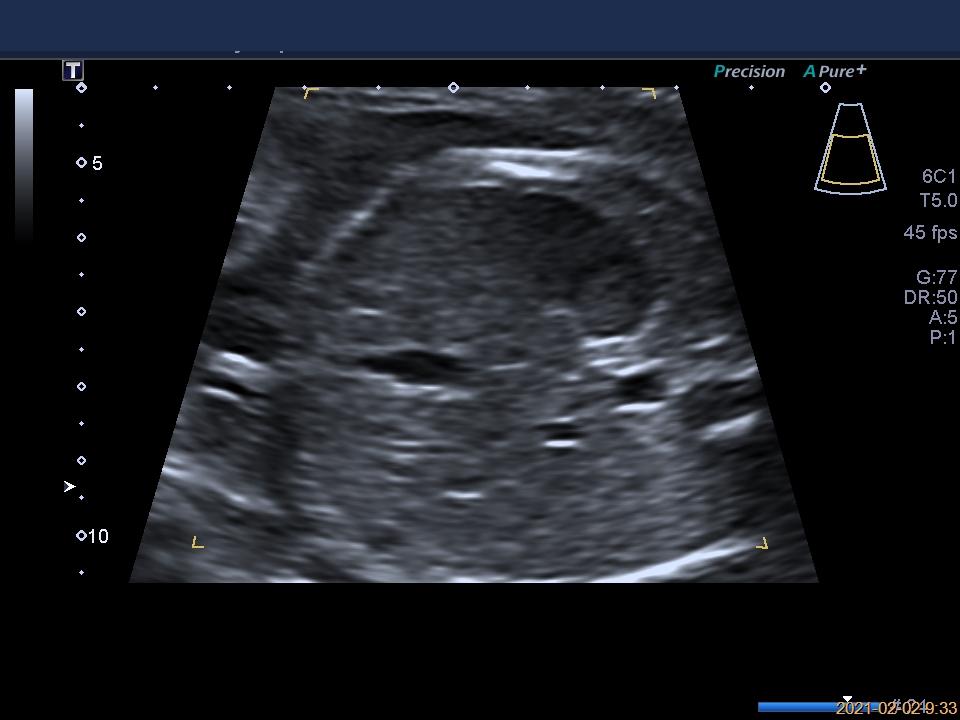

Supplement: S1 Dataset — (ZIP) [file pone.0305250.s001.zip › FE-SD-1/images/train_res/1025_ab.jpg]

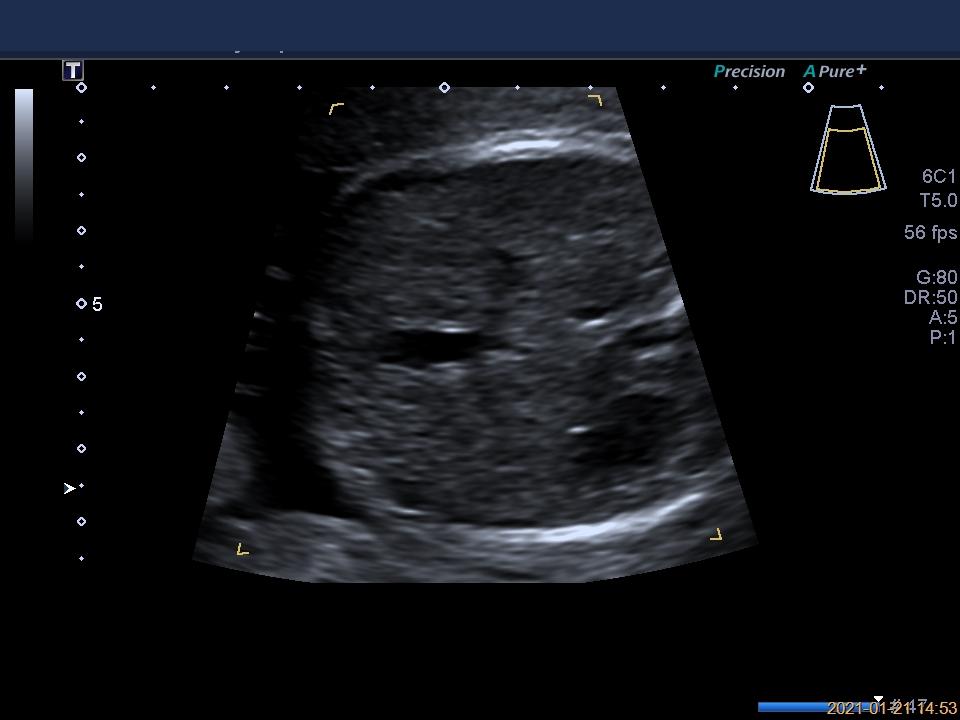

Supplement: S1 Dataset — (ZIP) [file pone.0305250.s001.zip › FE-SD-1/images/train_res/1026_ab.jpg]

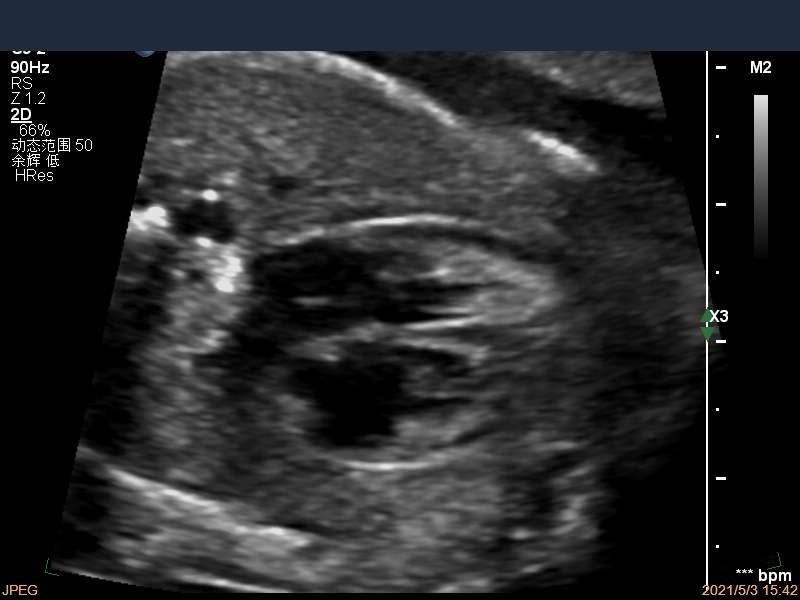

Supplement: S1 Dataset — (ZIP) [file pone.0305250.s001.zip › FE-SD-1/images/train_res/1026_fc.jpg]

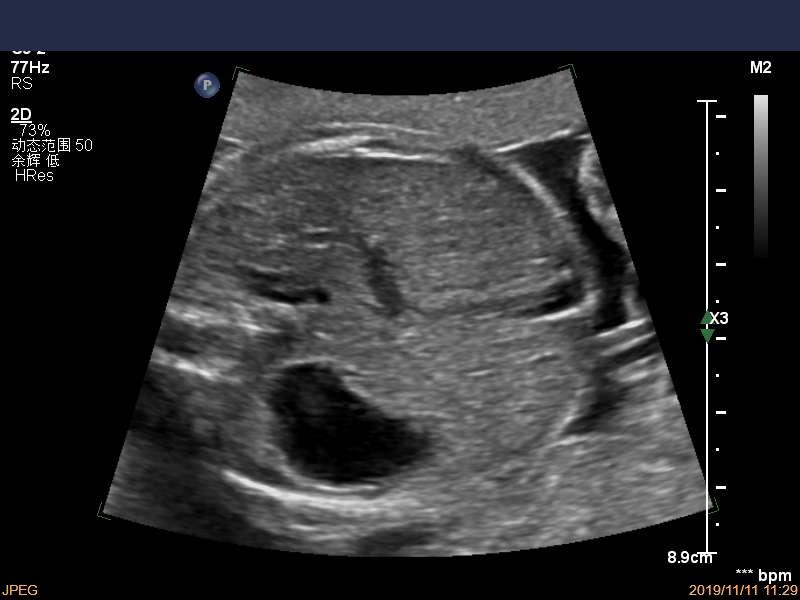

Supplement: S1 Dataset — (ZIP) [file pone.0305250.s001.zip › FE-SD-1/images/train_res/1027_ab.jpg]

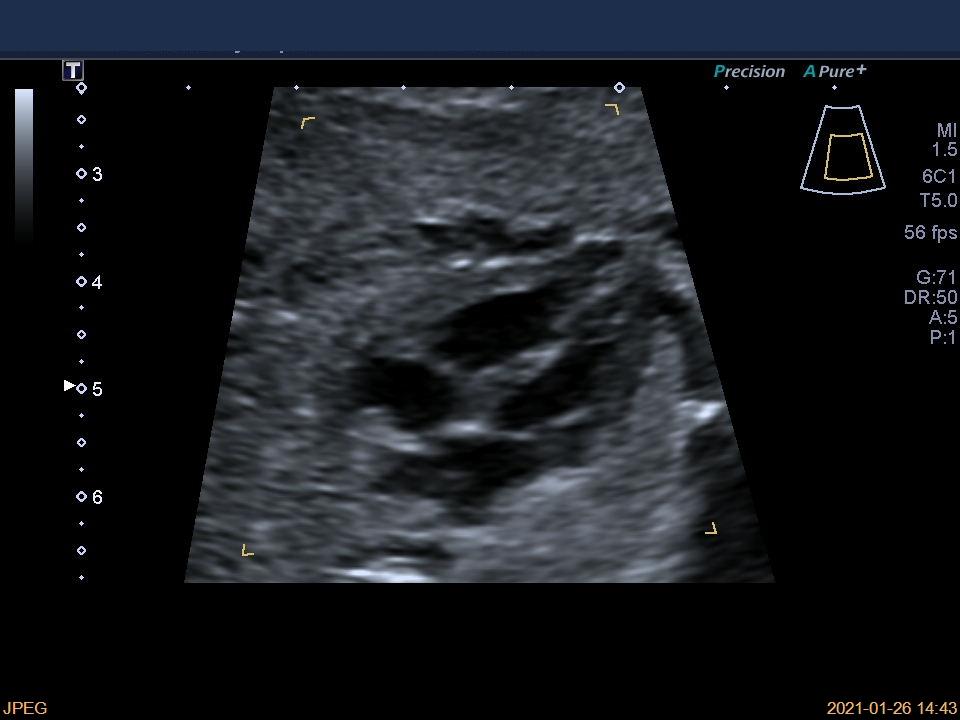

Supplement: S1 Dataset — (ZIP) [file pone.0305250.s001.zip › FE-SD-1/images/train_res/1027_fc.jpg]

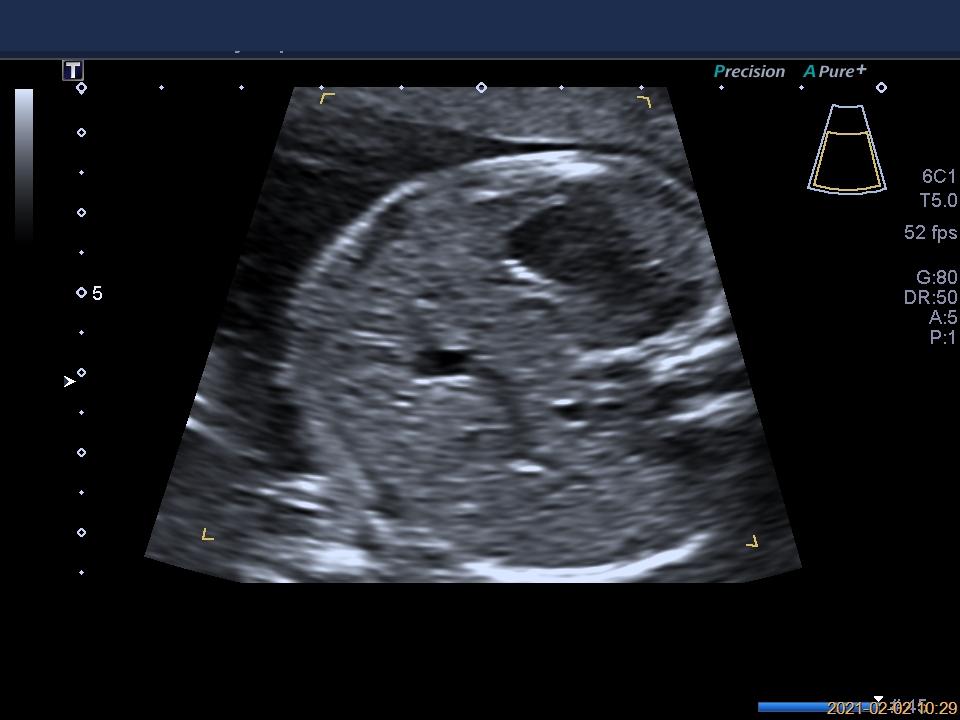

Supplement: S1 Dataset — (ZIP) [file pone.0305250.s001.zip › FE-SD-1/images/train_res/1028_ab.jpg]

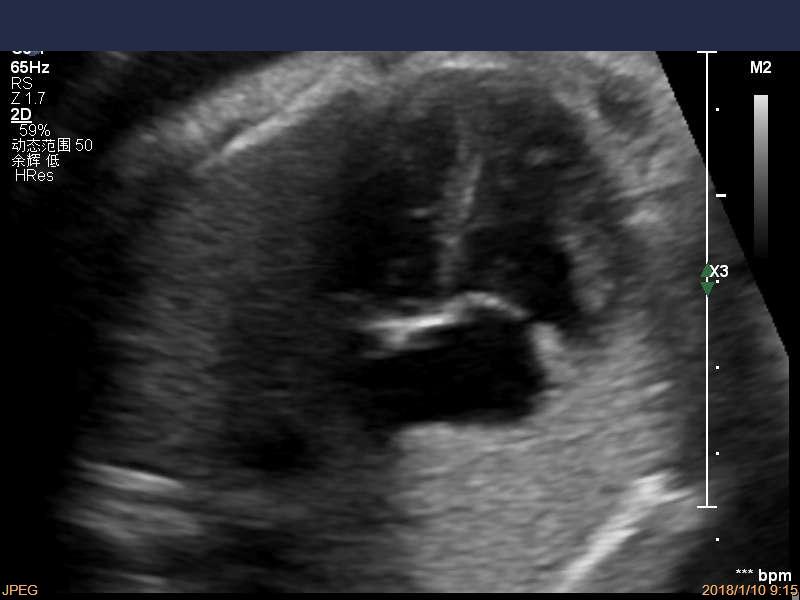

Supplement: S1 Dataset — (ZIP) [file pone.0305250.s001.zip › FE-SD-1/images/train_res/1028_fc.jpg]

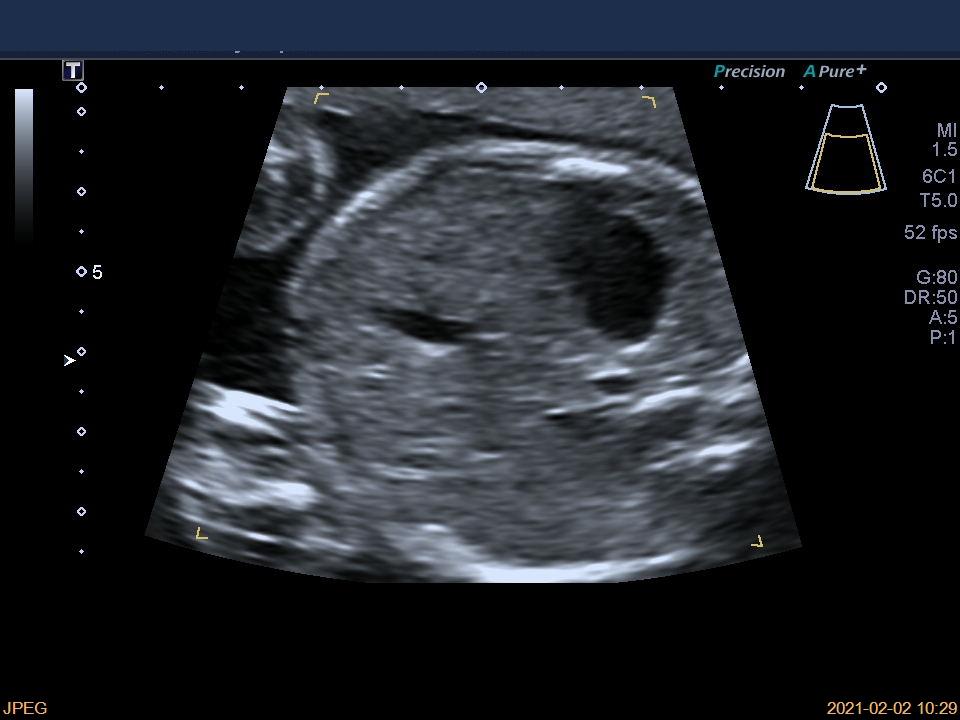

Supplement: S1 Dataset — (ZIP) [file pone.0305250.s001.zip › FE-SD-1/images/train_res/1029_ab.jpg]

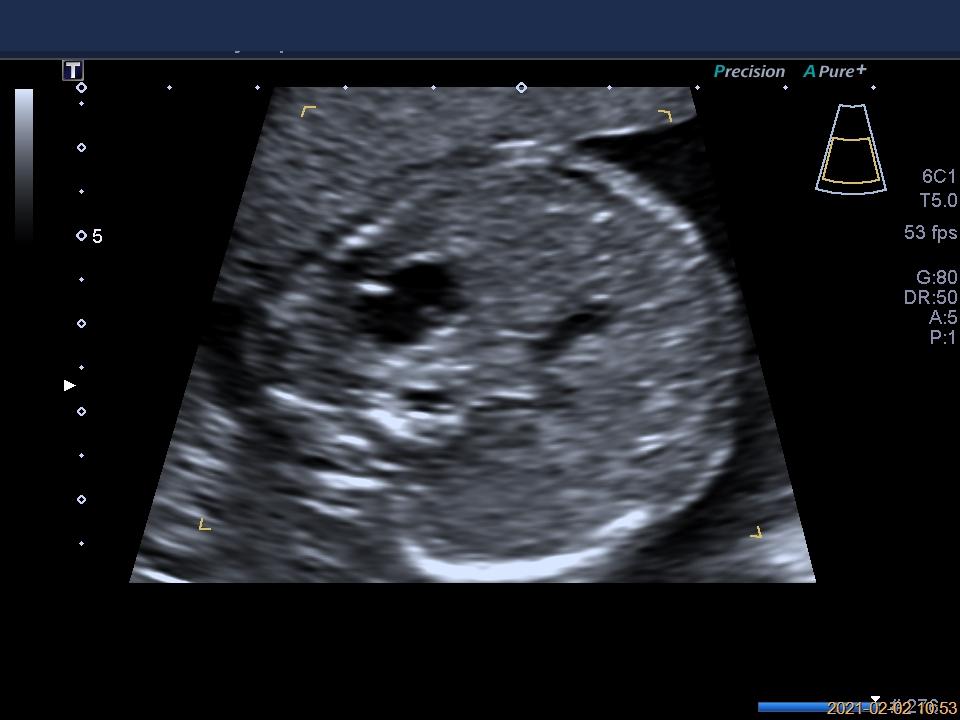

Supplement: S1 Dataset — (ZIP) [file pone.0305250.s001.zip › FE-SD-1/images/train_res/1030_ab.jpg]

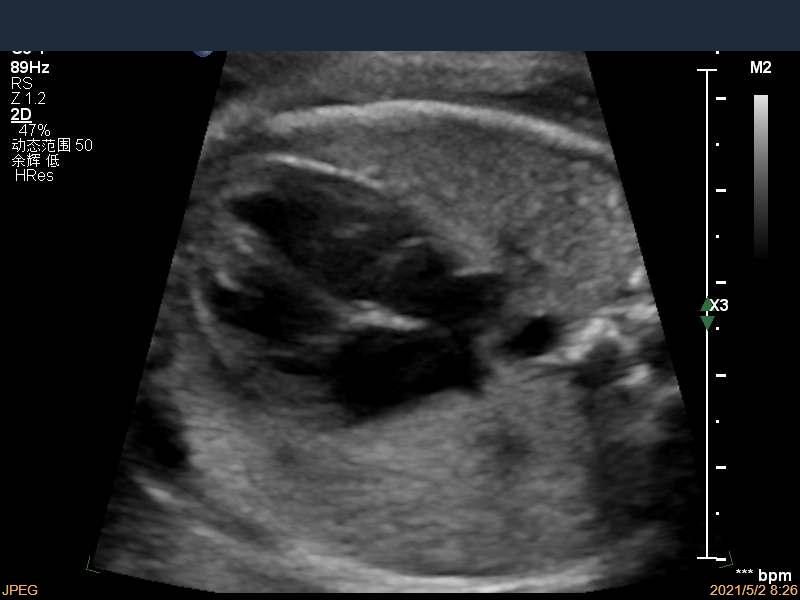

Supplement: S1 Dataset — (ZIP) [file pone.0305250.s001.zip › FE-SD-1/images/train_res/1030_fc.jpg]

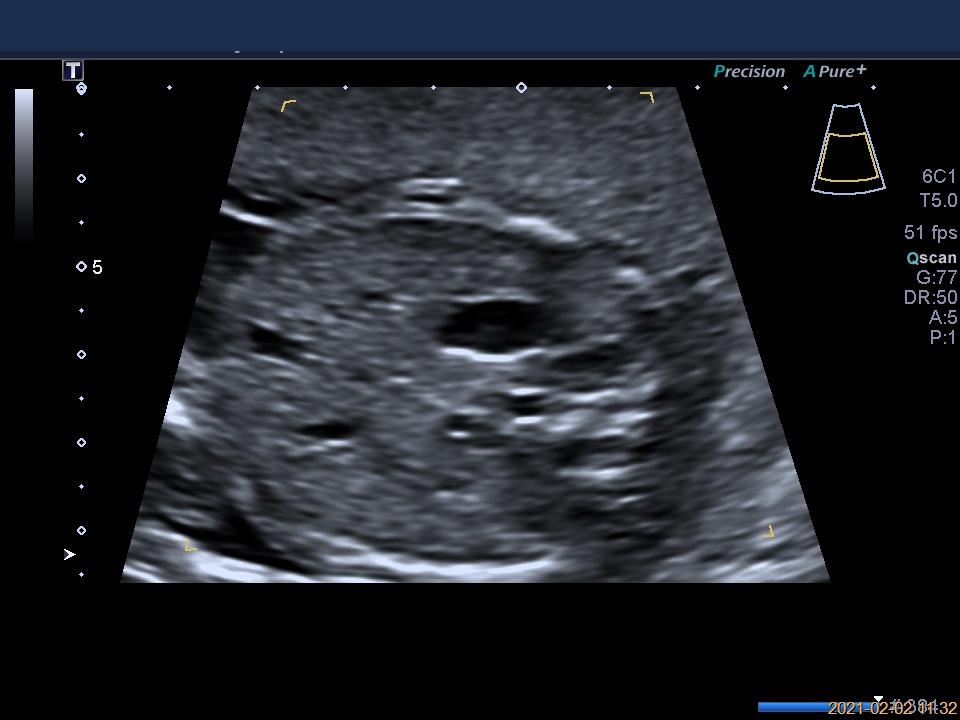

Supplement: S1 Dataset — (ZIP) [file pone.0305250.s001.zip › FE-SD-1/images/train_res/1031_ab.jpg]

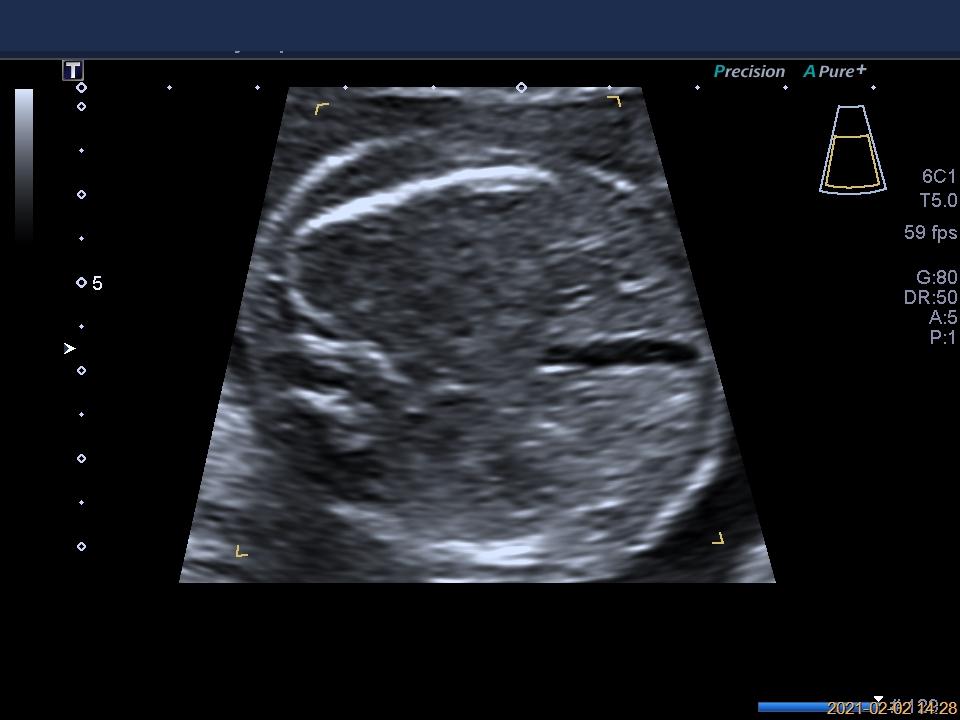

Supplement: S1 Dataset — (ZIP) [file pone.0305250.s001.zip › FE-SD-1/images/train_res/1032_ab.jpg]

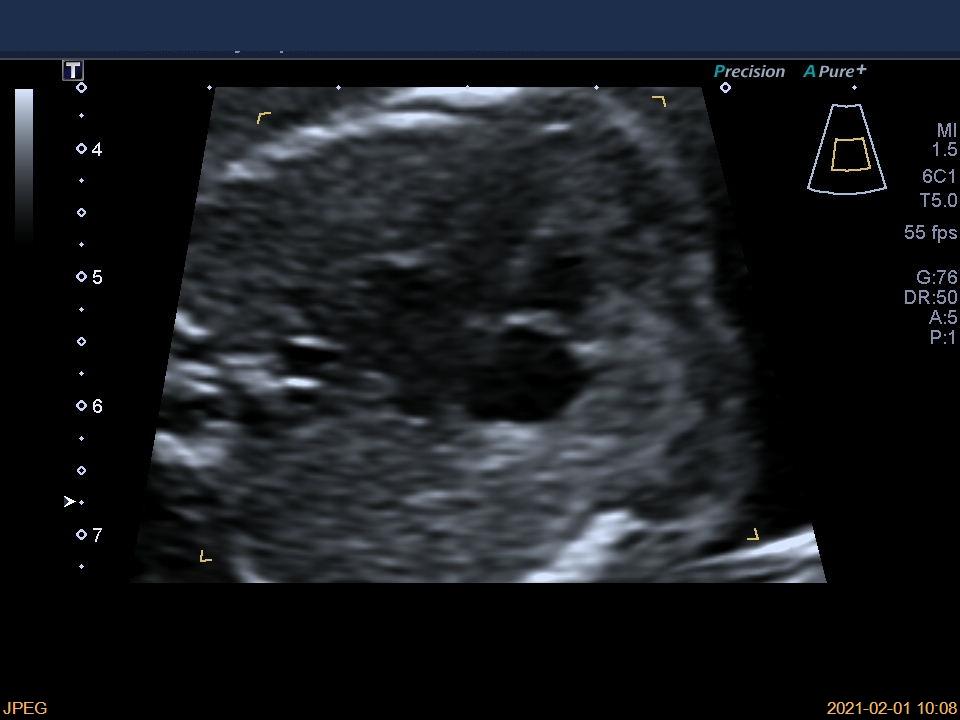

Supplement: S1 Dataset — (ZIP) [file pone.0305250.s001.zip › FE-SD-1/images/train_res/1032_fc.jpg]

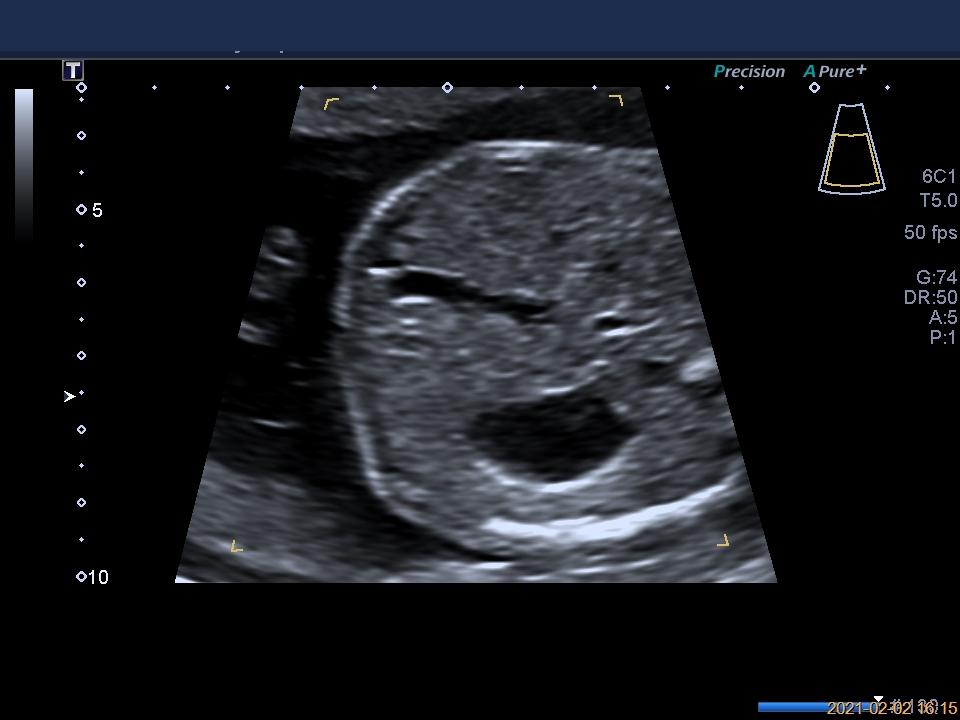

Supplement: S1 Dataset — (ZIP) [file pone.0305250.s001.zip › FE-SD-1/images/train_res/1033_ab.jpg]

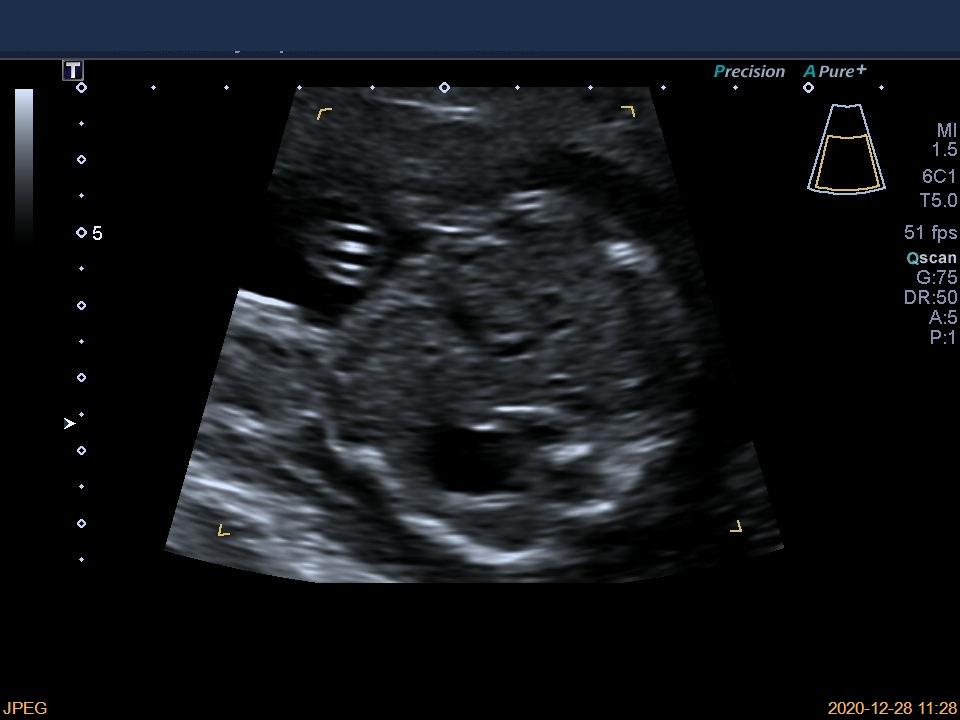

Supplement: S1 Dataset — (ZIP) [file pone.0305250.s001.zip › FE-SD-1/images/train_res/1034_ab.jpg]

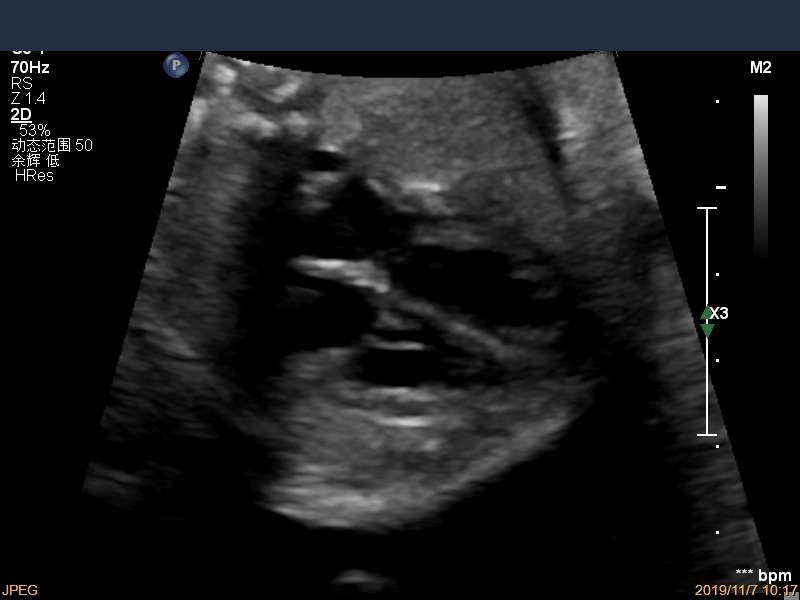

Supplement: S1 Dataset — (ZIP) [file pone.0305250.s001.zip › FE-SD-1/images/train_res/1034_fc.jpg]

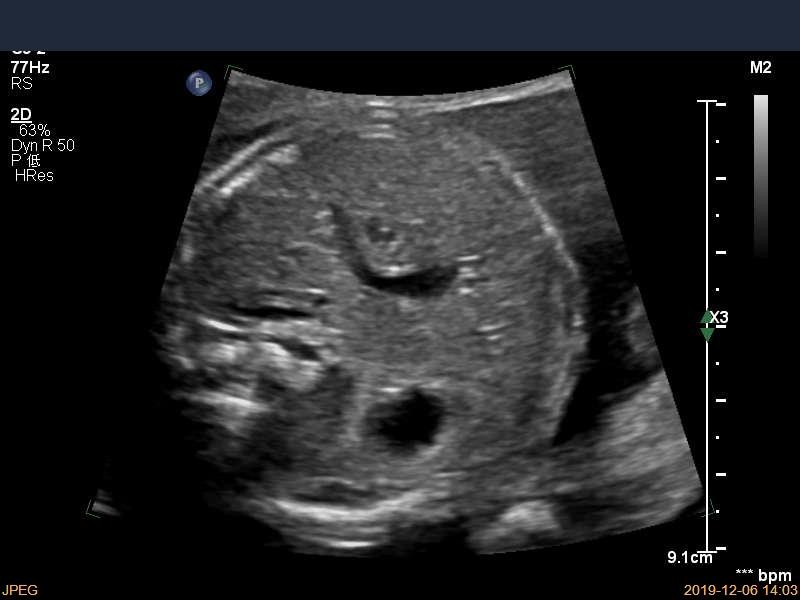

Supplement: S1 Dataset — (ZIP) [file pone.0305250.s001.zip › FE-SD-1/images/train_res/1035_ab.jpg]

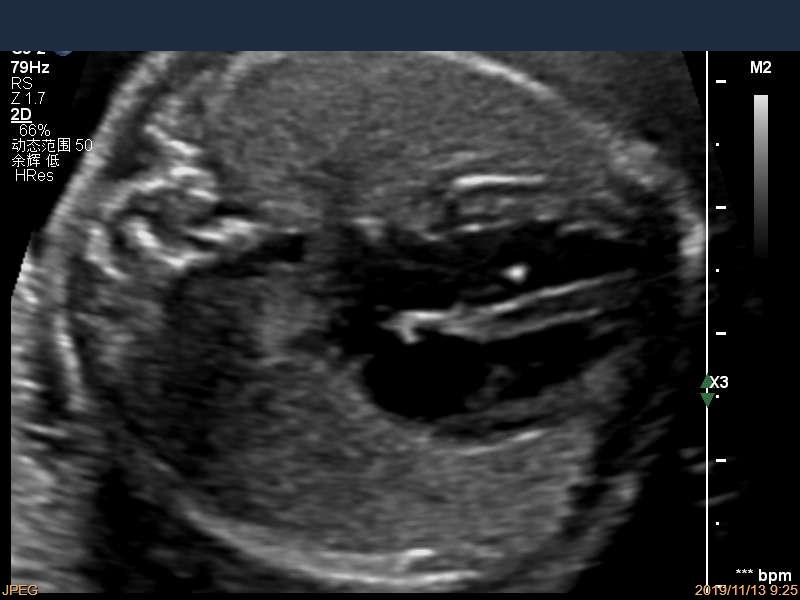

Supplement: S1 Dataset — (ZIP) [file pone.0305250.s001.zip › FE-SD-1/images/train_res/1035_fc.jpg]

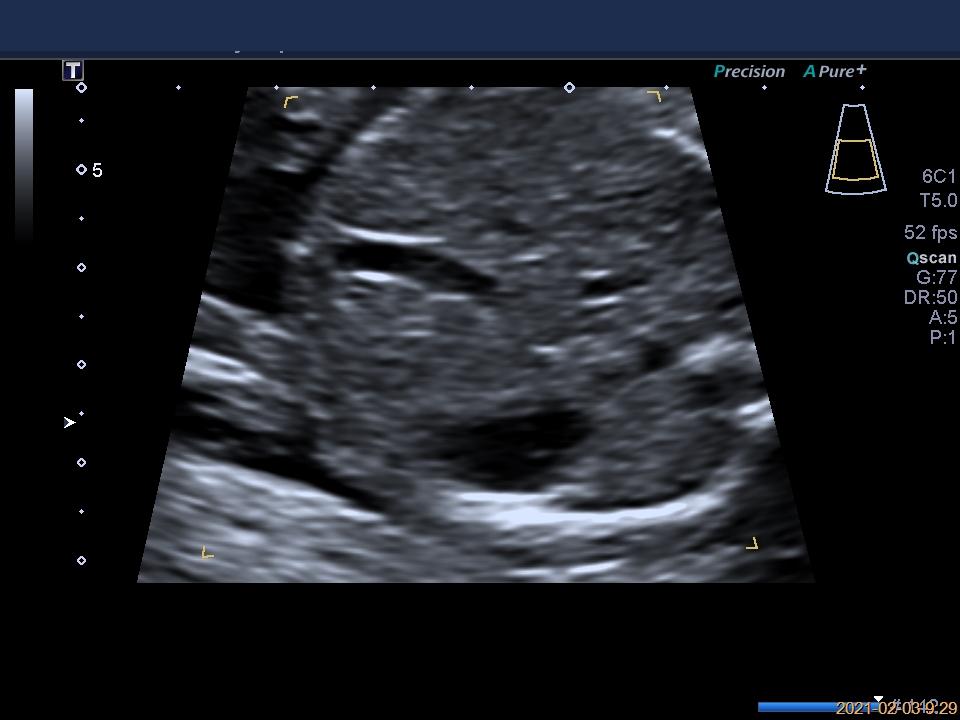

Supplement: S1 Dataset — (ZIP) [file pone.0305250.s001.zip › FE-SD-1/images/train_res/1037_ab.jpg]

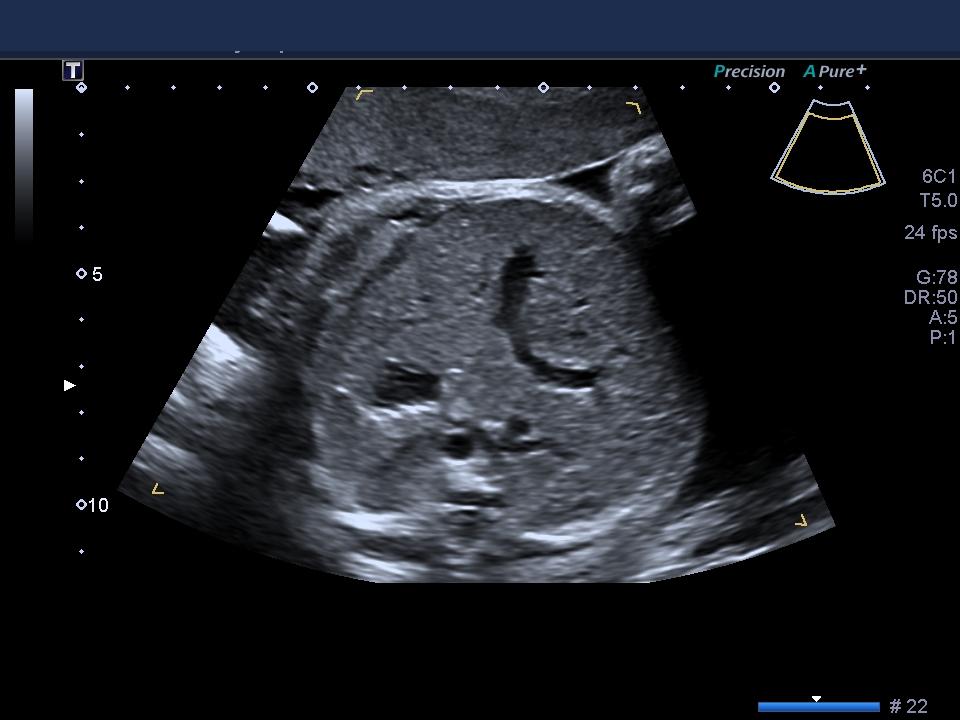

Supplement: S1 Dataset — (ZIP) [file pone.0305250.s001.zip › FE-SD-1/images/train_res/1038_ab.jpg]

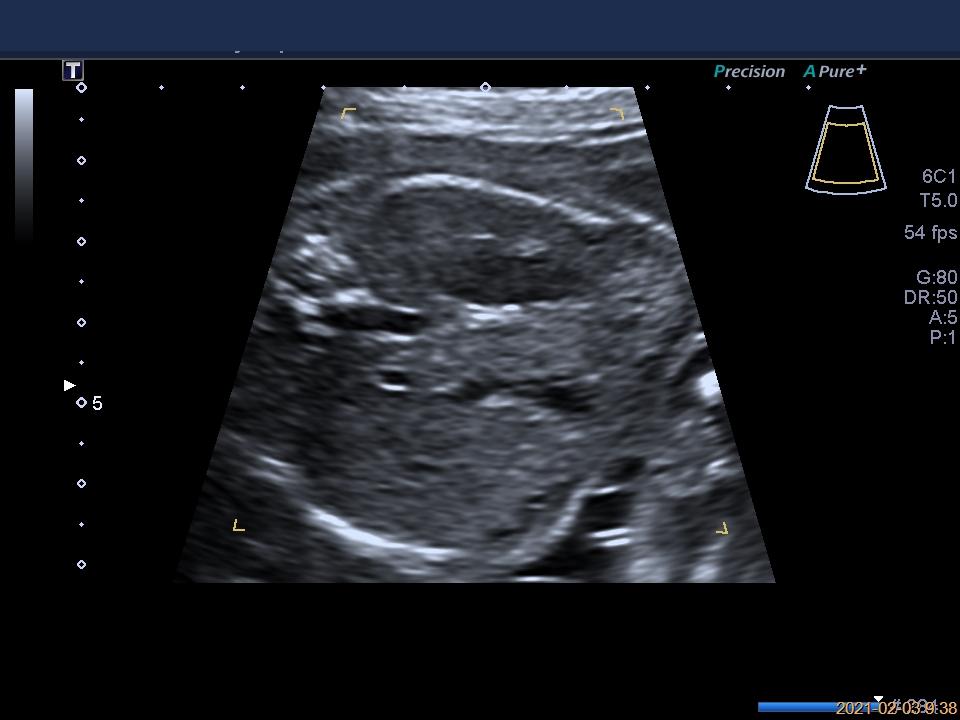

Supplement: S1 Dataset — (ZIP) [file pone.0305250.s001.zip › FE-SD-1/images/train_res/1039_ab.jpg]

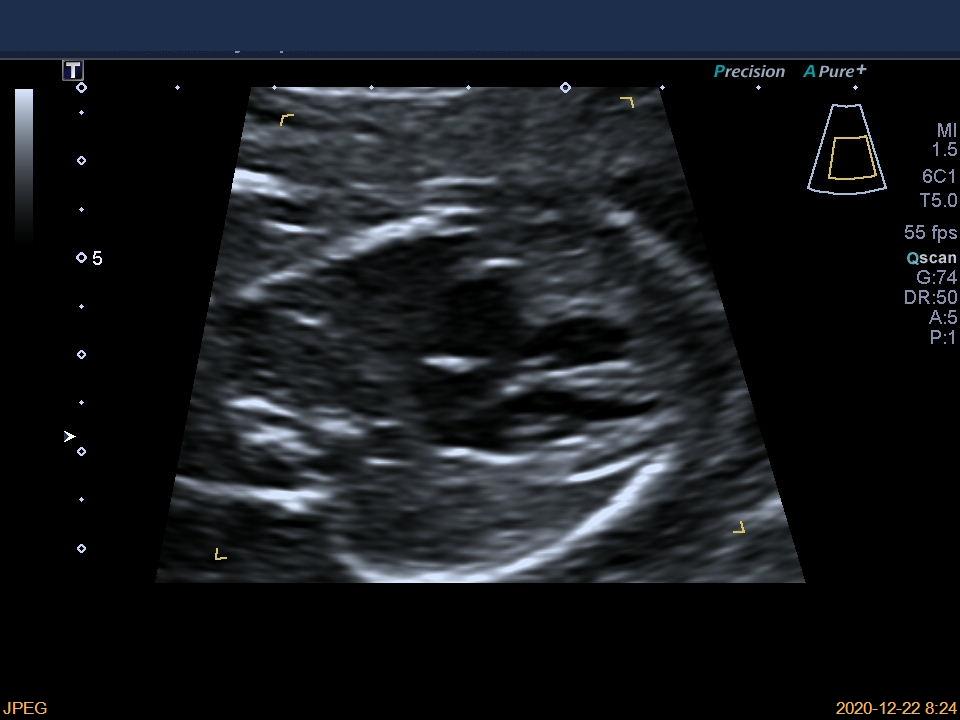

Supplement: S1 Dataset — (ZIP) [file pone.0305250.s001.zip › FE-SD-1/images/train_res/1039_fc.jpg]

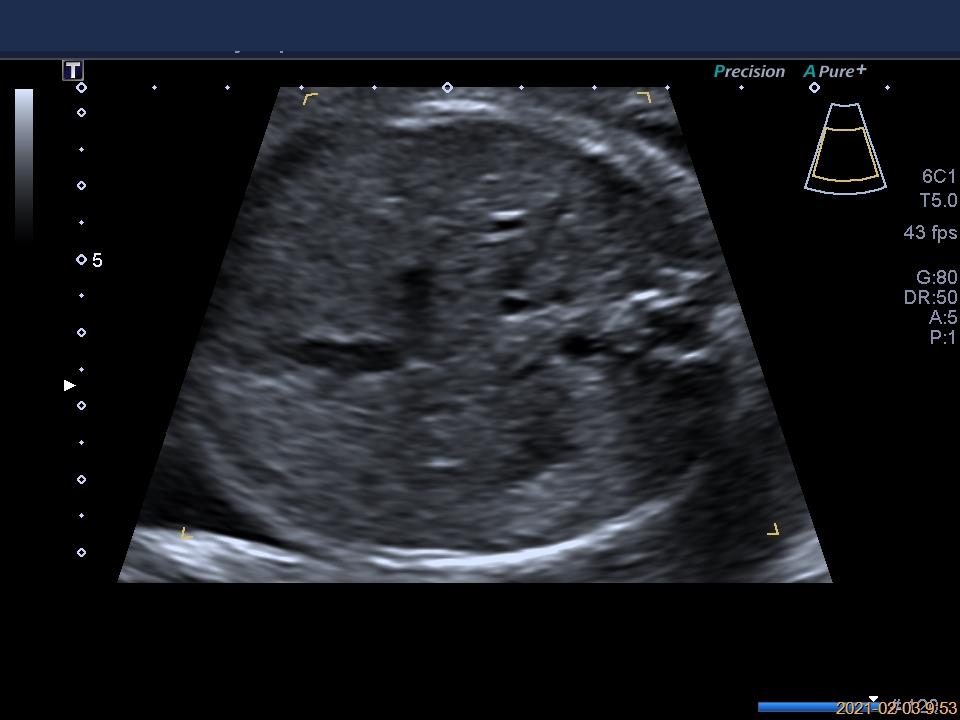

Supplement: S1 Dataset — (ZIP) [file pone.0305250.s001.zip › FE-SD-1/images/train_res/1041_ab.jpg]

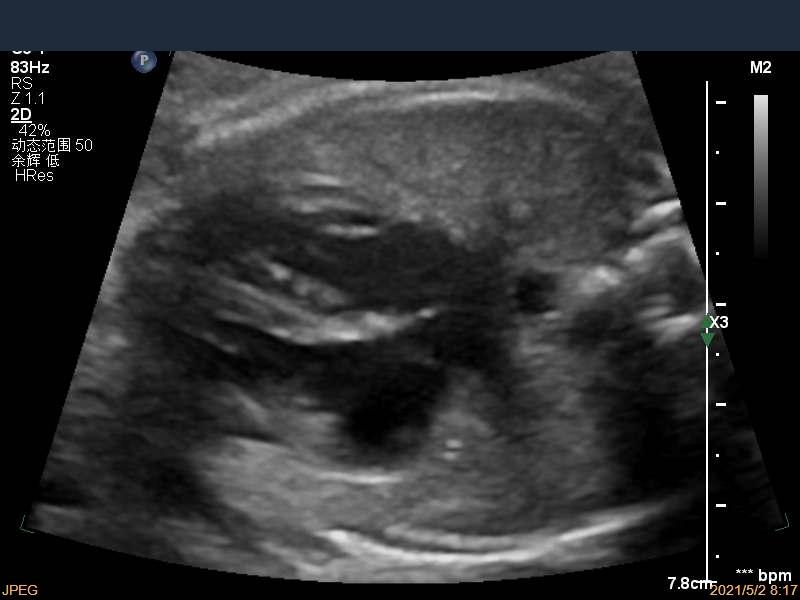

Supplement: S1 Dataset — (ZIP) [file pone.0305250.s001.zip › FE-SD-1/images/train_res/1041_fc.jpg]

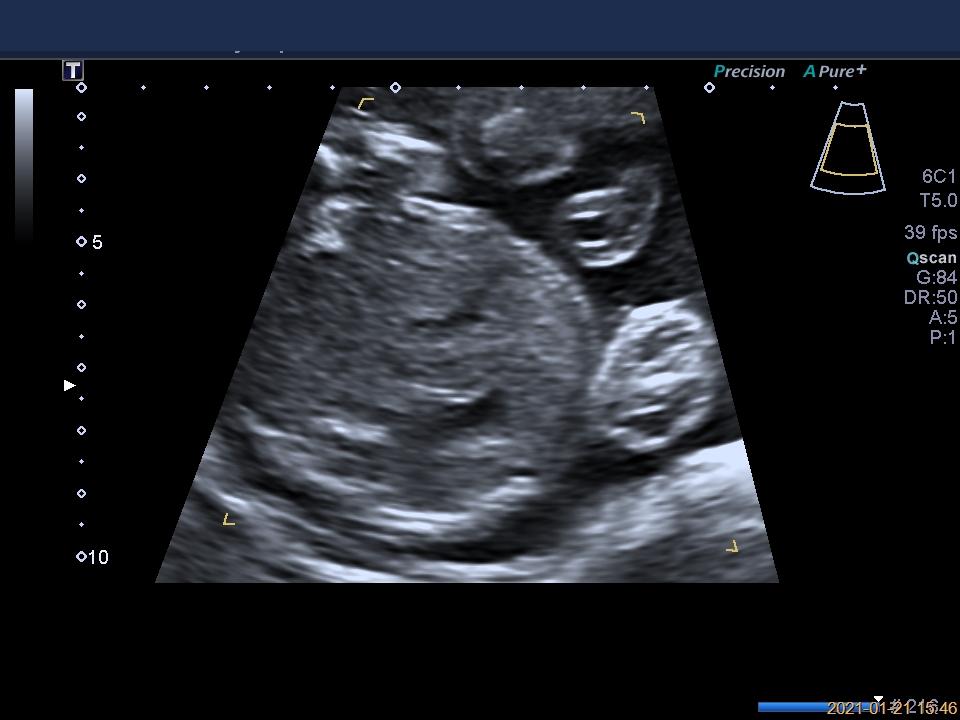

Supplement: S1 Dataset — (ZIP) [file pone.0305250.s001.zip › FE-SD-1/images/train_res/1042_ab.jpg]

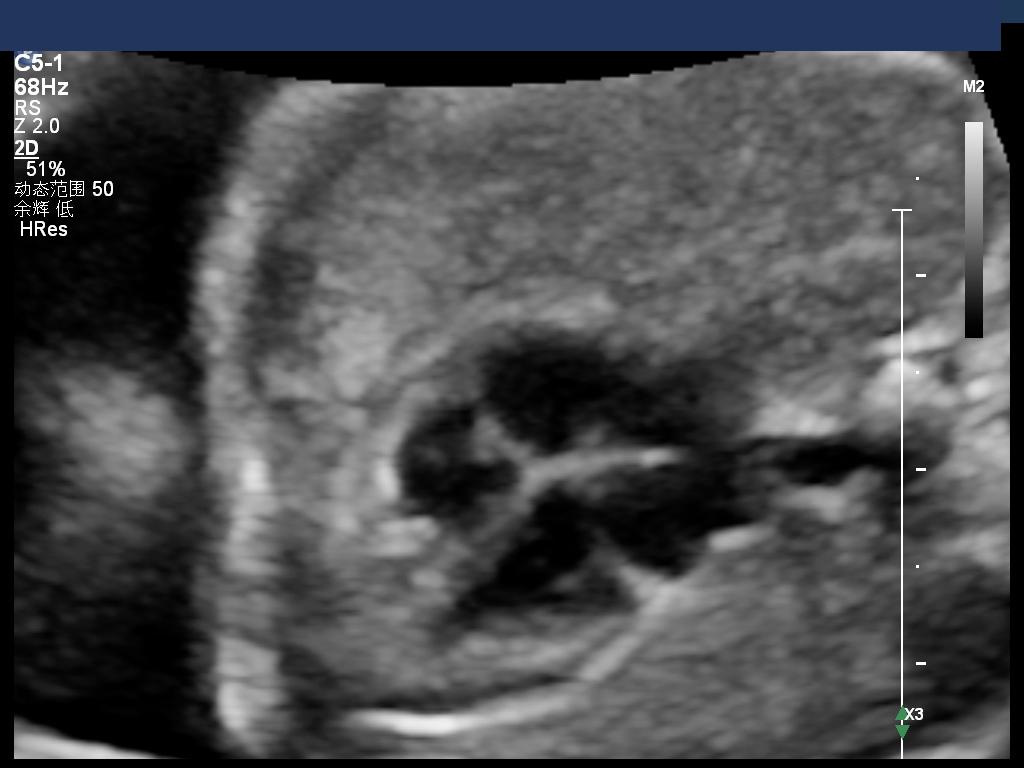

Supplement: S1 Dataset — (ZIP) [file pone.0305250.s001.zip › FE-SD-1/images/train_res/1042_fc.jpg]

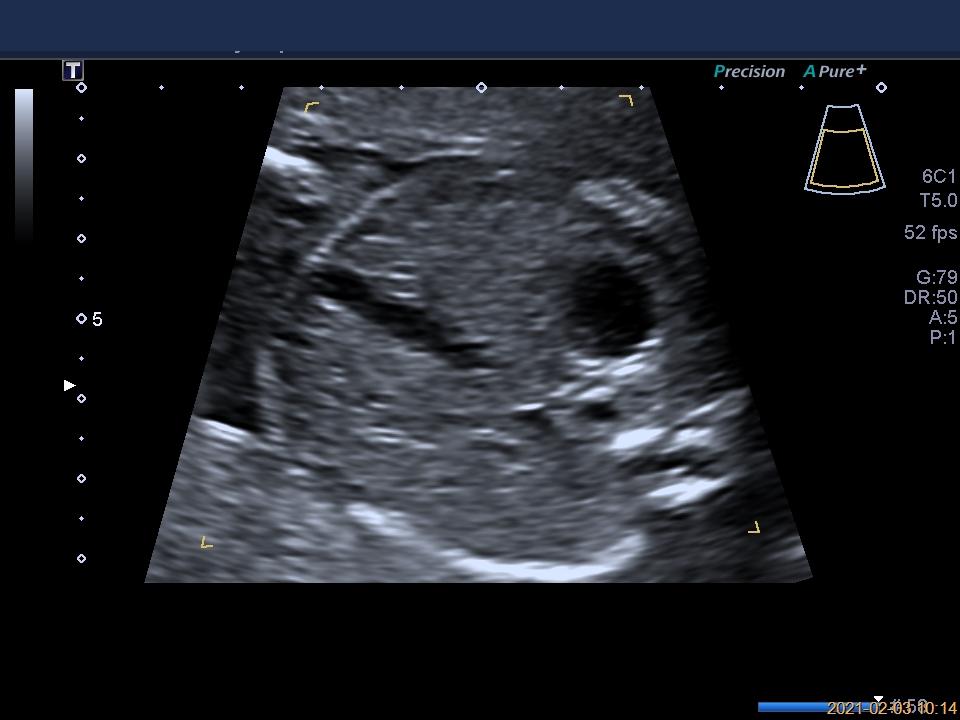

Supplement: S1 Dataset — (ZIP) [file pone.0305250.s001.zip › FE-SD-1/images/train_res/1044_ab.jpg]

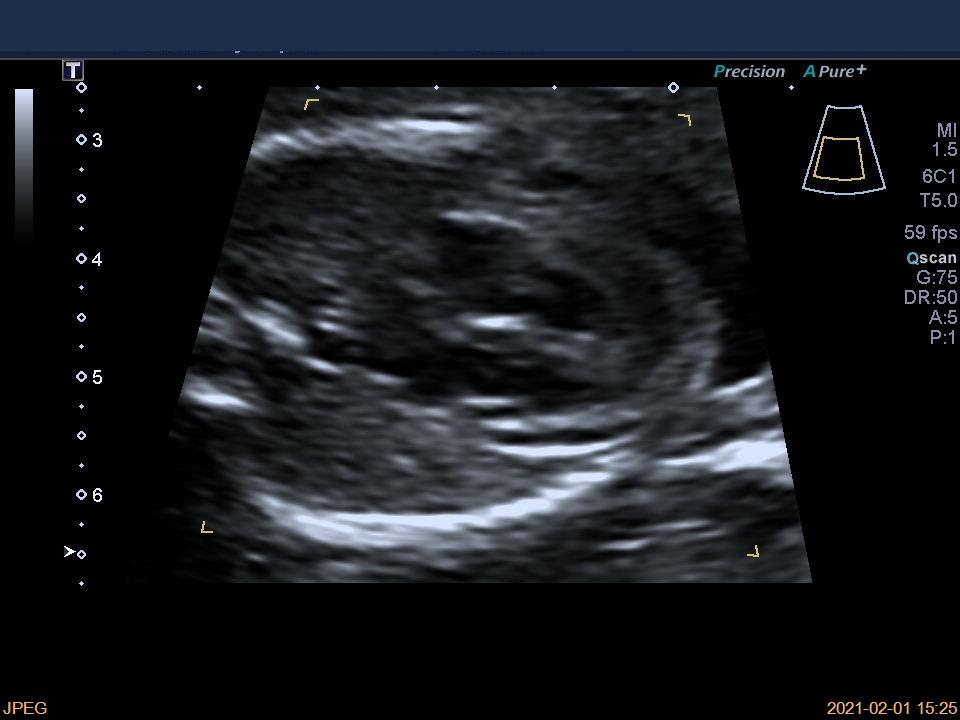

Supplement: S1 Dataset — (ZIP) [file pone.0305250.s001.zip › FE-SD-1/images/train_res/1045_fc.jpg]

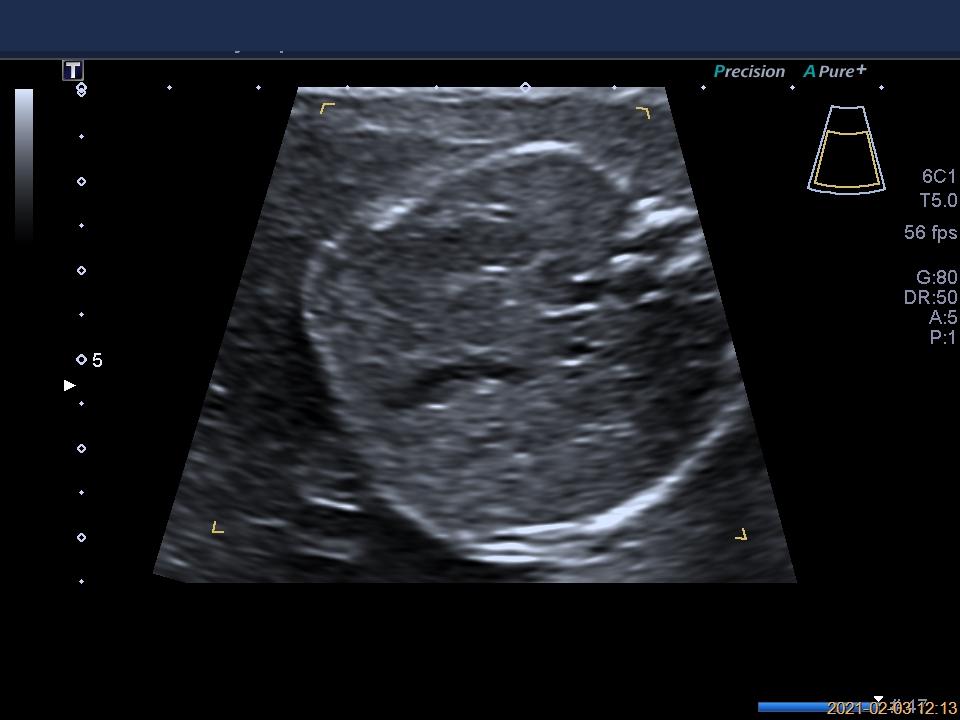

Supplement: S1 Dataset — (ZIP) [file pone.0305250.s001.zip › FE-SD-1/images/train_res/1046_ab.jpg]

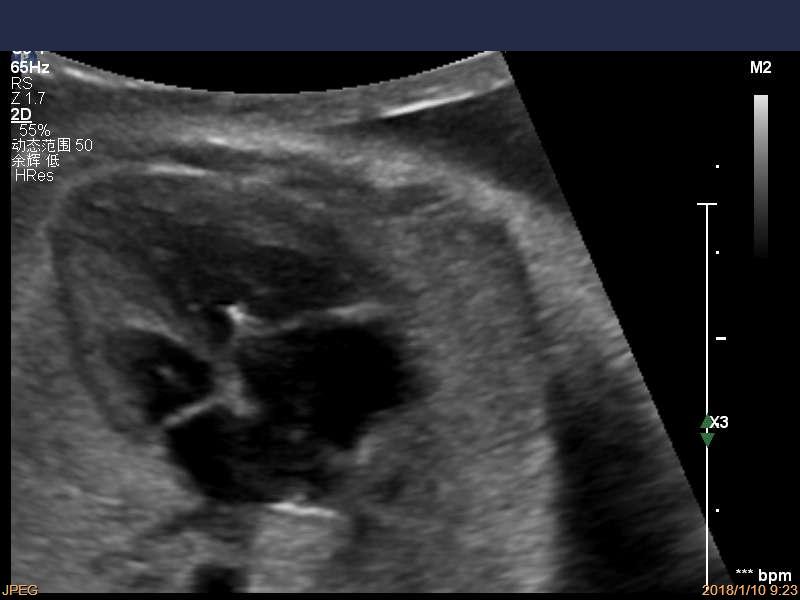

Supplement: S1 Dataset — (ZIP) [file pone.0305250.s001.zip › FE-SD-1/images/train_res/1046_fc.jpg]

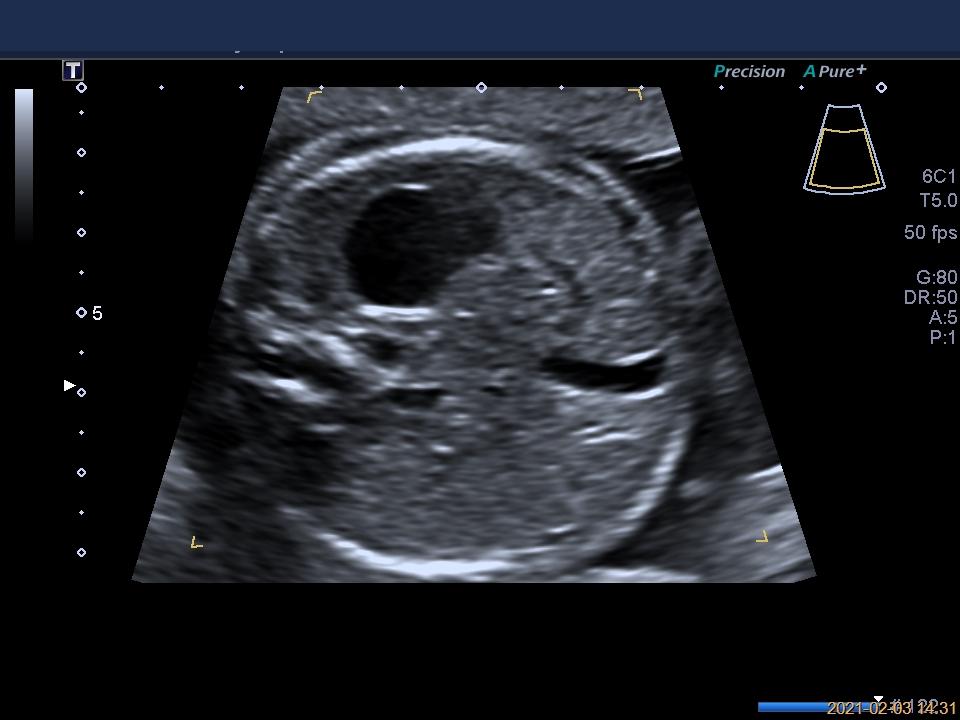

Supplement: S1 Dataset — (ZIP) [file pone.0305250.s001.zip › FE-SD-1/images/train_res/1047_ab.jpg]

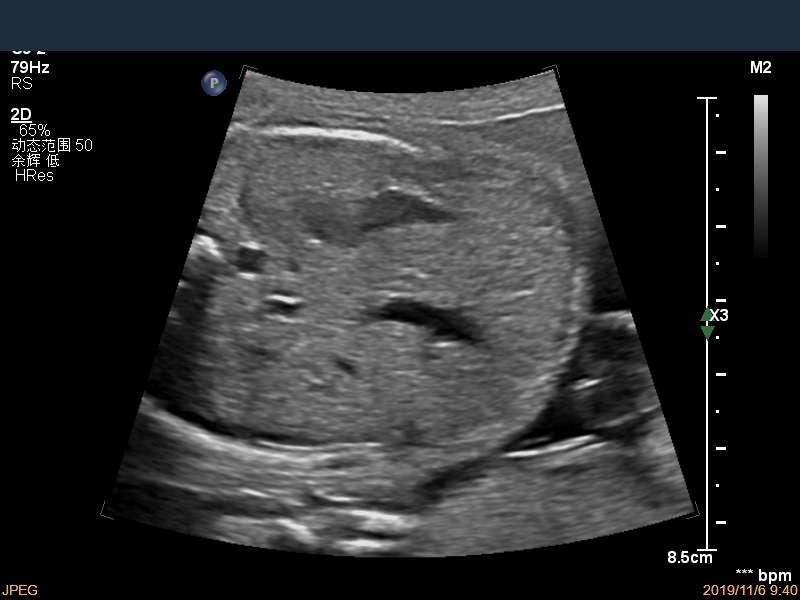

Supplement: S1 Dataset — (ZIP) [file pone.0305250.s001.zip › FE-SD-1/images/train_res/1048_ab.jpg]

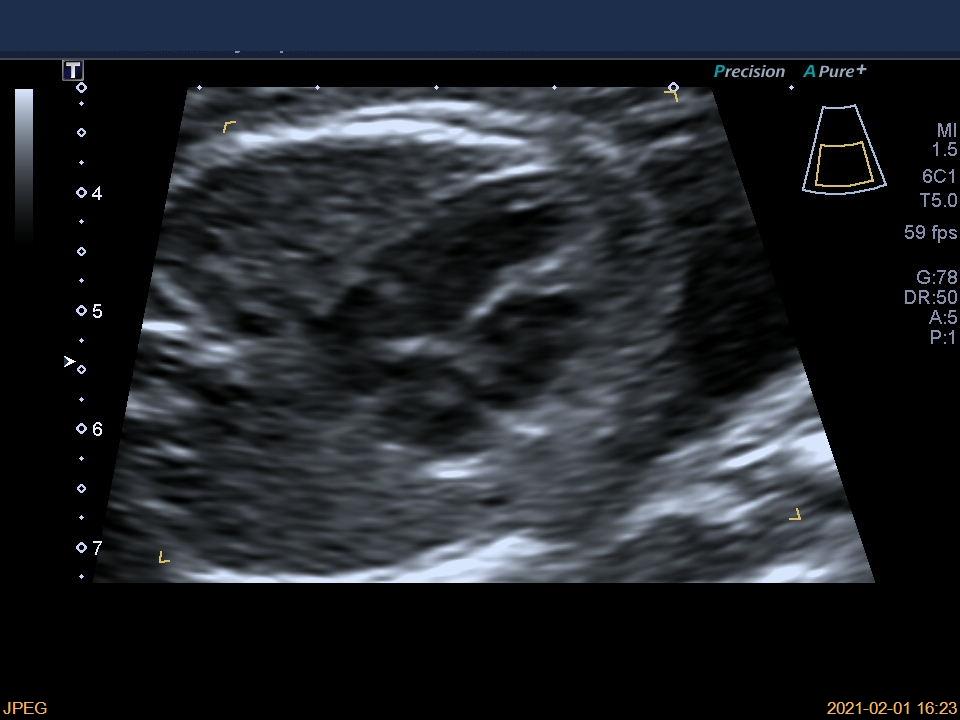

Supplement: S1 Dataset — (ZIP) [file pone.0305250.s001.zip › FE-SD-1/images/train_res/1048_fc.jpg]

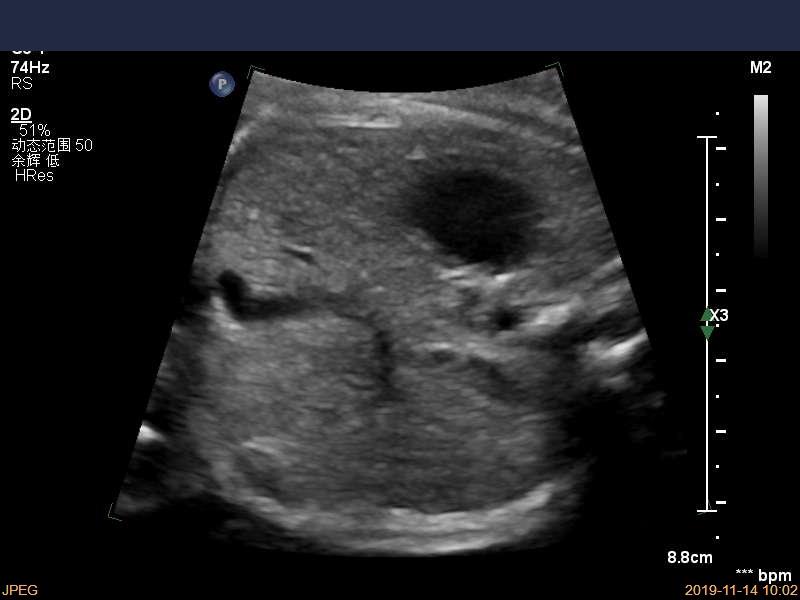

Supplement: S1 Dataset — (ZIP) [file pone.0305250.s001.zip › FE-SD-1/images/train_res/1049_ab.jpg]

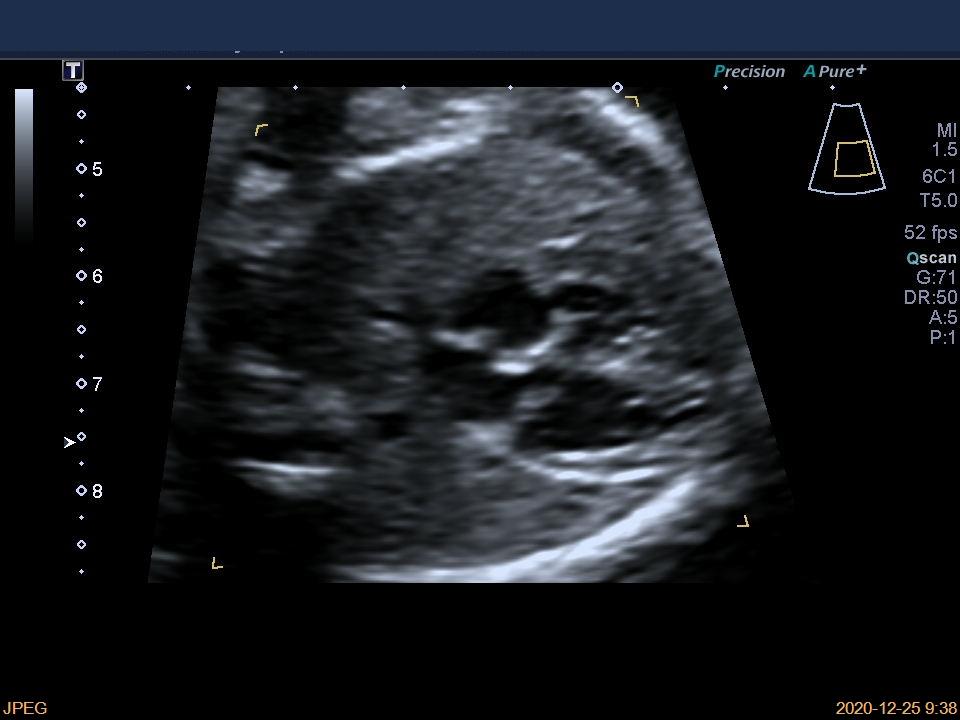

Supplement: S1 Dataset — (ZIP) [file pone.0305250.s001.zip › FE-SD-1/images/train_res/1049_fc.jpg]

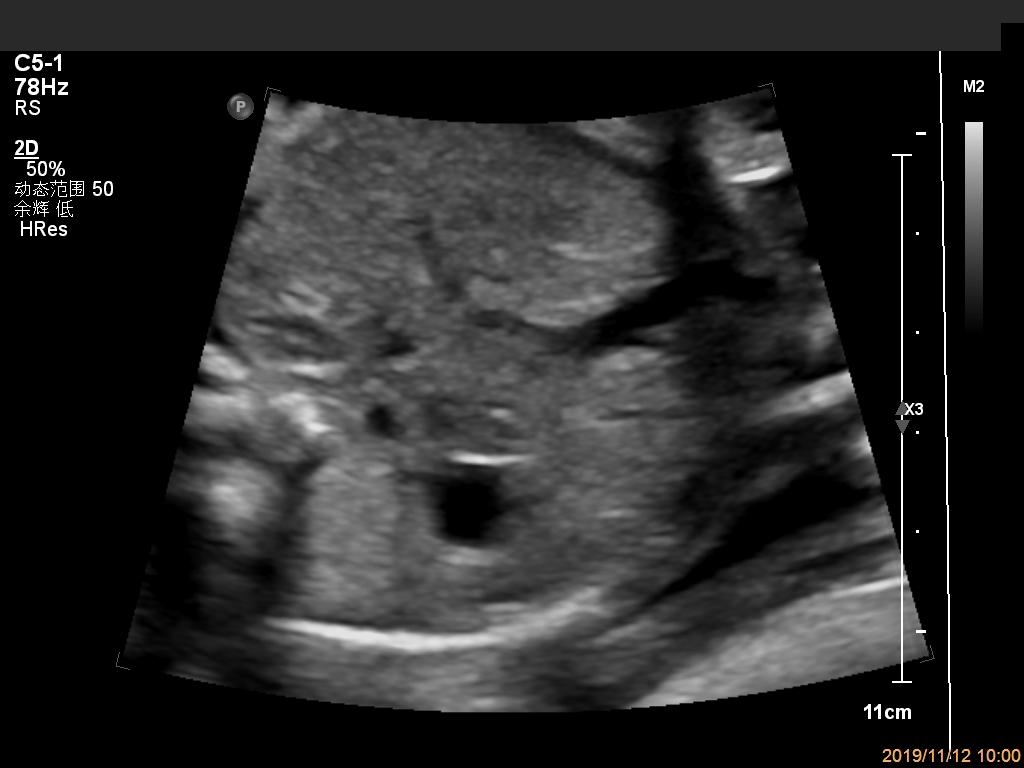

Supplement: S1 Dataset — (ZIP) [file pone.0305250.s001.zip › FE-SD-1/images/train_res/1050_ab.jpg]

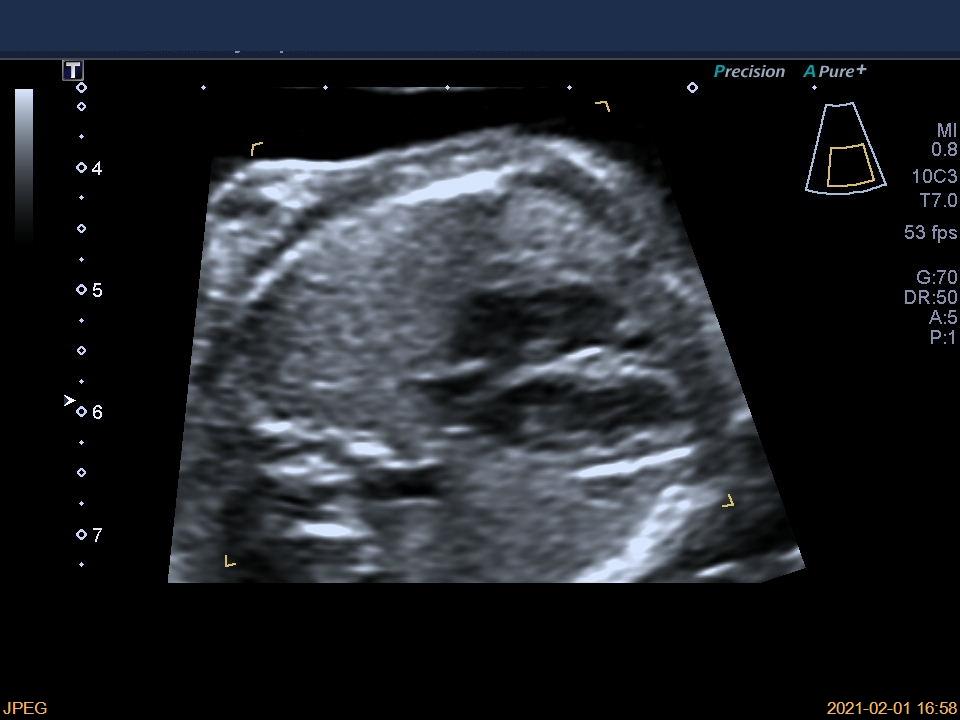

Supplement: S1 Dataset — (ZIP) [file pone.0305250.s001.zip › FE-SD-1/images/train_res/1050_fc.jpg]

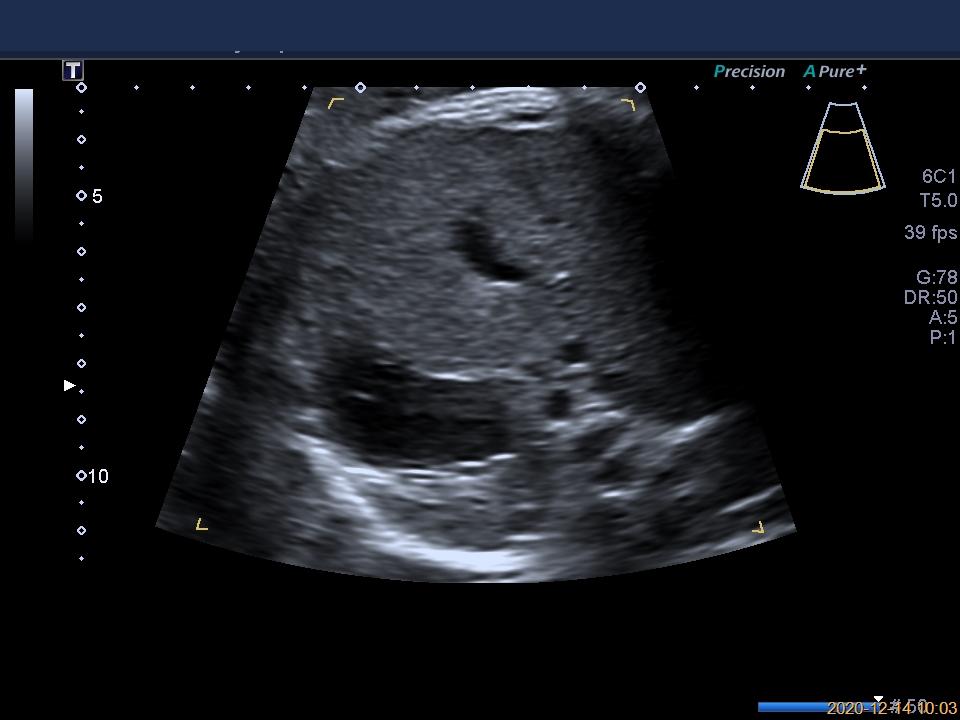

Supplement: S1 Dataset — (ZIP) [file pone.0305250.s001.zip › FE-SD-1/images/train_res/1051_ab.jpg]

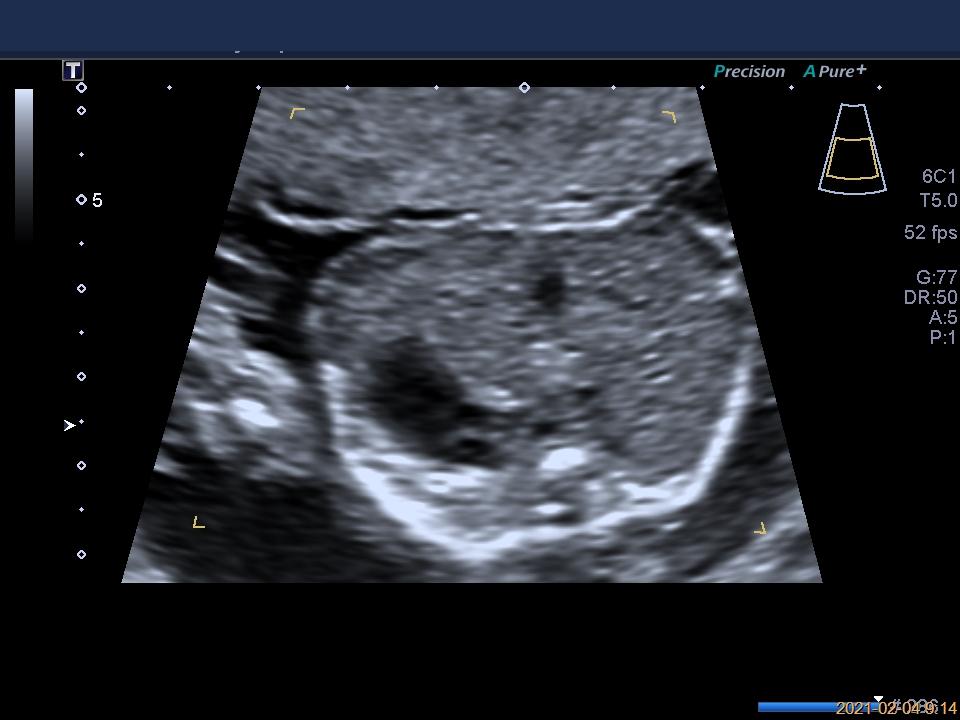

Supplement: S1 Dataset — (ZIP) [file pone.0305250.s001.zip › FE-SD-1/images/train_res/1052_ab.jpg]

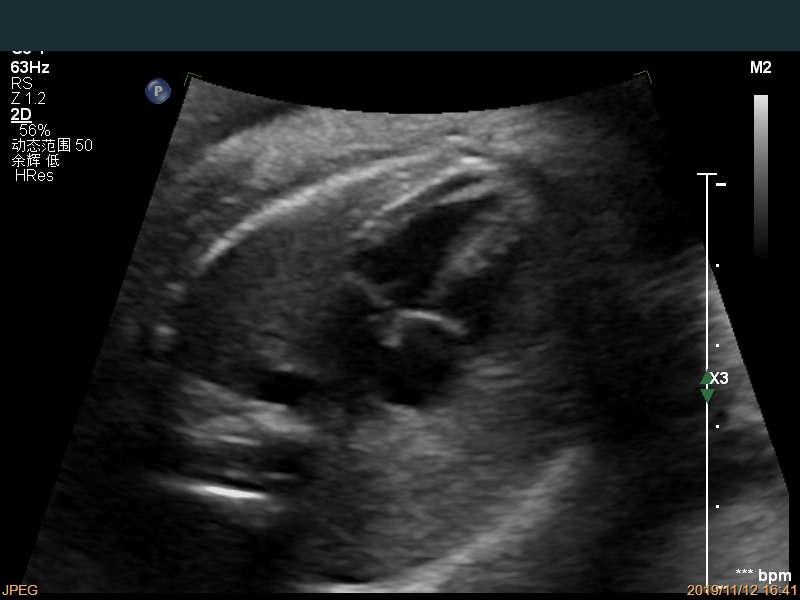

Supplement: S1 Dataset — (ZIP) [file pone.0305250.s001.zip › FE-SD-1/images/train_res/1052_fc.jpg]

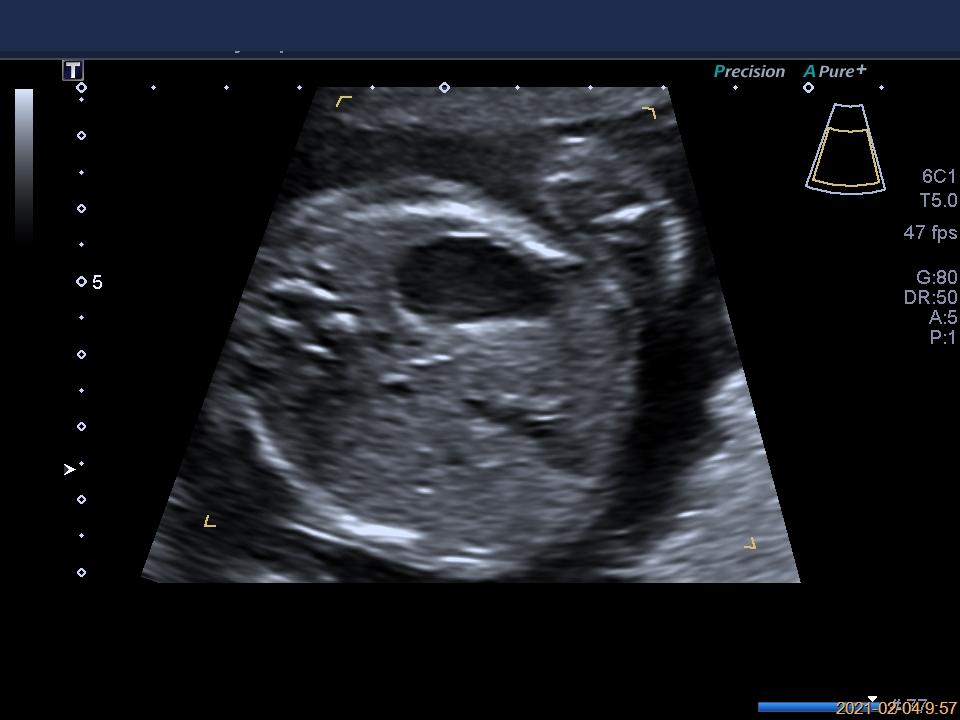

Supplement: S1 Dataset — (ZIP) [file pone.0305250.s001.zip › FE-SD-1/images/train_res/1053_ab.jpg]

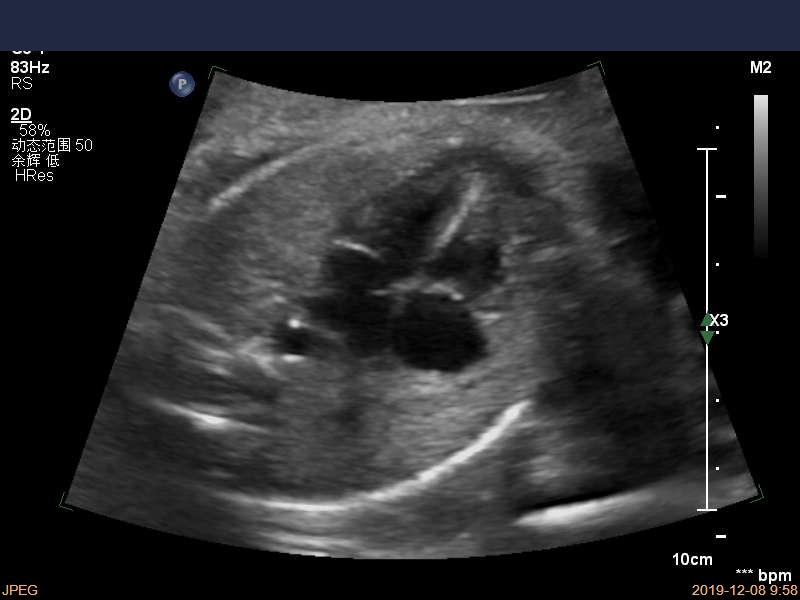

Supplement: S1 Dataset — (ZIP) [file pone.0305250.s001.zip › FE-SD-1/images/train_res/1053_fc.jpg]

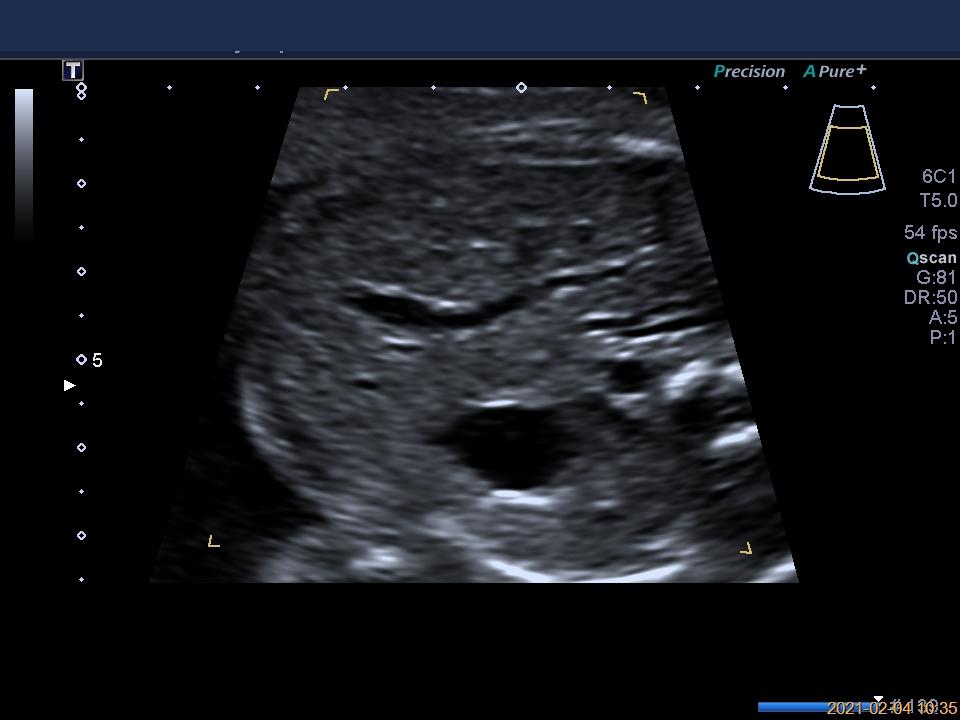

Supplement: S1 Dataset — (ZIP) [file pone.0305250.s001.zip › FE-SD-1/images/train_res/1054_ab.jpg]

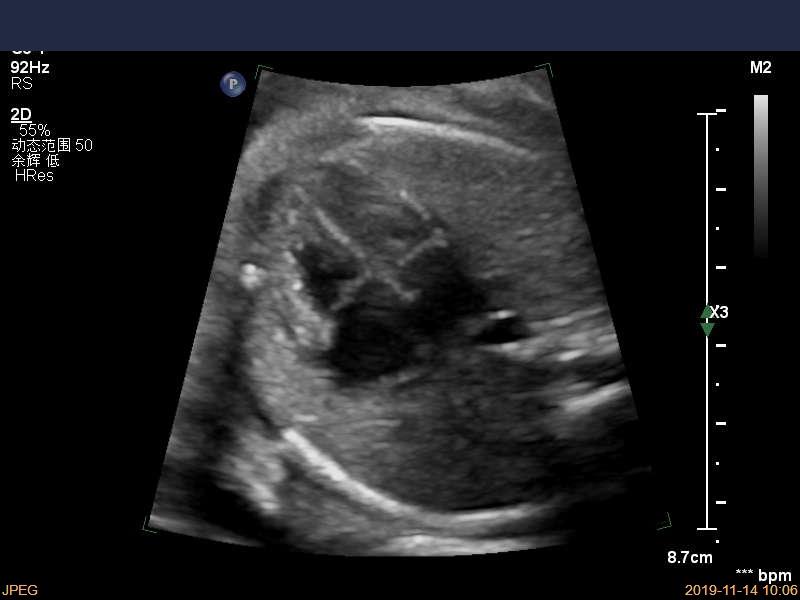

Supplement: S1 Dataset — (ZIP) [file pone.0305250.s001.zip › FE-SD-1/images/train_res/1054_fc.jpg]

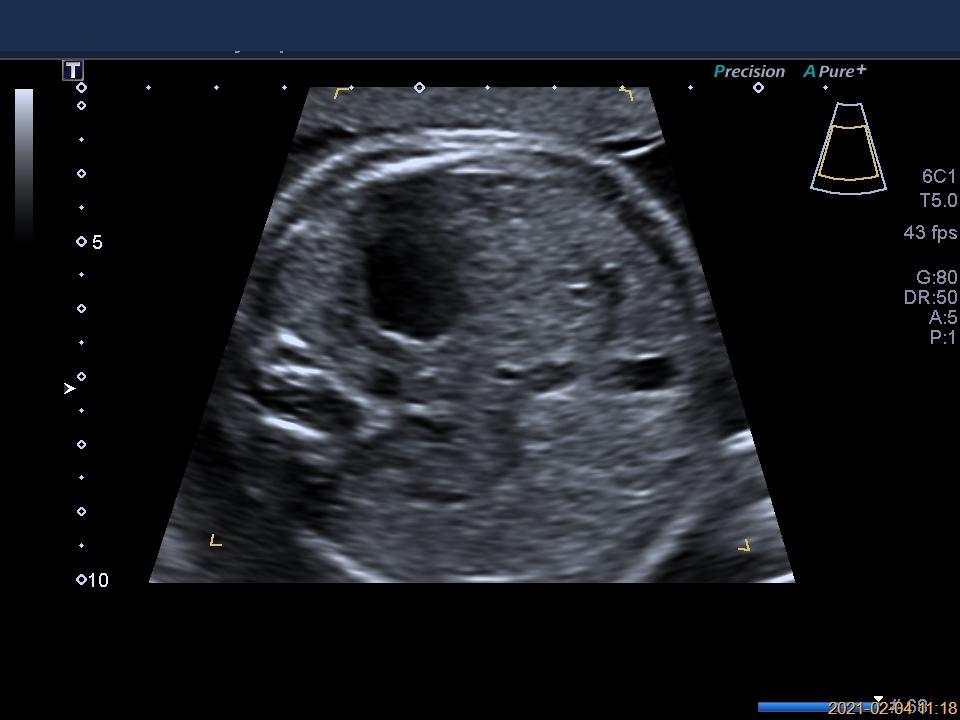

Supplement: S1 Dataset — (ZIP) [file pone.0305250.s001.zip › FE-SD-1/images/train_res/1055_ab.jpg]

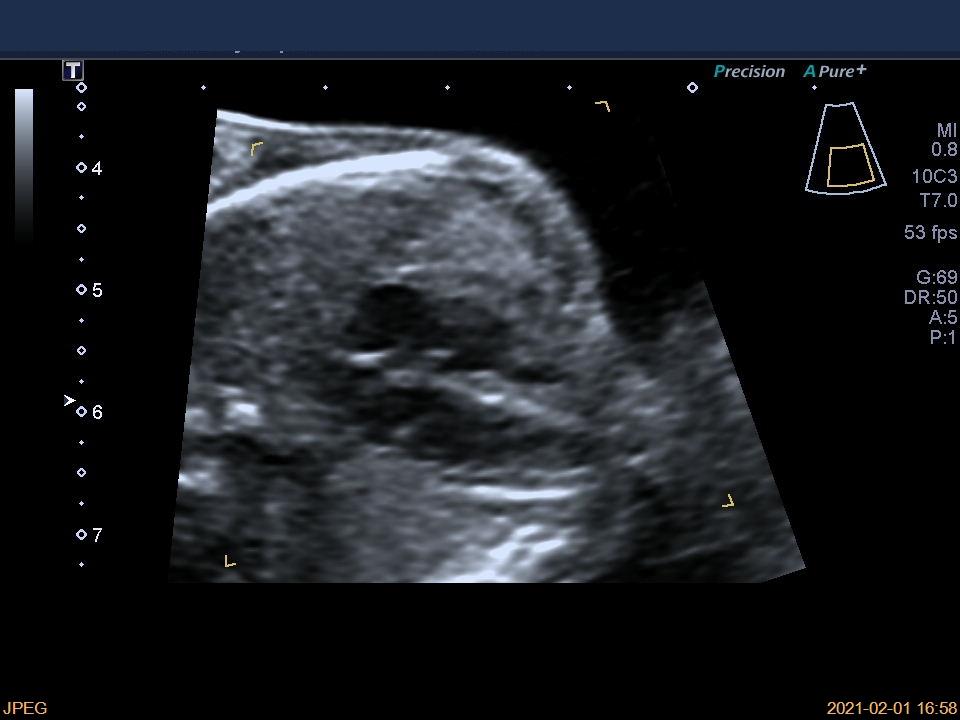

Supplement: S1 Dataset — (ZIP) [file pone.0305250.s001.zip › FE-SD-1/images/train_res/1055_fc.jpg]

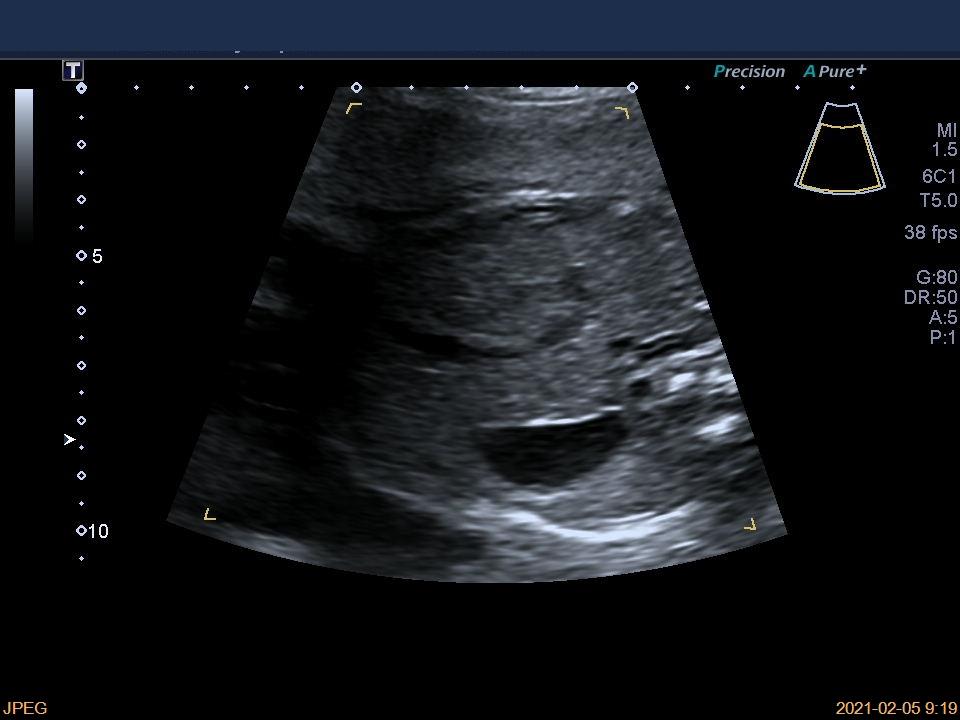

Supplement: S1 Dataset — (ZIP) [file pone.0305250.s001.zip › FE-SD-1/images/train_res/1056_ab.jpg]

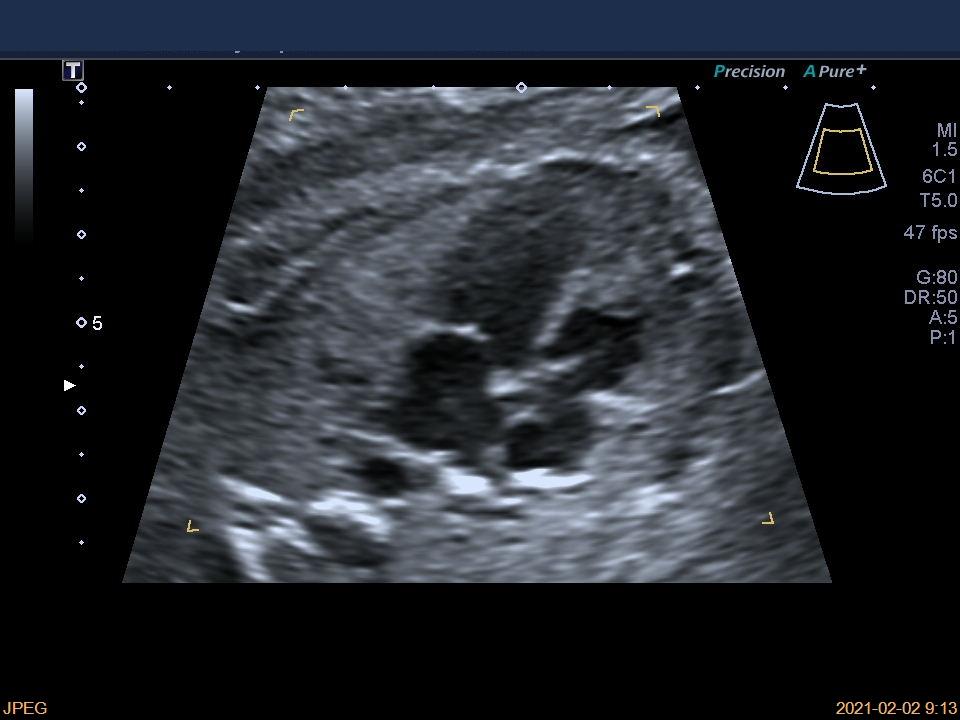

Supplement: S1 Dataset — (ZIP) [file pone.0305250.s001.zip › FE-SD-1/images/train_res/1056_fc.jpg]

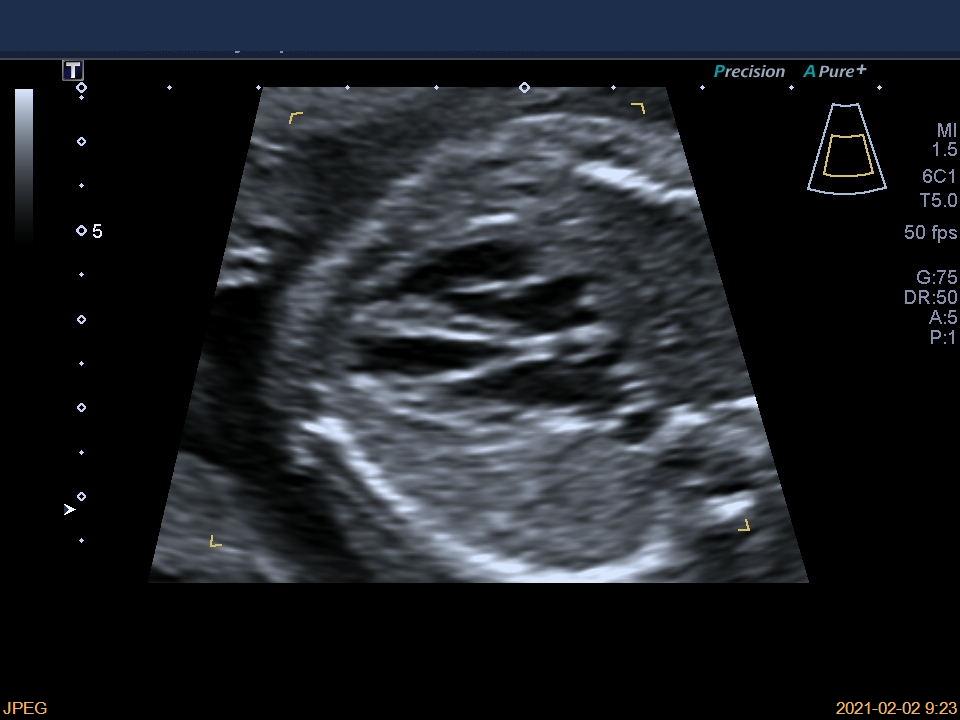

Supplement: S1 Dataset — (ZIP) [file pone.0305250.s001.zip › FE-SD-1/images/train_res/1057_fc.jpg]

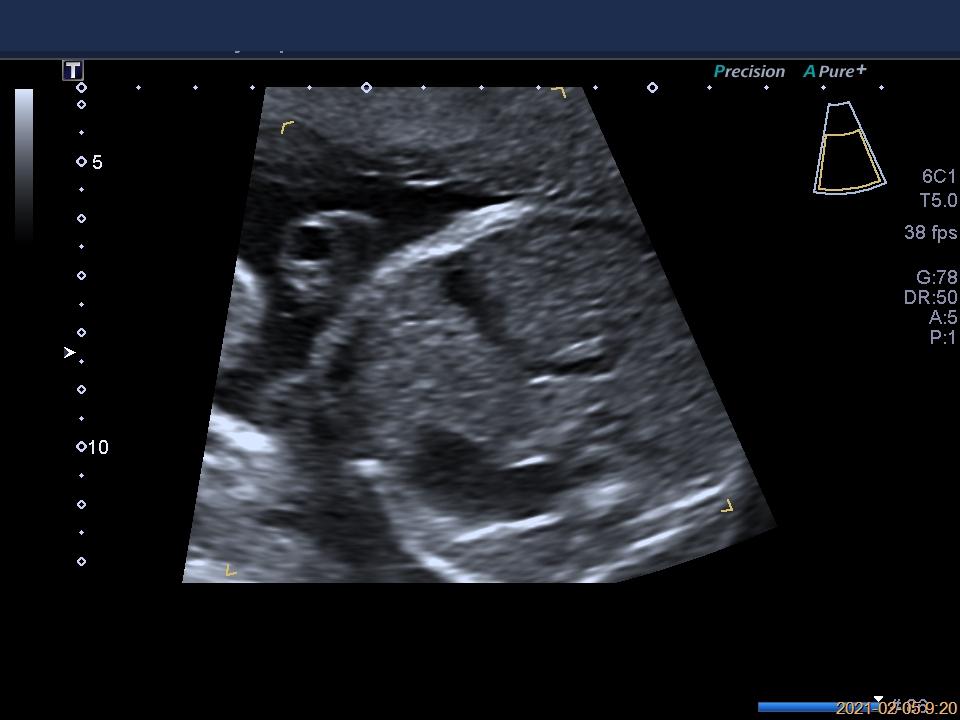

Supplement: S1 Dataset — (ZIP) [file pone.0305250.s001.zip › FE-SD-1/images/train_res/1058_ab.jpg]

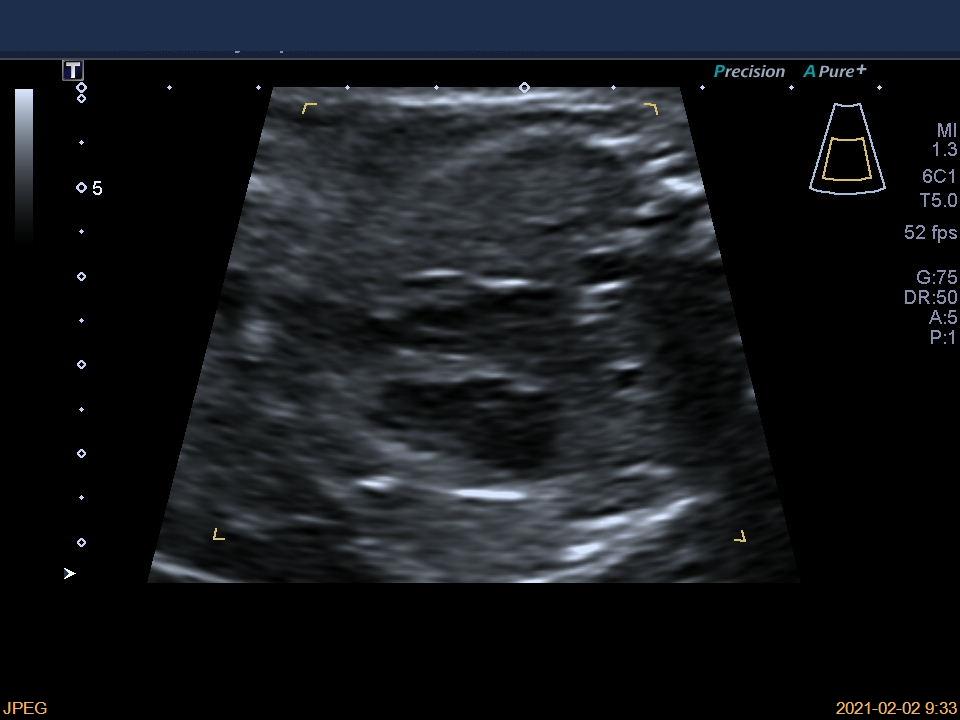

Supplement: S1 Dataset — (ZIP) [file pone.0305250.s001.zip › FE-SD-1/images/train_res/1058_fc.jpg]

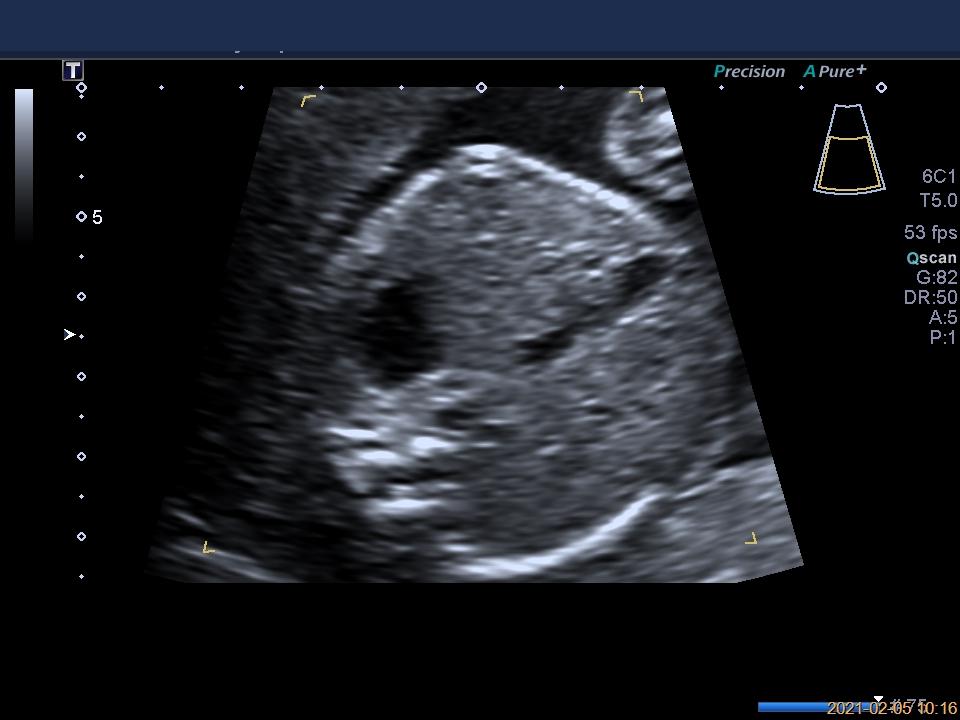

Supplement: S1 Dataset — (ZIP) [file pone.0305250.s001.zip › FE-SD-1/images/train_res/1059_ab.jpg]

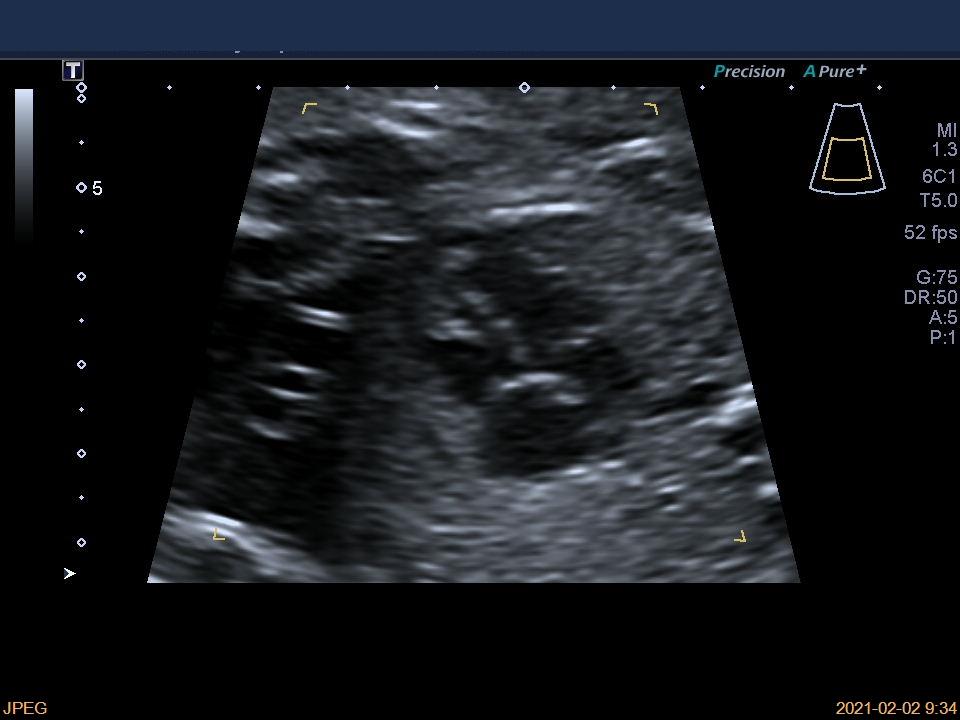

Supplement: S1 Dataset — (ZIP) [file pone.0305250.s001.zip › FE-SD-1/images/train_res/1059_fc.jpg]

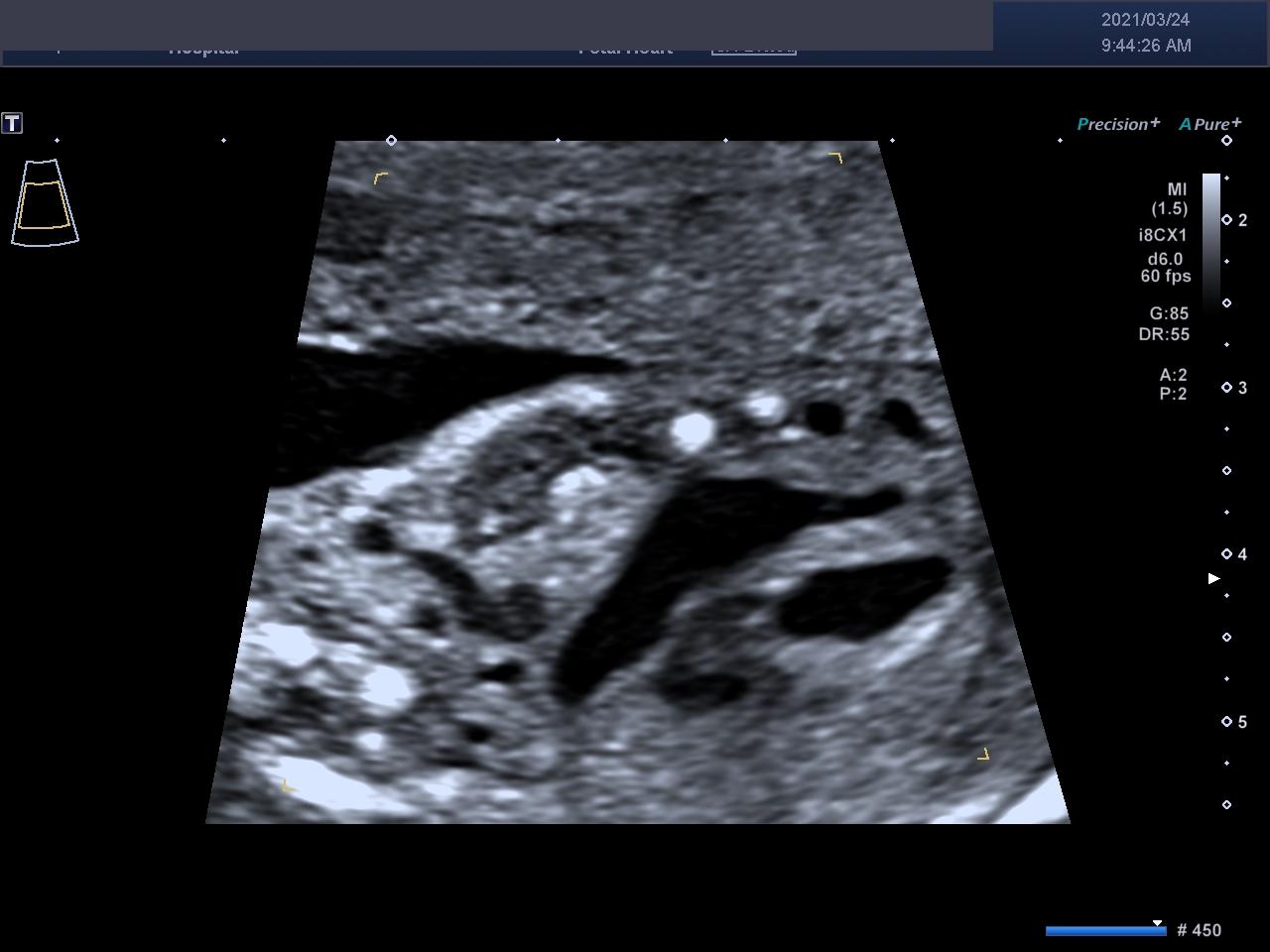

Supplement: S1 Dataset — (ZIP) [file pone.0305250.s001.zip › FE-SD-1/images/train_res/105_ro.jpg]

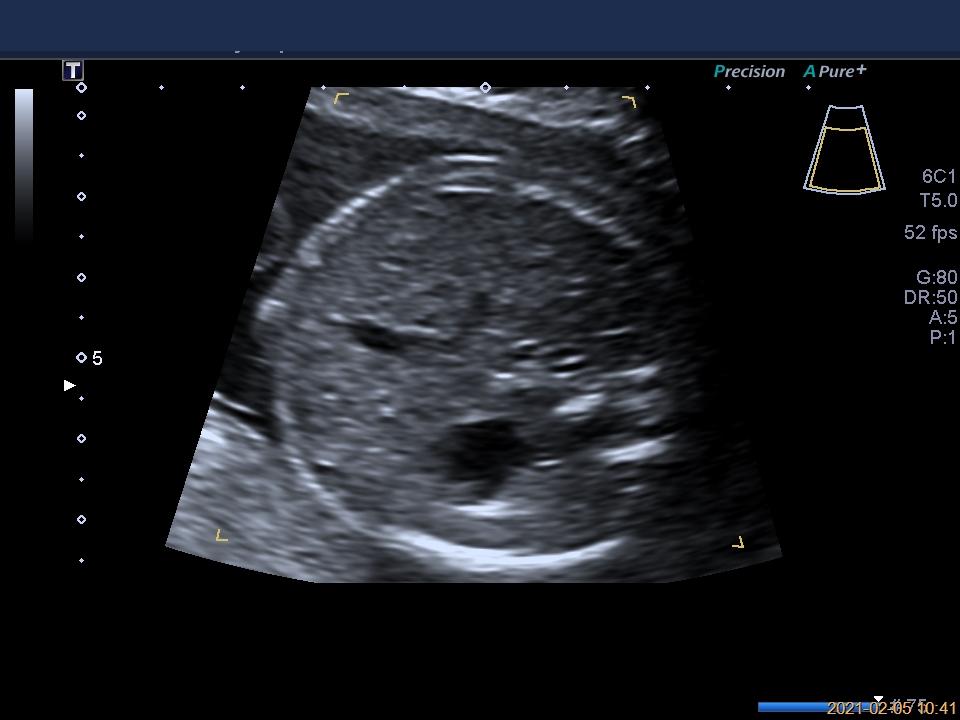

Supplement: S1 Dataset — (ZIP) [file pone.0305250.s001.zip › FE-SD-1/images/train_res/1060_ab.jpg]

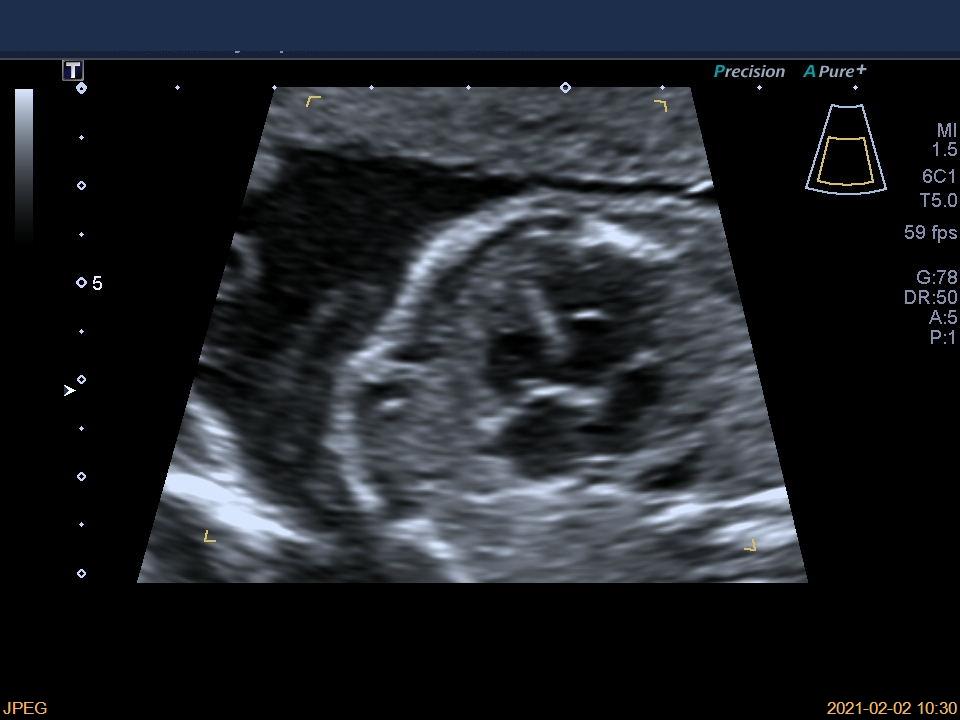

Supplement: S1 Dataset — (ZIP) [file pone.0305250.s001.zip › FE-SD-1/images/train_res/1060_fc.jpg]

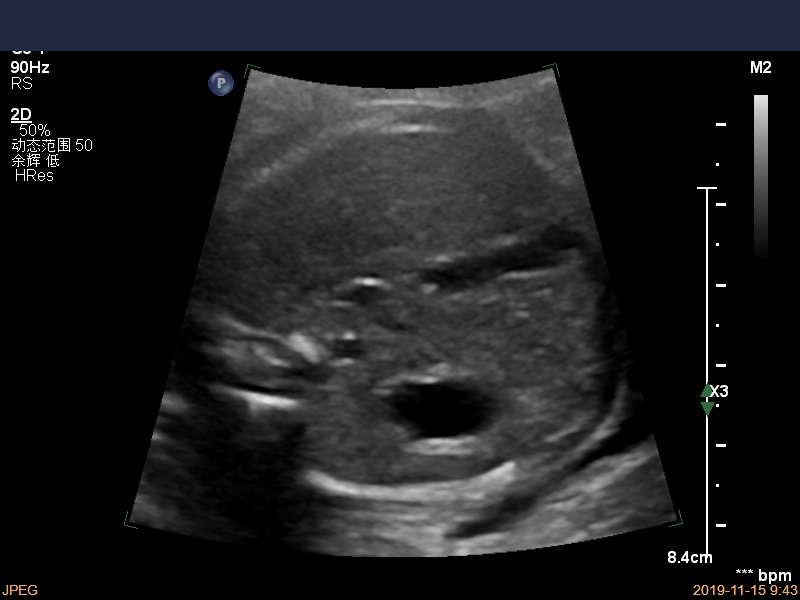

Supplement: S1 Dataset — (ZIP) [file pone.0305250.s001.zip › FE-SD-1/images/train_res/1061_ab.jpg]

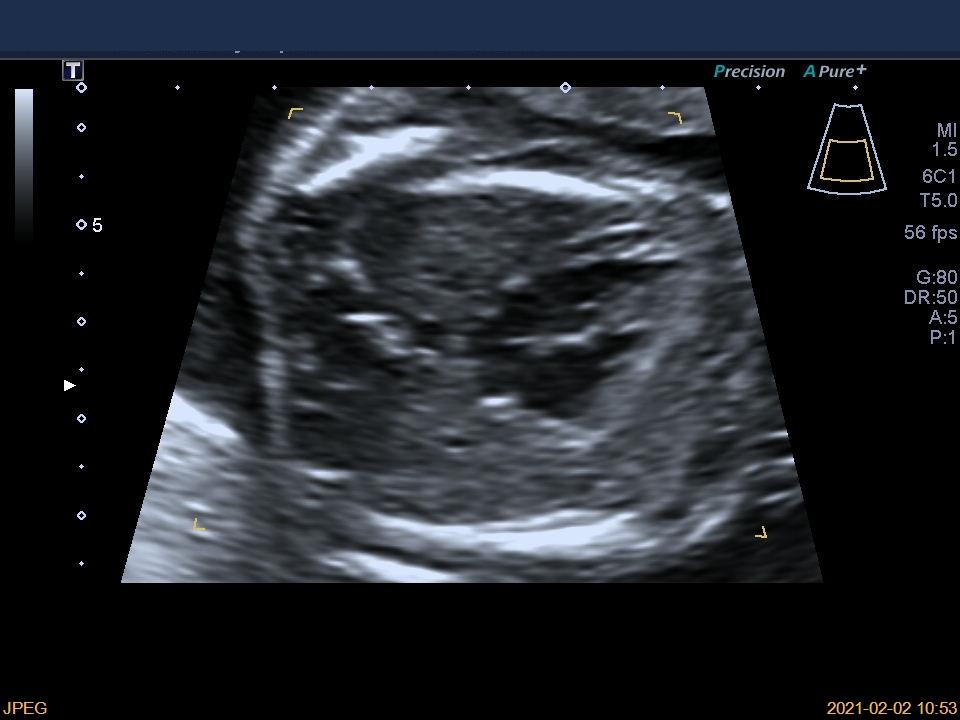

Supplement: S1 Dataset — (ZIP) [file pone.0305250.s001.zip › FE-SD-1/images/train_res/1061_fc.jpg]

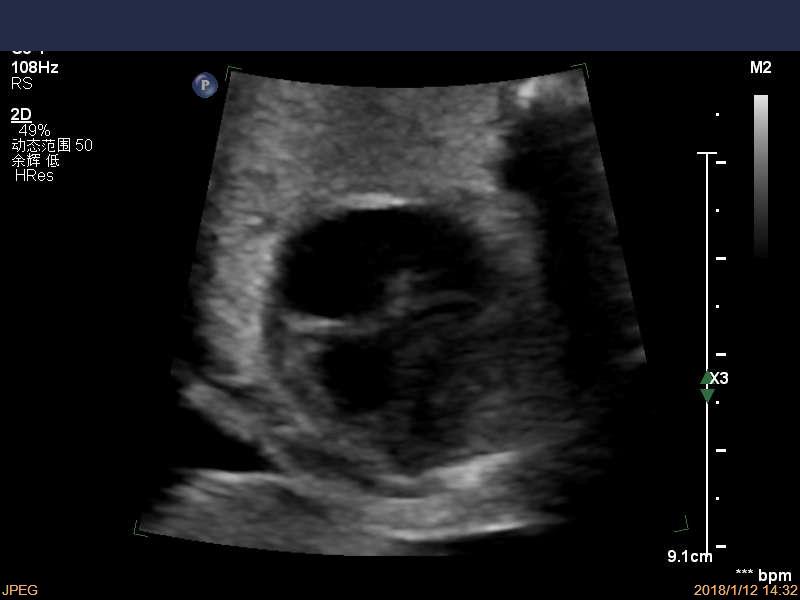

Supplement: S1 Dataset — (ZIP) [file pone.0305250.s001.zip › FE-SD-1/images/train_res/1062_fc.jpg]

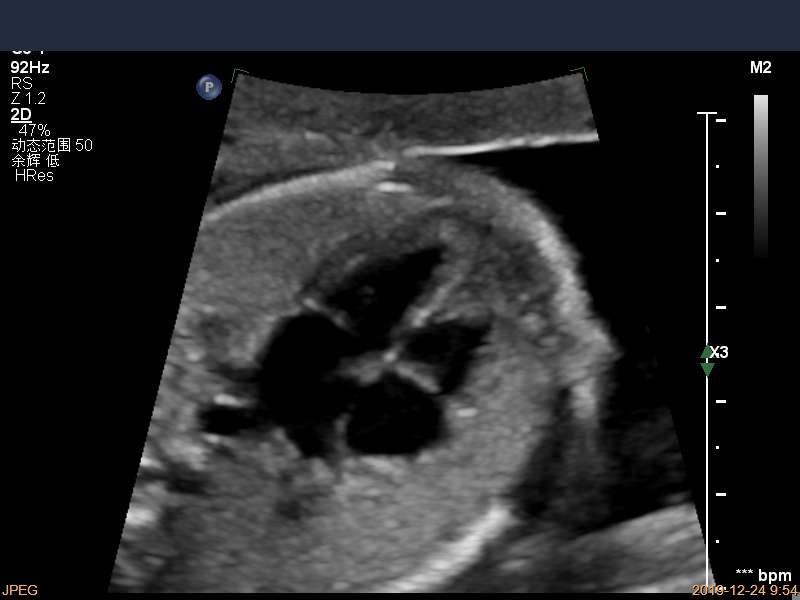

Supplement: S1 Dataset — (ZIP) [file pone.0305250.s001.zip › FE-SD-1/images/train_res/1065_fc.jpg]

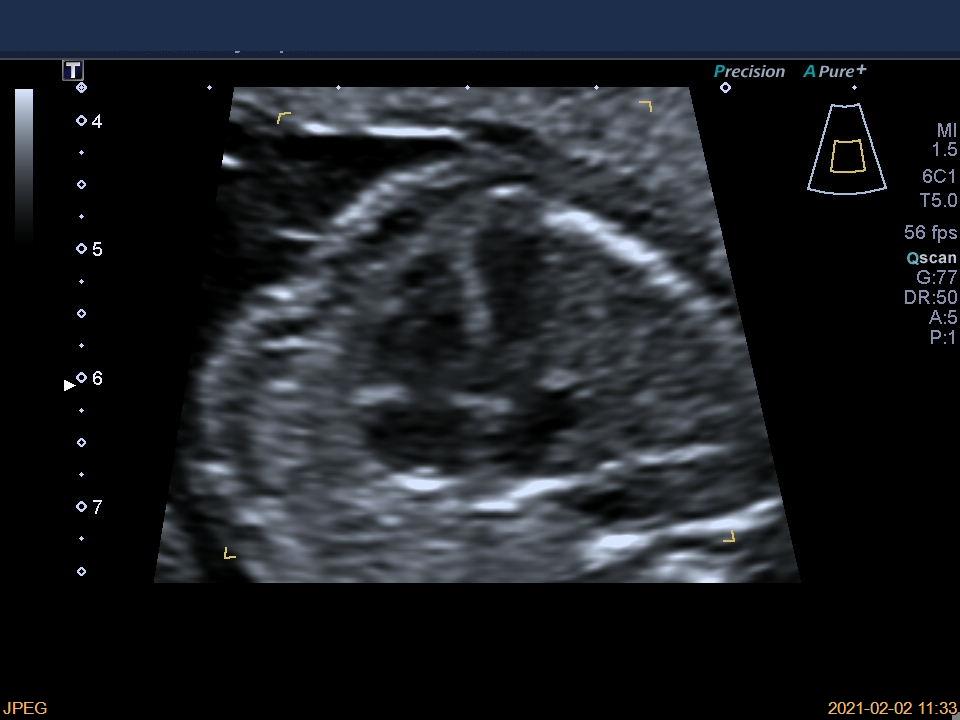

Supplement: S1 Dataset — (ZIP) [file pone.0305250.s001.zip › FE-SD-1/images/train_res/1066_fc.jpg]

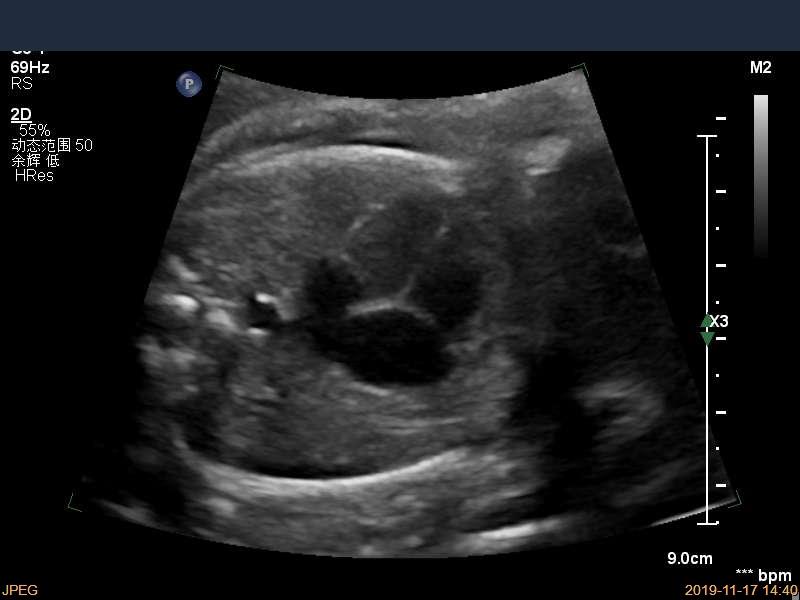

Supplement: S1 Dataset — (ZIP) [file pone.0305250.s001.zip › FE-SD-1/images/train_res/1067_fc.jpg]

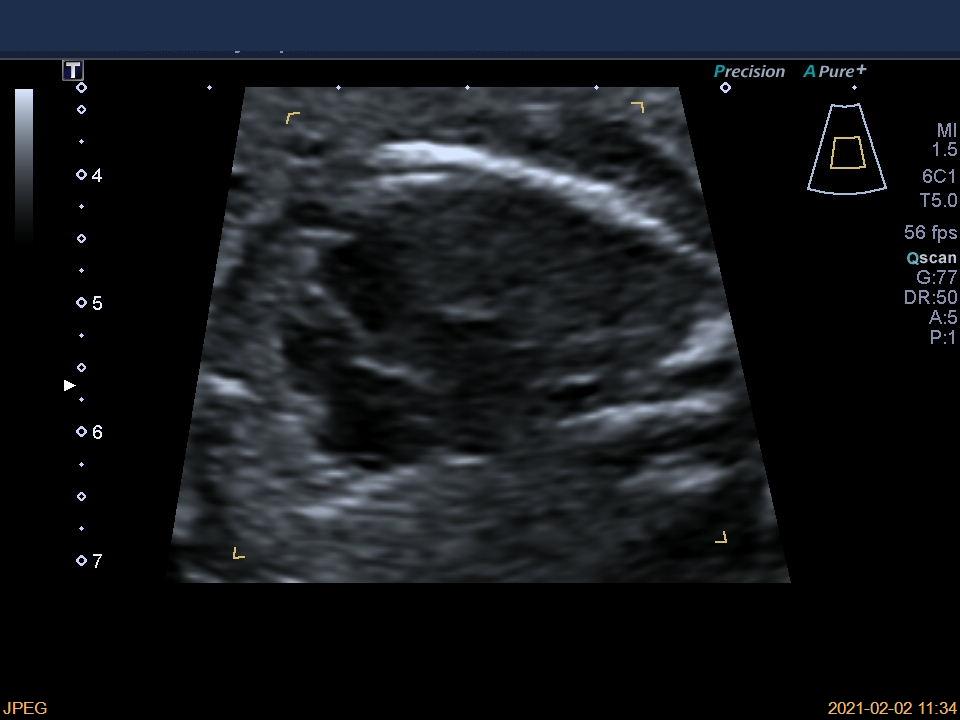

Supplement: S1 Dataset — (ZIP) [file pone.0305250.s001.zip › FE-SD-1/images/train_res/1068_fc.jpg]

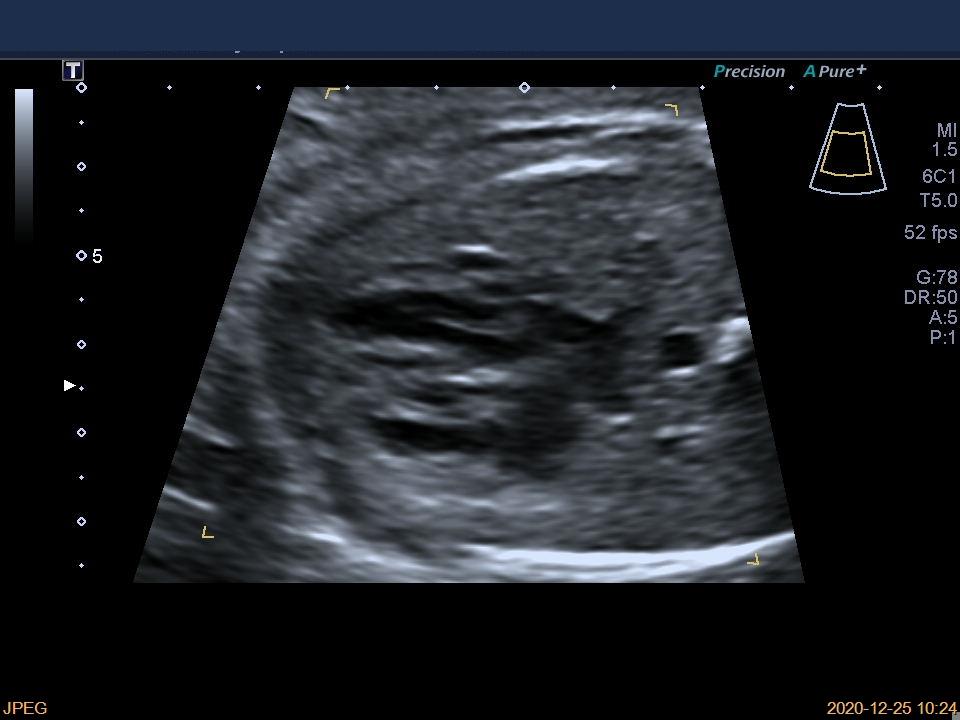

Supplement: S1 Dataset — (ZIP) [file pone.0305250.s001.zip › FE-SD-1/images/train_res/1069_fc.jpg]

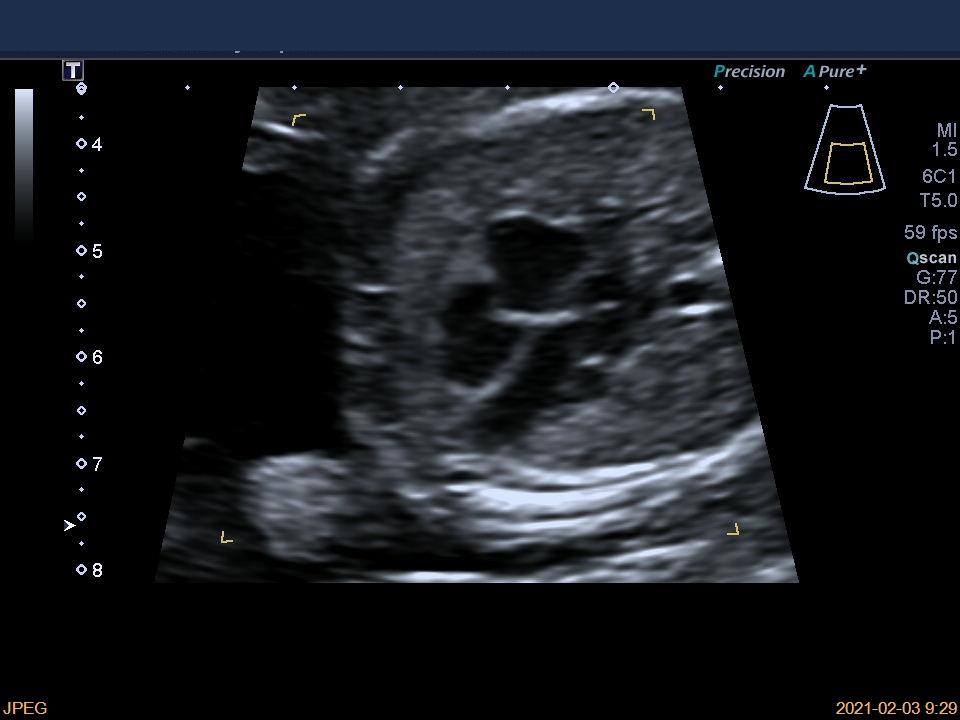

Supplement: S1 Dataset — (ZIP) [file pone.0305250.s001.zip › FE-SD-1/images/train_res/1072_fc.jpg]

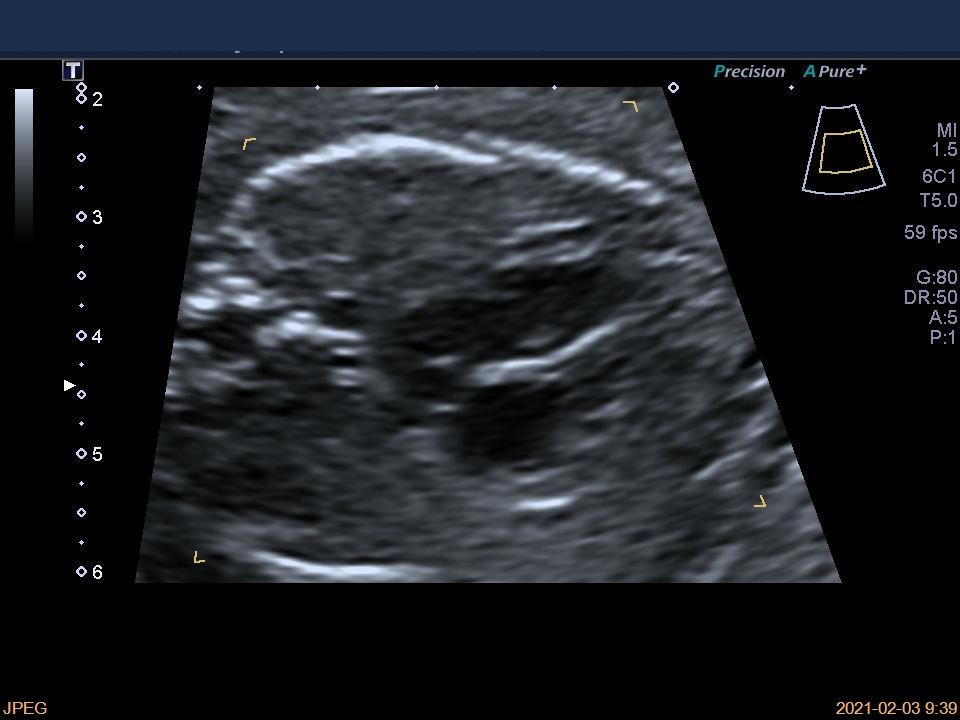

Supplement: S1 Dataset — (ZIP) [file pone.0305250.s001.zip › FE-SD-1/images/train_res/1073_fc.jpg]

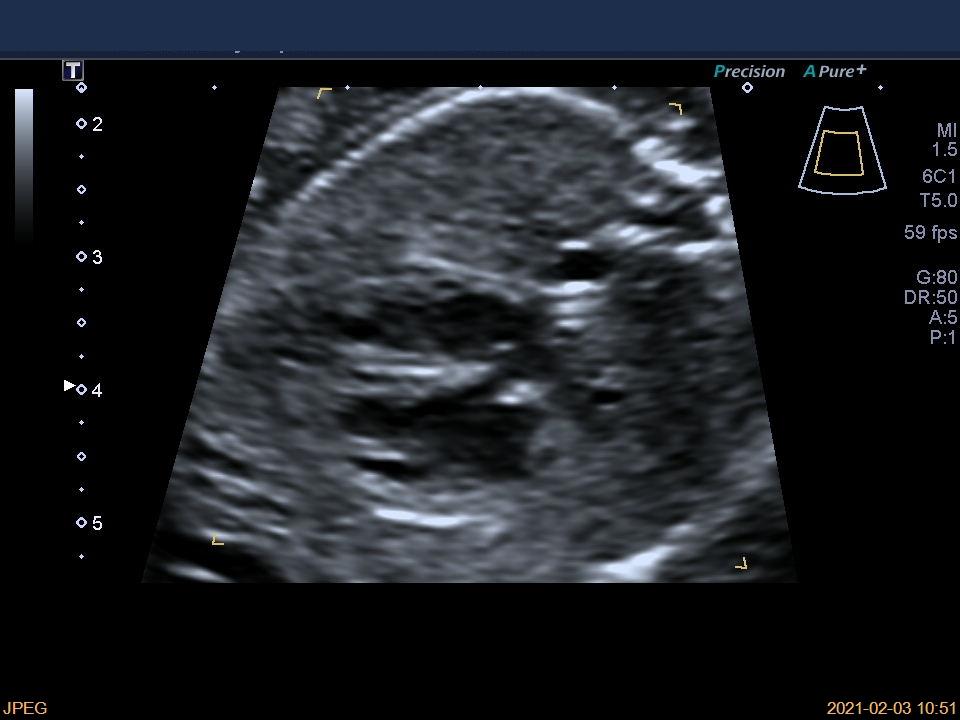

Supplement: S1 Dataset — (ZIP) [file pone.0305250.s001.zip › FE-SD-1/images/train_res/1074_fc.jpg]

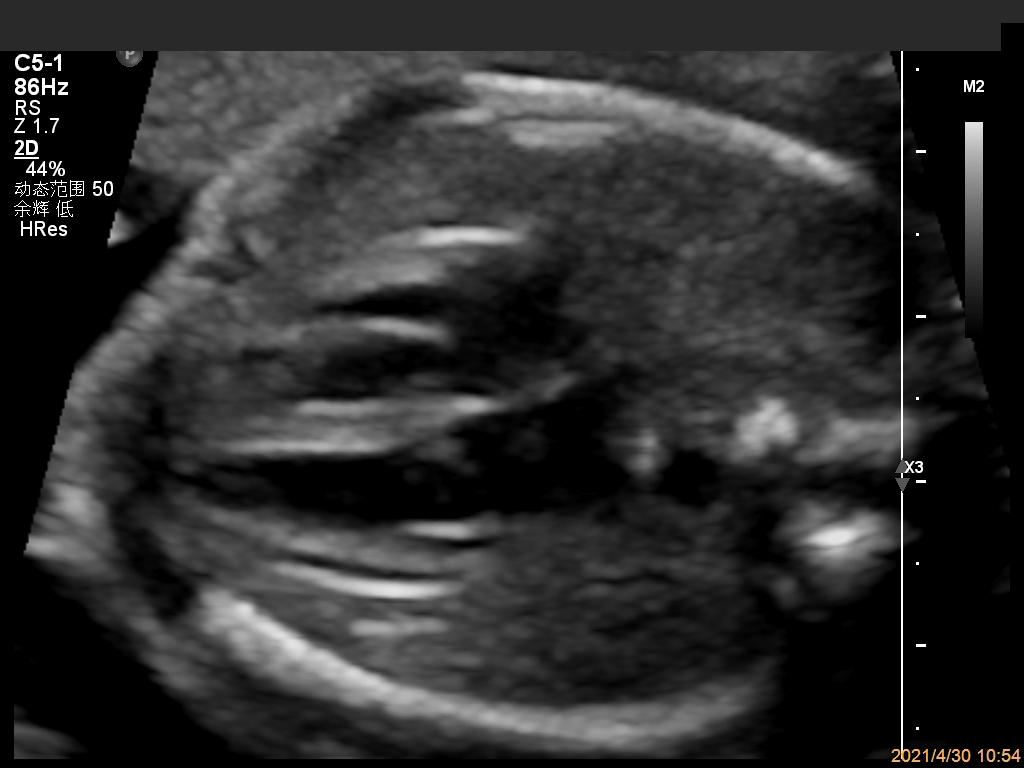

Supplement: S1 Dataset — (ZIP) [file pone.0305250.s001.zip › FE-SD-1/images/train_res/1075_fc.jpg]
