# Supplementary figures and images for: Implementation of resource-efficient fetal echocardiography detection algorithms in edge computing (part 4 of 4)
Source: PLoS One. 2024 Sep 23;19(9):e0305250. doi: 10.1371/journal.pone.0305250 (PMC11419364; doi:10.1371/journal.pone.0305250)

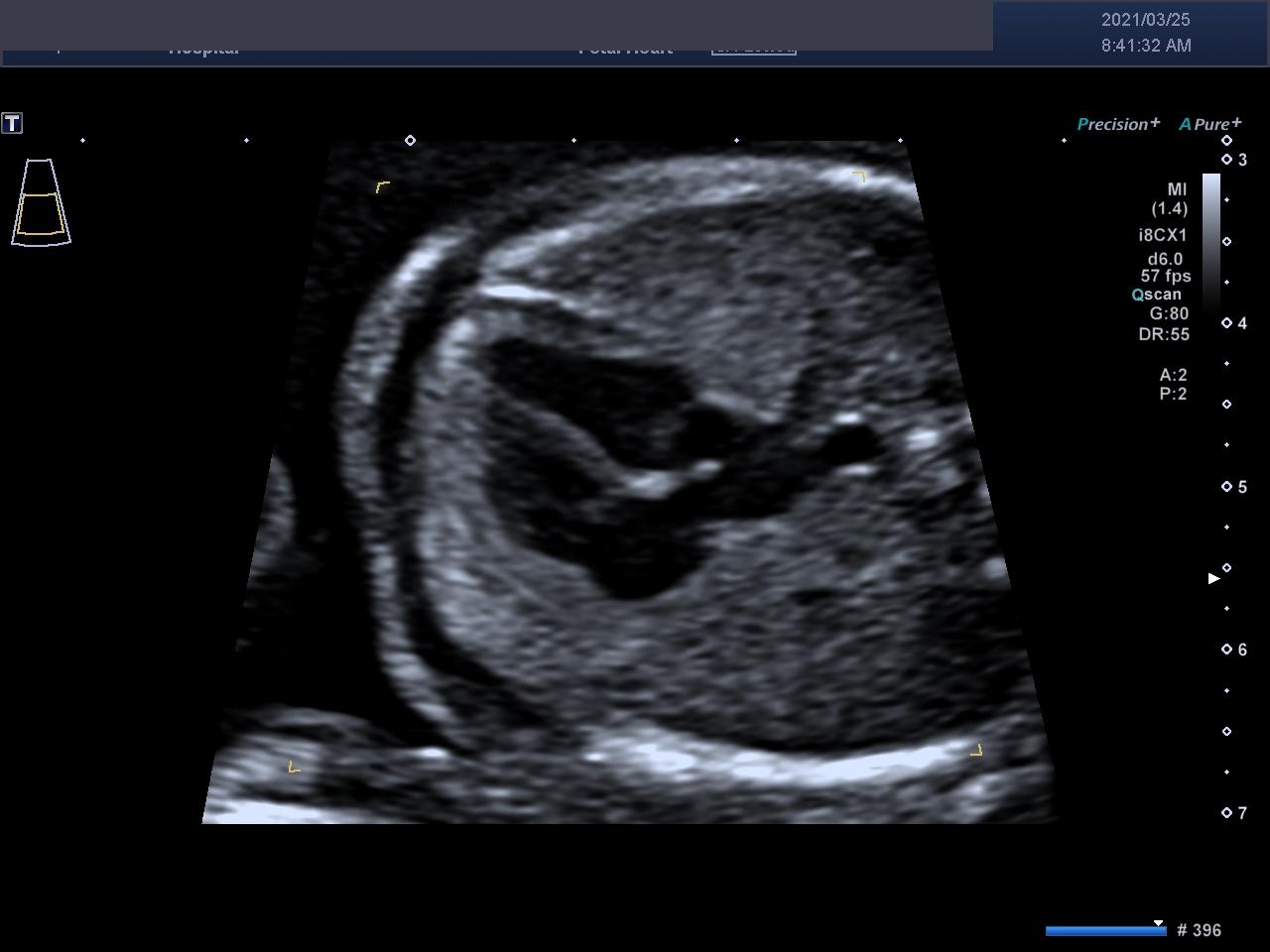

Supplement: S2 Dataset — (ZIP) [file pone.0305250.s002.zip › FE-SD-2/images/test_res/469_fc.jpg]

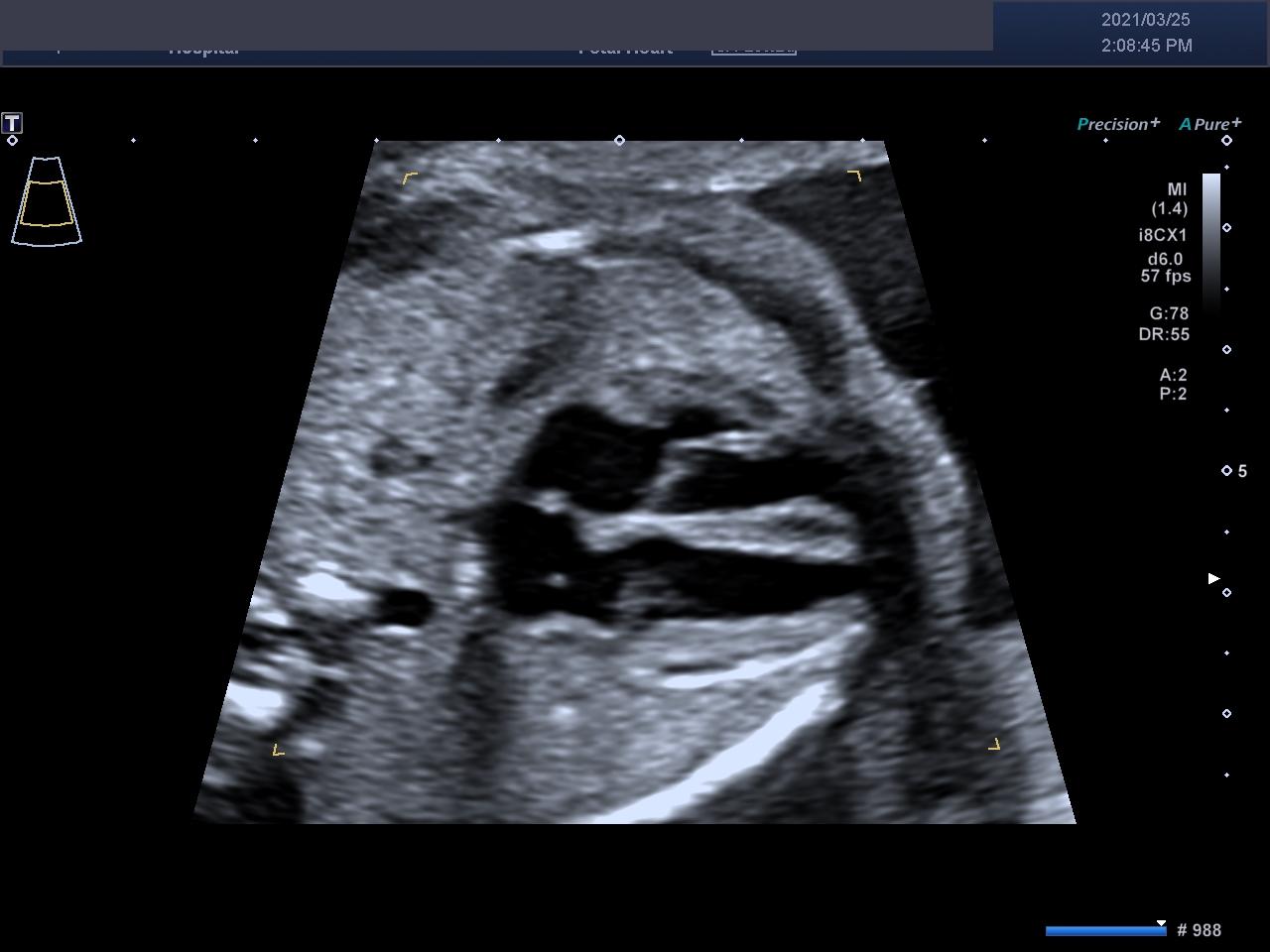

Supplement: S2 Dataset — (ZIP) [file pone.0305250.s002.zip › FE-SD-2/images/test_res/470_fc.jpg]

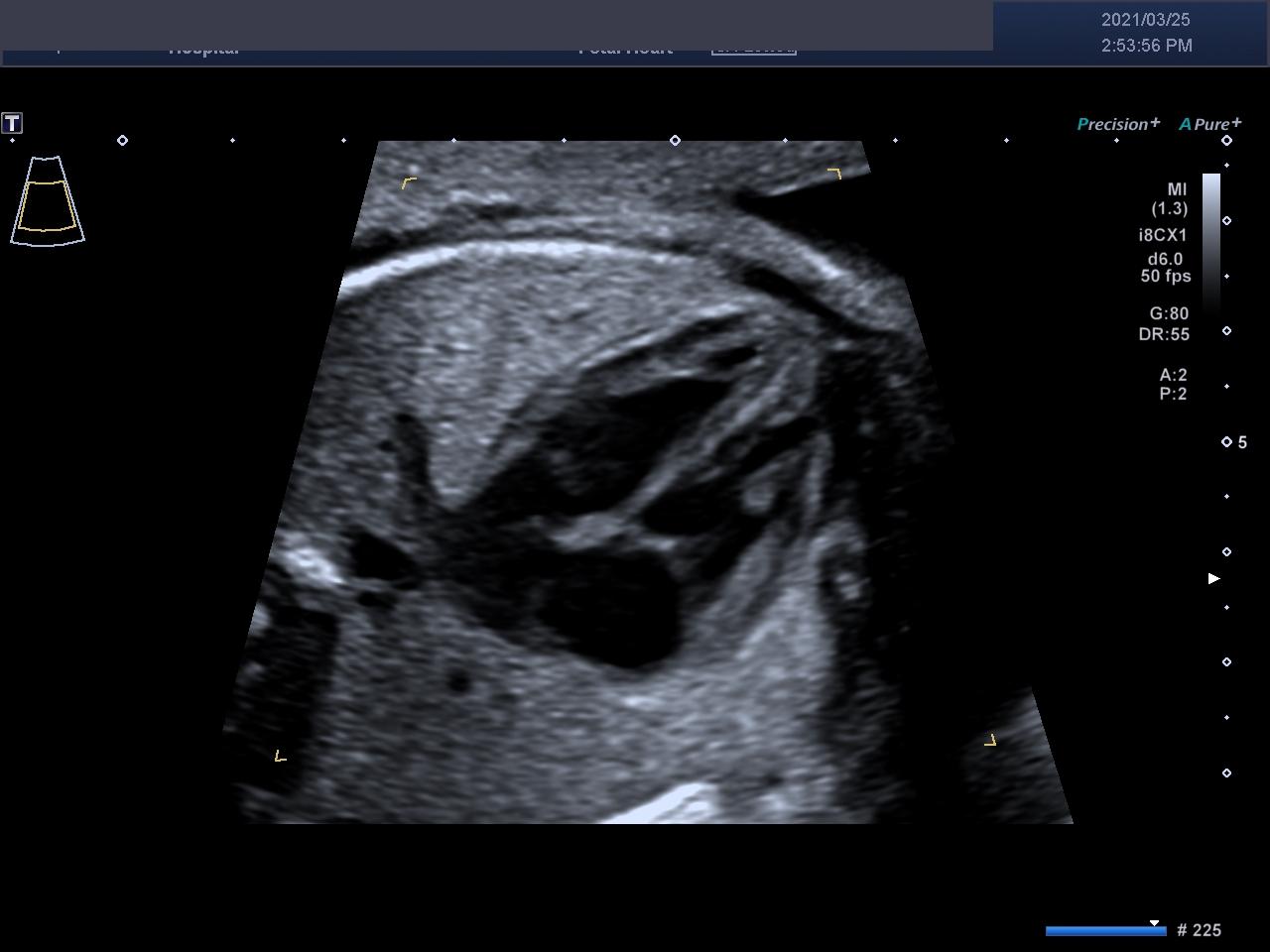

Supplement: S2 Dataset — (ZIP) [file pone.0305250.s002.zip › FE-SD-2/images/test_res/474_fc.jpg]

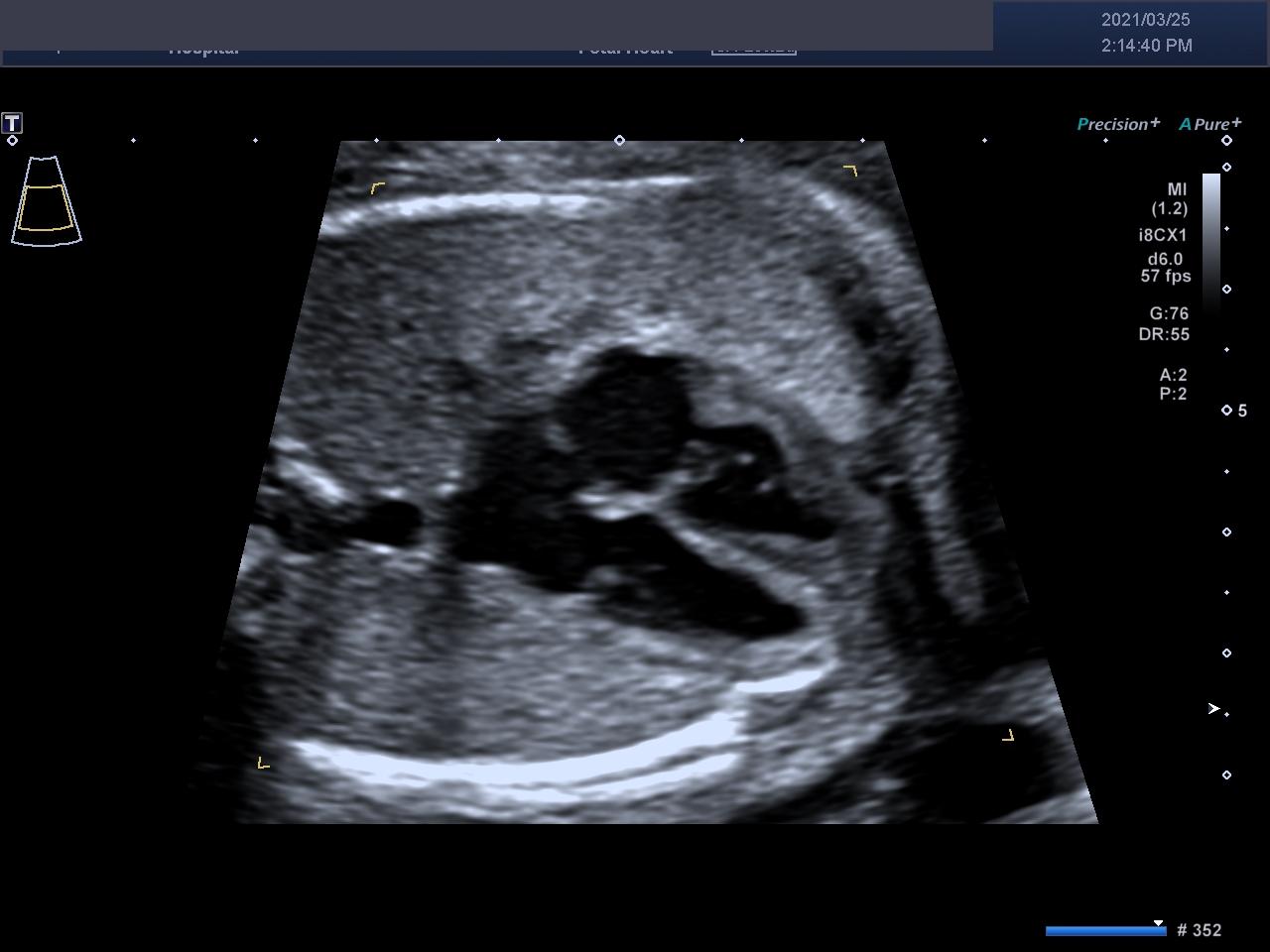

Supplement: S2 Dataset — (ZIP) [file pone.0305250.s002.zip › FE-SD-2/images/test_res/476_fc.jpg]

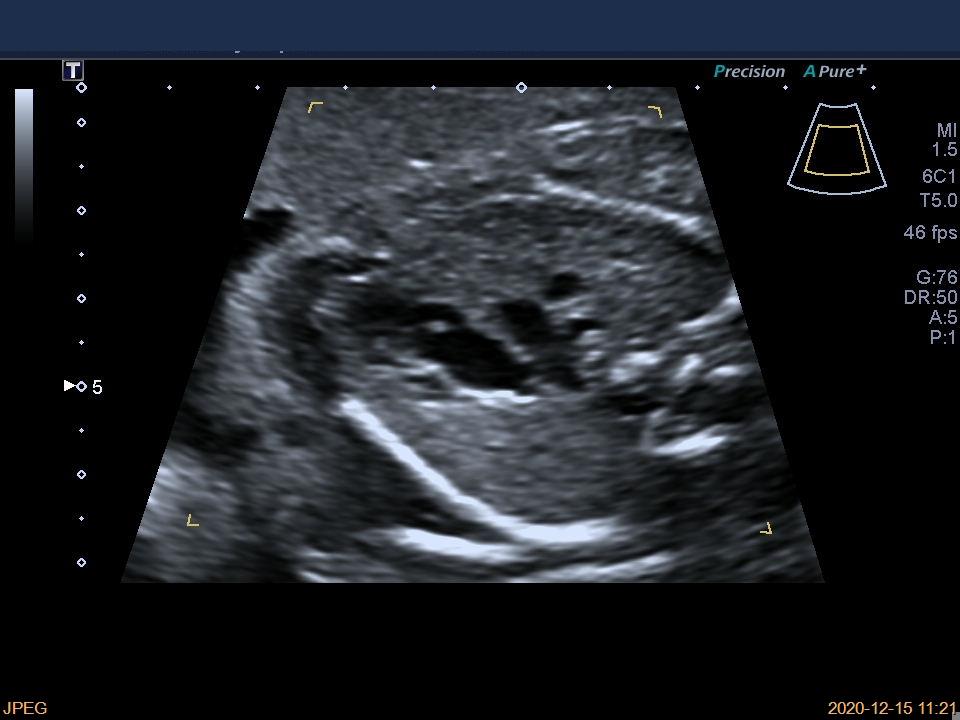

Supplement: S2 Dataset — (ZIP) [file pone.0305250.s002.zip › FE-SD-2/images/test_res/476_tv.jpg]

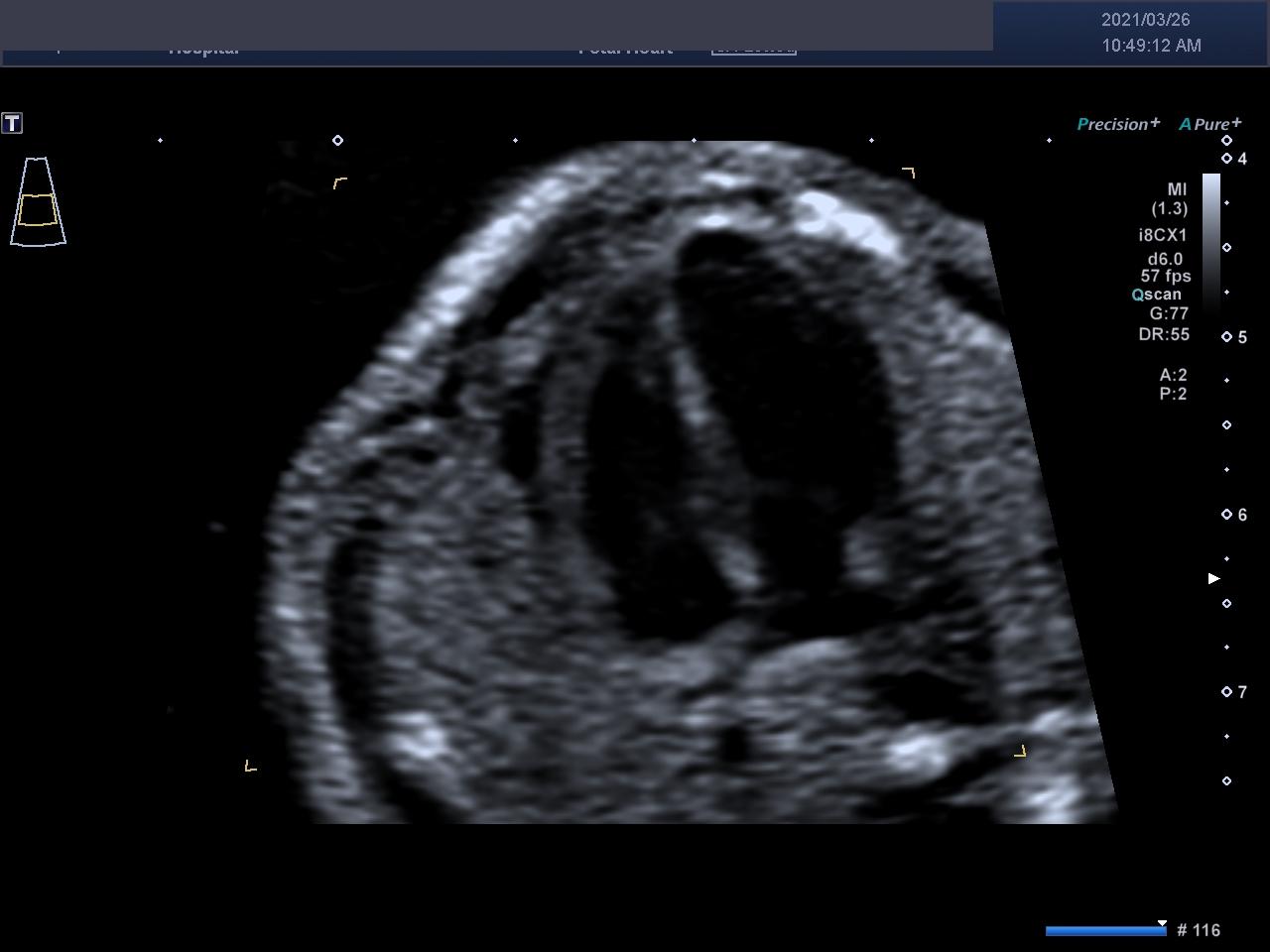

Supplement: S2 Dataset — (ZIP) [file pone.0305250.s002.zip › FE-SD-2/images/test_res/477_fc.jpg]

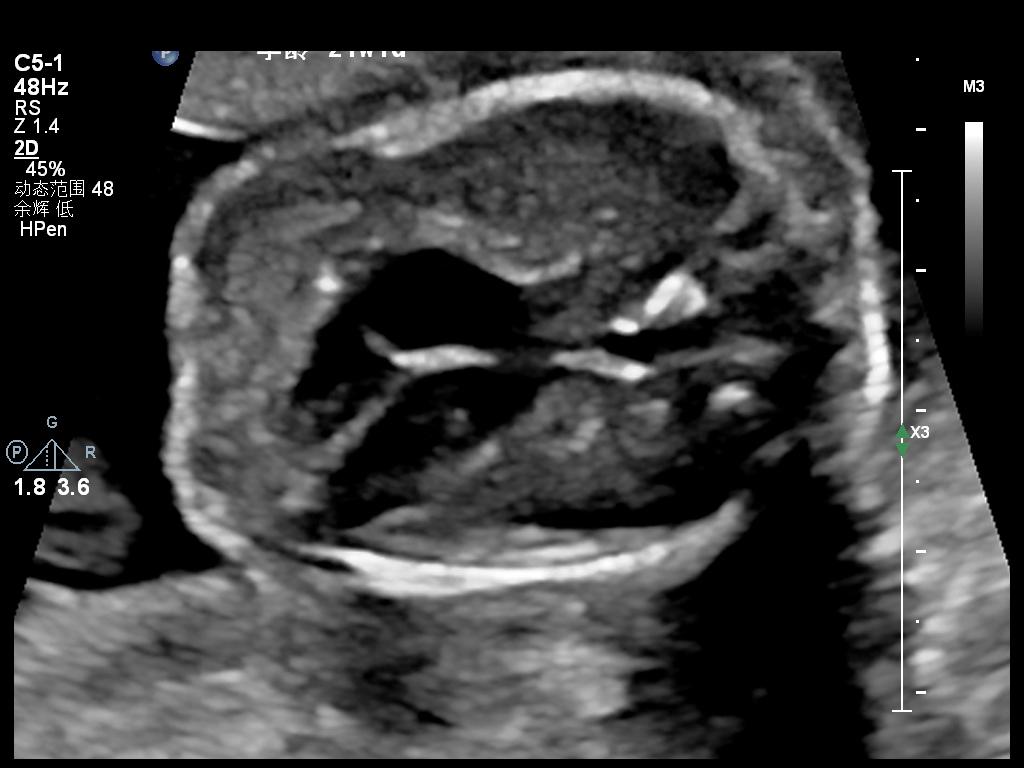

Supplement: S2 Dataset — (ZIP) [file pone.0305250.s002.zip › FE-SD-2/images/test_res/480_fc.jpg]

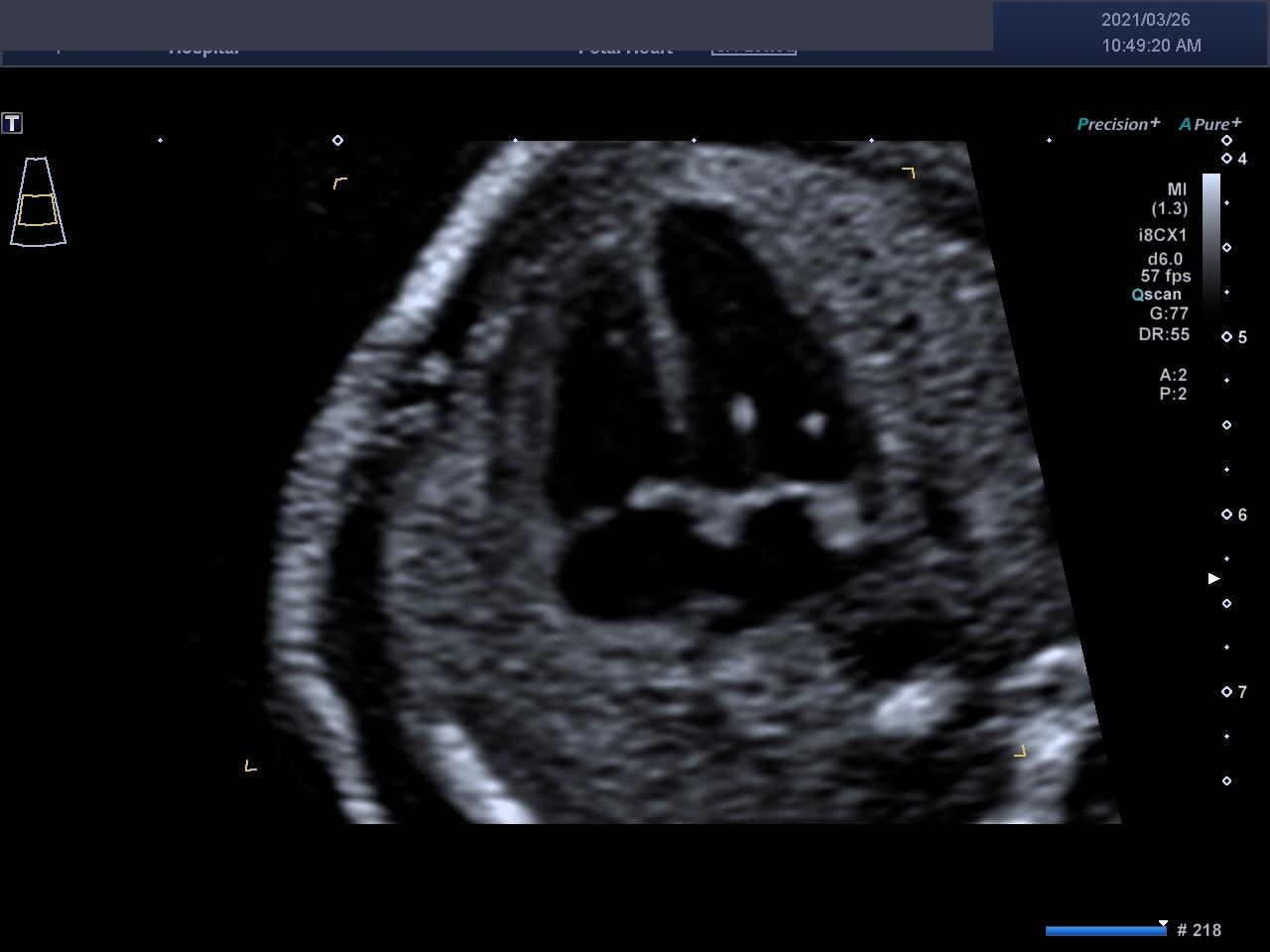

Supplement: S2 Dataset — (ZIP) [file pone.0305250.s002.zip › FE-SD-2/images/test_res/481_fc.jpg]

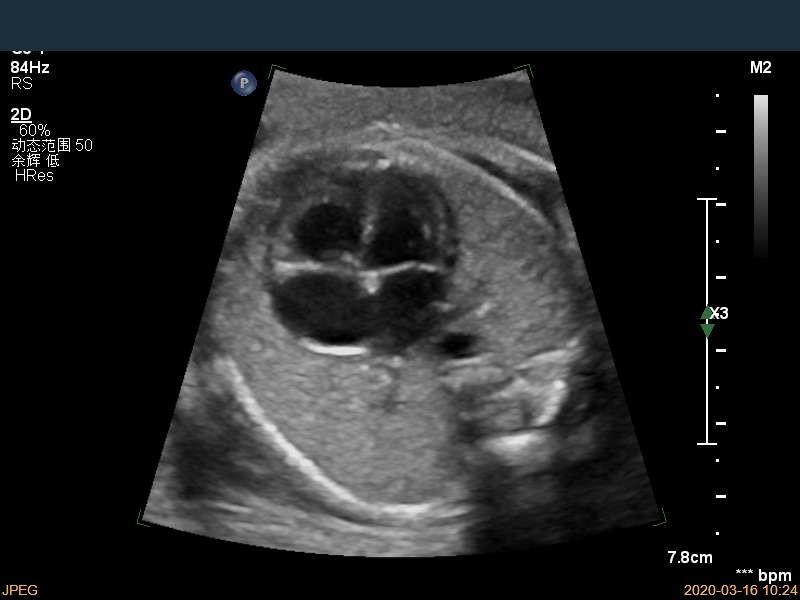

Supplement: S2 Dataset — (ZIP) [file pone.0305250.s002.zip › FE-SD-2/images/test_res/484_fc.jpg]

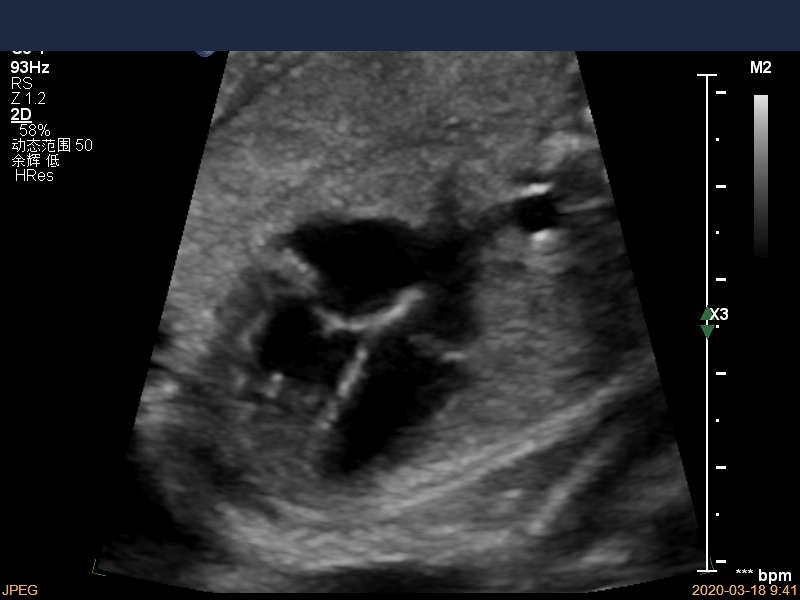

Supplement: S2 Dataset — (ZIP) [file pone.0305250.s002.zip › FE-SD-2/images/test_res/485_fc.jpg]

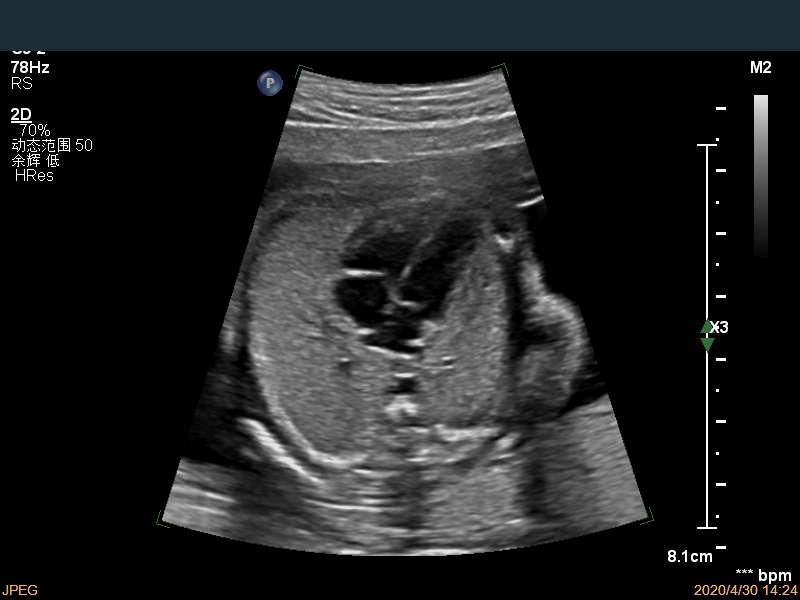

Supplement: S2 Dataset — (ZIP) [file pone.0305250.s002.zip › FE-SD-2/images/test_res/490_fc.jpg]

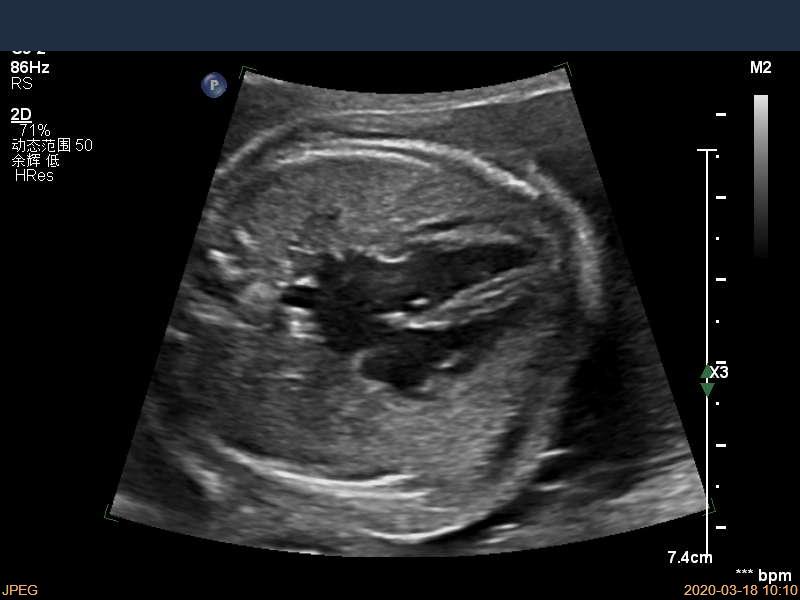

Supplement: S2 Dataset — (ZIP) [file pone.0305250.s002.zip › FE-SD-2/images/test_res/491_fc.jpg]

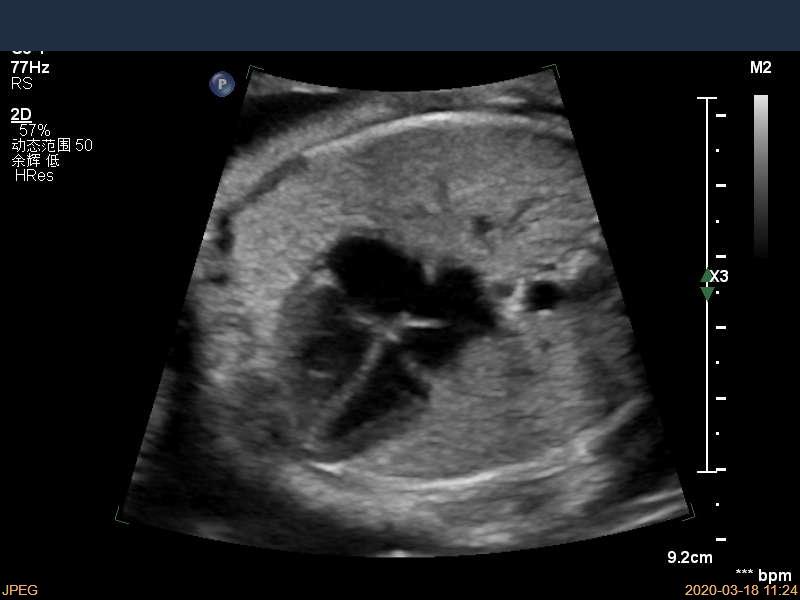

Supplement: S2 Dataset — (ZIP) [file pone.0305250.s002.zip › FE-SD-2/images/test_res/493_fc.jpg]

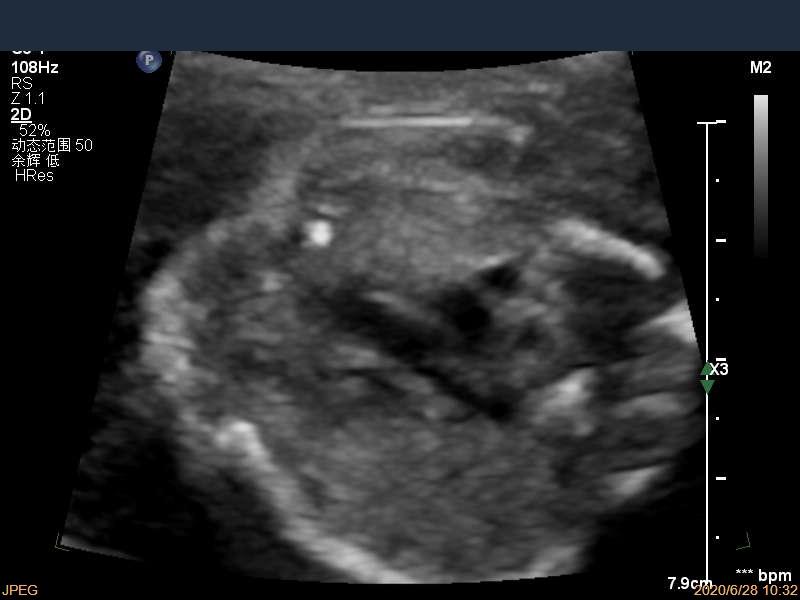

Supplement: S2 Dataset — (ZIP) [file pone.0305250.s002.zip › FE-SD-2/images/test_res/496_tv.jpg]

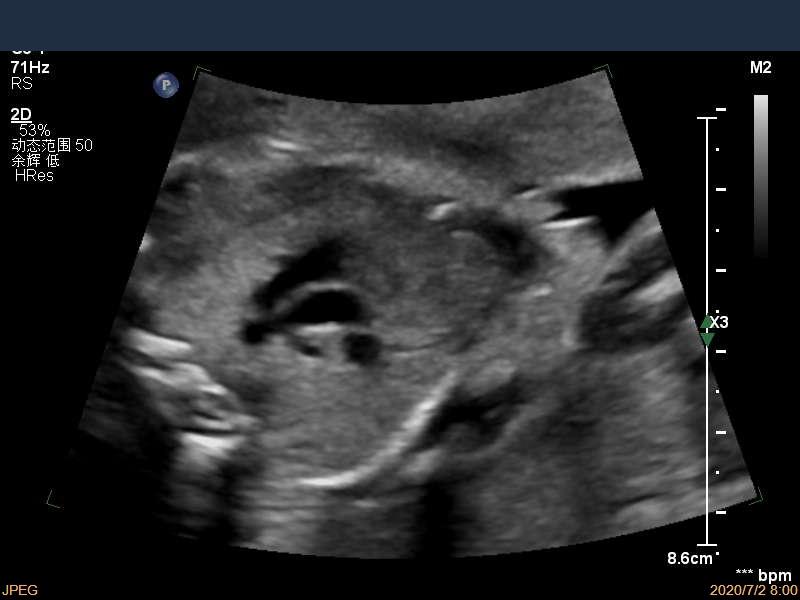

Supplement: S2 Dataset — (ZIP) [file pone.0305250.s002.zip › FE-SD-2/images/test_res/507_tv.jpg]

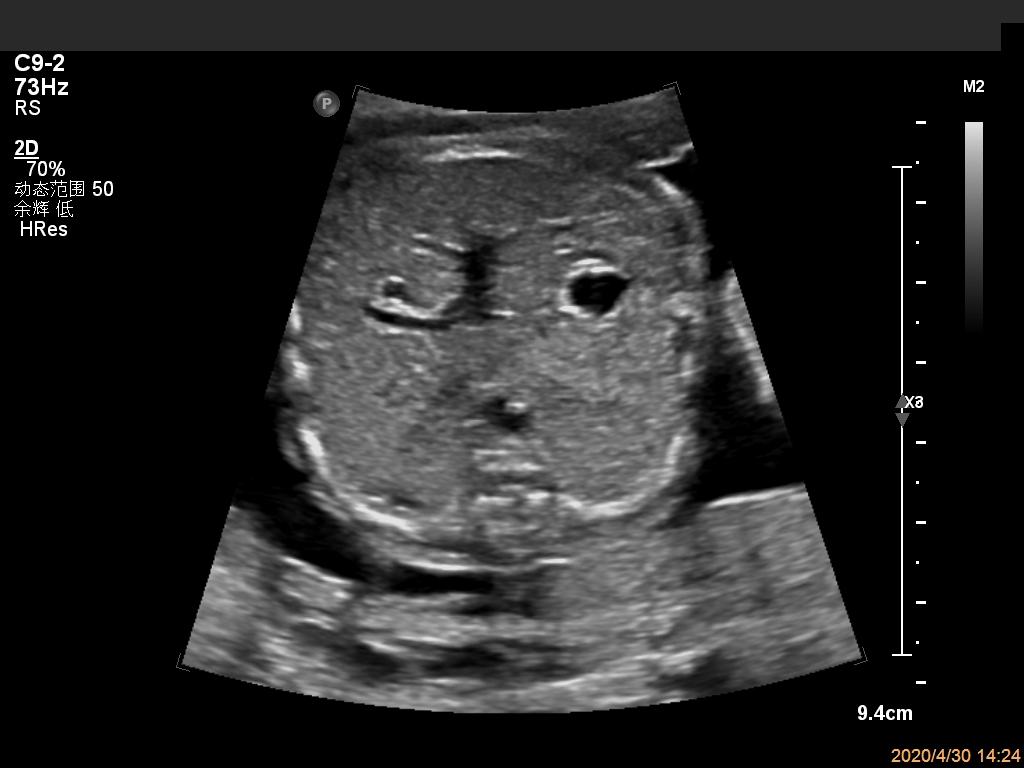

Supplement: S2 Dataset — (ZIP) [file pone.0305250.s002.zip › FE-SD-2/images/test_res/512_ab.jpg]

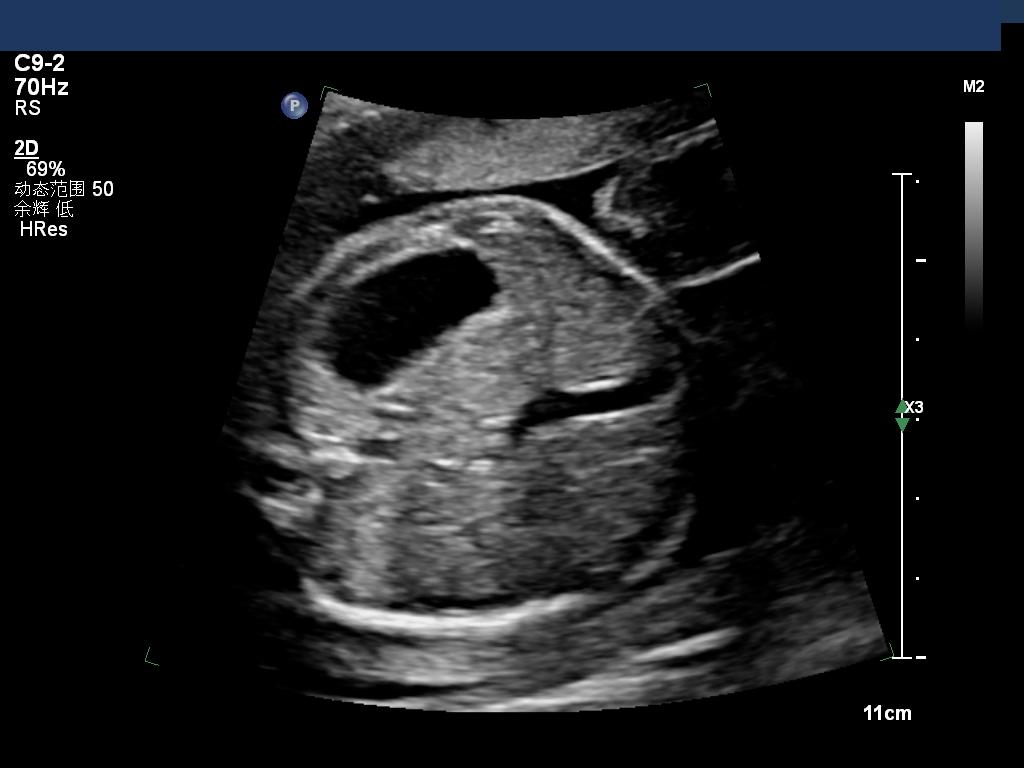

Supplement: S2 Dataset — (ZIP) [file pone.0305250.s002.zip › FE-SD-2/images/test_res/513_ab.jpg]

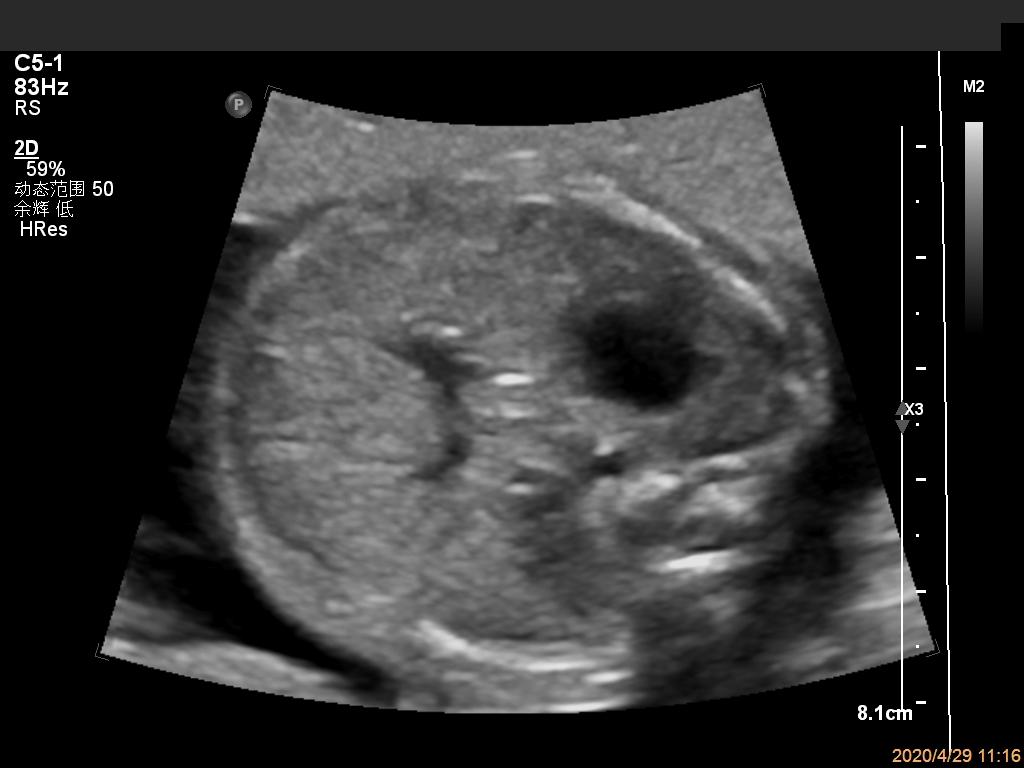

Supplement: S2 Dataset — (ZIP) [file pone.0305250.s002.zip › FE-SD-2/images/test_res/514_ab.jpg]

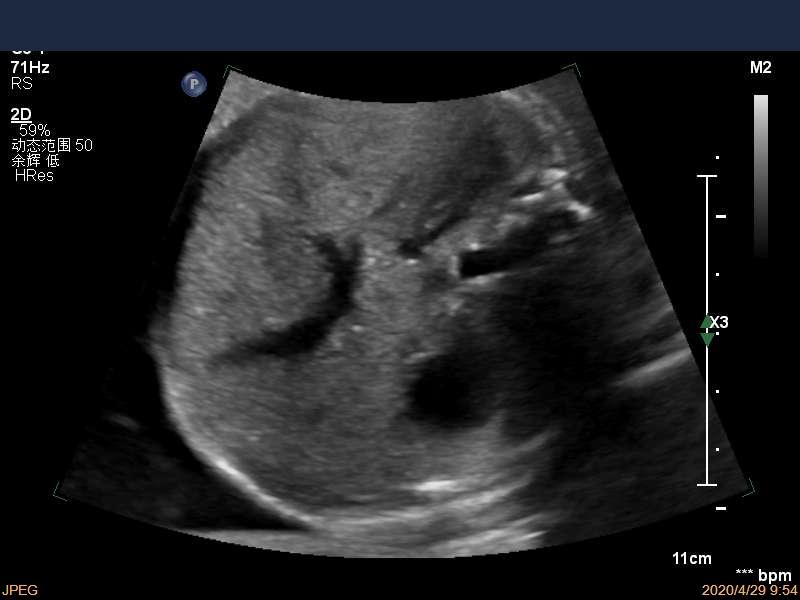

Supplement: S2 Dataset — (ZIP) [file pone.0305250.s002.zip › FE-SD-2/images/test_res/516_ab.jpg]

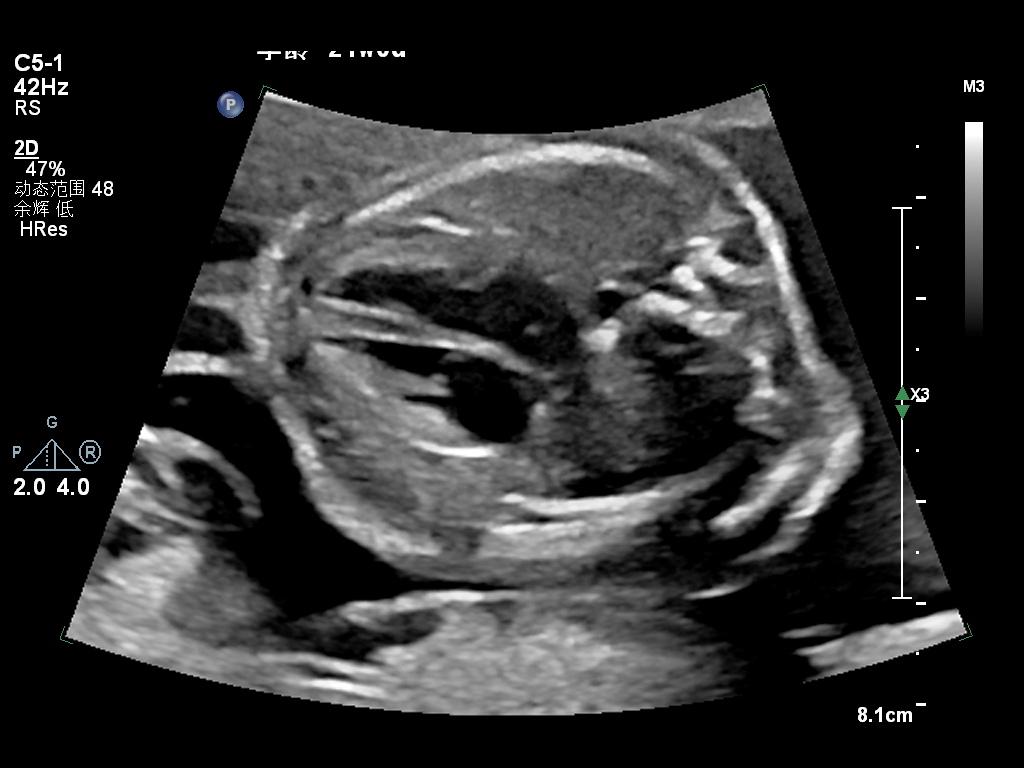

Supplement: S2 Dataset — (ZIP) [file pone.0305250.s002.zip › FE-SD-2/images/test_res/516_fc.jpg]

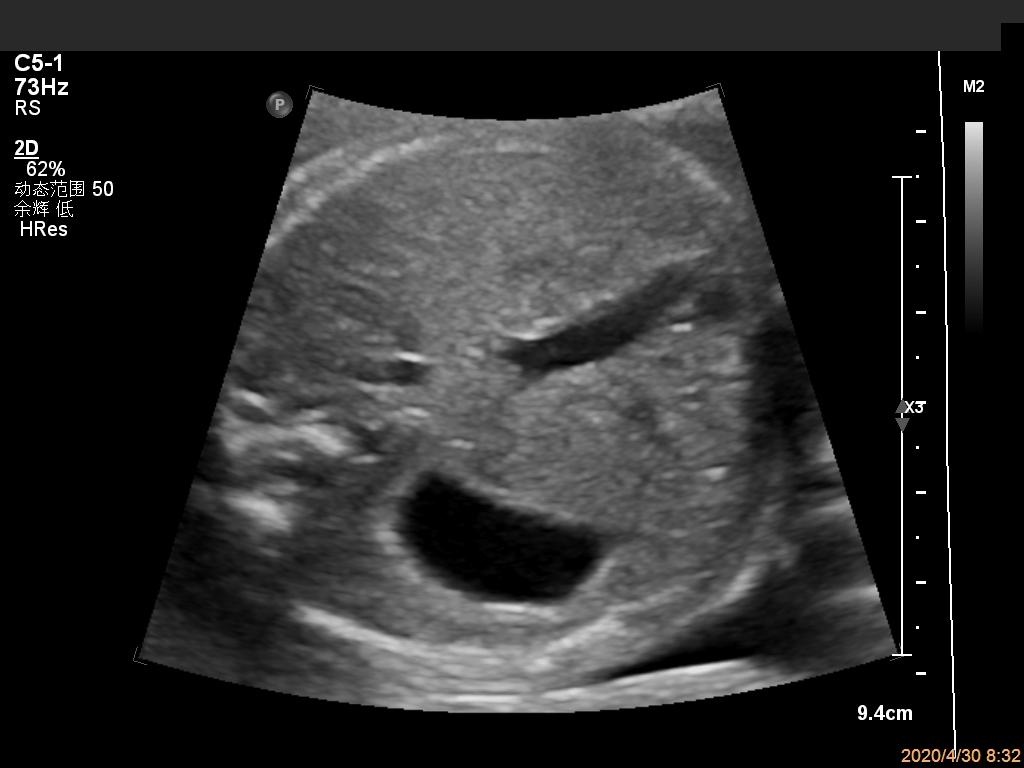

Supplement: S2 Dataset — (ZIP) [file pone.0305250.s002.zip › FE-SD-2/images/test_res/518_ab.jpg]

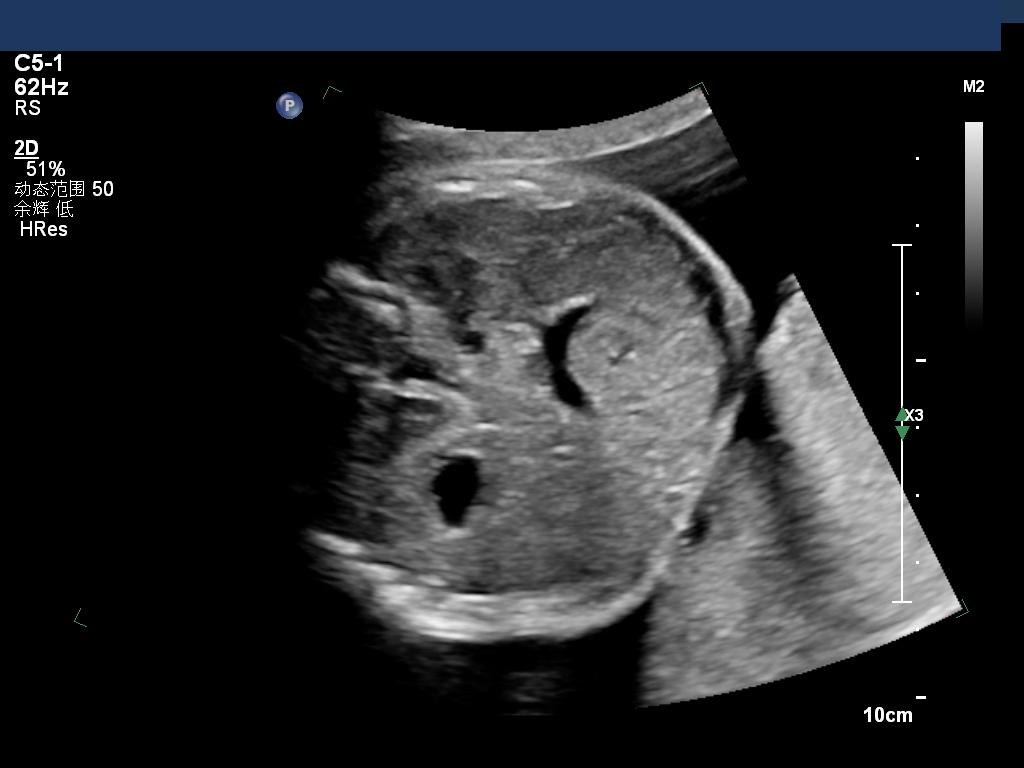

Supplement: S2 Dataset — (ZIP) [file pone.0305250.s002.zip › FE-SD-2/images/test_res/520_ab.jpg]

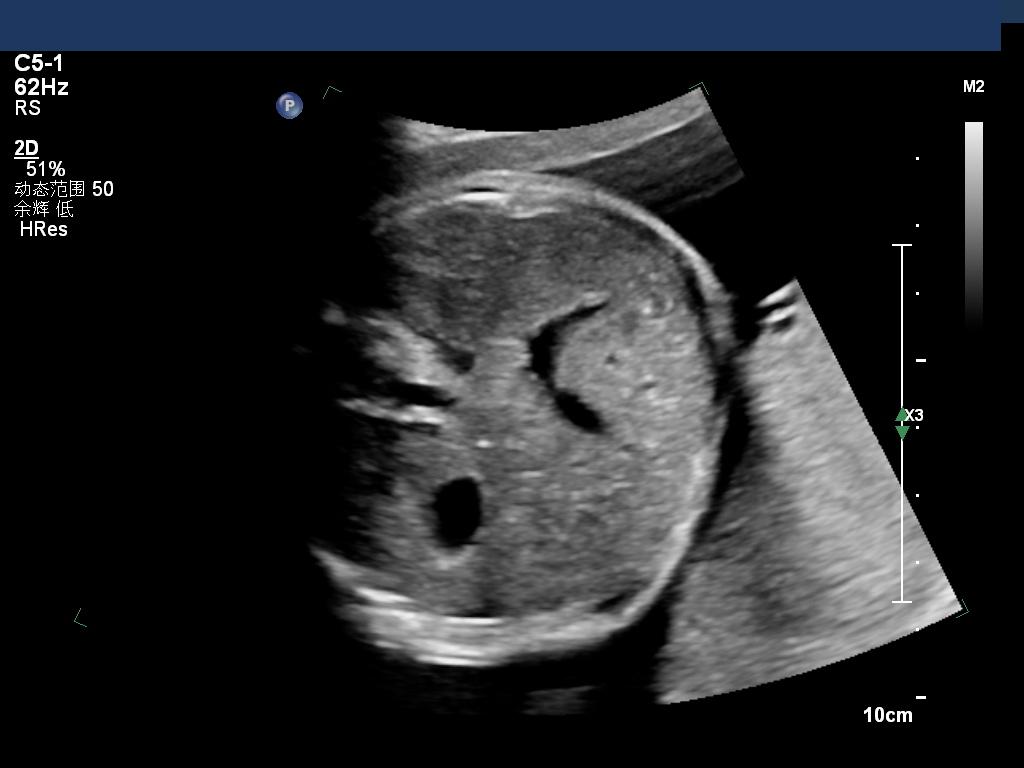

Supplement: S2 Dataset — (ZIP) [file pone.0305250.s002.zip › FE-SD-2/images/test_res/521_ab.jpg]

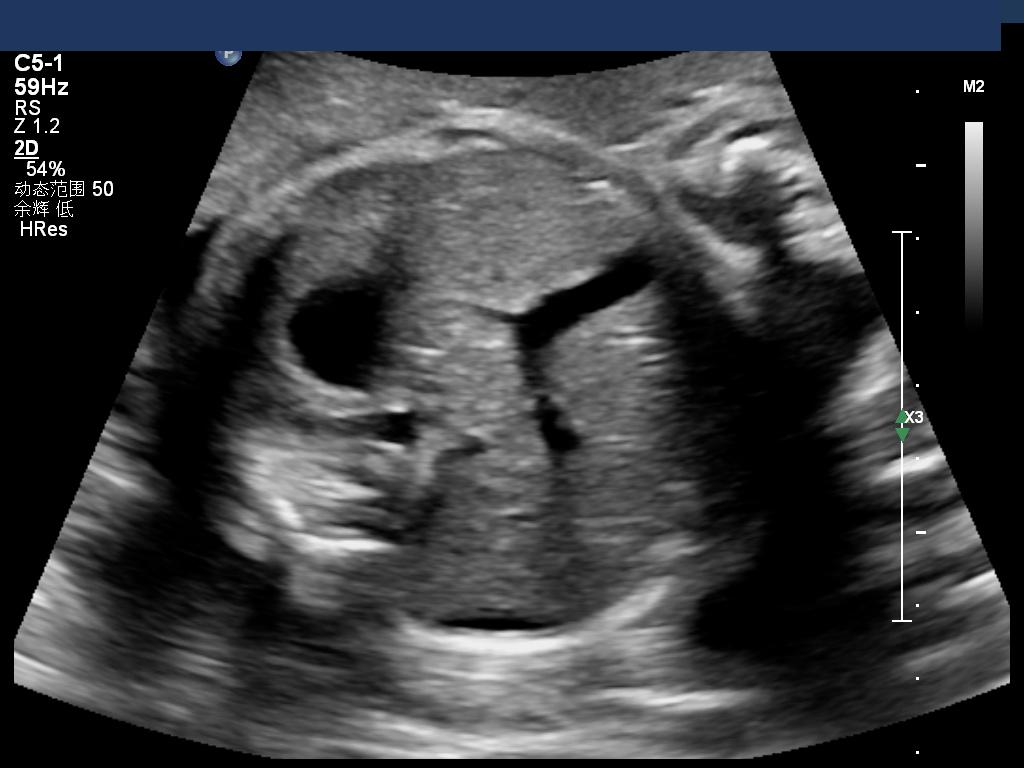

Supplement: S2 Dataset — (ZIP) [file pone.0305250.s002.zip › FE-SD-2/images/test_res/523_ab.jpg]

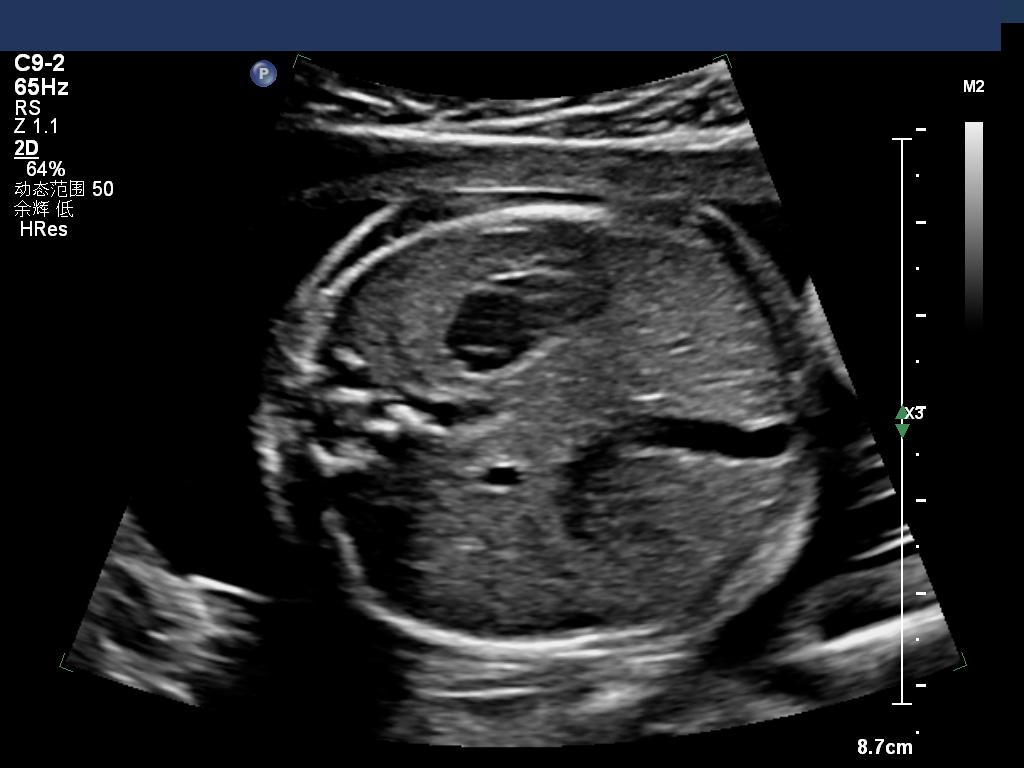

Supplement: S2 Dataset — (ZIP) [file pone.0305250.s002.zip › FE-SD-2/images/test_res/524_ab.jpg]

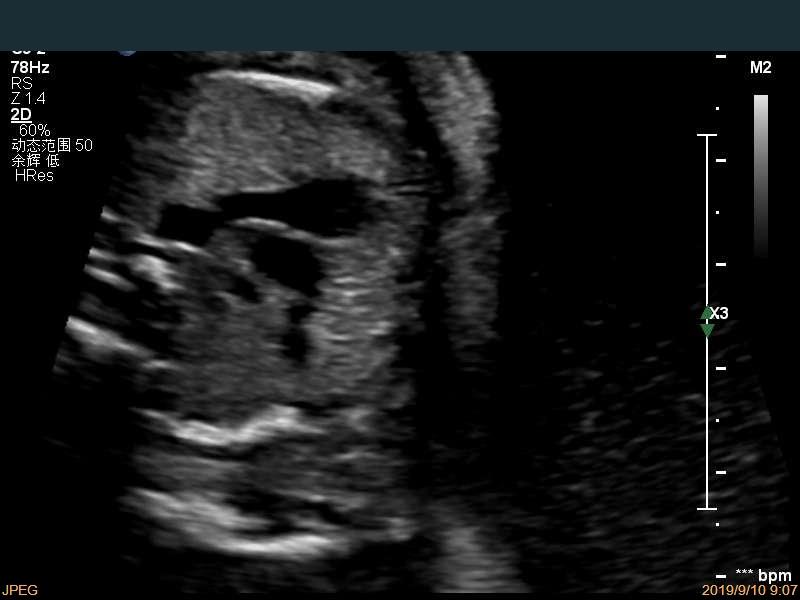

Supplement: S2 Dataset — (ZIP) [file pone.0305250.s002.zip › FE-SD-2/images/test_res/532_tv.jpg]

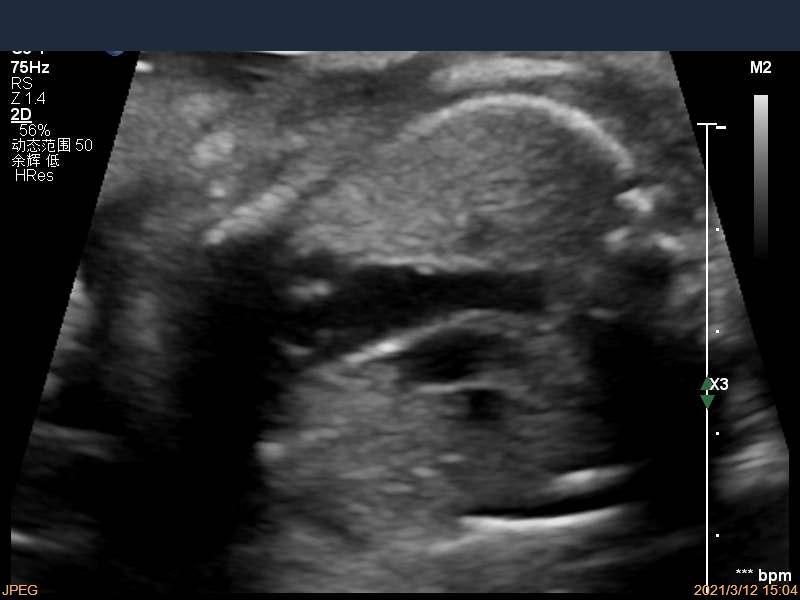

Supplement: S2 Dataset — (ZIP) [file pone.0305250.s002.zip › FE-SD-2/images/test_res/533_tv.jpg]

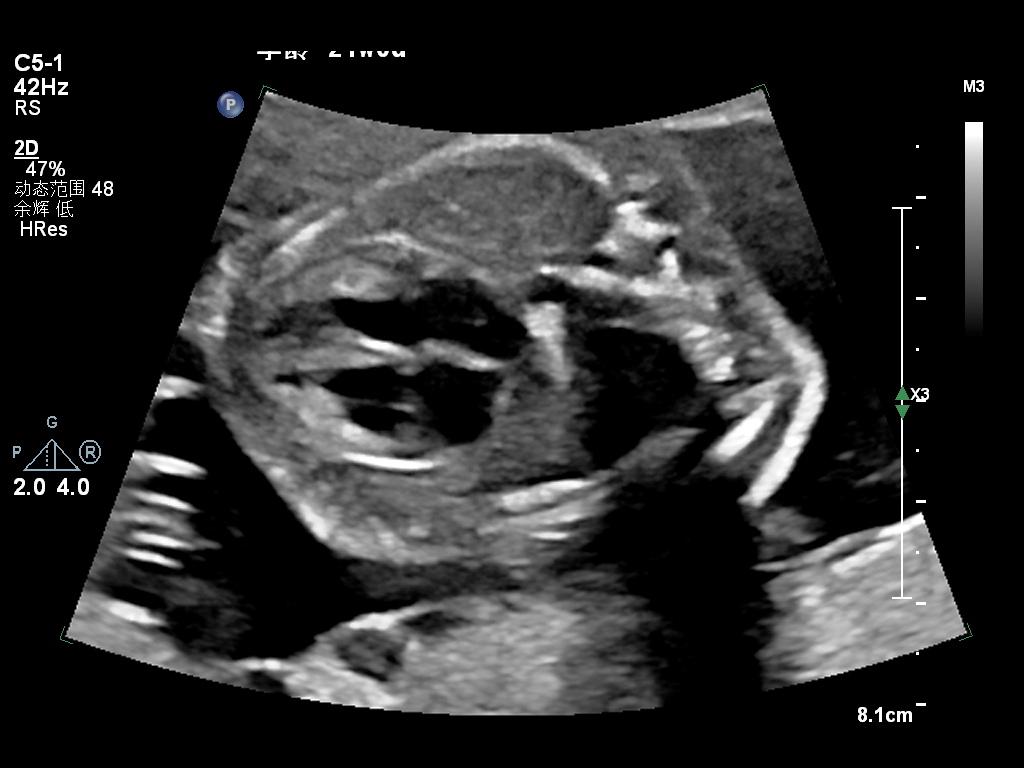

Supplement: S2 Dataset — (ZIP) [file pone.0305250.s002.zip › FE-SD-2/images/test_res/537_fc.jpg]

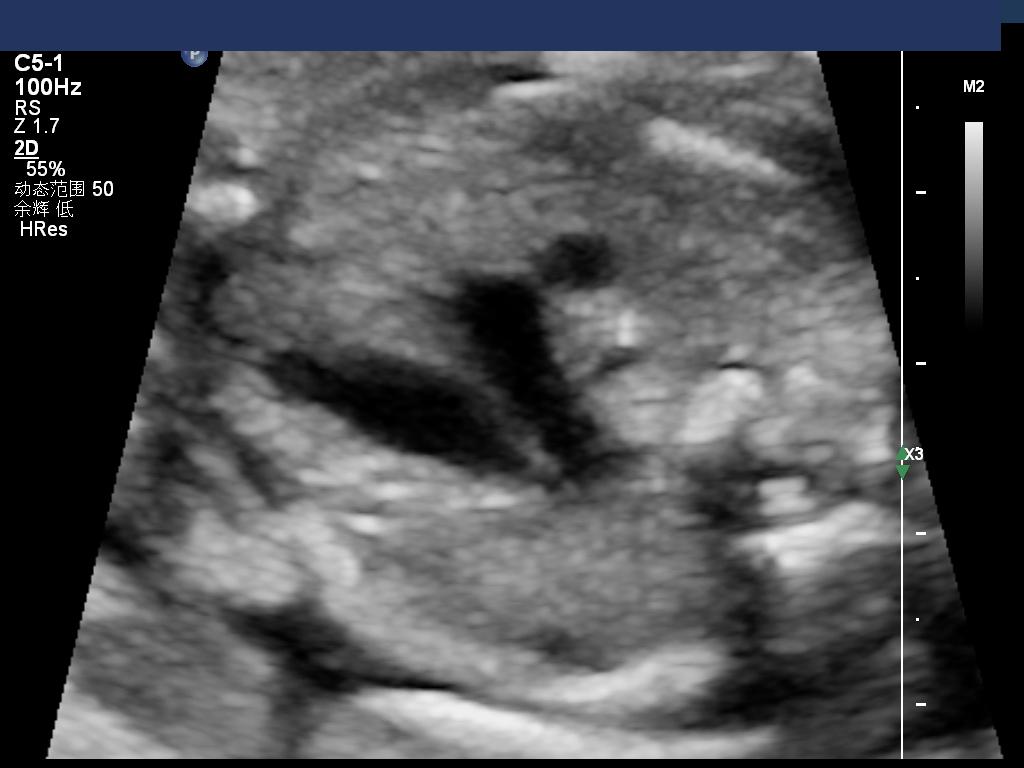

Supplement: S2 Dataset — (ZIP) [file pone.0305250.s002.zip › FE-SD-2/images/test_res/541_tv.jpg]

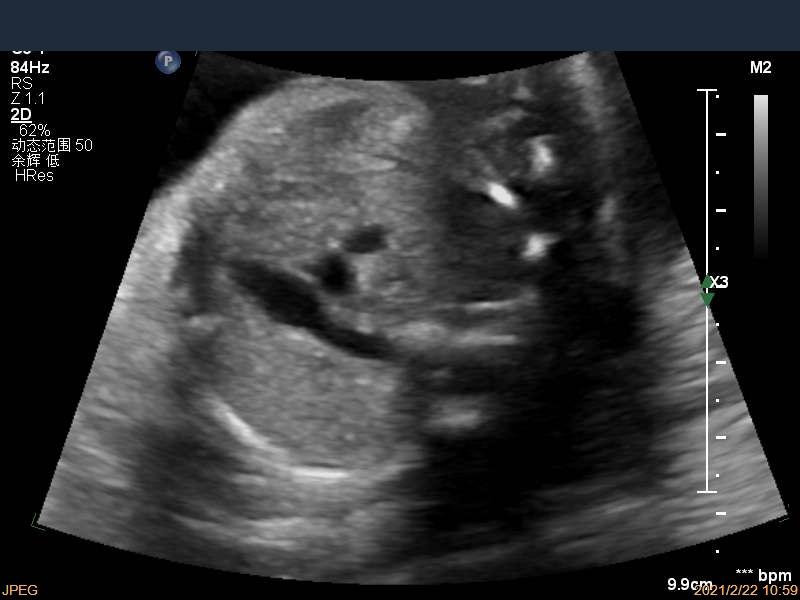

Supplement: S2 Dataset — (ZIP) [file pone.0305250.s002.zip › FE-SD-2/images/test_res/544_tv.jpg]

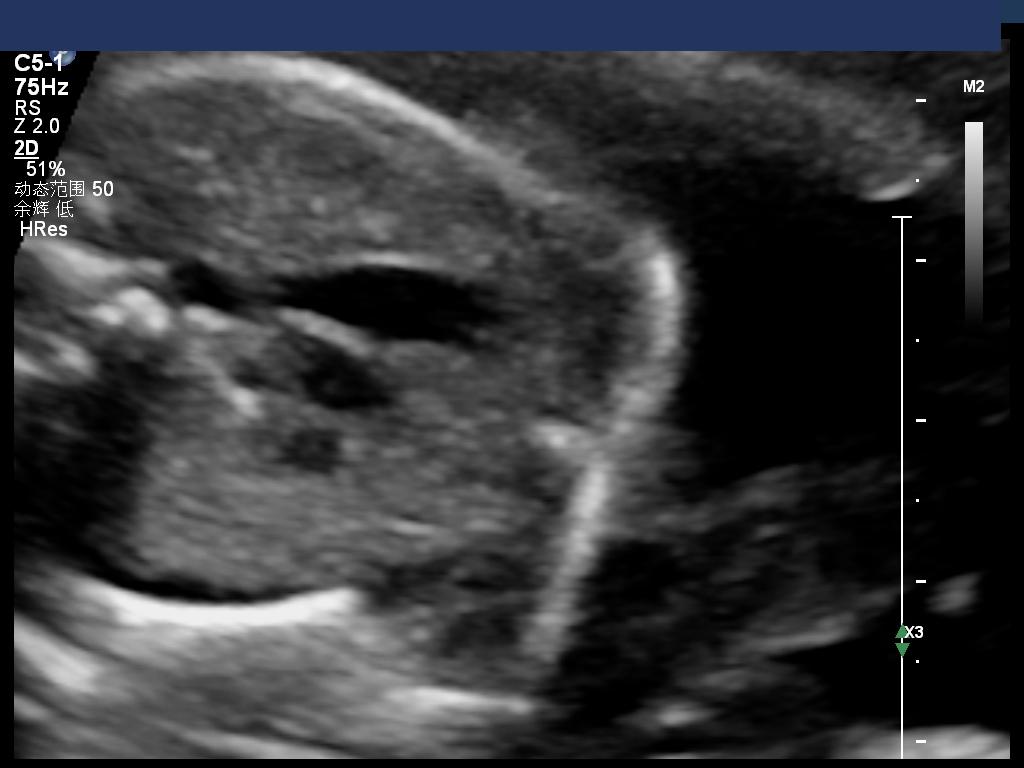

Supplement: S2 Dataset — (ZIP) [file pone.0305250.s002.zip › FE-SD-2/images/test_res/553_tv.jpg]

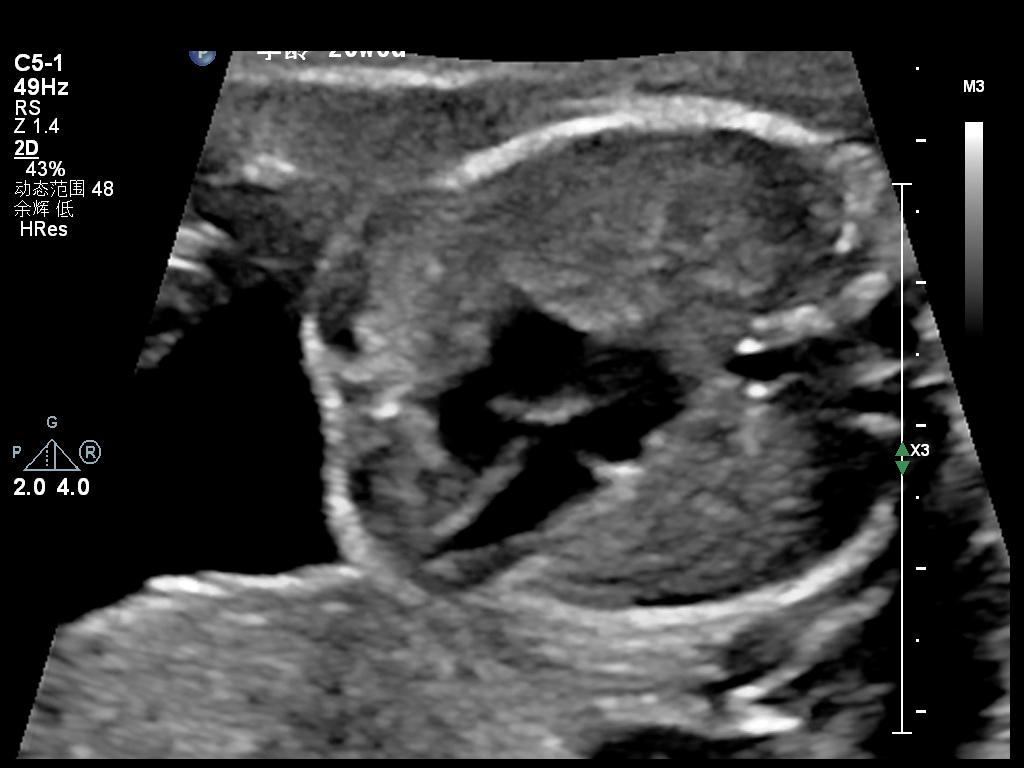

Supplement: S2 Dataset — (ZIP) [file pone.0305250.s002.zip › FE-SD-2/images/test_res/554_fc.jpg]

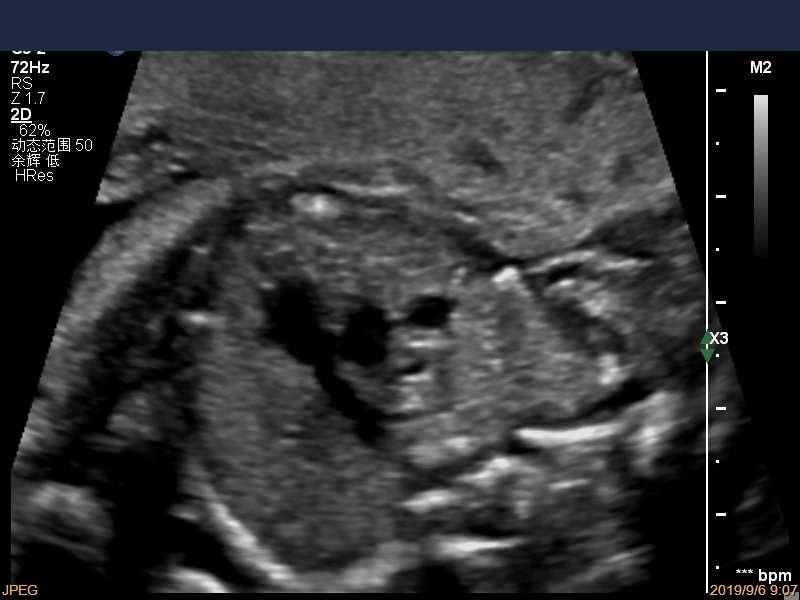

Supplement: S2 Dataset — (ZIP) [file pone.0305250.s002.zip › FE-SD-2/images/test_res/570_tv.jpg]

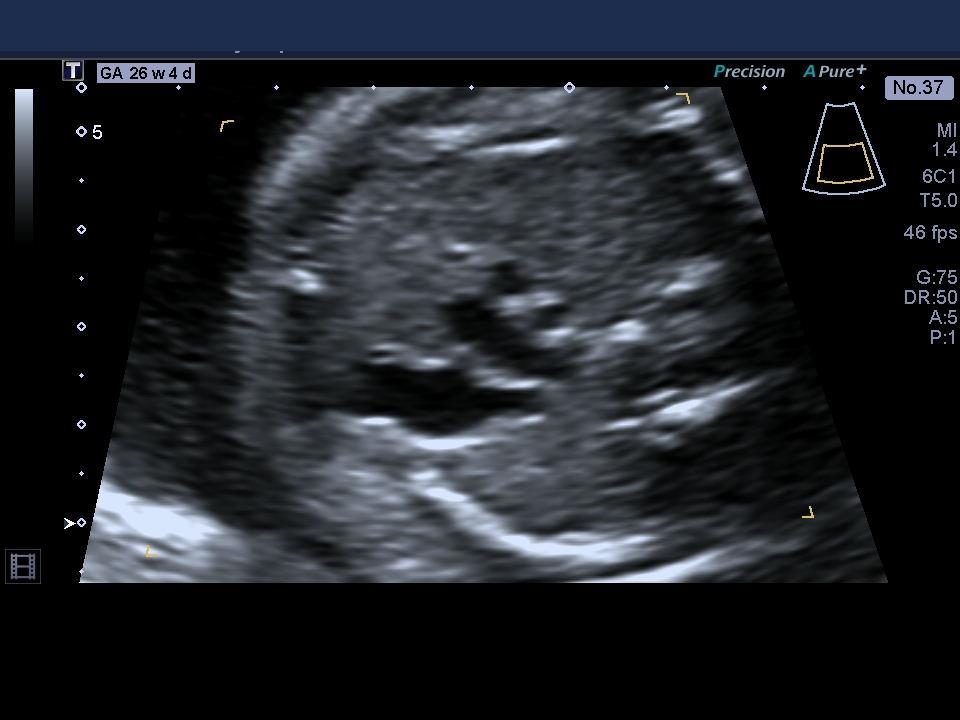

Supplement: S2 Dataset — (ZIP) [file pone.0305250.s002.zip › FE-SD-2/images/test_res/572_tv.jpg]

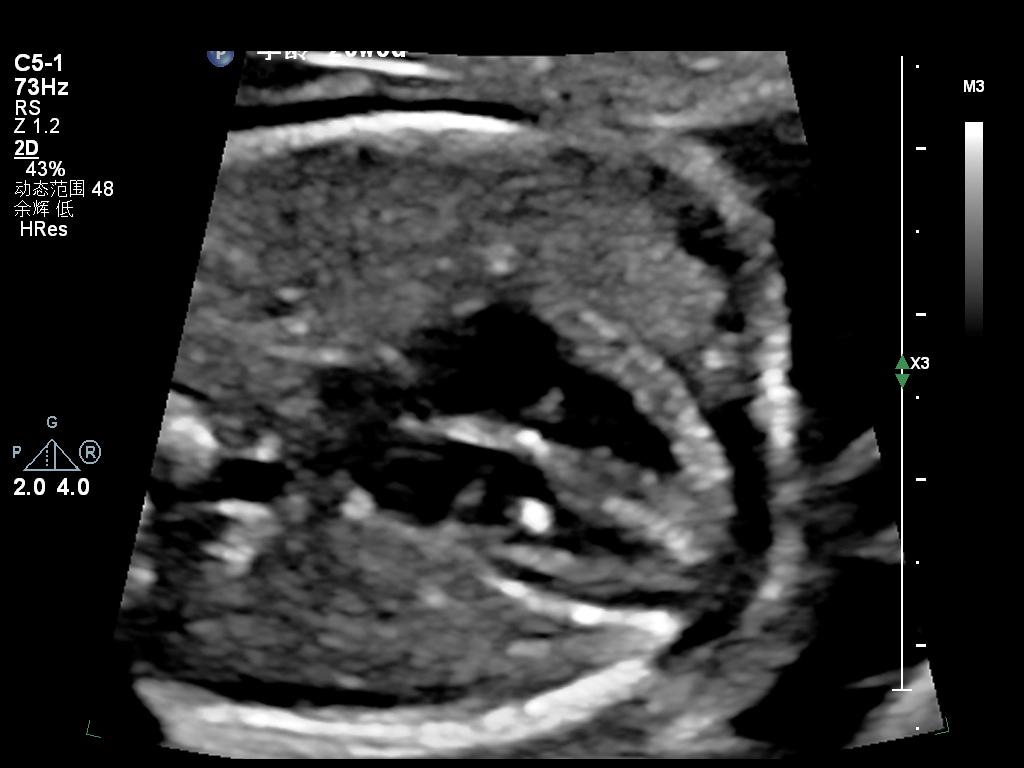

Supplement: S2 Dataset — (ZIP) [file pone.0305250.s002.zip › FE-SD-2/images/test_res/578_fc.jpg]

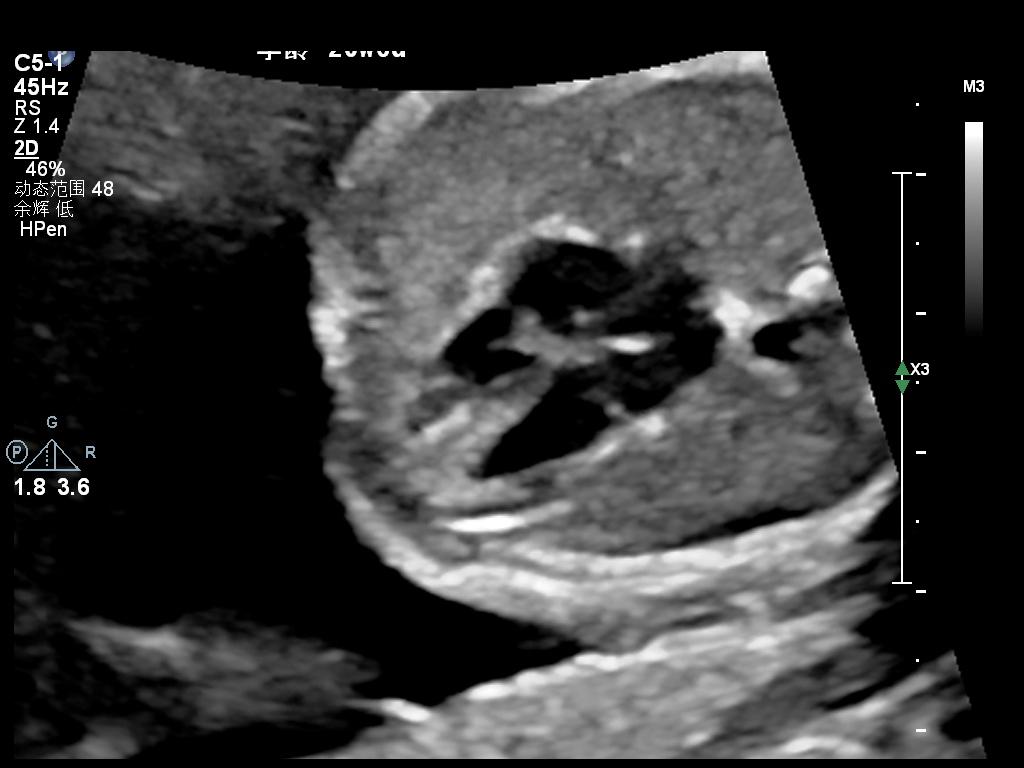

Supplement: S2 Dataset — (ZIP) [file pone.0305250.s002.zip › FE-SD-2/images/test_res/583_fc.jpg]

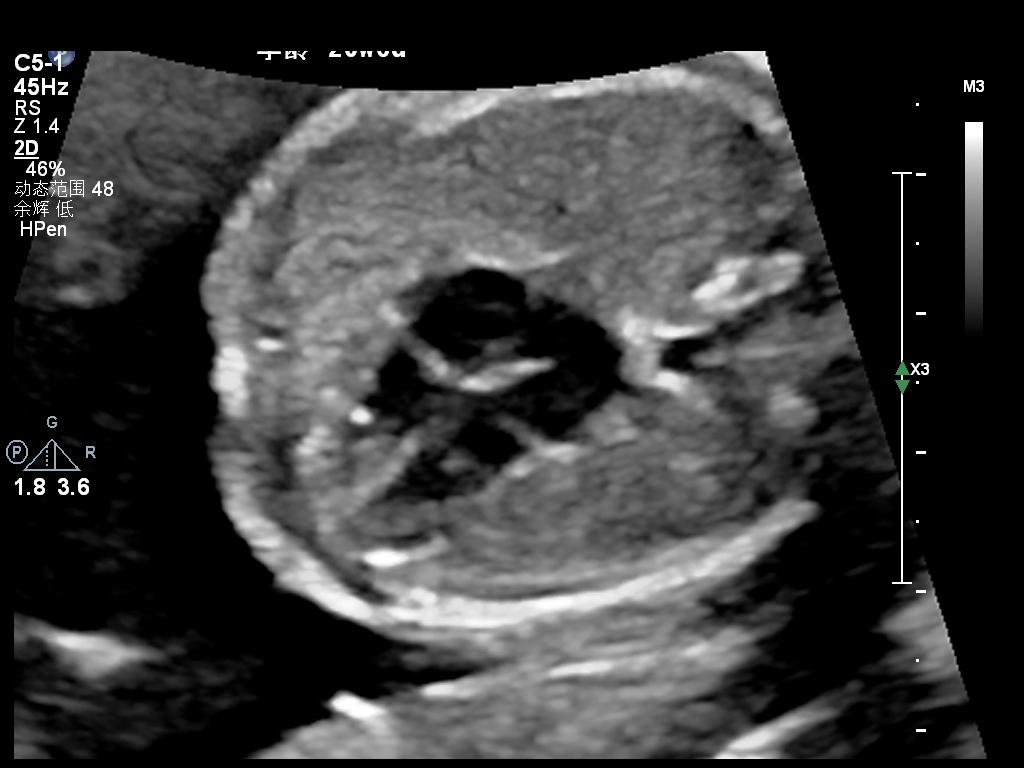

Supplement: S2 Dataset — (ZIP) [file pone.0305250.s002.zip › FE-SD-2/images/test_res/584_fc.jpg]

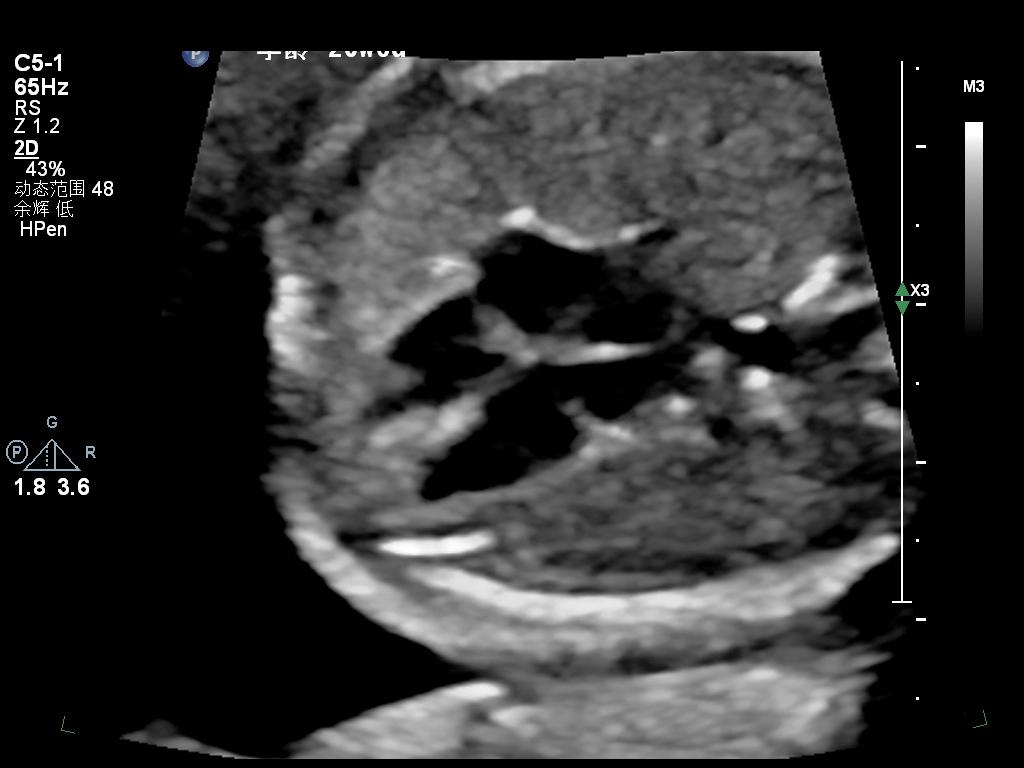

Supplement: S2 Dataset — (ZIP) [file pone.0305250.s002.zip › FE-SD-2/images/test_res/585_fc.jpg]

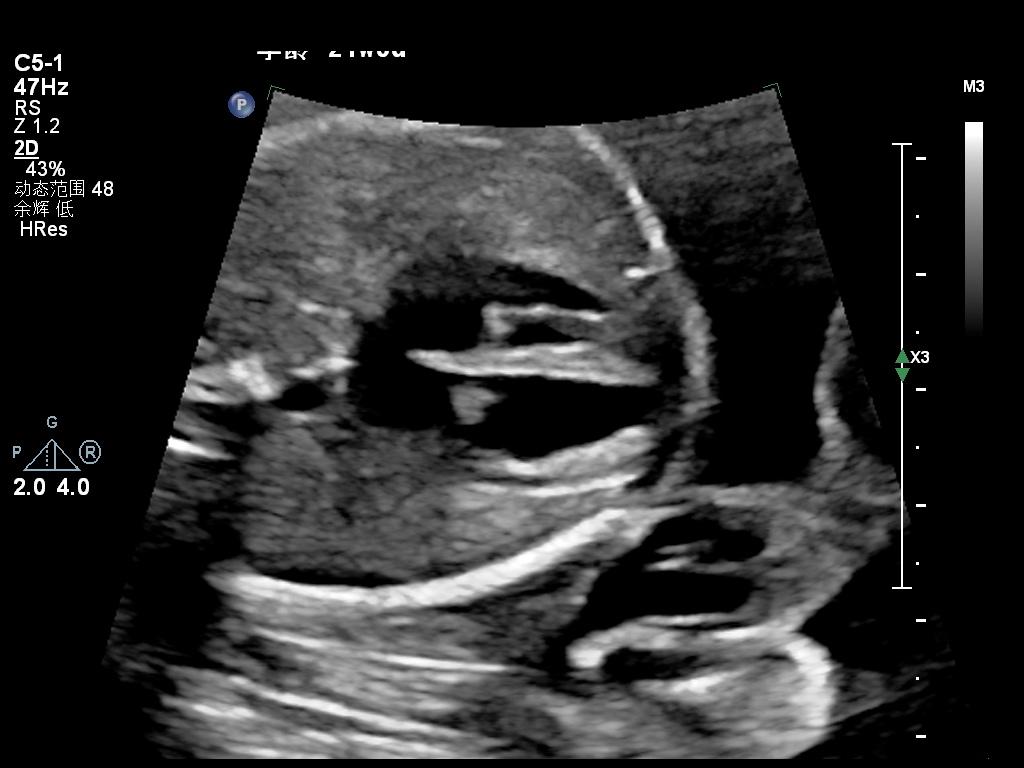

Supplement: S2 Dataset — (ZIP) [file pone.0305250.s002.zip › FE-SD-2/images/test_res/587_fc.jpg]

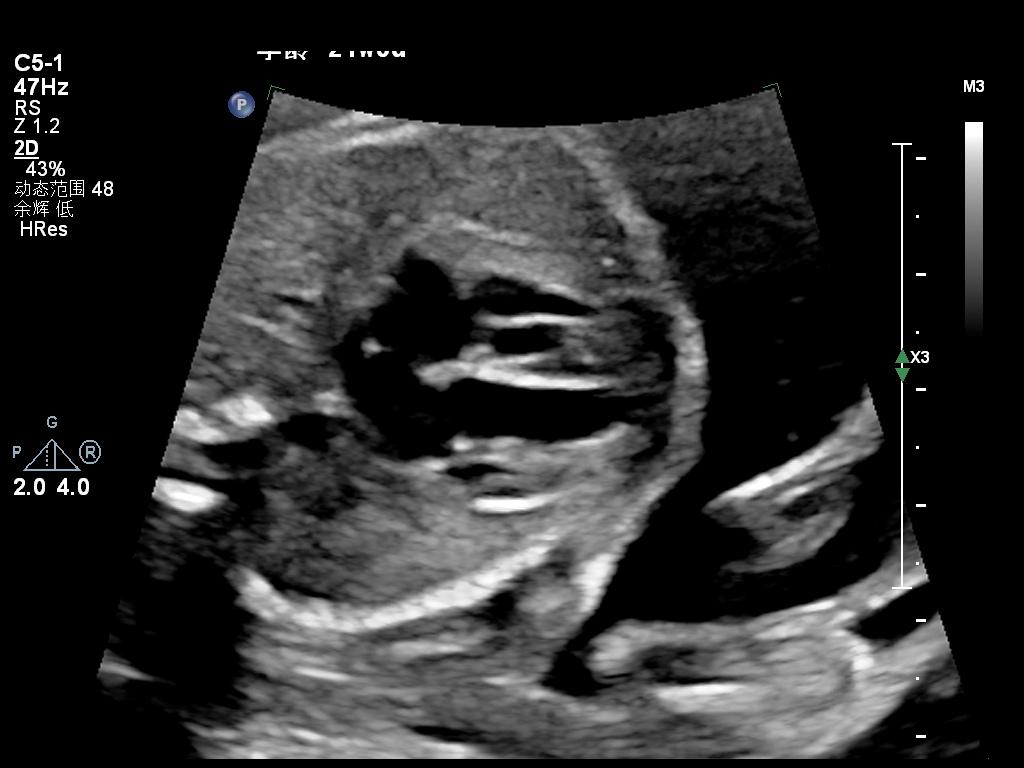

Supplement: S2 Dataset — (ZIP) [file pone.0305250.s002.zip › FE-SD-2/images/test_res/589_fc.jpg]

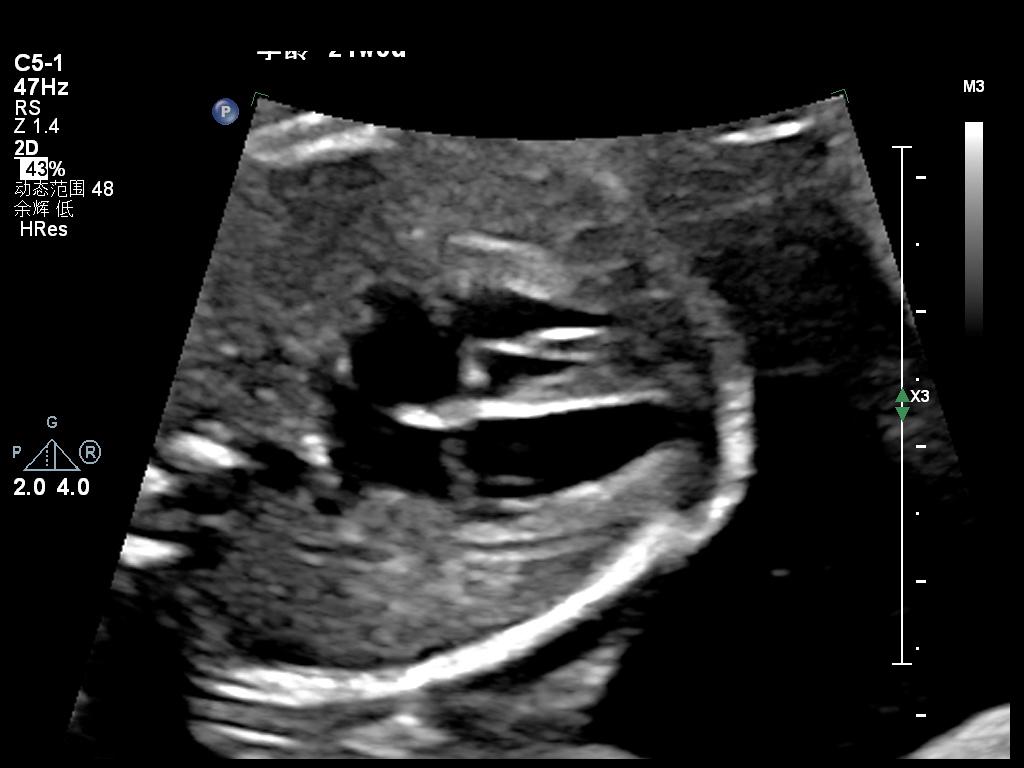

Supplement: S2 Dataset — (ZIP) [file pone.0305250.s002.zip › FE-SD-2/images/test_res/590_fc.jpg]

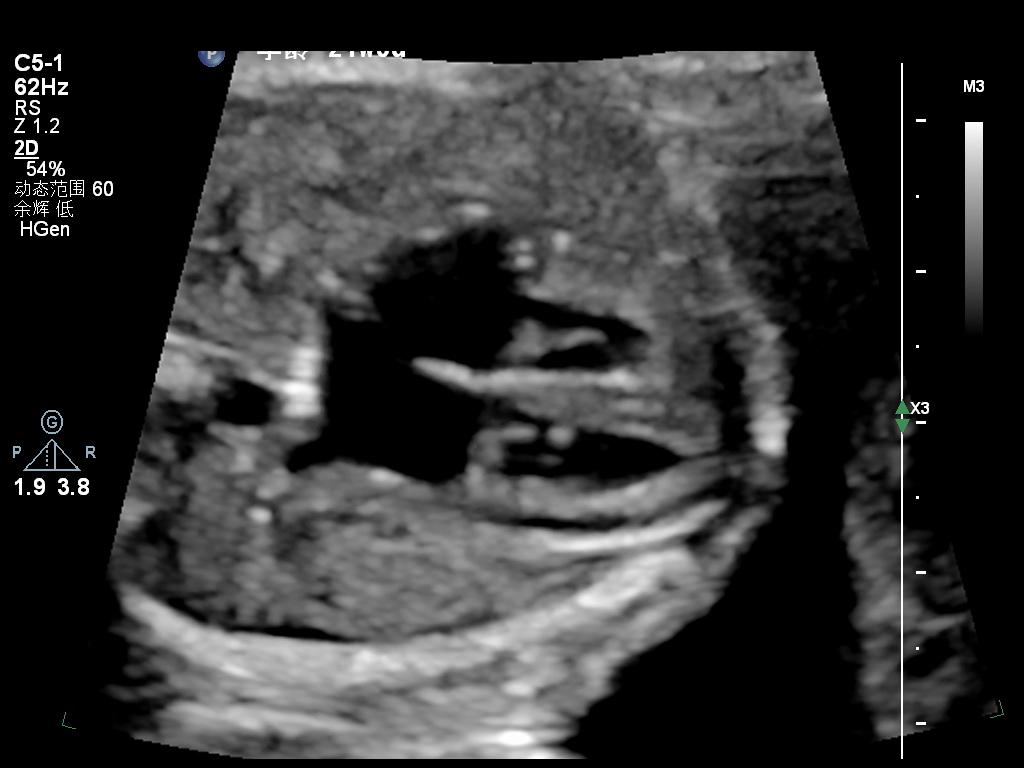

Supplement: S2 Dataset — (ZIP) [file pone.0305250.s002.zip › FE-SD-2/images/test_res/591_fc.jpg]

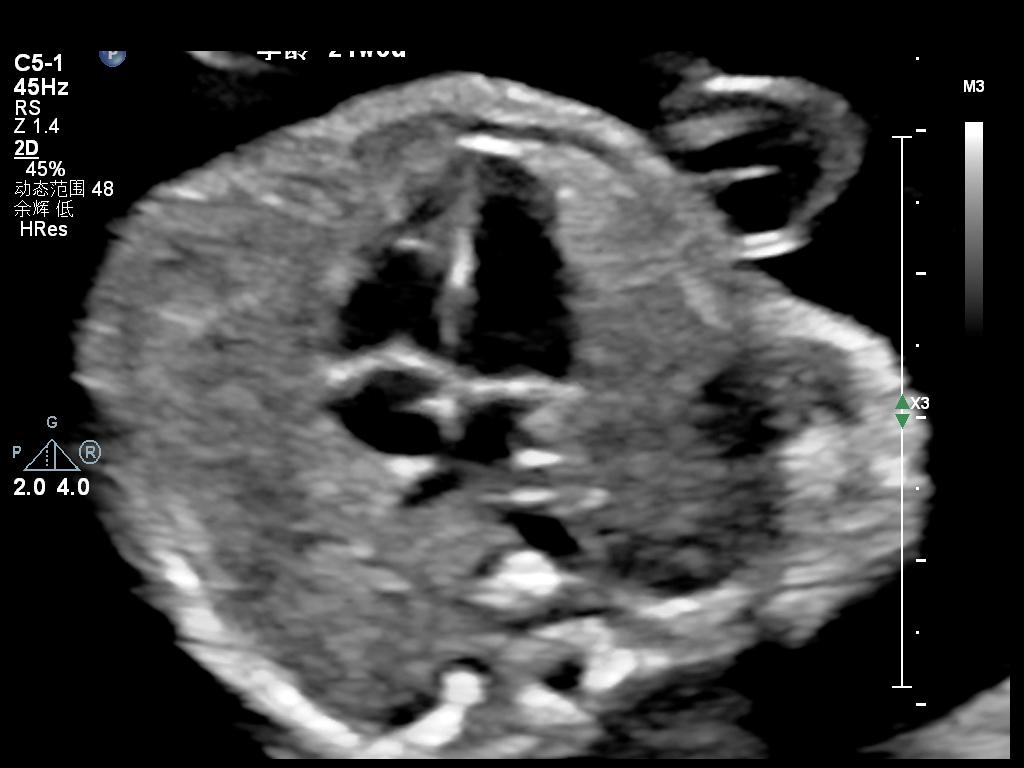

Supplement: S2 Dataset — (ZIP) [file pone.0305250.s002.zip › FE-SD-2/images/test_res/595_fc.jpg]

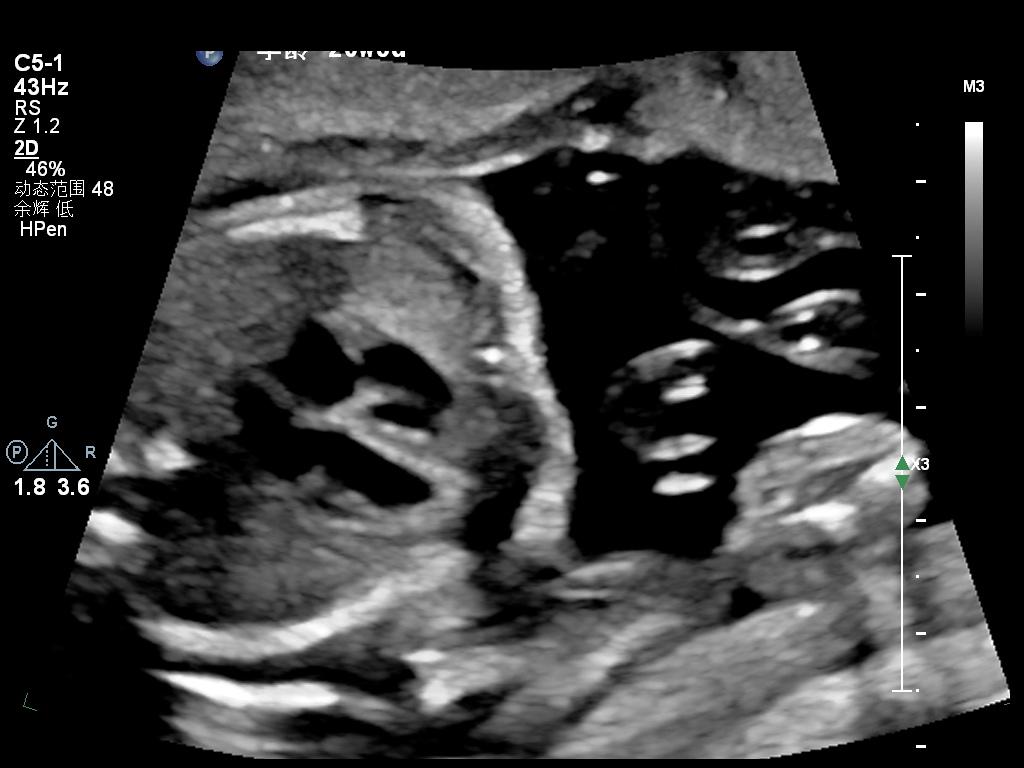

Supplement: S2 Dataset — (ZIP) [file pone.0305250.s002.zip › FE-SD-2/images/test_res/599_fc.jpg]

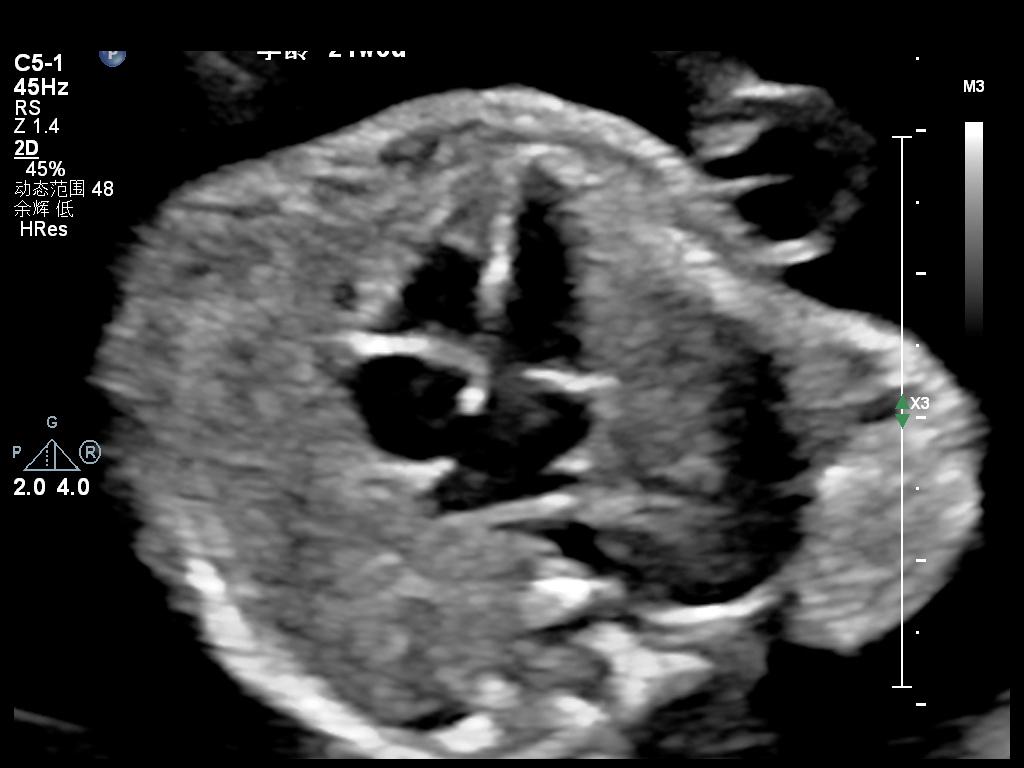

Supplement: S2 Dataset — (ZIP) [file pone.0305250.s002.zip › FE-SD-2/images/test_res/600_fc.jpg]

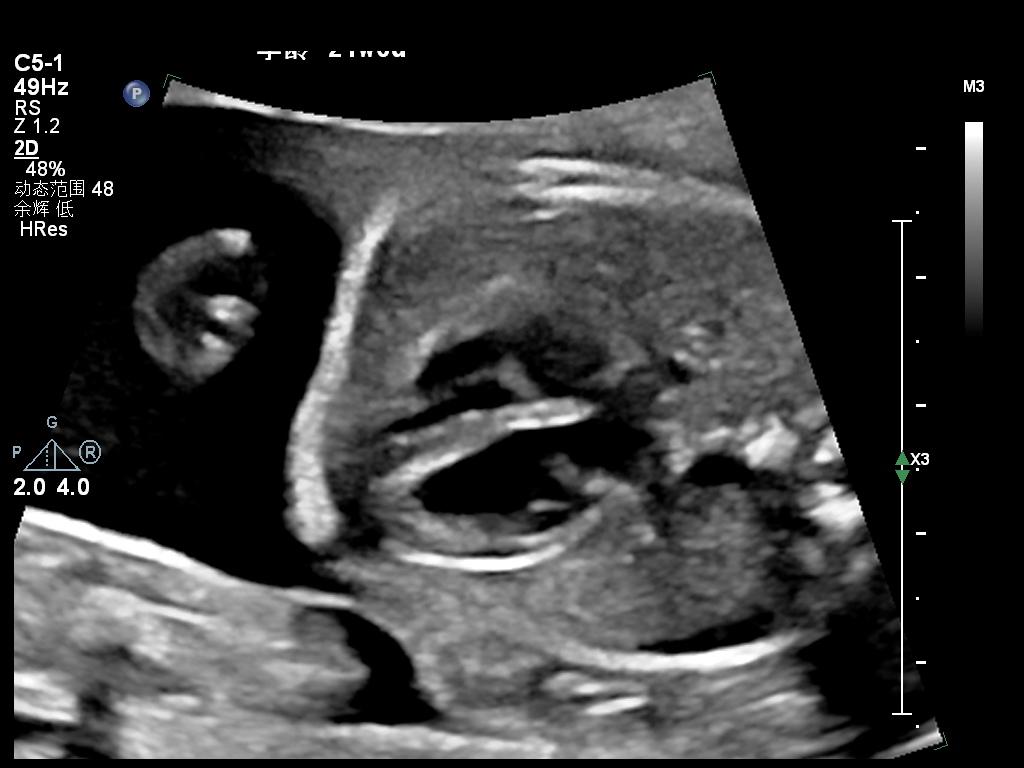

Supplement: S2 Dataset — (ZIP) [file pone.0305250.s002.zip › FE-SD-2/images/test_res/601_fc.jpg]

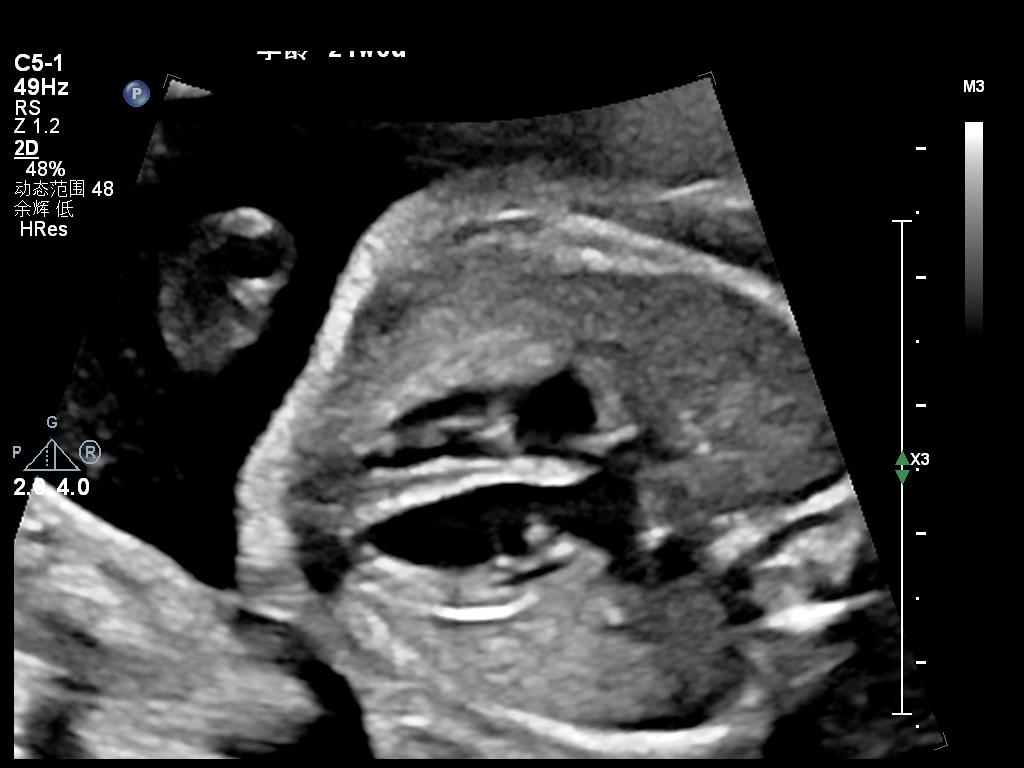

Supplement: S2 Dataset — (ZIP) [file pone.0305250.s002.zip › FE-SD-2/images/test_res/603_fc.jpg]

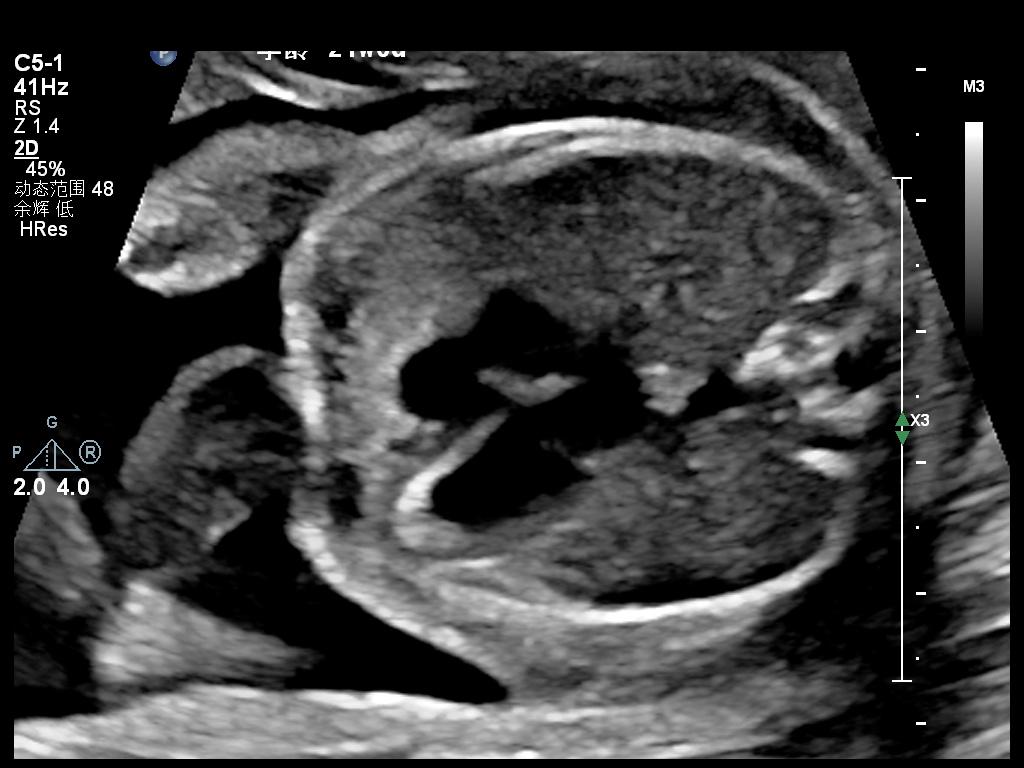

Supplement: S2 Dataset — (ZIP) [file pone.0305250.s002.zip › FE-SD-2/images/test_res/604_fc.jpg]

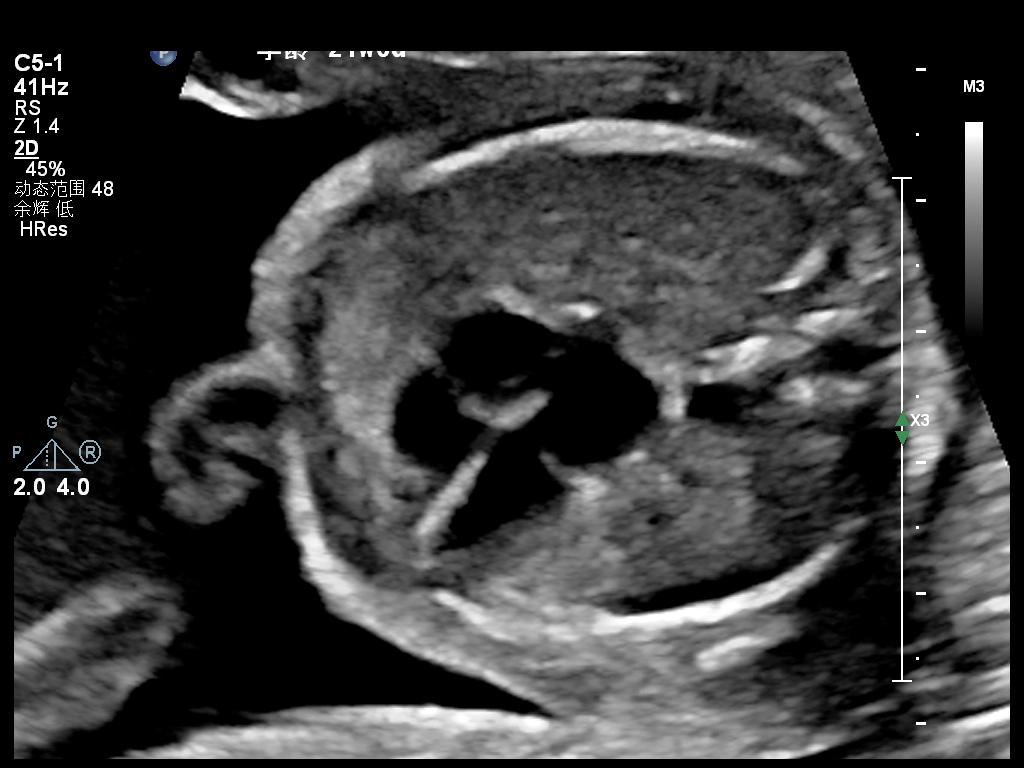

Supplement: S2 Dataset — (ZIP) [file pone.0305250.s002.zip › FE-SD-2/images/test_res/605_fc.jpg]

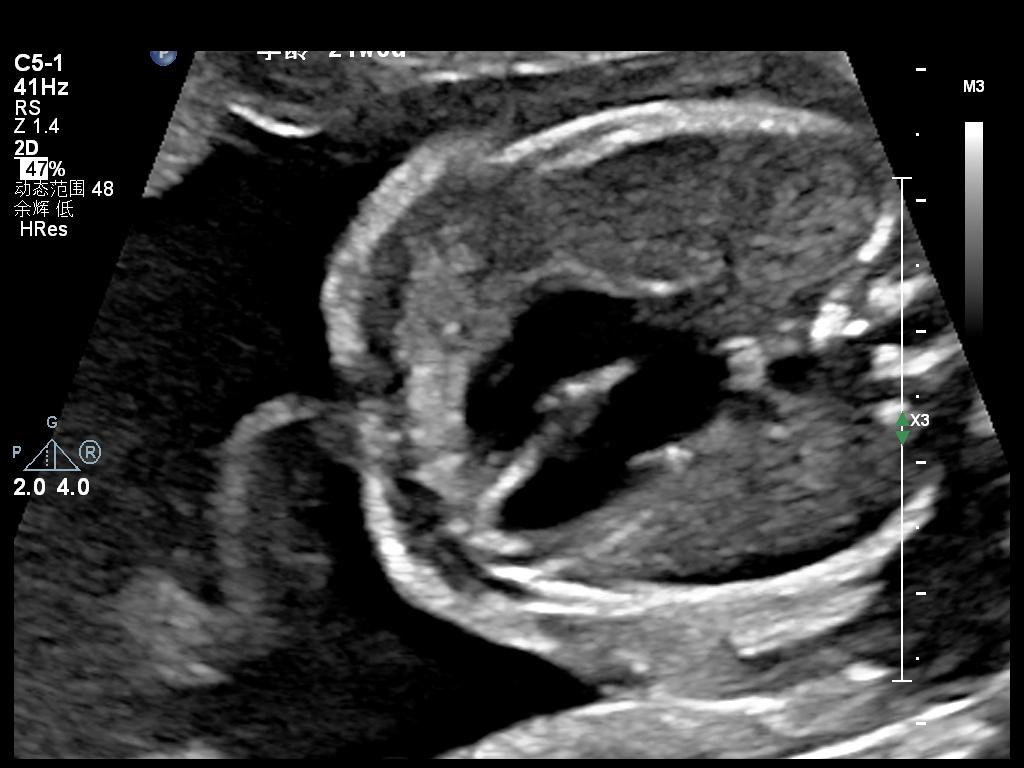

Supplement: S2 Dataset — (ZIP) [file pone.0305250.s002.zip › FE-SD-2/images/test_res/606_fc.jpg]

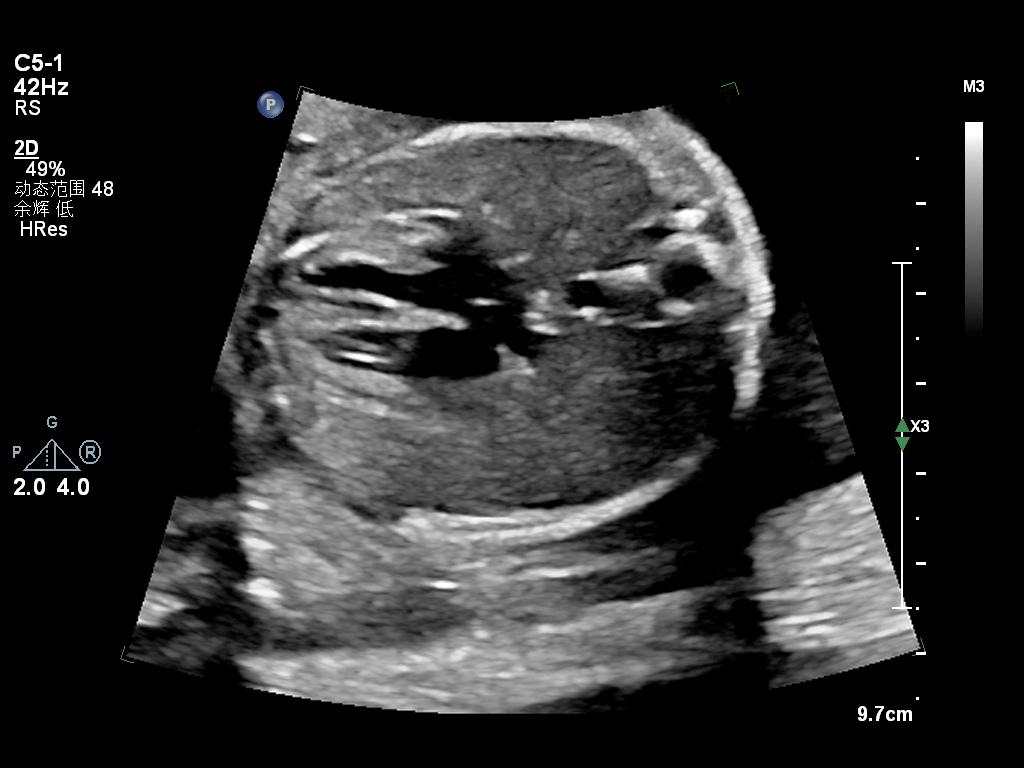

Supplement: S2 Dataset — (ZIP) [file pone.0305250.s002.zip › FE-SD-2/images/test_res/608_fc.jpg]

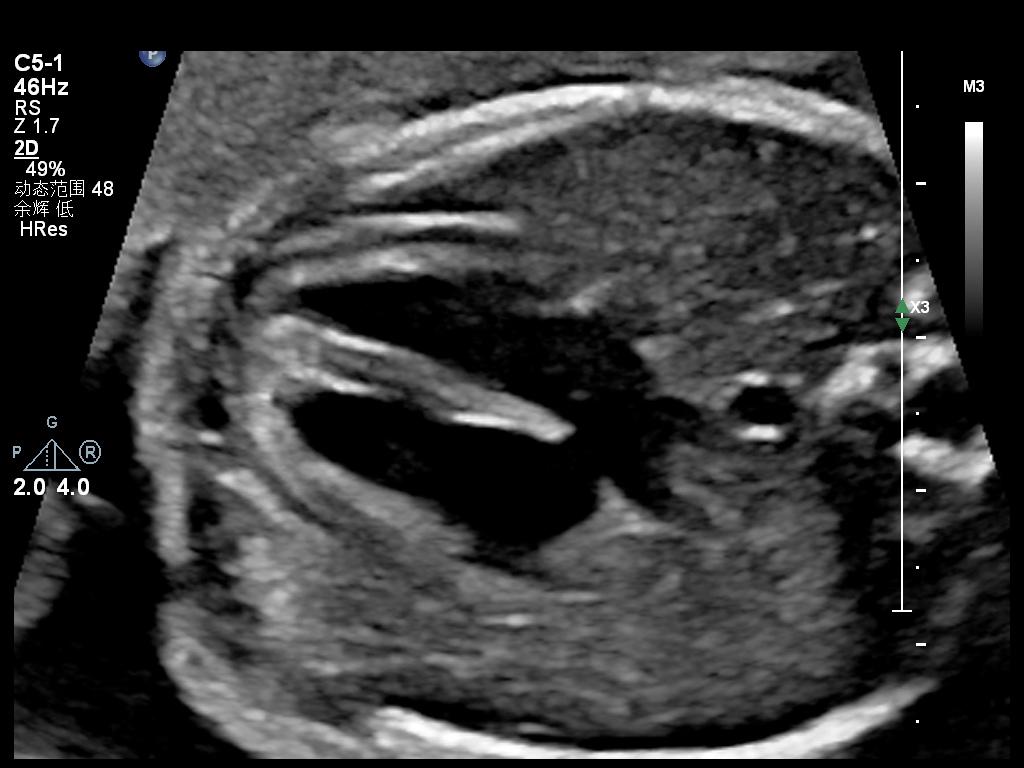

Supplement: S2 Dataset — (ZIP) [file pone.0305250.s002.zip › FE-SD-2/images/test_res/612_fc.jpg]

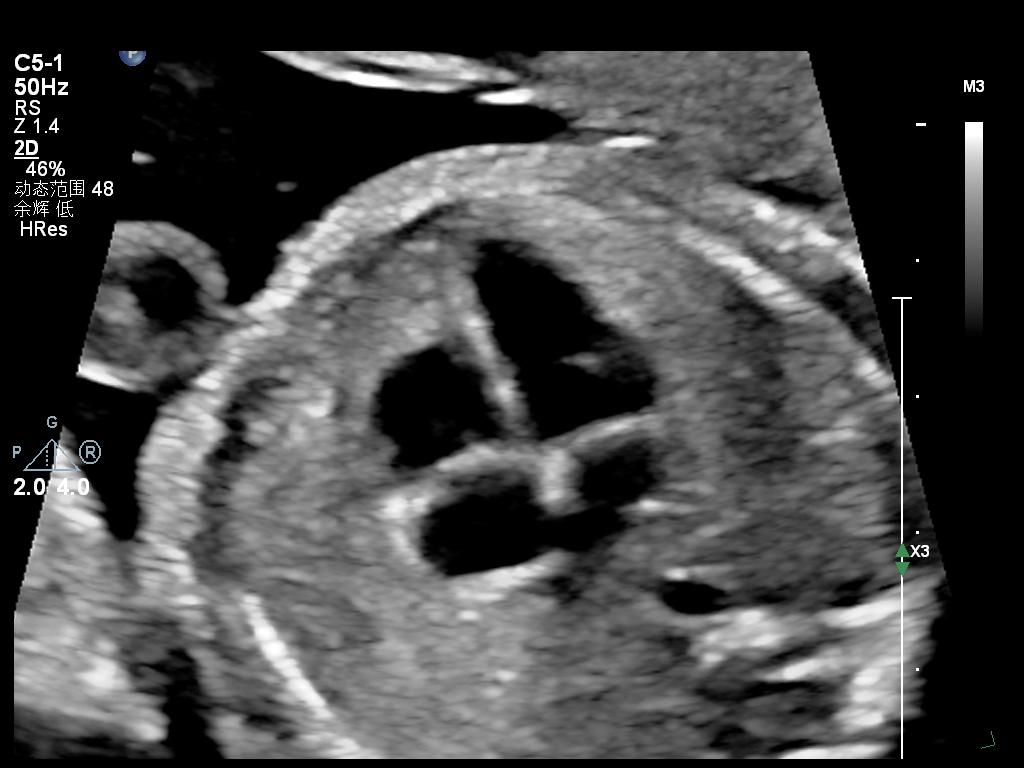

Supplement: S2 Dataset — (ZIP) [file pone.0305250.s002.zip › FE-SD-2/images/test_res/616_fc.jpg]

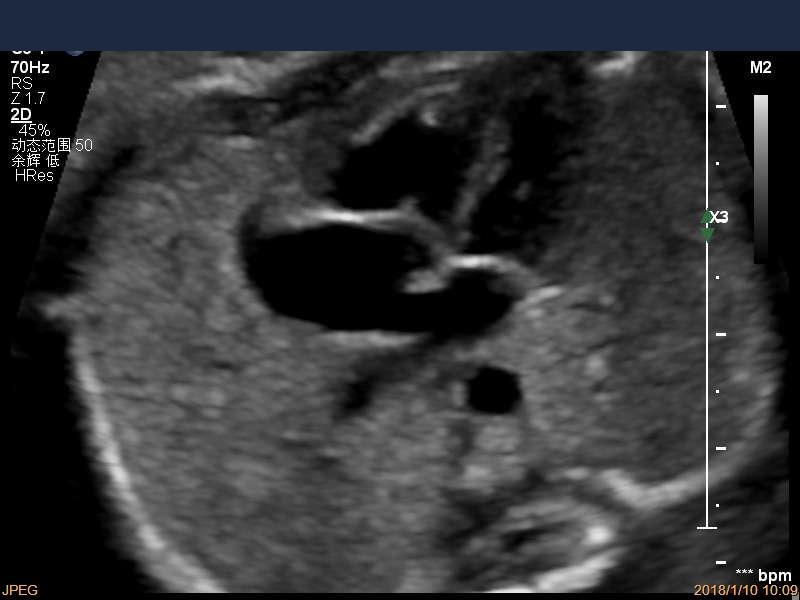

Supplement: S2 Dataset — (ZIP) [file pone.0305250.s002.zip › FE-SD-2/images/test_res/617_fc.jpg]

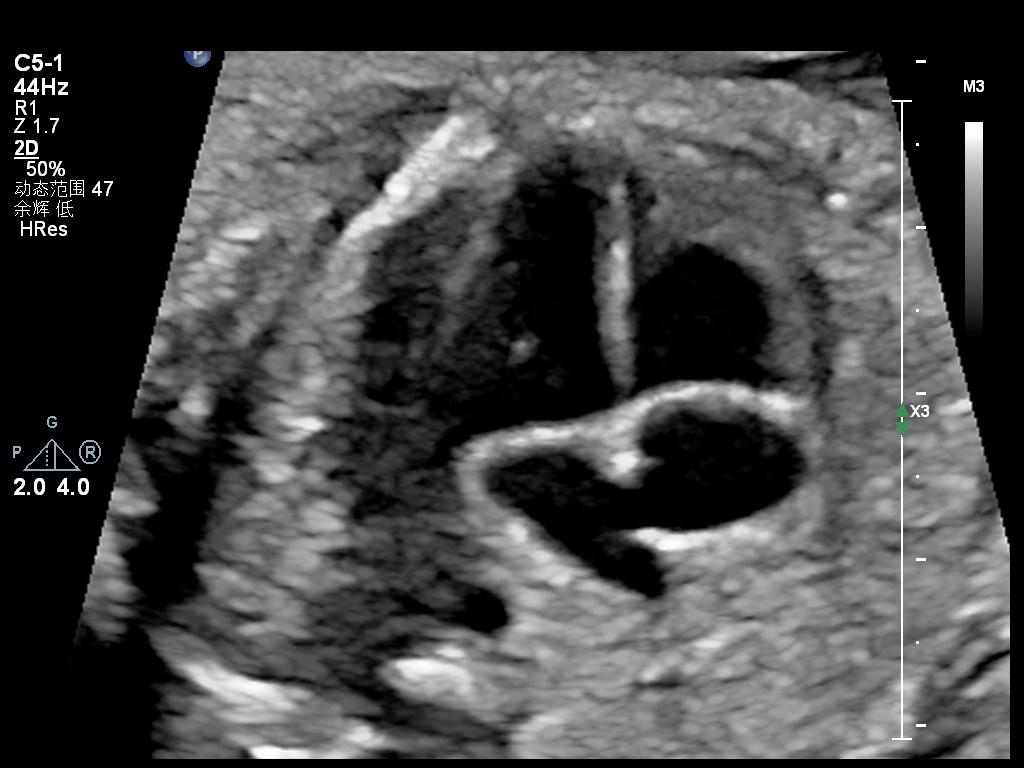

Supplement: S2 Dataset — (ZIP) [file pone.0305250.s002.zip › FE-SD-2/images/test_res/618_fc.jpg]

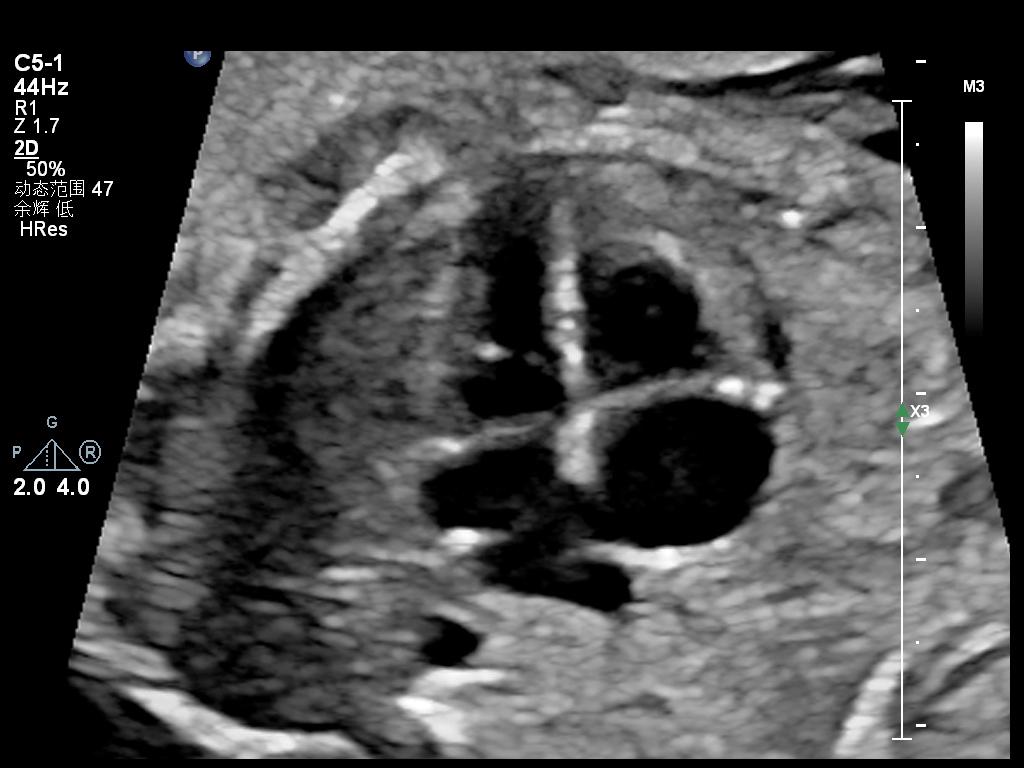

Supplement: S2 Dataset — (ZIP) [file pone.0305250.s002.zip › FE-SD-2/images/test_res/621_fc.jpg]

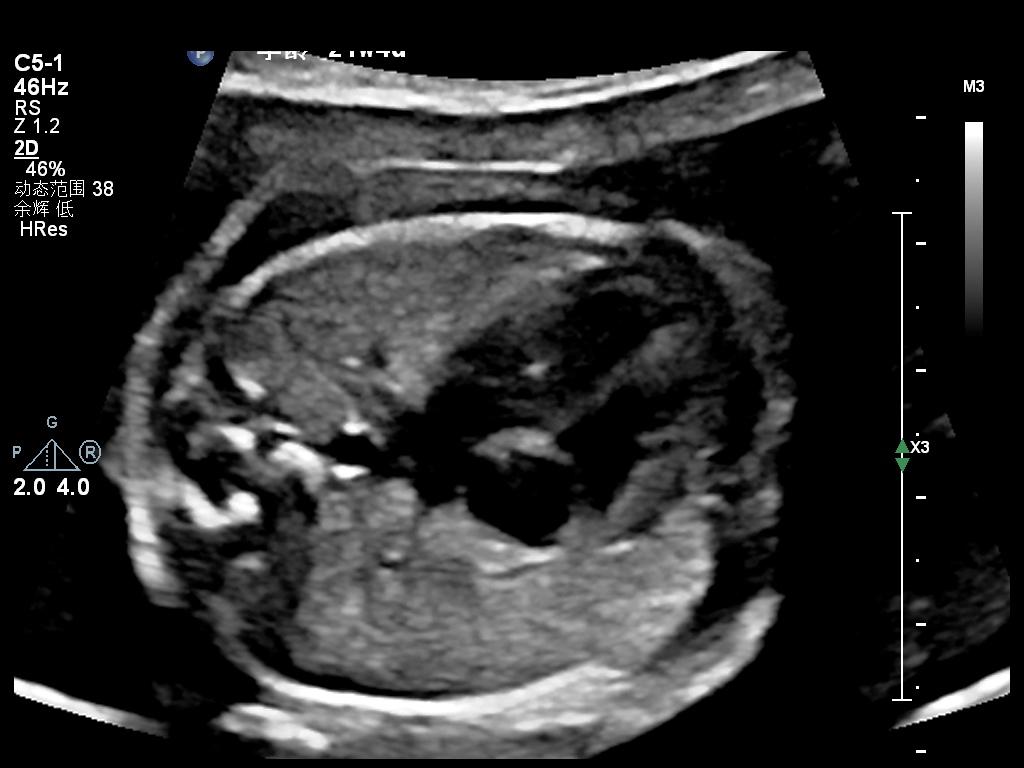

Supplement: S2 Dataset — (ZIP) [file pone.0305250.s002.zip › FE-SD-2/images/test_res/622_fc.jpg]

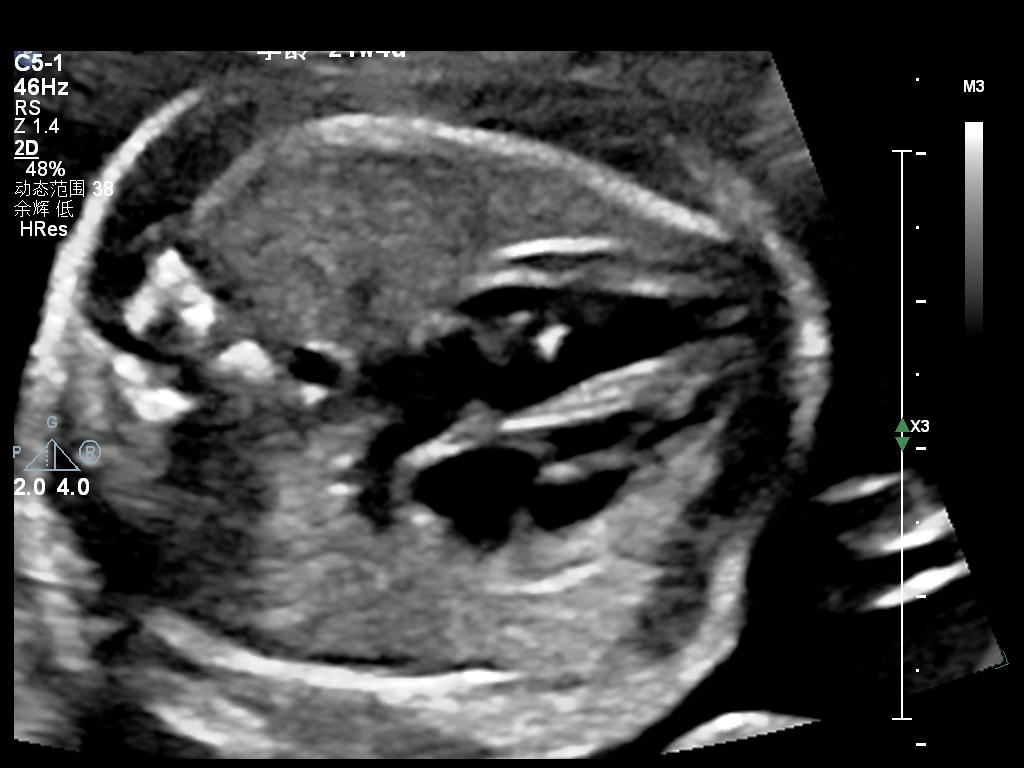

Supplement: S2 Dataset — (ZIP) [file pone.0305250.s002.zip › FE-SD-2/images/test_res/623_fc.jpg]

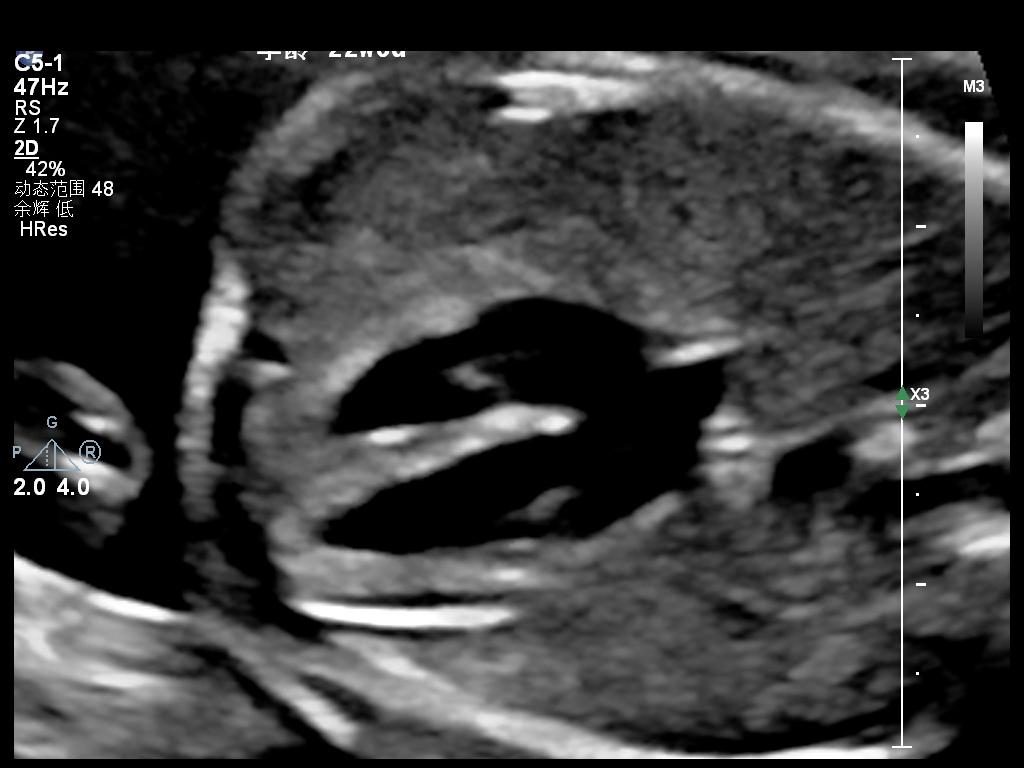

Supplement: S2 Dataset — (ZIP) [file pone.0305250.s002.zip › FE-SD-2/images/test_res/625_fc.jpg]

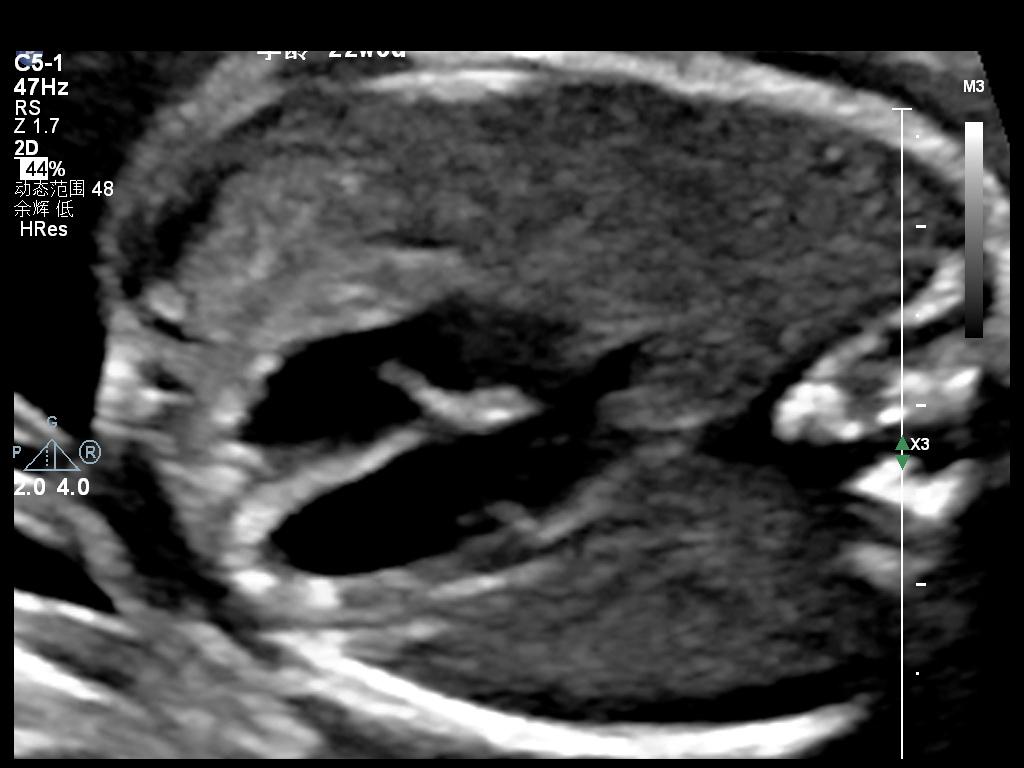

Supplement: S2 Dataset — (ZIP) [file pone.0305250.s002.zip › FE-SD-2/images/test_res/626_fc.jpg]

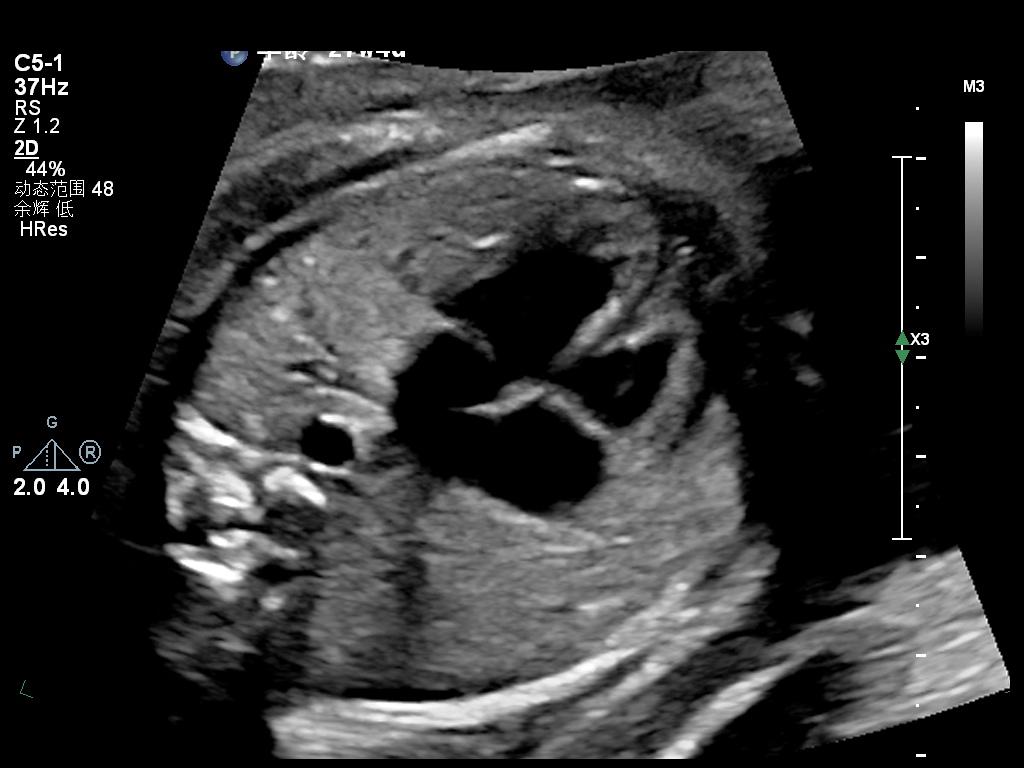

Supplement: S2 Dataset — (ZIP) [file pone.0305250.s002.zip › FE-SD-2/images/test_res/627_fc.jpg]

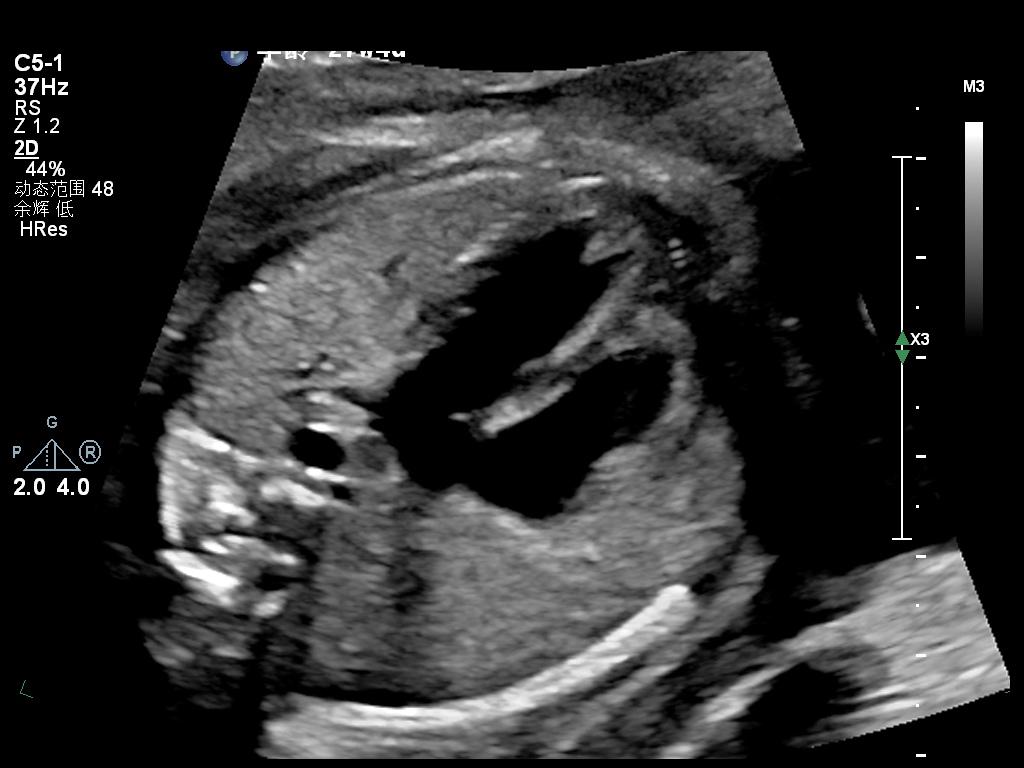

Supplement: S2 Dataset — (ZIP) [file pone.0305250.s002.zip › FE-SD-2/images/test_res/629_fc.jpg]

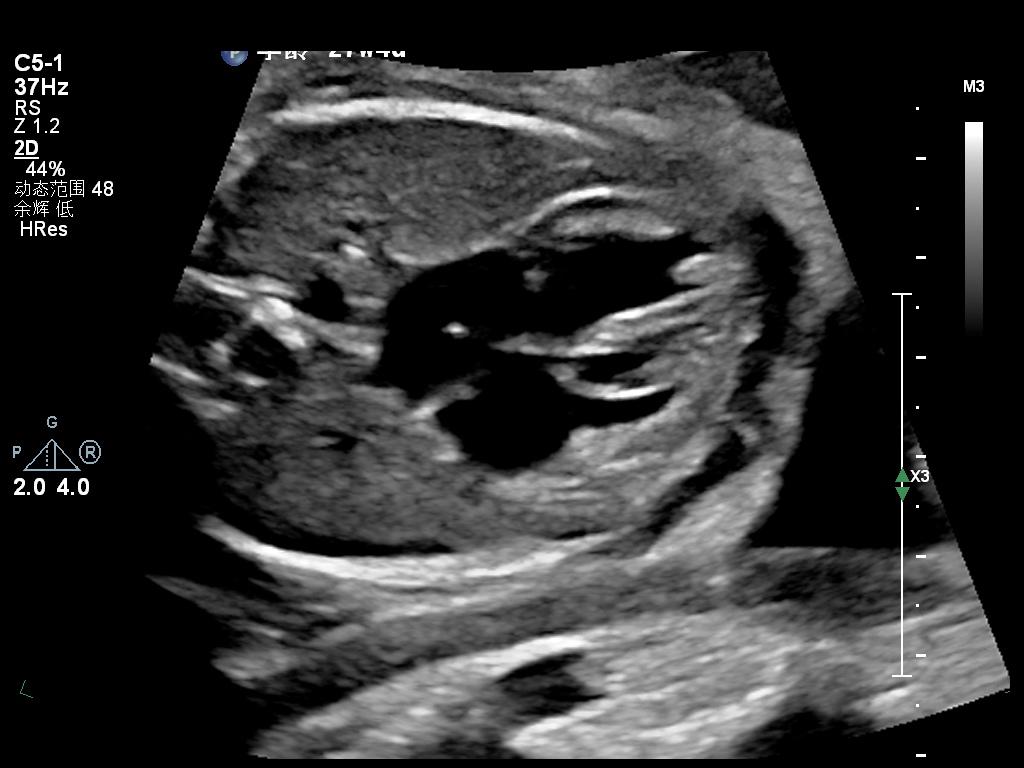

Supplement: S2 Dataset — (ZIP) [file pone.0305250.s002.zip › FE-SD-2/images/test_res/634_fc.jpg]

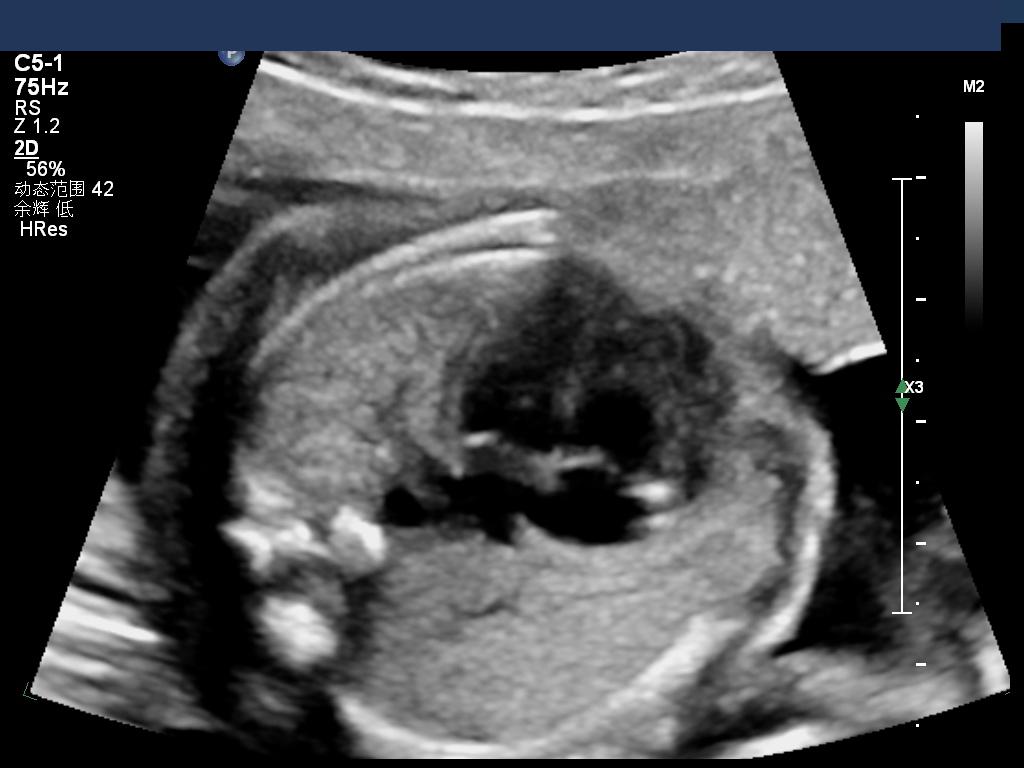

Supplement: S2 Dataset — (ZIP) [file pone.0305250.s002.zip › FE-SD-2/images/test_res/635_fc.jpg]

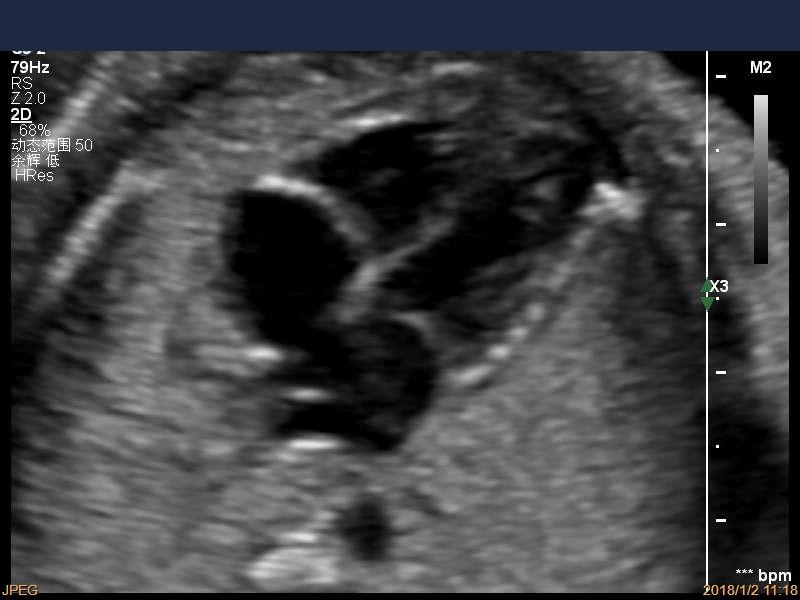

Supplement: S2 Dataset — (ZIP) [file pone.0305250.s002.zip › FE-SD-2/images/test_res/636_fc.jpg]

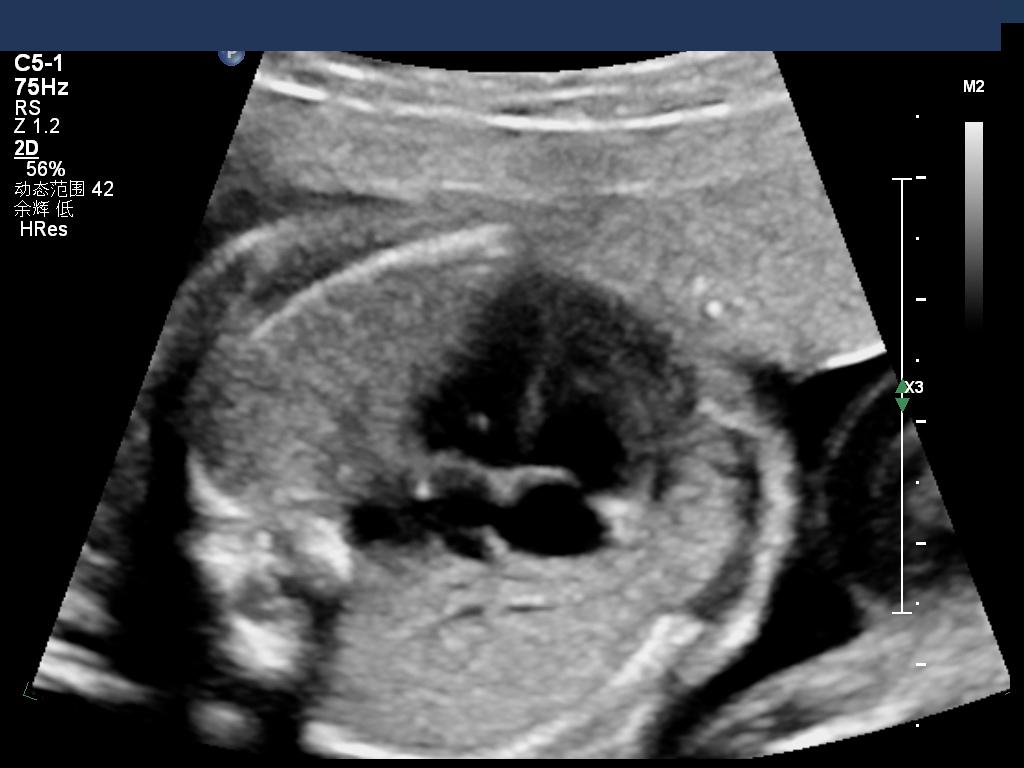

Supplement: S2 Dataset — (ZIP) [file pone.0305250.s002.zip › FE-SD-2/images/test_res/637_fc.jpg]

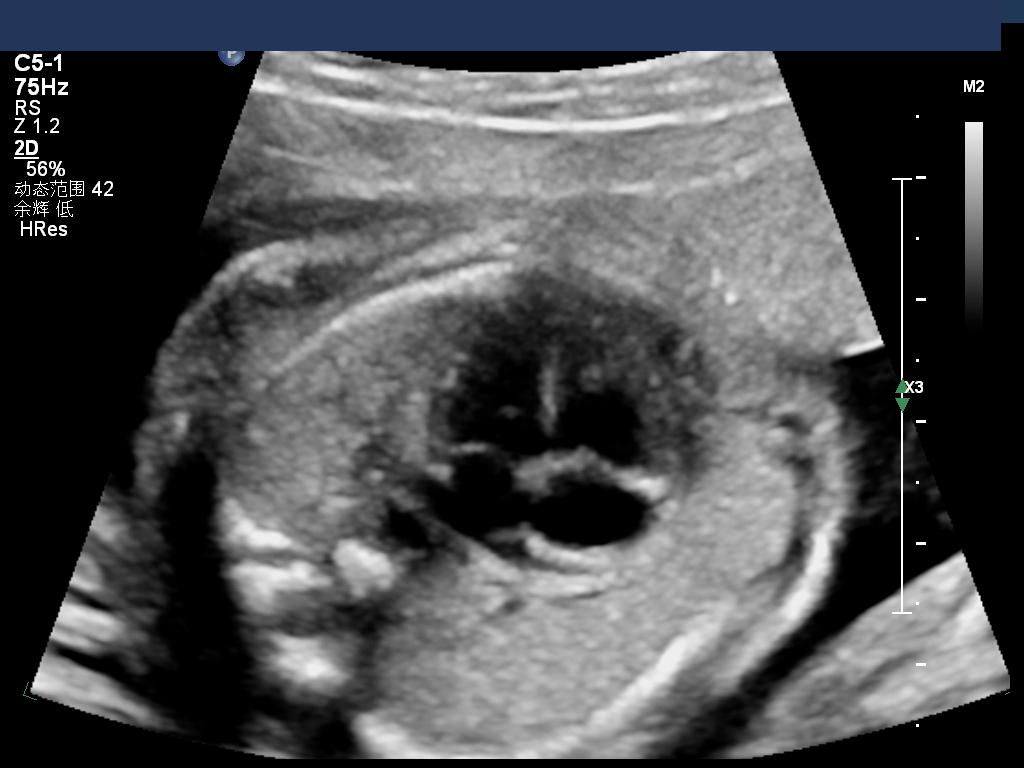

Supplement: S2 Dataset — (ZIP) [file pone.0305250.s002.zip › FE-SD-2/images/test_res/638_fc.jpg]

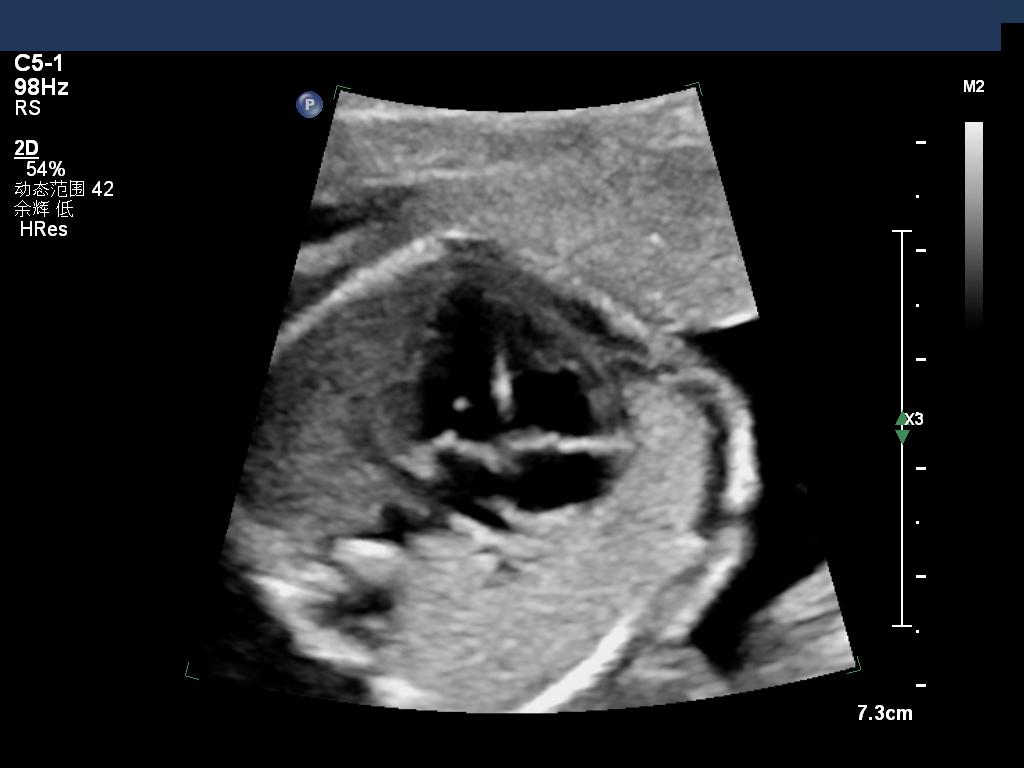

Supplement: S2 Dataset — (ZIP) [file pone.0305250.s002.zip › FE-SD-2/images/test_res/640_fc.jpg]

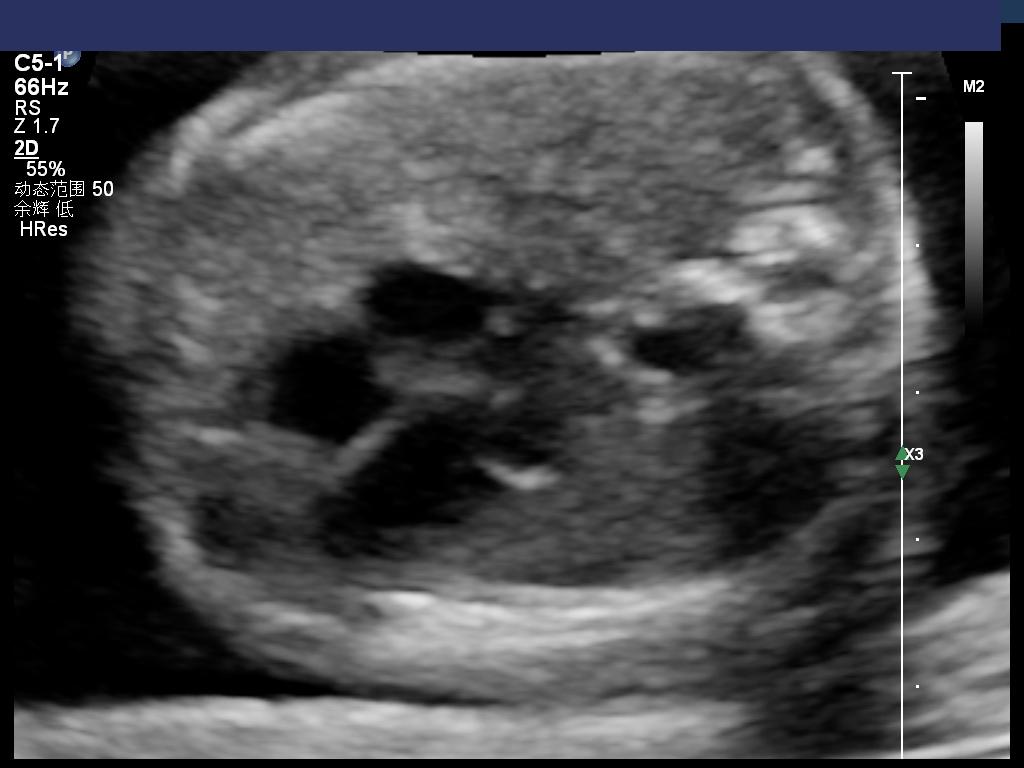

Supplement: S2 Dataset — (ZIP) [file pone.0305250.s002.zip › FE-SD-2/images/test_res/641_fc.jpg]

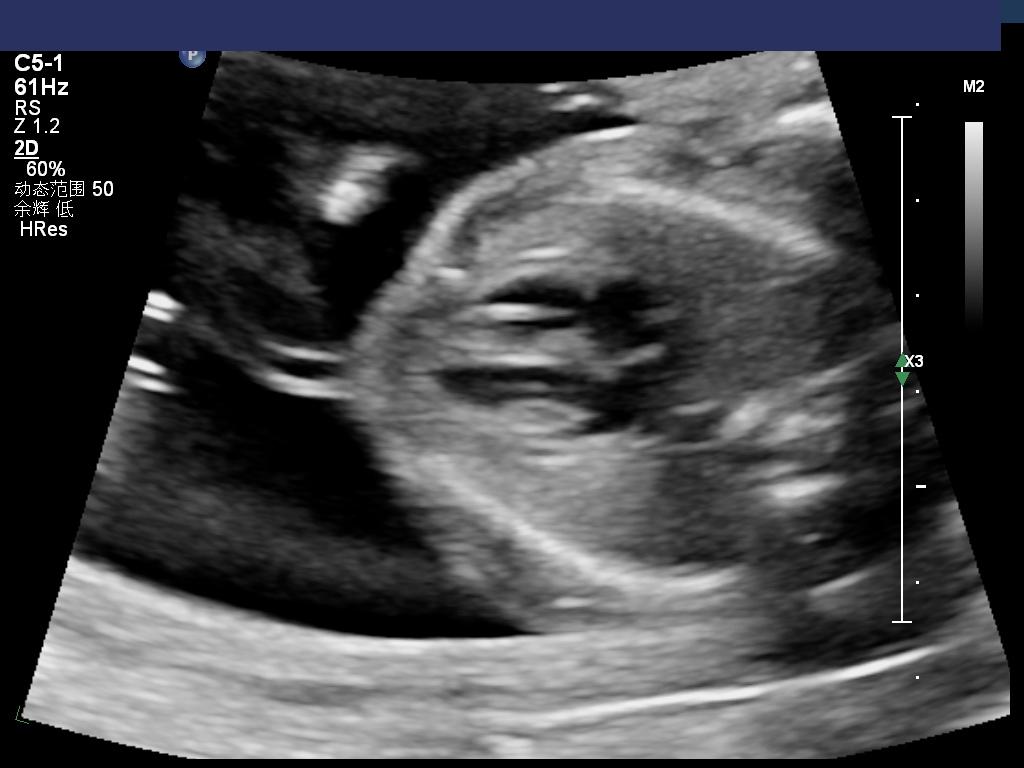

Supplement: S2 Dataset — (ZIP) [file pone.0305250.s002.zip › FE-SD-2/images/test_res/644_fc.jpg]

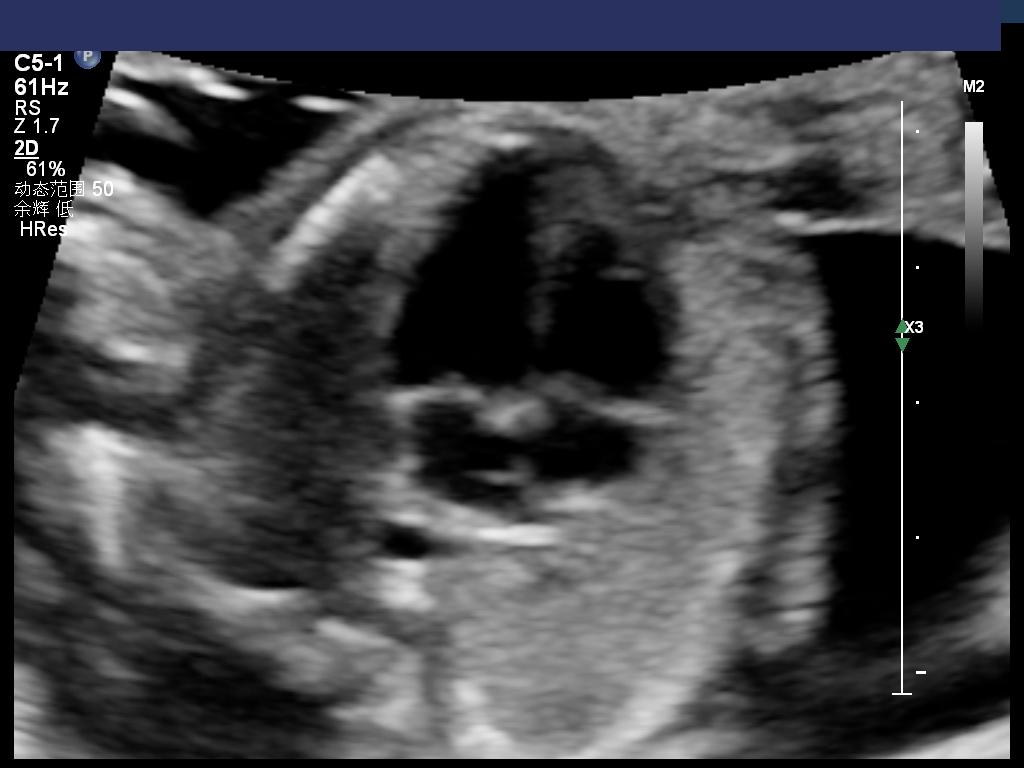

Supplement: S2 Dataset — (ZIP) [file pone.0305250.s002.zip › FE-SD-2/images/test_res/646_fc.jpg]

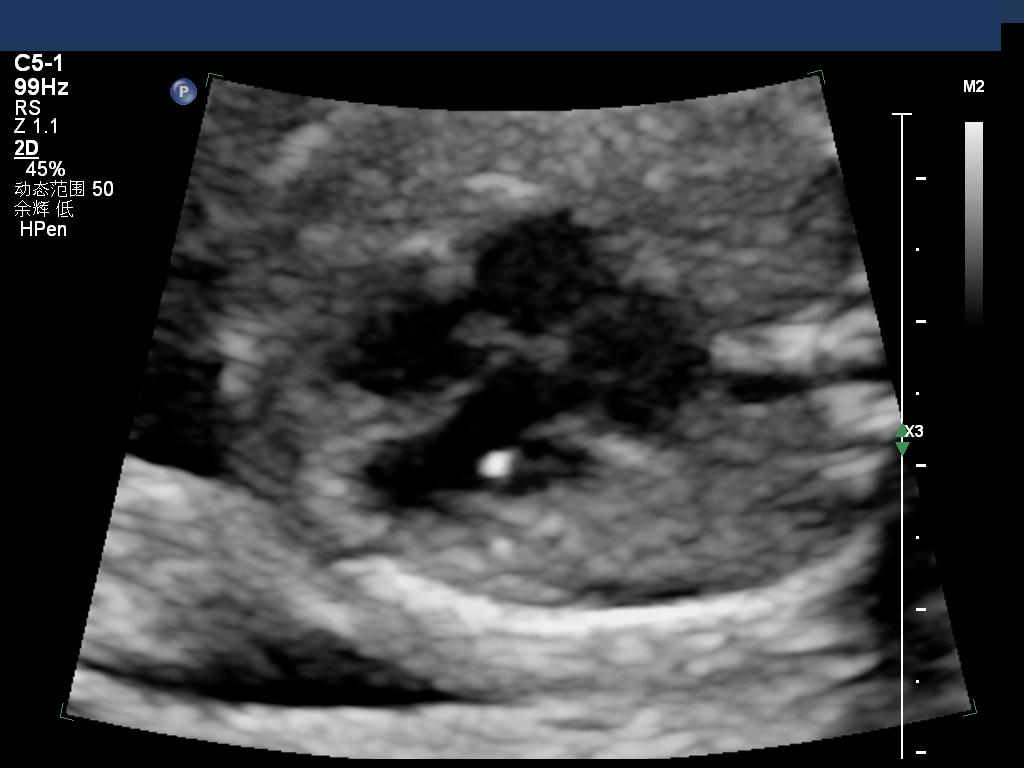

Supplement: S2 Dataset — (ZIP) [file pone.0305250.s002.zip › FE-SD-2/images/test_res/649_fc.jpg]

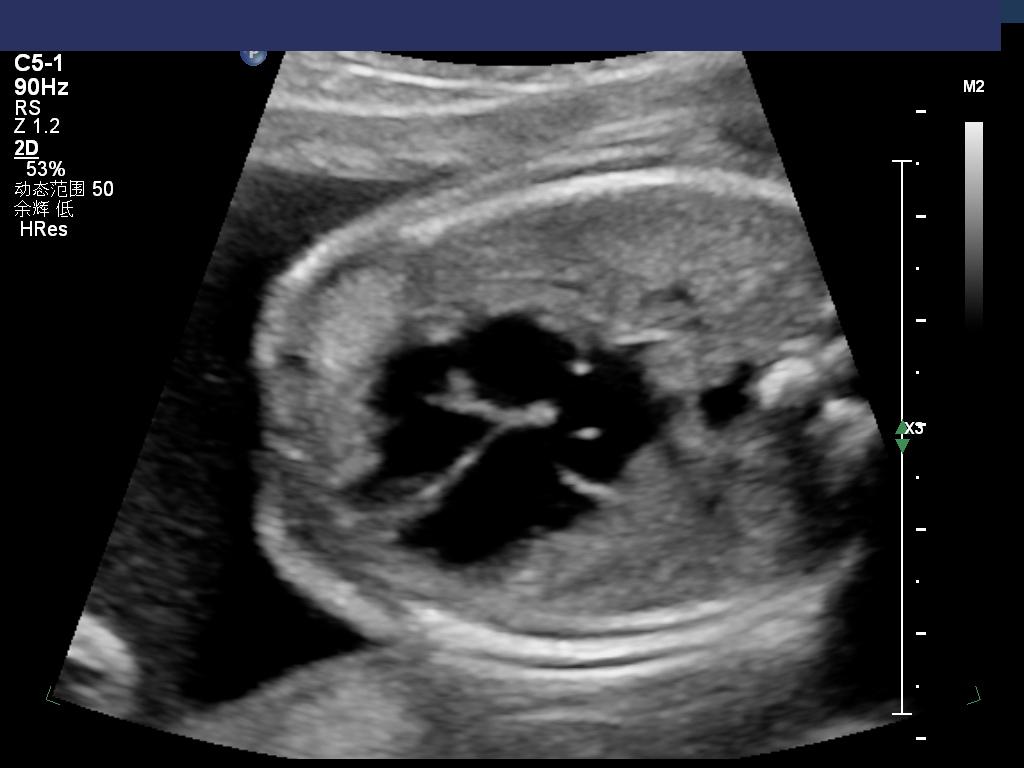

Supplement: S2 Dataset — (ZIP) [file pone.0305250.s002.zip › FE-SD-2/images/test_res/650_fc.jpg]

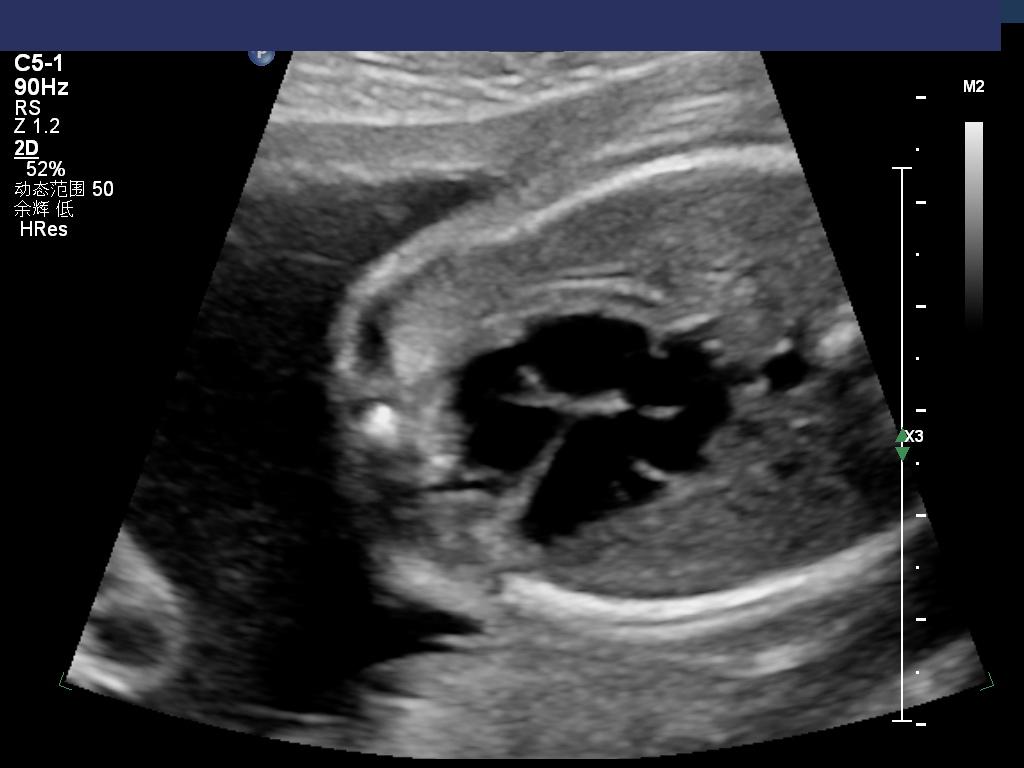

Supplement: S2 Dataset — (ZIP) [file pone.0305250.s002.zip › FE-SD-2/images/test_res/651_fc.jpg]

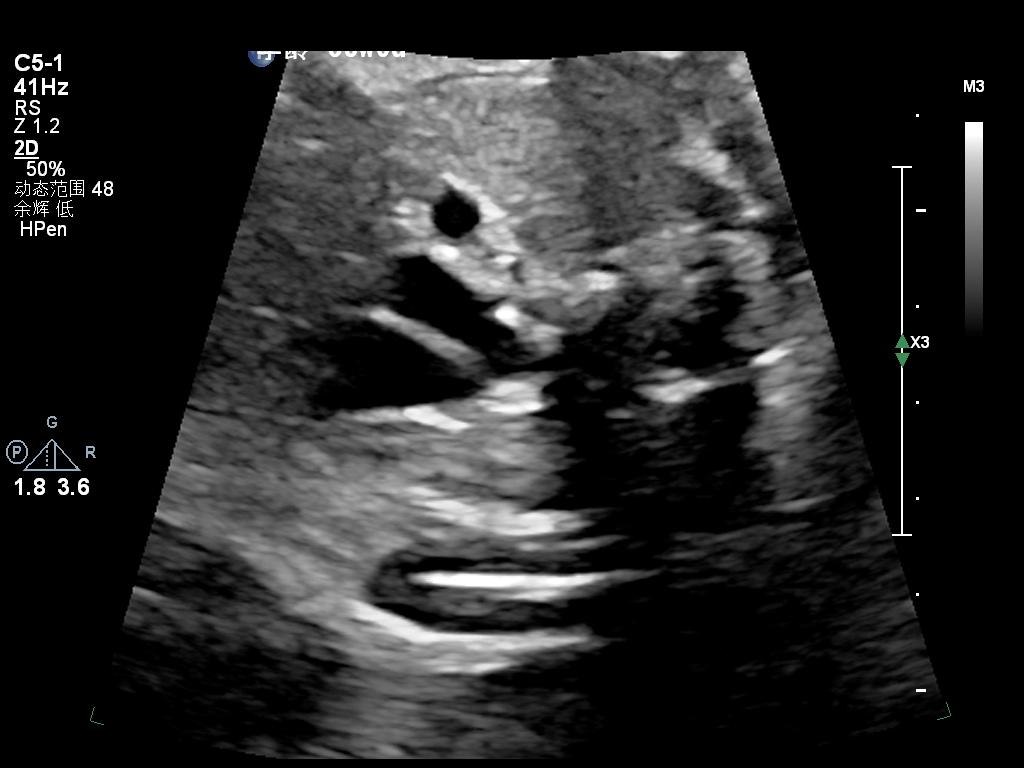

Supplement: S2 Dataset — (ZIP) [file pone.0305250.s002.zip › FE-SD-2/images/test_res/654_tv.jpg]

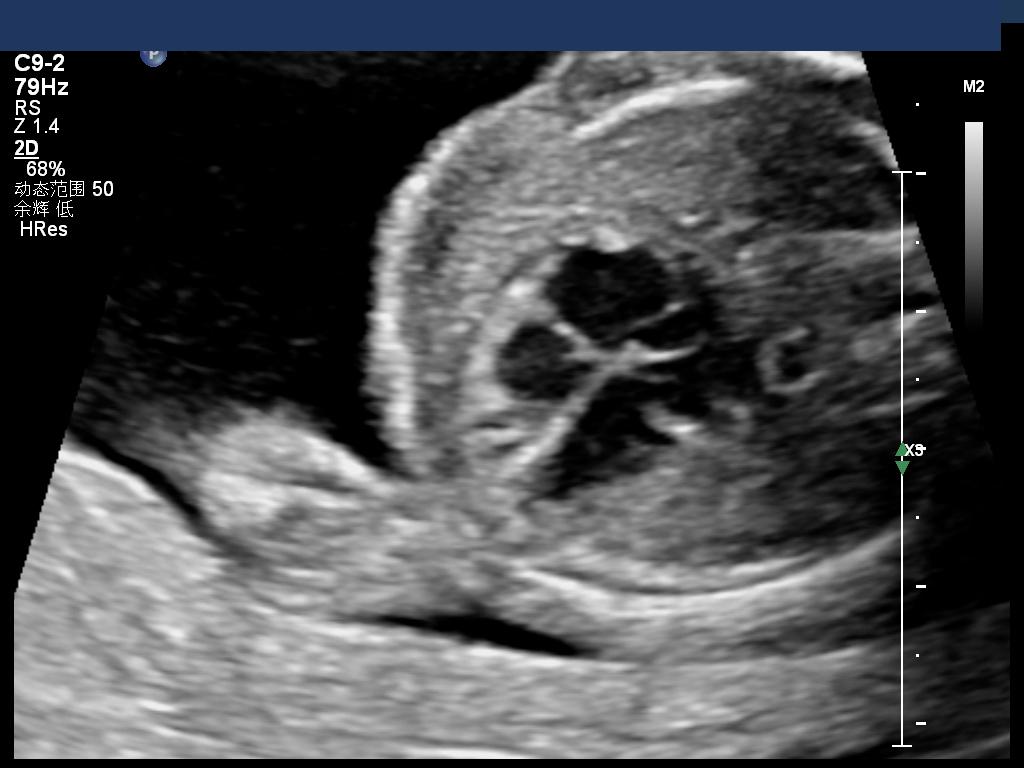

Supplement: S2 Dataset — (ZIP) [file pone.0305250.s002.zip › FE-SD-2/images/test_res/655_fc.jpg]

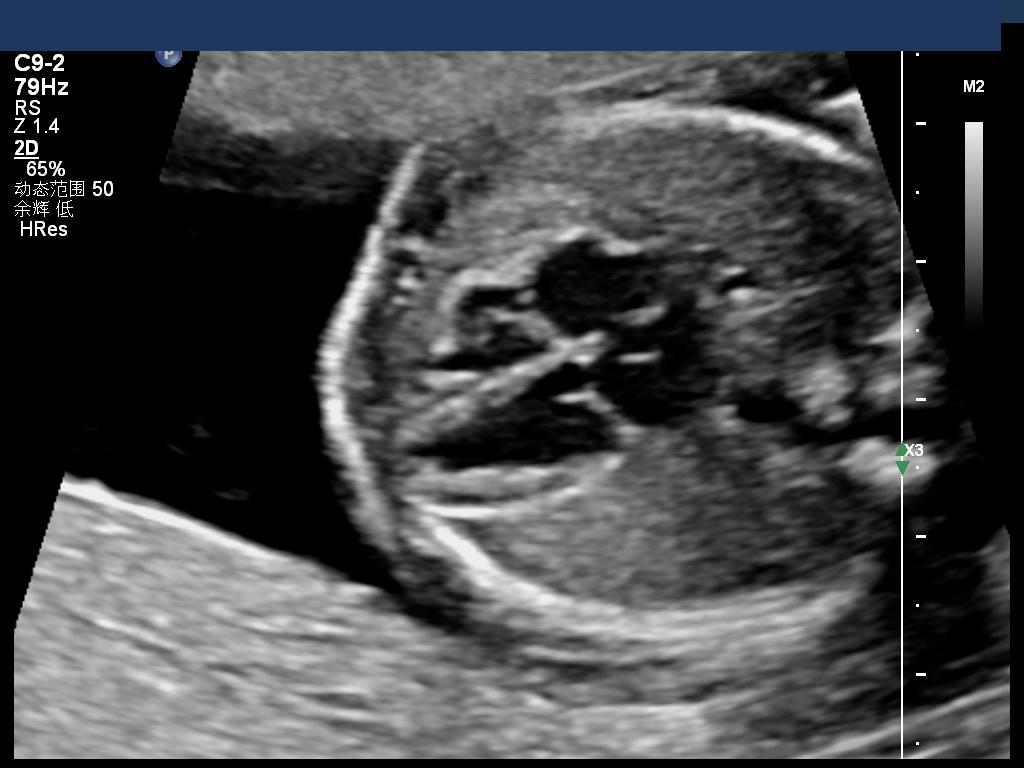

Supplement: S2 Dataset — (ZIP) [file pone.0305250.s002.zip › FE-SD-2/images/test_res/658_fc.jpg]

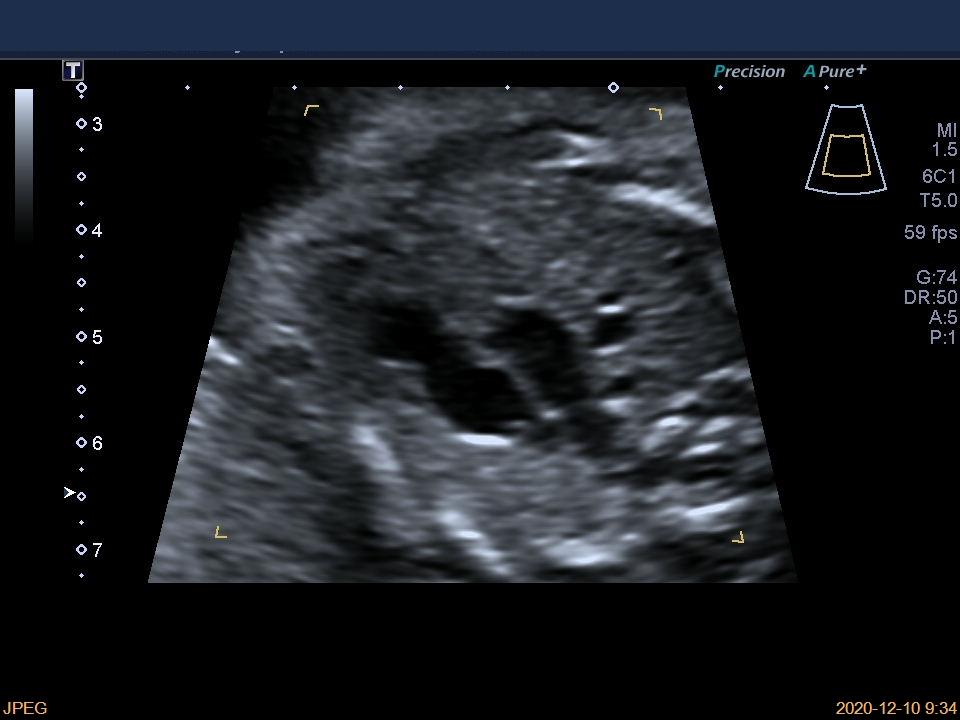

Supplement: S2 Dataset — (ZIP) [file pone.0305250.s002.zip › FE-SD-2/images/test_res/658_tv.jpg]

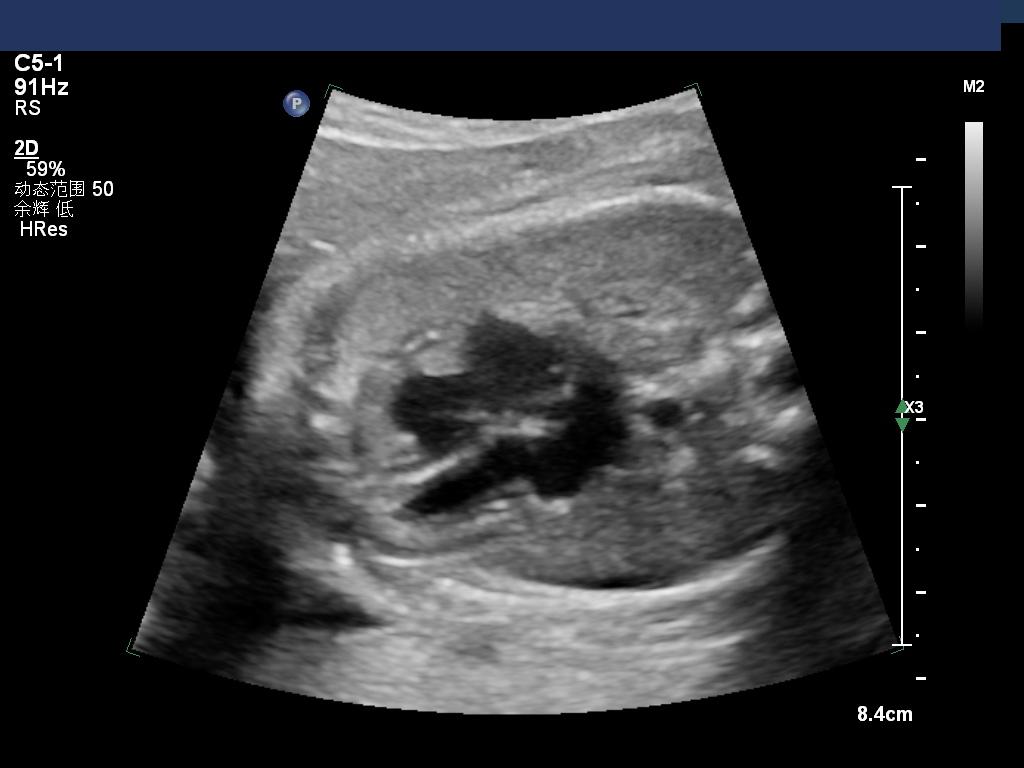

Supplement: S2 Dataset — (ZIP) [file pone.0305250.s002.zip › FE-SD-2/images/test_res/659_fc.jpg]

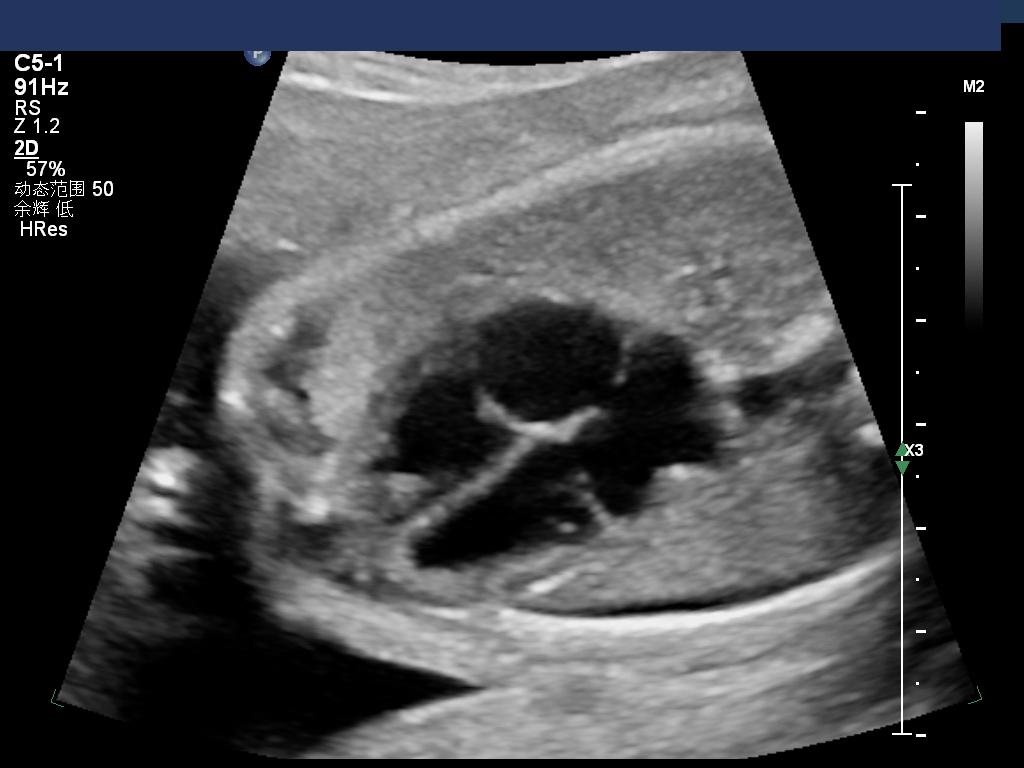

Supplement: S2 Dataset — (ZIP) [file pone.0305250.s002.zip › FE-SD-2/images/test_res/660_fc.jpg]

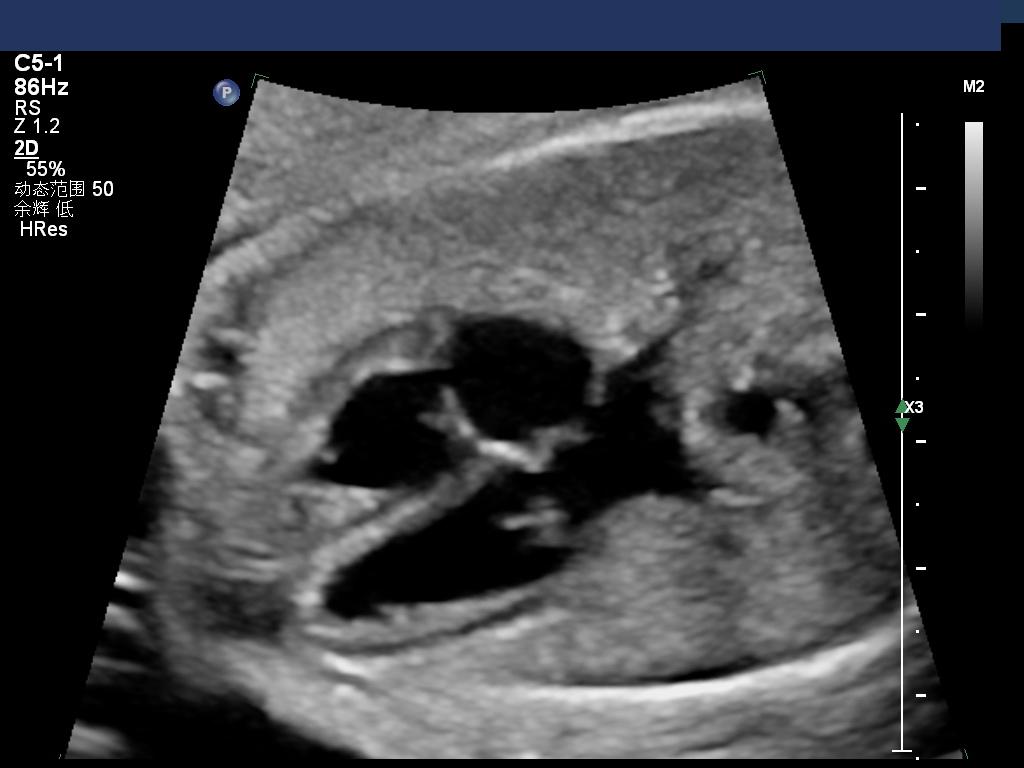

Supplement: S2 Dataset — (ZIP) [file pone.0305250.s002.zip › FE-SD-2/images/test_res/661_fc.jpg]

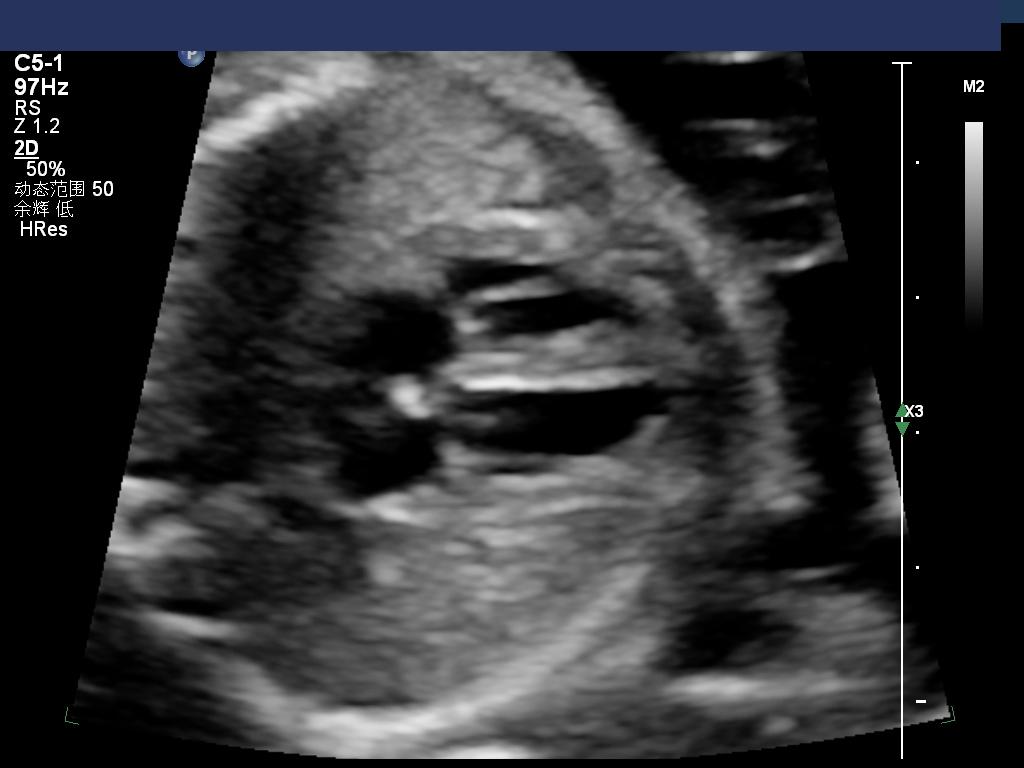

Supplement: S2 Dataset — (ZIP) [file pone.0305250.s002.zip › FE-SD-2/images/test_res/662_fc.jpg]

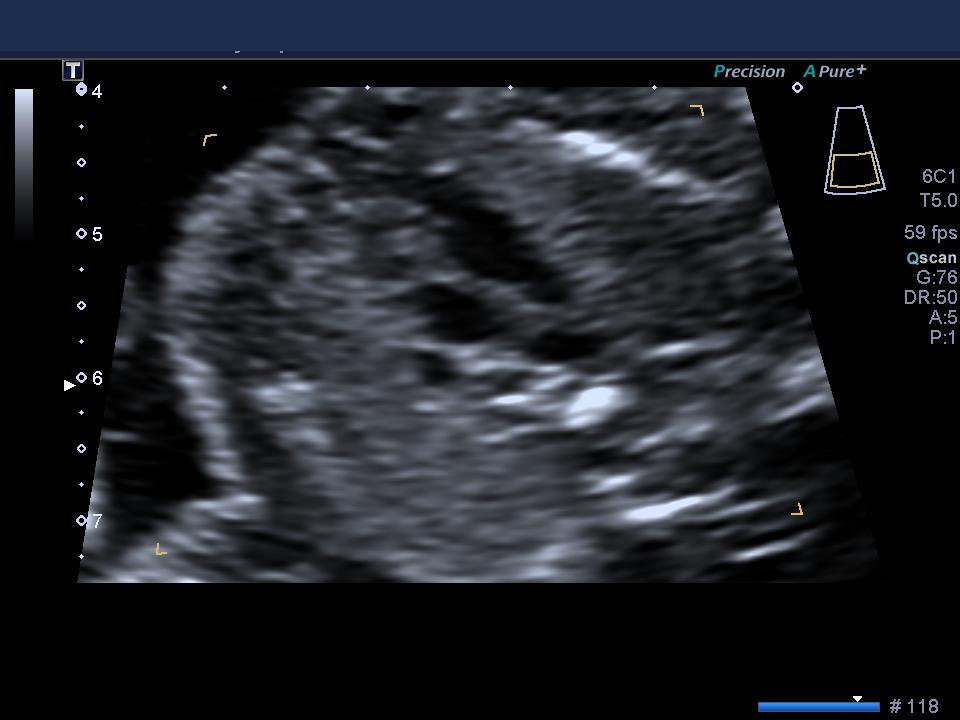

Supplement: S2 Dataset — (ZIP) [file pone.0305250.s002.zip › FE-SD-2/images/test_res/663_tv.jpg]

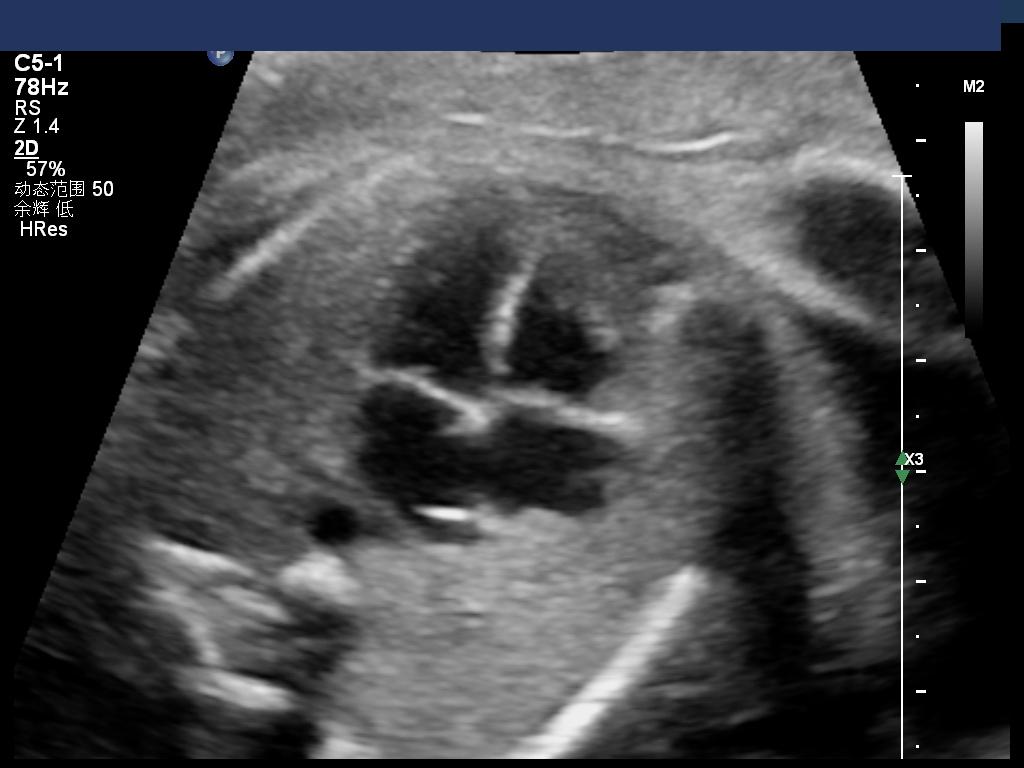

Supplement: S2 Dataset — (ZIP) [file pone.0305250.s002.zip › FE-SD-2/images/test_res/665_fc.jpg]

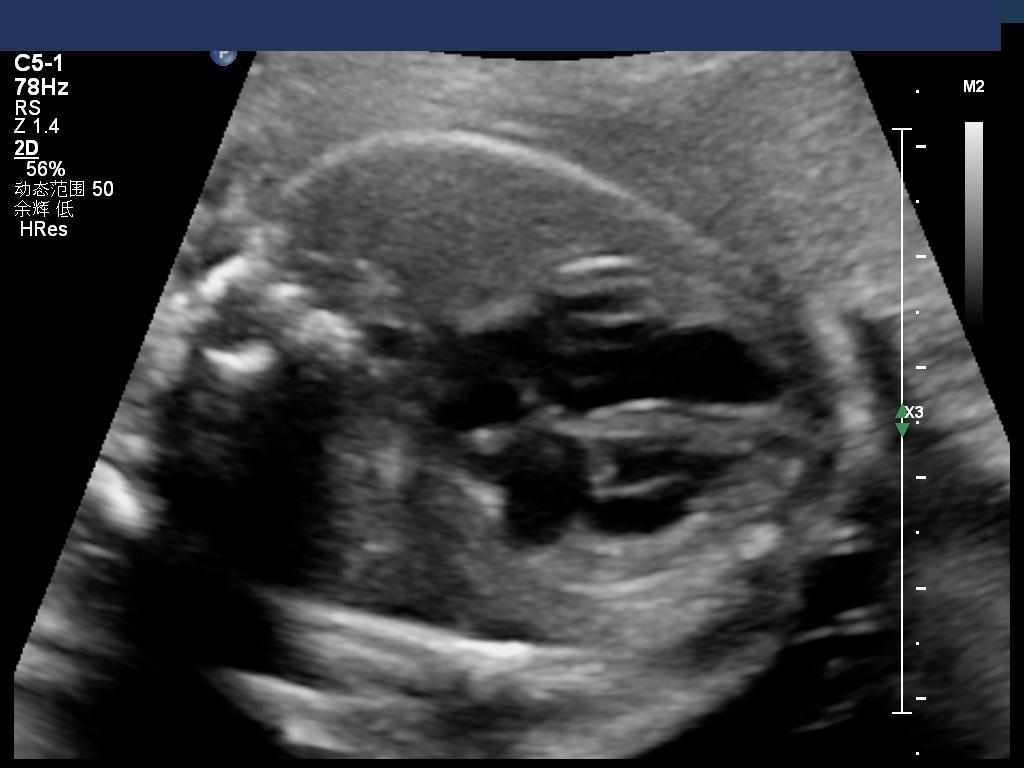

Supplement: S2 Dataset — (ZIP) [file pone.0305250.s002.zip › FE-SD-2/images/test_res/666_fc.jpg]

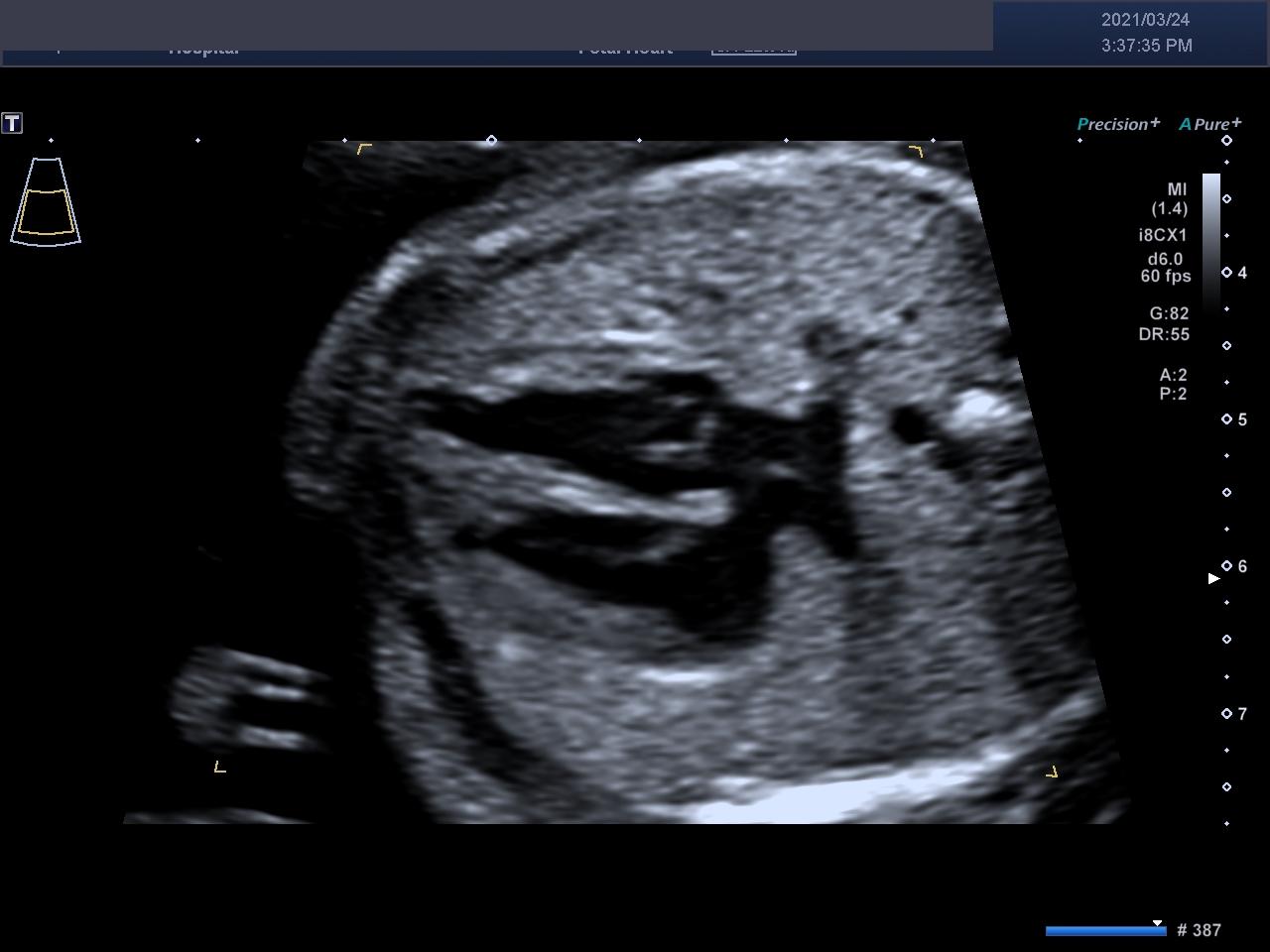

Supplement: S2 Dataset — (ZIP) [file pone.0305250.s002.zip › FE-SD-2/images/test_res/682_fc.jpg]

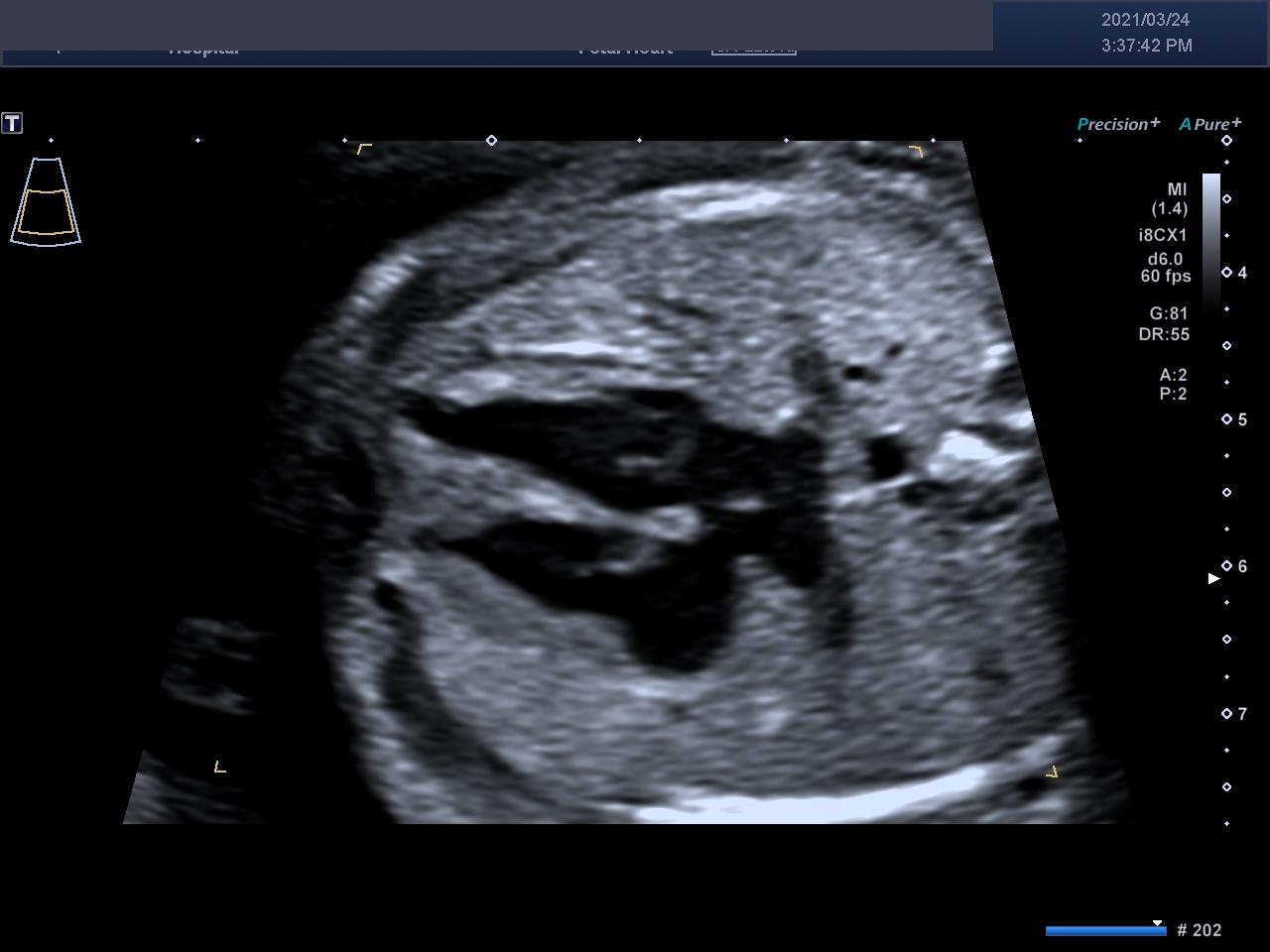

Supplement: S2 Dataset — (ZIP) [file pone.0305250.s002.zip › FE-SD-2/images/test_res/683_fc.jpg]

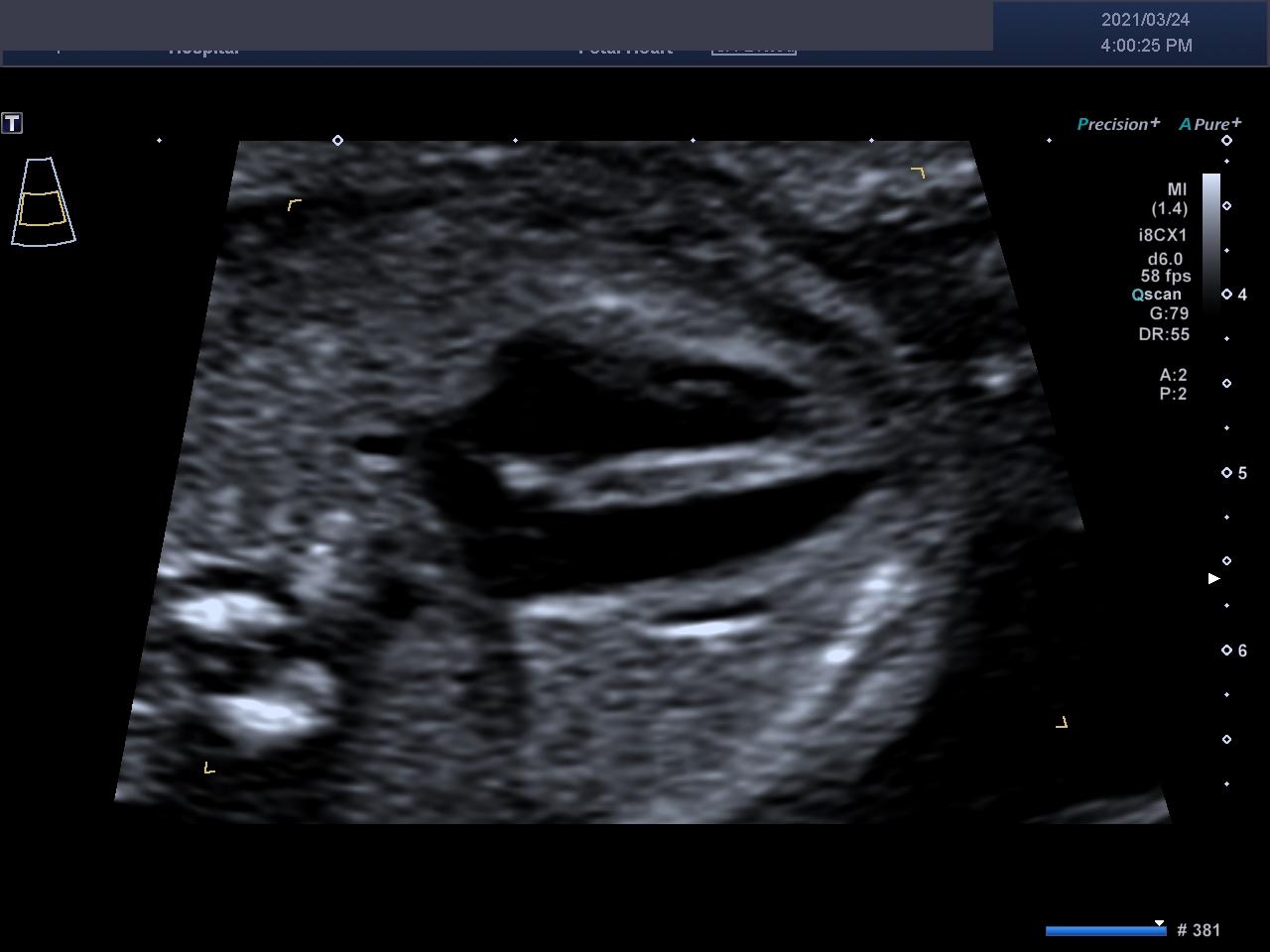

Supplement: S2 Dataset — (ZIP) [file pone.0305250.s002.zip › FE-SD-2/images/test_res/689_fc.jpg]

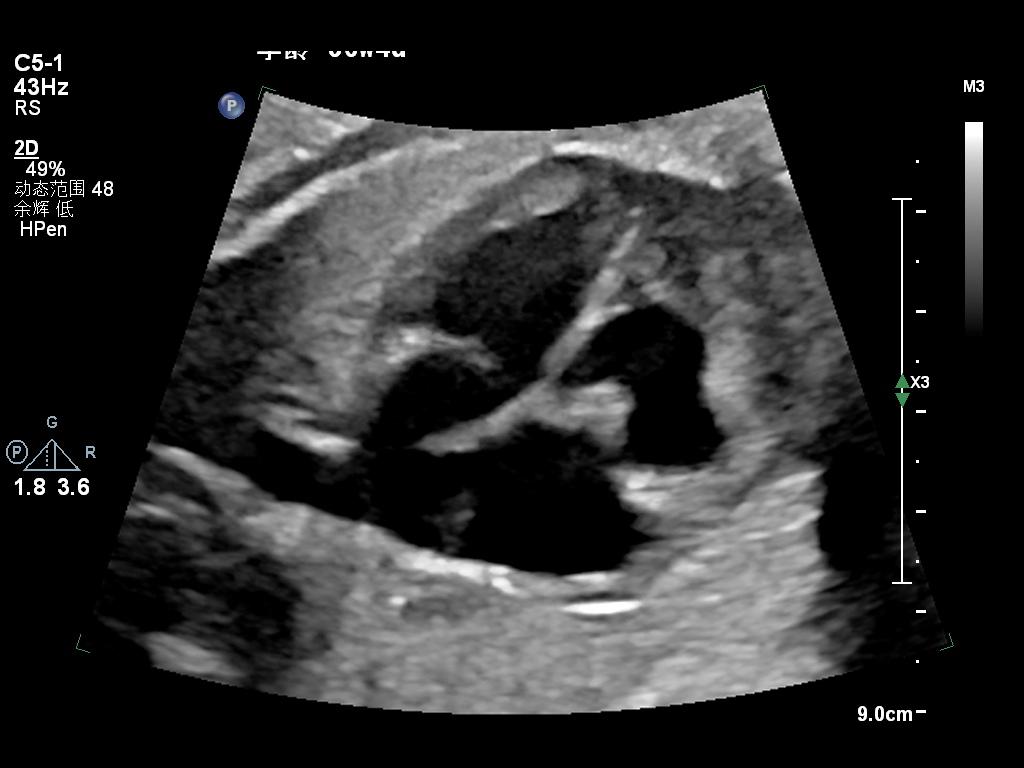

Supplement: S2 Dataset — (ZIP) [file pone.0305250.s002.zip › FE-SD-2/images/test_res/690_fc.jpg]

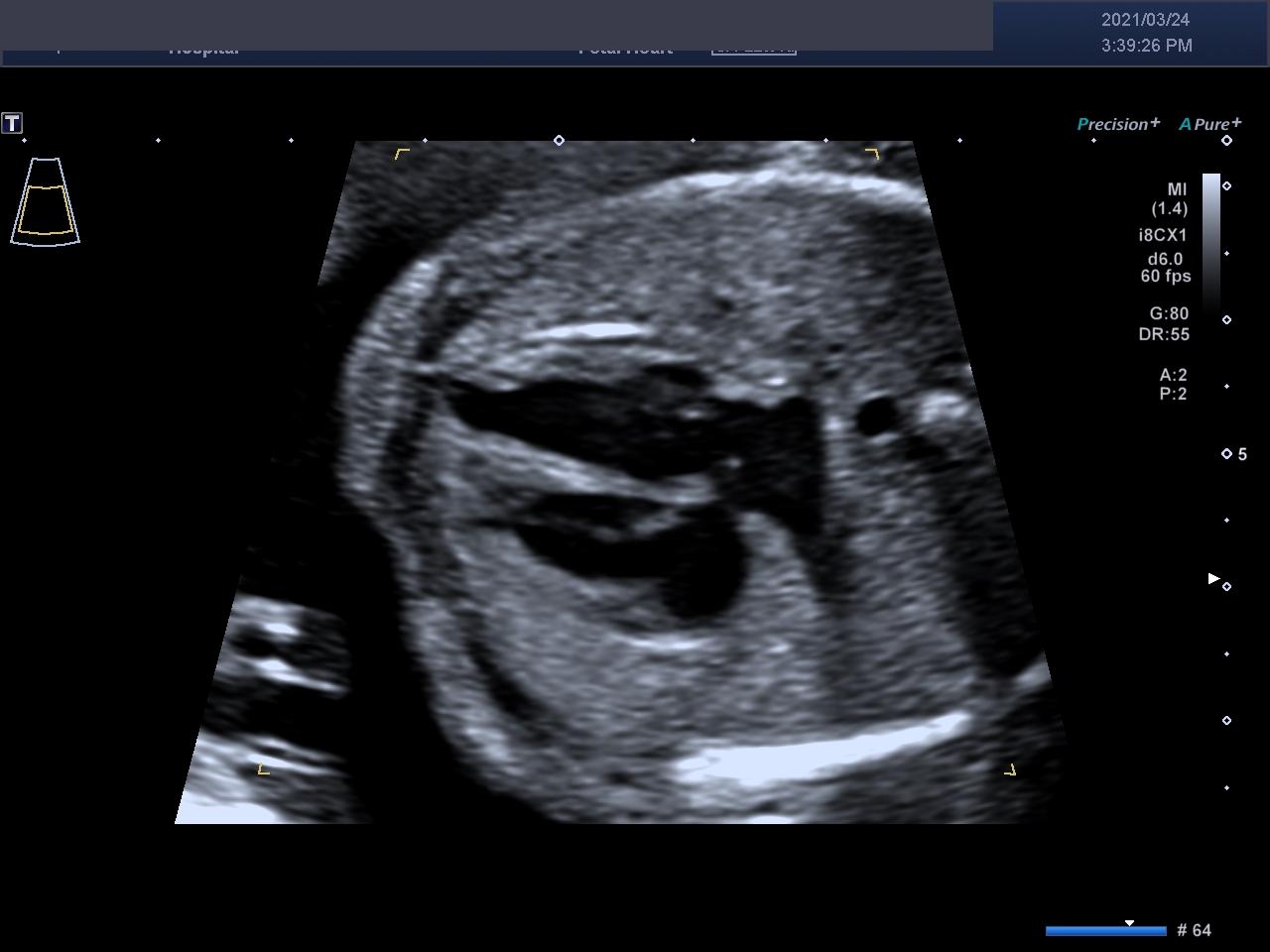

Supplement: S2 Dataset — (ZIP) [file pone.0305250.s002.zip › FE-SD-2/images/test_res/701_fc.jpg]

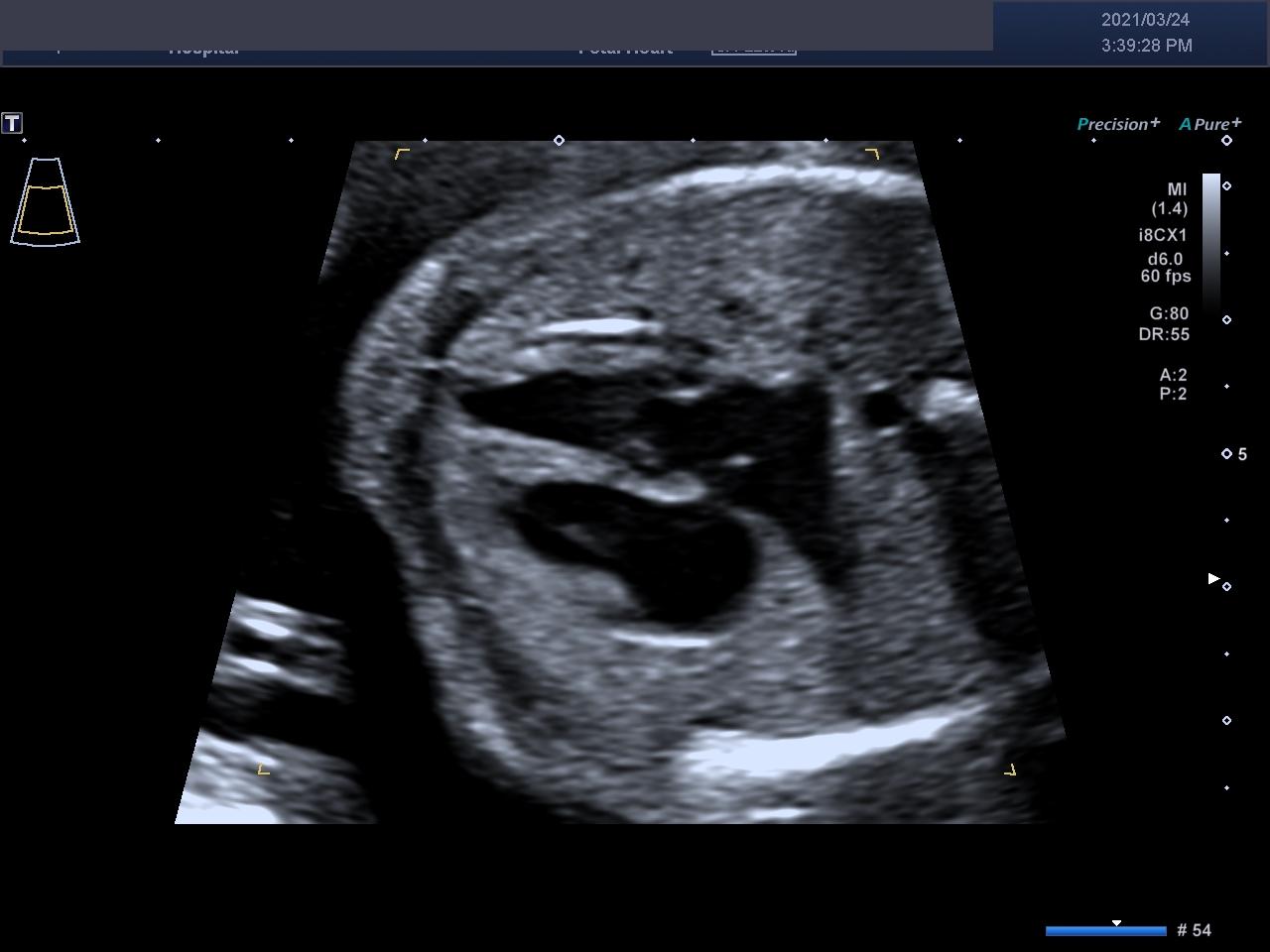

Supplement: S2 Dataset — (ZIP) [file pone.0305250.s002.zip › FE-SD-2/images/test_res/704_fc.jpg]

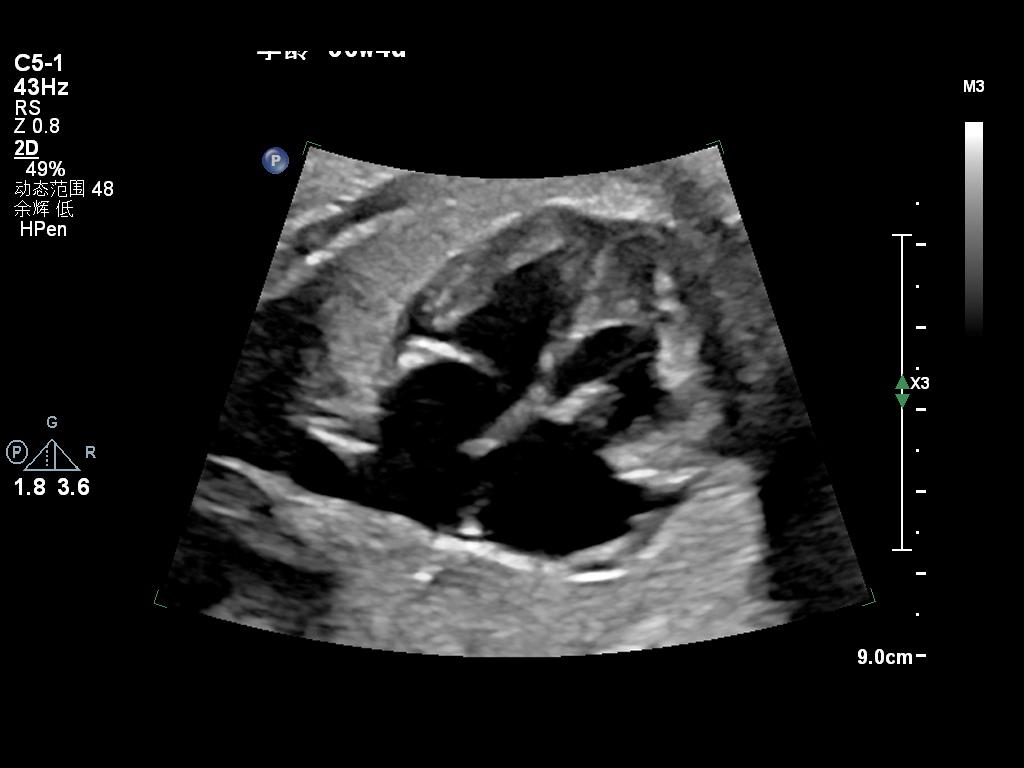

Supplement: S2 Dataset — (ZIP) [file pone.0305250.s002.zip › FE-SD-2/images/test_res/707_fc.jpg]

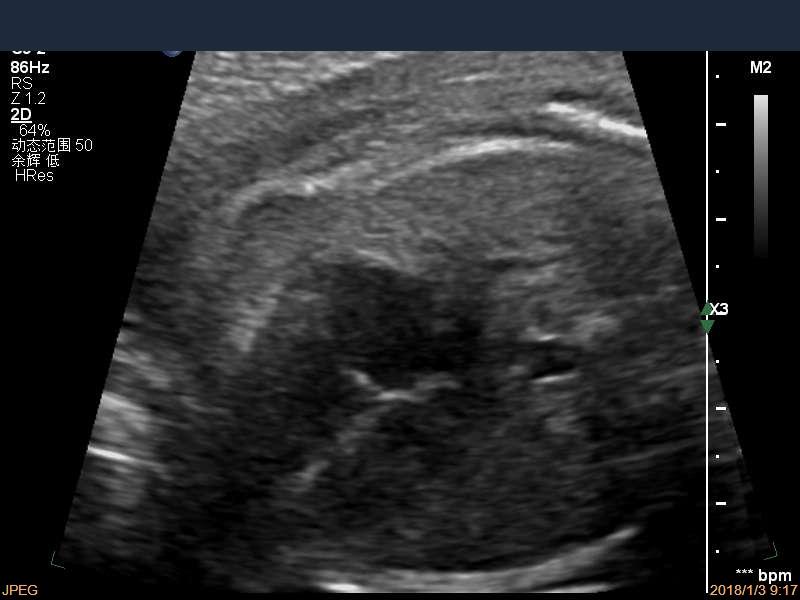

Supplement: S2 Dataset — (ZIP) [file pone.0305250.s002.zip › FE-SD-2/images/test_res/727_fc.jpg]

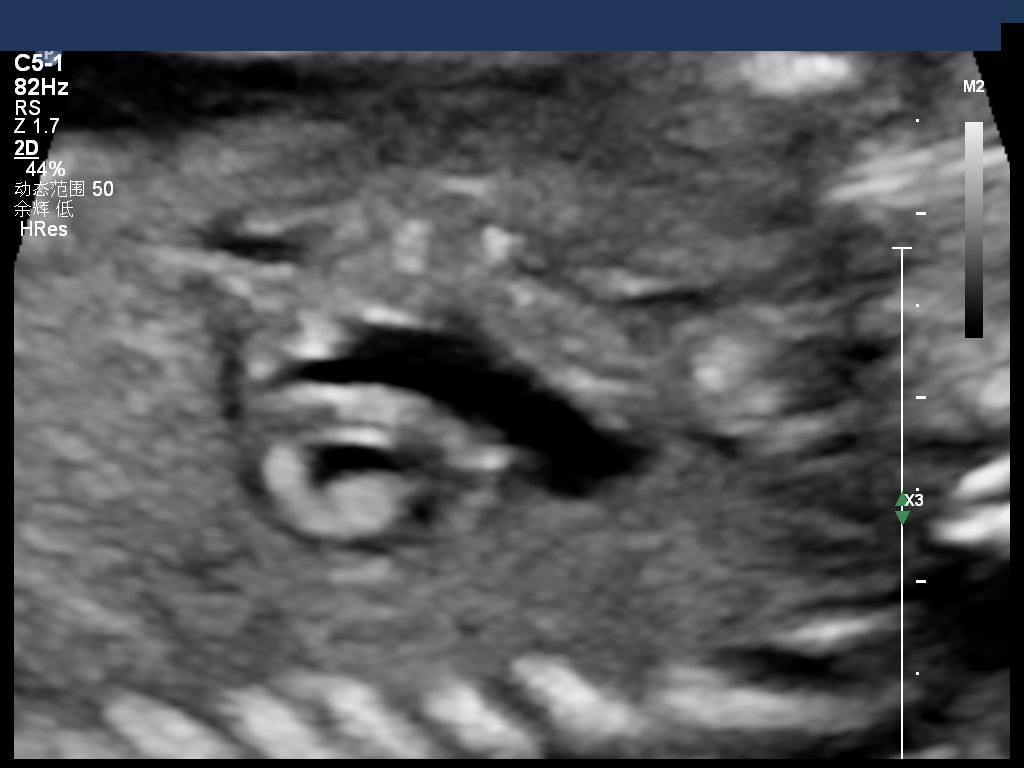

Supplement: S2 Dataset — (ZIP) [file pone.0305250.s002.zip › FE-SD-2/images/test_res/813_ro.jpg]

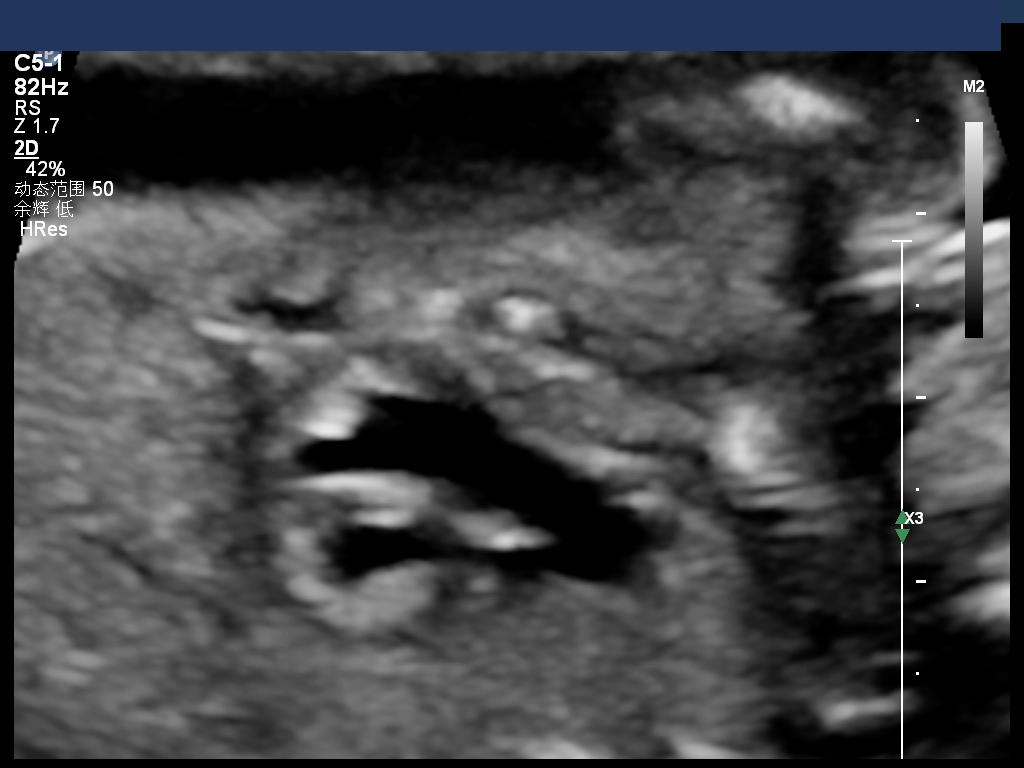

Supplement: S2 Dataset — (ZIP) [file pone.0305250.s002.zip › FE-SD-2/images/test_res/814_ro.jpg]

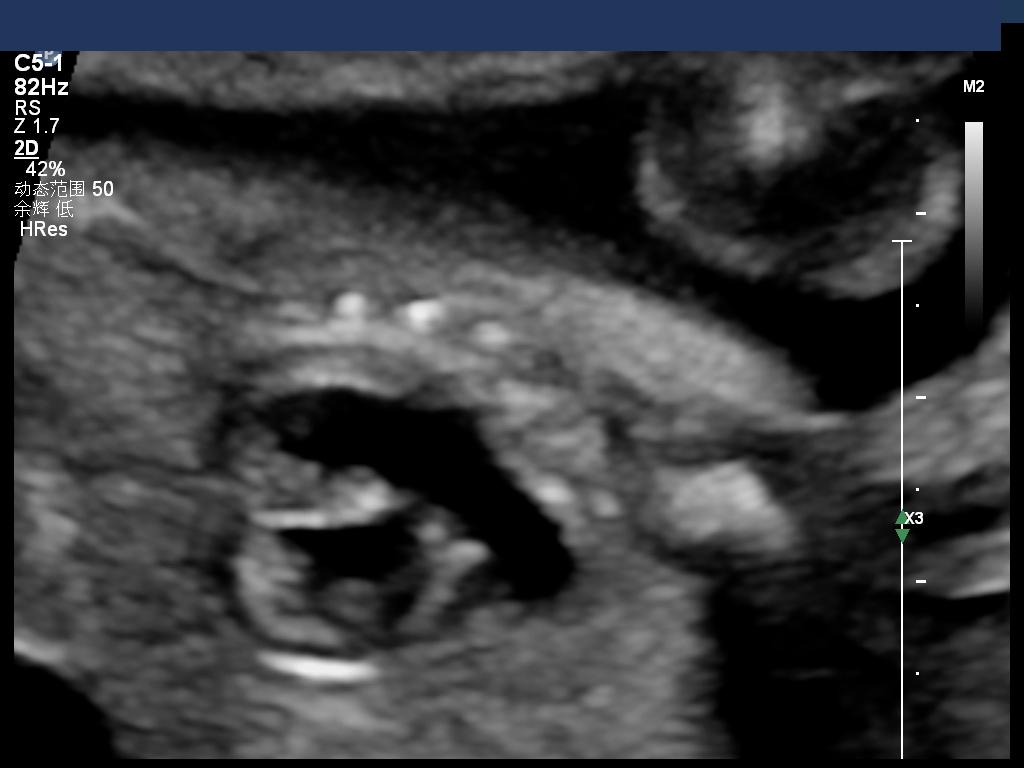

Supplement: S2 Dataset — (ZIP) [file pone.0305250.s002.zip › FE-SD-2/images/test_res/815_ro.jpg]

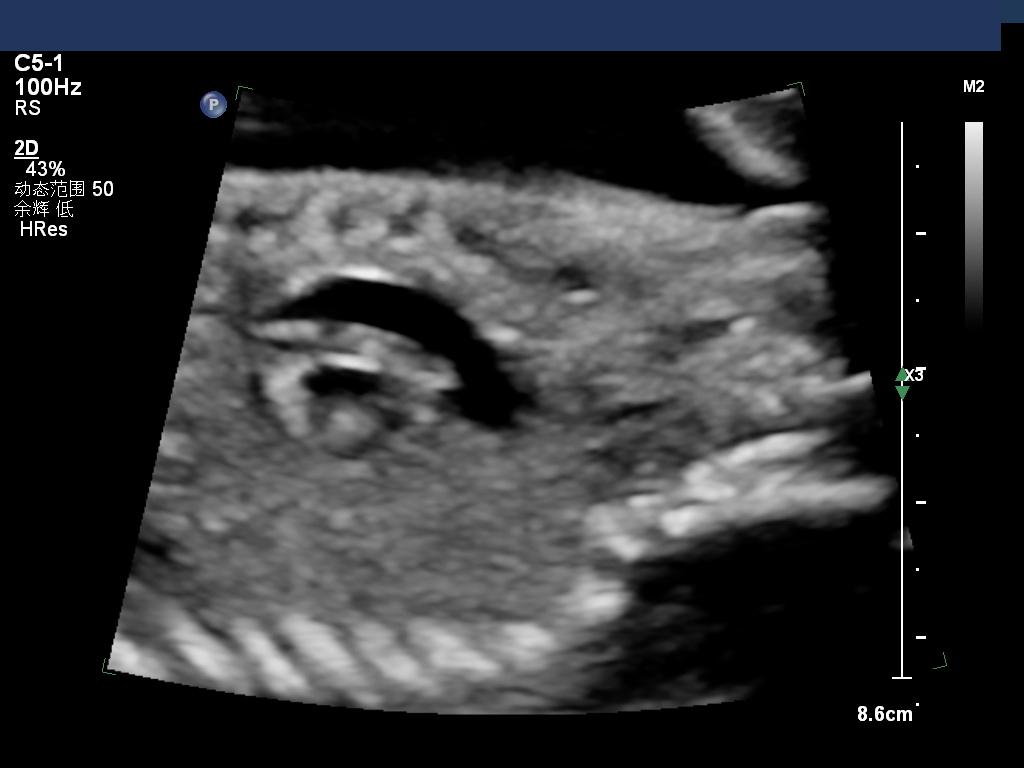

Supplement: S2 Dataset — (ZIP) [file pone.0305250.s002.zip › FE-SD-2/images/test_res/816_ro.jpg]

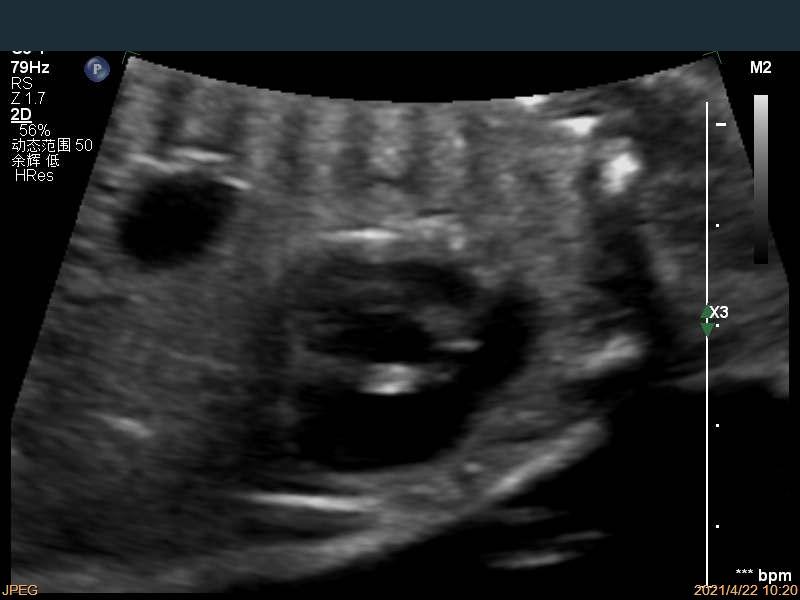

Supplement: S2 Dataset — (ZIP) [file pone.0305250.s002.zip › FE-SD-2/images/test_res/817_ro.jpg]

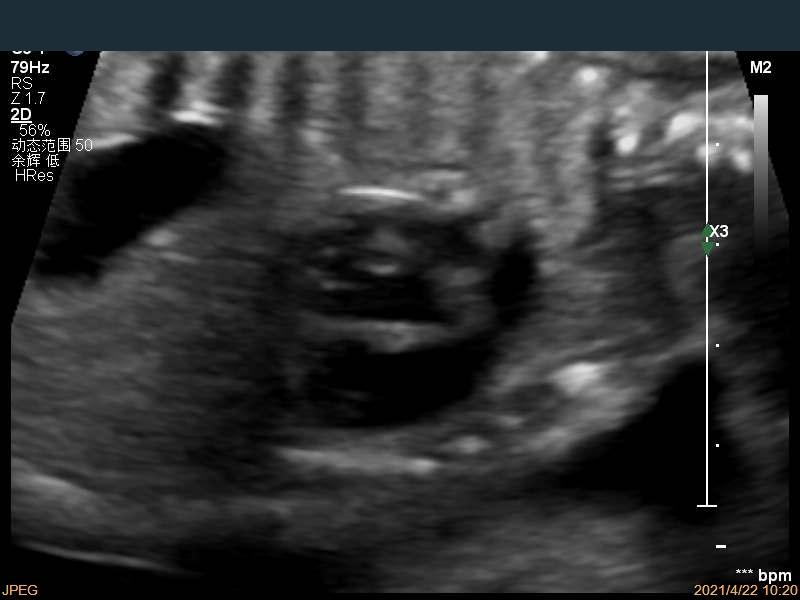

Supplement: S2 Dataset — (ZIP) [file pone.0305250.s002.zip › FE-SD-2/images/test_res/819_ro.jpg]

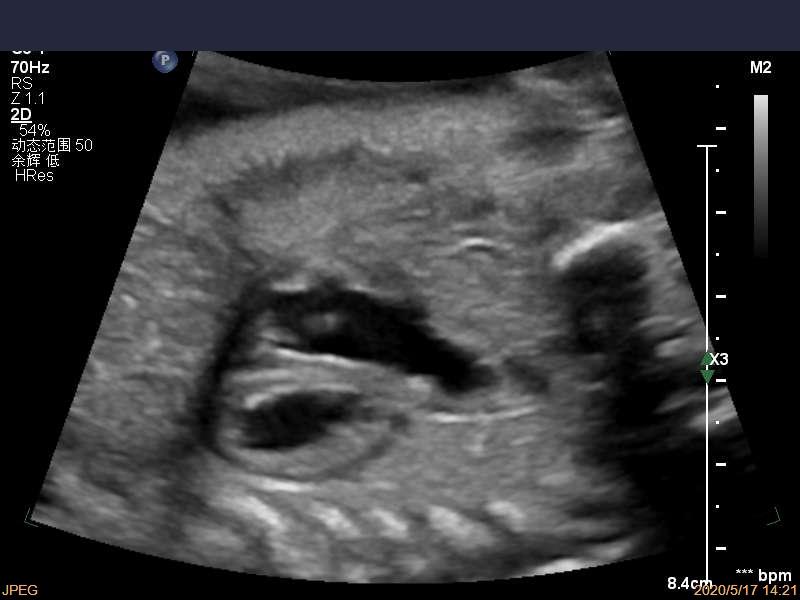

Supplement: S2 Dataset — (ZIP) [file pone.0305250.s002.zip › FE-SD-2/images/test_res/821_ro.jpg]
