# Supplementary figures and images for: Implementation of resource-efficient fetal echocardiography detection algorithms in edge computing (part 3 of 4)
Source: PLoS One. 2024 Sep 23;19(9):e0305250. doi: 10.1371/journal.pone.0305250 (PMC11419364; doi:10.1371/journal.pone.0305250)

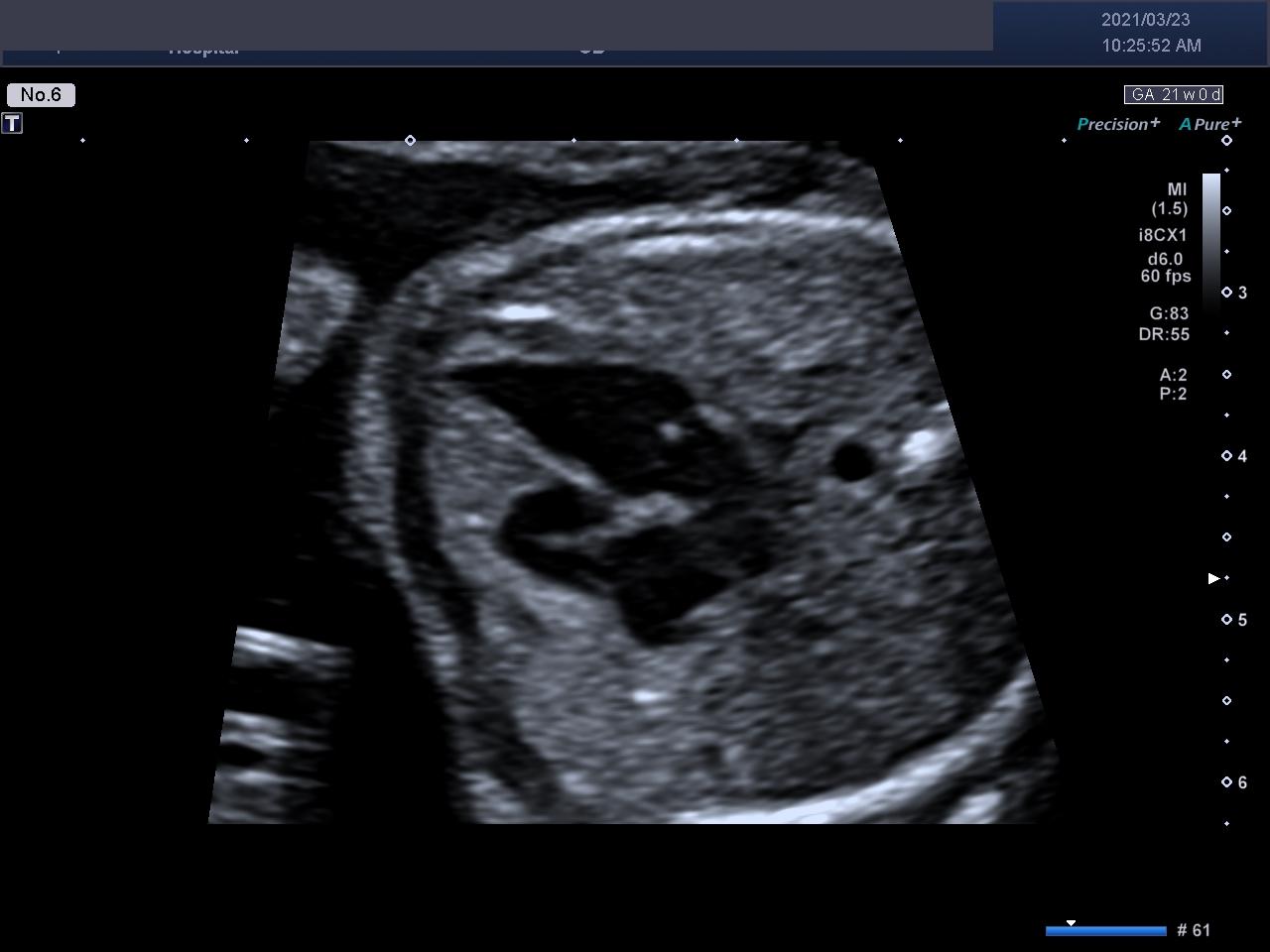

Supplement: S2 Dataset — (ZIP) [file pone.0305250.s002.zip › FE-SD-2/images/test_res/1005_fc.jpg]

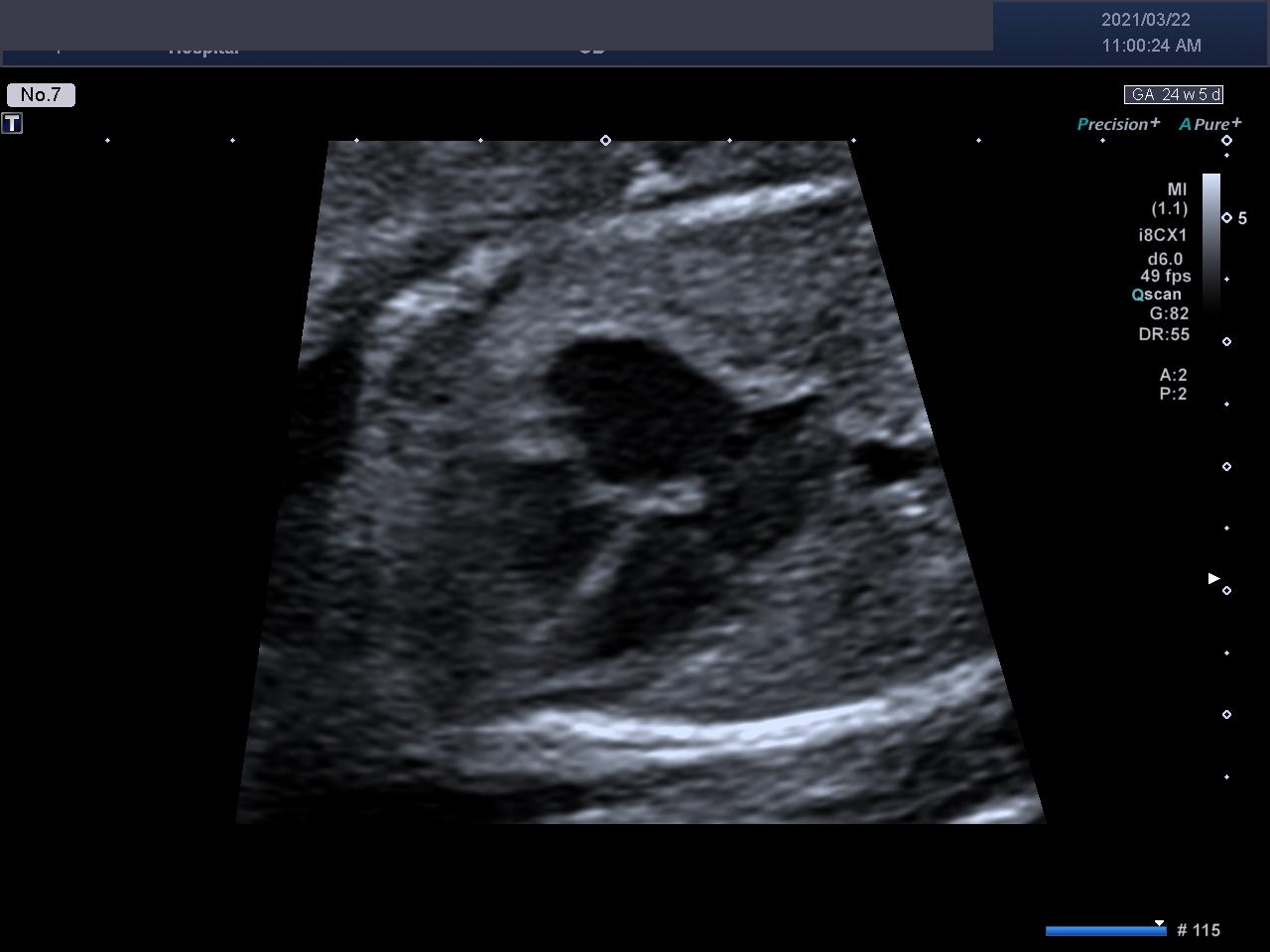

Supplement: S2 Dataset — (ZIP) [file pone.0305250.s002.zip › FE-SD-2/images/test_res/1006_fc.jpg]

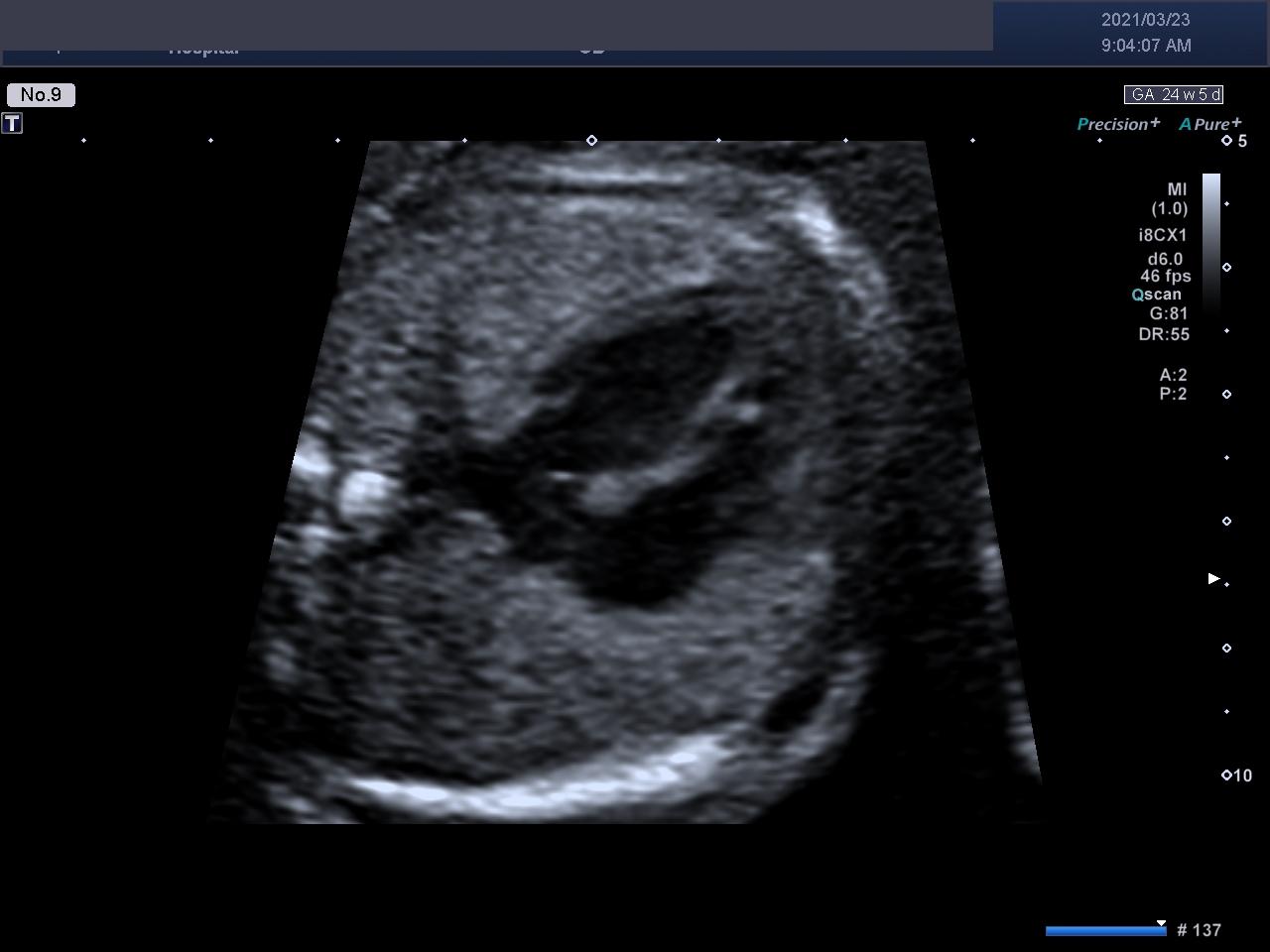

Supplement: S2 Dataset — (ZIP) [file pone.0305250.s002.zip › FE-SD-2/images/test_res/1007_fc.jpg]

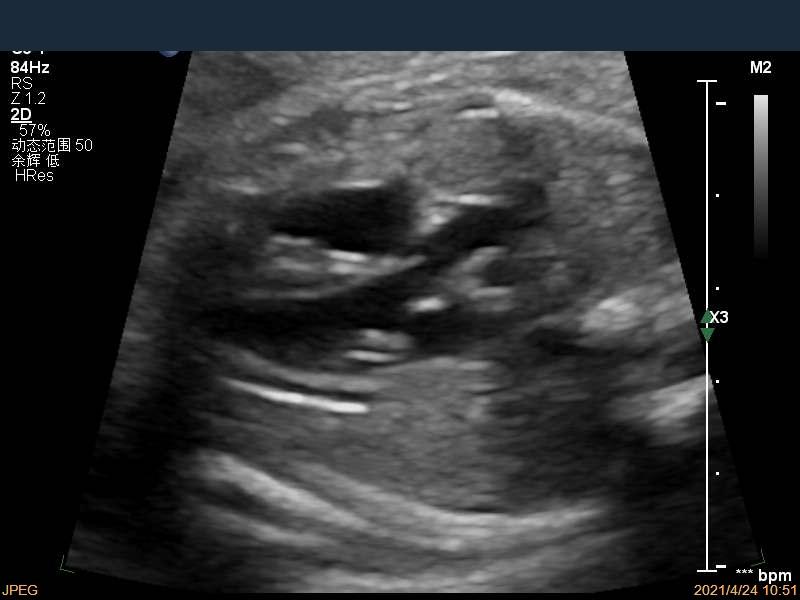

Supplement: S2 Dataset — (ZIP) [file pone.0305250.s002.zip › FE-SD-2/images/test_res/1009_lo.jpg]

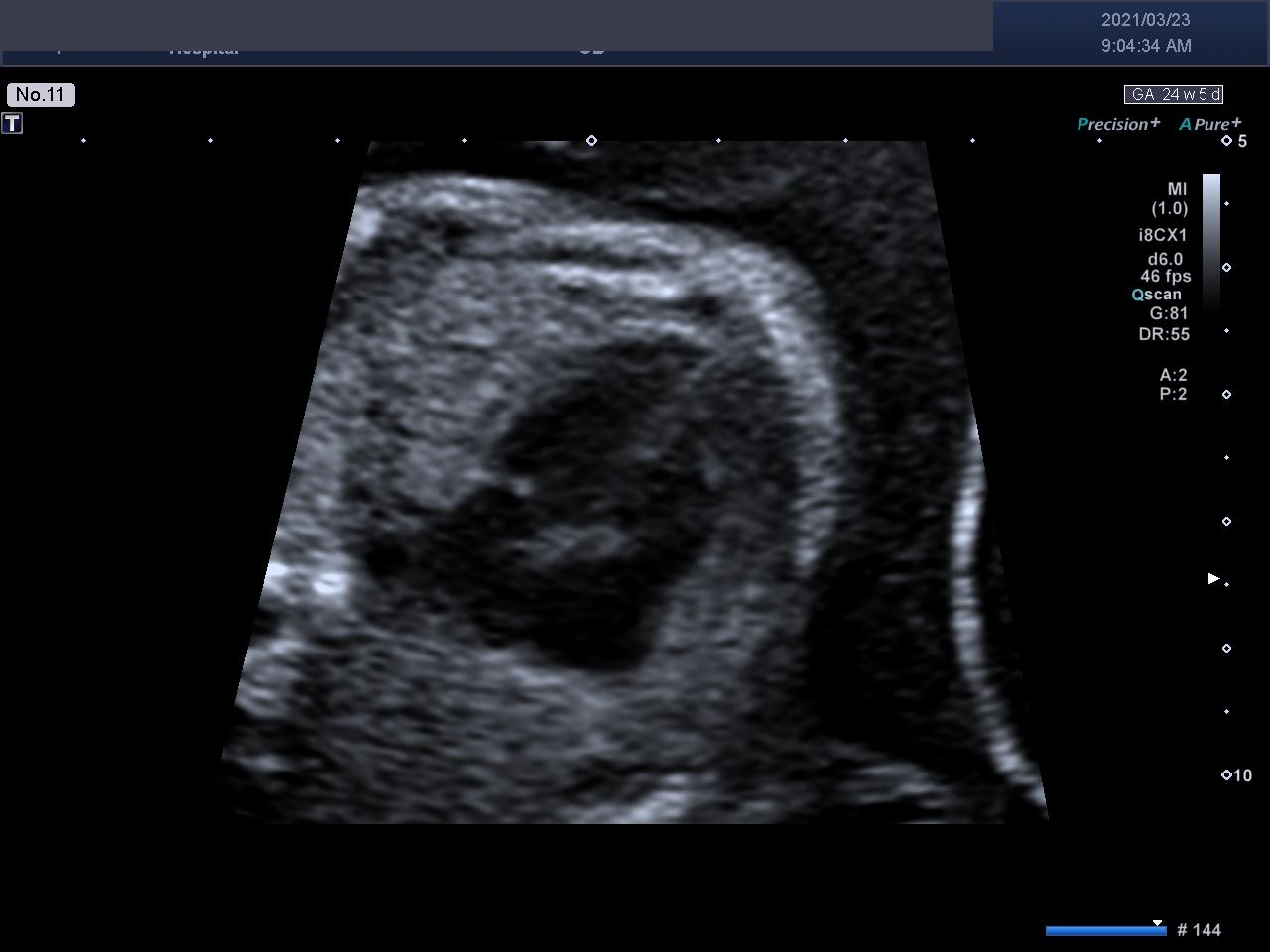

Supplement: S2 Dataset — (ZIP) [file pone.0305250.s002.zip › FE-SD-2/images/test_res/1010_fc.jpg]

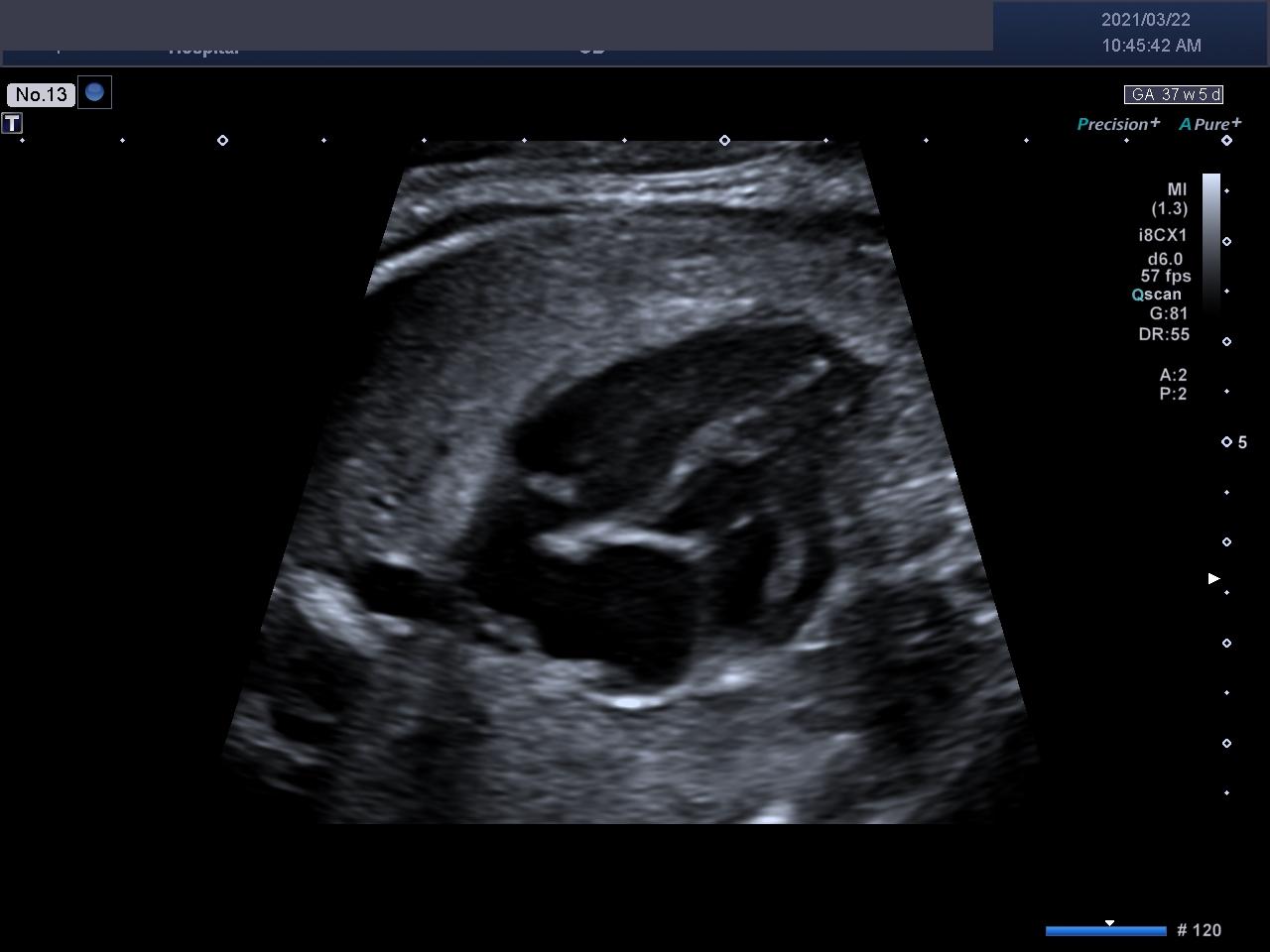

Supplement: S2 Dataset — (ZIP) [file pone.0305250.s002.zip › FE-SD-2/images/test_res/1011_fc.jpg]

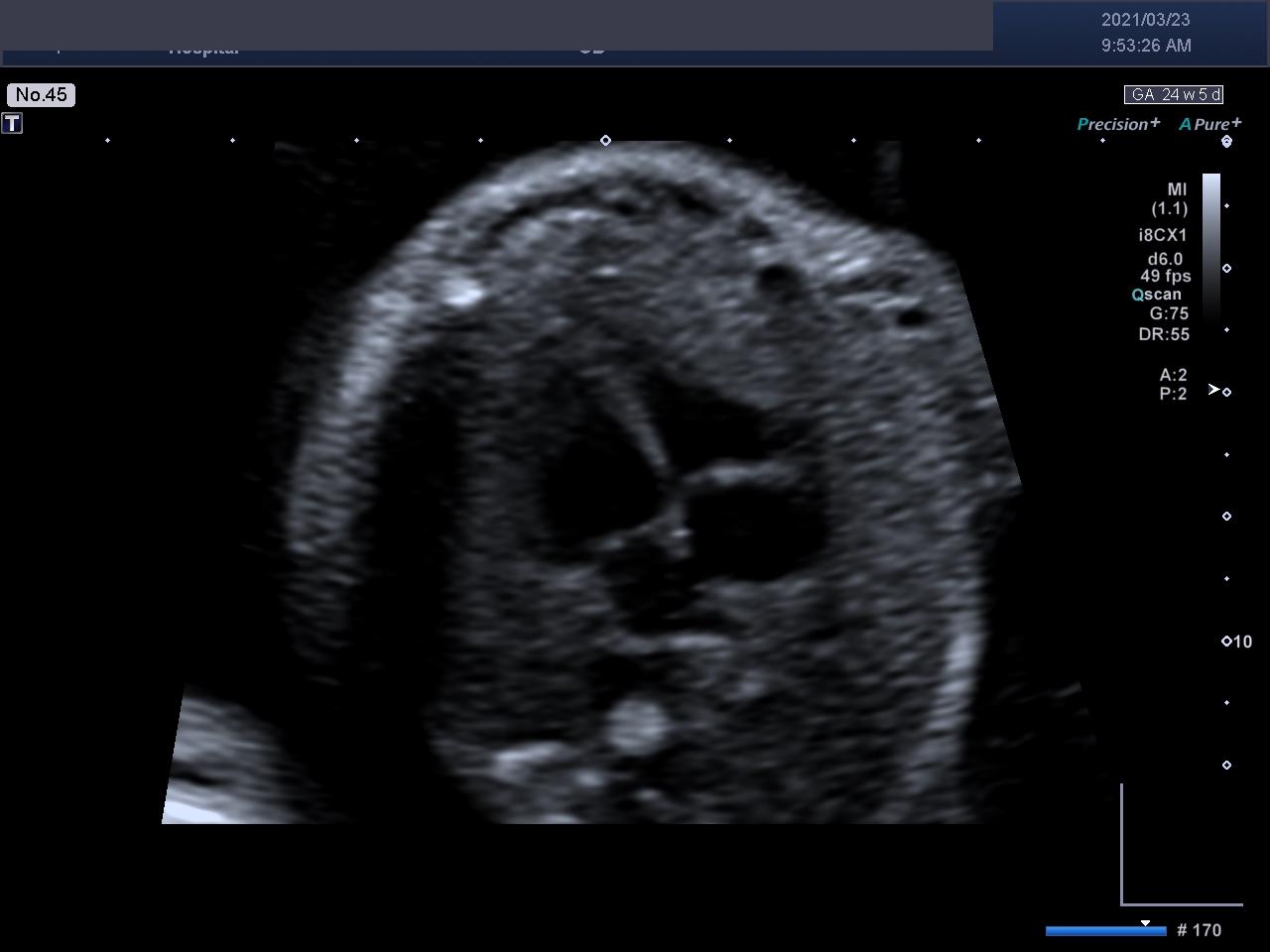

Supplement: S2 Dataset — (ZIP) [file pone.0305250.s002.zip › FE-SD-2/images/test_res/1013_fc.jpg]

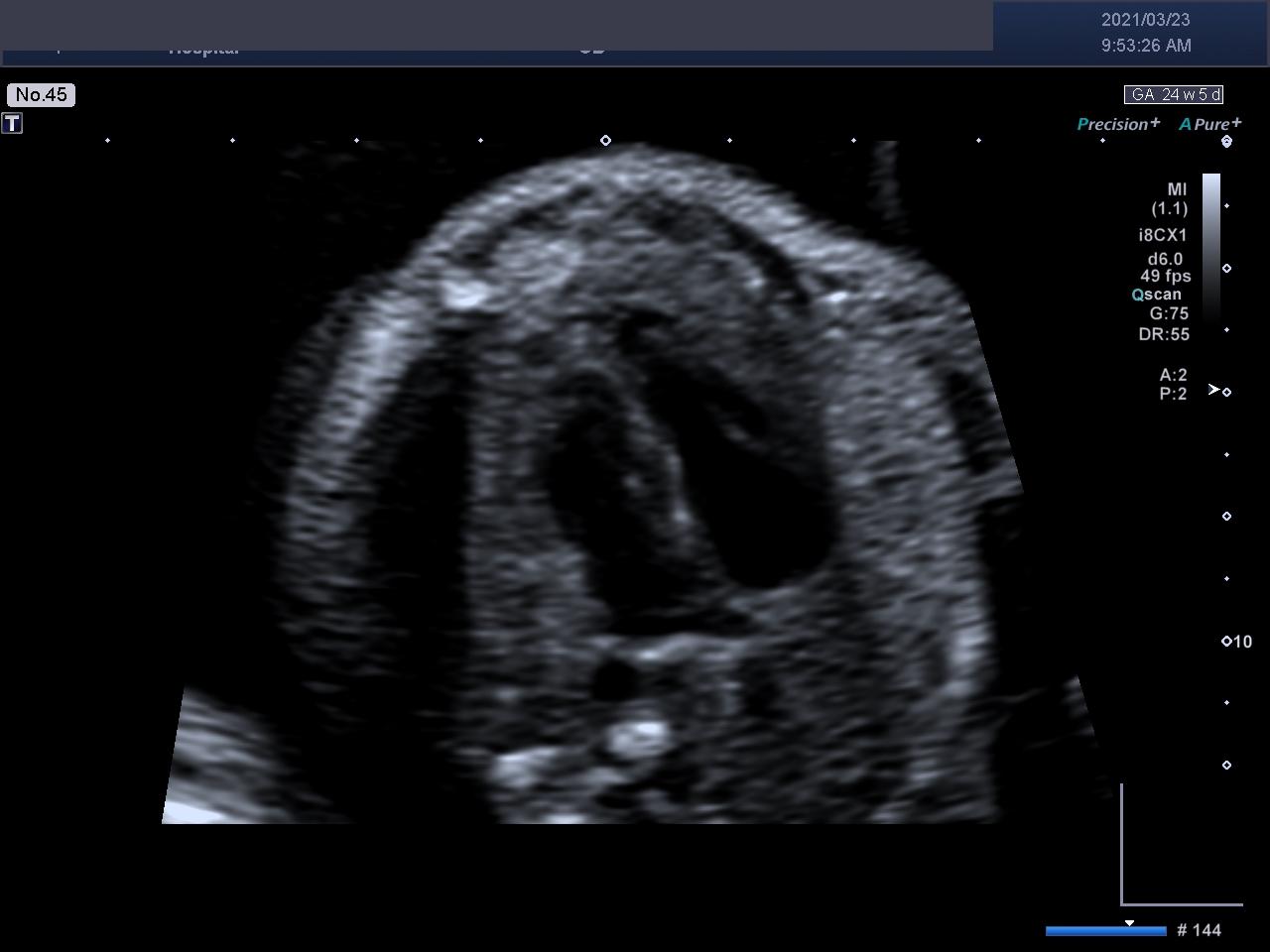

Supplement: S2 Dataset — (ZIP) [file pone.0305250.s002.zip › FE-SD-2/images/test_res/1015_fc.jpg]

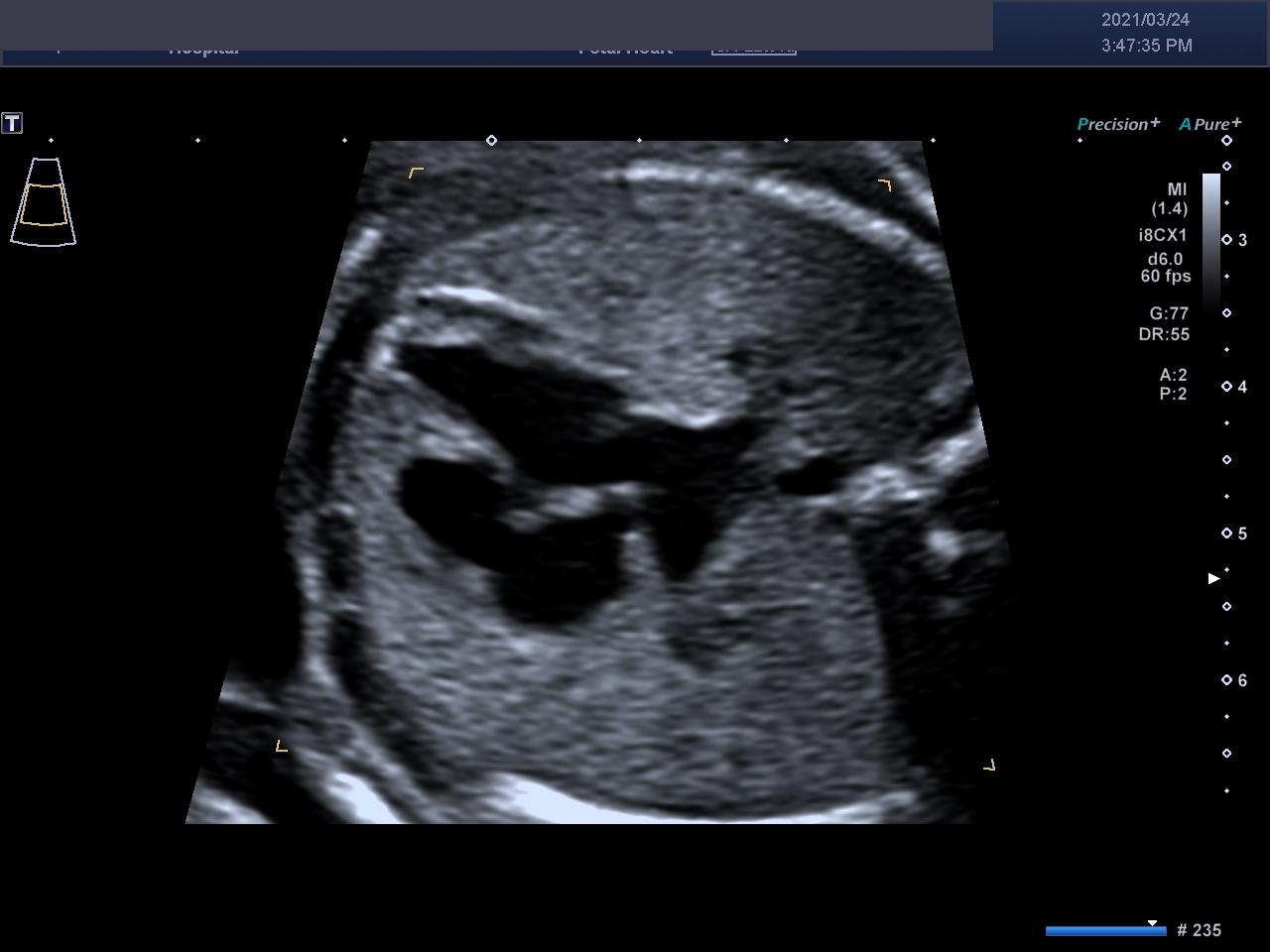

Supplement: S2 Dataset — (ZIP) [file pone.0305250.s002.zip › FE-SD-2/images/test_res/1016_fc.jpg]

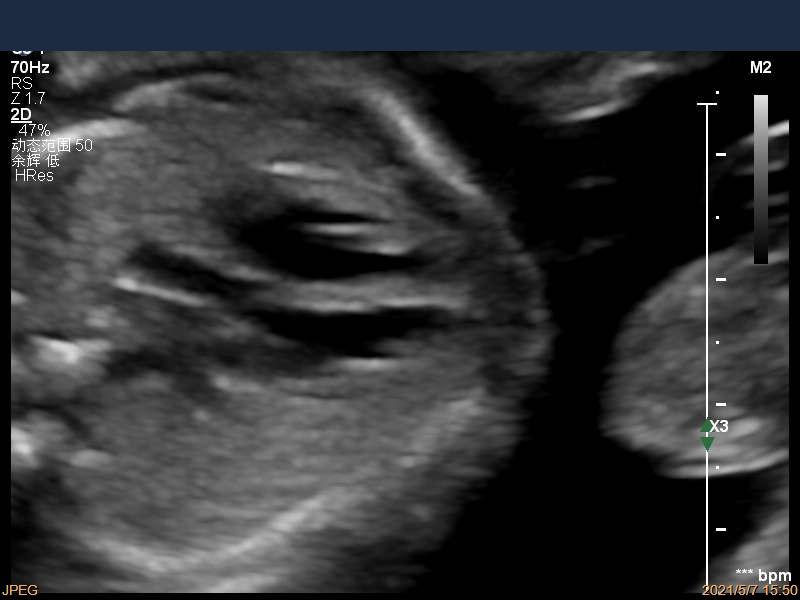

Supplement: S2 Dataset — (ZIP) [file pone.0305250.s002.zip › FE-SD-2/images/test_res/1021_lo.jpg]

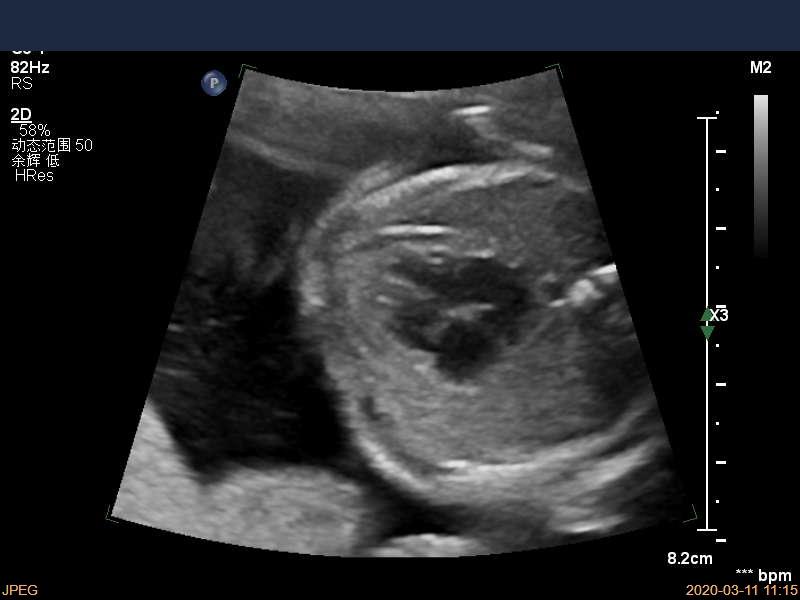

Supplement: S2 Dataset — (ZIP) [file pone.0305250.s002.zip › FE-SD-2/images/test_res/1091_fc.jpg]

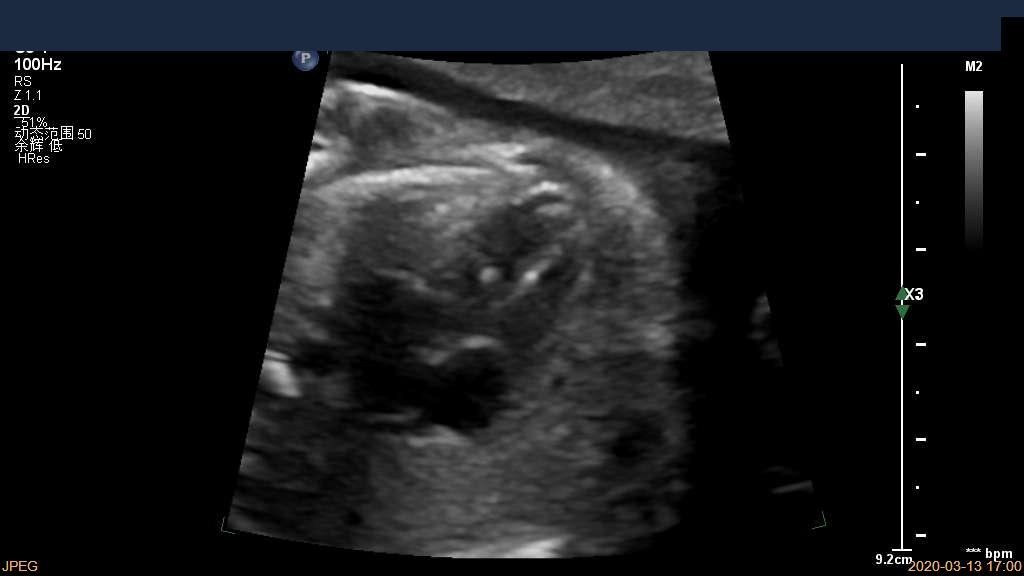

Supplement: S2 Dataset — (ZIP) [file pone.0305250.s002.zip › FE-SD-2/images/test_res/1092_fc.jpg]

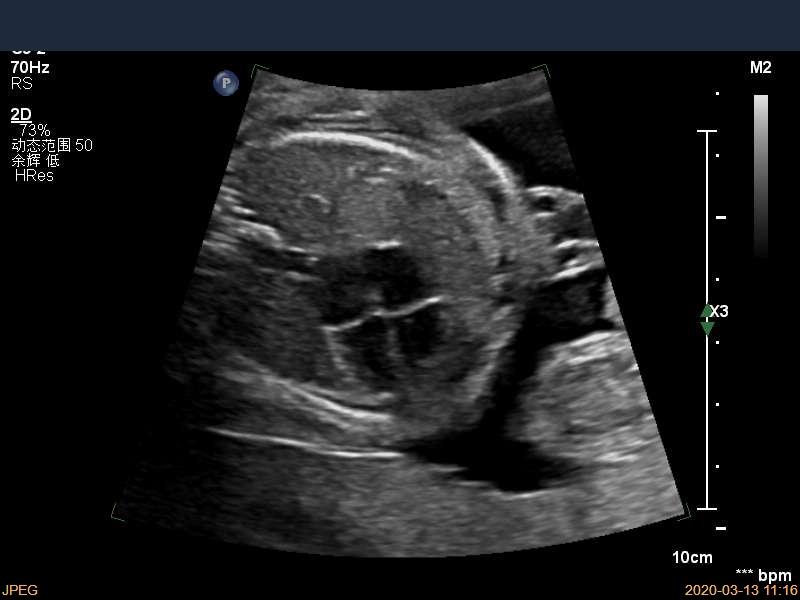

Supplement: S2 Dataset — (ZIP) [file pone.0305250.s002.zip › FE-SD-2/images/test_res/1093_fc.jpg]

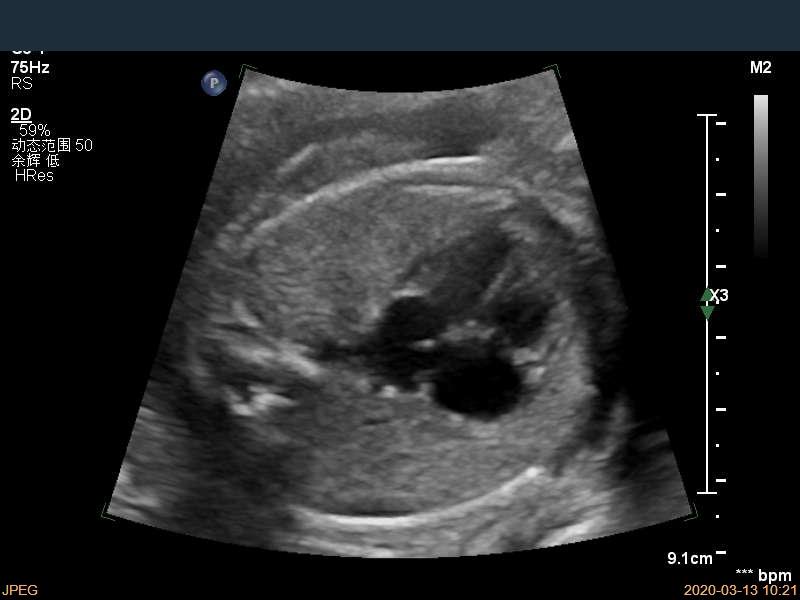

Supplement: S2 Dataset — (ZIP) [file pone.0305250.s002.zip › FE-SD-2/images/test_res/1096_fc.jpg]

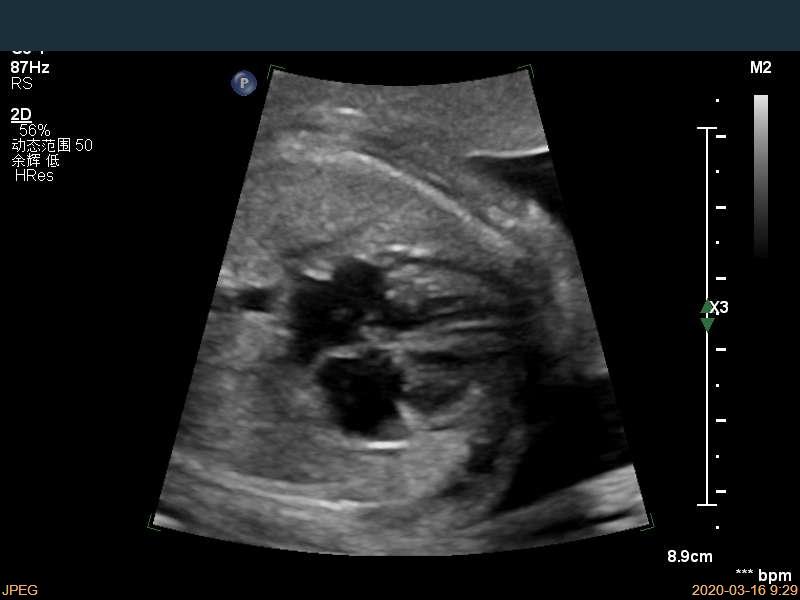

Supplement: S2 Dataset — (ZIP) [file pone.0305250.s002.zip › FE-SD-2/images/test_res/1098_fc.jpg]

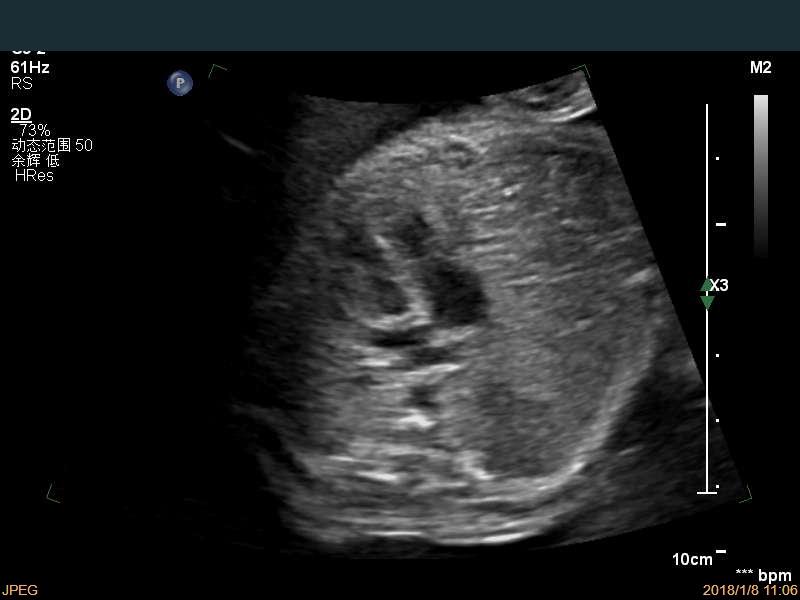

Supplement: S2 Dataset — (ZIP) [file pone.0305250.s002.zip › FE-SD-2/images/test_res/1101_fc.jpg]

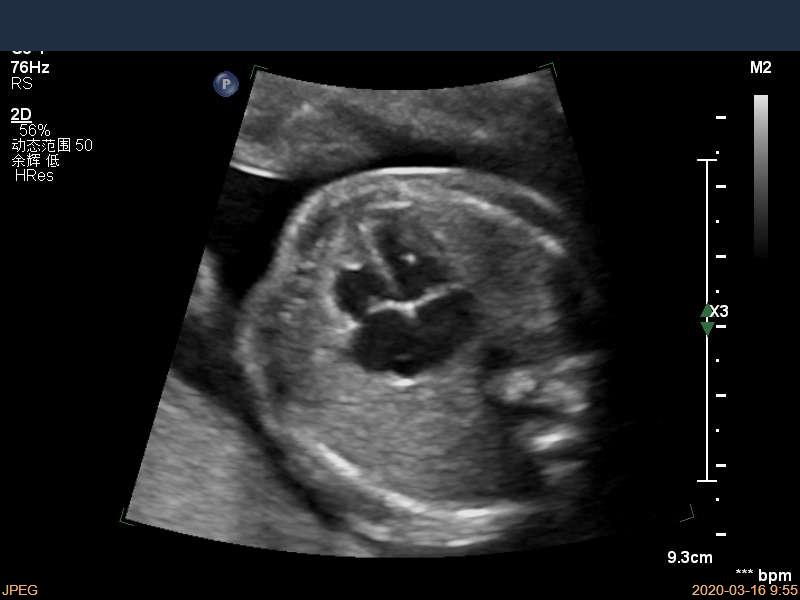

Supplement: S2 Dataset — (ZIP) [file pone.0305250.s002.zip › FE-SD-2/images/test_res/1104_fc.jpg]

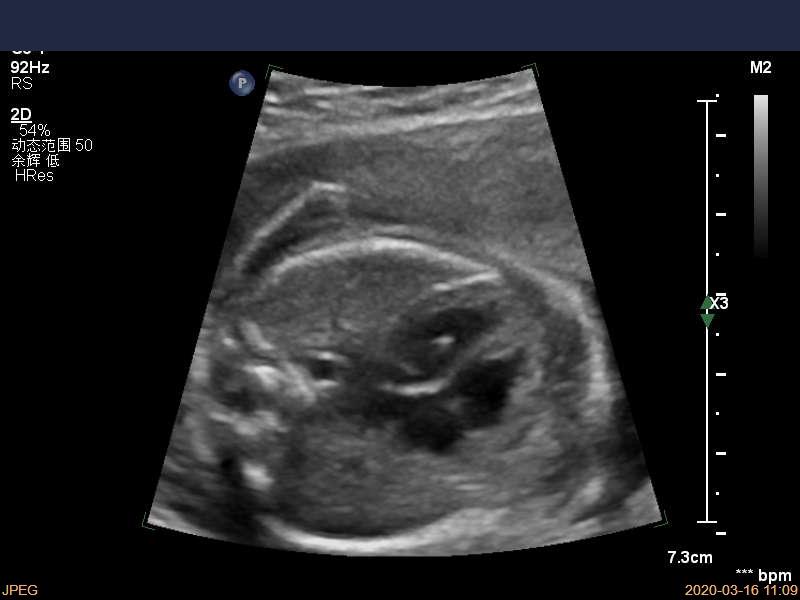

Supplement: S2 Dataset — (ZIP) [file pone.0305250.s002.zip › FE-SD-2/images/test_res/1106_fc.jpg]

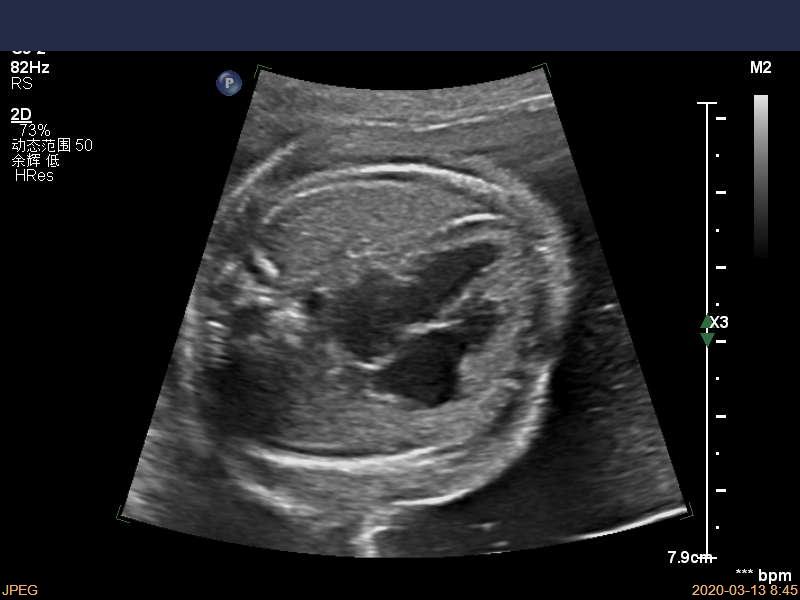

Supplement: S2 Dataset — (ZIP) [file pone.0305250.s002.zip › FE-SD-2/images/test_res/1109_fc.jpg]

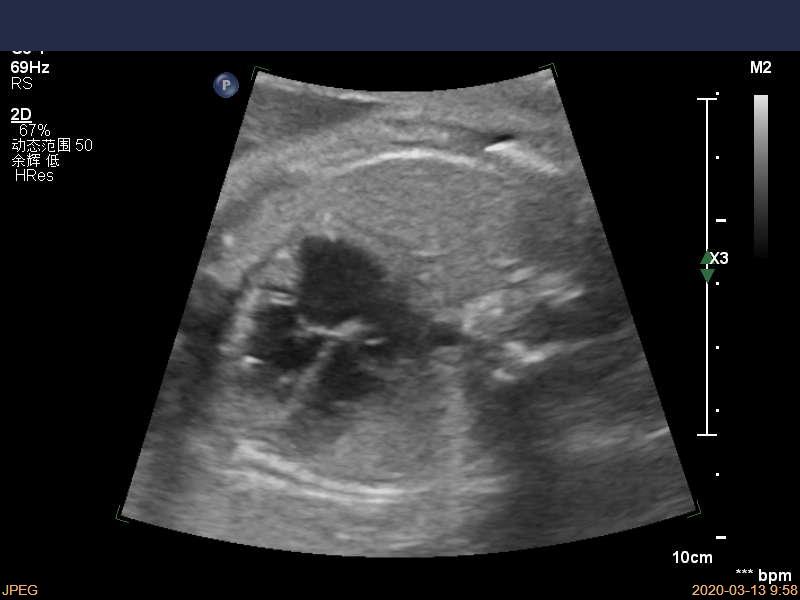

Supplement: S2 Dataset — (ZIP) [file pone.0305250.s002.zip › FE-SD-2/images/test_res/1111_fc.jpg]

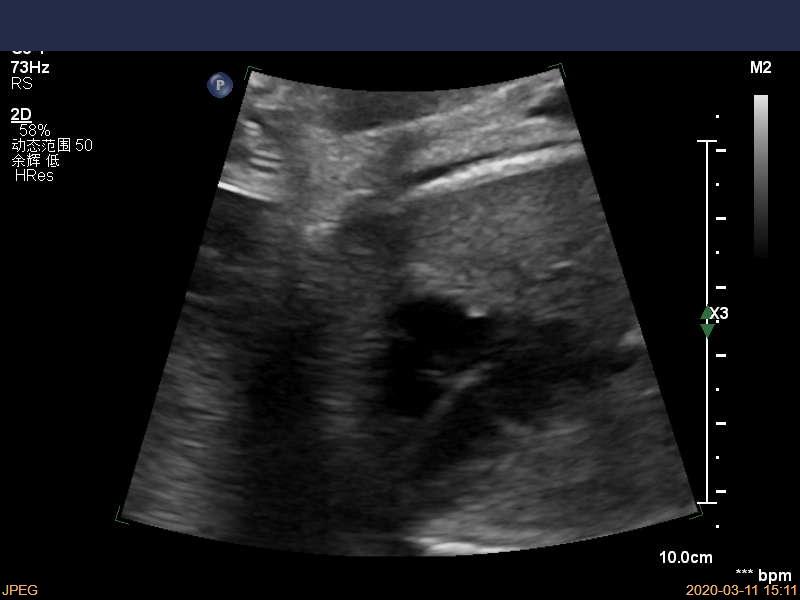

Supplement: S2 Dataset — (ZIP) [file pone.0305250.s002.zip › FE-SD-2/images/test_res/1113_fc.jpg]

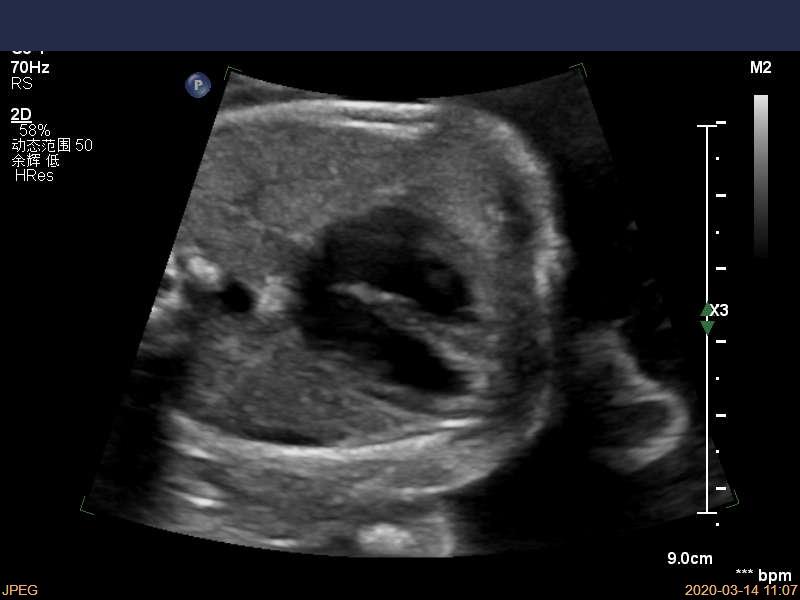

Supplement: S2 Dataset — (ZIP) [file pone.0305250.s002.zip › FE-SD-2/images/test_res/1114_fc.jpg]

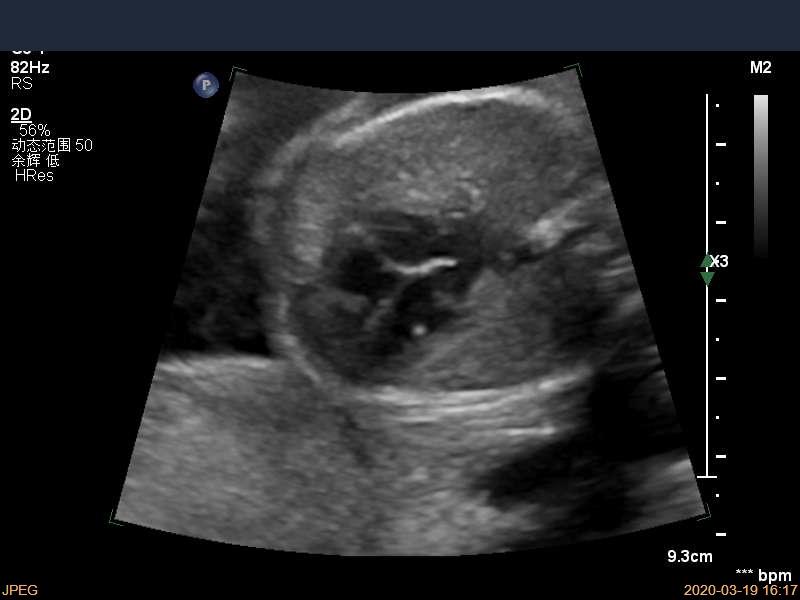

Supplement: S2 Dataset — (ZIP) [file pone.0305250.s002.zip › FE-SD-2/images/test_res/1116_fc.jpg]

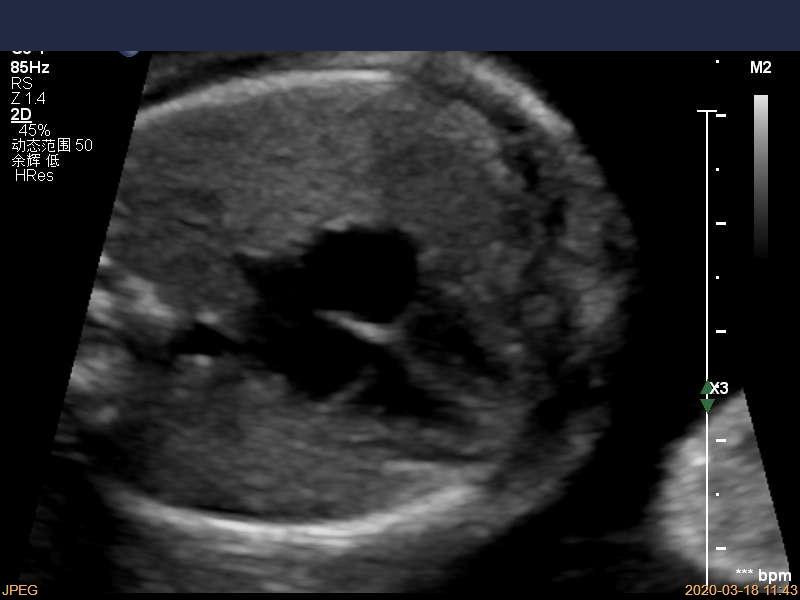

Supplement: S2 Dataset — (ZIP) [file pone.0305250.s002.zip › FE-SD-2/images/test_res/1120_fc.jpg]

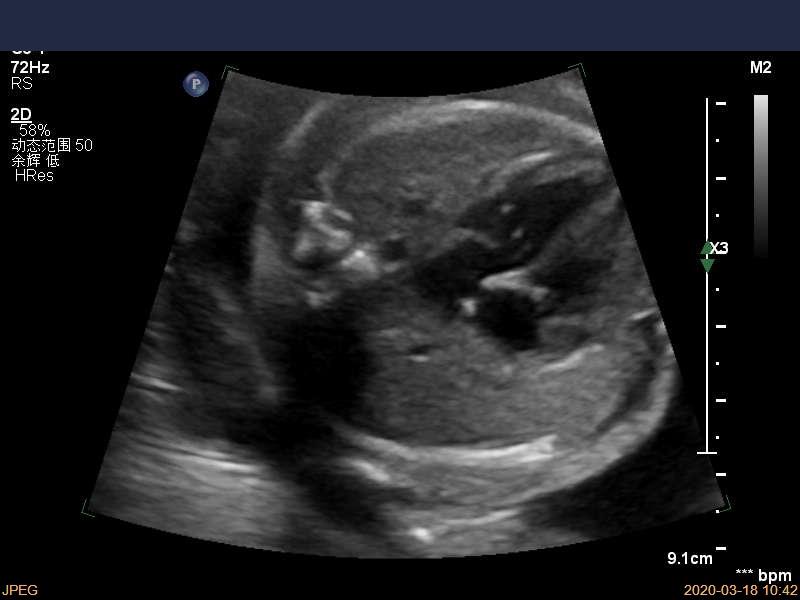

Supplement: S2 Dataset — (ZIP) [file pone.0305250.s002.zip › FE-SD-2/images/test_res/1123_fc.jpg]

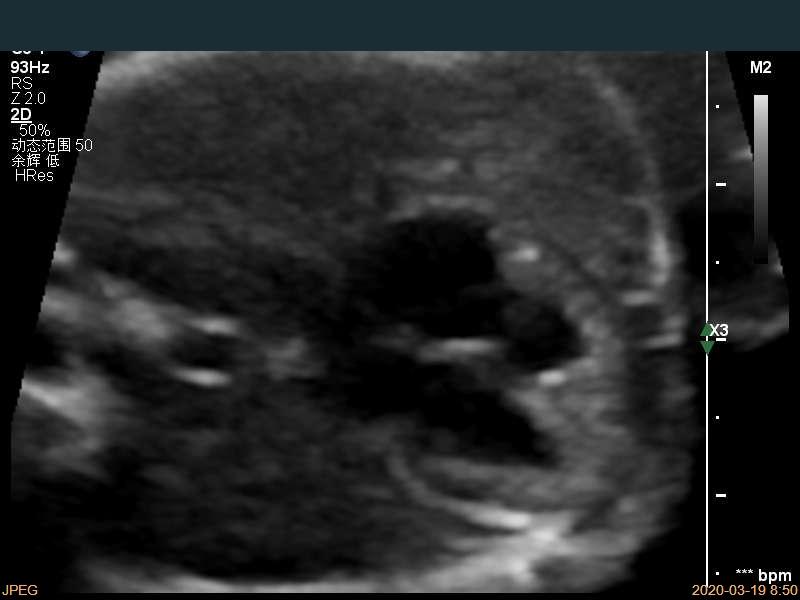

Supplement: S2 Dataset — (ZIP) [file pone.0305250.s002.zip › FE-SD-2/images/test_res/1127_fc.jpg]

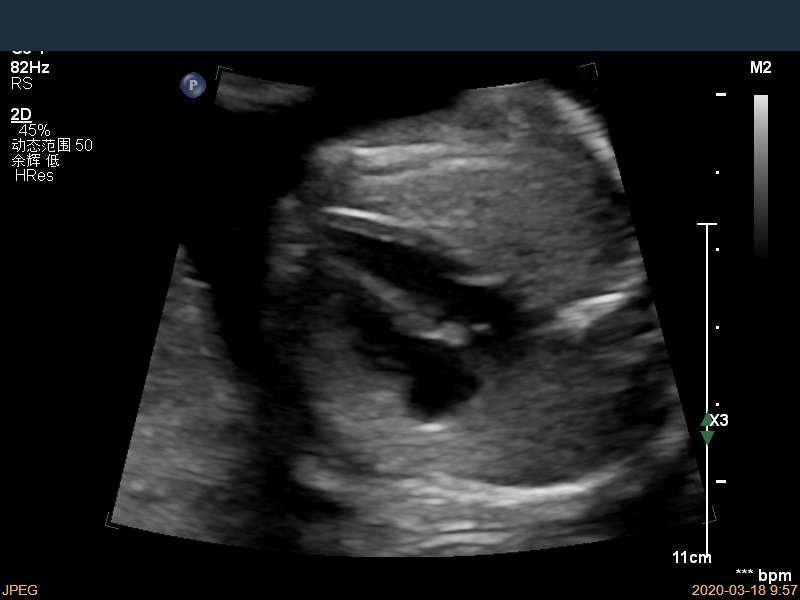

Supplement: S2 Dataset — (ZIP) [file pone.0305250.s002.zip › FE-SD-2/images/test_res/1128_fc.jpg]

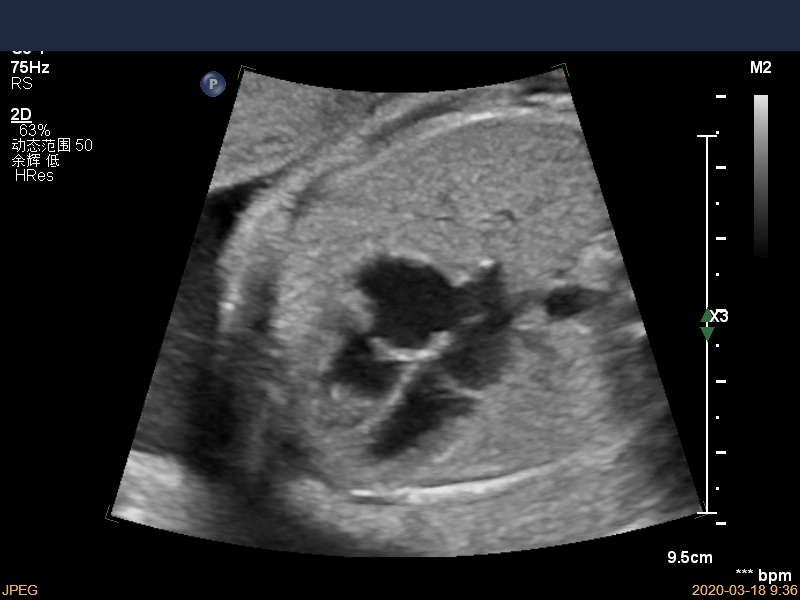

Supplement: S2 Dataset — (ZIP) [file pone.0305250.s002.zip › FE-SD-2/images/test_res/1129_fc.jpg]

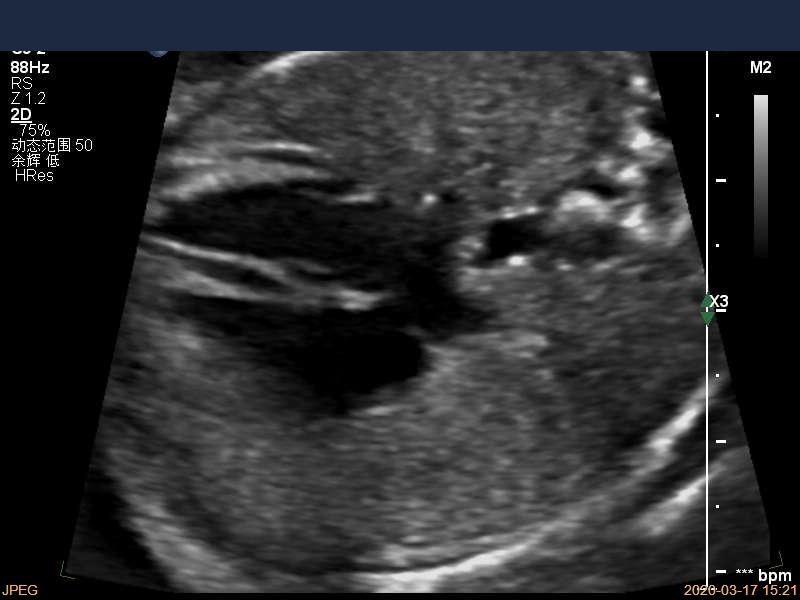

Supplement: S2 Dataset — (ZIP) [file pone.0305250.s002.zip › FE-SD-2/images/test_res/1131_fc.jpg]

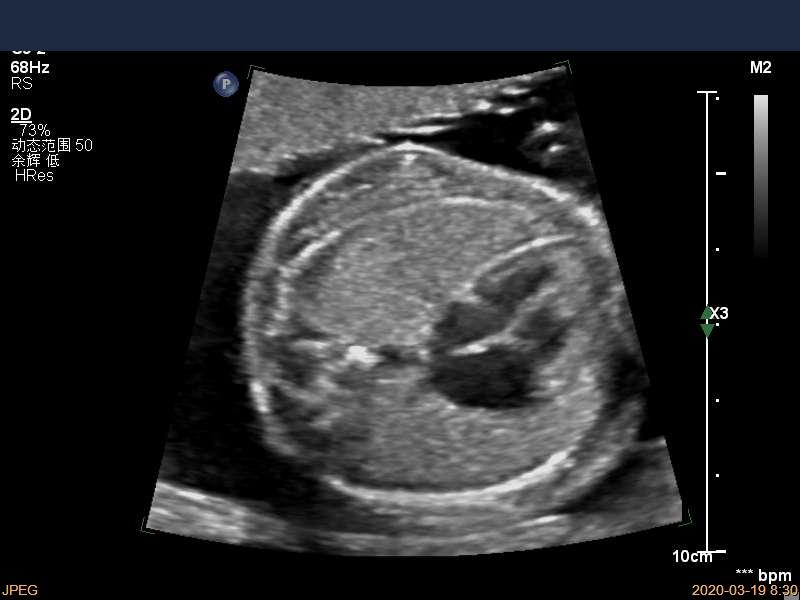

Supplement: S2 Dataset — (ZIP) [file pone.0305250.s002.zip › FE-SD-2/images/test_res/1132_fc.jpg]

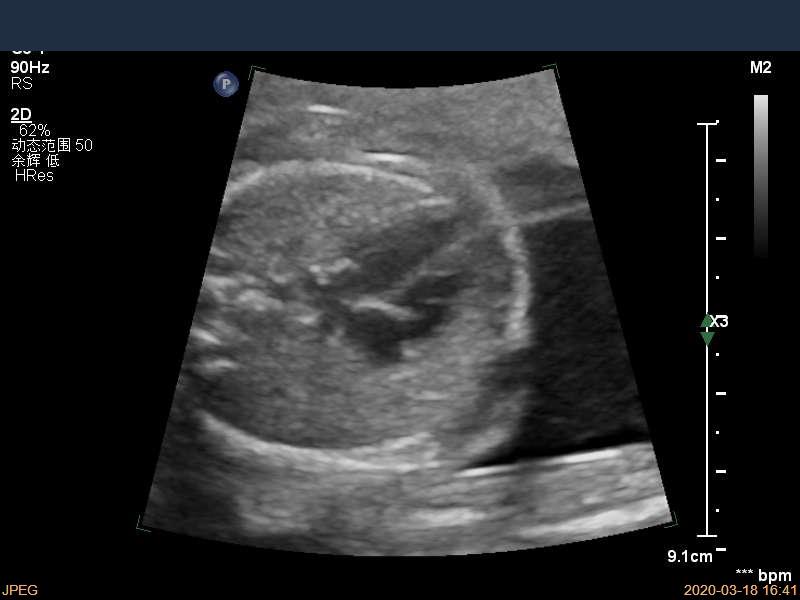

Supplement: S2 Dataset — (ZIP) [file pone.0305250.s002.zip › FE-SD-2/images/test_res/1133_fc.jpg]

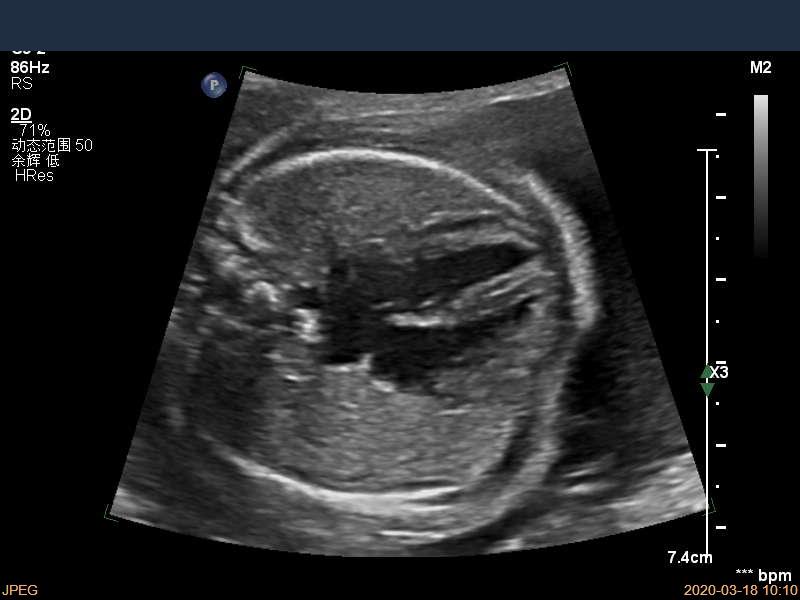

Supplement: S2 Dataset — (ZIP) [file pone.0305250.s002.zip › FE-SD-2/images/test_res/1134_fc.jpg]

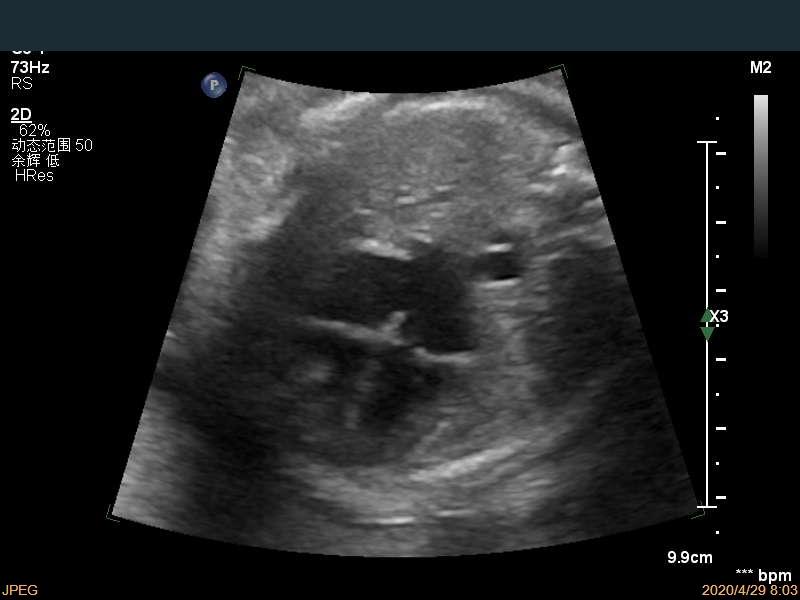

Supplement: S2 Dataset — (ZIP) [file pone.0305250.s002.zip › FE-SD-2/images/test_res/1138_fc.jpg]

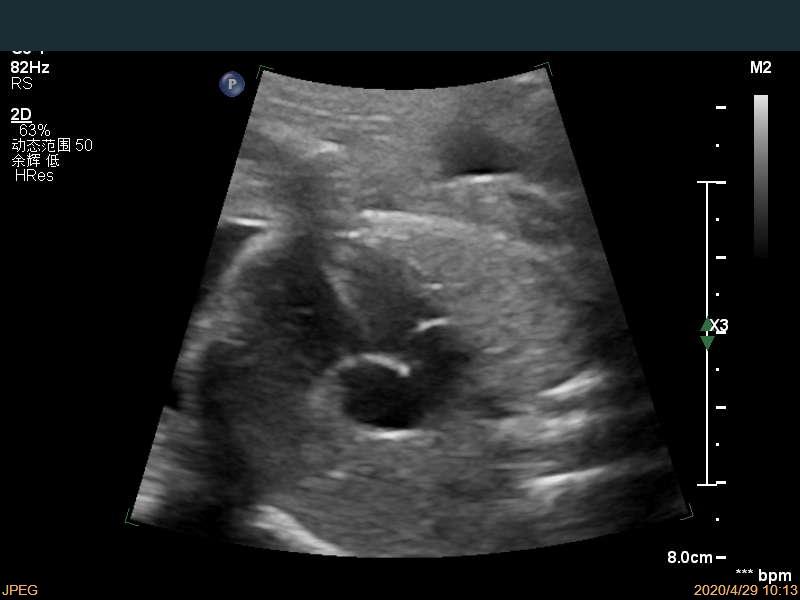

Supplement: S2 Dataset — (ZIP) [file pone.0305250.s002.zip › FE-SD-2/images/test_res/1139_fc.jpg]

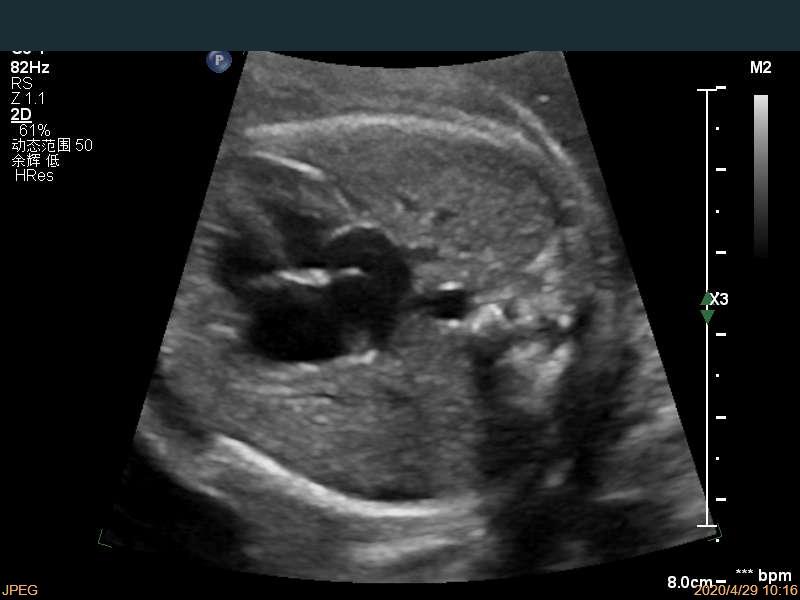

Supplement: S2 Dataset — (ZIP) [file pone.0305250.s002.zip › FE-SD-2/images/test_res/1143_fc.jpg]

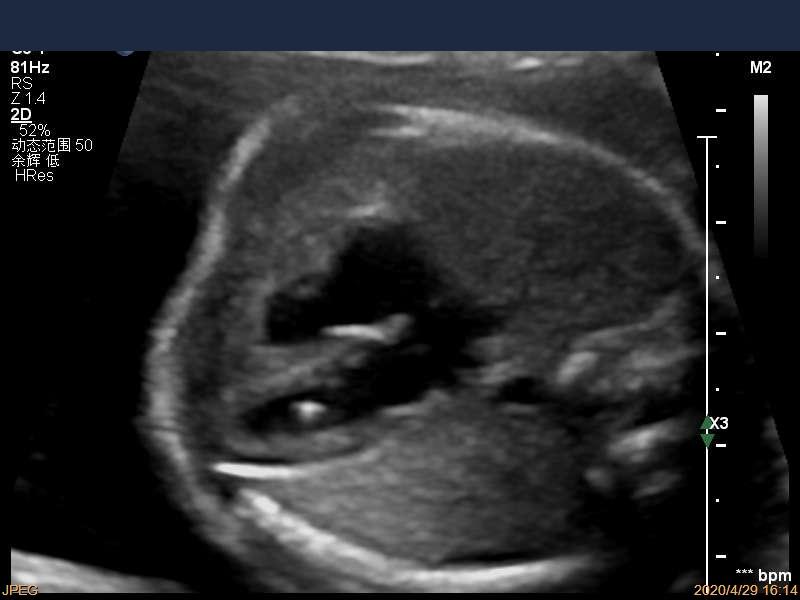

Supplement: S2 Dataset — (ZIP) [file pone.0305250.s002.zip › FE-SD-2/images/test_res/1144_fc.jpg]

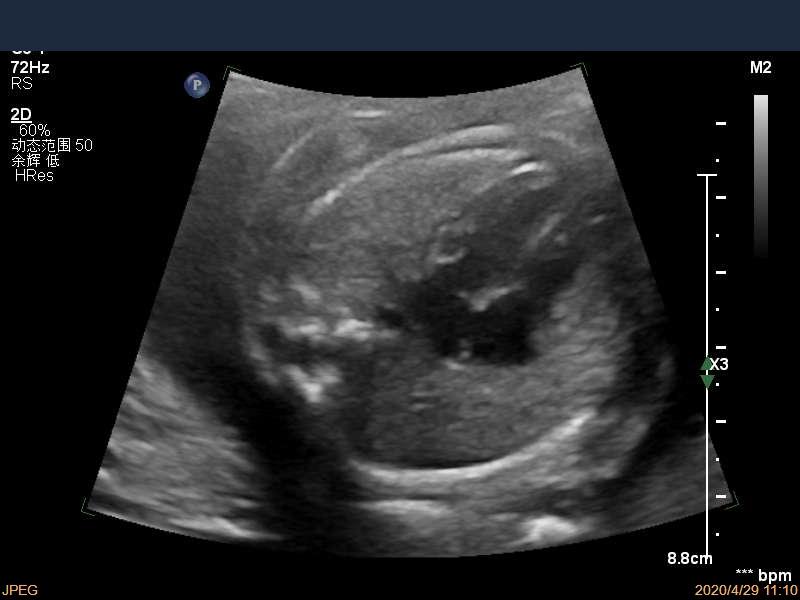

Supplement: S2 Dataset — (ZIP) [file pone.0305250.s002.zip › FE-SD-2/images/test_res/1145_fc.jpg]

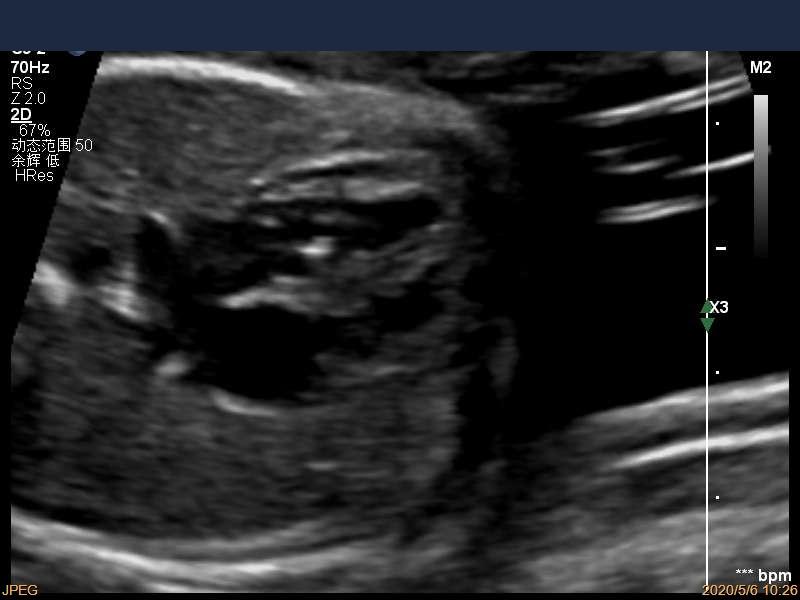

Supplement: S2 Dataset — (ZIP) [file pone.0305250.s002.zip › FE-SD-2/images/test_res/1146_fc.jpg]

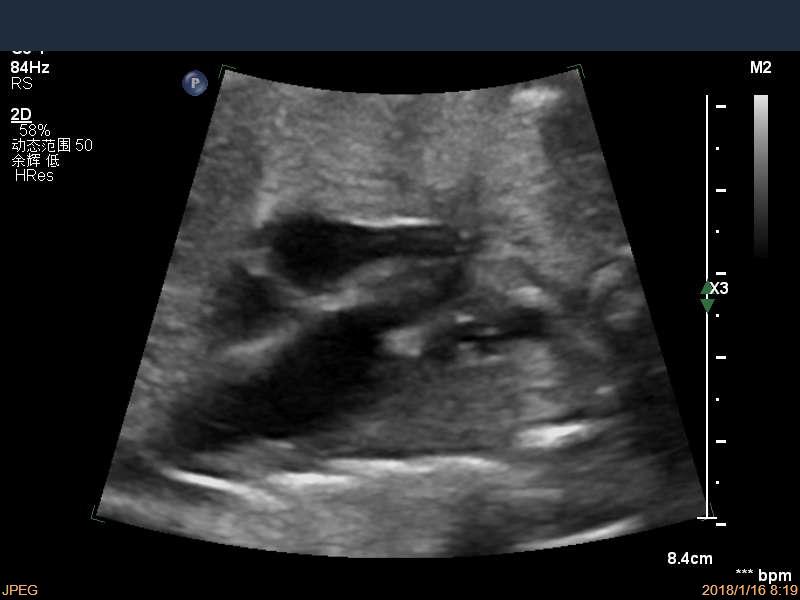

Supplement: S2 Dataset — (ZIP) [file pone.0305250.s002.zip › FE-SD-2/images/test_res/1333_fc.jpg]

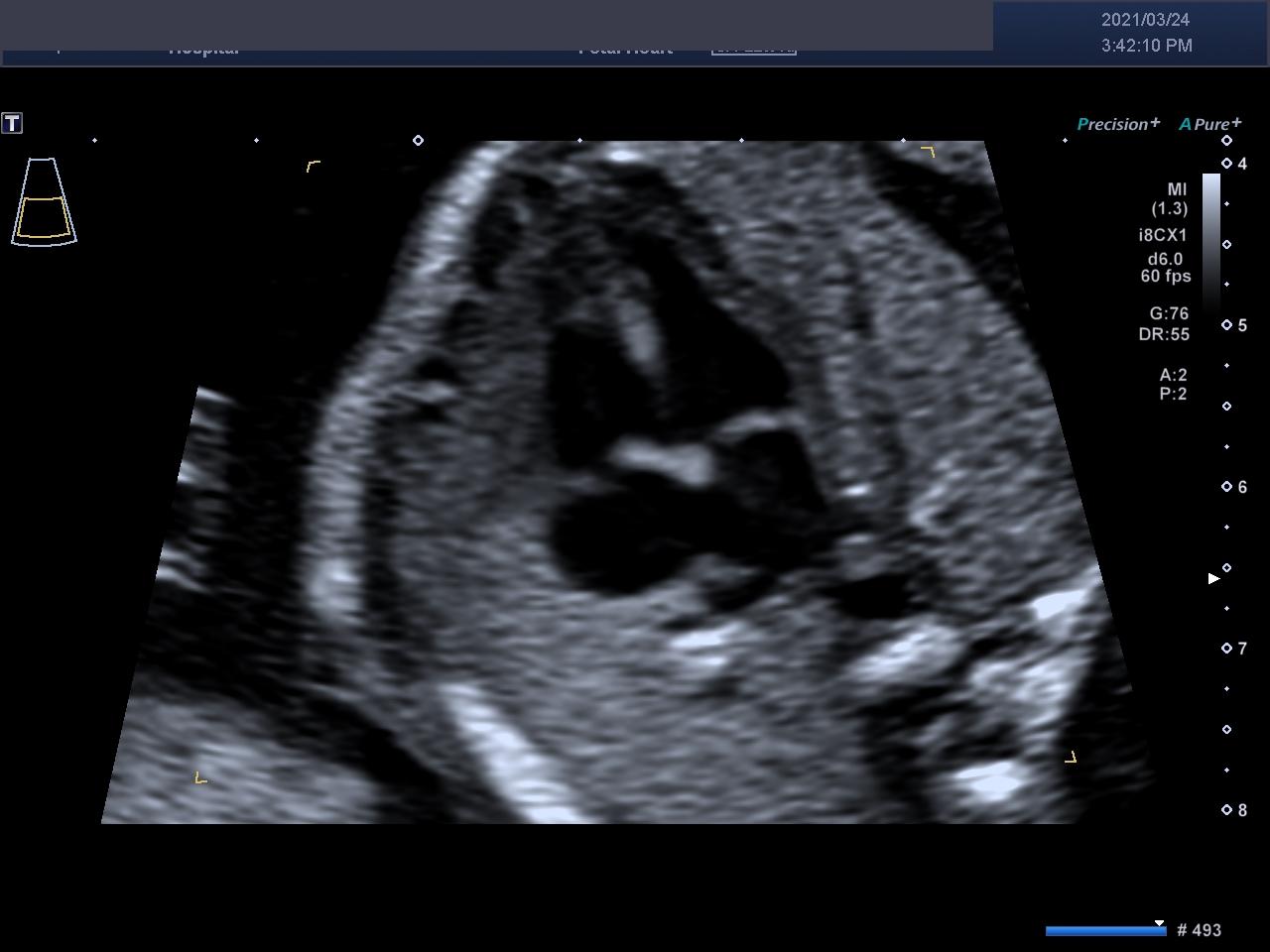

Supplement: S2 Dataset — (ZIP) [file pone.0305250.s002.zip › FE-SD-2/images/test_res/1358_fc.jpg]

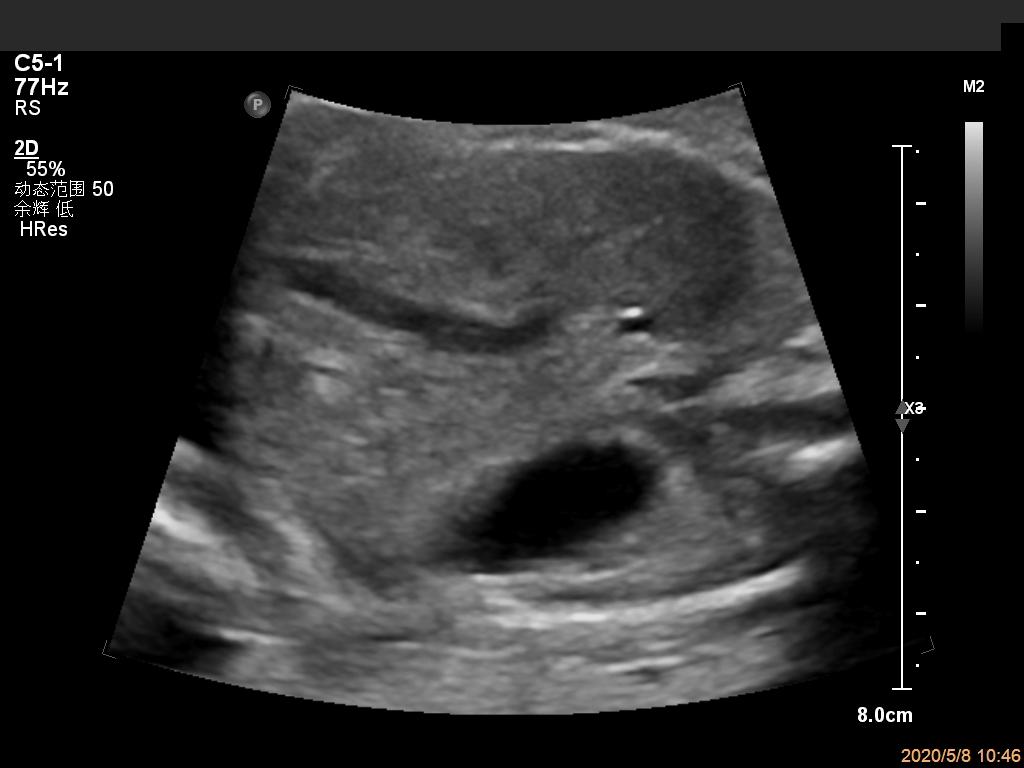

Supplement: S2 Dataset — (ZIP) [file pone.0305250.s002.zip › FE-SD-2/images/test_res/245_ab.jpg]

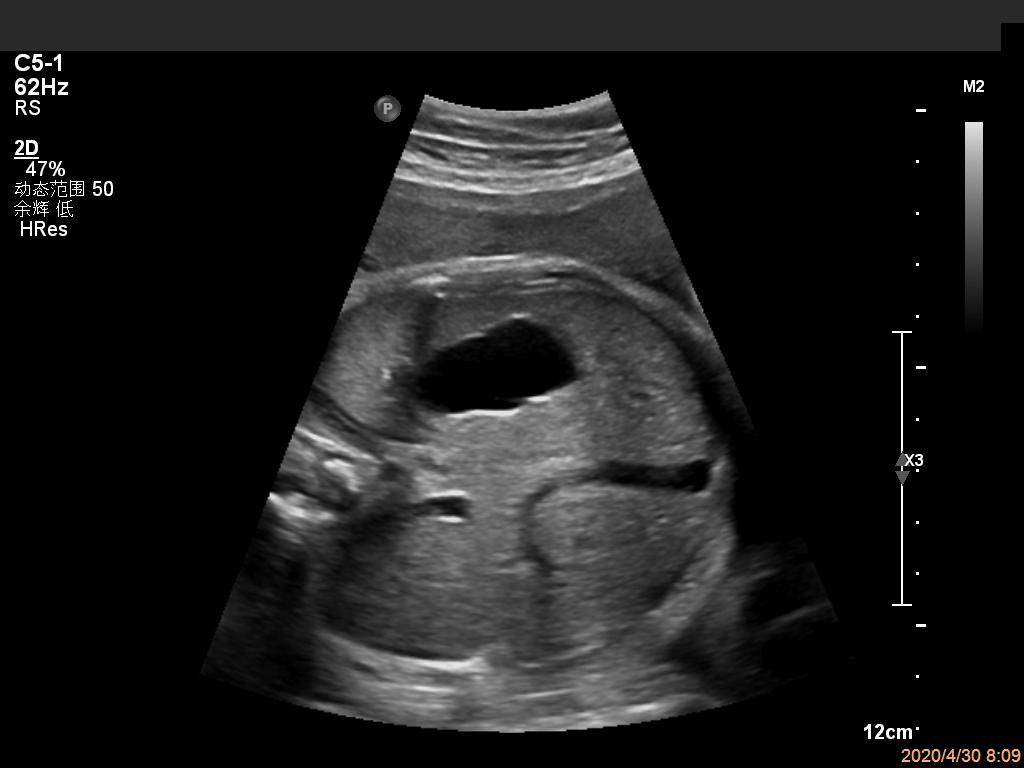

Supplement: S2 Dataset — (ZIP) [file pone.0305250.s002.zip › FE-SD-2/images/test_res/246_ab.jpg]

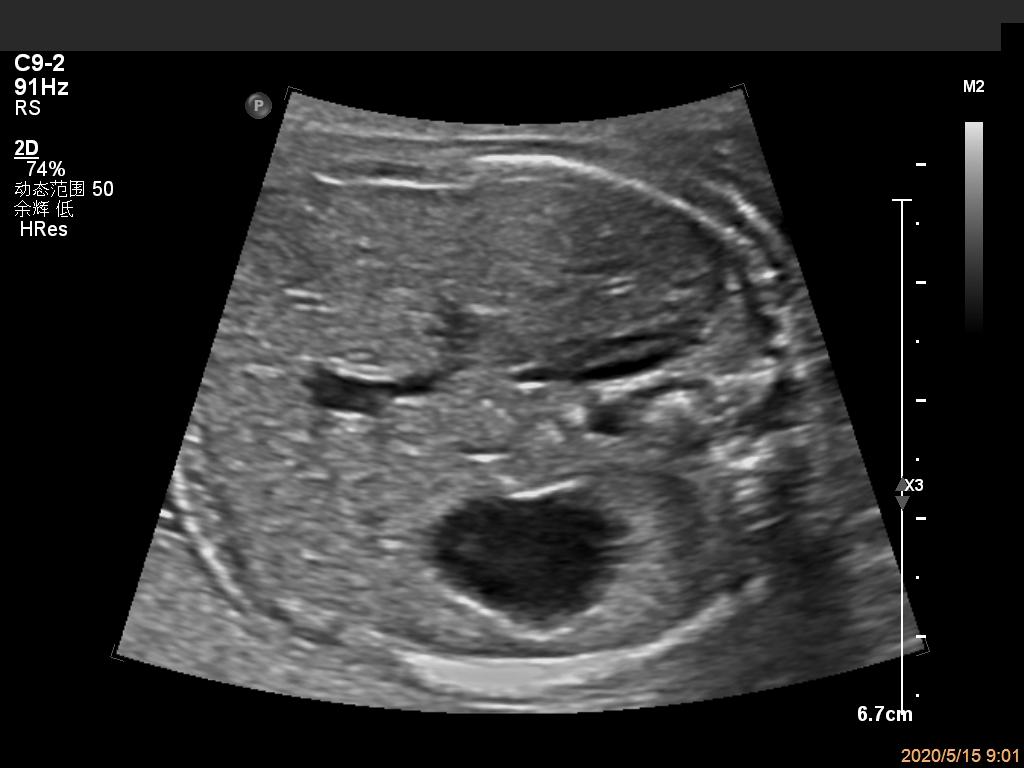

Supplement: S2 Dataset — (ZIP) [file pone.0305250.s002.zip › FE-SD-2/images/test_res/248_ab.jpg]

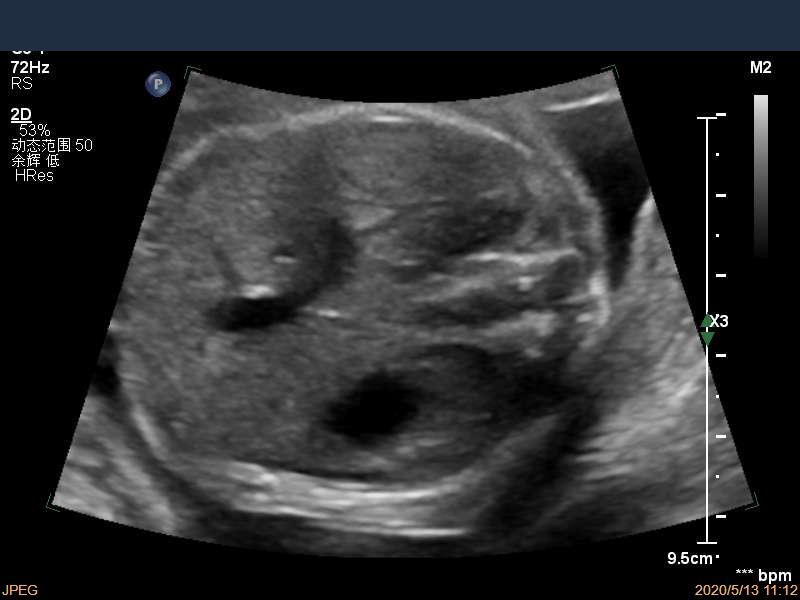

Supplement: S2 Dataset — (ZIP) [file pone.0305250.s002.zip › FE-SD-2/images/test_res/249_ab.jpg]

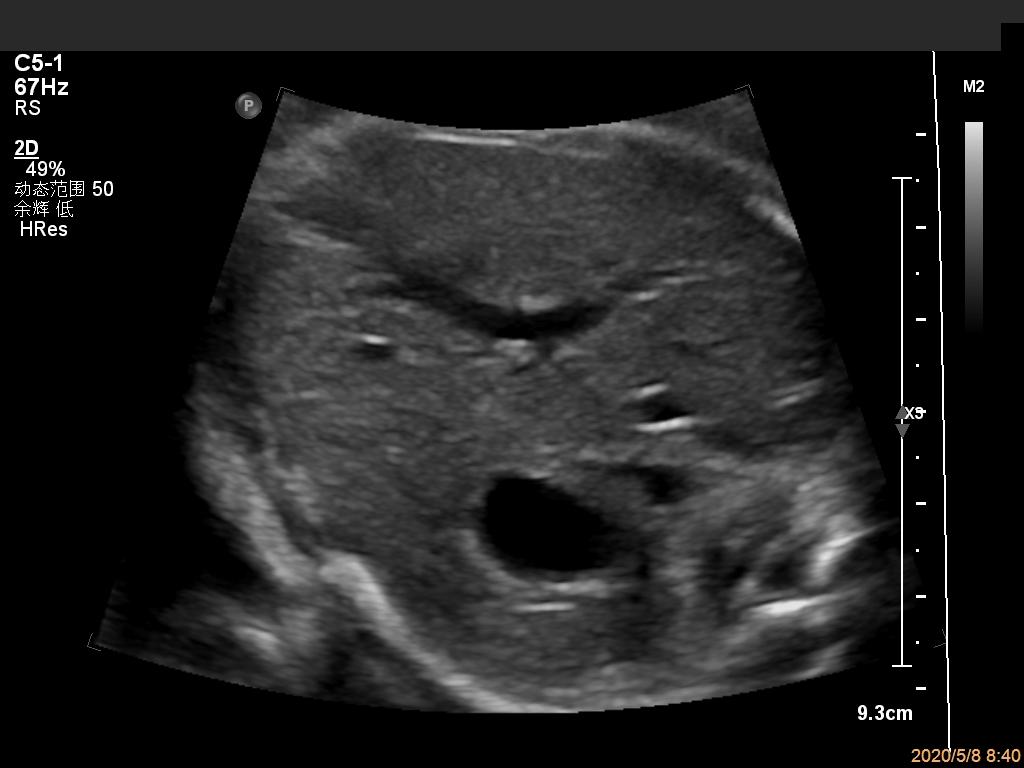

Supplement: S2 Dataset — (ZIP) [file pone.0305250.s002.zip › FE-SD-2/images/test_res/251_ab.jpg]

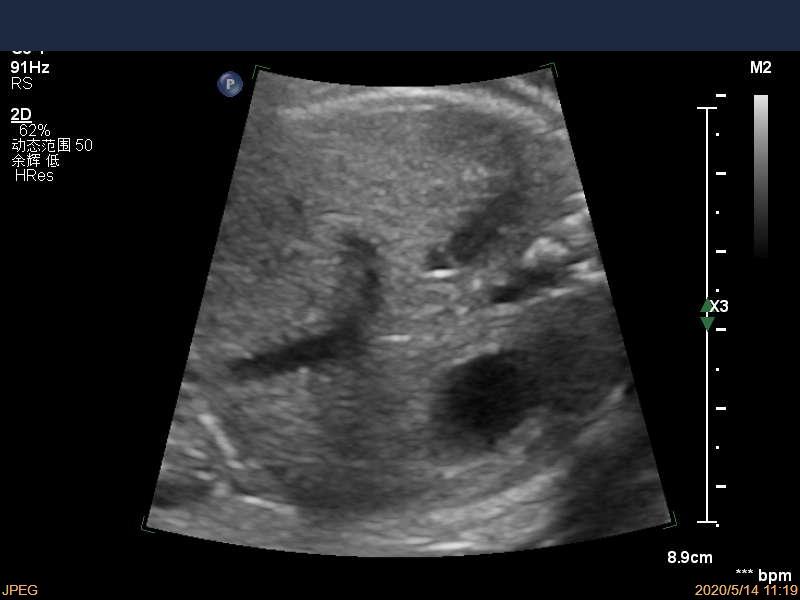

Supplement: S2 Dataset — (ZIP) [file pone.0305250.s002.zip › FE-SD-2/images/test_res/252_ab.jpg]

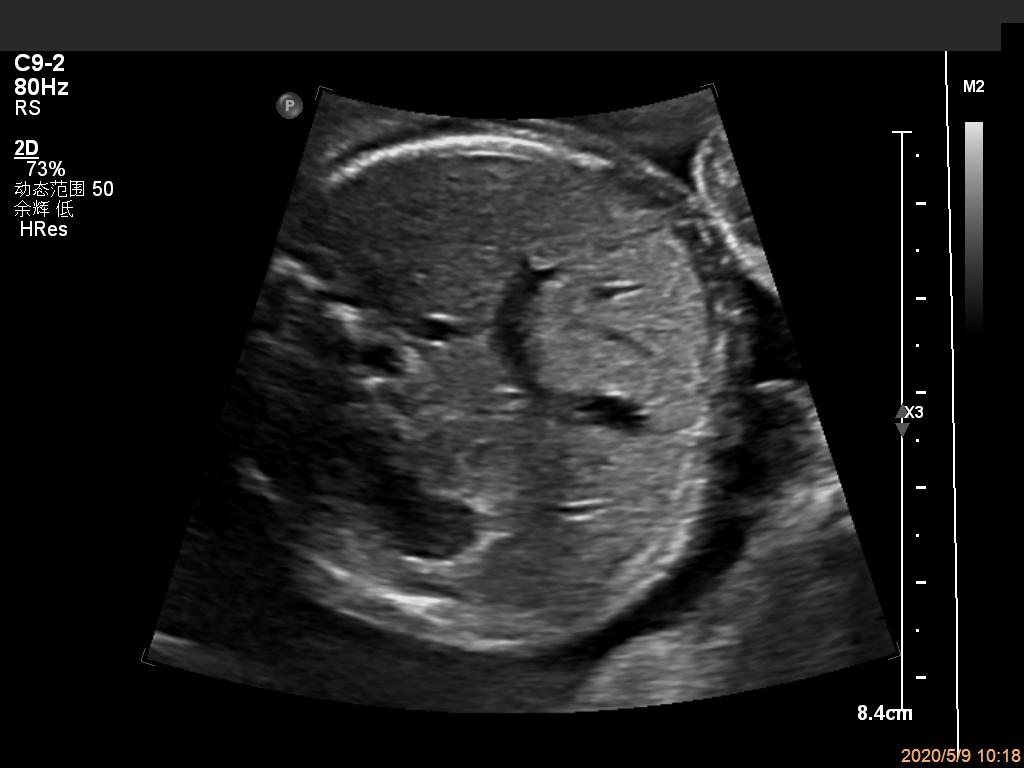

Supplement: S2 Dataset — (ZIP) [file pone.0305250.s002.zip › FE-SD-2/images/test_res/253_ab.jpg]

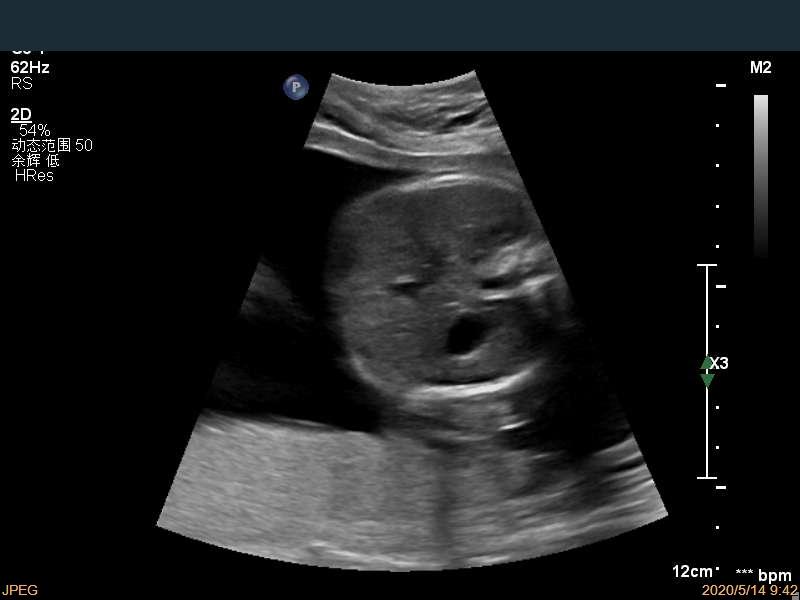

Supplement: S2 Dataset — (ZIP) [file pone.0305250.s002.zip › FE-SD-2/images/test_res/254_ab.jpg]

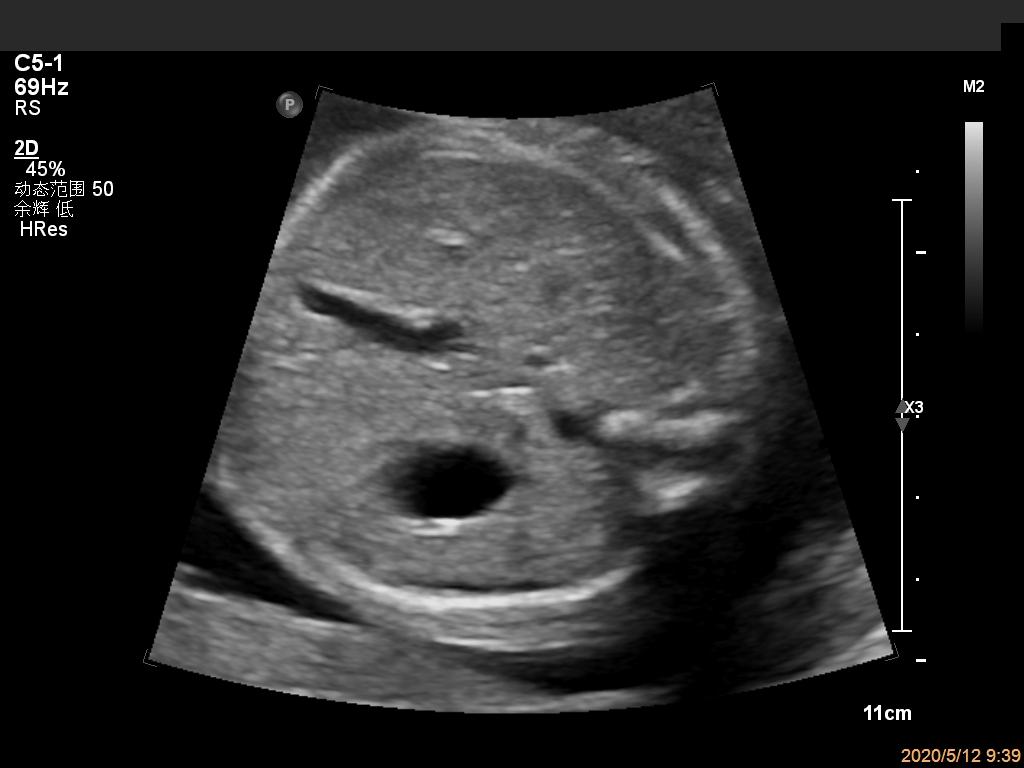

Supplement: S2 Dataset — (ZIP) [file pone.0305250.s002.zip › FE-SD-2/images/test_res/256_ab.jpg]

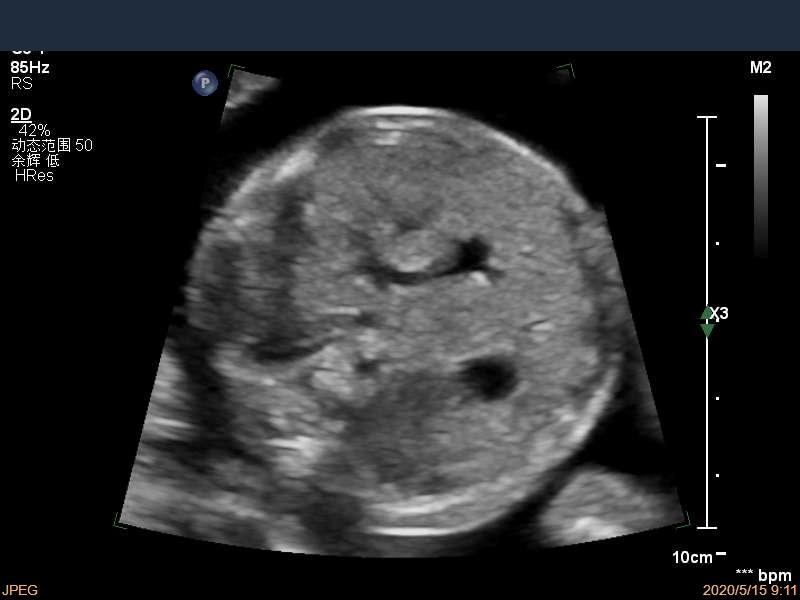

Supplement: S2 Dataset — (ZIP) [file pone.0305250.s002.zip › FE-SD-2/images/test_res/258_ab.jpg]

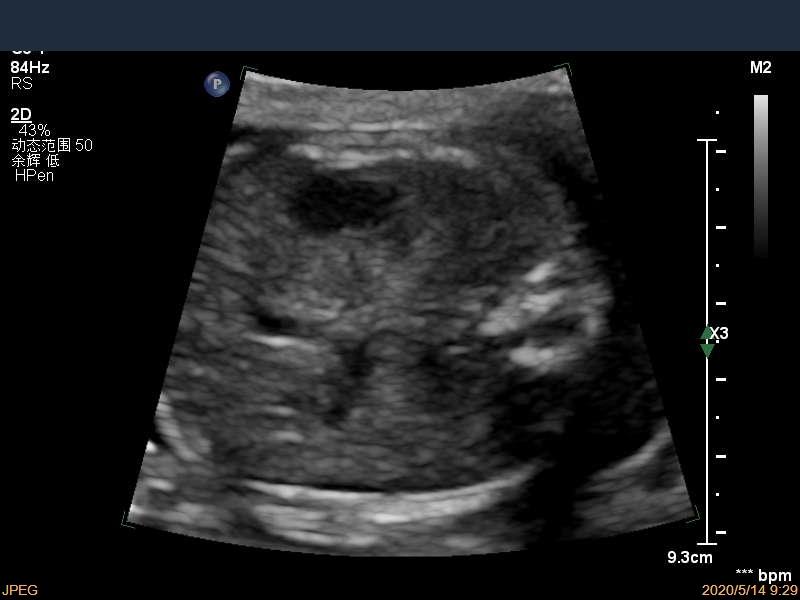

Supplement: S2 Dataset — (ZIP) [file pone.0305250.s002.zip › FE-SD-2/images/test_res/260_ab.jpg]

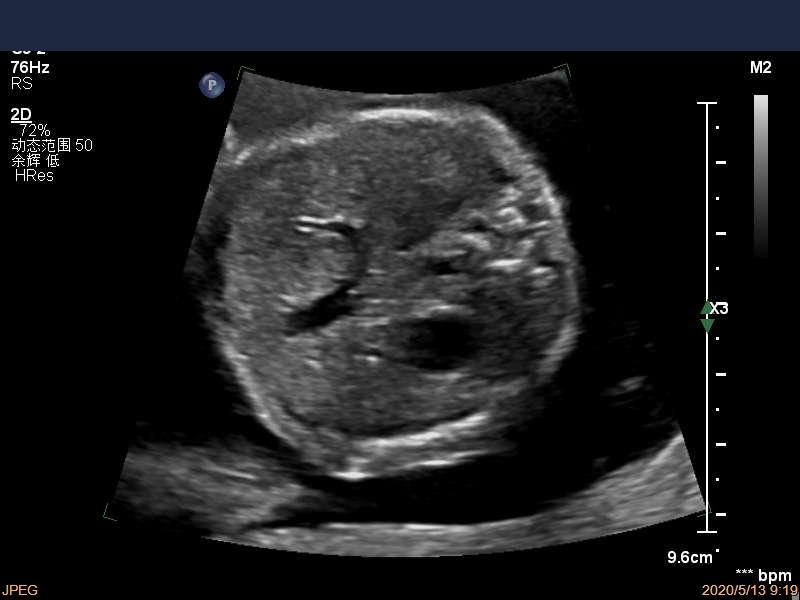

Supplement: S2 Dataset — (ZIP) [file pone.0305250.s002.zip › FE-SD-2/images/test_res/262_ab.jpg]

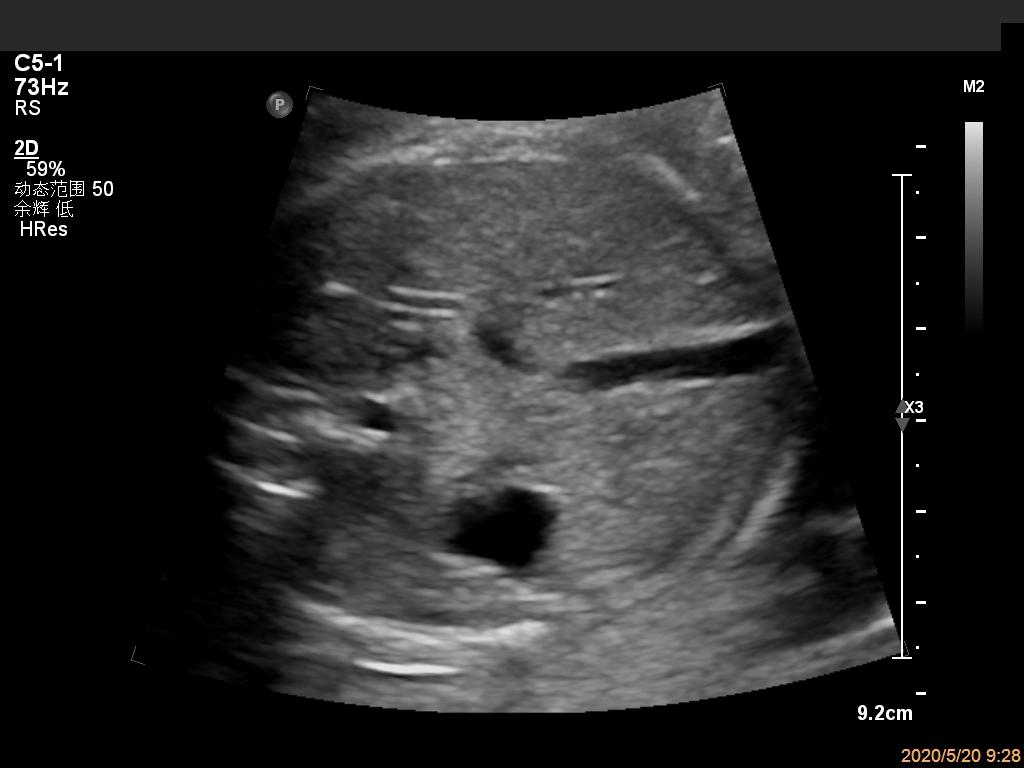

Supplement: S2 Dataset — (ZIP) [file pone.0305250.s002.zip › FE-SD-2/images/test_res/269_ab.jpg]

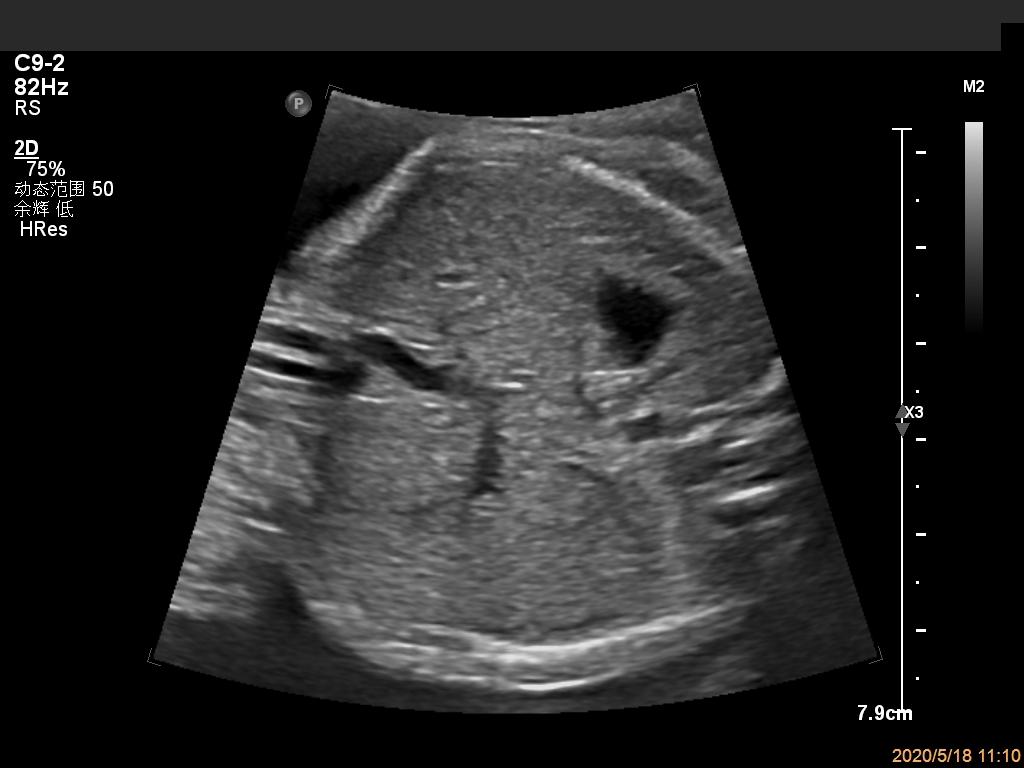

Supplement: S2 Dataset — (ZIP) [file pone.0305250.s002.zip › FE-SD-2/images/test_res/270_ab.jpg]

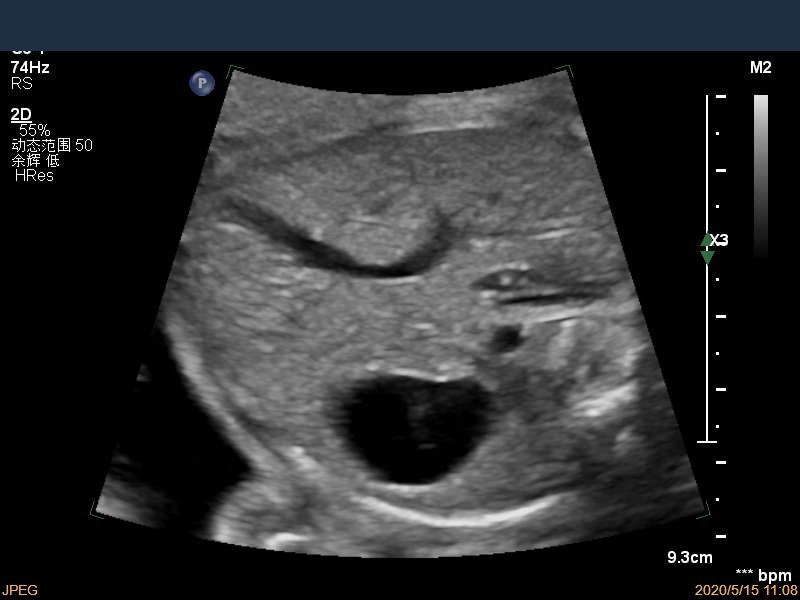

Supplement: S2 Dataset — (ZIP) [file pone.0305250.s002.zip › FE-SD-2/images/test_res/275_ab.jpg]

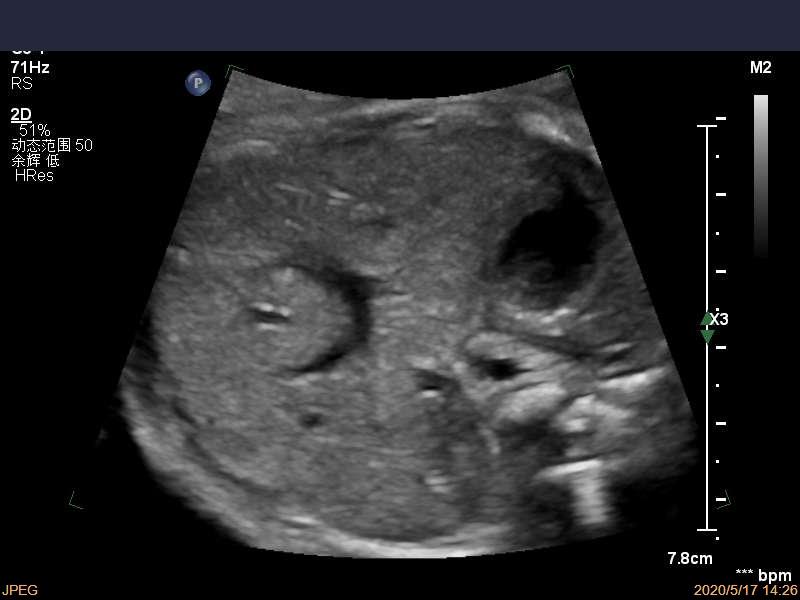

Supplement: S2 Dataset — (ZIP) [file pone.0305250.s002.zip › FE-SD-2/images/test_res/276_ab.jpg]

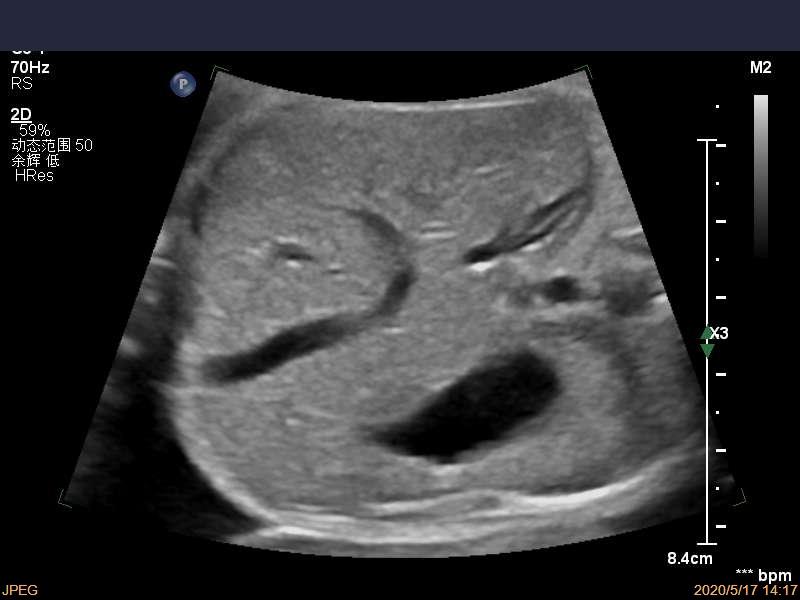

Supplement: S2 Dataset — (ZIP) [file pone.0305250.s002.zip › FE-SD-2/images/test_res/277_ab.jpg]

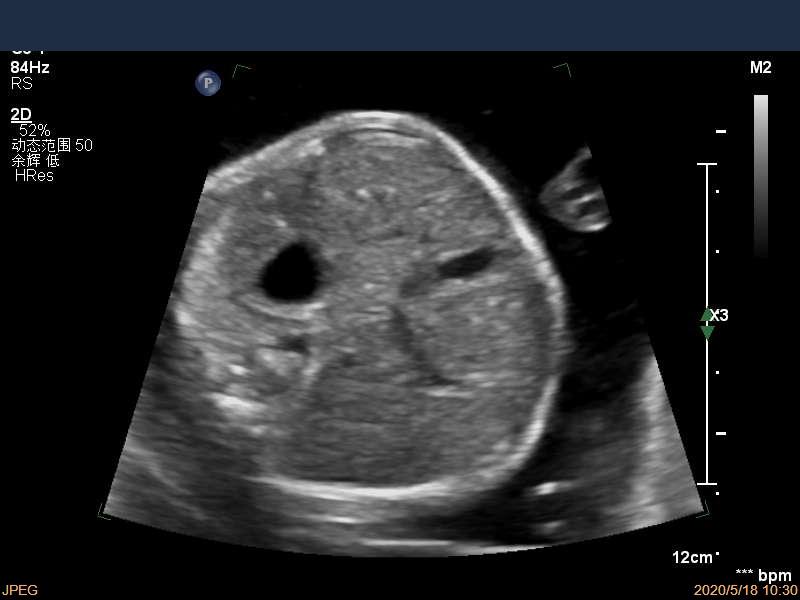

Supplement: S2 Dataset — (ZIP) [file pone.0305250.s002.zip › FE-SD-2/images/test_res/278_ab.jpg]

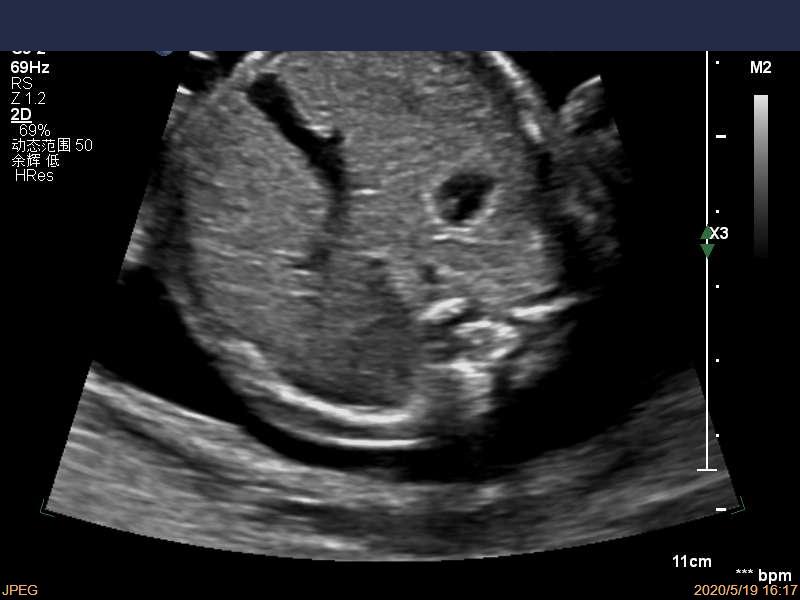

Supplement: S2 Dataset — (ZIP) [file pone.0305250.s002.zip › FE-SD-2/images/test_res/279_ab.jpg]

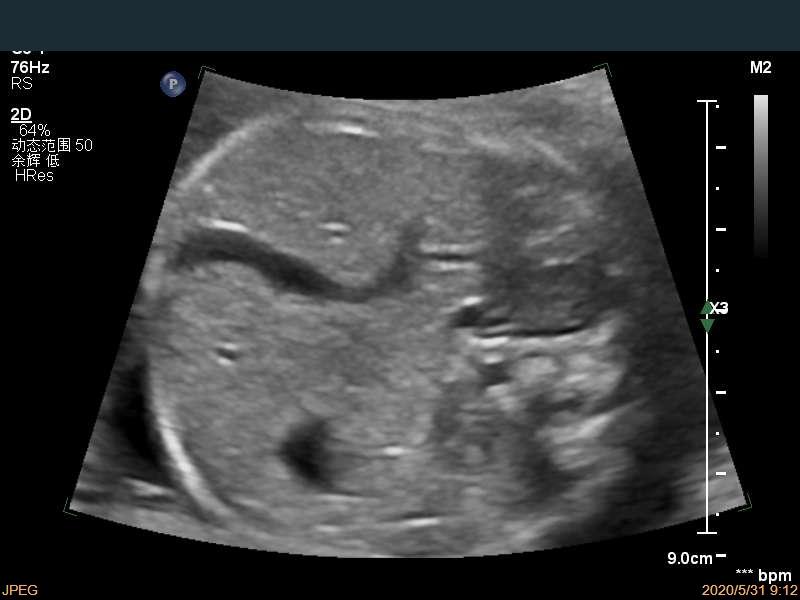

Supplement: S2 Dataset — (ZIP) [file pone.0305250.s002.zip › FE-SD-2/images/test_res/280_ab.jpg]

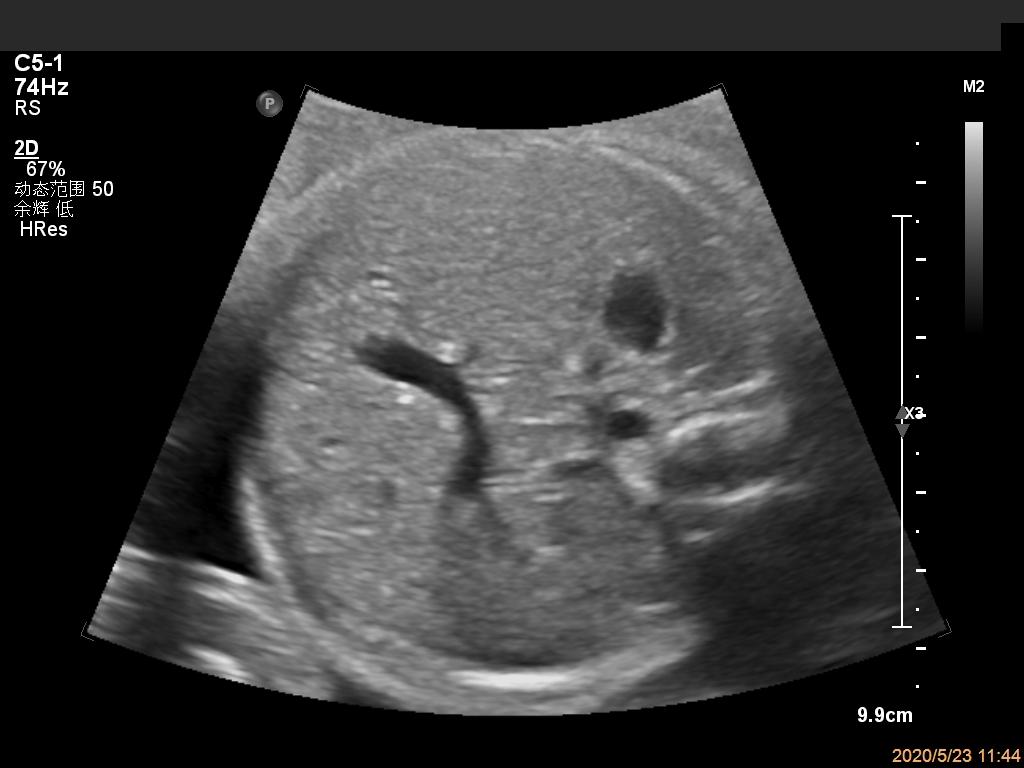

Supplement: S2 Dataset — (ZIP) [file pone.0305250.s002.zip › FE-SD-2/images/test_res/281_ab.jpg]

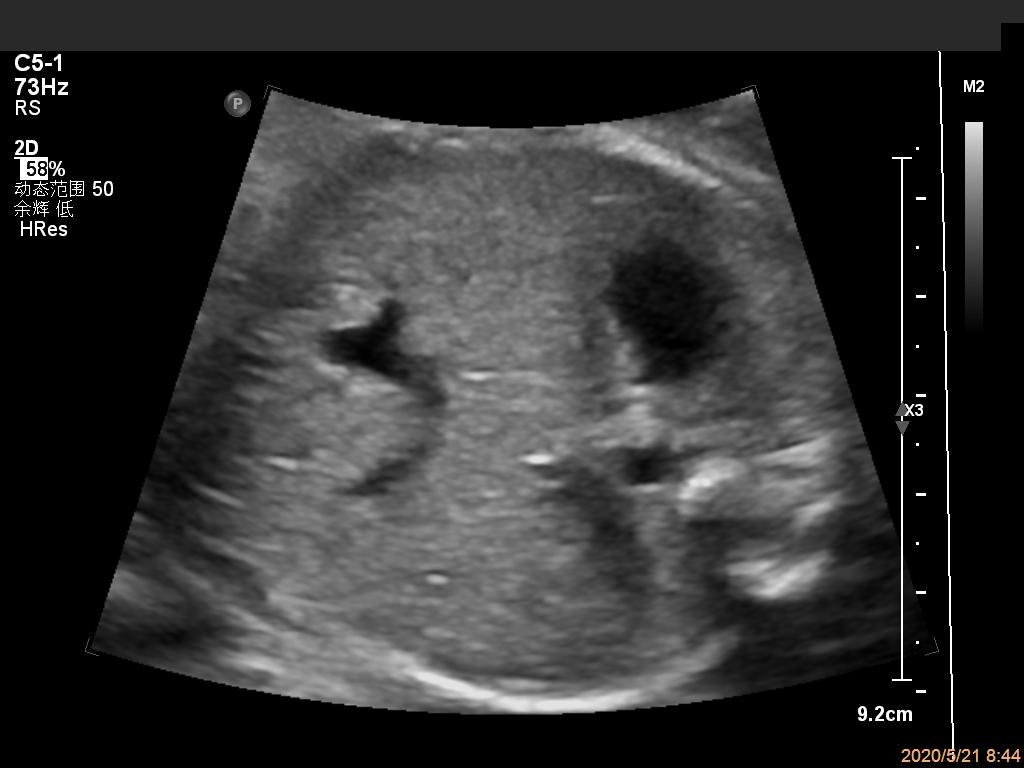

Supplement: S2 Dataset — (ZIP) [file pone.0305250.s002.zip › FE-SD-2/images/test_res/283_ab.jpg]

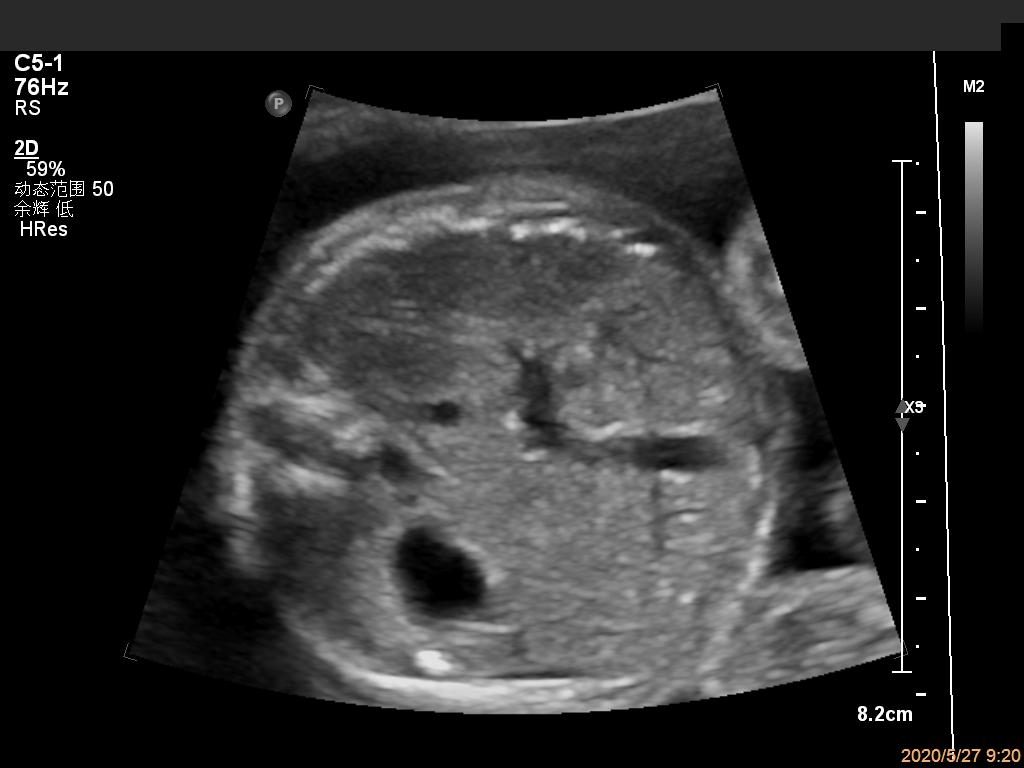

Supplement: S2 Dataset — (ZIP) [file pone.0305250.s002.zip › FE-SD-2/images/test_res/286_ab.jpg]

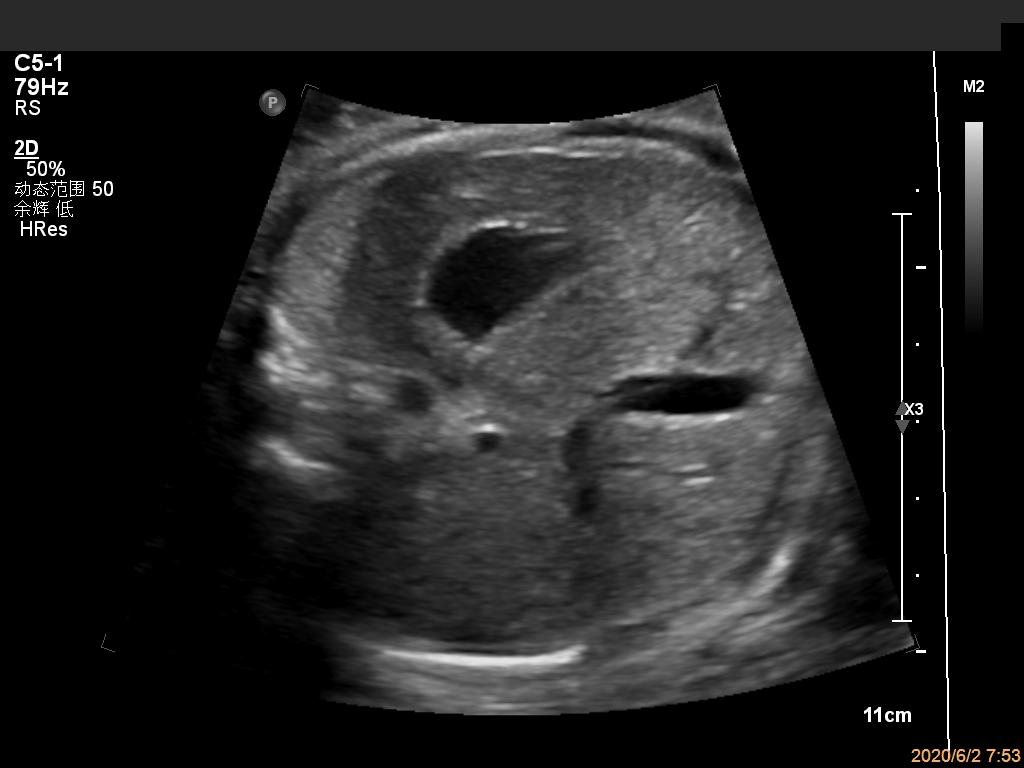

Supplement: S2 Dataset — (ZIP) [file pone.0305250.s002.zip › FE-SD-2/images/test_res/287_ab.jpg]

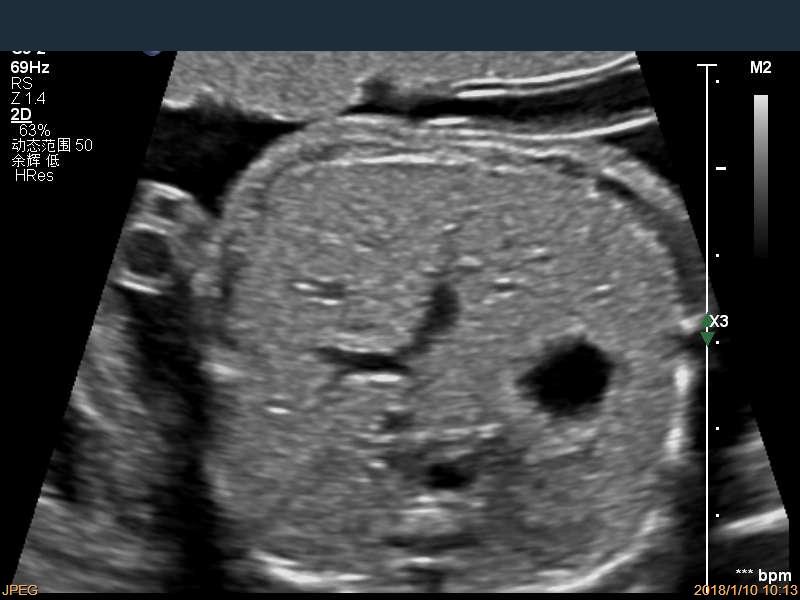

Supplement: S2 Dataset — (ZIP) [file pone.0305250.s002.zip › FE-SD-2/images/test_res/294_ab.jpg]

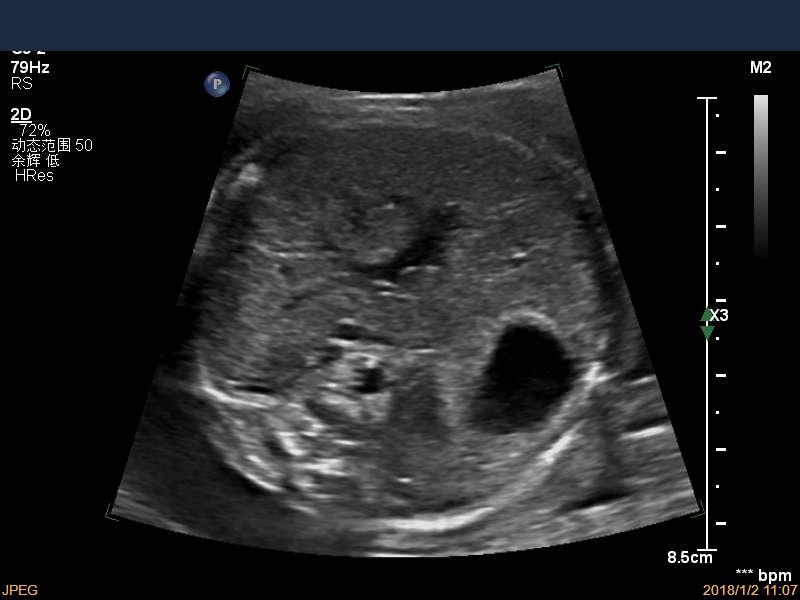

Supplement: S2 Dataset — (ZIP) [file pone.0305250.s002.zip › FE-SD-2/images/test_res/299_ab.jpg]

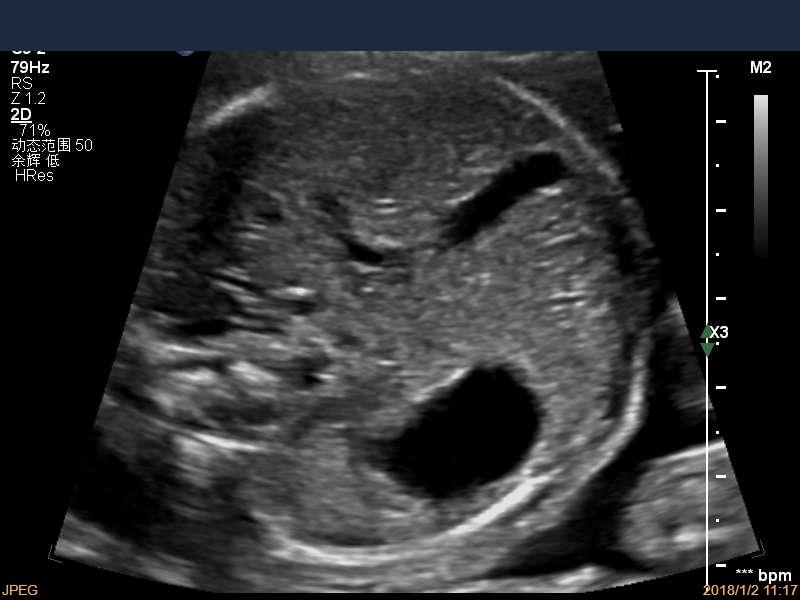

Supplement: S2 Dataset — (ZIP) [file pone.0305250.s002.zip › FE-SD-2/images/test_res/300_ab.jpg]

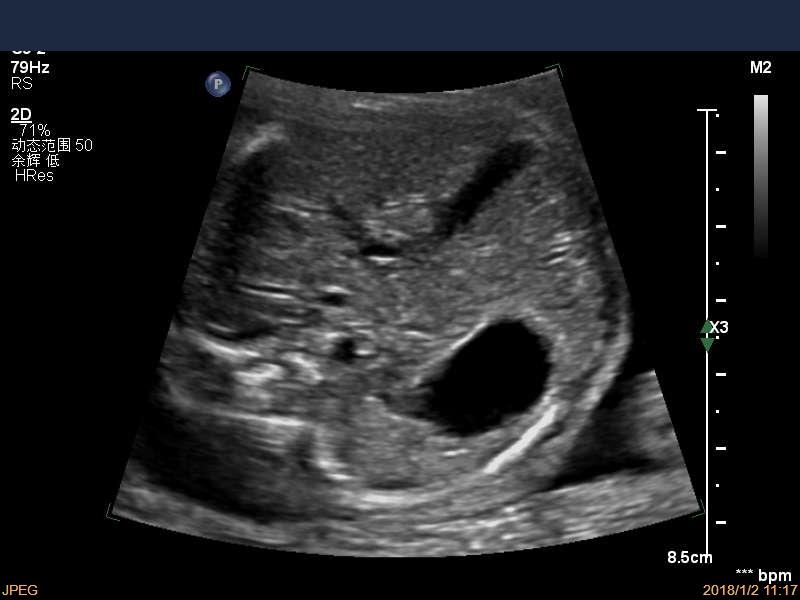

Supplement: S2 Dataset — (ZIP) [file pone.0305250.s002.zip › FE-SD-2/images/test_res/301_ab.jpg]

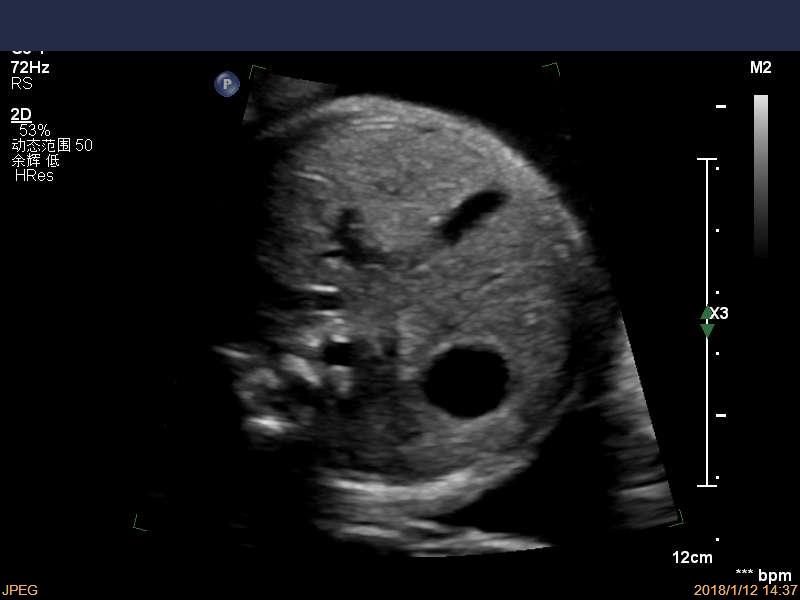

Supplement: S2 Dataset — (ZIP) [file pone.0305250.s002.zip › FE-SD-2/images/test_res/302_ab.jpg]

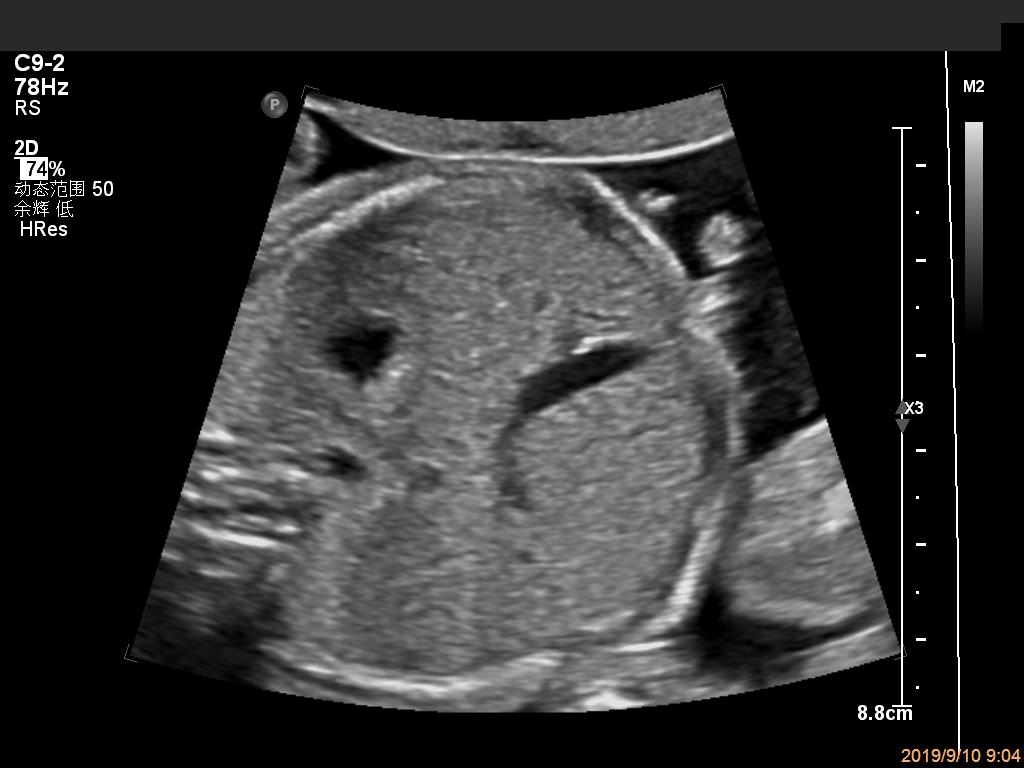

Supplement: S2 Dataset — (ZIP) [file pone.0305250.s002.zip › FE-SD-2/images/test_res/304_ab.jpg]

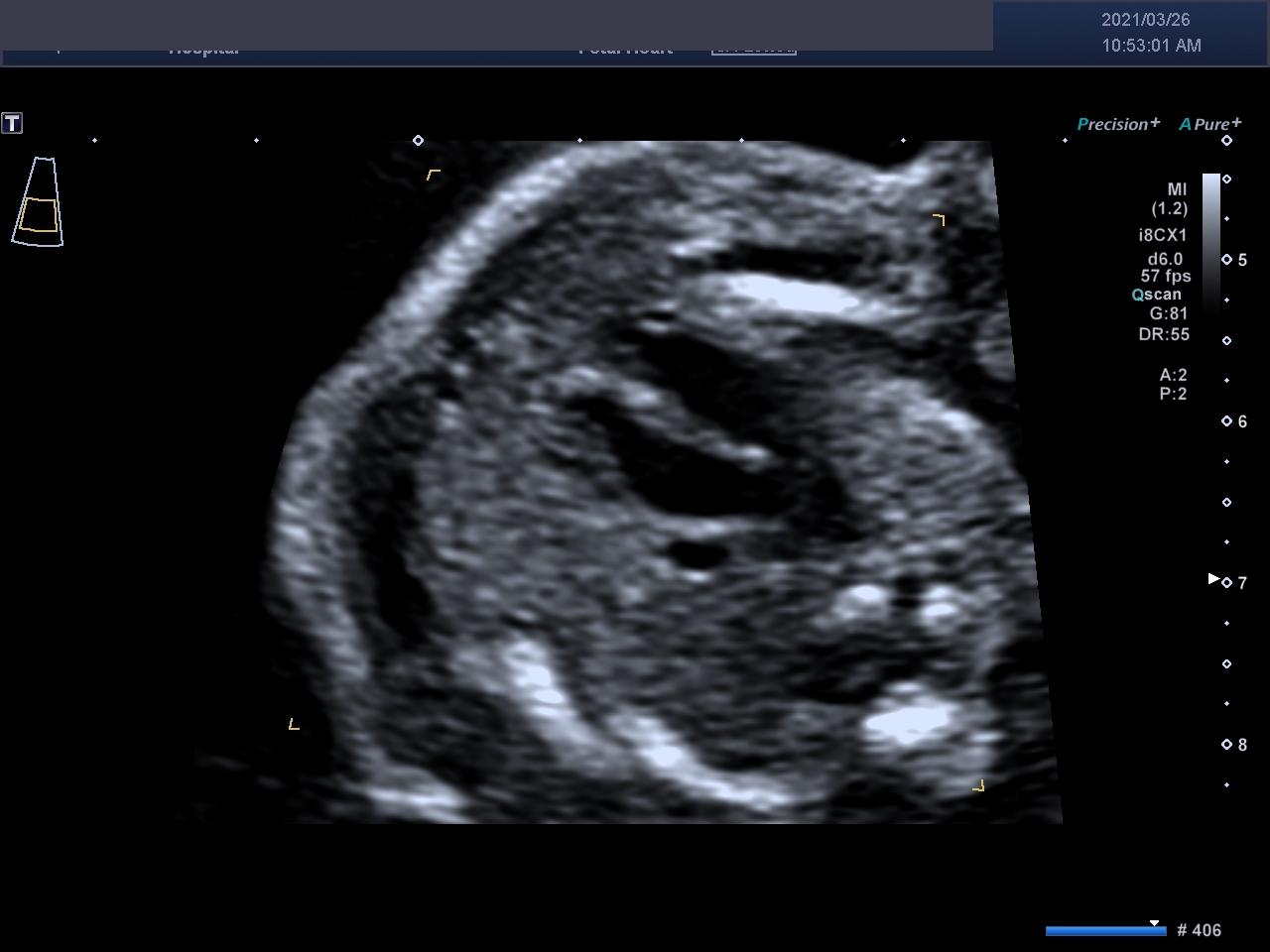

Supplement: S2 Dataset — (ZIP) [file pone.0305250.s002.zip › FE-SD-2/images/test_res/372_tv.jpg]

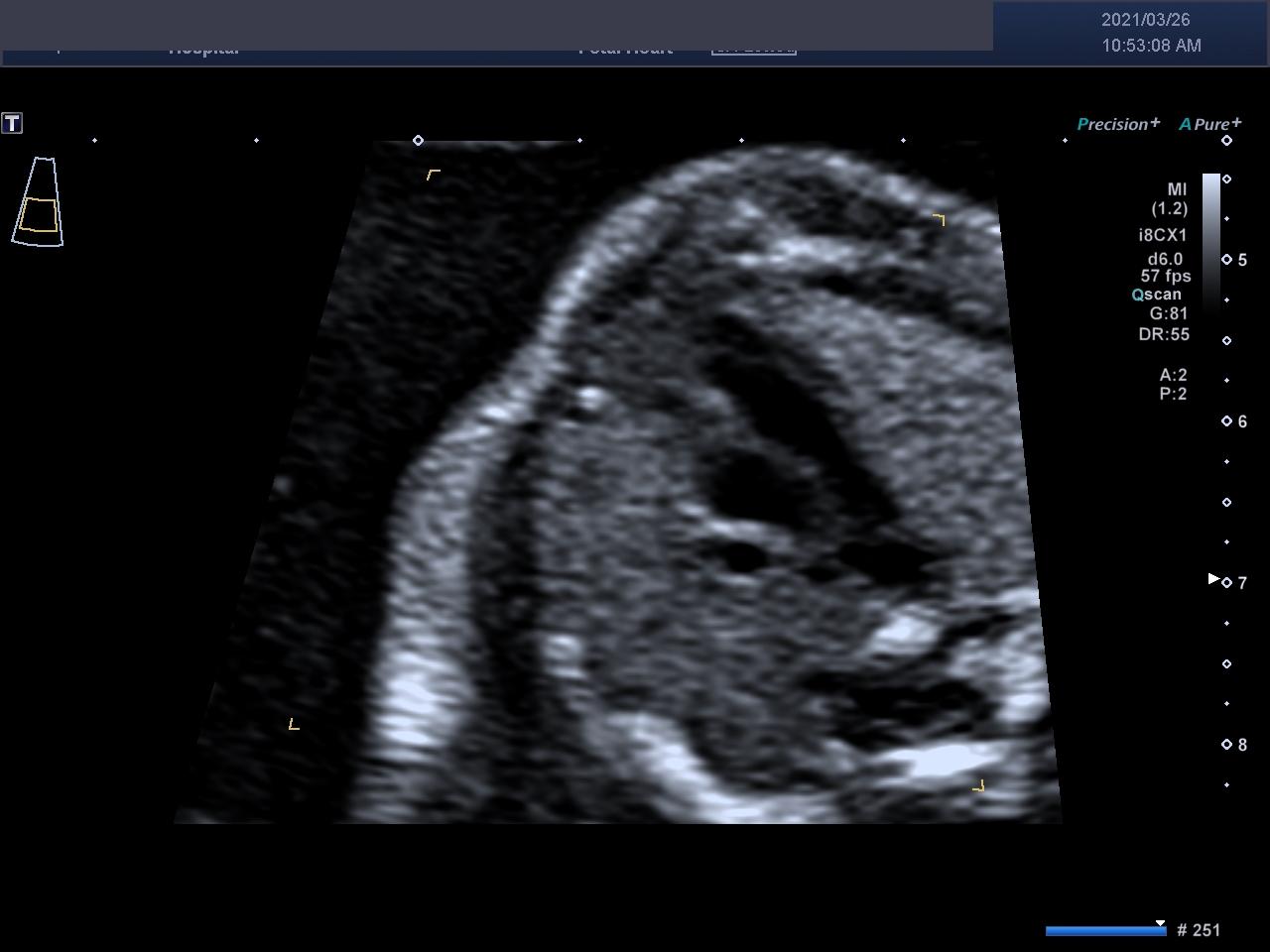

Supplement: S2 Dataset — (ZIP) [file pone.0305250.s002.zip › FE-SD-2/images/test_res/373_tv.jpg]

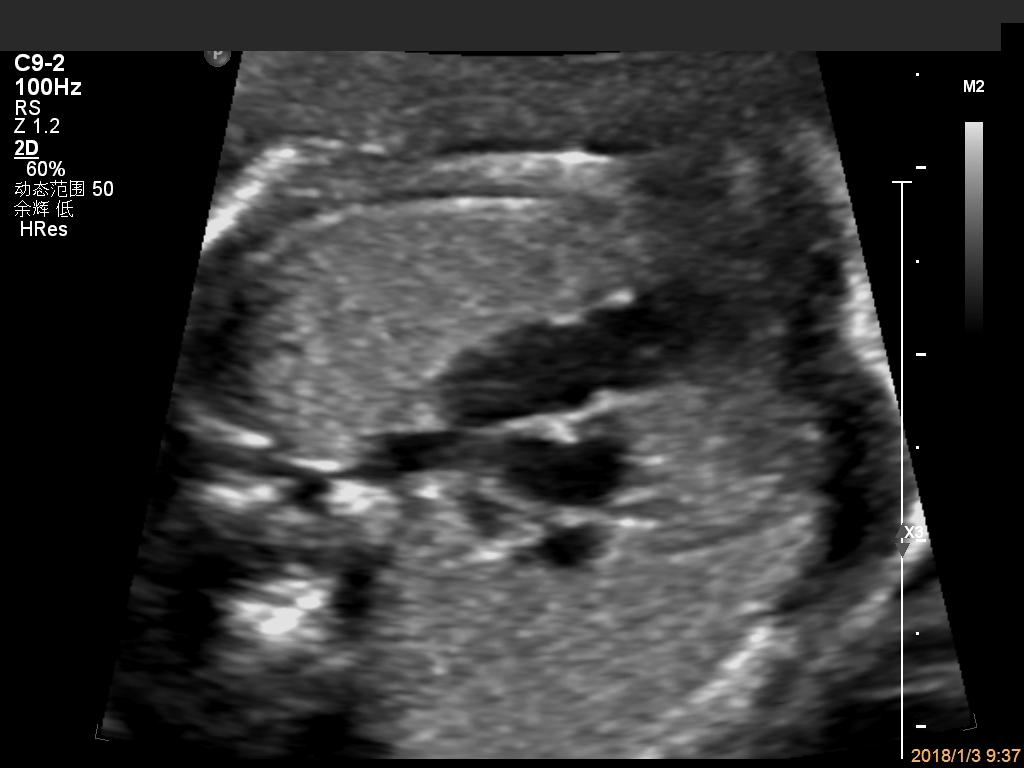

Supplement: S2 Dataset — (ZIP) [file pone.0305250.s002.zip › FE-SD-2/images/test_res/399_tv.jpg]

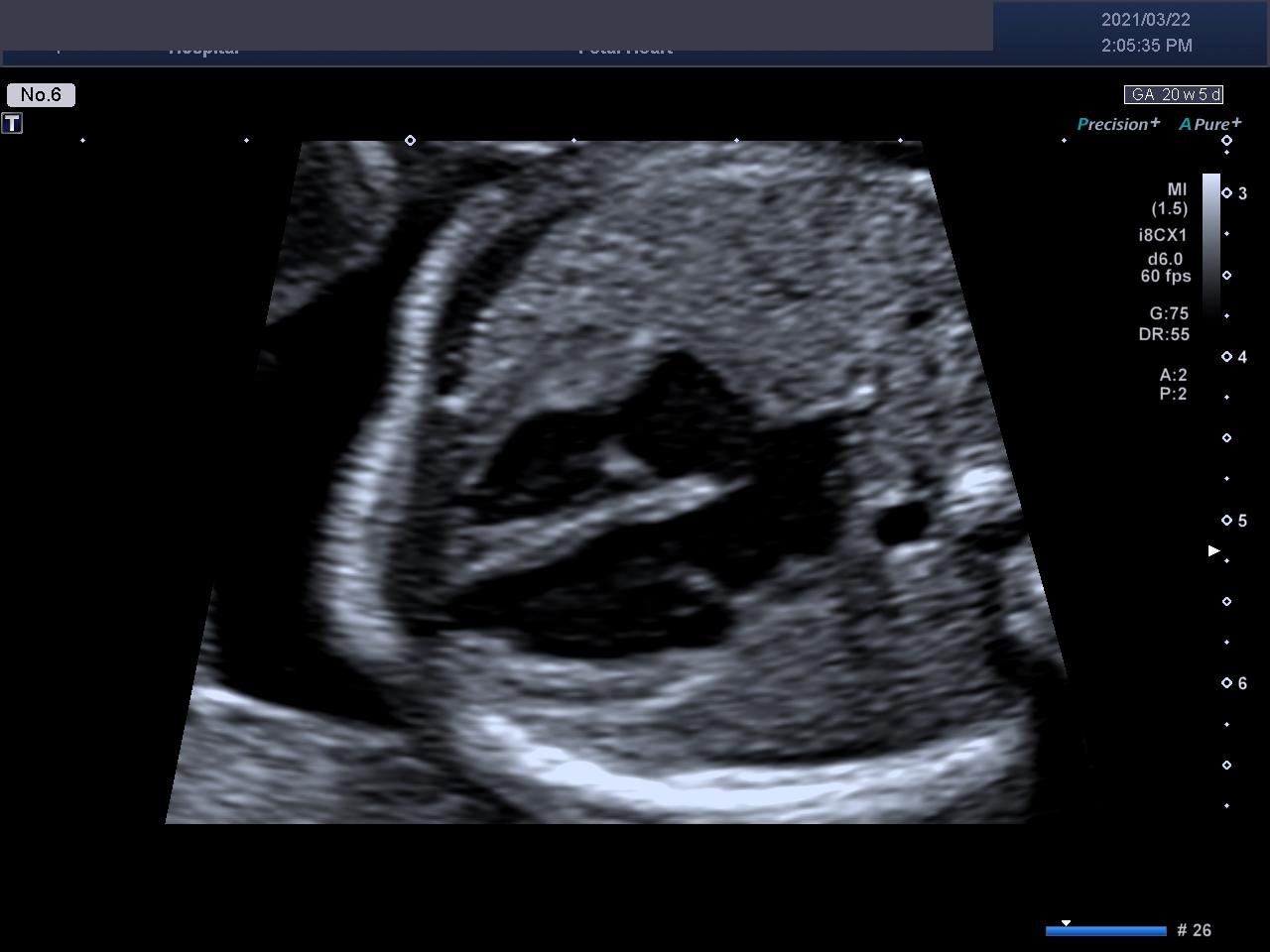

Supplement: S2 Dataset — (ZIP) [file pone.0305250.s002.zip › FE-SD-2/images/test_res/419_fc.jpg]

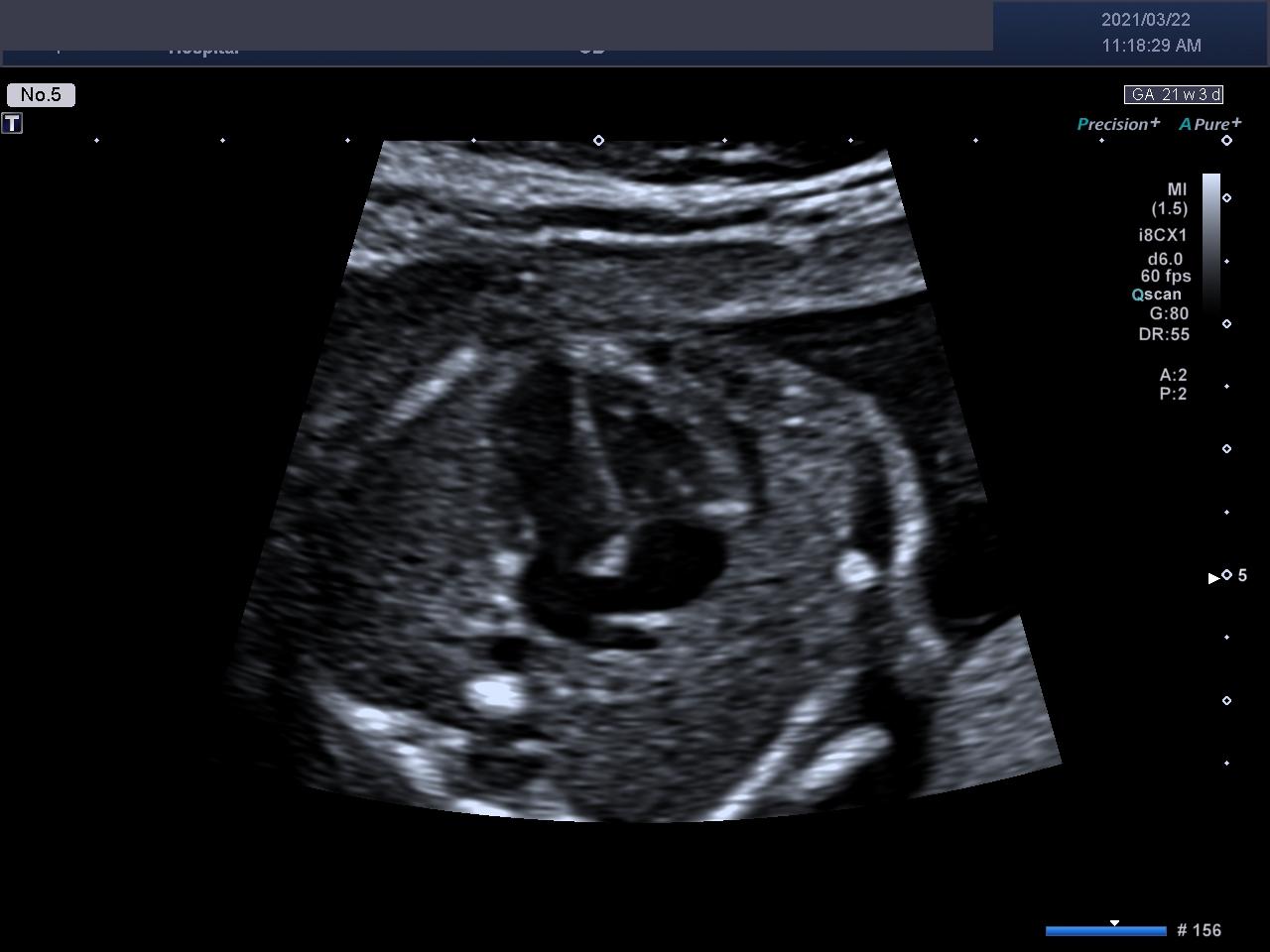

Supplement: S2 Dataset — (ZIP) [file pone.0305250.s002.zip › FE-SD-2/images/test_res/422_fc.jpg]

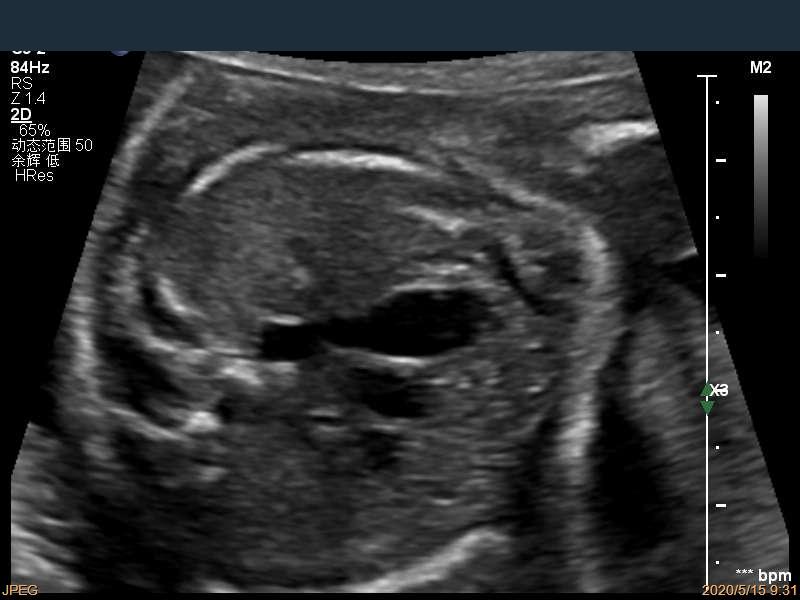

Supplement: S2 Dataset — (ZIP) [file pone.0305250.s002.zip › FE-SD-2/images/test_res/423_tv.jpg]

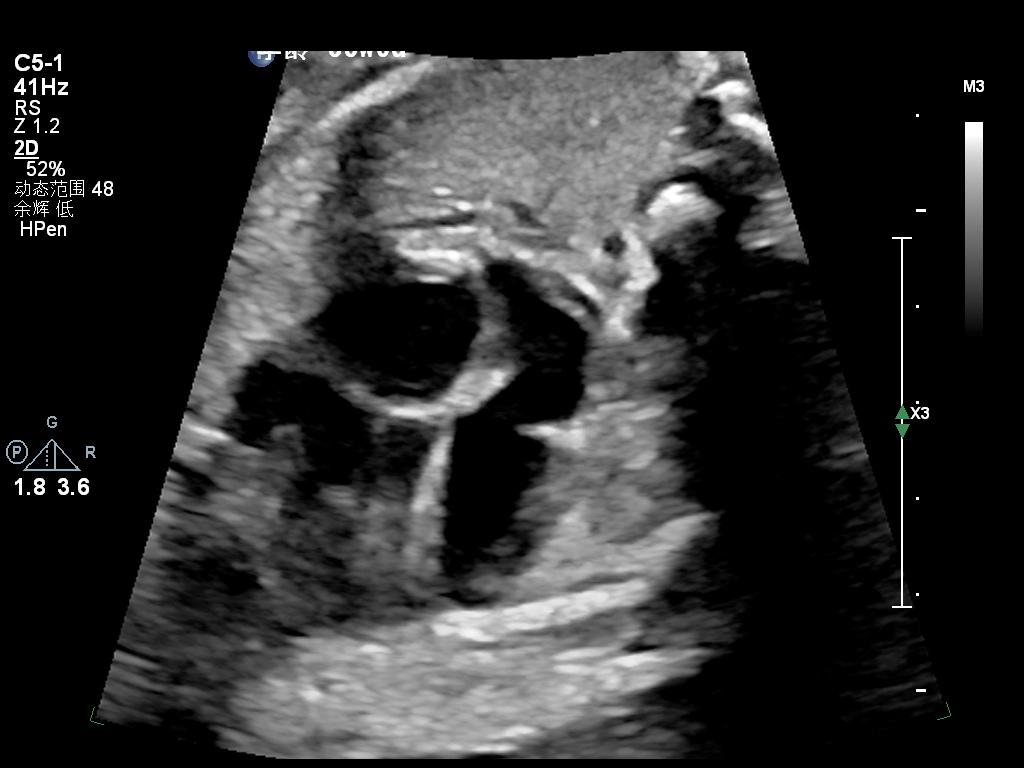

Supplement: S2 Dataset — (ZIP) [file pone.0305250.s002.zip › FE-SD-2/images/test_res/424_fc.jpg]

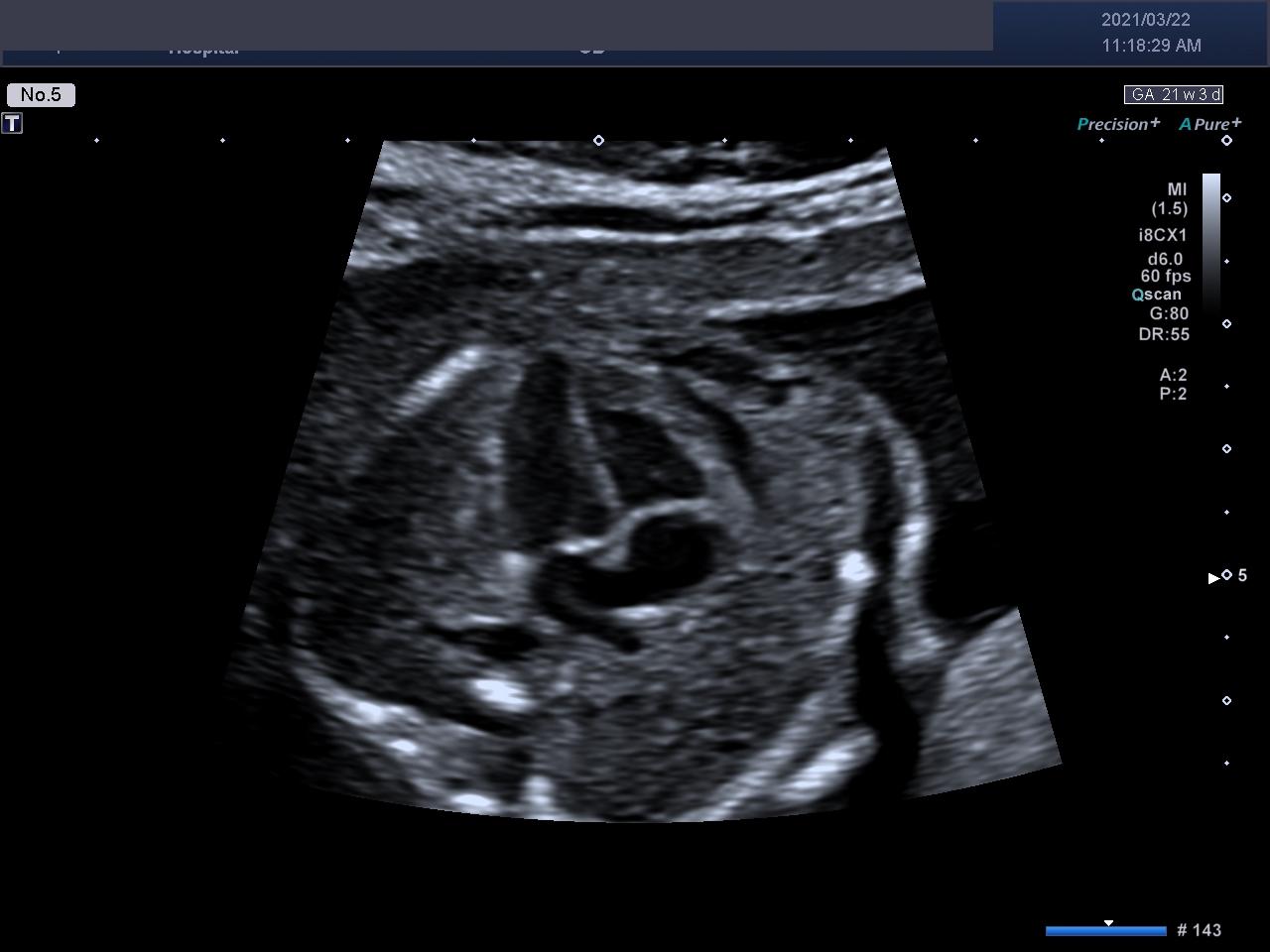

Supplement: S2 Dataset — (ZIP) [file pone.0305250.s002.zip › FE-SD-2/images/test_res/426_fc.jpg]

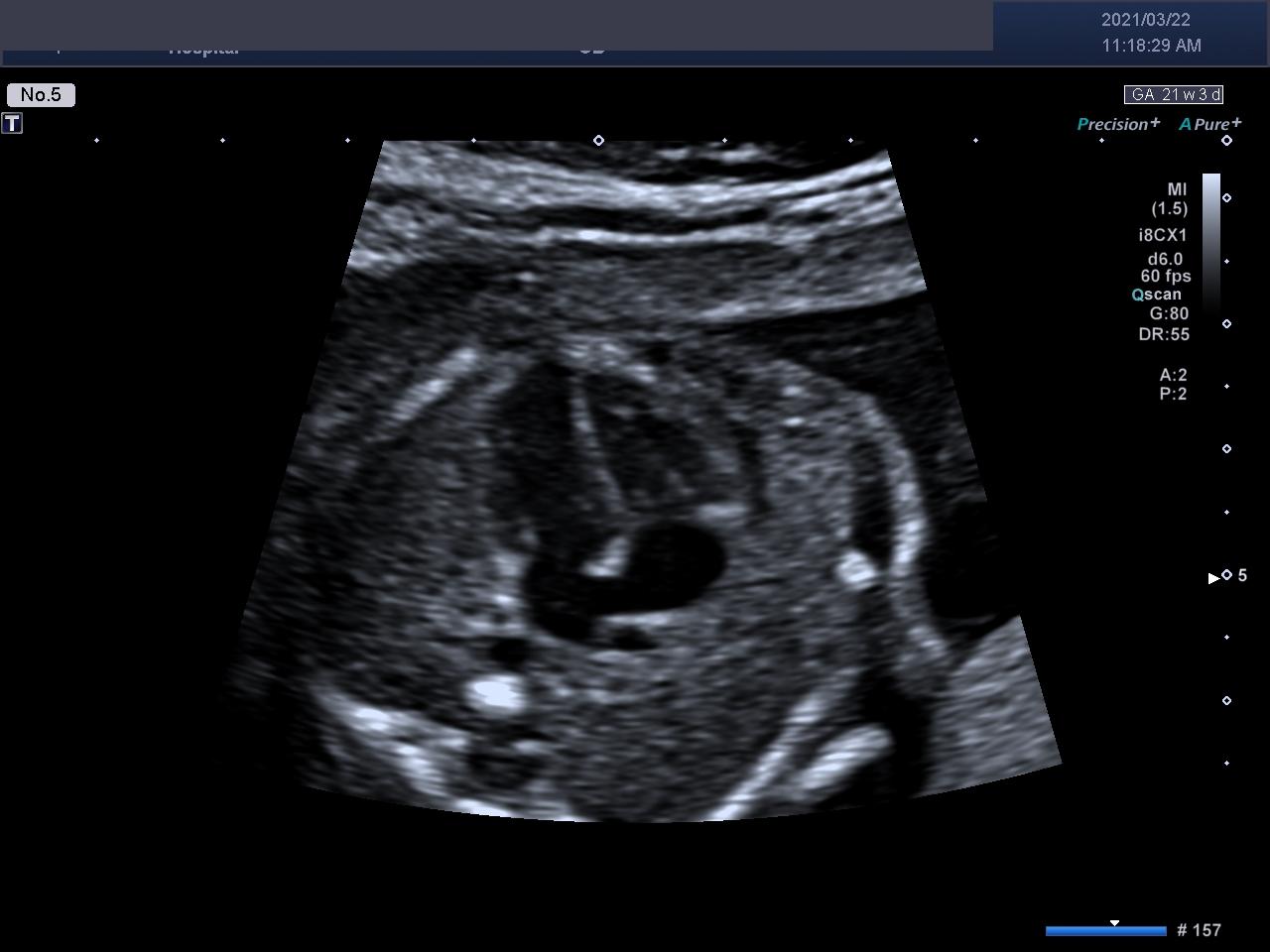

Supplement: S2 Dataset — (ZIP) [file pone.0305250.s002.zip › FE-SD-2/images/test_res/427_fc.jpg]

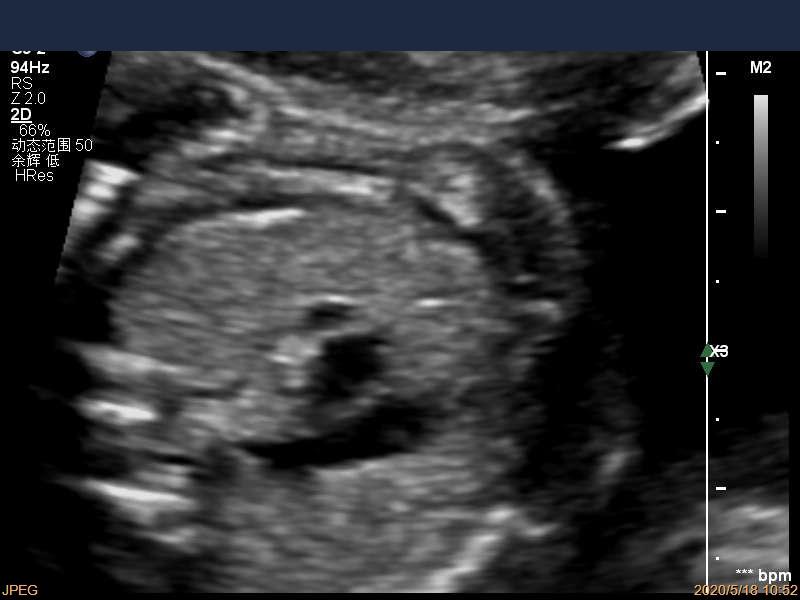

Supplement: S2 Dataset — (ZIP) [file pone.0305250.s002.zip › FE-SD-2/images/test_res/428_tv.jpg]

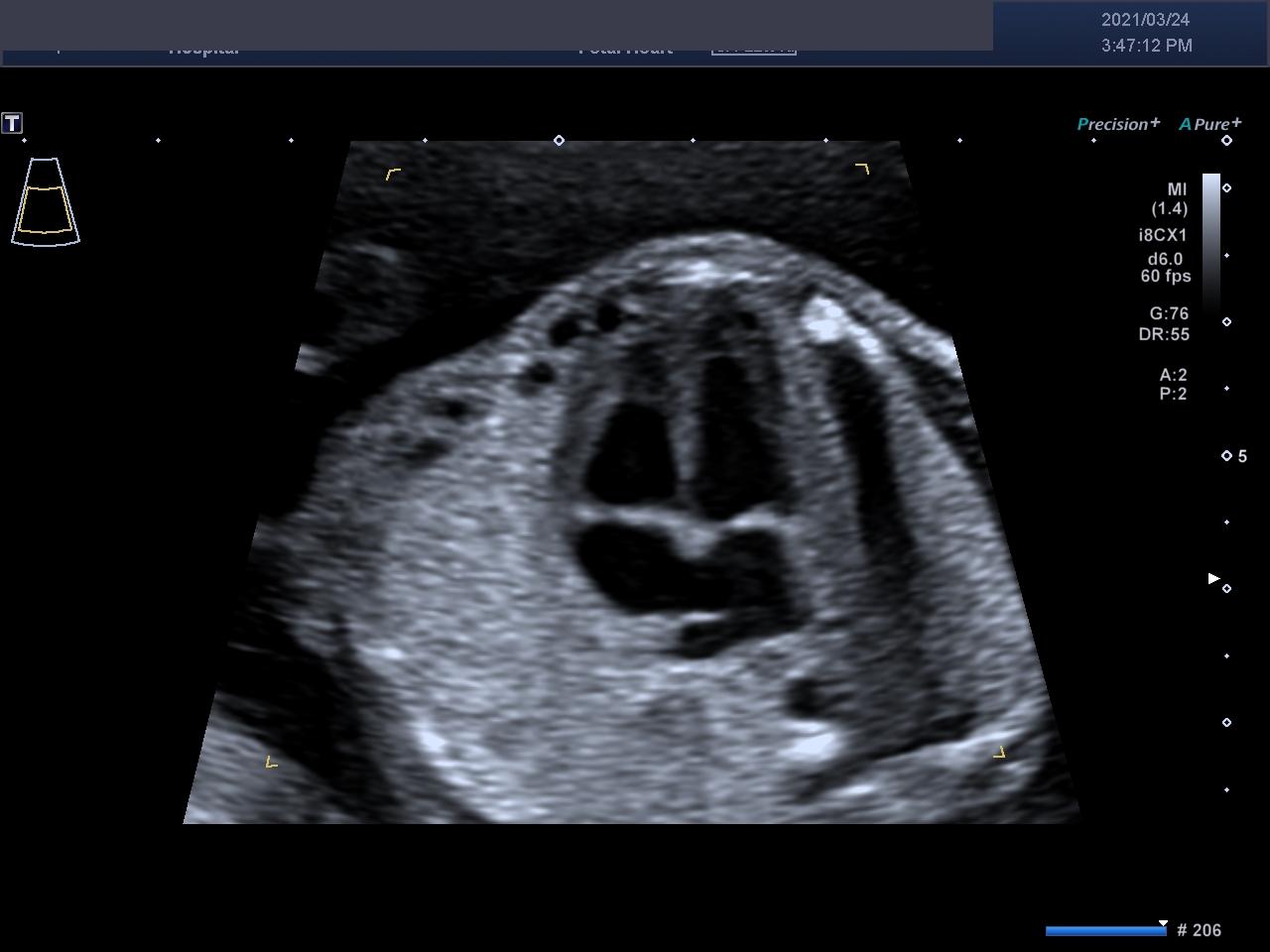

Supplement: S2 Dataset — (ZIP) [file pone.0305250.s002.zip › FE-SD-2/images/test_res/430_fc.jpg]

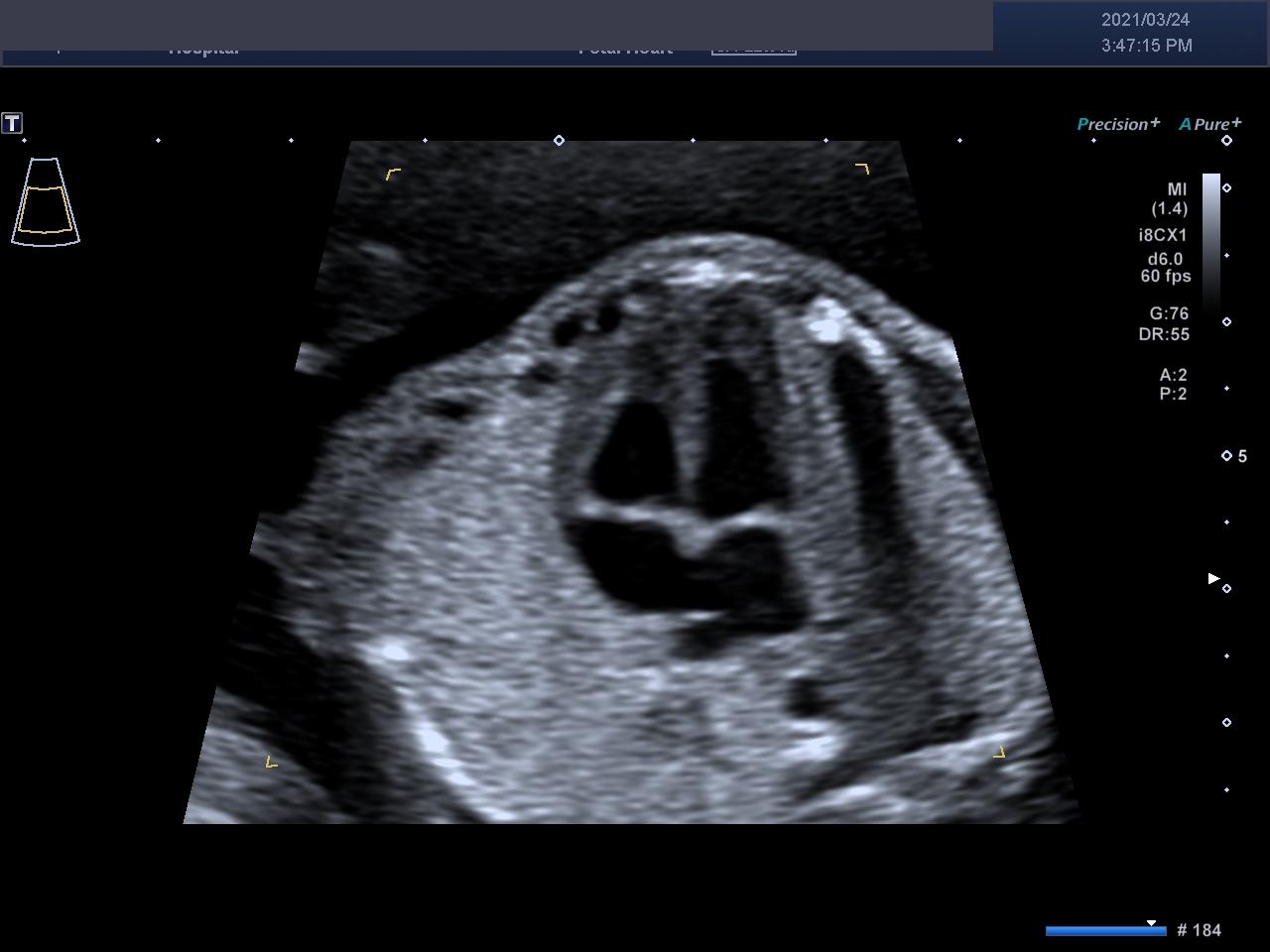

Supplement: S2 Dataset — (ZIP) [file pone.0305250.s002.zip › FE-SD-2/images/test_res/432_fc.jpg]

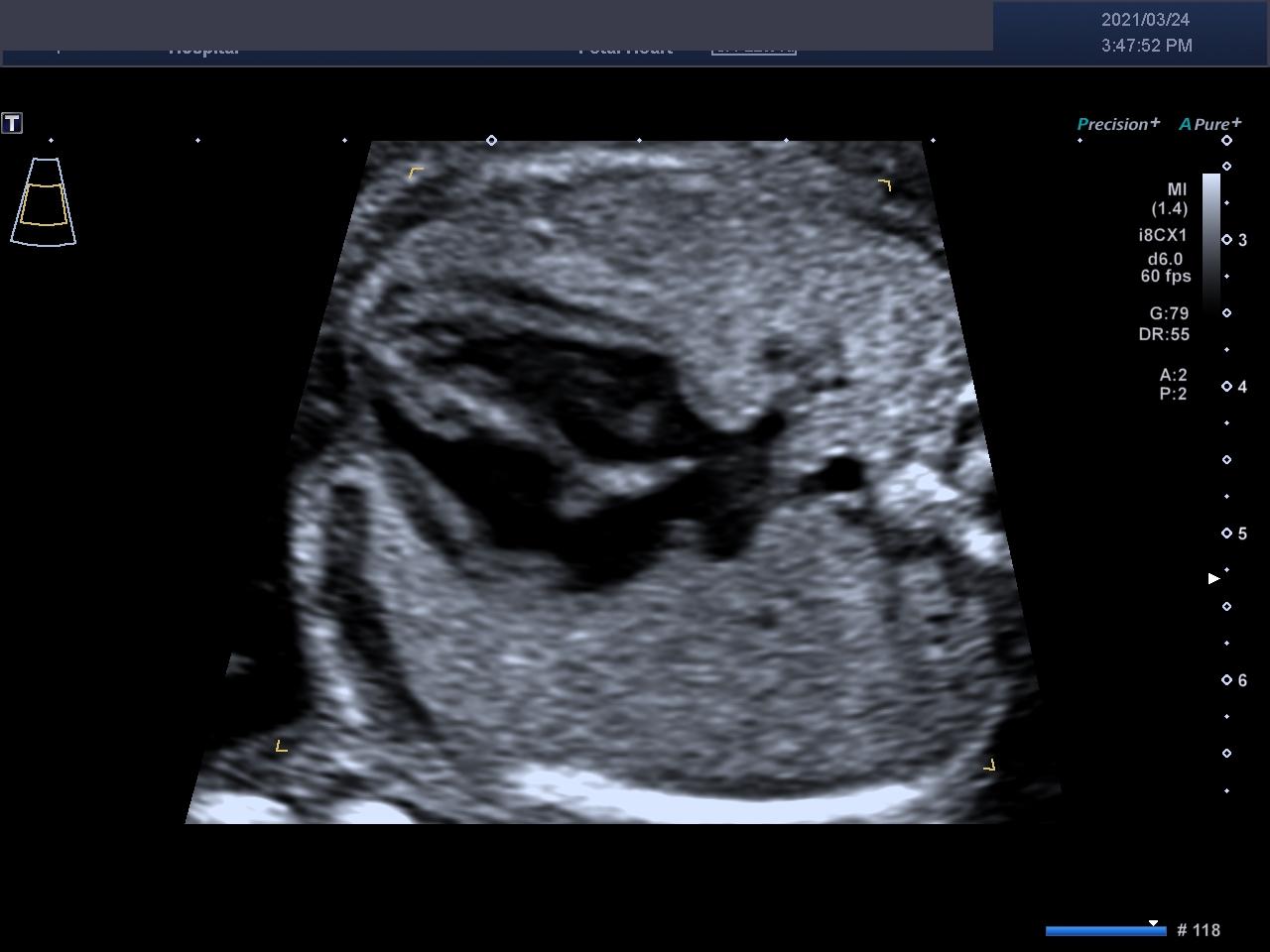

Supplement: S2 Dataset — (ZIP) [file pone.0305250.s002.zip › FE-SD-2/images/test_res/437_fc.jpg]

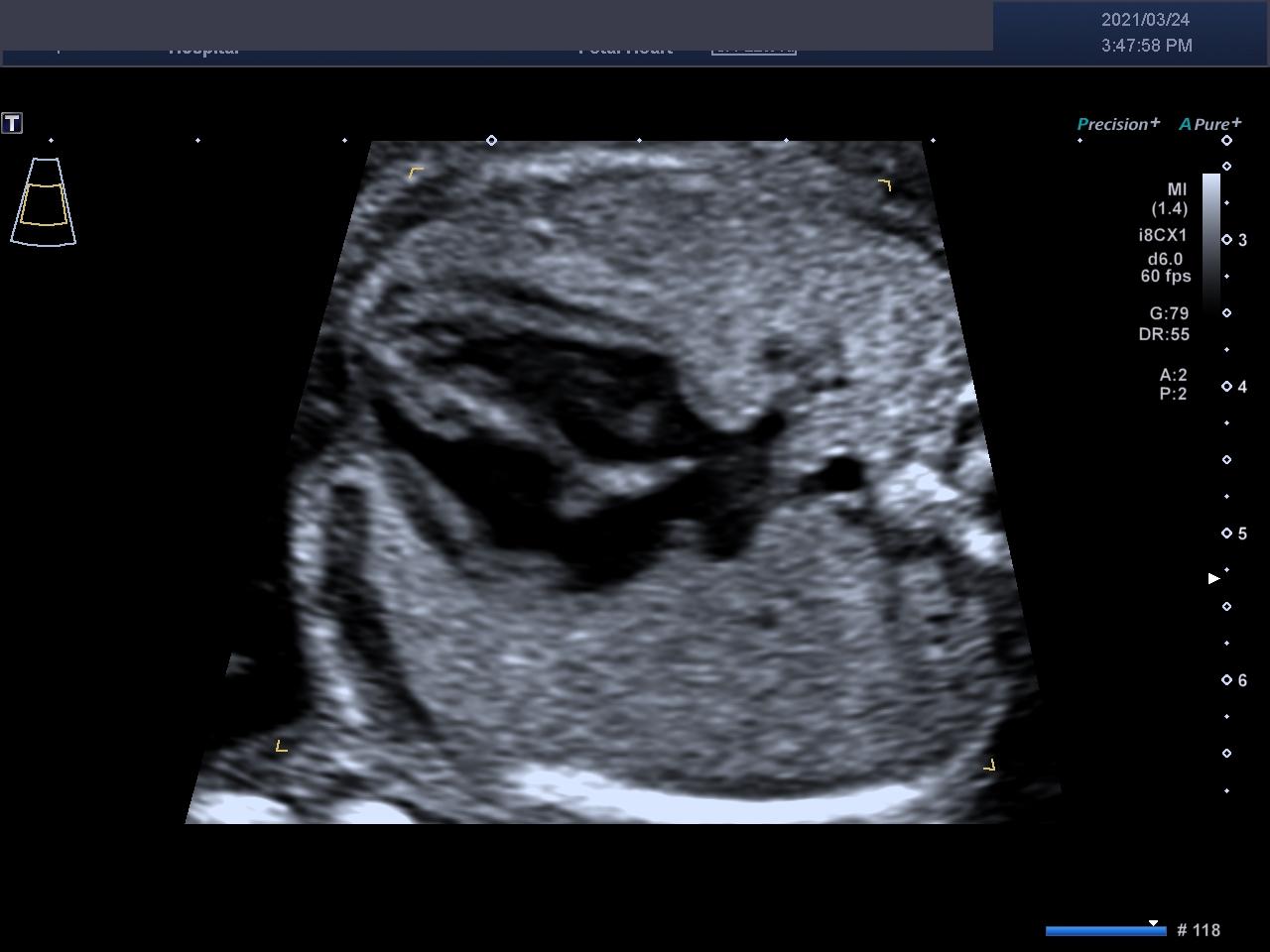

Supplement: S2 Dataset — (ZIP) [file pone.0305250.s002.zip › FE-SD-2/images/test_res/440_fc.jpg]

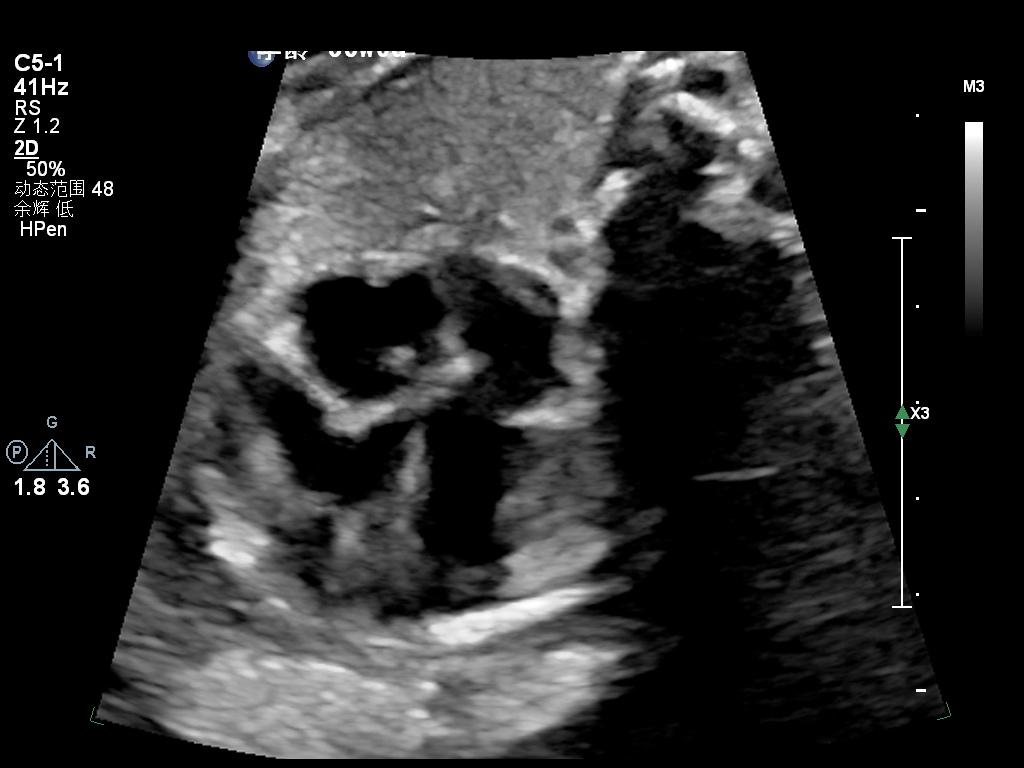

Supplement: S2 Dataset — (ZIP) [file pone.0305250.s002.zip › FE-SD-2/images/test_res/441_fc.jpg]

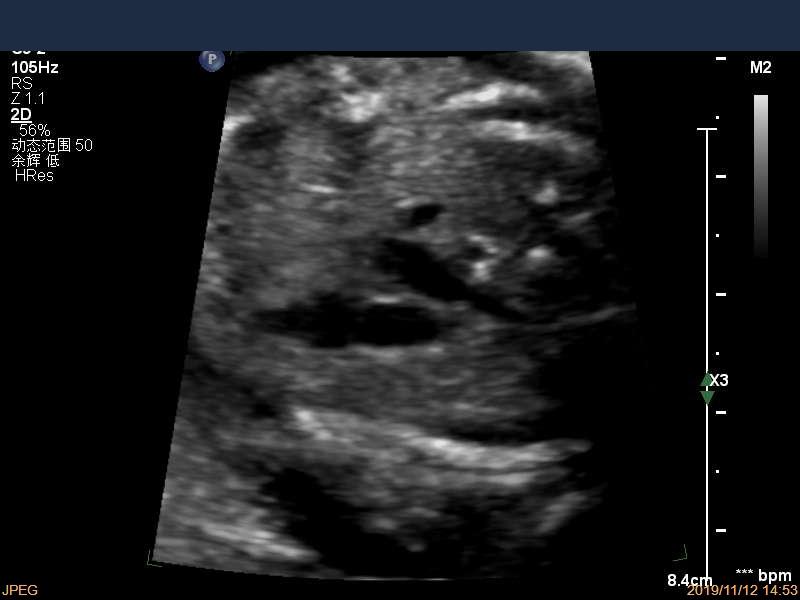

Supplement: S2 Dataset — (ZIP) [file pone.0305250.s002.zip › FE-SD-2/images/test_res/442_tv.jpg]

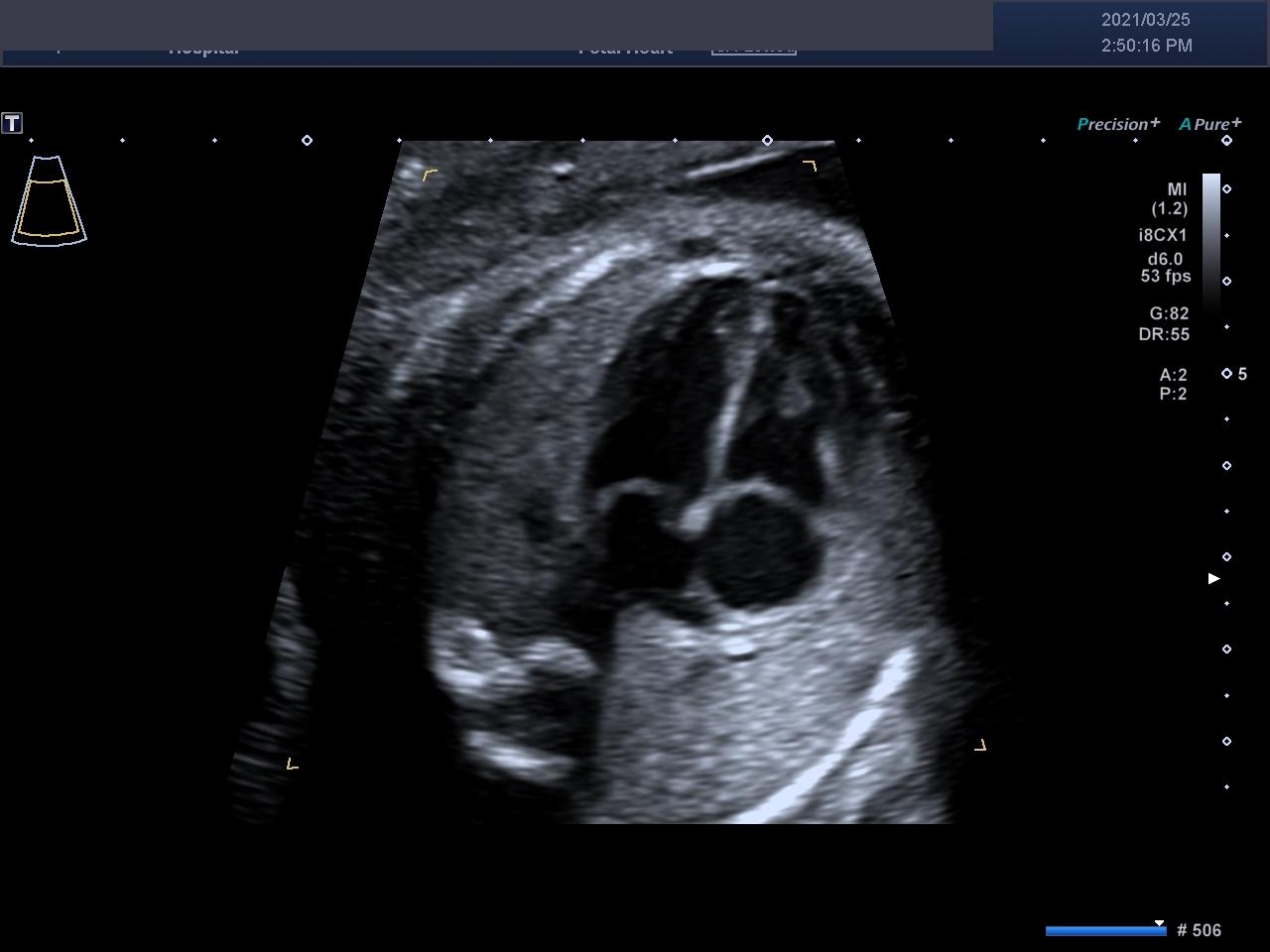

Supplement: S2 Dataset — (ZIP) [file pone.0305250.s002.zip › FE-SD-2/images/test_res/456_fc.jpg]

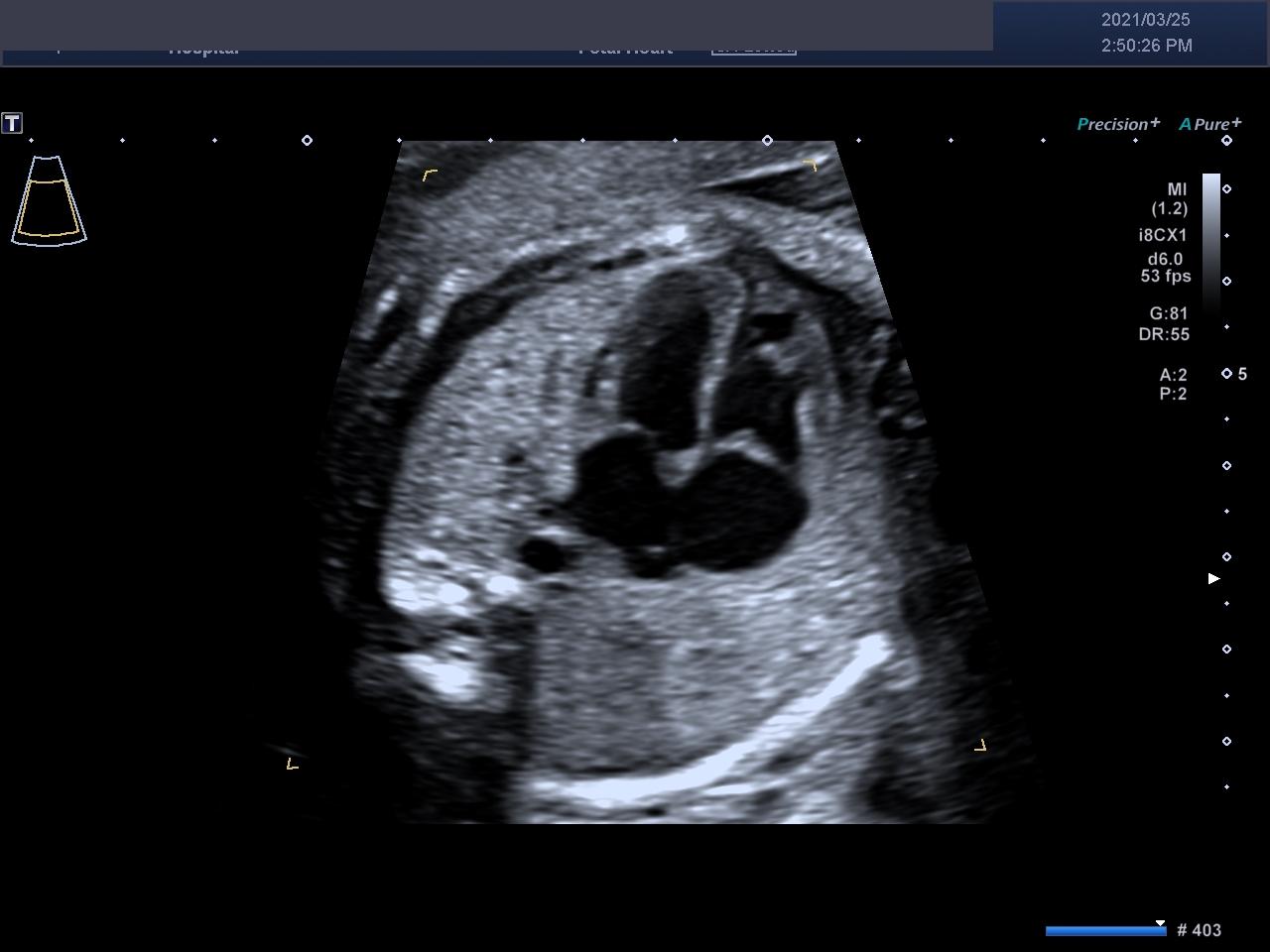

Supplement: S2 Dataset — (ZIP) [file pone.0305250.s002.zip › FE-SD-2/images/test_res/458_fc.jpg]

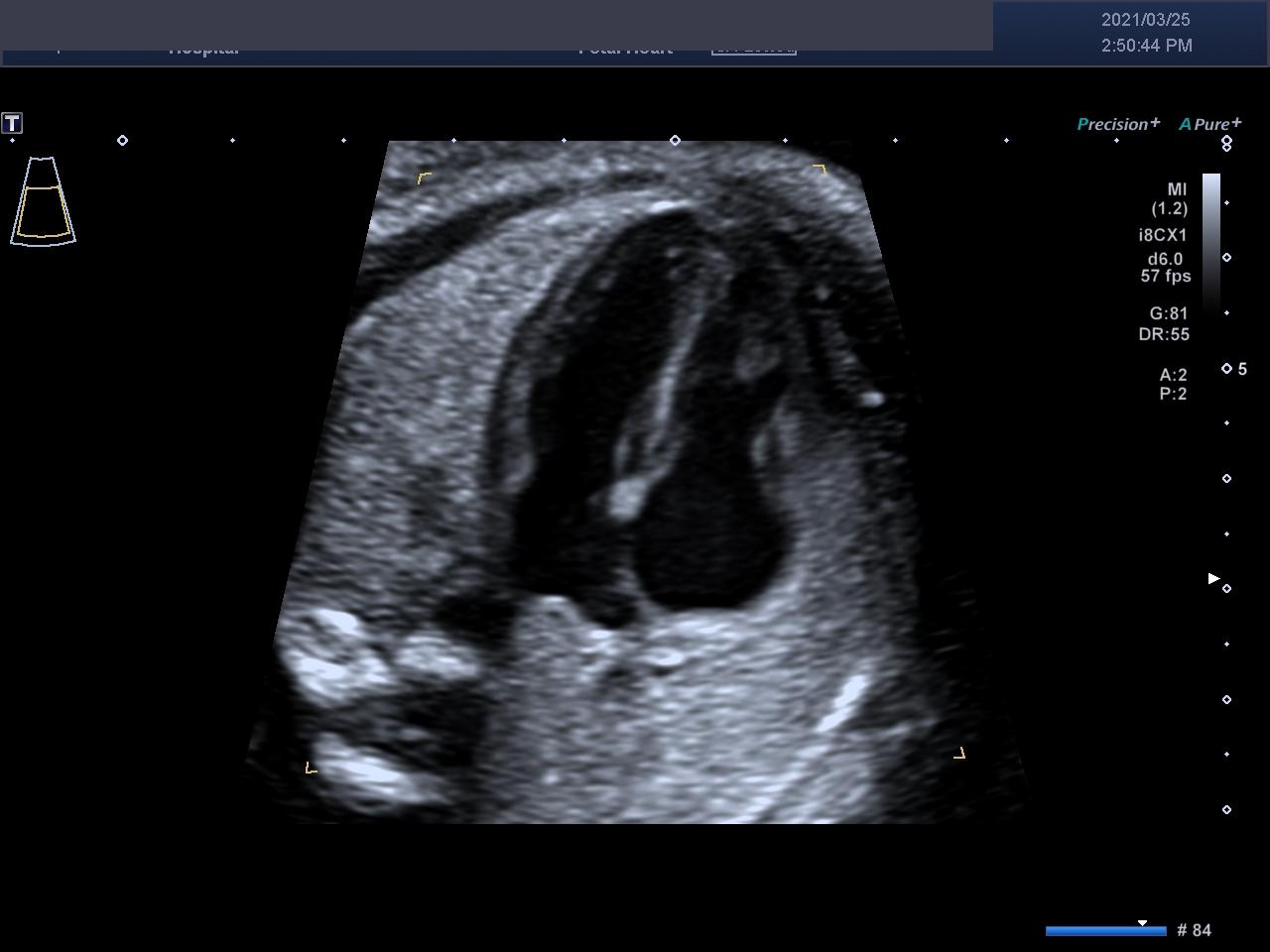

Supplement: S2 Dataset — (ZIP) [file pone.0305250.s002.zip › FE-SD-2/images/test_res/460_fc.jpg]

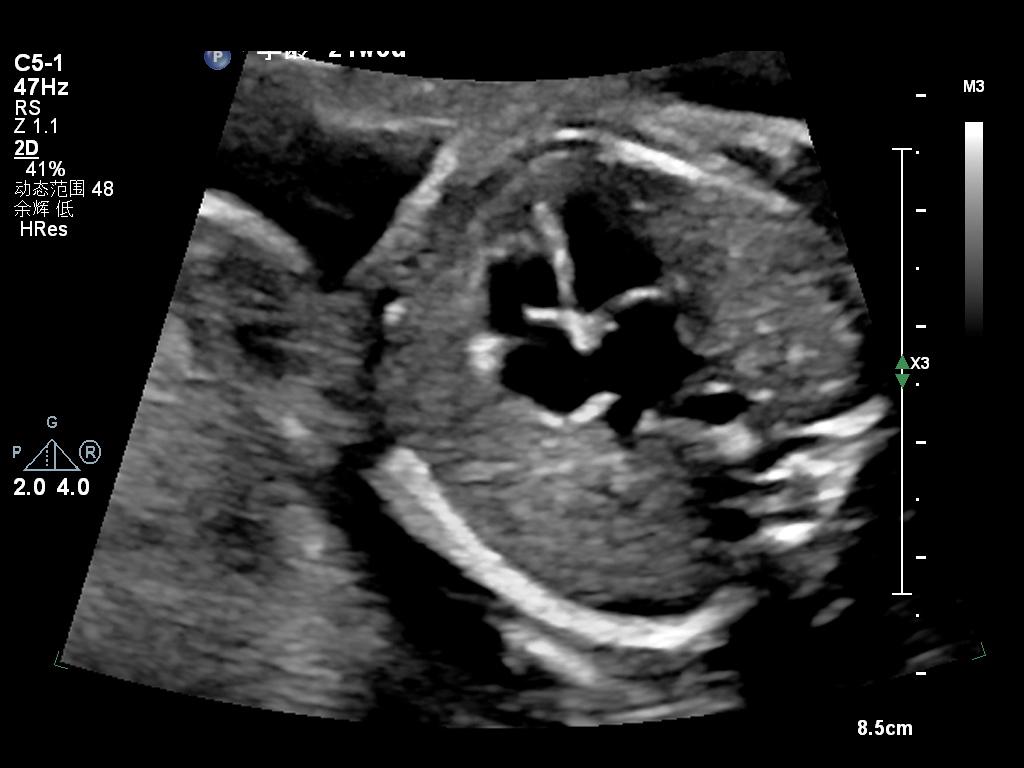

Supplement: S2 Dataset — (ZIP) [file pone.0305250.s002.zip › FE-SD-2/images/test_res/461_fc.jpg]

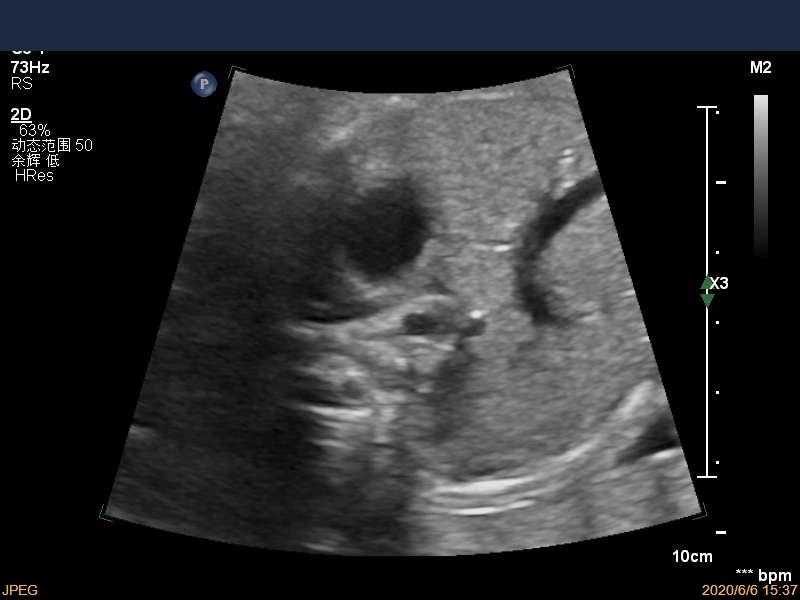

Supplement: S2 Dataset — (ZIP) [file pone.0305250.s002.zip › FE-SD-2/images/test_res/463_ab.jpg]

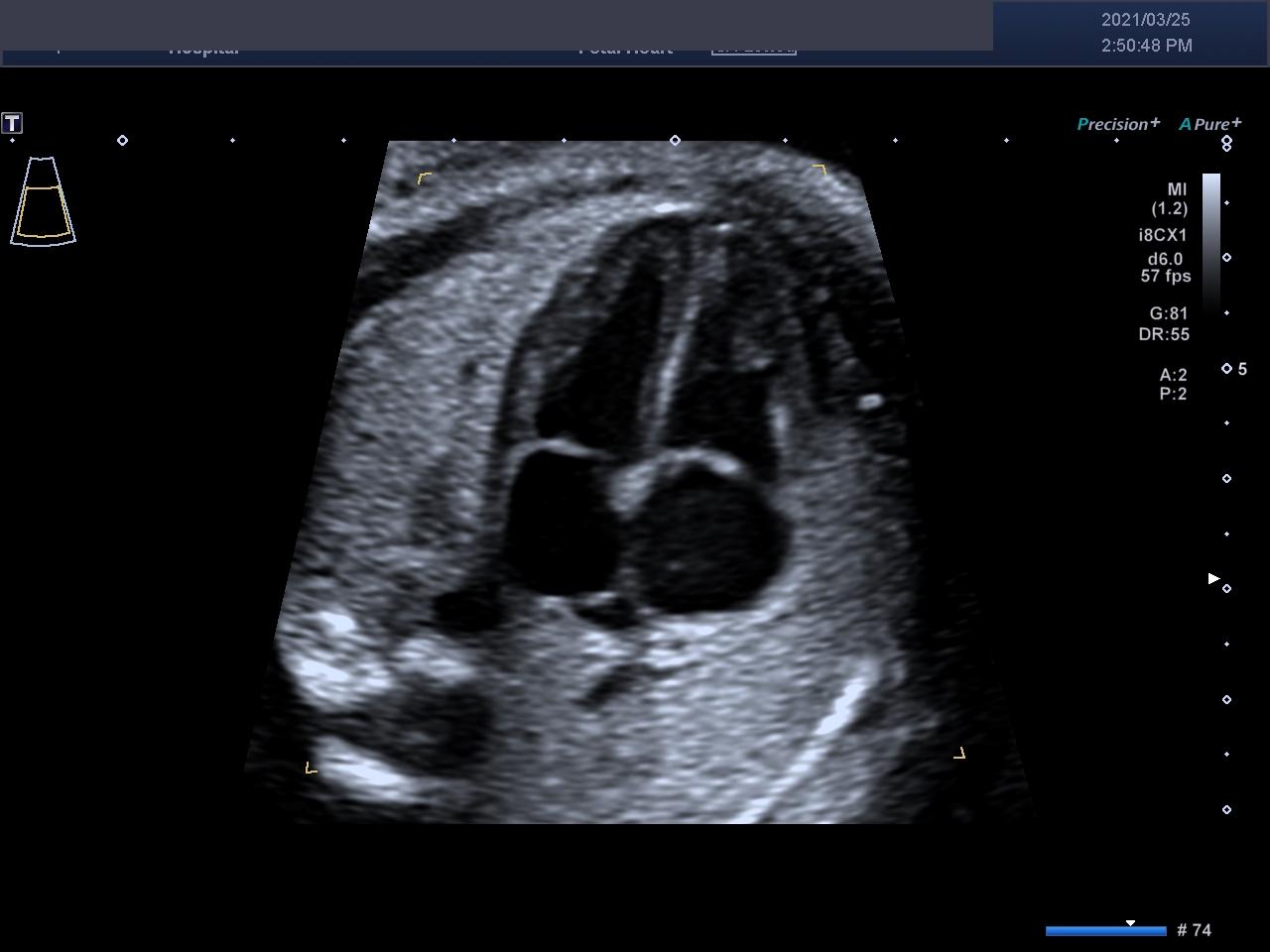

Supplement: S2 Dataset — (ZIP) [file pone.0305250.s002.zip › FE-SD-2/images/test_res/463_fc.jpg]

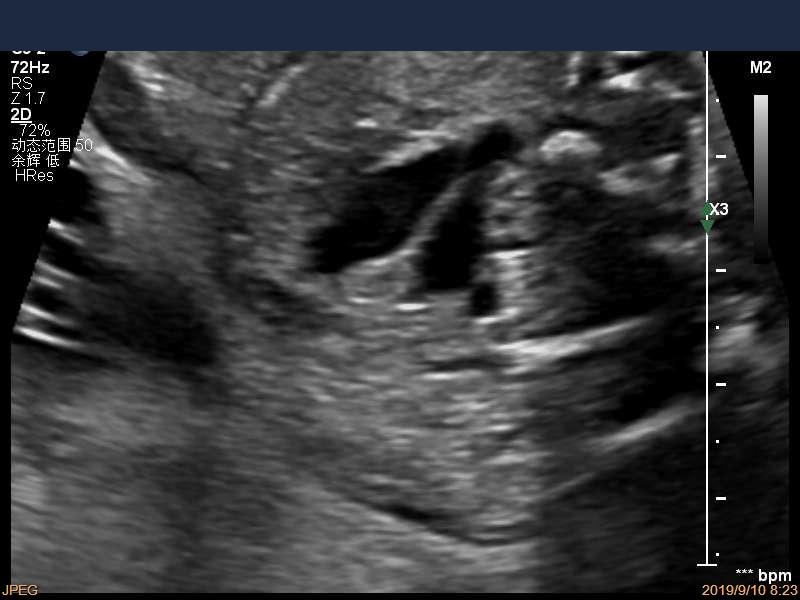

Supplement: S2 Dataset — (ZIP) [file pone.0305250.s002.zip › FE-SD-2/images/test_res/463_tv.jpg]

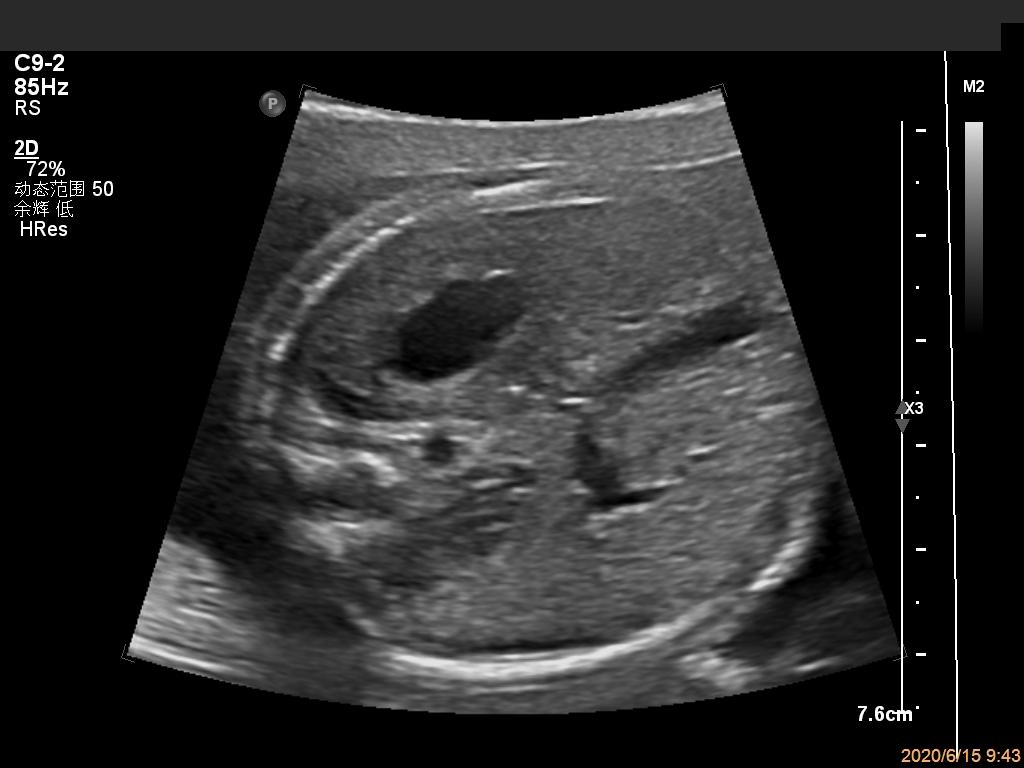

Supplement: S2 Dataset — (ZIP) [file pone.0305250.s002.zip › FE-SD-2/images/test_res/464_ab.jpg]

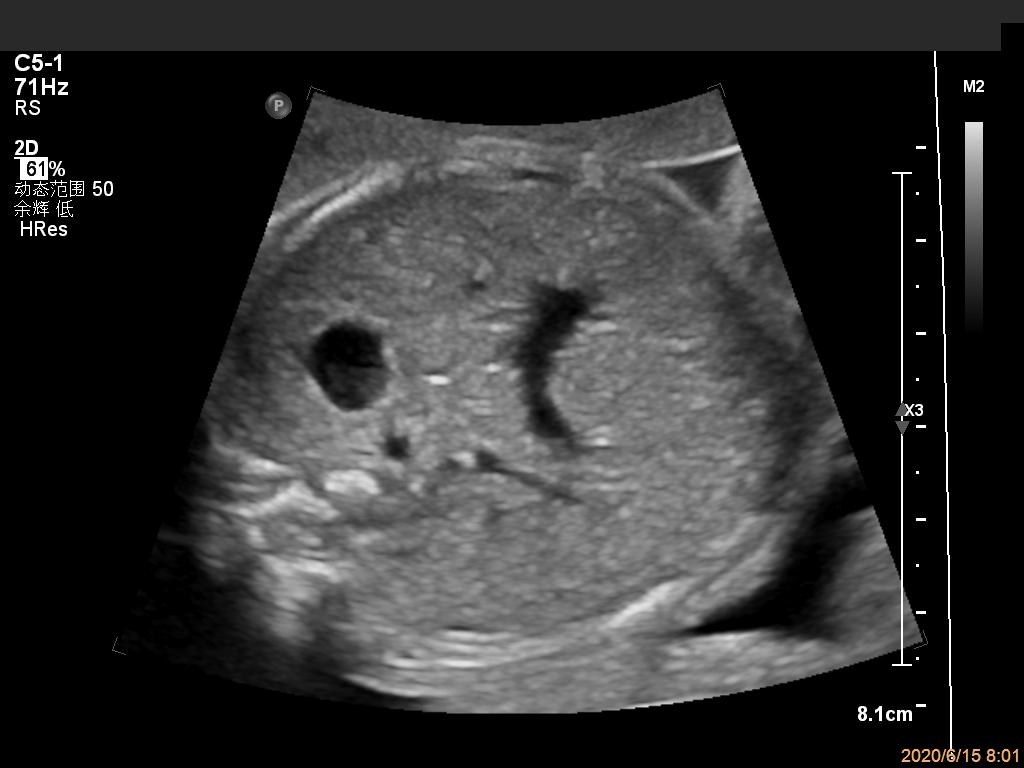

Supplement: S2 Dataset — (ZIP) [file pone.0305250.s002.zip › FE-SD-2/images/test_res/466_ab.jpg]

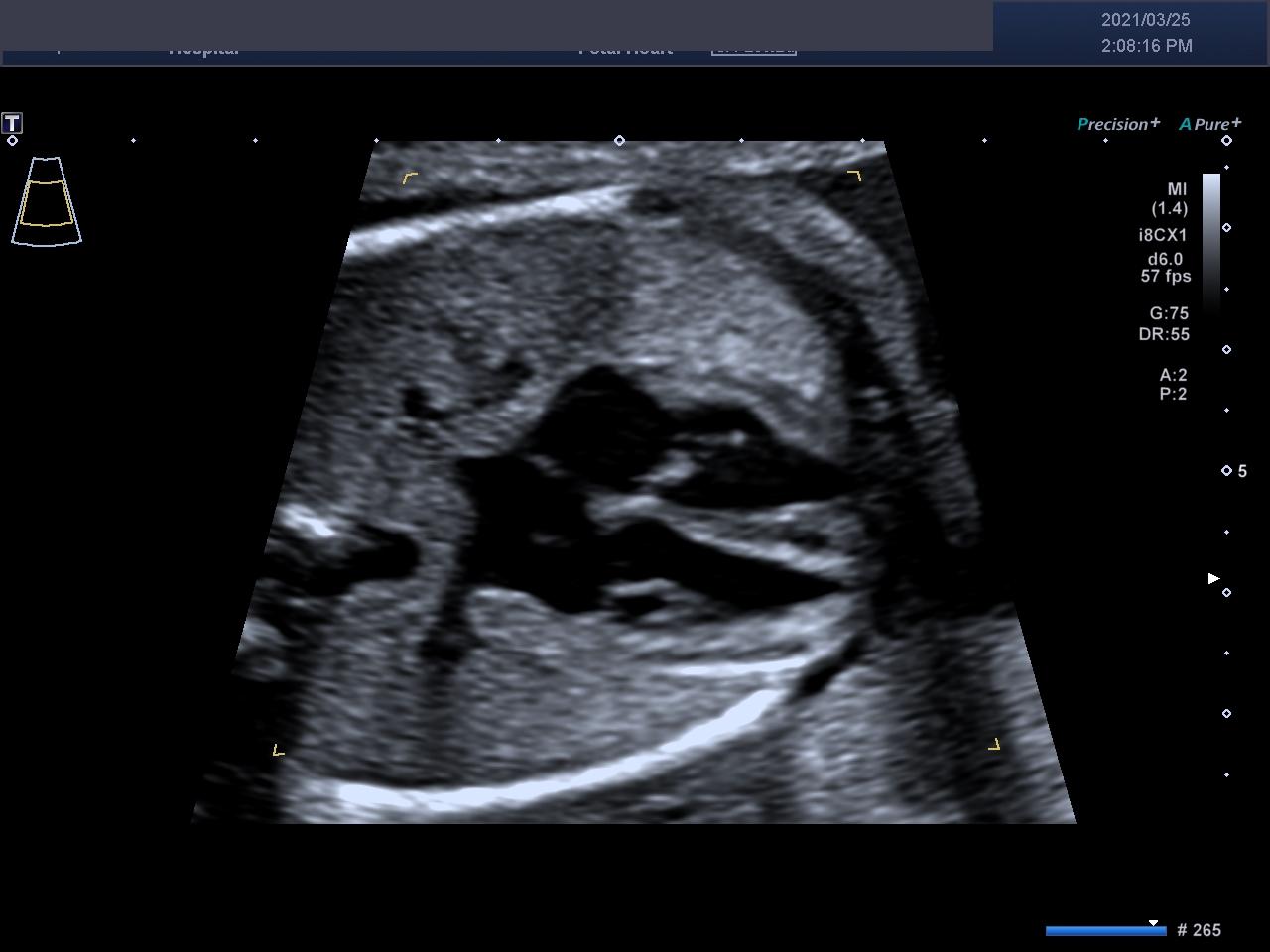

Supplement: S2 Dataset — (ZIP) [file pone.0305250.s002.zip › FE-SD-2/images/test_res/466_fc.jpg]

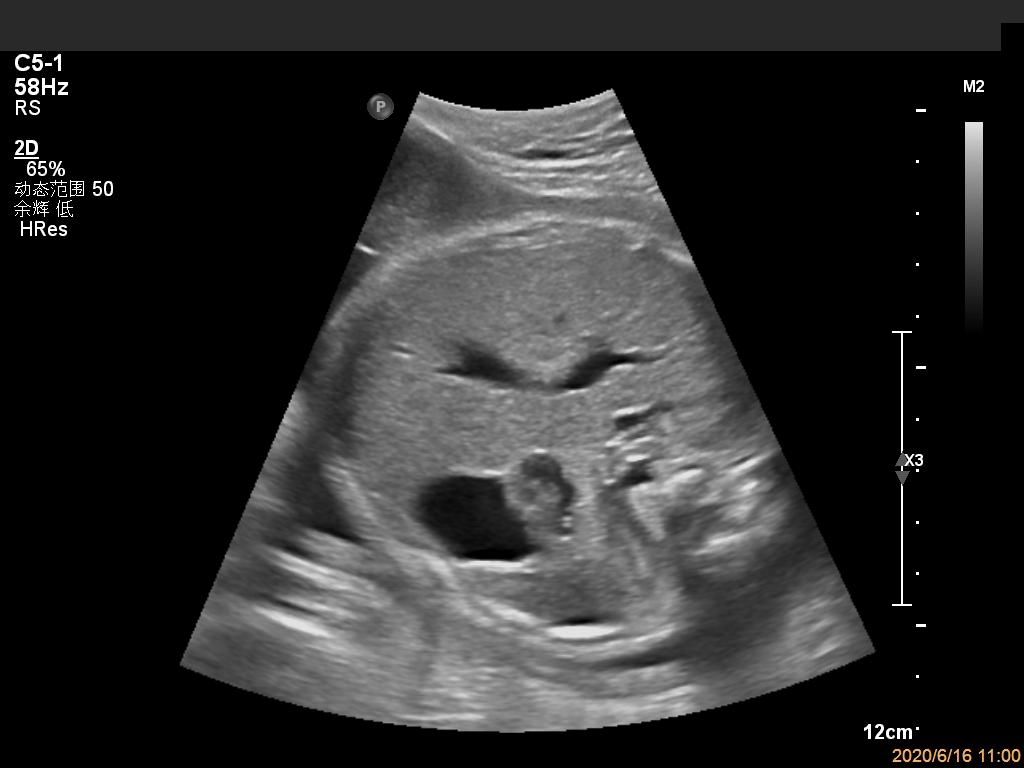

Supplement: S2 Dataset — (ZIP) [file pone.0305250.s002.zip › FE-SD-2/images/test_res/467_ab.jpg]

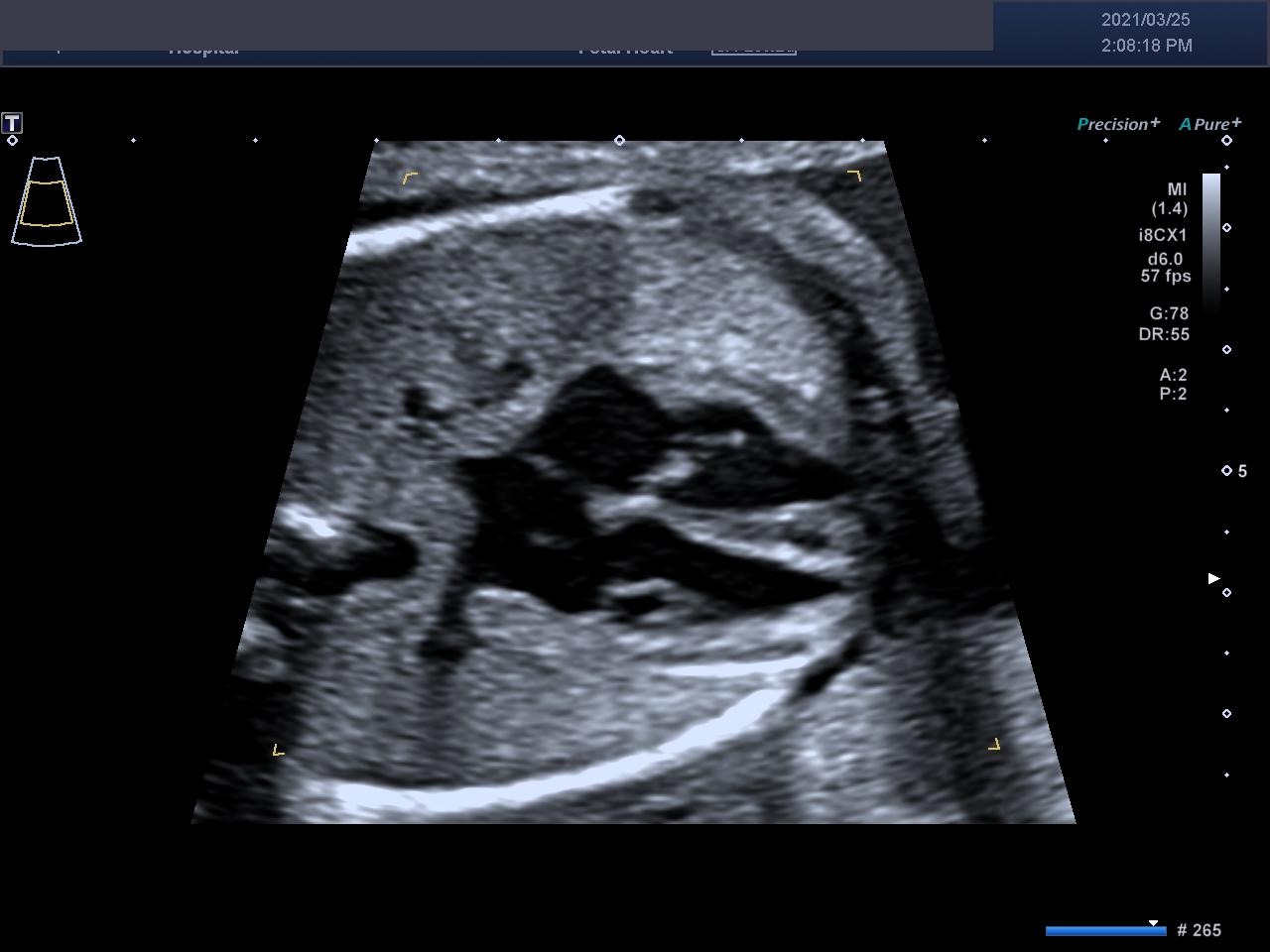

Supplement: S2 Dataset — (ZIP) [file pone.0305250.s002.zip › FE-SD-2/images/test_res/467_fc.jpg]

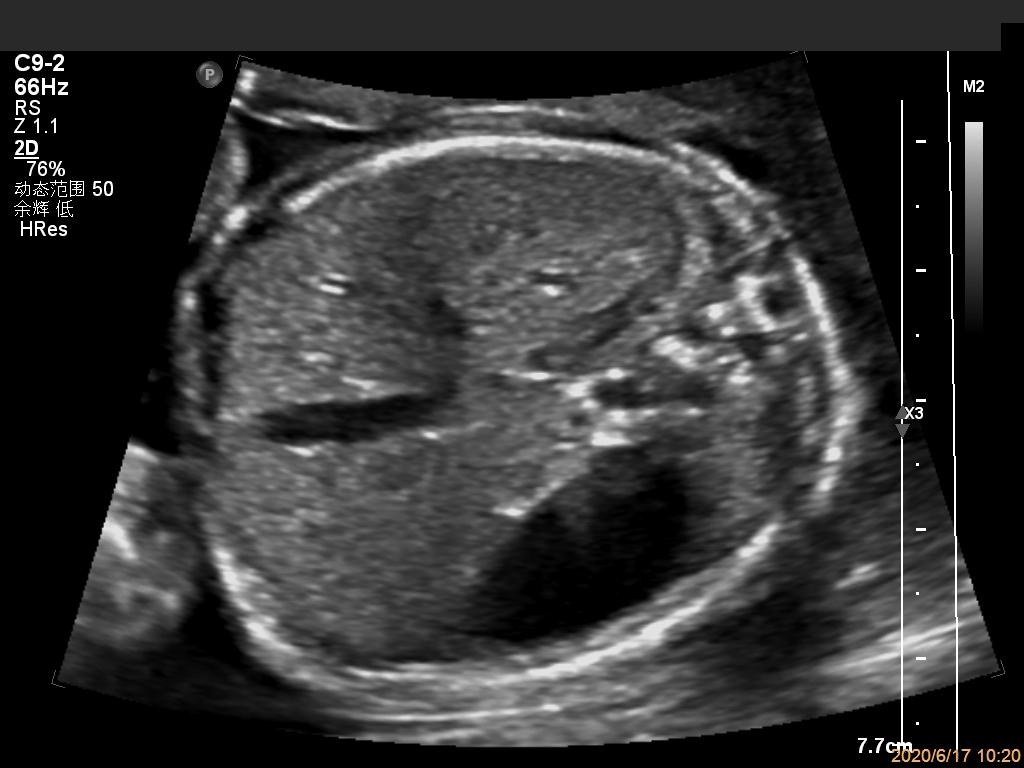

Supplement: S2 Dataset — (ZIP) [file pone.0305250.s002.zip › FE-SD-2/images/test_res/468_ab.jpg]

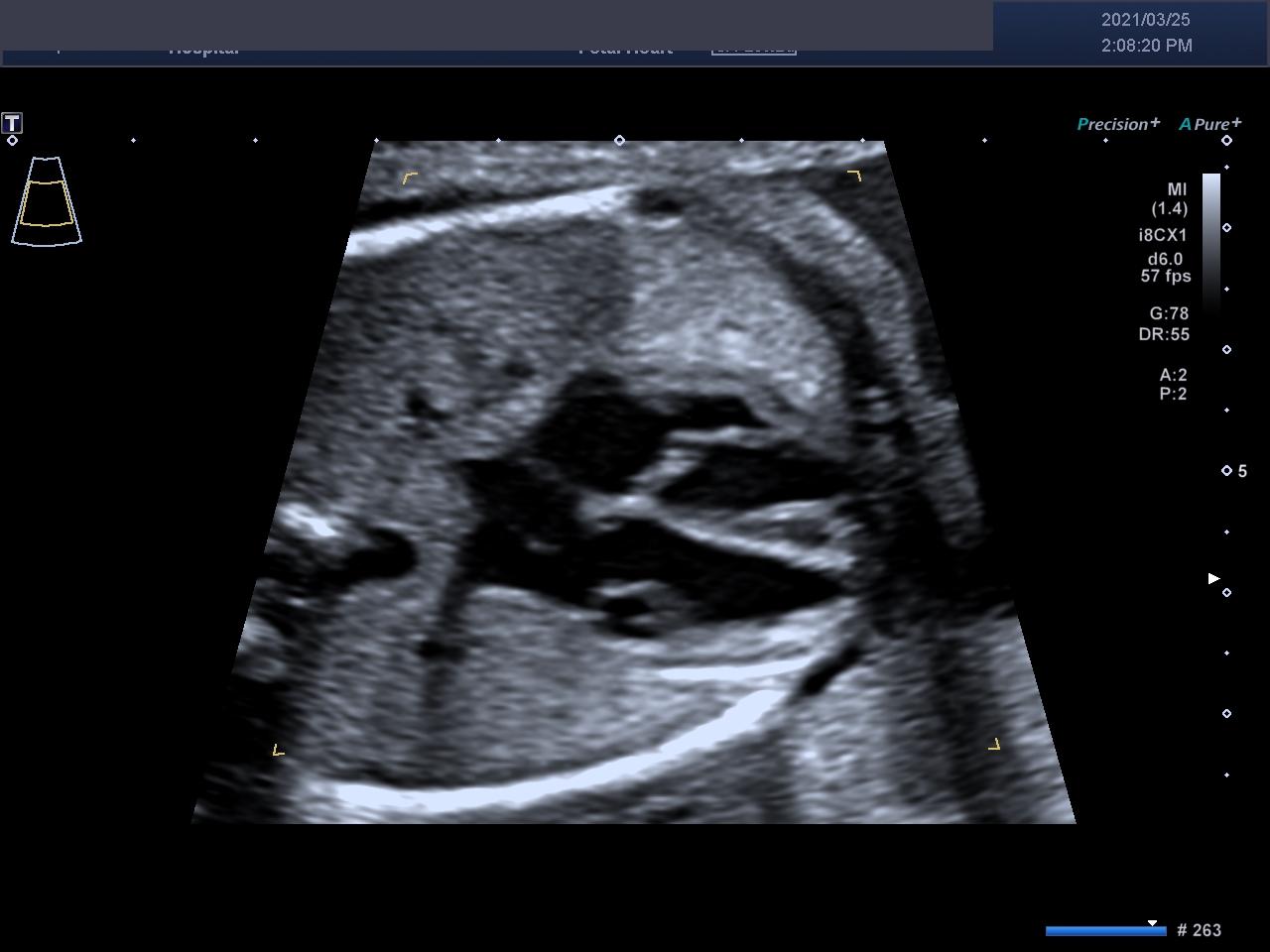

Supplement: S2 Dataset — (ZIP) [file pone.0305250.s002.zip › FE-SD-2/images/test_res/468_fc.jpg]
